# Gene Expression Program Underlying Tail Resorption During Thyroid Hormone-Dependent Metamorphosis of the Ornamented Pygmy Frog *Microhyla fissipes*

**DOI:** 10.3389/fendo.2019.00011

**Published:** 2019-01-25

**Authors:** Shouhong Wang, Lusha Liu, Jiongyu Liu, Wei Zhu, Yuta Tanizaki, Liezhen Fu, Lingyu Bao, Yun-Bo Shi, Jianping Jiang

**Affiliations:** ^1^Chengdu Institute of Biology, Chinese Academy of Sciences, Chengdu, China; ^2^Section on Molecular Morphogenesis, Eunice Kennedy Shriver National Institute of Child Health and Human Development (NICHD), National Institutes of Health (NIH), Bethesda, MD, United States; ^3^Department of Herpetology, Chengdu Institute of Biology (CIB), University of Chinese Academy of Sciences, Beijing, China

**Keywords:** SMRT sequencing, RNA-Seq, metamorphosis, *Xenopus*, thyroid hormone receptor, tail resorption, *Microhyla fissipes* (Neobatrachia)

## Abstract

Thyroid hormone (T3) is essential for vertebrate development, especially during the so-called postembryonic development, a period around birth in mammals when plasma T3 level peaks and many organs mature into their adult form. Compared to embryogenesis, postembryonic development is poorly studied in mammals largely because of the difficulty to manipulate the uterus-enclosed embryos and neonates. Amphibian metamorphosis is independent of maternal influence and can be easily manipulated for molecular and genetic studies, making it a valuable model to study postembryonic development in vertebrates. Studies on amphibian metamorphosis have been largely focused on the two highly related species *Xenopus laevis* and *Xenopus tropicalis*. However, adult *X. laevis* and *X. tropicalis* animals remain aquatic. This makes important to study metamorphosis in a species in which postmetamorphic frogs live on land. In this regard, the anuran *Microhyla fissipes* represents an alternative model for developmental and genetic studies. Here we have made use of the advances in sequencing technologies to investigate the gene expression profiles underlying the tail resorption program during metamorphosis in *M. fissipes*. We first used single molecule real-time sequencing to obtain 67, 939 expressed transcripts in *M. fissipes*. We next identified 4,555 differentially expressed transcripts during tail resorption by using Illumina sequencing on RNA samples from tails at different metamorphic stages. Bioinformatics analyses revealed that 11 up-regulated KEGG (Kyoto Encyclopedia of Genes and Genomes) pathways and 88 Gene Ontology (GO) terms as well as 21 down-regulated KEGG pathways and 499 GO terms were associated with tail resorption. Our findings suggest that tail resorption in *M. fissipes* and *X. laevis* shares many programs. Future investigations on function and regulation of these genes and pathways should help to reveal the mechanisms governing amphibian tail resorption and adaptive evolution from aquatic to terrestrial life. Furthermore, analysis of the *M. fissipes* model, especially, on the changes in other organs associated with the transition from aquatic to terrestrial living, should help to reveal important mechanistic insights governing mammalian postembryonic developments.

## Introduction

Thyroid hormone (T3) plays a critical role during vertebrate development. In mammals, the most important period of T3 action is the so-called postembryonic development, which is about 4 months before to several months after birth for human when plasma T3 level peaks (1, 2). Many important developmental changes take place during this period. Among them include brain development, organ maturation, the changes from fetal to adult hemoglobin, etc. Compared to embryogenesis, postembryonic development is poorly studied in mammals largely because of the difficulty to manipulate the uterus-enclosed embryos and neonates. On the other hand, defects during postembryonic development can lead to life-long diseases/abnormalities. Thus, alternative non-mammalian models are needed to understand this developmental period, especially the role of T3. Among vertebrates, frog metamorphosis bears the strong similarities with postembryonic development in mammals (1, 2). Like mammalian postembryonic development, frog metamorphosis is also characterized by a peak level of plasma T3. Furthermore, T3 plays a necessary and sufficient role for the transformation of a tadpole to a frog (3–6). Unlike mammalian development, frog metamorphosis is independent of maternal influence and can be easily manipulated by controlling the availability of T3 to the tadpoles. Furthermore, most, if not all, individual organs are genetically pre-determined to undergo organ-autonomous changes in response to T3, making it possible to induce metamorphosis in organ- and primary cell cultures with T3 treatment (1, 2, 7). These make frog metamorphosis a highly valuable model to study postembryonic development in vertebrates.

The most widely and best-studied frog models for metamorphosis are *X. laevis* and *X. tropicalis*, especially after the advent of transgenic and gene-editing technologies (8–11). Studies on the *Xenopus* models have yielded important insights on the roles of T3 and its two receptors, TRα and TRβ during development, and identified many T3-reponse genes and gene regulation profiles underlying metamorphosis in a number of organs and tissues (3, 4, 7, 12–17). On the other hand, it remains to be investigated if the findings from the *Xenopus* models apply to other frogs. This had been difficult due to the lack of genome sequence information for other frog species. Although a genome-wide transcriptome analysis for the developing tadpoles of the northern leopard frog (Lithobates pipiens) was reported but the study did not analyze changes during metamorphosis (18). Thus, it is important to study the gene expression program during metamorphosis in other frog species, especially considering that adult *X. laevis* and *X. tropicalis* remain aquatic.

*Microhyla fissipes* offers a number of advantages as an alternative model for developmental and genetic studies. *M. fissipes* is a typical anuran from the family of Microhylidae belonging to the Neobatrachia while *Xenopus* is a representative of Mesobatrachia (19). Studies on *M. fissipes* will thus allow a comparison between two different genuses to reveal adaptive mechanisms from aquatic to terrestrial life (19). Compared to the two *Xenopus* species, *M. fissipes* animals are much smaller in body size and have a shorter developmental time through metamorphosis (20, 21). *M. fissipes* is a diploid anuran and has large egg size (0.8–1.0 mm) (19), making it easy to adapt gene-editing tools for knockout studies of gene function during development. Most importantly, *M. fissipes* metamorphosis changes an aquatic tadpole to a terrestrial frog, better resembling postembryonic development in mammals. Thus, while many metamorphic changes, such as tail resorption and limb development, are expected to be conserved between *Xenopus* and *M. fissipes*, others that are critical for terrestrial living, such as the transformation of the lung and skin, may diverge.

To investigate whether we can use global gene expression analysis to study *M. fissipes*, we have made use of the advances in sequencing technologies to investigate the gene regulation profiles underlying the tail resorption program during metamorphosis in *M. fissipes*. Tail was chosen for this study in part due to easiness to characterize the changes at morphological and cellular level and in part due to available information from earlier studies on *Xenopus*. We have used Illumina RNA sequencing (RNA-Seq) combined with single molecule real-time (SMRT) sequencing for transcriptome assembly on RNA isolated from *M. fissipes* tails at different developmental stages. This allowed us to obtain 50,577 expressed transcripts and identified 4,555 differentially expressed transcripts (DETs) during tail resorption. We further analyzed the enriched Gene Ontology (GO) terms and Kyoto Encyclopedia of Genes and Genomes (KEGG) pathways among the DETs. Our finding revealed similar gene regulation programs underlying tail resorption in both *M. fissipes* and *Xenopus*. Future studies on *M. fissipes*, especially on the metamorphosis of tissues critical for terrestrial living, should help to reveal the mechanisms governing the adaptive evolution from aquatic to terrestrial life.

## Materials and Methods

### Experimental Animals

*Microhyla fissipes* breeding adults were collected from Shuangliu, Chengdu, China (30.5825°N, 103.8438°E). All animal care and treatments were done as approved by Animal Care Committee, Chengdu Institute of Biology (Permit Number: 20150121003). Fertilized eggs were obtained from one pair of frogs, incubated in glass petri dishes (diameter, 160 mm) with dechlorinated tap water. After hatching, every 60 tadpoles was transferred to a plastic container (420 × 300 × 230 mm^3^) with 80 mm depth dechlorinated tap water at 25 ± 0.6 °C, and tadpoles were fed with spirulina powder once daily. All animals were maintained under the 12: 12 h light: dark cycle. Developmental stages were determined as described (21).

### Tissue Collection

Six tadpoles at stage 39 (S39), S40, S41, and S43 each were randomly collected and anesthetized in 0.01% MS222 for tail morphological measurement. Three animals at each stage were used for hematoxylin-eosin (HE) staining and the rest were used for terminal deoxynucleotidyl-transferase mediated dUTP nick end labeling (TUNEL) analysis. For gene expression analysis, three biological replicates, each containing one animal tail, were used for RNA-seq at each developmental stage. To maximize the discovery of *M. fissipes* gene pools, heart, liver, spleen, lung, kidney, skin, ovary, testis from adult *M. fissipes*, tails (at S38, S40, S41, S43) and dorsal muscle (at S36, S40, S43, S45) were dissected for SMRT sequencing. All dissected tissue samples were immediately frozen in liquid nitrogen and stored at −80°C.

### Morphological Measurements and Histological Analysis

#### Length Measurement

Tail length (TL, from posterior edge of vent to the end of tail tip) and snout-vent length (SVL) were measured with a stereo microscope (JSZ8T, Jiang Nan Yong Xin, Nanjing, China) and Mshot Image Analysis system (Mc50-N) as described (21). To reduce allometric bias, tail length at each stage were presented as a ratio of TL/SVL.

#### HE Staining

After length measurement, tails were dissected and fixed in 4% paraformaldehyde for 24 h, and then embedded in paraffin. Next, tails were cross-sectioned at 5 μm with a Rotary Microtome (Leica, RM 2016, Germany), and tissue sections were stained with HE.

### TUNEL Assay

Apoptotic cells in the tail during metamorphosis were detected by using a TUNEL detection kit (Roche, 11684817910) according to manufacturer's instruction.

### Illumina RNA Sequencing (RNA-Seq)

Total RNA was extracted using TRIzol (Invitrogen, Carlsbad, CA, USA) and DNase I (Sigma, St. Louis, MO, USA) was used to remove DNA. RNA concentration was measured using Qubit 2.0 Fluorometer (Life Technologies, CA, USA) and RNA purity was checked by using Nanodrop 2000 Spectrophotometer (IMPLEN, CA, USA). Additionally, the integrity of RNA (RIN) was determined using Agilent Bioanalyzer 2100 system (Agilent Technologies, CA, USA) with RIN>8.0. Briefly, mRNA was purified from total RNA by using poly-T oligo-attached magnetic beads and chemically fragmented. First strand cDNA was synthesized by using random hexamer primers and M-MuLV Reverse Transcriptase (RNase H–). Second strand cDNA synthesis was subsequently performed by using DNA Polymerase I and RNase H. Twelve cDNA libraries were generated by using the TruSeq RNA Sample Preparation Kit (Illumina, San Diego, CA, USA) following the standard protocols. The libraries were sequenced on the Illumina X-ten platform to obtain 150 nt paired-end reads at NovoGene (Beijing, China). All the raw data from Illumina sequencing were deposited in the NCBI Short Read Archive (SRA) database with the accession number PRJNA504611.

### SMRT Sequencing

Tissue samples were homogenized and total RNA was isolated. The concentration and quality of RNA were assessed as above for RNA-Seq. The first full length strand cDNA was synthesized from 3 μg total RNA by using the SMARTer PCR cDNA Synthesis Kit (Clontech, Mountain View, CA, USA). After PCR cycle optimization, the double-strand cDNA was generated with 11 PCR cycles, and the PCR products were separated with agarose gel-based size selection into cDNA fractions of length 1–2, 2–3, and 3–6 kb. Then, large-scale PCR was utilized to amplify the cDNAs. In the end, three SMRTbell template libraries were generated from the amplified cDNA by using Pacific Biosciences template preparation kit (Menlo Park, CA, USA) as per the standard protocol. Then SMRT sequencing was performed on a PacBio RS II platform at NovoGene (Beijing, China). Full-length transcripts were obtained by using Pacific Biosciences' SMRT Analysis Server 2.0 (22). Finally, full-length transcripts were corrected with Proovread Software (23) by using Illumina Hiseq reads. The proofread-corrected sequences after removing the redundant sequences with using CD-HIT-EST (24) were used as the reference sequences for further analyses. All the raw data from Illumina sequencing were deposited in the NCBI Short Read Archive (SRA) database with the accession number PRJNA504611.

### Functional Annotation of Transcripts

The final corrected clean reads above were searched against the databases NR, Swiss-Prot and KOG by using diamond software version 0.8.22 with an E-value threshold of 1.0E-5 and KOG with an E-value threshold of 1.0E-3 to predict the gene identities. NT annotation was done with NCBI blast 2.2.28^+^ software version 2.2.28^+^ with an E-value threshold of 1.0E-5. Hmmer 3.0 package hmmscan was used for PFAM annotation with an E-value threshold of 0.01. GO annotations were determined based on the best BLASTX hit from the NR database using the Blast2GO software version 2.5 (*E*-value = 1.0E−6). KEGG pathway analyses were performed using the KEGG Automatic Annotation Server (KAAS, *E*-value = 1.0E−10). Clean reads were mapped to the full length reference transcripts by using Bowtie, and then gene expression level was calculated with RSEM (RNA-Seq by expectation-maximization) (25).

### DET Analysis

To compare the transcript expression levels among the four stages, the reads of each transcript were normalized to yield the number of sequenced fragments per kilobase of transcript sequence per millions base pairs sequenced (FPKM) for the four developmental stages. Pairwise comparisons of transcript expression levels among the four stages were carried out by using the DESeq R package (1.10.1) to identify DETs. Transcripts were considered as DETs at *q* value < 0.05 after adjustment for the false discovery rate (FDR). For heatmap analysis, log${}_{10}^{\left(\text{FPKM}+1\right)}$ values were used for each tested transcript. Gene expression profile and log2 (ratios) values were used for K-means clustering.

### GO and KEGG Enrichment Analysis of DETs

DETs were subjected to GO enrichment analysis by using the GOseqR package based on the Wallenius non-central hyper-geometric distribution (26). KEGG pathway enrichment analysis of the DETs was done with the KOBAS software (27).

## Results

### *Microhyla fissipes* as a Model for Studying Tail Resorption

**Figure 1:**
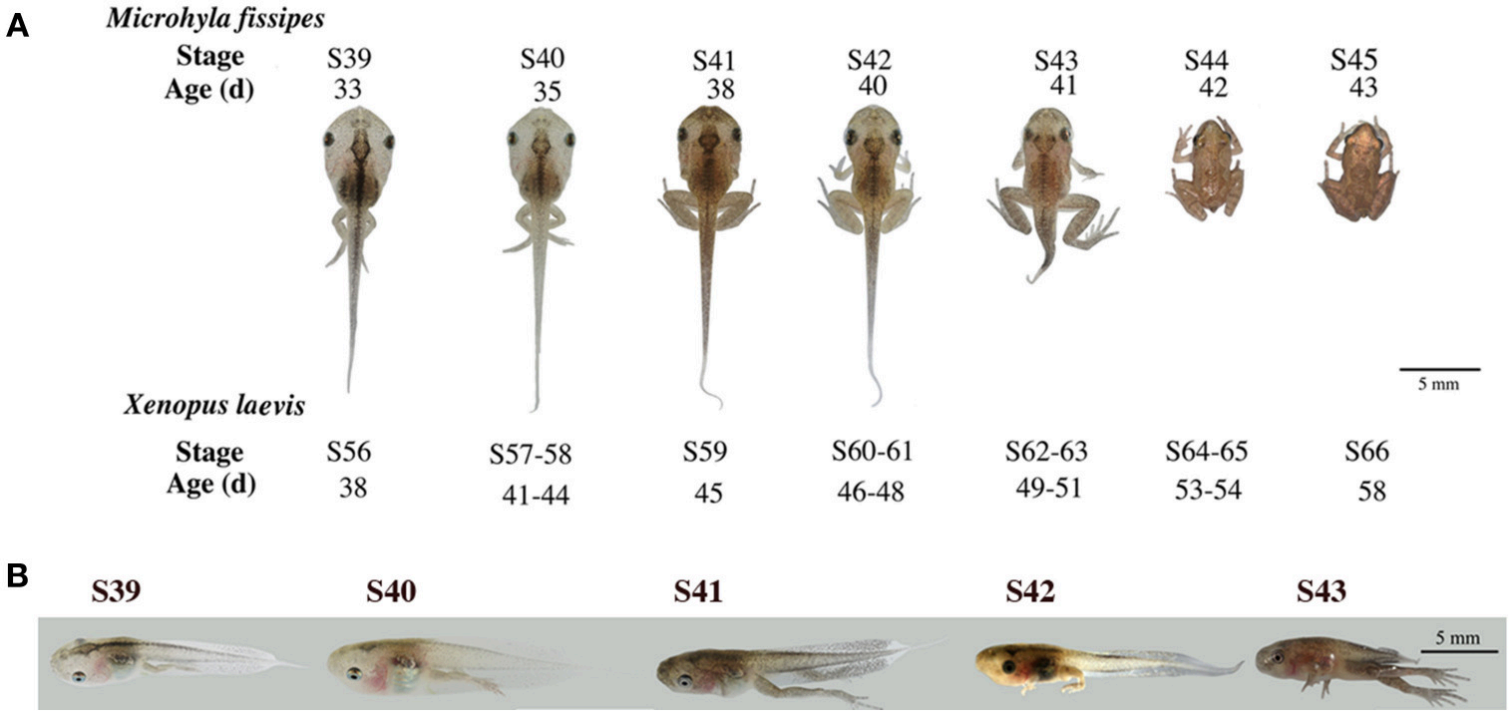
Morphological changes associated with tail resorption during *M. fissipes* metamorphosis. **(A)** A representative *M. fissipes* animal at indicated developmental stages from S39 (around the onset of metamorphosis) to S45 (end of metamorphosis) is shown together with typical age (in days, d) of the animal when reared at 22.9–25.4°C. Shown at the bottom are the corresponding stages of *X. laevis* animals and their ages when reared at 22–24°C (28), scale bar: 5 cm. **(B)** Lateral views of the tail at indicated developmental stages.

Studies on *X. laevis* indicate that tail resorption takes place mainly after stage 62 (S62) when rapid reduction in tail length occurs (28). This process involves apoptosis in essentially all tail tissues, especially, the epidermis, muscle, and notochord (29–31). To determine if *M. fissipes* undergoes a similar tail resorption process, we first compared the morphology of *M. fissipes* tadpoles at different stages of metamorphosis to that of *X. laevis* tadpoles (28). As shown in Figure 1, *M. fissipes* tadpoles at S39 resemble *X. laevis* tadpoles at S56, considered to be the early phase of metamorphosis (with stage 54/55 typically considered the onset of metamorphosis in *X. laevis*) (28). Under comparable rearing temperatures, *M. fissipes* tadpoles at S39 complete metamorphosis (reaching S45) in about 10 days, about ½ of the time that it takes for *X. laevis* tadpoles at S56 to reach the end of metamorphosis when tail resorption is complete (Figure 1). In particular, tail resorption, as defined from the onset of tail length reduction to complete resorption, requires only 3 days for *M. fissipes* tadpoles (from S42–S45) but 10 days for *X. laevis* tadpoles (from S61–S66) (Figure 1), indicating that *M. fissipes* tadpoles undergo a faster T3-dependent metamorphosis.

**Figure 2:**
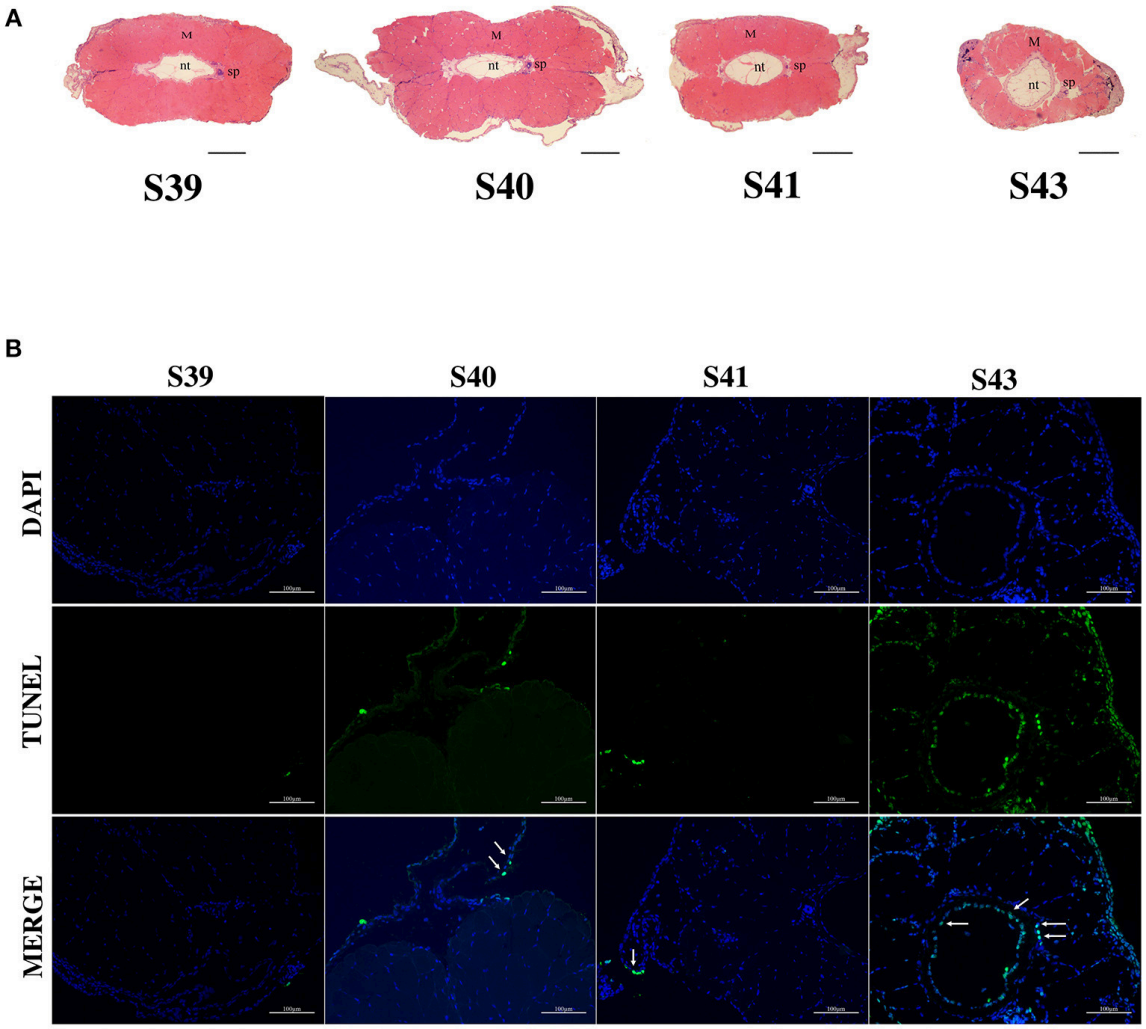
Extensive apoptosis in the epidermis and notochord is associated with tail resorption during natural metamorphosis. **(A)** H&E staining of tail cross-sections at indicated stages. Magnification, 40×, scale bar: 200 μm, M, muscle; nt, notochord; sp, spinal cord. **(B)** TUNEL labeling reveals apoptotic cells mainly in the epidermis and notochord at S43 when tail length reduces rapidly (see **A**). Tail sections at indicated stages were co-stained with TUNEL labeling (green) for apoptotic cells (indicated by white arrows) and DAPI (blue) for DNA. Magnification, 200×, scale bars: 100 μm.

We next isolated tails from animals at 4 different metamorphic stages, S39, S40, S41, and S43 for further analysis. *M. fissipes* S39 is equivalent to *X. laevis* S56 (Figure 1). The only noticeable metamorphic change that takes place at this stage is the hindlimb development (28). For this reason, *X. laevis* at S56 is often referred to as a premetamorphic stage and for simplicity, we will refer *M. fissipes* tadpoles at S39 as premetamorphic animals as well. S40–S43 are metamorphic climax stages, although tail length reduction occurs only after S41. When tail sections at these different metamorphic stages were analyzed, it was clear that significant muscle atrophy was observed only at S43 (Figure 2A). In addition, TUNEL assay showed that while some apoptotic cells were observed by S40/41, drastic increase in apoptotic cells were found at S43 (Figure 2B), consistent with rapid tail length reduction. These findings indicate that *M. fissipes* undergoes a similar tail resorption program as that during *X. laevis* tail metamorphosis (29–31).

### Transcript Assembly and Identification of DETs During Tail Resorption

To facilitate the genome-wide analysis of the gene expression program underlying tail resorption, we first carried out SMRT sequencing of different *M. fissipes* organs at different stages. From 108,614,218 nucleotide sequences thus obtained, a total of 67,939 transcripts were assembled. The lengths of these assembled transcripts ranged from 103 to 8,847 bp with an average length of 1,599 bp and N50 of 1,956 bp (Table S1). Annotations through NR, NT, KEGG, Swiss-Prot, PFAM, GO and KOG databases showed that 52,881 transcripts (78% of the 67,939 transcripts) were annotated in at least one of the databases (Table S1).

**Figure 3:**
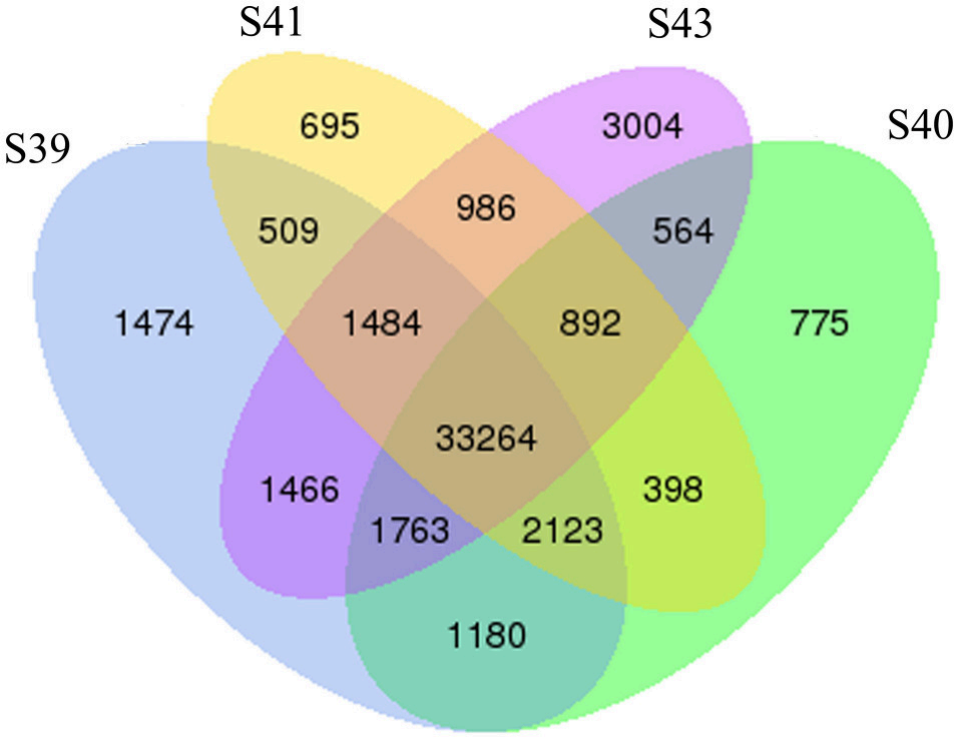
Venn diagram analyses reveal stage-specific of gene expression. Venn diagrams showing the number of expressed transcripts (FPKM > 0.3) at different stages in the tail during natural metamorphosis. Note that total 50,577 transcripts were expressed in which 33,264 transcripts were expression at all four stages and that each stage had some uniquely expressed transcripts, with S43 having the most. FPKM, fragments per kilobase of exon model per million mapped reads.

To determine the gene expression program underlying tail resorption, we carried out Illumina RNA-Seq on the tail at the four metamorphic stages (S39, S40, S41, S43). The gene expression level was determined from the sequence data by using RSEM (25). 50,577 expressed transcripts with FPKM >0.3 were thus identified in the tail among these four metamorphic stages and the vast majority of transcripts (33,264 transcripts) were commonly expressed at all four stages (Figure 3).

**Figure 4:**
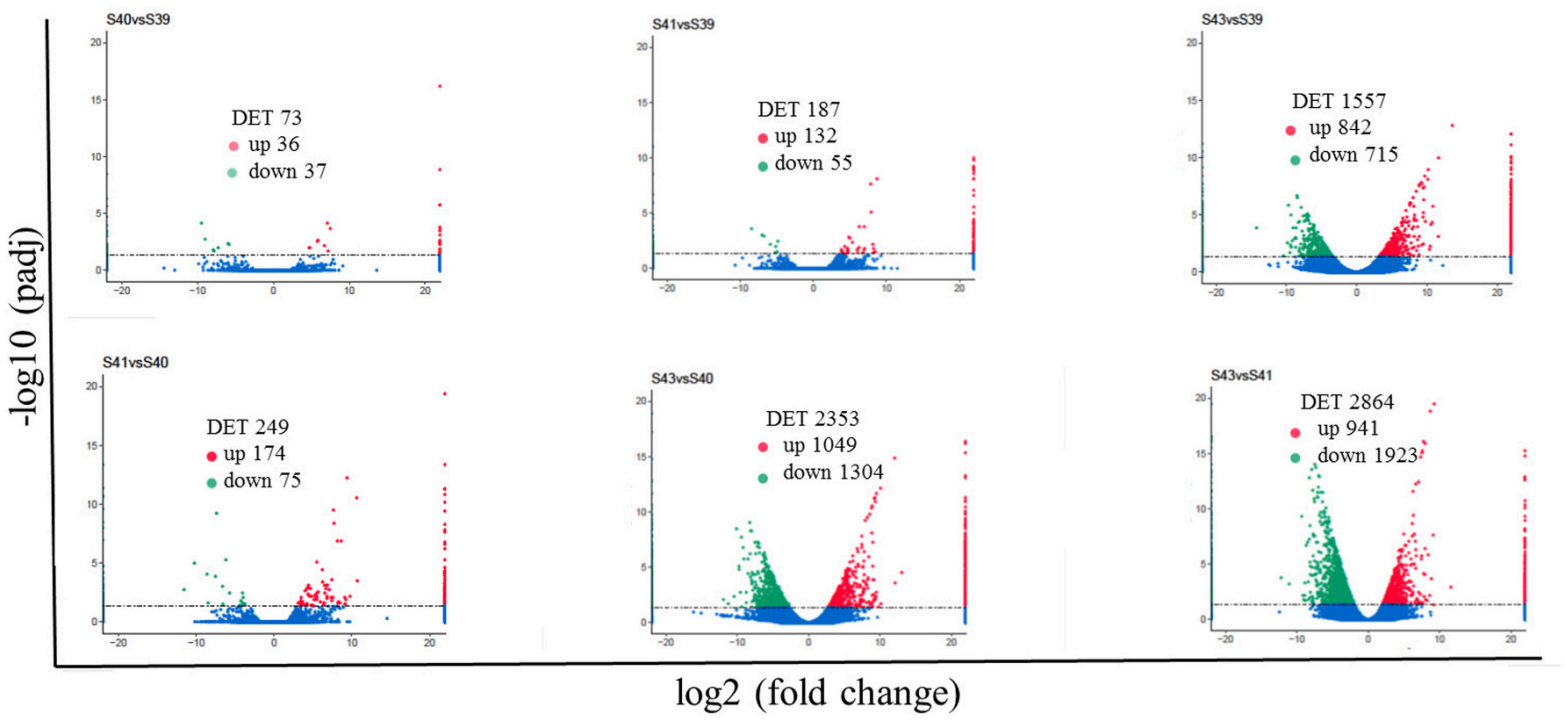
Pair-wise comparisons reveal that S43 tail has the most number of differentially expression transcripts (DETs, *q* < 0.05) during natural metamorphosis. Volcano plot of DETs in the tail for six different comparison groups. X axis represents fold-change (log2FC) of the DETs and the Y axis represents the –log10padj [adjusted *P*-value (*q*-value)] value of the DET, with 0.05 set as the significance cutoff level. Up-regulated DETs are shown in red while down-regulated ones in green; non-significantly DETs are presented in blue.

**Figure 5:**
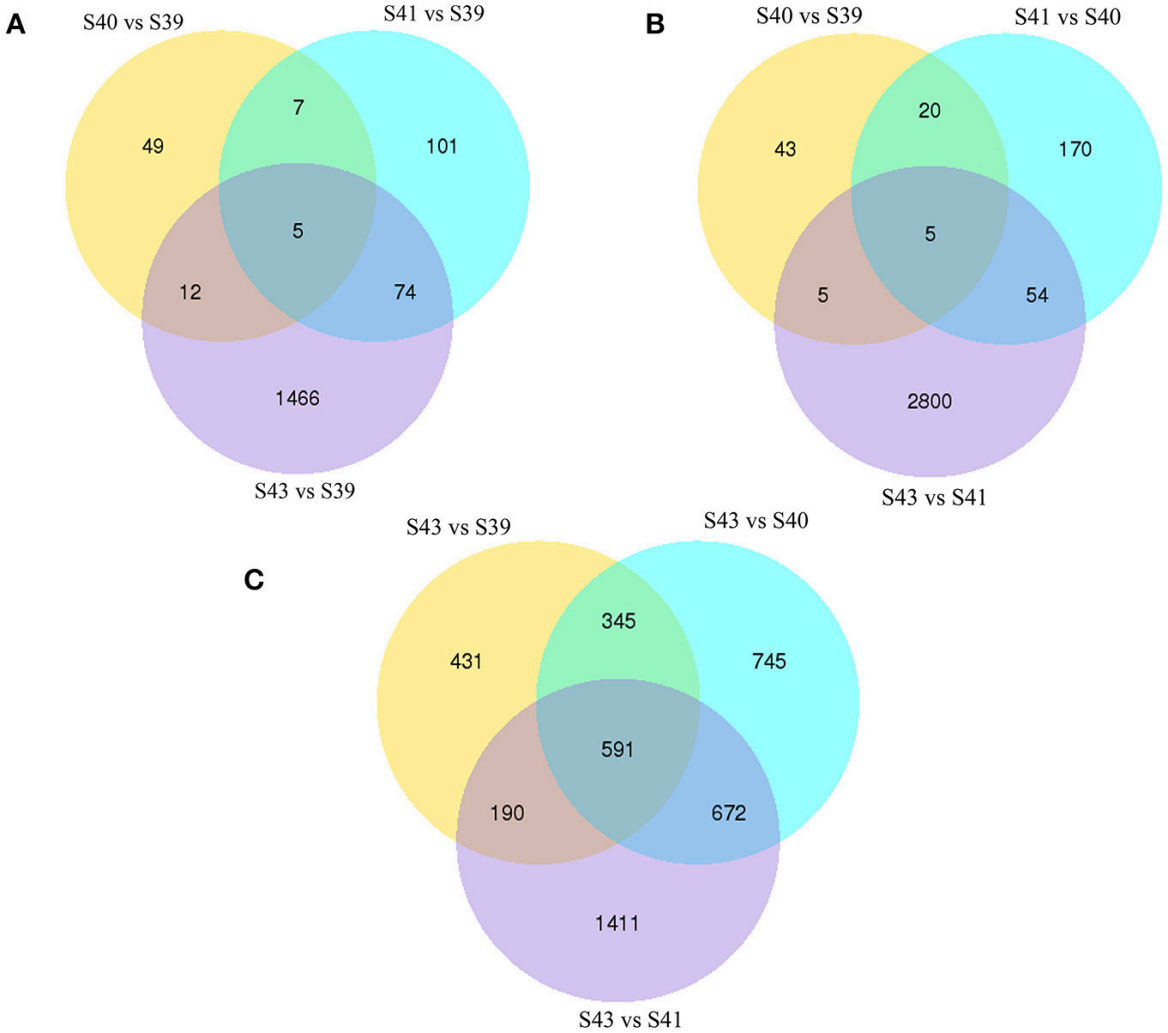
Venn diagrams of differentially regulated transcripts from pair-wise comparisons show transcript expression profiles at S39-S41 are similar but very different from those at S43. **(A)** Venn diagrams of DETs between S39 and the other three stages. Note that there were few DETs between S39 and S40 or S41 but much more between stages S39 and S43. **(B)** Venn diagrams of DETs between two successive stages. Note that there were few DETs between S39 and S40 or between S40 and S41 but much more between S41 and S43, indicating that the gene expression profile changes correlates well with the drastic morphological changes since drastic tail resorption takes place between S41 and S43 (Figure 1). **(C)** Venn diagrams of DETs between S43 and the other three stages. Note that there were many DETs between S43 and any other stages, including 591 common DETs between S43 and the other three stages.

Next, pair-wise comparisons of gene expression among the four stages were carried out, leading to the identification of 4,555 DETs in the tail during natural metamorphosis (Figure 4, Table S2). Among the 4 developmental stages, most DETs were found when comparing gene expression at S43 to any one of the other three stages, while relatively few DETs were found when the comparison was done among S39, S40, S41 (Figure 4). Consistently, Venn diagrams of the DETs between S39 and any one of the other three stages (Figure 5A) or the DETs between consecutive two stages (Figure 5B) showed very few commonly regulated genes. In contrast, Venn diagram of the DETs between S43 and any one of the other three stages showed that most of the DETs were common (Figure 5C). Finally, a heatmap of all 4,555 DETs showed that the vast of majority of the genes fell into two categories: highest and lowest expression at S43, respectively, but with similar expression levels among S39, S40, and S41 (Figure S1). These findings indicate that the tail at S43 has a very different gene expression program compared to those at the other three stages, while the tail gene expression programs at S39, S40, and S41 were very similar to each other, consistent with the lack of significant changes in the tail from S39 to S41 but drastic tail resorption by S43 (Figure 1).

### Identification of the Gene Regulation Programs Underlying Tail Resorption During Natural Metamorphosis

**Figure 6:**
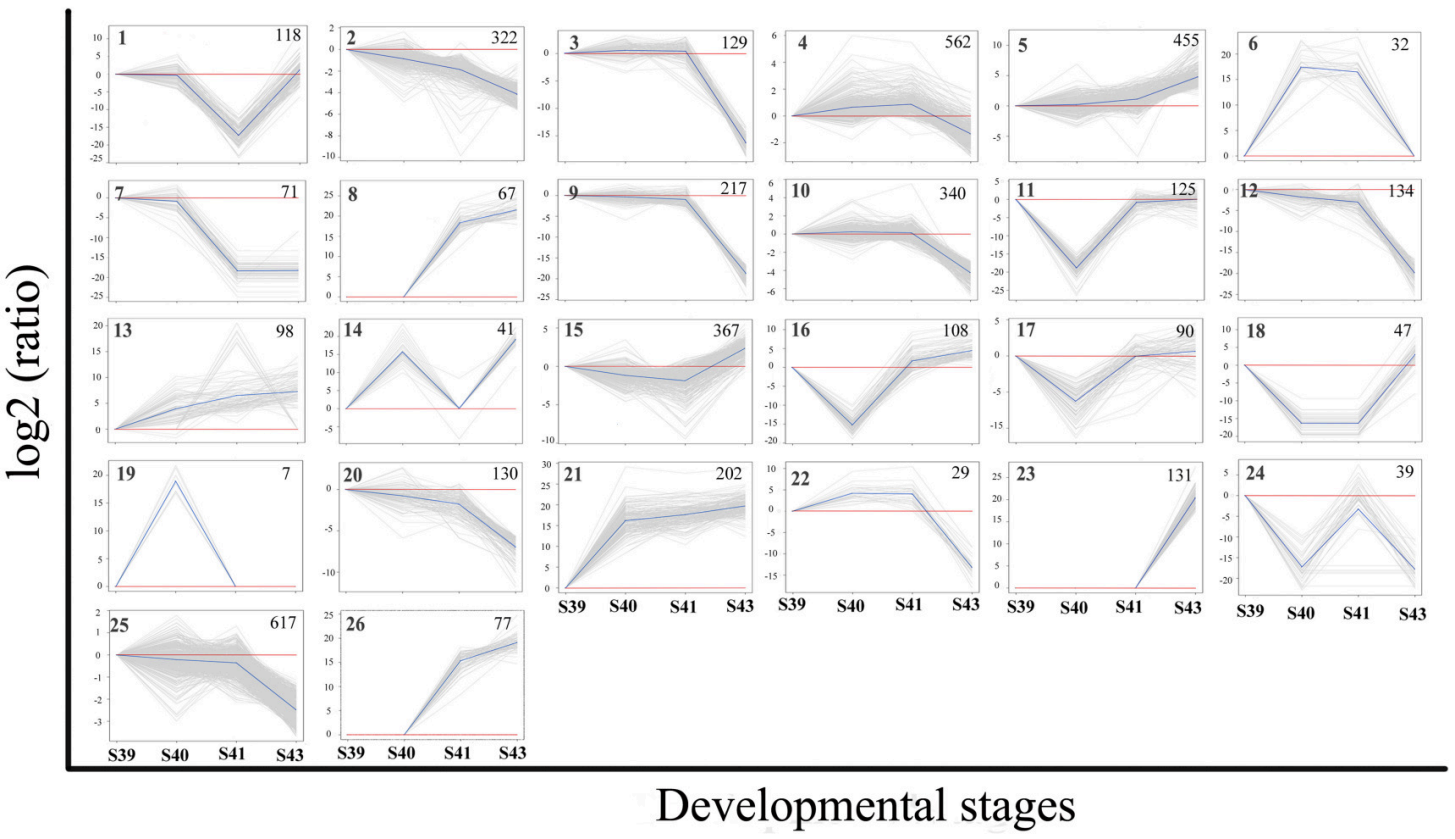
K-means clustering profile of DETs during tail resorption. A total of 4,555 DETs were grouped into twenty-six clusters based on their developmental regulation patterns. (Note that as there are three possible changes in gene expression between any two stages: up, down and unchanged, there are totally 27 possible clusters among 4 stages. One cluster, i.e., the one with no changes in gene expression among 4 stages, is absent among the DETs.) Each gray line represents one transcript and the average relative expression levels of all transcripts are shown as a blue line. The vertical axis represents the expression level and the horizontal axis shows the four developmental stages. The cluster number is shown as a bold number in the upper left corner and the number in the upper-right corner of each cluster indicates the number of transcripts in the cluster.

To identify the critical gene regulation programs governing tail resorption, we further analyzed the 4,555 DETs in the tail during natural metamorphosis. First, K-means clustering was performed to divide the DETs into the 26 possible expression patterns (clusters) (Figure 6). In agreement with the findings above, most of the DETs fell into two groups of clusters: one group of clusters (the up-clusters #5, 8, 13, 21, 23, 26) in which the DETs were gradually upregulated by S43 and another group of clusters (the down-clusters #3, 9, 10, 12, 20, 22, 25) in which the expression of the DETs changed little from S39 to S41 but were downregulated significantly between S41 and S43. Since metamorphosis begins around S39 and drastic tail resorption starts between S41 and S43, we focused on these two groups of clusters. Analyses of gene functional categories and biological pathways with GO and KEGG, respectively, on the 1,030 genes in the up-clusters revealed no pathways or GO terms was significantly enriched in the DETs based on the more stringent criterion of q < 0.05. On the other hand, a number of significantly enriched KEGG pathways and GO terms were found based on a value of *p* < 0.01 (Tables S3, S4, Figure S2). Most significant among them was the proteolysis GO term with 60 DETs (Figure S2), essentially all of which were upregulated by S43 but changed little from S39 to S41 (Figure S3, Table S5). The upregulation of this GO term is consistent with the rapid protein degradation associated with tail resorption after S41.

**Figure 7:**
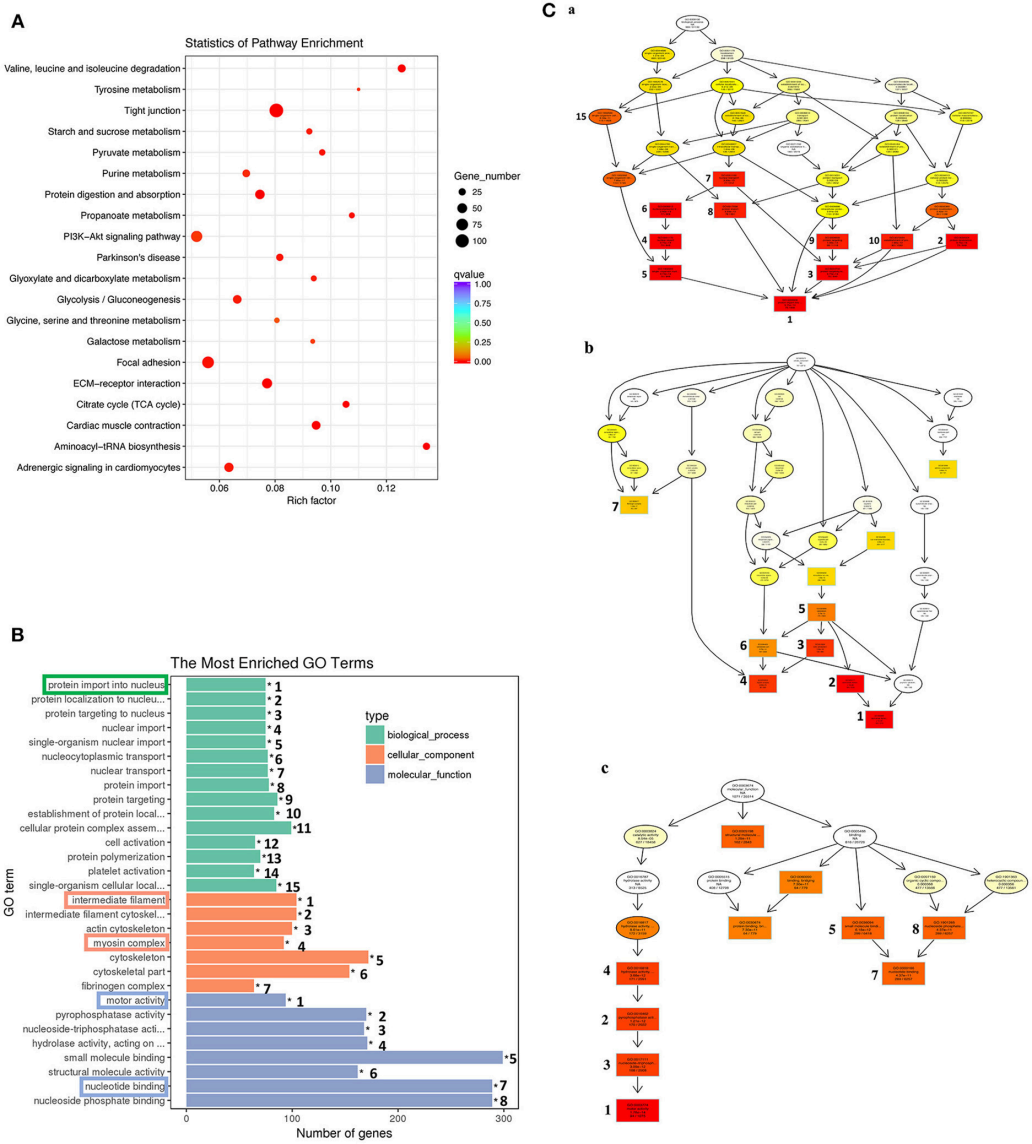
KEGG and GO analyses reveal important down-regulated pathways and GO terms during tail resorption. The 1,596 genes in the down-regulated subcluster-3, 9, 10, 12, 20, 22, 25, in Figure 6, were combined and subjected to GO and KEGG enrichment analyses. Twenty-one significantly enriched (*q* < 0.05) KEGG pathways (Table S6) and 499 significantly enriched (*q* < 0.05) GO terms (Table S7) were obtained. **(A)** Top 20 significantly enriched pathways based on smallest *q*-value (from the top to the bottom). Rich factor: the number of genes enriched relative to the known number of genes in the same pathway. Red color indicates the significant enriched pathways and the size of the circle corresponds to the known number of genes in the pathway. **(B)** Top 30 significantly enriched GO terms. Ranking numbers at right site indicate the significant level of the enriched GO terms; boxed ones on the left represent selected GO terms associated with tail resorption. **(C)** Directed acyclic graph (DAG) displaying the relationship among different GO terms. Each box and circle indicate a GO term and the descriptions inside of which show GO term id, GO description, *q*-value and the enriched DETs numbers comparison with background gene numbers of indicated GO term from top to bottom. Color depth represents the enrichment degree (red is the most enriched). The numbers next to the color boxes correspond to the GO ranking numbers in **B** within the three higher level GO terms as shown here: **(a)** Biological process (shown in green in **B**) with GO term “protein import into nucleus” as the most significantly down-regulated; **(b)**. Cellular component (shown in orange in **B**) with GO terms “myosin complex” and “intermediate filament” as the most significantly down-regulated; and **(c)**. Molecular function (shown in blue in **B**) with the GO term “motor activity” as the most significantly down-regulated.

Similar analyses on the 1,596 genes in the down-clusters revealed the enrichment of 21 KEGG pathways (Figure 7A, Table S6) and 499 GO terms (Figure 7B, Table S7) even based on the more stringent statistical criterion of *q* < 0.05. Many of the most significantly enriched KEGG pathways were related to metabolism, consistent with the tissue resorption process as the cells undergo apoptosis and reduce their own metabolism. The top 30 most significantly enriched GO terms were related to three higher level GO terms “Biological Process” (BP), “Cellular Component” (CC), and “Molecular Function” (MF) (Figure 7B), and were strongly associated with the protein import into nucleus, intermediate filament, myosin complex, motor activity and nucleotide binding (Figure 7B, boxed terms). When we carried out the analysis with directed acyclic graph (DAG), we found that the enriched GO terms within BP were all linked to a single node: the GO term “protein import into nucleus” (Figure 7Ca). On the other hand, the enriched GO terms within CC and MF could be linked to two GO nodes: the GO terms “myosin complex” and “intermediate filament” for CC (Figure 7Cb), and the GO terms “cytoskeletal components” and “muscle contraction” for MF (Figure 7Cc), respectively. The enrichment of these GOs among the down-regulated genes is again consistent with the tissue resorption program as the cells undergo apoptosis.

### Coordinated Regulation of Gene Categories Related to Tail Resorption During Natural Metamorphosis

**Figure 8:**
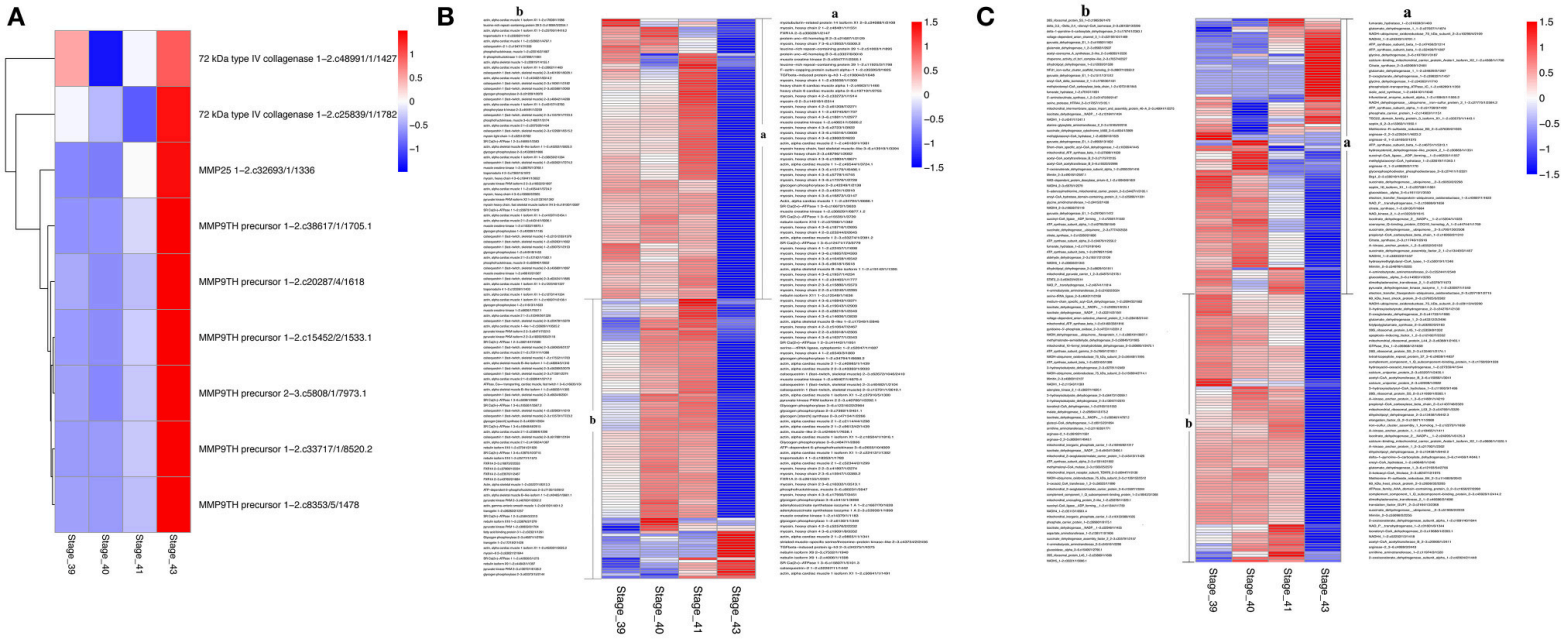
Heatmaps showing coordinated regulation of genes in selected GO terms during tail resorption. The intensity of color indicates relative expression levels. Red to blue corresponds to high to low levels of expression. **(A)** A heatmap of all transcripts encoding matrix metalloproteinases (MMPs) within the 4,555 DETs. Note that all MMP transcripts were highly upregulated at stage 43, although they were from only three different MMP genes. **(B)** A heatmap of all transcripts encoding genes related to muscle function within the 4,555 DETs. Note that nearly all were downregulated at stage 43 when tail resorption takes place. **(C)** A heatmap of all transcripts encoding genes related to mitochondrial function within the 4,555 DETs. Note that nearly all were downregulated at stage 43 when tail resorption takes place.

The discoveries of many KEGG pathways and GO terms associated with tail resorption above were based on genes that were coordinately regulated in up- or down-clusters. To determine if all DETs in a given KEGG pathway or GO term were coordinately regulated, we compared the gene regulation profiles of all DETs in selected pathways or GO terms that are likely important for tail resorption. It is well-known that matrix metalloproteinases (MMPs) are important for apoptosis and associated ECM degradation. A heatmap of all DETs encoding MMPs showed that all 9 DETs were coordinately upregulated dramatically at S43 (Figure 8A). Similarly, over 80% of the DETs belonging to the GO term “muscle function” (Figure 8B) or “mitochondrial function” (Figure 8C) coordinately down-regulated by S43, consistent with the apoptotic process taking place at S43.

**Figure 9:**
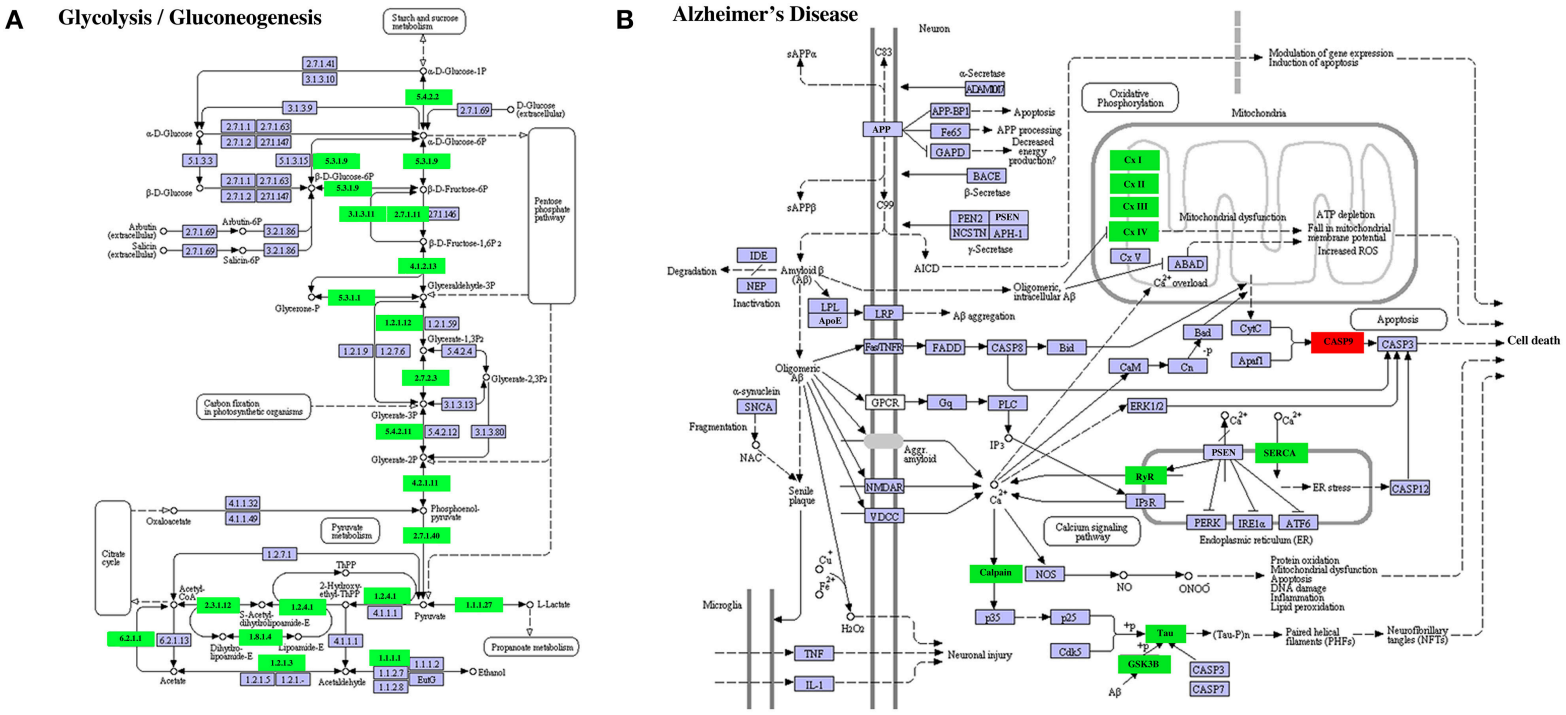
Selected enriched KEGG pathways among DETs between S41 and S43 during tail resorption. **(A)** Glycolysis/Gluconeogenesis pathway. Note that nearly all were downregulated between S41 and S43. A rectangle indicates a gene product (an enzyme). The down-regulated genes are shown in green. The light blue genes have no change in expression level. The numbers in the all boxes correspond to the enzyme commission numbers which are associated with a recommended name for the respective enzyme and clear circles indicate metabolic products. **(B)** Alzheimer's disease pathway. Note that all but one DETs were downregulated. The exception was CASP9, which is important for inducing cell death and not surprisingly upregulated. Green color indicates down-regulated DETs and red represents up-regulated DETs.

A similar result was obtained on DETs in the KEGG pathways “glycolysis/gluconeogenesis” (Figure 9A) or “Alzheimer's disease” (Figure 9B). In the former, all enriched DETs were down regulated between S41 and S43 while in the latter, 9 out of 10 DETs were downregulated. Thus, DETs in KEGG pathways or GO terms that are associated with apoptosis and tissue resorption are coordinately regulated during tail resorption.

## Discussion

Amphibian metamorphosis has long been used as a model to study postembryonic development in vertebrates, especially the developmental roles of T3. Earlier molecular and genetic studies on amphibian metamorphosis were mostly on *X. laevis* and more recently on *X. tropicalis*, especially with the advent of gene-editing technologies. While there are many advantages of using the *Xenopus* models, the aquatic nature of adult *X. laevis* and *X. tropicalis* makes them less than ideal as a model for mammalian postembryonic development. Toward developing the terrestrial diploid anuran *M. fissipes* as an alternative model for developmental and genetic studies, we have here assembled nearly 70,000 expressed transcripts and identified over 4,000 DETs during tail resorption. Our results highlight conserved gene regulation programs for tail resorption during *M. fissipes* and *X. laevis* and suggest that *M. fissipes* may be a valuable model for studying postembryonic development in mammals, especially for organs that are critical for the transition from aquatic to terrestrial living.

*Microhy fissipes* tadpoles are much smaller in body size and have a shorter developmental time through metamorphosis (Figure 1) (20, 21). *M. fissipes* is a diploid anuran and has large egg size (0.8–1.0 mm). These make it possible to adapt transgenic and gene-editing technologies for functional studies *in vivo*. On the other hand, *M. fissipes* genome is not yet sequenced. Using the newest sequencing platforms, we have here assembled nearly 70,000 transcripts and annotated nearly 80% of these transcripts by using various databases. These annotated transcripts should be a valuable resource for studying gene regulation and function during *M. fissipes* development.

We successfully used these annotated transcripts for analyzing RNA-seq data of *M. fissipes* tails at different stages of metamorphosis. In our study, we chose tadpoles at 4 different stages: S39, S40, S41, and S43. S39 is essentially a premetamorphic stage when few T3-depdent metamorphic changes occur. S40 and S41 are early metamorphic climatic stages when extensive metamorphic changes occur, particularly limb development and body/head structure transformations. However, tail remains essentially unchanged up to S41. Finally, S43 is in middle of most dramatic tail resorption with tail length halved (Figure 1). Thus, an RNA-seq analysis of these 4 stages should provide a detailed description of the gene expression profiles underlying tail resorption.

In agreement with the morphological changes of tail during these stages, our RNA-seq analyses revealed relatively few DETs among S39, S40, and S41. Most dramatic changes in gene expression profile take place between S41 and S43 when rapid tail resorption occurs, accompanied by increased levels of apoptosis or programmed cell death in the tail. Surprisingly, however, our GO ontology analysis of the upregulated DETs did not identify cell death as an enriched GO term among the DETs. In fact, of all DETs related to cell death, about ½ were upregulated and ½ were down regulated (data not shown) at S43. Thus, while cell death is an essential event during tail resorption, the expression of cell death-related genes was not coordinately regulated. This may reflect the fact that apoptosis often involves post-transcriptional activation of proteins involved in cell death. On the other hand, all DETs encoding MMPs were coordinately upregulated during tail resorption. MMPs are important cell surface or extracellular proteases and have been shown to be important players for development cell death, including amphibian metamorphosis (32, 33). These findings are in agreement with microarray studies of gene regulation during tail resorption in *X. laevis* (31).

The downregulated DETs had many highly enriched KEGG pathways and GO terms. In agreement with a degenerative process for tail resorption, the DETs in the glycolysis/gluconeogenesis pathway were coordinately downregulated by S43 as the tail length shortens rapidly. Interestingly, we also found many DETs in the Alzheimer's disease pathway and Parkinson's disease pathway were coordinately downregulated during tail resorption. This suggests that similarities exist between tail resorption and neuronal degeneration in the development of Alzheimer's disease (Figure 9B) and Parkinson's disease (data not shown), and that studying gene function during tail resorption may provide insights toward understanding these diseases. The GO terms “muscle function” and “mitochondrial function” were two highly enriched terms with some of largest numbers of downregulated DETs. Heatmap analysis of all DETs encoding genes in these two GO terms showed that the vast majorities (>80%) of the DETs in these two GO terms were coordinately downregulated at S43. Many of the DETs were common in these two GO terms. These findings are consistent with the fact that muscle is the predominant tissue in the tail and muscle degeneration is thus one of the critical, major events during tail resorption. As muscle cells undergo apoptosis, genes important for muscle function, including those for mitochondria activities, are expectedly downregulated. Similar findings were also obtained in the microarray studies of tail resorption during *X. laevis* metamorphosis (31), again supporting conserved gene regulation programs governing tail resorption in *X. laevis* and *M. fissipes*.

In summary, we report here the assembly of nearly 70,000 transcripts and annotation of over 50,000 of these transcripts. By using these transcripts and RNA-seq analysis, we have obtained a detailed gene regulation profiles underlying tail resorption during natural metamorphosis in *M. fissipes*. Our findings suggest the existence of conserved gene regulation programs for tail resorption in *X. laevis* and *M. fissipes*. We have also made a novel discovery that the many genes in the Alzheimer's disease pathway and Parkinson's disease pathway were coordinately downregulated during tail resorption, implicating studies on tail resorption in *M. fissipes* may help our understanding on neurodegenerative diseases. Finally, *M. fissipes* undergoes a transition from aquatic to terrestrial living, closely resembling the postembryonic development in mammals. Thus, it is a good alternative model to either *X. laevis* or *X. tropicalis* for studying postembryonic development of organs that are critical for terrestrial living.

## Ethics Statement

The care and treatment of animals used in this study were in accordance with the guidelines of the Animal Care Committee, Chengdu Institute of Biology (2015-AR-JJP-01).

## Author Contributions

SW, LL, and JJ conceived and designed the experiment. SW and Y-BS analyzed the data and prepared the manuscript. JL, WZ, YT, LF, and LB assisted with the bioinformatics analysis. All authors participated in the manuscript preparation and approve the final version of the manuscript.

### Conflict of Interest Statement

The authors declare that the research was conducted in the absence of any commercial or financial relationships that could be construed as a potential conflict of interest.
