# Supplementary material for: Gene Expression Program Underlying Tail Resorption During Thyroid Hormone-Dependent Metamorphosis of the Ornamented Pygmy Frog Microhyla fissipes
Source: Front Endocrinol (Lausanne). 2019 Jan 25;10:11. doi: 10.3389/fendo.2019.00011 (PMC6357680; doi:10.3389/fendo.2019.00011)
Supplement: Supplementary file 1 [file Table_1.pdf]

Table S1. Summary of sequencing and annotation statistics*.

SMRT Sequencing				Database	Number of Transcripts	Annotation Rate (%)
Index	Total Nucleotides	108,614,218	Annotation	NR	47,112	69
	All Transcripts	67,939		NT	34,376	51
	Length (200-500 bp)	149		KEGG	31,219	46
	Length (500-1 kbp)	16,938		SwissProt	42,850	63
	Length (1k-2k bp)	29,938		PFAM	39,822	59
	Length (>2k bp)	20,914		GO	41,254	61
	Mean length	1,599		KOG	23,217	34
	Max length	8,847		Annotated in all databases	14,755	22
	N50	1,956		Annotated in at least one	52,881	78

	Tail Sample Stage	Raw Reads	Clean Reads	Clean Bases (G)	Reads mapped to SMRT Sequences (Mapping Rate, %)	Total Expressed Transcripts	Total Differentially Expressed Transcripts
Illumina RNA-Seq	S39_1	55,552,298	53,791,200	8.07G	33,920,962 (63.06)	50,577	4,555
	S39_2	52,655,948	50,780,062	7.62G	24,868,472 (48.97)		
	S39_3	61,860,648	60,078,372	9.01G	41,010,354 (68.26)		
	S40_1	54,579,738	52,955,948	7.94G	34,743,530 (65.61)		
	S40_2	55,347,336	53,758,202	8.06G	36,672,612 (68.22)		
	S40_3	49,016,642	47,597,972	7.14G	30,202,684 (63.45)		
	S41_1	47,821,448	46,390,360	6.96G	36,530,468 (67.48)		
	S41_2	55,367,586	53,703,782	8.06G	36,530,468 (68.02)		
	S41_3	47,986,070	46,516,764	6.98G	30,388,544 (65.33)		
	S43_1	52,716,544	50,486,292	7.57G	21,951,654 (43.48)		
	S43_2	52,974,420	51,209,534	7.68G	19,024,704 (37.15)		
	S43_3	57,584,078	55,416,696	8.31G	24,868,472 (54.62)		

*Total RNA was isolated from heart, liver, spleen, lung, kidney, skin, ovary, and testis from an adult *M. fissipes* as well as from tails at four stages (S38, S40, S41, S43) and dorsal muscle at three stages (S36, S43, S45). The RNA was sequenced on a PacBio RSII platform. All high-quality short reads from the RNA-Seq from the tails at eight developmental stages (S18, S19, S21, S28, S39, S40, S41, and S43) were used to error-correct the SMRT sequences by using the Proovread correction software. Totally 108,614,218 nucleotides and 67,939 transcripts with an average length of 1,599 bp and N50 of 1,956 bp were obtained. The N50 value is defined as the contig length where half of the assembled transcripts were represented by contigs of this size or longer. In addition, 47,112; 34,376; 31,219; 42,850; 39,822; 41,254 and 23,217 transcripts were annotated through NR, NT, KEGG, Swiss-Prot, PFAM, GO and KOG databases, respectively. In the end, 52,881 transcripts (78% of the 67,939 transcripts) were annotates in at least one database (Table S1). In order to address the gene expression program underlying tail resorption, we used Illumina RNA-Seq clean reads of four key tail resorption stages (S39, S40, S41, S43) mapping to proofread-corrected reference sequences. Finally, the gene expression level was determined by using RSEM software {Li, 2011 #4213}. 50,577 expressed transcripts with FPKM (fragments per kilobase of exon model per million mapped reads)>0.3 were identified in the tail among the four metamorphic stages (S39, S40, S41, S43) and 4,555 DETs during natural metamorphosis were discovered through Pair-wise comparisons.
