# Supplementary material for: Gene Expression Program Underlying Tail Resorption During Thyroid Hormone-Dependent Metamorphosis of the Ornamented Pygmy Frog Microhyla fissipes
Source: Front Endocrinol (Lausanne). 2019 Jan 25;10:11. doi: 10.3389/fendo.2019.00011 (PMC6357680; doi:10.3389/fendo.2019.00011)
Supplement: Supplementary file 2 [file Table_2.pdf]

Table S2. All tail DETs among the four stages during natural metamorphosis.
4,555 DETs were obtained by pair-wise comparison of gene expression among the four stages and listed with detail sequence data, containing FPKM of 12 samples (four stages and three repeats).

Gene ID	NR Description	NR GI	NR ID	Fpkm_S39_1	Fpkm_S39_2	Fpkm_S39_3	Fpkm_S40_1	Fpkm_S40_2	Fpkm_S40_3	Fpkm_S41_1	Fpkm_S41_2	Fpkm_S41_3	Fpkm_S43_1	Fpkm_S43_2	Fpkm_S43_3
3-6.c1441/5/4345	PREDICTED: myosin-4 [Xe	847156920	XP_0128244	0	1.04	0	0	0	0	53.63	49.46	85.87	0	0	0
2-3.c2233/16/1957	PREDICTED: pre-mRNA 3'-	847088065	XP_0128194	0	72.71	0	0	0	0	18.65	39.37	32.99	0.26	20.03	0
3-6.c13156/2/2931	PREDICTED: obscurin isofo	847141105	XP_01282121	37.5	0	17.5	26.73	20.25	26.46	0	0	0	0	0	9.92
2-3.c41191/3/2279	PREDICTED: nebulin isofor	847166510	XP_01282661	14.42	7.55	42.69	0.22	0	0	51.48	26.53	72.64	7.06	43.11	41.39
3-6.c13721/1/3884	PREDICTED: RNA-binding	847153751	XP_0128236	12.01	0	7.59	10.48	7.5	14.19	0	0	0	0	12.79	13.24
2-3.c1136/122/2087	DEAD (Asp-Glu-Ala-Asp) b	171847235	AAI61473.1	0	27.76	7.27	0	0	0	15.45	13	23	0	6.24	0
2-3.c47767/1/2410.	repressor of RNA polymeras	148228865	NP_0010807	0	33.47	20.46	0	0	0	28.99	29.25	32	69.28	51.19	22.13
3-6.c13897/1/4006	PREDICTED: dynactin subu	512817195	XP_0049113	0	17.81	4.46	0	0	0	8.07	5.29	10.76	13.79	0	15.1
2-3.c53364/1/2475	--	--	--	0	0	0	0	0	0	10.93	12.4	13.23	0	1.98	5.14
2-3.c62424/4/2099	--	--	--	26.4	0	14.52	23.9	14.39	12.44	0	0	0	0	0	56.76
2-3.c27791/1/2087	ryanodine receptor beta isofo	29501272	BAA04647.2	5.48	0.3	26.99	22.12	14	12.8	0	0	0	0	4.44	6.31
1-2.c35161/1/1523	PREDICTED: paraplegin [X	301608280	XP_0029337	35.57	7.16	6.49	0	0	0.08	12.83	24.59	30.31	19.41	28.24	0
1-2.c3283/3/1520	coatomer subunit alpha [Xen	189230284	NP_0011214	0	0	0	0	0	0	19.84	15.62	19.46	0	0	75.59
2-3.c35035/1/2188	--	--	--	9.41	0.72	12.39	13.14	9.72	14.01	0	0	0	10.07	6.61	5.59
2-3.c1892/15/2499	PREDICTED: myosin-4-like	847156931	XP_0128244	0	0	58.82	0	0	0.68	47.07	35.29	31.9	0	0	0
1-2.c28106/2/1421	--	--	--	13.53	35.51	0.49	0	0	0	18.31	10.01	24.78	0	10.41	9.49
3-6.c7577/2/3351	PREDICTED: obscurin isofo	847141062	XP_0128211	23.33	0.27	33.09	37.91	30.53	40.16	0.42	0.18	0	0	0	13.23
2-3.c61888/2/2470	gsn protein [Xenopus (Silura	159155320	AAI54876.1	0	0.01	0	8.95	7.89	7.71	0	0	0	20.77	0	12.35
2-3.c7227/1/2395	PREDICTED: dynactin subu	847096031	XP_01281121	0	37.3	6.29	0.08	0	0.2	21.91	8.78	20.83	45.33	0	45.17
3-6.c18138/1/2699	--	--	--	2.69	6.58	0	0	0	0	3.74	5.46	9.09	0	0	1.47
2-3.c41733/1/1985	2-oxoglutarate dehydrogenas	148231573	NP_00108361	0	0	4.92	10.31	7.38	10.26	0	0	0	0	0	0
1-2.c7280/1/1456	alanine aminotransferase 2 [X	147898847	NP_0010861	0	6.04	0	0	0	0	13.1	6.21	15.21	39.73	51.45	6.56
1-2.c33588/1/1278.	NmrA-like family domain-co	226371956	ACO51603.1	0.43	2.92	3.16	0	0	0	19.24	18.38	11.01	0	2.11	2.65
1-2.c43620/1/1741	hedgehog acyltransferase-lik	147901798	NP_0010794	21.02	0	0	23.27	23.69	7.12	0	0	0	0	0.63	3.94
2-3.c7627/1/2303	LOC100170459 protein [Xen	189441985	AAI67321.1	0	0	0	0	0	0.06	7.34	6.04	8.2	0	0	26.14
1-2.c22445/2/1199	v-ral simian leukemia viral o	156717550	NP_00109631	0	39.05	0	0	0	0.22	15.64	19.02	16.56	0	0	0
1-2.c36862/1/1190	PREDICTED: LOW QUALI	823461821	XP_0041745	35.19	0	0	0	0	0	95.21	48.58	22.49	35.75	49.21	5.63
1-2.c21098/1/1790	--	--	--	16.32	3.56	3.26	0	0	0	4.92	7.43	7.3	7.3	8.73	0.55
1-2.c16956/2/1310	PREDICTED: propionyl-Co	831302652	XP_0126833	14.61	27.66	0	0	0	0	13.39	8.57	4.68	11.21	16.07	13.2
3-6.c12176/96/3037	PREDICTED: endoplasmin,	514758710	XP_00502261	0	5.14	0	5.91	7.42	16.05	0	0	0.38	9.54	7.13	0.01
2-3.c19240/1/2186	--	--	--	3.26	26.87	0.04	0	0	0	2.99	3.61	5.23	7.62	5.28	5.57
1-2.c41203/1/1702	--	--	--	0.91	0.19	8.52	2.32	0	0	32.45	39.65	29.04	8.5	65.65	50.42
2-3.c51959/2/2303	alanine aminotransferase 2 [X	147898847	NP_0010861	20.5	0	7.32	17.95	15.11	4.19	0	0.03	0	0	0.02	3.25
2-3.c11669/5/2172	PREDICTED: TATA-bindin	733916099	XP_0107201	3.56	19.25	4.35	2.32	3.31	7.97	0	0	0	0	0	0
2-3.c41096/1/2003.	--	--	--	24.08	13.64	1.34	15.36	13.27	8.09	0	0	0	22.03	0	1.53
2-3.c4365/2/2244	--	--	--	0.74	13.61	0	0	0	0	4.47	2.68	2.71	0	0	0
2-3.c52049/2/2237	--	--	--	0.41	12.19	0.76	0.28	0	0	6.85	7.02	7.23	15.25	4.27	0.14
1-2.c20910/1/8680.	PREDICTED: tubulin alpha-	820994203	XP_01236371	0	0	0	0	0	0	15.37	6.87	5.82	57.17	63.04	24.55
2-3.c36785/2/1936	--	--	--	0.86	7.92	5.07	0	0	0	5.67	1.63	3.78	1.09	0	0.21
2-3.c57641/1/1851	PREDICTED: moesin isofor	847156257	XP_0128243	0	8.3	2.77	4.72	1.72	8.39	0	0	0	0	0	0
3-6.c9057/1/2915	protein NDRG1-B [Xenopus	147906416	NP_0010806	0.49	1.5	0	0	0	0	3.79	1.55	1.46	0	1.08	0
1-2.c2630/24/1697	PREDICTED: microtubule-a	847167931	XP_0128270	0.88	13.93	5.61	9.71	6.17	2.64	0	0	0	0	0	1.91
3-6.c3590/4/2974	PREDICTED: AP-1 complex	620946340	XP_0076704	9.17	1.55	1.02	0	0	0	1.56	2.53	2.03	0.79	1.8	0
2-3.c4225/1/2520	--	--	--	18.72	0.12	0.09	15.03	14.65	3.47	0	0.03	0.05	0	5.96	12.09
2-3.c24293/1/2276.	--	--	--	5.63	9.79	0.13	0	0	0	10.61	7.4	3.91	178.68	10.57	102.24
2-3.c2940/4/2236	--	--	--	4.3	3.87	0.92	0	0	0	3.61	3.95	1.01	0.73	2.42	3.69
1-2.c32719/1/1162	Syntaxin-12 [Rana catesbeia	226372600	ACO51925.1	0	0	0	0	0	0	14.17	8.88	2.93	44.03	57.64	3.65
1-2.c28087/1/1379	increased sodium tolerance 1	147903060	NP_0010866	26.84	4.61	0	0	0	0	2.51	11.6	9.12	0	0.24	0
2-3.c26330/1/2172.	--	--	--	14.72	62.57	0.82	15.1	16.98	32.21	0	0.19	0.23	42.72	31.7	90.68
1-2.c40050/2/1435	--	--	--	0	0	2.4	5.74	4.63	5.25	0	0	0	7.19	3.65	9.46
2-3.c40177/2/2081	PREDICTED: 5'-3' exoribon	301620220	XP_0029394	1.54	0.69	2.19	0	0	0	3.08	2.39	2.83	1.11	5.33	2.69
2-3.c49412/1/1982	PREDICTED: uncharacterize	332219891	XP_0032590	0	6.25	1.44	0	0	0.08	3.08	3.43	4.92	3.6	1.7	0
2-3.c59243/1/1988	probable ATP-dependent RN	156717218	NP_0010961	0	10.51	0	1.45	4.02	4.44	0	0	0	0	0	0
1-2.c42745/6/1737	myosin, heavy chain 4, skele	47575800	NP_0010012	28.28	2.63	0	0	0	0	0	211.06	392.8	0	0	0

2-3.c19652/1/2088	--	--	--	0	13.09	9.56	0.05	0	0	9.32	9.11	32.65	0	6.88	15.21
1-2.c9178/2/1376	--	--	--	0	34.99	0	3.49	0	0	149.12	47.38	65.57	199.12	399.87	3.69
2-3.c20118/2/2131	Unknown (protein for IMAG	197245540	AAI69188.1	0.53	0.23	0	0	0	0	5.26	2.01	1.58	0	0	0
2-3.c56283/1/2248	PREDICTED: ubiquitin carb	847116837	XP_01281604	2.9	4.63	3.25	3.08	2.96	1.85	0	0	0	0	0	4.74
2-3.c20505/2/1972	PREDICTED: myosin-4 [Xe	847156920	XP_0128244	171.75	0	0	0	0	0	0	437.51	275.86	0	0	0
3-6.c10108/1/3081	hypoxia-inducible factor 1 al	166343757	ABY86629.1	0.7	0	0	0	0	0	1.82	1.64	1.28	2.74	0	2.32
1-2.c8929/3/1527	--	--	--	0	0	1.27	0	0	0	10.01	2.82	3.69	5.42	0	8.97
1-2.c51783/5/1362	collagen, type 1, alpha 2 prec	147898763	NP_00108072	0	1765.76	0	0	0	0	48.95	2.04	58.28	33.32	0	0
2-3.c20273/3/2456	Unknown (protein for IMAG	197245540	AAI69188.1	0	3.59	0	0	0.06	0	3.22	2.11	3.38	0	0	0
1-2.c13672/1/1172	--	--	--	4.53	58.34	26.79	2.43	1.25	2.54	28.85	37.42	57.11	8	67.98	55.34
1-2.c51597/201/175	tyrosine 3-monooxygenase/tr	89515082	ABD75373.1	1.08	177.11	82.16	0	129.73	113.06	0	0	0	83.57	0	91.29
2-3.c1621/73/1987	nucleosome assembly protein	537227872	ERE83488.1	3.05	7.88	0	1.38	6.48	4.31	0	0	0	0	0	0
1-2.c39930/1/1674	--	--	--	1.37	0	0	4.71	1.04	4.84	0	0	0	1.7	0.28	0
1-2.c6741/1/1788	WD repeat-containing protei	147901327	NP_00109144	0	4.42	0.25	0.1	0	0	3.12	5.28	3.54	3.01	5.76	0
1-2.c37842/2/1583.	PREDICTED: epsilon-sarcog	641796609	XP_0081616	0	0	0	0.19	0	0	6.38	7.78	3.01	18.12	15.93	2.47
2-3.c31999/4/1960	PREDICTED: angio-associat	602656532	XP_0074339	0	8.61	0	0	0	0	1.87	2.34	3.13	0	0	0
1-2.c20983/1/1384.	--	--	--	0	0.77	24.62	19.21	20.2	10.18	0.17	0	0.33	160.2	1.29	0.37
1-2.c41057/2/1395	PREDICTED: ras suppressor	507973237	XP_0046913	0.14	29.24	0.23	0	0	0	13.82	5.38	3.14	32.01	18.72	2.99
2-3.c2151/20/2271	methionine-R-sulfoxide redu	261399890	NP_00100497	1.85	0	0.86	1.09	2.23	3.86	0	0	0	0.41	0	0
1-2.c22970/1/1459.	PREDICTED: triadin isoforn	637253484	XP_0081182	0	0	0	0	0	0	179.37	134.68	0	123.39	0	0
2-3.c30752/1/2047	PREDICTED: splicing factor	823464129	XP_0124304	0	0	1.3	0	0	0	3.26	1.74	1.84	2.87	0.31	0.15
1-2.c48124/2/1326	LOC733385 protein [Xenopu	76779703	AAI06651.1	0	1.14	1.98	0	4.19	0	25.33	32.13	28.97	1.16	36.64	2.34
1-2.c23008/2/1331	--	--	--	2.8	0	0	0	0	0	5.32	1.76	4.86	22.3	18.51	16.96
2-3.c42844/1/2482	serine/threonine kinase 38 [X	147899268	NP_00108041	1.04	2.05	0	0	0	0	3.1	1.29	1.06	0	0	0
2-3.c35643/1/2578.	polycomb protein SCMH1 [X	54262200	NP_00100579	3.34	0.42	0	0	0	0	4.57	0.66	4.44	1.13	6.18	7.97
2-3.c40609/1/1885	PREDICTED: nebulin isofor	847166508	XP_0128266	0	2.26	1.75	0.1	0	0	5.08	2.98	2.62	1.27	0	0
1-2.c29316/1/1343	increased sodium tolerance 1	147903060	NP_00108665	27.56	0.55	7.32	0.49	0.84	0.49	13.23	17.2	13.63	20.85	36.91	1.44
2-3.c12246/5/1957	PREDICTED: nebulin isofor	847166510	XP_0128266	0	0	0	0	54.47	53.52	0	0	0	0	0	0
2-3.c54827/1/2299	PREDICTED: uncharacterize	332219891	XP_0032590	2.12	23.99	7.39	0.86	1.42	0.99	12.79	20.17	22.43	21	13.99	1.44
1-2.c23709/1/1265	heat shock protein 110 [Xeno	91805313	ABE65386.1	6.32	22.23	5.53	1.74	4.31	7.43	0	0	0	0	0	14.46
1-2.c45532/1/1394	PREDICTED: basic proline-r	612060000	XP_0075061	5.19	0	0	6.25	3.51	2.33	0	0	0	0	11.08	11.92
3-6.c15844/1/3295	PREDICTED: nebulin isofor	847166495	XP_0128266	0	0	0	0.5	79.45	125.19	0	0.05	0	0	0	0
3-6.c16115/1/3550	glucosidase, alpha; neutral A	148232724	NP_00109123	1.6	3	0.62	0	0	0	1.64	1.38	0.35	0	0.83	5.16
2-3.c58589/4/2000	vigilin [Xenopus (Silurana) t	55742112	NP_00100685	0	0	0	0	0	0	57.62	79.51	0	0	0	23.49
1-2.c9374/2/1526.1	PREDICTED: lipid phosphat	847087033	XP_0128161	0.36	0	0	0.11	0	0	9.88	1.98	6.25	33.92	39.02	14.54
1-2.c37563/2/1783	PREDICTED: keratin, type I	326919555	XP_0032060	0.02	0	43.17	1.01	5.19	0	36.42	35.52	27.01	0.01	2.79	0
1-2.c49476/1/1248	increased sodium tolerance 1	147903060	NP_00108665	13.32	11.14	0	0	0	0	2.64	2.58	6.2	30.62	60.76	0
2-3.c33454/2/2381.	tax1-binding protein 1-like B	820160924	KKX21603.1	0	2.46	0	0	0	0	1.42	2.61	2.88	0	0.45	0
2-3.c39404/1/2181.	aquaporin 3-like [Hyla chrys	86553029	ABC98210.1	0	5.67	8.26	0	0.58	0	17.37	8.11	5.2	44.1	0	120.52
2-3.c12316/3/2702	claudin-1 [Xenopus (Silurana	62751357	NP_0010157	31.36	28.74	23.58	0	0	0	27.85	0	21.58	0	0	71.28
2-3.c13805/4/2578	transcription factor Dp-1 [Xe	148222844	NP_00108805	1.45	8.42	1.55	1.72	2.48	0.9	0	0	0	1.89	0	3.3
2-3.c60180/1/2529	PREDICTED: pseudouridyla	847109932	XP_0128145	0	0.62	0.27	1.27	1.66	2.54	0	0	0	0	0.31	0.73
1-2.c45554/1/1861.	uncharacterized protein LOC	73853872	NP_00102751	0	0	0	0	0	0.16	5.75	2.54	5.41	8.96	7.83	16.98
1-2.c29226/1/9331.	tyrosine 3-monooxygenase/tr	89515082	ABD75373.1	11.8	0	4.37	8.84	5.22	3.94	0	0	0	0	36.83	16.88
1-2.c11691/1/1275	PREDICTED: LOW QUALI	655894700	XP_0027232	3.61	0	20.71	25.36	16.27	7.19	0	0	0.95	0.82	1.14	4.89
1-2.c16967/2/1621	PREDICTED: pre-mRNA 3'-	847088056	XP_0128194	51.3	0	26.99	25.83	22.25	16.14	3.37	0.41	0	19.99	17.96	24.12
1-2.c8009/1/1826	PREDICTED: ATP-depende	641754248	XP_0081770	0	29.5	10.32	0	0	0	3.65	6.1	1.18	0	0	3.85
2-3.c4869/1/2060	scinderin [Xenopus laevis]>g	148236707	NP_00108608	0	18.25	0	0.3	0	0.16	2.19	8.34	7.69	4.29	1.37	0
3-6.c4669/1/4636.2	PREDICTED: myosin-4 [Xe	847156920	XP_0128244	108.43	3.27	0	0	0	0.04	0	290.43	196.98	0	0	0
1-2.c48375/1/1497	exocyst complex component	54020886	NP_0010056	1.23	13.44	0.72	1.01	0.37	0.53	11.97	11.81	12.92	25.6	3.82	0.73
2-3.c4959/1/1894	PREDICTED: RNA polymer	847153928	XP_0128237	0	4.31	0	0	0	0	3.34	1.43	1.52	6.23	0.81	0
2-3.c43967/1/2629	probable UDP-sugar transpor	156717564	NP_00109632	1.66	0	1.63	1.51	2.5	0.69	0	0	0	0	0.52	2.51
1-2.c25949/1/1545	nuclear factor interleukin-3-r	62751891	NP_00101571	3.41	0.77	0	0	0	0	10.8	5.69	1.26	2.42	2.12	1.13
2-3.c39269/3/2173	--	--	--	0	16.22	0	3.15	5.67	18.83	0	0	0	0	0	0
2-3.c39742/1/2119	PREDICTED: myosin-4 [Xe	847156920	XP_0128244	0	0	0	0	0	0	0	24.21	21.48	0	0	0
3-6.c15231/1/3518	ubiquitin carboxyl-terminal h	187608155	NP_00112016	4.93	6.1	0	0	0	0	3	2.57	0.24	1.18	2.71	1.8
1-2.c16622/1/5384	DDB1- and CUL4-associated	45360843	NP_989097.1	0	0	0	0.56	0	0	11.35	3.13	6.84	30.79	50.37	21.13
1-2.c15273/1/1831.	PREDICTED: glycophorin-C	719767613	XP_0102181	0	8.71	0	0	0	0	3.32	2.29	3.95	0	0	0

2-3.c6790/1/2408	sequestosome-1 [Xenopus (S	56118722	NP_00100789	23.4	2.99	0	0.77	0	2.45	31.98	19.31	9.51	31.72	11.51	1.89
2-3.c13532/1/2165	--	--	--	2.44	6.15	1.74	0	0	0	0.82	1.43	2.86	0	1.62	0
1-2.c15002/7/1695	uncharacterized protein LOC	73853872	NP_00102751	0	0	0.1	0	0	0	2.07	7.07	2.79	6.02	0	9.97
2-3.c700/130/2508	succinate dehydrogenase [ubi	148237169	NP_00108347	80.04	21.99	0	0	0	0.14	51.49	54.2	0.27	0	9.04	19.37
1-2.c30843/1/1681	PREDICTED: dehydrogenase	224051869	XP_0022004(	10.42	0	15.63	5.15	6.5	3.09	0	0	0	0	5.35	0
2-3.c1219/130/2348	inner-ear cytokeratin [Rana ca	6980088	AAF34720.1	0.51	0	0	0	0	0	3.84	5.99	0.76	5.84	0	0
1-2.c34719/1/1705	PREDICTED: male-specific	513163754	XP_416839.2	1.07	10	2.6	3.08	1.91	2.19	0	0	0	0	0	0
1-2.c35453/1/1427.	--	--	--	10.78	11.74	5.43	0.07	0	1.32	8.02	13.2	7.02	0.56	13.8	12.3
1-2.c9778/5/1817	PREDICTED: nebulin isofor	847166508	XP_01282661	4.88	10.19	0	0	0	0	18.73	22.54	0	0.27	0	0
2-3.c54975/1/2031	--	--	--	2.73	2.76	2.94	2.06	1.88	2.38	0	0	0	3.91	5.05	0
1-2.c43474/1/1404	uncharacterized protein LOC	148223946	NP_00108465	2.68	0	0	0	0	0.9	5.46	5.63	10.62	22.59	36.16	11.21
2-3.c33273/1/1914	myosin, heavy chain 4, skelet	47575800	NP_00100124	13.25	1.65	76.79	0	0	2.33	20.5	113.88	78.65	0	0	0
2-3.c5957/4/2234	--	--	--	6.44	24.4	4.99	0	0.35	0	8.36	1.59	6.45	0	1.85	14.58
1-2.c49551/1/1243	actin-related protein 10 [Xen	58332420	NP_00101100	0	0.59	2.39	0	0	0	2.32	2.99	3.9	10.11	1.46	0
2-3.c35404/1/2568	PREDICTED: protein PRRC	512859823	XP_00294134	16.48	20.84	0	0	13.44	13.26	0	0	0	10.53	1.91	13.85
2-3.c63257/1/2048	collagen, type 1, alpha 2 prec	147898763	NP_00108072	2.95	617.17	3.42	4.07	1.95	15.44	63.45	54.12	96.38	38.29	7.45	2.58
2-3.c49296/1/2507	PREDICTED: IQ and ubiquit	641752321	XP_00817099	13.22	1.19	0.39	11.95	10.15	7.29	0.33	0.82	0.57	0.82	13.16	12.59
2-3.c13071/3/2427	methionine-R-sulfoxide reduc	261399890	NP_00100497	4.1	0	1	0.99	0.9	3.11	0	0	0	0.46	0	0
1-2.c37339/2/1496.	PREDICTED: tropomyosin b	617601237	XP_00752287	0	139.27	32.07	0	0	0	31.91	0	40.79	124.6	0	147.29
1-2.c45650/1/1695	PREDICTED: myosin-4-like	847156931	XP_01282446	1.23	0	1.56	0	0	0.03	4.25	1.61	1.7	0	0	2.32
1-2.c28402/1/1354	--	--	--	0.87	0.11	0.16	0	0	0	0.81	3	4.36	4.02	0.01	0
2-3.c23229/2/2172	PREDICTED: protein FAM6	301615719	XP_00293731	2.01	6.55	1.09	1.13	2.04	2.45	0	0	0	0	0	0
2-3.c47178/1/2228	--	--	--	6.77	0.82	0	0	0	0.1	3.34	7.79	2.44	11.54	13.49	14.98
2-3.c46364/1/2139	PREDICTED: plakophilin-4	847164041	XP_01282604	0.15	8.86	2.64	0	0	0	0.38	3.22	2.79	0.02	2.07	8.72
3-6.c6141/1/3008	PREDICTED: nebulin-like is	617383919	XP_00754735	0	0	52.07	0	0	0	0	20.73	13.97	0	0	0
2-3.c41598/1/2074.	PREDICTED: uncharacterize	847139136	XP_01282091	20.85	10.47	0	0.12	0.34	0.41	19.41	6.87	9.48	11.91	1.51	0.93
1-2.c23568/1/1373	PREDICTED: lupus La prote	591363494	XP_00705665	0	11.04	3.96	1.11	2.92	6.72	0	0	0	3.05	0	3.14
2-3.c1306/80/1927.	larval type I keratin [Rana ca	134140868	ABO61147.1	3.31	11.63	1155.19	2.95	0	1.25	3.77	1078.23	1031.32	86.63	0	103.7
3-6.c4097/1/3792	mediator of RNA polymerase	148224252	NP_00109072	0.41	0.06	0	0	0	0	0.44	0.82	1.27	0.66	1.31	0.78
1-2.c48729/1/1546	PREDICTED: eukaryotic tra	558148079	XP_00612003	0	0	0	0	0	0	1.95	0.85	4.27	14.93	4.79	0
2-3.c61413/1/2273	--	--	--	0	18.06	0	0	0	0	0	9.77	15.98	0	0	0
1-2.c37470/19/1514	v-ral simian leukemia viral o	156717550	NP_00109631	5.38	12.02	0	0	0	0	3.26	3.12	0.42	0	20.66	6.72
2-3.c53678/1/2292	--	--	--	4.78	6.8	3.17	0.19	0	0.14	1.26	5.37	5.55	0	2.23	0
2-3.c17331/6/2356	PREDICTED: protein FAM6	301615719	XP_00293731	0	0	0	0	0	0	0.38	1.72	1.93	0	0	0.79
1-2.c31638/3/1403	PREDICTED: fumarate hydr	512842641	XP_00293586	2.87	0	0.13	0.01	0	0	12.13	0.2	12.59	3.32	3.46	0
1-2.c44164/1/1639	PREDICTED: AMP deamina	513224977	XP_00493506	6.48	0	0	0	0	0	14.79	0	12.88	0.74	0	0.93
2-3.c7513/1/1911.1	PREDICTED: uncharacterize	847139136	XP_01282091	8.49	3.99	0	0.08	0	0.06	7.57	2.65	2.18	4.9	0.92	0.92
2-3.c14444/1/2610	LINE-1 reverse transcriptase	808857515	KKF10076.1	5.43	3.08	0.02	0.11	0.03	0.13	5.08	2.05	1.69	2.49	1.49	0.48
3-6.c16909/7/3127	PREDICTED: cytochrome P	512848263	XP_00293789	0	0	0.51	0.6	0.72	2.16	0	0	0	0	0	0.78
2-3.c37840/15/2079	PREDICTED: integrin beta-l	512821557	XP_00293713	0	19.57	2.3	7.32	5.24	32.67	0	0	0	0	0	0
2-3.c28118/1/2205	PREDICTED: UBAP1-MVB	301618738	XP_00293876	0.08	1.49	0.5	0.18	0	0.07	2.38	2.75	3.85	3.56	4.75	0.26
1-2.c25777/1/1573	PREDICTED: nebulin isofor	847166510	XP_01282661	0	0	0	0.06	92.06	270.19	0	0	0	8.68	0	0
2-3.c6877/2/2237.1	methionine-R-sulfoxide reduc	261399890	NP_00100497	1.08	0	9.14	2.28	4.15	2.17	0	0	0	0	0	0
2-3.c47163/1/2532	DNA replication licensing fac	148232250	NP_00108146	0	0	0	0.5	1.31	2.68	0	0	0	0	0	0
1-2.c28549/1/1526	--	--	--	0.16	17.71	0.06	0	0.08	0	36.1	9.18	8.01	67.28	49.37	0
2-3.c5912/1/2552.1	--	--	--	0.22	6.67	0	0.08	0.04	0.05	3.49	2.48	1.59	0.21	2.69	0
2-3.c28437/1/2093.	PREDICTED: probable 28S	301624894	XP_00294172	0	0	0	0	0	0	2.83	1.49	0.86	0.97	4.43	1.44
2-3.c55119/1/2100	PREDICTED: E3 ubiquitin-p	821477217	XP_01240197	0.35	0	0	0	0	0	4.71	0.96	1.67	0.01	1.76	2.12
2-3.c16859/1/2341	Uncharacterized protein B04	677429359	KFQ30293.1	14.29	4.87	3.48	2.74	0	4	31.81	18.5	16	3.01	9.56	7.23
1-2.c50019/1/1346	PREDICTED: hydroxymethy	591387351	XP_00706809	0	0	0	4.61	6.17	0.67	0	0	0	0	0	0
2-3.c40246/2/1975	PREDICTED: protein-L-isoa	395506555	XP_00375759	0	1.5	2.88	0	0	0	2.28	2.09	0.27	0.69	0	0
2-3.c26644/1/2488.	LINE-1 reverse transcriptase	808857515	KKF10076.1	17.67	7.94	0	0.14	0	0	13.47	5.99	5	4.67	0.52	0.38
2-3.c16875/2/2353	fragile X mental retardation s	147899444	NP_00108178	0	0	0	2.27	0.72	1.28	0	0	0	0	0	0
2-3.c16025/1/1745	PREDICTED: heat shock cog	512854037	XP_00293757	0	0	0	0	0	0	79.21	0	36.46	261.1	456.45	454.7
1-2.c9795/1/1741	PREDICTED: guanine nucle	395821567	XP_00378410	2.83	7.27	1.72	0.35	2.41	3.57	0	0	0	0	0	0
2-3.c19155/1/2496	--	--	--	0	0	7.99	0	0	0	0	11.06	8.3	7.38	0	0
1-2.c28213/2/1626	--	--	--	10.73	7.18	8.14	0	0	0	12.14	0.16	9	0	0	33.34
2-3.c48994/1/2294	--	--	--	0	0	0	0	0	0	1.45	1.69	0.72	1.21	0.91	0

2-3.c31996/3/2057	PREDICTED: basic salivary	694948810	XP_00942313	1.37	0	0	2.04	1.83	1.26	0	0	0	0	0	4.17
2-3.c4883/1/2005	--	--	--	0	0	0	0	0	0	7.3	6.25	0	0	18.82	0
2-3.c40145/1/2199	cellular retinoic acid-binding	25453404	NP_058940.1	6.37	2.09	0.28	3.49	3.77	8.48	0.75	0.03	0	1.1	0	24.01
2-3.c51969/2/2233	succinate dehydrogenase [ubi	148237169	NP_00108347	0	13.27	0	0	0	0	22.3	10.9	0	0	0	0
2-3.c10298/4/2109	PREDICTED: NADH-ubiqui	697482752	XP_00967305	0	0	0	0	0	0	7.75	0	6.79	8.05	0	0
1-2.c9072/3/1269	PREDICTED: serine/threonin	768407032	XP_01159967	5.24	0.02	0	0	0	5.24	29.8	12.82	14.35	98.75	144.78	64
1-2.c29157/1/1469	PREDICTED: triadin isoform	847130849	XP_01281908	0	1.79	0.26	1.02	0	0	158.59	137.67	0	85.29	0	0.6
2-3.c39293/1/2614	WD repeat-containing protein	147901327	NP_00109144	0	1.14	0	0	0	0	0.7	0.66	2.49	0	0.22	0
2-3.c40566/1/2374	--	--	--	0.33	0	0.58	0	0	0	0.74	2.1	0.73	0	1.17	0.03
2-3.c32217/1/2449	kelch-like protein 40 [Xenopu	147902760	NP_00109046	0	0	0	0	0	0	1.31	0.33	2.03	3.43	0	0
2-3.c59310/1/2520	--	--	--	6.68	1.47	0.27	0	0	0.19	1.04	4.94	3.46	5.36	0.24	2.82
1-2.c14614/4/1333	eukaryotic translation initiatio	148235981	NP_00108766	0	0	0.62	0	0	0	10.49	9.42	0	19.07	33.14	18.18
2-3.c44046/5/2067	transcription factor jun-B [Xe	113205822	NP_00103795	0	0	0	0	0	0	7.07	0	9.3	0	0	0
2-3.c36189/1/4603	PREDICTED: myosin-4 [Xen	847156920	XP_01282446	759.56	0	0	0	0	6.74	0	1071.9	904.71	0	0	0
2-3.c45487/1/2394	--	--	--	3.04	1.69	3.14	3.81	4.85	7.64	0.29	0.39	0.18	1.05	1.29	22.06
2-3.c28113/4/2290	PREDICTED: NADH-ubiqui	641774292	XP_00817072	0	8.58	0	0.02	18.86	12.46	0	0	0	1.54	0	0
1-2.c11354/2/1921	PREDICTED: nebulin isoform	847166510	XP_01282661	0	0	0	0.56	91.29	80.93	0	0.42	0	0	0	0
2-3.c13577/1/2627	PREDICTED: ATP-dependen	512849013	XP_00293318	1.01	1.81	0	0	0	0	2.2	1.19	0.27	0	0.92	0
3-6.c10600/1/3071	PREDICTED: probable ATP-	556977386	XP_00599596	0.72	3.44	0	0	0.06	0	2.37	2.08	0.34	0	0	1.27
1-2.c10771/3/1362	Myozenin-1-like [Xenopus la	148237556	NP_00107945	1.78	3.58	881.18	1.67	0.78	1.89	551.9	0.5	674.72	82.41	0	0
1-2.c27356/3/1487	PREDICTED: eukaryotic init	847126504	XP_01281807	55.75	23.11	44.57	0	0	0	15.87	0	28.54	0	0	7.69
1-2.c38885/1/1256	--	--	--	14.98	9.8	0	0	1.33	4.1	21.98	14.47	21.84	87.81	112.26	26.23
2-3.c36023/1/1917	--	--	--	3.8	22.93	0	0	0	0	32.49	8.31	3.68	8.11	13.05	7.25
2-3.c55318/1/2062.	--	--	--	11.9	0.74	0	0	0	0	9.77	9.63	0	12.84	14.04	19.65
1-2.c45548/1/1384	trimeric intracellular cation cl	148237167	NP_00107953	0	6.36	26.77	0	14.69	13.22	0	0	0	0	0	0
1-2.c29870/1/1679.	larval type I keratin [Rana cat	134140868	ABO61147.1	0.36	4.1	428.62	1.32	0.97	0.84	1.59	510.38	433.88	59.51	9.46	78.45
2-3.c55263/1/2348	RAB21, member RAS oncog	148230174	NP_00108340	0.34	6.99	0	0	0	0	0	4.26	6.04	8.12	3.59	0
2-3.c27951/1/2210.	--	--	--	12.54	4.37	24.98	0	0	0	22.53	1.25	17.4	0	0.75	2.88
1-2.c11045/2/1314	PREDICTED: ATP-binding c	512859677	XP_00293951	0	0	3.07	0.26	0	0	16.01	4.89	4.45	0	0	0
1-2.c11892/2/1245	annexin A5 [Rana catesbeian	148726782	BAF63788.1	9.01	19.98	2.69	0	0	0	11.31	0	15.52	20.87	12.95	23.48
2-3.c8960/1/2111	--	--	--	0	4.79	0	0	0.01	0	2.87	0.15	2.54	6.95	3.99	4.02
3-6.c13001/2/2963	PREDICTED: DNA-binding	620966761	XP_00765655	0	0	5.06	0	0	0	5.06	0	8.21	23.28	20.39	13.25
1-2.c47622/2/1291	polyubiquitin-C [Cricetulus g	346986300	NP_00123130	160.6	0	0	12.21	13.32	27.74	172.69	163.51	80.54	200.17	642.94	407.35
2-3.c3380/2/2450	PMCA1bx [Rana catesbeiana	12963455	AAK11272.1	0	0.65	0	0	0	0	4.64	3.66	0	7.55	8.15	14.5
2-3.c37579/2/1993	protein phosphatase 3, catalyt	148235473	NP_00108737	0	0.16	0	0.56	1.84	2.57	0	0	0	0.9	0	1.61
1-2.c20088/1/1464	PREDICTED: probable 28S r	301608151	XP_00293365	4.03	5.72	1.95	0	0.01	0	7.38	1.48	2.33	0	2.71	2.7
1-2.c25274/2/1318	PREDICTED: dehydrogenase	224051869	XP_00220040	26.92	1.05	23.22	18.96	14.48	7.77	0.92	1.14	0.86	13.76	32.49	22.62
1-2.c15648/1/1568	poly(rC) binding protein 3 [X	148226696	NP_00108012	0	0	0	10.54	0	9.76	0	0	0	0	60.16	0
1-2.c35081/1/1692	oxidative-stress responsive 1	148228567	NP_00108693	0	0	0	0	0	0	2.38	2.21	0.43	0	0.38	3.73
1-2.c45603/1/1340	alpha1 type II collagen [Cync	5360532	BAA82043.1	1.76	0	4.1	3.13	3.07	0.09	24.29	19.15	23.06	10.06	9.6	0
2-3.c38650/3/2725	stromal interaction molecule	45433580	NP_991397.1	0	20.25	2.35	3.16	0.06	4.06	0	0	0	0	0	17.52
2-3.c46251/1/2268.	--	--	--	13.68	1.25	0	0	3.04	0.96	29.78	21.77	17.48	130.74	221.7	21.94
1-2.c41479/1/1217	--	--	--	0	0	0	0	0	0	20.61	0	13.28	422.26	813.44	292.56
1-2.c11980/1/1891	--	--	--	0	15.89	3.74	0	5.87	7.43	0	0	0	8.08	4.61	0
2-3.c48941/1/2702	PREDICTED: Krueppel-like	512821706	XP_00293197	0	2.25	0	0	0	0	4.44	3.15	0	0.17	0	17.77
2-3.c48088/1/2524	ovarian fibroin-like substance	10954052	AAG25718.1	0	2.65	0.69	0	0	0	1	0.73	1.49	0.08	0	0.68
1-2.c43575/1/1340	PREDICTED: TATA-binding	823470855	XP_01242417	2.47	0	0	1.08	0	0	10.75	3.42	6.88	4.55	14.62	8.49
1-2.c2265/25/1452	MGC80266 protein [Xenopus	147904447	NP_00108550	32.86	33.95	0	0	0	0.09	33.55	19.72	0	0.14	75.7	55.52
2-3.c47427/1/1986	PREDICTED: homeobox pro	847152597	XP_01282325	0	1.13	0.11	0	0	0	1.18	2.17	0.53	0.7	0	0
1-2.c43972/1/1382	cystathionine gamma-lyase [X	148238287	NP_00108798	3.59	0	14.3	21.86	12.23	3.54	0	0.2	0.15	0	0	0
2-3.c37251/1/2448	--	--	--	1	2.82	1.33	0.75	0.98	2.13	0	0	0	0	0	3.08
2-3.c55244/1/2548	4-aminobutyrate aminotransfe	189230071	NP_00112152	3.39	0	1.71	2.3	1.81	0.18	0	0	0	1.23	0	0.6
2-3.c17277/1/2466.	CD99 [Xenopus laevis]	213626008	AAI69812.1	2.12	0	0	0	0	0	1	1.51	1.03	0	0	4.45
1-2.c38547/2/1333	PREDICTED: kinectin isofor	847152938	XP_01282336	0	0	0	0	0	0	0	5.92	7.32	0	0	0
3-6.c14965/1/3295	glucosidase, alpha; neutral AI	148232724	NP_00109123	0	11.08	2.52	4.23	5.99	1.95	0	0.25	0.56	1.83	0	0.69
2-3.c5196/1/2126	PREDICTED: cordon-bleu pr	847166697	XP_01282666	0.51	1.8	0.54	0	0	0	0.45	1.95	1.17	0	0.42	0
2-3.c62557/2/2324	PREDICTED: eukaryotic pep	560964464	XP_00620464	5.42	5.08	0	0	5.54	9.91	0	0	0	0	0	1.52
2-3.c1567/35/2478	PREDICTED: myosin-4-like	847156931	XP_01282446	60.32	0	0	0	0	1.03	0	279.25	178.47	0	0	37.7

2-3.c60993/1/2206	PREDICTED: matrin-3-like i	525012774	XP_00505399	3.94	9.65	0.14	0	4.73	5.09	0	0	0	0	0	5.2
1-2.c19344/1/1741	--	--	--	0	2.81	0	0.08	0	0	1.07	3.18	1.74	12.55	9.45	0
1-2.c39465/1/1514	--	--	--	8.53	0	0	0	0	0	12.35	6.96	0	20.66	24.89	19.34
2-3.c43312/2/2496	glutamate dehydrogenase 1, r	58331978	NP_00101113	0	9.02	25.69	15.94	0.32	8.81	0	0	0	0	0	0
3-6.c15641/1/10736	--	--	--	0	0	4.22	0	0.46	0	6.16	4.78	1.79	0.55	2	0
1-2.c28168/1/1493	--	--	--	0	0	0	0.21	0.19	0	4.63	2.78	4.34	19.33	38.21	0.44
2-3.c61026/1/2382	PREDICTED: adseverin [Xei	301619350	XP_00293905	8.04	0	8.6	2.22	7.63	4.48	0.68	0	0	0	0	6.71
2-3.c14714/1/1906	DEK oncogene [Xenopus lae	291290881	NP_00116747	4.32	0	0	0	4.8	6.68	0	0	0	1.1	1.34	0
1-2.c25466/1/1423	basic leucine zipper and W2 c	147906588	NP_00108029	0	59.94	0	0	8.94	16.42	0	0	0	35.79	0	0
1-2.c35534/1/2703.	--	--	--	3.12	2.29	0	0	0	0	6.3	0.9	4.05	22.11	28.72	8.86
2-3.c41598/1/2074.	endonuclease/reverse transcri	148645276	ABR01162.1	17.33	5.6	0	0	0	0	17.51	5.32	4.21	4.07	0	0.36
1-2.c35568/2/1345	S-adenosylmethionine syntha	45361639	NP_989395.1	0.81	0	0	5.01	0	0	46.57	13.27	22.79	20.79	32.34	0
1-2.c27027/1/1874	glutamate dehydrogenase 1, r	58331978	NP_00101113	0	0	0	0	0	0	4.26	4.61	0	0	3.85	0
2-3.c22611/1/2344.	--	--	--	35.28	10.54	1.24	1.78	1.69	0	8.15	21.89	13.96	32.25	1.48	24.28
1-2.c24095/1/6125.	isocitrate dehydrogenase 2 (N	148227256	NP_00108032	45.8	3.66	44.97	37.83	49.35	30.19	0	0	8	0	0	0
1-2.c46860/1/1190	--	--	--	12.33	29.67	0.71	0	0	0	6.93	7.01	0	38.22	67.53	37.23
1-2.c19271/1/1510	PREDICTED: NACHT, LRR	699666029	XP_00988359	17.78	2.67	0.95	0.82	0.89	2.03	17.51	8.44	11.86	20.28	25.75	32.06
2-3.c27778/1/1918	nuclear factor, erythroid 2-lik	147900041	NP_00108001	1.49	0	0	0	0	0	13.08	6.02	0.1	13.74	9.63	31.8
2-3.c29051/1/2312.	--	--	--	1.93	0	0	0	0	0	3.92	2.64	2.19	21.56	12.51	28.87
2-3.c5455/9/2421	PREDICTED: protein FAM6	301615719	XP_00293731	4.6	7.2	2.96	3.03	3.88	2.47	0	0.29	0.12	6.29	7.59	0
1-2.c19662/3/1297	--	--	--	4.34	0	0	0.63	0	0	10.46	1.46	9.02	13.17	26.67	0.19
3-6.c17786/15/3106	PREDICTED: alpha-actinin-:	602667068	XP_00743910	142.97	12.66	388.36	278.54	208.79	404.92	44.6	108.89	135.4	0	0	0
2-3.c42464/1/7638.	PREDICTED: LOW QUALI	700363815	XP_00992652	684.81	0	548.35	629.31	553.97	261.4	575.77	418.7	0	0	0	0
1-2.c48431/1/1352.	PREDICTED: olfactomedin 4	847154698	XP_01282391	0	0	0	0	0	0	0.17	0	0	659.08	442.44	988.28
1-2.c3369/2/1167	--	--	--	0	0.86	0	0	0	0	267.03	0	46.65	445.11	652.17	1288.28
1-2.c15452/2/1533.	matrix metalloproteinase-9TH	147901642	NP_00109130	1.38	0	0	0	0.3	0	6.38	0.62	0	772.11	604.96	1159.04
1-2.c21064/8/1952	solute carrier family 37 (gluc	147901932	NP_00108340	19.59	3.1	103.33	80.29	76.82	124.98	41.26	46.55	105.76	0	0	0
2-3.c42102/1/2100	LOC398634 protein, partial [	32450475	AAH53779.1	0	2.62	0	0	0	0	0	0	0	130.95	109.33	75.66
1-2.c52436/7/1287	PREDICTED: ATP synthase	557324504	XP_00603528	0	0	0	0	0	0	113.67	0	0	126.78	244.06	158.14
1-2.c42638/3/1834	polyadenylate-binding protein	52346016	NP_00100505	92.9	165.26	64.37	71.14	50.64	27.27	35.69	49.57	28.26	0	0	0
1-2.c28637/1/2058.	LOC494693 protein [Xenopu	52138935	AAH82684.1	4.77	0.74	0.33	2.42	0.02	0.07	2.79	0.57	0	2292.79	1806.27	1241.13
1-2.c35595/2/1825	F-actin-capping protein subu	161086978	NP_00110309	0.21	34.24	37.47	21.21	33.38	51	0.36	20.33	0	0	0	0
2-3.c42736/1/2384	Apolipoprotein A-I-like precu	148227056	NP_00107939	1.88	3.75	1.17	25.9	47.54	8.54	37.8	20.98	19.07	24705.84	42592.67	45497.04
1-2.c38617/1/1705.	matrix metalloproteinase-9TH	147901642	NP_00109130	14.55	5.09	2.86	16.84	2.58	3.87	59.27	22.11	12.17	10239.52	10289.61	7741.04
1-2.c3235/1/1394.2	--	--	--	35.89	0	1.74	20.77	4.22	0	33.93	2.91	2.06	12189.75	9318.57	7750.28
3-6.c8004/1/2790.1	PREDICTED: ethanolamine-	641767307	XP_00816835	0.67	0.92	1.37	0	0	0	10.24	1.32	0	201.64	181.22	96.13
1-2.c12894/1/1436	apolipoprotein A4 precursor [	148222300	NP_00108800	4.88	1.61	0.2	0	1.26	0.12	1.58	0.59	1.54	535.26	642.07	612.71
1-2.c3650/4/1363	PREDICTED: trifunctional e	847164769	XP_01282620	0	0	0	0	0	0	2.33	0	0	119.19	227.49	92.49
2-3.c1304/108/2008	PREDICTED: protein transpo	734599634	XP_01075368	31.87	66.51	35.95	18.07	23.39	25.5	6.86	5.86	3.2	0	0	0
1-2.c33717/1/8520.	matrix metalloproteinase-9TH	147901642	NP_00109130	6.58	0	0.77	2.44	2.76	2.54	17.62	2.74	0	2525.98	2184.25	2334.95
1-2.c51791/13/1641	PREDICTED: heat shock pro	795388413	XP_01175268	0	0	0	0	0	0	0	0	0	196.94	51.27	160
1-2.c1527/106/1399	apolipoprotein A4 precursor [	148222300	NP_00108800	2.79	0	0	2.39	0	0	1.28	0.5	0	647.65	616.62	853.61
2-3.c63465/6/2077	ATPase, Na+/K+ transporting	148229987	NP_00108258	20.89	30.91	15.78	19.73	23.69	12.74	0	0	28.07	0	0	0
1-2.c43857/1/1238	V-type proton ATPase 16 kD	60678280	NP_991117.1	0	0	0	0	0	0	0	0	0	77.98	67.69	73.97
1-2.c44872/1/1337.	PREDICTED: tropomyosin b	505767831	XP_00460027	0	0	0	0	0	0	0	0	2.46	144.31	117.85	80.32
1-2.c31902/2/1174	PREDICTED: tropomyosin a	847143254	XP_01282187	0	0	0	0	0	0	0	0	0	81.14	65.4	55.75
2-3.c5808/1/7973.1	matrix metalloproteinase-9TH	147901642	NP_00109130	5.95	1.3	0	2	1.3	0.44	8.74	3.35	2.62	847.41	640.81	1356.5
1-2.c10670/9/1416	obg-like ATPase 1 [Xenopus	148228515	NP_00107968	21.54	18.89	55.39	30.6	17.55	28.29	34.76	27.41	0	0	0	0
1-2.c43622/1/1269	--	--	--	0	0	0	0	0	0	9.84	0	0	68.93	83.9	28.43
1-2.c11543/1/1301	NADH dehydrogenase 1 alph	226371850	ACO51550.1	29.41	10.04	17.19	27.31	45.06	28.12	0	15.1	27.61	0	0	0
2-3.c29390/1/2341	Mmp-9th protein [Xenopus la	124504280	AAI28677.1	3.34	0.28	0.1	2.85	0.32	0.31	6.68	3.54	3.1	599.63	489.56	949.35
2-3.c15571/2/2133	--	--	--	0	4.04	0	0	0	0	11.92	0	0	32.27	18.2	35.39
1-2.c12164/1/1434	PREDICTED: isocitrate dehy	831547357	XP_01272691	210.39	76.36	163.13	129.79	77.43	39.06	0.01	75.1	0.17	0	0	0
1-2.c38469/1/6160.	PREDICTED: telethonin [Xe	301622194	XP_00294041	62.92	51.94	83.02	36.67	66.33	53.49	13.35	47.65	34.95	0	0	0
1-2.c39401/1/1023	Apolipoprotein A-I-like precu	148227056	NP_00107939	82.55	5.49	1.06	45.88	5.45	1.61	49.08	6.99	7.92	7385.49	7255.37	11197.63
3-6.c15224/1/3466.	TCDD-inducible poly [ADP-	163914785	NP_00110641	0	0	0	0	0	0	0.32	3.68	0	106.85	86.51	33.54
2-3.c11181/294/202	PREDICTED: telethonin [Xe	301622194	XP_00294041	93.06	51.43	94.03	33.28	94.01	110.27	24.77	47.91	26.15	0	1.09	0.36
1-2.c40985/1/1439	actin, alpha cardiac muscle 2	45361557	NP_989355.1	27.18	7.1	38.76	12.58	20.85	28.44	4.34	40.31	2.32	0	0	0

2-3.c9812/1/2365	--	--	--	2.8	82.43	7.53	8.32	9.82	23.28	4.03	3.76	5.2	0	0	0
1-2.c14294/4/1468	LOC445827 protein, partial [	49899736	AAH76748.1	0	0	0	0	0	0	0	0	0	39.23	48.92	24.52
1-2.c42964/2/1354	keratin 8 [Rana catesbeiana]	15341208	BAB63946.1	0	0	0	0	0	0	0	0	6.62	294.93	416.48	98.7
1-2.c38190/1/1834	--	--	--	0	0	0	0	0	0	0	0	0	27.26	37.93	19.65
3-6.c4316/4/2803	RecName: Full=Casein kinas	20141531	P28020.2	13.36	23.62	2.46	5.47	19.12	13.92	7.9	9.63	3.02	0	0	0
1-2.c33102/1/1245	ubiquinol-cytochrome c redu	147902934	NP_00107971	0	0	0	0	0	0	0	0	0	53.92	43.44	31.87
1-2.c4247/1/1347.1	NADH dehydrogenase [ubiqu	58332714	NP_00101143	60.23	16.51	64.19	33.32	48.3	16.07	10.64	22.46	0	0	0	0
3-6.c3079/4/2849	PREDICTED: DDB1- and CU	847088170	XP_01281968	2.08	3.12	8.42	7.97	9.49	4.87	5.14	4.25	5.78	0	0	0
1-2.c39832/1/1457	2-oxoglutarate dehydrogenas	71897293	NP_00102655	0	1.5	0	0	0	0	10.35	0	1.42	34.44	46.51	19.45
1-2.c42164/4/1511	PREDICTED: LOW QUALIT	641786874	XP_00531376	15.12	36.35	18.22	9.24	27.33	31.52	7.76	0	5.33	0	0	0.14
3-6.c14709/1/3340	PREDICTED: 5'-nucleotidas	530647152	XP_00531019	1.32	3.92	3.02	2.64	6.97	10	4.98	4.9	6.21	0	0	0
3-6.c2822/9/3431	PREDICTED: eukaryotic pep	560964464	XP_00620464	0	0	0.25	3.72	5.01	9.82	7.28	2.66	0	0	0	0
1-2.c31922/8/1613	periostin precursor [Xenopus	163915007	NP_00110637	78.3	242.23	151.1	75.1	127.47	82.22	101.92	70.4	63.73	0	0	2.02
1-2.c35598/1/1274.	ribosomal protein, large, P0 [	147904649	NP_00108013	627.66	1500.65	804.62	445.79	545.36	596.68	210.29	348.37	242.08	1	0	11.2
1-2.c44333/1/1325	MAM domain-containing pro	118403772	NP_00107284	0.55	2.41	0	0	0.25	0	1.81	0	1.25	76.6	79.26	48.08
1-2.c43281/1/3847.	PREDICTED: actin, cytoplas	795093282	XP_01181546	1.11	0	0	0	0	0	1.57	1.07	0	43.34	27.06	88.5
1-2.c23595/1/7023.	beta-actin [Scomberomorus n	836592739	AKM70876.1	0	0	0	0	0	0	0	0	0	91.17	45.32	160.34
1-2.c5988/17/1605	tomm22 protein [Xenopus (S	169642622	AAI60473.1	15.83	20.5	12.2	11.58	18	7.34	10.46	9.59	6.71	0	0	0
1-2.c27273/8/1480	novel aldo-keto reductase fan	113931624	NP_00103926	16.96	4.12	27.98	25.5	27.92	16.47	12.89	14.29	18.43	0	0	0
2-3.c17057/1/5105.	PREDICTED: serine proteas	301606403	XP_00293282	33.8	0	5.98	14.56	13.27	4.85	0	14.17	0.55	0	0	0
1-2.c49686/1/1384	--	--	--	16.71	0.89	0	0	0	0	0	0	12.37	31	35.1	28.95
1-2.c19767/1/1482	protease, serine, 8 precursor [	148222777	NP_00108172	1.47	2.64	3.71	1.79	0.39	0	11.2	2.82	3.22	224.97	319.57	200.23
1-2.c9009/3/1421	RecName: Full=Casein kinas	20141531	P28020.2	20.44	24.31	4.06	14.44	13.18	13.61	7.32	6.49	11.97	0	0	0
2-3.c16603/1/1966	reverse transcriptase, partial [	513131148	AGO18322.1	15.79	1.95	6.29	9.7	6.65	15.75	5.74	15.84	6.05	0	0	0
1-2.c48567/1/1601	PREDICTED: protein phosph	512830294	XP_00491303	20.83	3.66	12.71	17.44	21.76	11.57	8.39	20.14	16.76	0.14	0	0
1-2.c13686/6/1656	PREDICTED: NAD(P) transl	768393841	XP_01159260	42.93	0	0	0	0	0	0	0	39.03	22.84	18.24	27.02
1-2.c44800/2/1252	golgi reassembly stacking pro	89267909	CAJ82834.1	11.41	0.18	6.72	0	0	0	7.75	0	6.12	29.13	42.24	22.43
2-3.c49393/1/2313	--	--	--	0	0	0.71	0	0	0	0	0.12	3.25	13.54	19.95	12.27
1-2.c25705/3/1472	--	--	--	0	0	0	0	0	0	12.69	0	0	67.1	112.51	32.02
2-3.c28061/1/2114.	--	--	--	0	0	1.06	0	0	0	0	0	0	10.64	22.6	18.96
2-3.c40548/2/1995	PREDICTED: 5'-3' exoribonu	301620220	XP_00293947	4.31	27.14	4.12	3.04	9.2	12.14	2.38	2.29	2.84	0	0	0.01
2-3.c11883/2/2376	PREDICTED: AMP deamina	706103160	XP_01020846	0	41.66	171.37	54.96	85.51	162.79	0	132.08	0	4.06	0	0
2-3.c48609/1/2373.	--	--	--	0	0	0	0	0	0.28	16.22	4.85	0	117.94	107.8	49.06
1-2.c2253/15/1434	PREDICTED: voltage-depen	823417697	XP_01241959	72.46	76.05	121.34	67.02	88.26	108.58	45.42	61.5	69.85	0	0	2.8
1-2.c46934/2/1201	tubulin beta chain-like [Scler	820146195	KKX13732.1	0	0	0	0	0	0	0	0	0	158.55	146.74	33.58
2-3.c49731/1/2071.	--	--	--	0	0	0	0	0	0	0	0	0	35.87	39.37	69.3
2-3.c38214/47/2580	RecName: Full=Sarcoplasmic	9789732	Q92105.1	303.67	0	0	298.7	378.34	236.23	0.02	540.95	0	0	0	12.42
2-3.c63564/3/1811	PREDICTED: elongation fac	821107511	XP_01237909	0	0	0	0	0	0	0	0	0	162.15	51.59	66.75
1-2.c49773/1/1338	heterogeneous nuclear ribonu	45361501	NP_989327.1	0	0	0	0	0	0	0	0	7.36	33.13	25.3	17.6
1-2.c43826/1/1255	PREDICTED: E3 ubiquitin-p	847100968	XP_01281275	0	0	0	0	0	0	8.19	0	0	30.41	41.77	15.38
1-2.c52310/5/1399	PREDICTED: ATP synthase	557324504	XP_00603528	251.51	166.21	177.83	219.65	154.41	125.55	164.4	221.43	290.72	5.66	0	1.82
2-3.c12984/1/2041	uncharacterized protein C6or	187608789	NP_00112027	0	8.51	0.33	0	0	0	7.37	0	0	21.11	11.21	12.22
2-3.c28064/2/2107	PREDICTED: ATP-depender	551492924	XP_00579803	16.04	16.29	0	0	0	0	16.01	5.26	0	14.14	14.71	14.22
1-2.c9417/11/1793	homogentisate 1,2-dioxygena	148230238	NP_00108750	25.61	25.05	24.56	10.66	9.64	5.07	7.91	13	11.22	0	0	0
1-2.c51905/2/1359	eukaryotic translation initiati	147901729	NP_00108058	7.24	0	0	0	0	0	2.03	0	24.57	25.27	31.64	15.06
2-3.c53810/1/5367.	larva-specific keratin RLK [R	14017762	BAB47395.1	0	84.38	0	0	0	0	0	0	150.91	118.75	753.05	796.85
2-3.c58790/1/2310.	--	--	--	9.73	1.07	0	0	0	2.14	54.03	1.94	25.07	241.02	295.51	98.47
1-2.c18674/1/1218	DCN1-like protein 2 [Xenopu	187607513	NP_00112003	6.43	6.97	13.33	7.13	14.46	18.91	1.85	11.38	0.55	0	0	0
2-3.c11717/10/2071	PREDICTED: tubulin beta ch	795248736	XP_01185447	0	0	0	0	0	0	22.01	0	0	21.26	11.79	9.91
1-2.c38348/1/1336.	serum paraoxonase/arylestera	148232543	NP_00108064	17.77	2.57	31.56	16.05	28.94	17.15	0	18.72	17.35	0	0	0
1-2.c38530/1/1252	--	--	--	9.62	0	0	0	0	0.03	4.69	0.95	0.21	20.29	31.21	33.41
1-2.c7274/6/1353	PREDICTED: nebulin isofor	847166495	XP_01282660	0	22.73	0	0	0	0	0	0	0	91.94	80.94	20
2-3.c13689/2/2027	PREDICTED: plasma membr	847108317	XP_01281394	37.68	7.95	12.1	35.66	24.7	14.4	23.23	16.2	31.97	0.73	0.21	0.01
1-2.c52174/2/1624	PREDICTED: alpha-actinin-3	558140029	XP_00611831	0	0	0	0	0	0	0	0	0	18.68	27.34	8.89
1-2.c12630/2/1300	PREDICTED: Y+L amino ac	602645598	XP_00742923	0	0.33	0	0.38	0	0.28	12.45	0	0	65.63	85.47	51.26
1-2.c39903/2/1257	glutamate oxaloacetate transa	333805525	BAK26556.1	0	0	0	0	0	0	0	0	0	48.75	57.04	14.51
1-2.c49089/1/1468	Y+L amino acid transporter 2	147902872	NP_00109124	0	0	0	0	0	0	13.65	0.01	0	25.53	20.88	15.71
1-2.c52344/2/1299	actin, alpha cardiac muscle 2	45361557	NP_989355.1	49.28	4.69	154.68	76.12	159.38	116.74	50.7	139.07	67.02	2.94	0	2.76

2-3.c3904/1/2488.1	--	--	--	25.05	7.86	32.06	5.24	13.69	20.85	2.22	11.17	8.02	0	0	0.12
2-3.c20434/3/2208	PREDICTED: ATP-depender	829804302	XP_01262791	7.17	4.3	5.03	2.84	5.92	10.82	2.59	0	0.17	0	0	0
1-2.c25477/1/1459	--	--	--	0	0	0	0	0	0	4.28	3.61	0	25.76	25.13	9.12
2-3.c25444/2/2551	PREDICTED: death-associat	847142187	XP_0128215(	8.98	0.08	8.24	7.44	8.75	3.24	2.91	0	4.83	0	0	0
2-3.c18837/1/2422.	--	--	--	2.3	1.65	0	0	0	2.27	8.21	5.31	5.6	129.2	198.5	119.1
2-3.c41903/1/2083	homeodomain-interacting prc	118404554	NP_00107275	1.82	9.27	9.11	8.12	3.06	7.73	0	4.5	9.22	0	0	0
2-3.c7657/4/2527	PREDICTED: chaperone acti	557262176	XP_00601661	4.15	12.96	10.23	5.43	6.37	2.79	2.05	2.37	5.13	0	0	0
1-2.c28454/1/1353	structural maintenance of chr	148232365	NP_00108049	0.13	0	0	0	0	0	6.64	0.26	0	17	31.07	15.25
3-6.c14596/1/3278.	hypothetical protein [Plasmoc	156094450	XP_00161326	6.55	0	20.2	8.57	10.08	3.51	1.33	15.29	6.15	0	0	0
1-2.c5593/1/1588	Ras-related GTP binding C [X	148227166	NP_0010884(	0	0	0	0	0	0	0	0	0	18.97	23.9	10.52
2-3.c39819/2/2237	uncharacterized protein LOC	189217724	NP_0011213(	0	1.41	0	0	0	0	0.58	0	1.01	8.57	10.51	14.81
1-2.c50832/6/1778	tropomyosin alpha-1 chain [X	52345962	NP_00100502	0	0	0.92	0	0	0	1.51	0	0	11.65	19.19	13.45
2-3.c9016/1/2157.1	PREDICTED: mitogen-activa	512866147	XP_00491777	7.37	2.82	16.93	6.6	7.66	4.68	4.25	8.06	7.23	0	0	0
1-2.c18629/1/1579	PREDICTED: E3 ubiquitin-p	512856132	XP_00294359	0	0	0	0	0	0	2.62	0	0	22.1	18.78	9.58
2-3.c1764/13/2275	PREDICTED: adenosine kina	874462866	XP_01295145	3.45	11.81	8.97	2.29	5.93	8.06	2.51	2.25	3.53	0	0	0
2-3.c29912/1/2077	Calnexin, partial [Anas platyr	483507247	EOA97896.1	0.22	0.75	1.1	0	0	0	0.2	0	0	8.37	9.58	17.68
1-2.c13863/3/1361	PREDICTED: MAP7 domair	512819424	XP_00491162	27.56	4.32	18.57	9.33	11.52	7.8	2.26	8.26	8.62	0	0	0
1-2.c4461/2/1493	LOC398314 protein, partial [	68534356	AAH99043.1	0	0	0	0	0	0	24.74	0	5.87	21.34	23.45	9.11
1-2.c5180/1/1513.1	--	--	--	1.36	0	0	0.31	0	0.05	0	2.43	0.32	55.2	67.89	27.47
2-3.c54534/1/2093	Uba1a protein [Xenopus laev	28958137	AAH47256.1	7.06	13.27	5.28	2.36	6.66	8.38	3.99	0	3.63	0	0	0
3-6.c4513/4/2927	PREDICTED: baculoviral IA	847099212	XP_01281215	0	0	0	0.32	0	0	0	0	5.79	23.5	15.17	39.86
1-2.c28833/2/1760	GDP dissociation inhibitor 2	148234617	NP_00108023	0	2.75	0	0	0	0	11.27	0	0	11.09	11.11	19.69
2-3.c13047/1/2370	3-hydroxyisobutyrate dehydro	71896099	NP_0010256(	16.29	26.32	19.79	21.72	17.95	19.09	11.76	15.93	16.62	0.94	0	0
1-2.c27799/2/1629	ankyrin repeat and SOCS box	148229923	NP_00108543	18.24	0	7.83	6.98	10.51	4.03	8.13	12.25	14.68	0	0	0
2-3.c23405/1/2075	PREDICTED: nascent polype	768400950	XP_01159639	72.42	28.31	55.48	51.26	39.49	90.38	34.58	48.7	55.73	0.28	3.99	0
2-3.c38919/1/2106	--	--	--	1.2	2.28	0	0	0	0	0.67	0	0	12.07	13.13	7.78
1-2.c39880/1/1956	PREDICTED: casein kinase I	847117974	XP_01281644	0.3	0	0	0	0	0	8.75	0.39	4.14	17.06	14.34	5.48
2-3.c53640/1/1784	CD63 antigen [Rana catesbei	226372482	ACO51866.1	0.05	7.6	5.37	10.81	10.46	8.4	12.88	9.06	0.04	0	0.11	0.03
2-3.c7776/1/2186	--	--	--	2.92	10.55	5.15	5.67	5.45	4.01	0	1.18	4.51	0	0	0
1-2.c12205/1/1792	thrombospondin-4 precursor		118404528	NP_00107267	17.77	41.66	25.47	19.29	15.54	21.82	5.37	22.34	25.68	0	0	0.64
1-2.c2116/28/1771	PREDICTED: ornithine amin	768381361	XP_01158831	54.79	42.47	80	74.8	56.66	93.8	35.5	41.16	58.45	0.34	2.75	1.97
3-6.c9358/4/3478	periostin precursor [Xenopus	163915007	NP_00110637	1.18	183.53	0.15	13.63	3.67	22.58	0	17.39	0	0	0	0
1-2.c7489/4/1483	heterogeneous nuclear ribonu	147903924	NP_0010805(	0	0	0	0	0	0	0	0	3.21	11.52	15.83	21.42
1-2.c51610/3/1635	PREDICTED: tubulin beta ch	823416057	XP_01241517	8.89	0	0	0	0	0	0	0	0	17.37	19.04	6.87
2-3.c14771/1/2416	--	--	--	2.87	0.97	4.44	2.23	4.98	6.19	3.88	1.75	0.69	0	0	0
1-2.c32857/2/1449	cholinephosphotransferase 1		213983097	NP_00113569	0	0	0	0	0	0	0	0	0	24.5	28.13	27.24
1-2.c45671/1/1548.	ER membrane protein comple	147906471	NP_00108517	18.51	16.18	4.91	7.98	8.57	12.97	0	2.62	3.29	0	0	0
1-2.c46319/1/1604	PREDICTED: casein kinase I	847117966	XP_01281643	2.43	12.15	3.98	7.55	8.19	5.39	0	3.81	2.57	0	0	0
1-2.c33121/1/7580.	PREDICTED: poly(rC)-bindi	847099221	XP_01281215	0	3.41	27.46	25.06	28.61	12.97	0	0	6.35	0	0	0
1-2.c41396/2/1583	Protein kinase C and casein k	148232870	NP_00107941	6.74	3.71	4.45	6.33	4.57	10.55	10.69	0.2	6.33	0	0	0
2-3.c40338/1/2041.:	--	--	--	7.68	1.3	1.09	0.95	3.35	6.87	1.77	1.38	2.81	658.55	278.78	574.47
3-6.c17475/13/3451	PREDICTED: superkiller vira	301623717	XP_00294115	1.46	13.25	2.27	2.74	2.79	3.2	0.72	2.76	1.07	0	0	0
2-3.c51746/3/2360.:	--	--	--	14.96	5.48	0	0	0	0	0	0	0	33.46	27.87	28.41
3-6.c14516/1/3159	PREDICTED: nebulin-like is	557018869	XP_0060098(	8.76	0	0.05	32.17	89.25	87.15	30.96	84.37	0	5.48	0	0
2-3.c8146/1/1992.1	--	--	--	23.75	4.47	0	16.29	10.05	7.82	0	38.73	0	0	0	0
1-2.c26130/1/1312	BTB/POZ domain-containing	187607270	NP_00112048	0	3.31	0	0	0	0	0	0.53	0.25	14.54	20.18	18.05
2-3.c62420/52/2257	gnl3-prov protein, partial [Xe	38649400	AAH63220.1	0.14	22.69	0.09	4.19	3.54	6.44	0	3.63	1.34	0	0	0
3-6.c4887/2/2789	Citrate synthase [Danio rerio]	183986405	AAI66040.1	29.26	66.54	94.87	88.55	91.35	71.89	68.74	56.47	17.83	0.7	2.8	3.34
2-3.c51390/30/2255	ankyrin-1 [Xenopus (Silurana	73853810	NP_00102748	29.19	21.83	108.7	90.27	70.19	123.99	87.84	29.06	60.03	3.04	1.69	2.77
2-3.c54357/1/2072.	PREDICTED: cystinosin isof	847100949	XP_01281274	0	0	0	0	0	0	0.55	0	0	19.7	22.96	20.38
1-2.c14836/1/1596	PREDICTED: LOW QUALIT	573880367	XP_00662838	1.62	54.16	310.5	207.18	223.46	138.06	1.52	276.59	1.94	16.8	0.27	0
1-2.c20465/2/1448	--	--	--	0	0	0	0	0	0	4.65	0	0	14.77	10.3	20
1-2.c30366/1/1319	CD63 antigen [Rana catesbei	226372482	ACO51866.1	4.52	53.64	42.34	58.62	76.57	20.03	59.37	53.24	2.3	0	0	0.88
2-3.c17712/1/2674	bystin [Xenopus (Silurana) tr	62752053	NP_00101583	7.89	7.18	4.67	4.03	3.55	5.44	3.28	3.32	2.94	0	0.05	0
2-3.c49251/1/4697.	PREDICTED: plasma membr	847108317	XP_01281394	15.77	0	5.74	10.42	14.78	3.09	5.13	1.9	7.08	0	0	0
1-2.c29698/1/1198	--	--	--	0	0	0	0	0	0	0	0.35	0	23.83	22.45	8.38
1-2.c11613/1/1633	PREDICTED: glycogen phos	426369041	XP_0040515(	4.14	0	11.82	14.33	14	3.11	12.51	9.71	6.64	0	0	0
1-2.c43038/1/1480	--	--	--	0	31.53	7.69	9.46	8.7	27.95	13.39	9.3	0.05	0	0	0

1-2.c8135/7/1864	PREDICTED: citrate synthas	557324568	XP_00603531	61.05	1.12	0.77	0.49	0.08	0.47	0.31	0.27	61.45	51.38	47.98	46.76
1-2.c10246/82/1317	mitochondrial inorganic phos	22653426	AAN04052.1	341	0	289.55	139.84	227.83	144.3	172.56	103.14	76.07	0	0	13.88
1-2.c21108/3/1509	PREDICTED: RNA-binding	301612449	XP_00293572	3.25	0	0	0	0	0	1.58	2.12	0.79	12.49	12.4	14.11
3-6.c999/47/3266	thrombospondin-4 precursor	118404528	NP_00107267	17.48	31.11	27.44	15.01	6.23	22.46	2.11	9.01	11.84	0.5	0	0.4
1-2.c44202/1/1541	Uncharacterized protein B04	677429359	KFQ30293.1	0	0	0	0	0	0	0	0	17.83	21.74	42.9	12.65
2-3.c34028/3/2472	ethanolamine-phosphate phos	147905107	NP_00108663	2.39	11.96	1.84	5.36	4.49	0	57.98	4.42	2.31	306.25	488.83	254.74
2-3.c60283/1/2759	RNA-binding protein 24 [Xe	45360685	NP_989016.1	3.63	3.09	1.38	2.79	2.43	4.44	0.79	1.15	1.49	0	0	0
2-3.c63612/1/2086	PREDICTED: aspartate--tRN	847163066	XP_01282572	0	0	0	0	0	0	0	0	0	9.41	8.39	7.95
1-2.c7003/5/1440	synaptophysin-like protein 2	54606873	NP_00100611	56.49	68.83	130.07	83.23	68	156.96	38.24	66.17	67.62	2.62	2.73	4.21
1-2.c37657/1/1489	--	--	--	61.1	56.95	139.11	80.07	46.31	98.82	85.44	79.15	49.96	3.27	0.35	3.17
2-3.c26879/1/2245	PREDICTED: calcium/calmo	847142050	XP_01282146	1.85	3.41	2.29	2.22	4.23	4.86	1.98	1.21	3.2	0	0	0
1-2.c8141/4/1761	PREDICTED: dolichyl-diphc	847168158	XP_01282717	0	0	0	0	0.3	0	0	0	0.91	21.95	20.86	25.67
3-6.c14588/1/3800	--	--	--	21.39	8.45	18.91	39.15	24.33	21.88	23.52	27.94	40.07	0	0	2.46
1-2.c29941/1/1249	--	--	--	0	0	0	0	0	0	0.96	1.85	0	20.98	12.81	10.66
1-2.c3232/1/1261	fatty acid binding protein 3, r	160774264	AAI55023.1	0	0	0	0	0	0	0	0	0	28.43	14.13	8.96
1-2.c34228/1/1368	--	--	--	0	0	0	0	0	0	0	0	0	22.07	37.63	9.79
2-3.c6464/1/9356.1	lysophosphatidylcholine acyl	148228263	NP_00108886	0	0	0	0	0	0	2.12	0	0	39.53	51.47	11.99
1-2.c15772/1/9144.	PREDICTED: LOW QUALI	637377851	XP_00812323	2447.03	387.59	0	0	0	0	5317.42	0	0	5554.9	4681.93	0
1-2.c28317/2/1270	voltage-dependent anion char	147906224	NP_00108535	0	0	0	0	0	0	0	0	0	51.74	55.66	9.82
1-2.c32943/1/8414.	PREDICTED: protein PFC07	556961274	XP_00599098	61.51	38.36	130.08	84.33	33.21	95.52	90.99	65.57	40.79	2.73	1.05	2.96
2-3.c25926/1/2216.	--	--	--	0	0	0	0.74	0	0	3.45	2.1	0	54.36	86.34	46.38
1-2.c15204/1/1023	isocitrate dehydrogenase 2 (N	148227256	NP_00108032	30.5	0	0	0	0	0	4.25	0	0	20.89	53.98	21.09
1-2.c27544/1/1407	PREDICTED: LOW QUALI	731504681	XP_01059620	3.27	2.22	0	0	0	0.19	3.2	0	0	18.07	28.73	16.65
1-2.c19175/4/1719	PREDICTED: dolichyl-diphc	597761591	XP_00724478	0	0	0	0	0	0	0	0	5.3	14.27	20.31	4.95
2-3.c7175/7/2135	acetyl-CoA acetyltransferase	148234082	NP_00108602	5.74	6.17	7.33	3.42	4.13	4.7	3.27	3.92	3.09	0	0	0
3-6.c3139/7/3219	PREDICTED: calcium-bindi	664741920	XP_00852826	0	0	7.52	2.05	3.72	1.35	0	0	5.19	0	0	0
3-6.c3508/3/3339	PREDICTED: junctophilin-2	301607262	XP_00293323	42.5	24	67.32	49.1	70.73	129.17	48.67	71.97	56.72	9.71	0	0
1-2.c11240/2/1417	PREDICTED: heterogeneous	597735333	XP_00722994	0.06	0	0	0.13	0	0	16.27	0	10.17	43.54	81.44	23.47
1-2.c45092/1/1501.	PREDICTED: UAP56-intera	512859648	XP_00491695	0	0	14.36	10.67	17.65	41.09	27.04	0	18.72	0.16	0	0
1-2.c34320/1/1412	--	--	--	2.29	0	0	0	0	0	0	0	0	12.62	11.64	11.17
1-2.c29465/1/1788	PREDICTED: dnaJ homolog	301610031	XP_00293455	0	11.2	3.9	4.71	4.56	4.94	7.04	2.5	0.07	0	0	0
2-3.c37908/1/2334	fragile X mental retardation s	147899444	NP_00108178	0	0	0	17.27	27.92	52.51	0	21.1	0	2.36	1.19	0
2-3.c62450/4/2489	PREDICTED: mitogen-activa	847126378	XP_01281804	2.86	6.45	2.76	2.25	2.33	5.01	2.23	1.68	3.22	0	0	0
1-2.c4093/10/1626	PREDICTED: rRNA 2'-O-m	558189237	XP_00612907	13.05	119.71	11.87	26.52	26.96	59.07	10.49	22.31	10.16	1.95	0.71	0.92
1-2.c37910/5/1300	PREDICTED: actin, alpha ca	803091489	XP_00401102	9.28	1.06	80.33	29.67	39.12	59.71	65.69	44.63	15.92	0.27	2.3	1.56
1-2.c32943/1/8414.	--	--	--	76.65	65.93	167.49	104.27	72.77	210.6	131.02	84.28	80.97	2.18	1.32	8.86
2-3.c55459/1/2136.	--	--	--	0	0	0	0.33	0	0	0	0	2.45	51.09	52.16	17.84
1-2.c48697/1/1502	PREDICTED: large neutral a	847086110	XP_01281357	5.12	0.8	0	0	0	0	19.46	1.12	3.67	226.73	176.68	16.51
3-6.c6256/1/3668.3	--	--	--	2.77	0.08	1.98	8.42	12.58	0	266.9	8.65	0	646.89	623.69	590.16
1-2.c33221/1/5086.	NADH dehydrogenase subun	164519190	YP_00164887	0	0	0	4.15	26.5	21.7	4.74	6.48	18.64	0	0	0
3-6.c15807/1/5101.	RecName: Full=Sarcoplasmi	9789732	Q92105.1	477.92	57.34	3500.07	200.11	1011.94	554.94	505.05	782.21	3065.79	0	3.76	0
2-3.c51054/4/2303	PREDICTED: uncharacterize	847110867	XP_01281479	2.72	0	3.7	3.04	2.12	4.74	1.45	0.59	2.22	0	0	0
2-3.c26860/4/2225	PREDICTED: ubiquitin carb	847100581	XP_01281262	0.86	0	2.96	3.1	3.51	3.39	1.73	3.93	0	0	0	0
1-2.c45091/1/1270	solute carrier family 3, memb	148229024	NP_00108024	0	5.93	5.99	18.95	17.36	3.3	0	0	34.97	0	0	0
1-2.c5151/6/1369	PREDICTED: potassium cha	545835340	XP_00565346	0.44	1.49	0.18	0.86	0.2	0.44	14.34	0.51	0.93	36.62	70.66	59.49
1-2.c25479/4/1349	ubiquinol-cytochrome c redu	148222361	NP_00108668	6.18	7.57	0	2.15	2.33	0	109.86	0	76.62	142.72	194.87	76.91
2-3.c60875/2/2495	PREDICTED: 14-3-3 protein	821027291	XP_01235685	112.34	94.82	95.58	46.88	77.05	149.6	56.59	87.7	81.21	0	0	7.12
1-2.c22412/1/1302	PREDICTED: actin, alpha ca	803091489	XP_00401102	9.76	0.67	37.32	16.7	26.42	20.26	13.34	28.86	13.58	0.75	0	0.75
1-2.c38472/4/1496	--	--	--	21.66	0	0	0	0	0	3.14	0	3.96	9.6	15.44	6.74
2-3.c14521/1/2006.	Protein CBG19037 [Caenorh	309356830	CAP36349.2	58.73	12.22	1.15	2.33	1.38	2.49	69.27	33.07	1.18	181.28	219.54	141.46
1-2.c50406/1/1224	PREDICTED: actin-related p	768340511	XP_01158074	0	0	0	0	0	0	43.08	0	17.55	184.11	285.62	29.31
1-2.c20761/1/1631	SEC13 homolog [Xenopus la	148233584	NP_00108044	0	7.42	0.17	0	0	0	1.6	0	2.03	16.74	3.4	12.74
1-2.c34576/1/1506.	--	--	--	22.97	1.71	0.39	45.97	20.78	3.01	650.37	21.03	10.69	2115.36	1301.43	1733.26
3-6.c16661/1/2853	PREDICTED: DNA-binding	620966761	XP_00765655	1.83	0.29	0.5	3.37	2.4	1.44	0	3.33	0	0	0	0
1-2.c18576/1/1747	PREDICTED: ubiquitin carb	301614077	XP_00293651	0	0	0	0	0	0	5.58	0	3.29	7.68	17.94	6.57
3-6.c6341/1/3171	extracellular sulfatase Sulf-2	54020831	NP_00100566	0	0	0	1.53	5.96	10.24	0	2.7	1.62	0	0	0
1-2.c14136/1/1746.	PREDICTED: glycerophosph	847157584	XP_01282458	0.67	1.52	0.28	0.55	0.63	2.7	13.91	0.21	1.03	89.93	110.44	163.35
2-3.c4864/3/2416	PREDICTED: ankyrin-1 isof	847110220	XP_01281460	18.13	0	71.15	40.35	66.45	28.19	26.38	17.34	28.7	0	0.83	4.58

1-2.c20903/1/1452	solute carrier family 37 (gluc	62201355	AAH93460.1	0	0	0	0	0	0	0.03	0	0.8	15.46	9.5	6.91
1-2.c25526/1/1355	Na-K-2Cl cotransporter 1 [Xe	147904042	NP_00109133	1.7	4.66	0.95	1.7	0.73	1.76	10.64	1.44	1.97	106.82	131.88	104.16
3-6.c2655/10/4009	PREDICTED: ATP-depender	301608355	XP_00293375	34.96	1.79	29.16	26.42	41.57	22.06	15.31	25.64	24.35	0.25	0	3.33
1-2.c47273/2/1538	--	--	--	11.84	78.63	0	0	0	0	10.35	0.04	65.08	81.91	45.07	13.51
1-2.c2114/44/1298	actin, alpha cardiac muscle 2	45361557	NP_989355.1	80.43	7.19	368.93	183.57	338.3	283.19	132.49	214	176	10.68	11.65	15.77
2-3.c54986/1/2343	PREDICTED: WW domain-c	558170054	XP_00612483	0	0	1.94	0	0	0	0	1.32	0	6.28	8.66	3.56
1-2.c36333/1/1697.	CD63 antigen [Rana catesbei	226372482	ACO51866.1	0	13.03	8.41	5.53	4.79	12.83	25.57	16.21	0	0	0	0
2-3.c7392/1/2451.1	PREDICTED: glycogen phos	512834332	XP_00293541	65.84	5.14	77.92	100.67	113.42	36.77	0	68.37	73.66	0	0	7.17
3-6.c13916/1/3100	--	--	--	2.47	2.37	1.58	2.96	6.2	3.9	0.28	0.78	0.87	0	0	0.2
1-2.c42850/5/1273	RecName: Full=Sarcoplasmic	9789732	Q92105.1	0	0	0	0	0	0	344.8	0	0	2313.67	4242.5	62.63
1-2.c50568/1/1318	solute carrier family 37 (gluc	62201355	AAH93460.1	0	2.11	0	0	0	0	0	0	0	8.29	10.84	15.87
1-2.c9613/42/1439	actin, alpha cardiac muscle 2	45361557	NP_989355.1	2.26	1.14	24.3	13.19	11.49	18.51	12.11	13.34	3.85	0.37	0	0.5
2-3.c51744/53/2162	regulator of G-protein signali	291290887	NP_00116747	0.65	2.04	0	0	0.38	0	3.49	0.38	0	23.09	13.52	20.76
1-2.c19020/1/1481	uncharacterized protein C9or	288684380	NP_00116577	0	0	0	0	0	0	0	0	0	12.17	14.18	6.47
1-2.c19564/1/7437.:	PREDICTED: myosin regula	736243225	XP_01078562	2.33	0	0	15.08	0	0	71.78	0	81.65	1017.87	1418.31	400.49
2-3.c61385/3/1953	tetranectin precursor [Xenopu	58332698	NP_00101142	10.81	2.44	19.23	19.4	15.67	2.62	0	9.07	8.98	0	0	0
2-3.c26687/1/2559	--	--	--	0	7.32	1.63	3.46	1.21	3.3	3.64	1.63	1.96	0	0	0
1-2.c12846/1/1900	PREDICTED: protein PFC07	556961274	XP_00599098	18.76	14.33	51.36	30.93	17.15	57.69	36.09	24.95	21.83	1.05	0.4	1.61
1-2.c6946/7/1253	fructose-1,6-bisphosphatase 2	291290927	NP_00116749	69.21	27.83	53.61	139.93	77.91	72.96	86.99	81.12	121.4	5.16	4.05	4.34
2-3.c34474/1/2219	--	--	--	0	4.71	0.08	2.91	1.99	4.32	6.32	2.95	1.83	0	0	0
3-6.c15068/1/3041	acetyl-CoA acetyltransferase	148234082	NP_00108602	1.67	16.18	19.21	13.24	13.49	15.02	0.79	0.15	10.94	0.77	0.51	0.35
1-2.c51338/3/1294	vigilin [Xenopus (Silurana) tr	55742112	NP_00100685	10.47	11.85	0.99	1.51	0.56	4.77	135.7	81.9	11.14	76.61	193.93	194.79
2-3.c33216/1/4581.:	PREDICTED: beta-enolase [	637381761	XP_00812393	2.18	0	5.07	10.95	9.1	2.19	13.46	1.91	14.54	0	0	0
1-2.c25028/1/5817.	PREDICTED: creatine kinase	852796670	XP_01288811	20869.62	2089.09	1.04	0.47	0	0.42	19933.25	0.24	0	7346.36	6179.93	0
3-6.c2358/17/3000.:	PREDICTED: telethonin [Xe	301622194	XP_00294041	18	7.57	37.91	3.97	18.34	17.09	2.02	9.6	5.88	0.14	0.16	0
2-3.c16215/3/1978	PREDICTED: acetylcholines	512876899	XP_00491915	4.66	0.61	6.5	2.66	3.73	3.74	1.75	1.69	3.63	0	0	0
2-3.c15308/1/1962	PREDICTED: nicotinamide r	874470990	XP_01295408	6.58	7.97	3.72	2.8	4.17	5.56	0.11	5.2	12.04	0	0.09	0
1-2.c26248/1/1585	PREDICTED: protoporphyrin	512881338	XP_00294247	0	0	0	0	0	0	0	0	0	12.01	9.08	7.1
1-2.c44528/1/1874.	--	--	--	0.64	0	0	0	0	0	3.36	2.18	0	33.73	36.97	16.47
1-2.c18465/1/1411	PREDICTED: A-kinase anch	602626968	XP_00742043	3.68	11.65	0	2.36	6.88	5.95	0	0	2.92	0	0	0
2-3.c11746/1/2616	Citrate synthase, mitochondri	808865946	KKF16719.1	42.56	0	0	0	0	0	0	0	26.63	1.82	10.49	10.14
1-2.c43556/1/1538.	--	--	--	2	12.21	5.47	6.05	9.39	6.88	0	0	6.25	0	0	0
1-2.c44518/1/1720	--	--	--	2.21	0	0	0	0	0.55	1.25	0	0	29.56	18.43	18.38
1-2.c12626/2/1462	--	--	--	3.92	21.36	10.49	9.86	5.3	15.55	3.49	6.05	6.44	0	0	0.49
1-2.c1223/40/1327	PREDICTED: actin, alpha ca	803091489	XP_00401102	224.23	12.37	619.46	352.43	580.95	484.24	363.91	721.37	347.33	42.2	10.63	23.87
2-3.c44884/40/2239	PREDICTED: kelch-like prot	301610740	XP_00293489	76.14	12.51	82.4	37.8	95.58	107.87	30.33	38.81	69.39	2.51	3.6	6.02
2-3.c39915/1/2371.	PREDICTED: flotillin-2 isofo	847099593	XP_01281229	8.62	27.84	7.36	3.45	9.33	7.7	5.58	7.48	2.94	0	0	0.19
2-3.c43037/1/2288	--	--	--	17.61	16.61	13.98	8.31	7.24	13.84	7.29	6.26	7.99	0.62	0.31	0
2-3.c16891/1/2128	PREDICTED: uncharacterize	530592326	XP_00528979	0	0	0	0	0	0	0	0	0.9	3.53	7.33	7.79
1-2.c25522/1/1601	PREDICTED: myosin-4 [Xer	847156920	XP_01282446	0.49	1.67	1.62	0.72	0.46	0.29	4.36	1.14	0.39	28.4	29.41	70.25
2-3.c21951/2/2219	periostin precursor [Xenopus	163915007	NP_00110637	26.67	82.15	33.2	33.17	54.99	59.41	46.04	36.37	41.72	5.6	1.75	0.5
2-3.c41442/1/1951	RecName: Full=Sarcoplasmic	9789732	Q92105.1	1045.21	200.73	2940.85	1705.14	2139.5	1133.41	188.55	597.12	1852.88	0	0	250.74
1-2.c32640/1/1469	--	--	--	0	0	0	0	0	0	4.13	0	5.04	14.94	26.23	6.22
2-3.c19708/4/2538	PREDICTED: kelch-like prot	301610740	XP_00293489	1.83	0.39	1.81	1.23	2.37	3.92	0.98	0.61	1.28	0	0	0
1-2.c14510/2/1730	sestrin-1 [Xenopus (Silurana)	194018650	NP_00112341	0	0	0	0.16	0	0	0	0.66	0	22.92	35.43	9.06
1-2.c40780/1/4598.:	fructose-1,6-bisphosphatase 2	291290927	NP_00116749	13.49	1.02	21.83	16.06	11.31	14.44	4.68	10.76	12.4	0	0	0.43
1-2.c46255/1/1557	succinyl-CoA ligase [ADP-fo	45361311	NP_989233.1	0	0	0	0	0	0	0	0	0	12.55	6.5	6.75
2-3.c61326/1/2590.:	PREDICTED: ovostatin-like	512878734	XP_00491941	3.27	3.11	0	0	0	4.61	7.02	0.89	4.95	61.36	87.06	143.69
1-2.c40995/1/1534	PREDICTED: diacylglycerol	301610039	XP_00293457	3.28	0	0.2	0	0	0	0.19	0	2.75	11.66	9.15	8.87
2-3.c12088/1/2321.	PREDICTED: band 4.1-like p	847168900	XP_01280819	0	3.23	0	0	0	0	0	0	0	14.03	21.66	5.14
1-2.c7050/1/1519.1	PREDICTED: glucosylceram	557017449	XP_00600913	0	2.24	1.96	3	0	0.96	2.18	3.44	7.43	96.33	114.04	75.22
1-2.c19245/2/1322	leucine aminopeptidase 3 [Xe	148228748	NP_00108469	0	0	0	0	0	0	5.03	0	0.56	12.18	5.88	14.25
2-3.c40476/1/2204	PREDICTED: protein Red, p	706108693	XP_01020783	2.4	15.74	3.89	0.85	4.38	5.43	0.45	0	3.29	0	0	0
1-2.c51161/3/1577	RecName: Full=Nucleolar RN	765680610	Q9DF36.2	88.19	398.23	87.82	68.7	55.91	94	82	62.49	115.31	0	0	9.82
3-6.c15588/1/3291	PREDICTED: nebulin isoforı	847166495	XP_01282660	0.14	0	0	7.72	0.91	9.7	4.98	16.26	0	0	0	0
2-3.c23106/2/2177.	--	--	--	0	0.09	0	0	0	0	6.96	0	6.34	8.92	19.45	14.69
1-2.c25537/1/1315	PREDICTED: lysine (K)-spe	847155363	XP_01282414	0	7.03	1.83	0.39	0	0	2.2	0	3.31	18.57	18.85	27.24
2-3.c21101/1/2607	PREDICTED: ubiquitin carbo	514753716	XP_00502143	6.95	2.8	1.22	2.63	2.82	1.69	1.01	2.59	2.35	0	0	0

2-3.c27108/1/2172	ubiquitin-conjugating enzym	56118358	NP_00100795	0.71	0	0.92	0	0	0	0	0	0.86	2.71	17.39	14.31
1-2.c49494/1/1738	PREDICTED: protein phosph	512830294	XP_00491303	7.64	0.88	6.78	8.45	12.31	5.7	2.06	0	6.26	0.39	0.34	0
2-3.c53489/1/1896	PREDICTED: ras-related pro	612007529	XP_00136477	15.02	5.48	6.57	10.33	11.2	5.75	0	10.06	10.27	0.55	0.28	0
1-2.c23510/3/1343	PREDICTED: actin, cytoplas	724842428	XP_01036529	11.09	1.03	24.58	16.84	22.18	20.47	12.43	18.37	18.95	0.9	0.9	0.42
2-3.c56087/1/2037.	alpha-actinin-3 [Xenopus (Sil	213983245	NP_00113551	230.02	46.5	391.4	208.04	341.4	345.45	109.9	233.72	150.25	1.54	6.44	36.45
2-3.c14676/5/2209	solute carrier family 37 (gluc	147901932	NP_00108340	1.76	4.38	3.94	0.87	5.44	5.21	1.33	0.56	6.53	0	0	0
2-3.c17718/19/2251	DDX5 protein [Xenopus laev	52430509	AAH82849.1	287.85	391.34	148	189.68	203.46	180.66	380.6	255.28	211.53	35.3	0	0
1-2.c34020/1/1572	--	--	--	9.89	0	0	0	0	0	0	0	4.44	10.3	5.01	11.92
1-2.c20938/1/1363	uncharacterized protein LOC	166158248	NP_00110750	0	0.69	3.05	0	2.19	1.15	3.22	0	4.96	67.08	130.34	64.12
3-6.c14436/1/4046.	delta-1-pyrroline-5-carboxyla	156717284	NP_00109618	6.76	6	15.33	10.76	7.92	10.61	3.27	3.13	7.25	0.09	0.15	0.58
1-2.c40095/1/1613	pinin [Xenopus (Silurana) tro	54020926	NP_00100570	0	10.77	0.89	2.43	4.61	4.44	4.74	0	0	0	0	0
3-6.c7531/1/3246.1	von Willebrand factor A dom	148228482	NP_00107996	1.2	0	1.95	0.84	2.84	3.43	0	2.55	0.79	0	0	0
2-3.c27889/1/1831	hypothetical protein L798_08	646642601	KDQ71589.1	2.91	0	1.99	0	0	0	12.87	0.17	2.45	28.93	82.32	21.84
1-2.c45291/1/1938	--	--	--	0	1.85	0.79	5.88	2	3.88	0	1.32	0	0	0	0
2-3.c31997/3/2183	PREDICTED: ankyrin repeat	512839176	XP_00293509	14.31	0	6.88	8.74	14.47	2.23	5.36	13.59	17.25	0	0	0
1-2.c29105/1/1436.	--	--	--	15	7.38	3.69	0	0.94	1.58	21.13	0	11.37	63.41	114.56	66.3
1-2.c5549/1/1526.1	--	--	--	2.34	13.65	3.53	4.13	3.8	10.31	1.92	3.46	4.64	0	0	0
1-2.c52297/1/1366	RecName: Full=Transitional	257051069	P23787.3	0	0	0	0	0	0	18.37	0	0	300.76	329.7	0
2-3.c60084/1/4948.	aconitase 2, mitochondrial [X	147904130	NP_00108626	36.94	7.67	28.52	21.85	42.17	17.35	20.38	14.83	29.7	4.02	0	0
1-2.c51003/1/1895	leucine-rich repeat-containing	194332463	NP_00112373	6.59	9.51	4.86	9.27	10.94	11.48	0	5.45	0	0.28	0.87	0
2-3.c47658/1/2469	inner-ear cytokeratin [Rana ca	6980088	AAF34720.1	66.19	54.18	41.31	35.96	88.57	29.04	81.67	52.36	17.02	0	0	1.07
1-2.c25523/1/1409	HMG box-containing protein	147906895	NP_00108595	0	0	0	0	0	0	12.33	0.93	0	7.93	13.14	6.15
1-2.c22082/2/1301	PREDICTED: laminin subun	512844752	XP_00293635	0	2.57	0	0	0	0	7.71	0	0	15.61	12.46	3.18
1-2.c36348/1/4797.:	isocitrate dehydrogenase 2 (N	148227256	NP_00108032	489.78	319.59	648.73	579.65	499.07	427.53	291.97	446.8	429.66	26.35	18.17	43.68
1-2.c50902/15/1572	prosaposin (variant Gaucher c	148236249	NP_00108761	58.79	0	9.33	0	0	2.92	10.69	26.66	5.41	50.02	40.5	83.39
1-2.c14996/1/1493	potassium channel, inwardly	156717570	NP_00109632	1.8	0	1.4	0.27	0	0	0.69	1.66	0.02	18.16	26.93	7.94
1-2.c15349/36/1328	actin, alpha cardiac muscle 2	45361557	NP_989355.1	129.84	12.23	357.27	258.1	350.9	279.16	179.78	322.59	205.02	24.5	10.59	20.35
1-2.c17933/1/1363	MGC83940 protein [Xenopus	50925006	AAH79747.1	4.37	0	0	1.57	0	0	1.34	1.52	0.79	59.81	46.17	26.64
1-2.c40653/1/1299	periostin precursor [Xenopus	163915007	NP_00110637	20.83	56.09	85.83	27.84	53.2	60.69	22.56	14.88	14.67	2.21	0	4.48
2-3.c19079/7/2087	ARP2 actin-related protein 2	15778930	AAH14546.1	2.02	1.81	3.98	3.64	0.72	4.29	0.49	0.73	4.58	0	0	0
2-3.c24538/2/2210	--	--	--	0.2	2.02	2.85	0	0	0	0	0	2.93	5.07	7.59	4.62
2-3.c28684/2/2591	PREDICTED: nebulin isofor	847166510	XP_01282661	21.62	7.24	0.77	34.88	57.68	66.22	41.63	51.18	0	9.78	0	0
2-3.c14691/1/2173.	epiphycan precursor [Xenopu	54020763	NP_00100562	5.94	15.61	16.87	15.48	5.21	9.61	11.74	10.15	16.72	0	0	0
3-6.c17085/1/2740	RecName: Full=Voltage-dep	46576355	O57483.1	34.82	12.68	45.97	31.05	43.05	48.8	17.64	29.83	28.53	3.93	0.82	2.37
3-6.c17714/30/2969	eukaryotic translation initiati	148237458	NP_00108006	5.17	0	7.06	6.84	11.86	7.49	3.15	8.7	5.37	0	0	0.97
2-3.c62766/2/2114	LOC398139 protein [Xenopu	46329514	AAH68910.1	88.34	15.7	65.33	33.87	72.04	31.7	41.73	56.86	66.96	0	0.01	5.45
2-3.c6856/1/2093.1	PREDICTED: tectonic-3 [Xe	512883714	XP_00294175	0	0	0	0	0	0.08	1.42	0	0.83	12.46	15.18	11.47
1-2.c34997/6/1434	tubulin beta chain-like [Scler	820146195	KKX13732.1	0	0	0	0	0	0	5.58	0	0	44.89	12.35	16.6
3-6.c2744/8/3086	thrombospondin-4 precursor		118404528	NP_00107267	7.17	27.5	10.72	7.06	6	7.13	4.44	9.16	10.47	0	0.56	0.25
2-3.c1438/35/2389	ADAM metallopeptidase don	90968576	NP_00103510	12.37	0.27	9.9	12.23	17.55	16.21	0	5.49	0	0.08	2.48	0
3-6.c14981/1/4219	PREDICTED: A-kinase anch	512820714	XP_00293685	5.72	3.45	2.72	3	6.09	3.57	0.97	2.46	1.72	0.27	0	0.15
2-3.c29964/1/2289	--	--	--	562.56	450.35	510.7	782.64	515.88	279.98	912.63	506.28	521.42	37.89	27.35	17.64
2-3.c16223/1/1964	PREDICTED: nebulin isofor	847166506	XP_01282661	0.74	9.01	0	6.24	7.66	3.49	1.16	1.47	0.75	0	0	0.3
1-2.c30262/1/1207	PREDICTED: F-actin-cappin	525020623	XP_00505782	151.11	106.44	239.51	139.16	181.89	145.56	93.89	167.47	131.51	0	0	25.27
2-3.c20371/1/2460	--	--	--	0.79	2.44	0	0	0	0	0	0	1.32	2.84	4.57	6.39
2-3.c43363/1/2033	actin, alpha cardiac muscle 2	45361557	NP_989355.1	4682.12	983.69	8484.87	2690.54	5710.56	5954.48	4462.68	8821.38	4039.68	325.36	102.42	476.74
1-2.c38658/1/1657	PREDICTED: protein PFC07	556961274	XP_00599098	11.43	3.6	36.99	19.8	34.59	116.79	25.56	21.34	34.51	0	0	0
2-3.c11447/13/2042	cell division cycle 23 [Xenop	147903791	NP_00107989	2.49	7.72	1.95	0.76	3.39	4.61	0.54	1.39	0	0	0	0
1-2.c5599/1/1519	--	--	--	0	0	0	0	0	0	0	0	0	10.37	10.89	4.36
1-2.c10612/8/1750	uncharacterized protein LOC	148237928	NP_00109116	21.4	15.22	28.34	32.62	36.34	58.35	19.92	40.44	26.73	2.27	2.77	2.35
1-2.c43666/1/1083	myosin heavy chain, partial [	4249695	AAD13769.1	0	0	0	0	0	0	0	16.07	0	248.03	320.59	7.23
2-3.c24232/1/2578	PREDICTED: protein phosph	512830294	XP_00491303	6.51	1.37	7.37	7.08	9.68	5.37	3.24	7.26	6.91	0	0.51	0.35
2-3.c49637/1/2329	fibronectin precursor [Xenop	148237139	NP_00108127	7.11	50.32	2.69	2.15	2.9	4.72	14.95	2.26	3.3	147.82	171.57	175.82
1-2.c24795/2/1617	PREDICTED: alpha-actinin-:	558140029	XP_00611831	171.94	30.27	215.4	158.34	193.71	195.55	27.41	168.95	35.81	0	0	31.41
1-2.c40888/1/1636	LOC733250 protein [Xenopu	66910737	AAH97624.1	0	0	0	0	0	0.21	0	0	5.15	53.57	17.69	109.85
1-2.c36290/1/1435	PREDICTED: delta(3,5)-Delt	597740413	XP_00723236	0	0	0	0.03	0	0	4.06	0	0.75	8.62	13.03	8.52
2-3.c56525/1/1890	3-oxoacid CoA transferase 1	148222725	NP_00108324	17.87	46.43	33.29	25.72	20.26	50.23	24.97	20.78	16.56	2.19	1.67	1.5
2-3.c50653/1/2108	E3 ubiquitin-protein ligase RI	47575796	NP_00100124	0	0	0	0	0	0	0	0	0	27.59	40.39	4.4

1-2.c5848/1/1483	PREDICTED: protein transp(	847087089	XP_0128162(	0	0	0	0	0	0	4.12	0	0	7.37	6.05	10.77
3-6.c13049/2/2759	--	--	--	26.3	1.12	0	58.6	25.2	7.36	9.69	37.75	26.96	0	0	0
1-2.c37257/2/1361	--	--	--	7.84	0	0.14	0	2.5	1.13	0	0	4.16	83.56	71.02	68.4
3-6.c12894/25/3147	RecName: Full=DNA replica	108935850	P55861.2	2.35	36.54	2.02	1.57	4.52	8.07	0.2	1.86	1.71	0	0	0.1
2-3.c22409/1/2005	phosphatidylinositol 4-kinase	156717328	NP_0010962(	0	0	0	0	0	0	2.69	1.75	0	8.44	10.32	1.94
1-2.c2867/13/1632	PREDICTED: L-lactate dehy	675698937	XP_00275513	242.11	10.06	376.53	436.62	538.96	209.18	182.51	187.83	444.14	0.06	0	74.59
1-2.c20496/1/1589.	adult beta globin [Rhacophor	451957813	BAM84268.1	0	0	0.24	0.6	0.14	0.16	8.66	0.13	0	153.03	197.36	626.1
1-2.c13220/2/1481	solute carrier family 16 (mon	148232306	NP_0010803(	0	0.13	0	0	0	0	6.5	0	0	4.06	12.76	6.51
2-3.c55328/1/2089	PREDICTED: proteoglycan 4	734645154	XP_01075165	67.81	40.4	80.63	51.2	61.13	94.95	49.32	50.12	59.56	4.93	3.18	5.3
3-6.c4810/1/4152	PREDICTED: myb-binding p	512820645	XP_0049117(	2.5	4.26	1.49	1.78	2.03	4.45	1.07	1.34	1.09	0	0.18	0.04
1-2.c28288/1/1446	--	--	--	1.4	2.12	0.31	0	0.53	0.94	0	1.12	1.52	39.33	40.59	25.94
1-2.c32534/2/1543	PREDICTED: actin, cytoplas	826278329	XP_01249437	0.31	0.13	0	0	0	0	0.06	0	0	10.77	4.09	8.77
1-2.c45696/1/1693	LOC100135355 protein [Xen	163915685	AAI57657.1	0	0	0	0	0	0	4.27	0	2.64	9.62	6.88	3.5
1-2.c7021/1/1558	secretory carrier membrane p	148225835	NP_00108753	0.01	0	0	0	0	0.02	4.01	0	1.38	3.66	10.87	8.03
1-2.c2075/20/1434	actin, alpha cardiac muscle 2	45361557	NP_989355.1	3.48	1.37	33.39	18.9	18.18	29.31	32.3	29.46	12.35	0	1.47	1.75
1-2.c24448/3/1348	--	--	--	4.03	4.18	0	4.81	7.59	1.99	9.1	0	0	0	0	0
2-3.c50059/1/2712	PREDICTED: myosin-4 [Xen	847156920	XP_0128244(	0	0	86	28.08	32.95	0.43	118.58	0	0	0	0	0
1-2.c16836/1/1318	--	--	--	3.27	4.63	0	0	0	2.36	8.61	1.27	3.76	48.37	62.99	32.38
1-2.c49017/1/1386	--	--	--	4.81	0	1.11	1.04	0	0	2.86	0	0	37.53	23.57	27.31
3-6.c2935/9/3663	PREDICTED: bifunctional m	768353287	XP_01157358	22.16	36.06	35.43	11.6	19.07	12.03	6.76	18.03	12.89	0.59	0.06	1.74
1-2.c51832/2/1365	legumain precursor [Xenopus	54020950	NP_00100572	0	0	0	0	0	0	0	0	0	181.34	203.8	0
1-2.c10620/16/1316	Unknown (protein for IMAG	62132943	AAH92152.1	5.35	12.09	4.29	4.75	6.05	2.95	1.47	1.08	0.68	0	0	0
1-2.c16997/1/2138.	PREDICTED: actin, alpha ca	803091489	XP_00401102	68.49	6.61	218.37	175.53	200	176.64	118.03	200.52	142.53	15.36	7.8	15.42
1-2.c12272/1/1850	PREDICTED: iron-sulfur clu	859911141	XP_00477417	0	6.08	6.73	5.53	7.38	4.2	0	0.05	3.03	0	0	0.33
3-6.c6143/1/2855	glycogen phosphorylase, brai	50054411	NP_0010019(	8.32	8.28	3.6	31.69	17.07	7.65	17.95	9.59	14.46	0	0	0.46
1-2.c49150/2/1774	PREDICTED: monocarboxyl	704293877	XP_01016065	6.87	0	13.63	29.58	31.45	10.12	4.27	6.92	0.17	0	4.7	0
2-3.c31481/105/266	Unknown (protein for IMAG	115528309	AAI24911.1	4.22	14.27	6.08	2.49	4.99	4.07	12.61	3.42	2.86	0.27	0	0
2-3.c36943/1/2136.	replication termination factor	148236615	NP_0010827(	36.06	35.33	19.78	21.93	21.56	7.98	15.38	17.37	17.81	0	0	1.84
2-3.c27148/2/2441	--	--	--	13.61	3.76	14.59	11.63	9.95	7.84	3.7	4.65	6.5	1.22	0.26	0.06
1-2.c34721/1/1320	PREDICTED: translocation p	847130489	XP_0128190(	6.06	0	4.67	1.93	4.29	6.82	0.89	0	5.26	0	0	0
1-2.c20802/1/1590.	--	--	--	0	0	0	0	0	0	0	0	0	17.53	23.67	25.68
2-3.c6984/2/2518	PREDICTED: leucine-rich re	847138470	XP_01282083	0	0.43	0	0	0	0	0	0	0	4.22	2.65	5.42
1-2.c26527/1/1806	PREDICTED: reticulon-4 iso	847127975	XP_01281854	41.66	43.93	125.12	82.02	100.98	162.77	82.76	96.87	109.54	7.52	2.32	13.45
1-2.c43459/1/1591	--	--	--	2.16	1.81	4.43	3.03	3.61	8.86	0.86	1.56	1.03	0	0	0.14
2-3.c32459/1/2613	--	--	--	5.45	28.25	4.81	6.66	4.95	10.31	0.52	1.46	4.02	0.14	0.32	0.43
2-3.c24500/2/2265	--	--	--	2.01	3.15	4.16	4.26	3.88	6.09	1.92	5.27	2.11	0.35	0	0
1-2.c4762/2/1399	26S proteasome non-ATPase	62751452	NP_00101581	0	4.64	0	0.19	0	0	2.01	0	0.51	54.26	9.22	37.65
1-2.c47809/96/1627	uncharacterized protein LOC	89886093	NP_988922.2	3.06	16.49	0.11	10.69	2.99	4.34	0	3.32	3.95	0.03	0	0
1-2.c33227/1/1253	PREDICTED: transcription i	847108696	XP_01281408	0	0	0	0	0	0	0	1.36	0	7.7	12.21	6.58
1-2.c13222/2/1615	PREDICTED: NAD kinase 2	847088841	XP_01282125	0.09	5.08	1.69	0.22	0	0	2.77	0.31	2.34	14.15	16.83	5
1-2.c5722/4/1362	protein disulfide isomerase fa	147906274	NP_00108664	0.78	0	4.96	1.45	0	0	13.68	0.5	3.87	49.02	41.37	15.86
2-3.c2231/9/2133	phospholemman precursor [X	58332490	NP_00101132	16.01	20.68	19.9	16.91	24.17	52.69	6.49	5.28	17.4	0.34	0.38	3.2
2-3.c36236/1/2607	trimeric intracellular cation cl	148237167	NP_00107953	124.65	224.65	299.88	214.65	234.96	559.51	214.96	228.21	254.04	25.09	9.49	30.98
1-2.c5196/1/1365	PREDICTED: DDB1- and CU	847086461	XP_01281478	1.01	1.17	0	4.14	0	0	15	6.36	14.58	70.93	92.56	65.57
1-2.c36367/1/1496	PREDICTED: lysyl oxidase h	704337203	XP_01017403	0	0	0	0	0	0	5.83	0.91	0	8.67	8.41	4.64
2-3.c45840/1/2051	--	--	--	0	23.27	0	4.18	11.7	13.54	10.09	6.55	10.6	0	1.61	0
2-3.c13368/1/1826	PREDICTED: tropomyosin a	573881544	XP_0066289(	0	30.98	0	0	0	0	0	0	19.56	96.58	91.75	0
1-2.c34979/1/10400	PREDICTED: LOW QUALIT	637377851	XP_00812323	0.65	0	0	0.32	0	0	0	0	0	1327.4	1837.21	0.35
1-2.c7620/1/1697.1	--	--	--	74.29	14.02	65.29	25.84	62.17	63.76	26.61	38.36	32.29	4.12	3.46	0.17
1-2.c46302/1/1517.	--	--	--	0.18	0	0.22	0.52	0.08	1.31	0.85	0	0.61	54	47.97	35.15
2-3.c45771/1/2261.	PREDICTED: U2 small nucl	301617734	XP_0029383(	0	0	0	0	0	0	0	0	0	6.22	8.54	1.83
2-3.c60918/1/2239	PREDICTED: myomesin-1 [	512850290	XP_00293895	12.42	8.13	40.17	25.11	28.33	52.86	19.53	26.34	30.4	1.61	0.99	4.16
1-2.c40211/1/4830.	glutamate oxaloacetate transa	333805525	BAK26556.1	105.15	7.26	114.34	118.58	134.34	44.61	29.6	86.01	95.78	0.89	0	17.03
1-2.c29762/1/1513	--	--	--	0	0	0	0	0	0	2.28	0	0	7.98	13.69	3.05
2-3.c21792/1/2302	PREDICTED: A-kinase anch	512820714	XP_00293685	9.93	8.72	4.99	9.02	8.16	12.53	2.26	0.64	10.77	0.73	0.48	0.38
1-2.c33636/2/1343	NADH dehydrogenase subun	164519190	YP_00164887	68.1	0	0	0	73.91	65.57	24.83	71.07	76.86	0	0	0
2-3.c52048/2/2020	mediator of RNA polymerase	62858965	NP_00101697	0	0	0	0	0	0	1.85	0	0	3.78	6.04	4.77
2-3.c52576/2/2232	myosin, heavy chain 4, skelet	47575800	NP_00100124	5.64	2.64	526.66	256.87	131.61	0	724.52	80.62	129.96	0	0	0

3-6.c5836/1/3573	PREDICTED: laminin subun	512830498	XP_00293314	0.11	6.64	0	1.93	1.07	0.78	1.46	0	2.91	0	0	0
1-2.c1273/14/1334	PREDICTED: actin, alpha ca	803091489	XP_00401102	4.42	0.32	19.59	14.59	8.34	14.52	10.76	16.03	7.55	0.37	0.15	0.84
2-3.c12113/1/2392	ATPase, Ca++ transporting, s	147907326	NP_00108644	0.24	4.35	0.62	0.76	2.34	2.82	0	0.07	0.25	0	0	0
3-6.c2494/7/2939	zygotic DNA replication licen	113931568	NP_00103923	0.48	15.1	1.15	0.42	2.49	3.38	0	0.36	0.24	0	0	0
1-2.c22107/2/1263	nuclear Y/CCAAT-box bindi	148233547	NP_00108380	3.1	0	0	0	0	0	1.2	0	3.39	7.12	7.21	9.21
2-3.c20973/1/2385	protein-arginine deiminase ty	156717898	NP_00109649	8.74	2.57	32.59	32.68	27.29	17.13	25.31	11.63	16.73	0	0	4.91
2-3.c38653/3/2392	eukaryotic elongation factor 2	187607479	NP_00112014	1.67	1.04	3.2	5.61	4.37	1.03	0.71	1.57	1.21	0.07	0	0
1-2.c35025/2/1409	PREDICTED: coatomer subu	327281271	XP_00322537	0	0	0	0	0	0	0	0	0	71.71	105.35	5.36
1-2.c4677/1/1258	ribose-phosphate pyrophosph	45361007	NP_989140.1	0.81	0	10.13	5.2	5.29	2.63	0	4.91	0.9	0	0	0
3-6.c12902/5/3256	nuclear factor 7, brain [Xenop	284009784	NP_00116500	2.91	14.26	2.1	2.57	8.27	4.49	2.5	2.12	0.38	0.44	0	0
2-3.c39996/1/2290	--	--	--	0.62	0	0	0	0	0	0	0	0	7.26	11.1	1.95
1-2.c21069/2/1660	MGC97489 protein [Xenopus	58477438	AAH90081.1	0	0	0	0.03	0	0	1.09	0	0.03	8.42	10.71	2.97
1-2.c11469/1/1569	--	--	--	0.98	0	0.18	1.63	0.7	4.14	17.87	1.26	4.47	216.95	277.83	73.8
1-2.c24105/1/1356	acyl-Coenzyme A dehydroge	89269093	CAJ81939.1	0	7.16	1.9	1.64	13.08	11.4	7.73	3.05	8.28	0	0	0
1-2.c35380/1/5809.:	PREDICTED: ADP/ATP tran	499026607	XP_00456404	0	0	0	0	0	0	0	0	0	147.78	262.28	18.5
1-2.c44753/2/1726	PREDICTED: myosin-bindin	556950430	XP_00598765	1.79	0.36	3.81	1.94	4.11	2.41	2.61	3.82	5.84	0	0	0
2-3.c46272/1/4506.:	ubiquitin carboxyl-terminal h	187608155	NP_00112016	0	0	0	0	0	0	0	0.87	0	4.02	3.7	5.12
2-3.c7988/1/2180	--	--	--	0	19.15	0	0	0	0	0.33	0	0	5.47	3.68	4.16
2-3.c58978/1/2396.	--	--	--	2.23	1.77	11.41	4.97	12.91	12.09	2.6	3.24	10.34	0.74	0.71	0.12
1-2.c20240/1/1590	sarcoplasmic/endoplasmic ret	383511148	AFH40438.1	1900.52	35.25	5805.04	2283.13	3091.6	1519.6	777.53	2118.78	4104.72	0	0	530.52
3-6.c14250/1/3002	PREDICTED: transmembran	847111920	XP_01281499	6.53	4.7	2.1	2.05	2.19	2.86	4.44	3.44	3.23	79.46	193.5	99.54
1-2.c19560/5/1393	periostin precursor [Xenopus	163915007	NP_00110637	7.65	175.81	49.7	20.49	33.83	63.8	0.11	9.48	20.27	7.9	0.43	0.07
1-2.c2170/8/1809	SET and MYND domain con	148225404	NP_00108546	66.26	38.35	64.62	51.6	19.27	47.07	42.27	87	38.98	1.9	4.53	2.7
3-6.c18948/48/2915	RecName: Full=Sarcoplasmic	9789732	Q92105.1	967.19	0.03	0	1083.23	1345.43	697.18	627.88	856.27	0	0	0	241.83
1-2.c2135/29/1515	coenzyme Q6 monooxygenas	148226512	NP_00108956	18.32	1.48	15.08	16.9	11.31	1.7	4.7	2.43	10.07	0	0	0
3-6.c10246/1/2750	DNA replication licensing fac	54020819	NP_00100565	0.78	10.16	0.82	1.32	2.01	4.4	0	0.74	0.97	0	0	0.08
1-2.c49169/2/1685.	--	--	--	0.03	1.17	0	0	0.04	0	1.79	0	1.24	8.5	11.05	8.81
3-6.c1140/24/3858	LIM domain-binding protein	45360839	NP_989095.1	9.96	15.45	57.77	39.87	52.37	81.81	49.73	27.18	47.85	2.66	0.93	9.64
1-2.c35370/1/1411	clathrin interactor 1 [Xenopus	147902057	NP_00108923	0	1.75	0	0	0	0.91	8.93	0	1.1	26.17	21.02	16.12
3-6.c10523/5/2996	acetyl-CoA acetyltransferase	148234082	NP_00108602	12.17	10.98	9.86	8.39	5.63	7.91	6.18	6.29	6.45	0.42	0.33	0.44
1-2.c17428/1/8059.	PREDICTED: serine/arginine	768366525	XP_01158055	0.43	0	36.33	0	0	0	0	0	0	107	124.26	0
2-3.c52057/2/1997	PREDICTED: isocitrate dehy	641774347	XP_00817074	0	0.18	0	0	0	0	4.06	0	2.1	8.8	1.15	10.57
2-3.c9054/6/2135	LOC398314 protein [Xenopu	80477501	AAI08429.1	37.53	29.79	45.13	34.12	30.23	30.31	10.65	26.55	15.03	3.63	0.87	2.67
3-6.c5374/1/2775	SH3 domain binding kinase 1	163914831	NP_00110662	0.11	0	3.21	1.6	1.27	2.14	0.5	1.86	1.54	0	0	0
1-2.c14149/3/1555	--	--	--	0	10.84	1.4	0.34	0	0.69	0.55	0.84	1.44	15.58	15.13	36.57
2-3.c22824/1/4823.:	aconitase 2, mitochondrial [X	147904130	NP_00108626	0	0	0	0	0	0	0	0	0	4.25	1.34	6.38
1-2.c38059/2/1384	PREDICTED: actin, alpha ca	803091489	XP_00401102	3.8	0	19.55	14.65	7.94	14.51	19.86	24.52	7.66	0.67	0	0.87
1-2.c23168/2/1377	PREDICTED: alpha-tectorin-	847131824	XP_01281925	1.14	0	1.26	0.1	0	0	0.85	1.27	0	15.1	11.08	5.07
1-2.c50543/1/1501	PREDICTED: myosin-10-like	847150223	XP_01282287	0.92	0.7	1.01	0	0	0	0.82	0	0.53	8.31	6.98	5.43
3-6.c16273/1/3924	RecName: Full=FACT comp	110287969	Q9W603.2	0.96	6.12	1.45	0.29	2.83	3.36	0.51	0	0	0	0	0
2-3.c55066/1/2457.	--	--	--	136.09	87.92	50.1	92.16	34.78	75.3	15.26	76.94	77.46	2.26	4.07	6.75
2-3.c22637/3/2209.	PREDICTED: monocarboxyl	513191317	XP_00494220	2.97	3.38	1.32	3.86	4.46	0.72	25.08	3.73	7.68	88.21	125.06	245.05
2-3.c61992/8/2407	PREDICTED: uncharacterize	672020667	XP_00877445	0.14	0	0	0.13	0	0	0.03	0.03	0	9.01	5.59	5.58
1-2.c52285/12/1164	PREDICTED: myosin-4 [Xen	847156920	XP_01282446	0	0	0	0	0	0	0.18	0	0	551.38	298.47	0
3-6.c18726/24/3600	PREDICTED: myosin-4 [Xen	847156920	XP_01282446	4.88	1.08	105.9	56.61	30.75	0.81	137.65	75.04	115.57	0	0	0
1-2.c23145/3/1629	--	--	--	6.4	0	2.49	0	0	0.81	0	4.54	0	39.21	26.12	10.15
2-3.c21019/1/2275.	syf2 protein [Xenopus (Silura	56268984	AAH87564.1	21.05	17.44	16.07	9.49	26.31	8.27	3.79	5.52	0	0	0	0
1-2.c33457/1/4870.	translation elongation factor I	211907091	ACJ12080.1	0	0	0	0	0	7.38	136.92	20.73	0	404.41	431.83	92.63
1-2.c42801/180/182	PREDICTED: myosin-bindin	556950430	XP_00598765	369.09	164.4	786.7	493.57	537.45	400.02	586.11	733.12	646.74	32.07	21.9	72.31
1-2.c7390/2/1355	PREDICTED: protein Shroom	847100721	XP_01281266	3.07	6.84	2.93	1.38	2.29	3.5	1.57	3.35	2.85	93.66	117.34	90.64
3-6.c14081/1/3349	histone-lysine N-methyltrans	148229543	NP_00108865	1.16	3.92	1.11	0	0	0	0.11	0.52	0	2.08	2.12	3.78
1-2.c11073/1/1327	PREDICTED: UDP-glucuron	743726910	XP_01095746	0	0	0	0	0.51	0	5.47	2.17	0	19.19	17.68	16.88
2-3.c29088/1/2064	uncharacterized protein LOC	147906100	NP_00108999	1.81	0.05	0	0.06	0	0	1.92	0	0.6	6.74	4.83	6.01
1-2.c30883/1/1351	hydroxysteroid dehydrogenas	56711282	NP_00100867	0	0	0	0	0	0	0	0	0	16.74	20.11	2.44
2-3.c4597/1/2009	PREDICTED: arrestin domai	847088847	XP_01282126	0.69	0	0	1.05	0	0.22	0.33	0.19	0	19.47	30.62	21.76
1-2.c25297/2/1428	PREDICTED: G-rich sequen	847162878	XP_01282565	0	0	0	0	0	0	6.12	2.46	0	8.92	9.24	2.33
1-2.c23850/1/1482.	uncharacterized protein LOC	148232280	NP_00108860	0.65	16.95	3.26	2.73	8.93	7.18	0.73	3.53	3.89	0	0	0
1-2.c31228/2/1721	UBX domain-containing prot	54262234	NP_00100581	1.76	1.73	3	1.53	3.25	3.44	0	2.14	1.67	0	0	0

1-2.c51760/2/1460	guanine nucleotide binding pı	148231283	NP_00108051	0	0	0	0	0	0	0	0	0	26.54	47.37	5.7
1-2.c23631/1/1376	7-dehydrocholesterol reducta	90186582	ABD91556.1	0	0	0.17	6.51	0	0.64	51.36	4.12	3.62	129.68	96.96	80.98
1-2.c9083/1/1384.1	PREDICTED: rho GTPase-ac	512868524	XP_00491809	25.13	8.07	5.09	6.11	14.46	3.91	0	5.56	0.53	0	0	0
3-6.c16303/1/7767.	RAB21, member RAS oncog	148230174	NP_00108340	2	0	1.01	2.08	1.46	1.22	0.87	0	0	0	0	0
2-3.c4895/2/2370	PREDICTED: papilin isofor	512862153	XP_00491729	1.43	0	1.12	2.68	2.21	0.54	1.92	1.34	0	0	0	0
1-2.c24013/1/1493	PREDICTED: eukaryotic trar	530563903	XP_00527854	0	0	0	0	0	0	0	0	1.85	8.73	18.09	4.08
2-3.c30406/1/1911.	Unknown (protein for IMAG	62739300	AAH94077.1	3.36	0.11	0.48	2.42	0.31	1.31	4.12	1.24	1.03	83.26	28.51	85.49
3-6.c18210/1/3904	PREDICTED: nebulin isofor	847166512	XP_01282661	3.13	0.2	0	4.18	10.09	10.61	5.85	7.12	0	1.81	0	0
3-6.c14072/1/5319.	--	--	--	101.64	0	0	39.61	54.71	3.89	64.18	105.76	61.41	1107.26	1538.95	1171.14
2-3.c17464/1/2500.	serine hydroxymethyltransfer	148231095	NP_00108035	2.59	14.08	3.55	4.15	3.12	7.06	3.45	3.67	3.6	0	0	0.35
2-3.c36658/1/1949	splicing factor proline/glutam	189230206	NP_00112142	0	69.94	9.04	0	0	0	24.96	0	0	54.86	56.97	0
1-2.c51304/8/1443	PREDICTED: LOW QUALIT	488565982	XP_00447266	0	0	0	0	0	0	2.62	0	0	5.28	9.64	5.15
1-2.c12227/2/1532	--	--	--	112.43	22.51	0	18.93	5.47	13.24	55.74	73.63	79.43	499.16	544.55	339.54
3-6.c16902/1/3051	PREDICTED: LOW QUALIT	612009927	XP_00748849	28.85	28.73	85.12	65.38	60.85	122.6	58.75	57.33	69.59	7.31	2.6	11.28
3-6.c13434/1/2809	--	--	--	0	0	0	0	0	0	0	0	0	67	104.27	0.02
3-6.c13878/1/3837	PREDICTED: myozenin-2 is	847087249	XP_01281675	6.89	8.35	14.37	5.12	6.21	12.34	6.09	8.75	6.65	0.71	0.23	0.64
1-2.c44147/1/1608	zinc finger protein 207 [Xenc	89269088	CAJ81941.1	0	0	0	0	0	0.59	0.53	1.91	0	16.56	18.3	10.38
1-2.c44843/1/1397	PREDICTED: nebulin isofor	847166508	XP_01282661	0	0	0	0	0	0	0	0	12.54	14.19	81.15	32.28
2-3.c41773/1/2153	ubiquitin thioesterase zranb1-	148225652	NP_00108846	0.05	0	0.01	0.31	0	0	21.32	0	0	34.3	43.88	7.46
1-2.c46353/1/1303	PREDICTED: adiponectin re	847112473	XP_01281509	5.67	0.8	0	2.82	0.35	0.78	21.82	2.66	0.63	51.7	93.91	41.68
2-3.c36739/1/2313	cellular retinoic acid-binding	25453404	NP_058940.1	0	0	0	0	0	0	0	0	0	46.69	41.49	0
2-3.c5261/1/2460.1	DnaJ (Hsp40) homolog, subfa	148233978	NP_00108036	0	0	0	3.51	0	2.19	31.66	9.15	13.96	103.05	106.26	51.11
1-2.c7311/11/1388	actin, alpha cardiac muscle 2	45361557	NP_989355.1	4.14	0.9	10.19	9.41	7.69	16.31	5.45	11.17	6.21	0.57	0	0.8
2-3.c32669/1/2125.	PREDICTED: splicing factor	634894020	XP_00795547	0.47	253.18	1.31	60.66	117.2	143.97	0.69	57.23	115.07	18.93	9.36	0
1-2.c17092/1/1629	DDX5 protein [Xenopus laev	52430509	AAH82849.1	10.97	75.38	5.55	11.34	0	14.93	44.87	8.31	22.68	316.56	368.36	231.34
1-2.c24737/2/1349	sorting nexin 9 [Xenopus laev	148237966	NP_00108661	3.48	10.36	2.54	0.39	0	0	3.77	0	0	20.31	10.08	14.03
1-2.c46289/1/1350	PREDICTED: phospholipid-t	395511539	XP_00376001	4.94	3.64	3.14	3.79	3.27	3.7	34.41	3.69	4.36	123.08	136.77	149.03
2-3.c33837/1/2408	Dupd1 protein [Xenopus laev	68534444	AAH99351.1	1.45	0.15	4.79	2.79	3.13	9.92	3.15	1.78	3.35	0	0.15	0
3-6.c8938/2/2775	autocrine motility factor recep	147901912	NP_00108315	0	0	0	2.3	0.63	1.59	1.74	2.24	0	0	0	0
1-2.c1844/104/1420	PREDICTED: ADP/ATP trar	829867054	XP_01264039	1835.04	401.95	1903.37	1973.19	1833.97	1590.49	1441.35	1401.85	1893.21	125.49	317.19	130.34
1-2.c7420/3/1485.1	intelectin 2 precursor [Xenop	351721678	NP_00116498	1.38	0	0.43	0.34	0.97	0.2	0.21	0.24	0.2	127.35	23.3	165.85
2-3.c1278/65/2199	PREDICTED: serine/arginine	530607466	XP_00529541	15.19	0.34	3.85	5.79	15	23.35	6.96	5.78	7.04	2.66	0	0
2-3.c25273/52/2227	serine hydroxymethyltransfer	148231095	NP_00108035	3.36	0	3.71	3.64	7.37	11.72	3.05	8.72	1.92	0	1.16	0.22
1-2.c47646/1/1594	guanine nucleotide binding pı	148231283	NP_00108051	0	0	0	0.1	0	0	9.93	0.28	7.85	6.02	30.08	16.51
2-3.c47478/1/2001	mitogen-activated protein kin	54262236	NP_00100582	0.28	0	0.21	0	0	0	0	0	0.41	4.92	8.53	1.78
1-2.c52147/2/1548	PREDICTED: ST3 beta-galac	847167879	XP_01282702	0	10.31	0	0.78	3.17	5.07	0	1.27	2.78	0	0	0
2-3.c59492/1/2064	--	--	--	0.83	8.99	1.38	1.76	2.63	1.72	0	1.16	1.28	0	0	0
2-3.c3501/1/2441	ethanolamine-phosphate phos	147905107	NP_00108663	8.1	0	1.69	3.06	5.56	10.74	10.47	16.62	5.9	119.81	210.31	311.37
1-2.c20623/3/1365	saccharopine dehydrogenase	147899649	NP_00108594	0	0	0	0	0	0	1.83	0	0	12.48	24.1	4.38
2-3.c56973/1/2232	PREDICTED: 5-oxoprolinase	847172228	XP_01280937	2.49	13.64	2.85	1.15	1.83	2.49	0.61	1.31	1.9	0	0	0
2-3.c10637/1/2295	myosin, light chain 1, alkali,	374533792	AEZ53809.1	455.17	165.28	1030.03	547.16	781.49	974.36	608.66	833.24	556.16	89.12	39.49	95.9
1-2.c7941/2/1535.1	PREDICTED: osteomodulin	847121387	XP_01281725	53.92	49.68	39.38	27.55	30.17	45.27	7.53	31.19	27.09	0.3	1.07	2.59
1-2.c30223/1/1534	--	--	--	1.32	2.51	0.51	0.57	0	1.8	7.56	0	0	35.55	33.12	36.26
1-2.c52406/2/1301	Na/K ATPase alpha	226444		0	0	0.36	0	0	0	39.11	11.16	0	0	118.91	213.25
1-2.c35113/1/6961.	NADH dehydrogenase subun	146149047	YP_00116545	45.86	0.03	18.35	0.63	91.79	116.48	91.42	113.83	125.71	0	0	0
2-3.c26202/1/2140	PREDICTED: immunoglobul	847130950	XP_01281911	0.82	4.14	0.67	2.13	2.3	3.97	0.17	0.22	0.55	0	0.13	0
1-2.c27318/2/1406	PREDICTED: decaprenyl-dip	620962451	XP_00766594	0	0	0	0	0	0.44	0	0	1.67	15.04	17.12	9.08
1-2.c24649/1/1289	PREDICTED: keratin, type II	698421871	XP_00981374	10.48	6.12	9.1	6.57	18.45	6.61	18.76	11.54	6.72	0	0	0.35
2-3.c51296/3/2313	RecName: Full=Voltage-depe	46576355	O57483.1	45.2	17.99	75.92	55.56	43.31	38.22	33.21	34.66	35.78	0	4.22	8.25
3-6.c16050/1/4660	PREDICTED: laminin subun	512844752	XP_00293635	4.91	3.02	4.69	2.61	4.8	7.45	3.76	4.77	4.08	0.43	0.33	0.27
1-2.c17351/1/1162	isoleucine--tRNA ligase, cytc	148231881	NP_00109069	0	24.37	0	0.18	0	0	18.63	0	9.44	25.84	42.47	8.16
1-2.c17315/1/1269	RecName: Full=Collagen alp	18202034	O42350.1	23.84	218.92	36.12	0.44	31.94	28.41	0	13.39	0	0	0	0
1-2.c48795/1/1645	PREDICTED: MAP/microtul	301620837	XP_00293977	0	0	0	0	0	0	0	0	0	7.24	5.12	3.45
1-2.c29225/1/1747	GDP dissociation inhibitor 2	148234617	NP_00108023	9.74	16.79	13.1	12.86	14.76	12.43	3.41	11.3	6.19	0	0.37	2.15
1-2.c33619/1/1343.	methylglutaconyl-CoA hydra	186910214	NP_00111953	0	0	0	0	0	0	0	0	0.5	11.25	11.42	3.09
2-3.c59239/1/1994	PREDICTED: alpha-actinin-	831481761	XP_01272875	370.31	116.96	562.85	423.11	470.45	447.72	291.87	459.29	372.16	24.79	0	99.73
1-2.c19811/1/1674	PREDICTED: isocitrate dehy	327270553	XP_00322005	0	0	0.01	0.01	0	0	8.26	3.63	0	3.94	4.43	7.03
1-2.c44433/1/1381	PREDICTED: DNA polymer	591360944	XP_00705539	0.18	0	1.02	0	0	0.04	0.81	0.39	0	9.07	5.04	9.32

1-2.c1532/75/1801	PREDICTED: eukaryotic init	847126504	XP_01281807	34.52	9.38	33.23	53.56	65.5	51.69	3.85	47.83	19.73	10.26	5.83	1.48
2-3.c30862/1/2333	uncharacterized protein LOC	148225160	NP_00108922	1.08	56.82	0.58	0.92	10.14	18.85	2.09	1.71	1.14	0	0	0
1-2.c5319/4/1207	PREDICTED: peptidyl-proly	742150246	XP_01088046	117.9	63.06	245.12	216.02	195.52	188.9	266.71	221.24	247.29	22.02	9.73	27.35
1-2.c42982/1/1688	PREDICTED: protein PFC07	556961274	XP_00599098	0.66	0.59	2.62	2.71	2.85	7.34	2.03	1.72	3.37	0	0	0.24
2-3.c27324/2/1999	SET and MYND domain con	148225404	NP_00108546	70.92	25.91	44.1	31.87	20.53	53.24	27.93	35.74	19.53	1.48	5.26	3.15
2-3.c62186/38/2430	methylcrotonoyl-CoA carbox	148229681	NP_00108606	8.54	9.21	19.87	10.11	7.1	4.04	6.81	10.03	15.05	0	0	1.15
1-2.c43372/1/1352	--	--	--	12.24	10.32	25.32	15.72	16.08	49.15	24.7	12.99	19.01	0.83	0.33	0.74
3-6.c17527/3/3163	LOC398447 protein [Xenopu	117558475	AAI25999.1	38.25	34.77	9.38	10.88	15.87	21.09	10.41	18.2	22.06	0.76	1.67	1.82
3-6.c18438/1/2623	aquaporin [Hyla japonica]	148356713	BAF63030.1	8.48	3.98	5.44	12.78	16.8	0	0	8.21	0	0	0	0
1-2.c39117/1/1720.	ribose-phosphate pyrophosph	45361007	NP_989140.1	7.15	9.74	6.63	7.19	13.74	6.81	4.71	8.99	6.98	0	0	0.69
1-2.c42327/5/1700	PREDICTED: myosin-bindin	556950430	XP_00598765	116.77	52.78	237.64	151.64	168.68	148.35	217.46	216.5	234.4	16.4	4.91	24.92
2-3.c22212/2/1898	PREDICTED: transmembran	602626865	XP_00742038	0	0	0	0	0	0	0	0	0	13.3	8.87	1.53
2-3.c41875/1/2166	calpain-8 [Xenopus (Silurana	113931326	NP_00103910	0.09	0	18.97	13.05	7.17	4.2	20.6	8.89	18.38	0	0.5	0.6
2-3.c21187/1/2248.	--	--	--	5.22	6.41	6.38	11.18	7.16	4.2	1.73	4.11	6.35	0.4	0	0.26
2-3.c35639/1/2168.	--	--	--	8.31	1.15	5.29	7.41	2.66	3.25	63.4	6.37	6.6	122.66	300.43	159.27
1-2.c11056/3/1935	hedgehog acyltransferase-like	147901798	NP_00107949	113.08	17.71	160.72	124.51	147.3	62.29	40.53	115.47	100.17	15.43	2.75	15.91
2-3.c34672/1/1940	--	--	--	0	9.79	0.01	2.12	1.77	2.46	0	0	0.16	0	0	0
2-3.c41116/1/2724.	--	--	--	2.33	5.37	4.58	7.99	9.95	12.56	17.24	10.59	14.95	333.92	447.43	338.54
3-6.c18077/1/2822.	--	--	--	311.02	9.43	8.49	38.99	22.82	2.33	228.17	189.66	44.35	631.68	1022.68	602.94
1-2.c46632/1/1305	actin, alpha skeletal muscle B	820145793	KKX13565.1	35.87	3.13	83.05	68.06	87.2	67.85	52.32	82.43	54.98	9.08	6.01	7.15
1-2.c11629/2/1644	Unknown (protein for IMAG	83405227	AAI10974.1	0	0.24	0	0.4	0.2	0	1.12	1.45	3.16	7.43	24.05	14.55
3-6.c15317/1/3178	PREDICTED: anoctamin-8 is	512814789	XP_00491107	2.01	0	1.15	1.51	1.57	0.52	0.9	0.57	1.1	0	0	0
1-2.c44724/3/1472	PREDICTED: interferon regu	847110323	XP_01281464	2.34	0.15	0	0	0.31	0	1.31	0	1.31	11.12	15.25	9.84
1-2.c19112/1/1538	E3 ubiquitin-protein ligase H	148231526	NP_00108707	0	0.27	0	0.44	0	0	0	0.82	0	6.21	16.41	16.97
1-2.c33449/3/1288	--	--	--	28.53	11.74	0.36	20.64	12.22	2.95	67.15	10.37	17.46	322.38	532.57	366.11
2-3.c40338/1/2041.	--	--	--	4.96	0.98	1.13	0.84	2.1	4.41	0.72	1.46	1.12	115.07	34.45	121.31
2-3.c41730/2/2436	--	--	--	31.02	16.59	41.22	29.4	25.77	43.58	24.84	15.67	48.01	1.38	4.58	3.71
1-2.c34551/1/1681	ubiquitin carboxyl-terminal h	113931406	NP_00103915	0	0.78	0	0	0	0	0	0	0	3.91	4.84	5.72
1-2.c45335/1/1714	tripeptidyl-peptidase 1 precur	156717508	NP_00109629	0	0	0	0	0	0	0	0	0	46.25	54.12	0
2-3.c23821/3/1933.	PREDICTED: thioredoxin dc	560931211	XP_00619174	14.53	36.09	17.35	13.98	22.63	12.48	3.7	2.89	6.65	0	0	3.26
2-3.c51963/5/2232	PREDICTED: myosin-4 [Xe	847156920	XP_01282446	0.71	0.22	29.19	24.31	17.31	0	40.19	27.79	39.32	0	0	0
1-2.c34829/2/1267	glutamate dehydrogenase 1, r	58331978	NP_00101113	1.71	0	0	0	0	0	8.45	2.02	0	40.27	19.43	5.4
1-2.c46615/1/1365	PREDICTED: beta-catenin-li	847167940	XP_01282706	0	0.96	0	0.51	0	0	4.49	0	1.99	14.29	19.89	11.96
1-2.c30776/2/1321	PREDICTED: erythrocyte ba	852789601	XP_01288561	0	0	0	0	0	0	0	0	0	58.45	65.63	0
1-2.c15050/1/1600	--	--	--	15.59	2.3	4.39	37.16	16.32	4.76	563.7	11.73	7.49	1040.23	390.65	1610.83
2-3.c5840/3/2220	--	--	--	0.3	3.57	0	1.8	0.7	3.08	1.48	0.06	0	0	0.03	0
1-2.c17552/1/1703	calsequestrin 1 (fast-twitch, s	148231736	NP_00108022	31.42	6.95	58.2	35.1	64.79	93.81	36.15	50.15	49.61	8.22	3.02	7.54
2-3.c39729/1/2624	uncharacterized protein LOC	148235829	NP_00108315	13.39	14.57	22.23	16.04	26.59	13.46	6.84	10.78	10.85	0.6	0.56	3.64
3-6.c7216/1/3400	RecName: Full=Voltage-depe	46576355	O57483.1	46.79	16.62	77.13	35.06	63.97	73.05	36.25	55	53.74	4.65	2.04	10.22
2-3.c60601/1/2302	--	--	--	3	8.3	0.02	0.62	3.21	1.74	1.41	0	0	0	0	0
2-3.c50385/3/2248	peptidyl-prolyl cis-trans isom	194018596	NP_00112338	2.62	65.41	14.73	5.69	8.87	15.2	7.74	4.72	15.74	0.54	0.2	1.22
1-2.c17218/2/1428	transgelin [Xenopus (Silurana	71896187	NP_00102557	0	0	0.25	7.53	3.13	7.39	20.41	0	11.54	282.59	202.11	120.22
1-2.c30427/1/1363	unnamed protein product [On	642108707	CDQ72316.1	0	0	0	0	0	0	0	0	0	63.14	52.92	0
2-3.c9563/2/2022	--	--	--	32.84	5.46	24.92	23.01	37.05	26.98	24.18	9.42	50.24	1.52	4.92	2.44
1-2.c1762/36/1637	adenylosuccinate lyase [Xenc	148226240	NP_00108059	67.44	49.07	93.56	78.5	72.32	109.76	72.2	80.02	77.71	8.57	8.64	9.72
1-2.c16791/1/1686	PREDICTED: ubiquitin-conj	836697765	XP_01278931	0	0	0	0	0	0	1.69	0	0	13.75	18.17	1.67
1-2.c26968/12/1659	PREDICTED: GTPase Era, n	512829715	XP_00293812	3.36	12.17	3.74	2.7	1.39	7.14	1.15	0.4	1.04	0	0	0
1-2.c45527/1/1343	poly(U)-binding-splicing fact	187607191	NP_00112008	0	0	0	0	0	0	0	0	0.3	17.38	40.88	7.33
1-2.c48291/1/1860	inositol hexakisphosphate kin	62859747	NP_00101595	2.53	0	1.73	6.53	0	2.48	8.33	2.32	1.32	127.77	71.87	97.12
1-2.c23962/1/1312	tetraspanin-31-B [Xenopus la	147903899	NP_00107965	0	0	0	0	0	0	2.61	0	1.91	16.86	17.34	1.61
1-2.c48448/1/1307	--	--	--	15.74	0	0	4.25	0.35	0.78	22.16	0	2.77	90.97	77.61	42.92
1-2.c5909/1/1250	hydroxyacyl-CoA dehydroge	148238090	NP_00108561	0	0	0	0	0	0	11.88	0	0	82.99	124.58	0.36
2-3.c49787/1/2595	alpha 1 type I collagen [Rana	3242649	BAA29028.1	22.8	565.34	123.28	68.6	111.27	319.4	42.71	38.15	77.51	1.25	0	2.99
2-3.c62446/7/2068	nucleolar protein 5 [Xenopus	89269840	CAJ82550.1	18.48	122.49	25.52	17.86	21.98	42.23	16.28	20.49	14.33	2.13	0.76	4.26
2-3.c47531/1/2281.	pyridoxine-5'-phosphate oxid	187608780	NP_00112001	1.38	5.72	4.54	4.41	4.29	3.09	3.25	4.14	4.8	0	0	0
1-2.c27775/1/2584.	PREDICTED: NADH dehydr	733928458	XP_01072510	0	0	0	0	0	0	0	0.9	1.29	52.94	35.88	8.41
1-2.c34159/1/4011.	PREDICTED: LOW QUALIT	829886178	XP_01264411	6.93	15.28	4.35	16.32	3.57	16.75	8.56	2.69	33.73	465	458.69	242.9
1-2.c15226/1/1474	Rab geranylgeranyltransferas	147900123	NP_00108929	0	0	0	0	0	0	0	0	0	5.63	6.89	5.8

2-3.c12389/1/2282.	signal peptide peptidase-like	118404330	NP_00107287	0	0	0	0	0	0	0.76	0	0	5.08	6.27	4.94
3-6.c15198/1/2939.	PREDICTED: guanine deami	512813022	XP_00491082	1.5	0.53	0.25	0	0.55	1.21	0.2	0.3	0.54	26.68	9.82	34.95
1-2.c28081/1/1433	--	--	--	0	0	0	0	0	0	0	0	0	47.53	47.53	0
1-2.c25284/1/4147.	PREDICTED: malate dehydr	149640897	XP_00151125	47.31	29.66	82.35	90.44	79.87	69.69	45.34	77.88	74.18	0	12.14	10.82
1-2.c28892/2/1517	PREDICTED: CCR4-NOT tr	301610011	XP_00293457	0.26	4.66	2.35	1.05	2.83	4.41	0.04	1.43	0	0	0	0
1-2.c51307/8/1416	eukaryotic translation initiatio	62859649	NP_00101672	0	0	0	0	0	0	24.11	0	0	60.71	82.26	0
1-2.c5664/3/1423	PREDICTED: unconventiona	301612177	XP_0029355(	3.37	4.93	1.77	0.55	0.07	0.45	9.89	2.02	2.29	22.97	21.32	17.67
1-2.c6185/2/1375	aconitase 2, mitochondrial [X	147904130	NP_0010862(	0	0	0	0	0	0	0	0	0	49.98	47.58	0
2-3.c13222/1/2137	PREDICTED: aspartyl/aspara	562852296	XP_00615483	1.16	1	2.43	1.25	3.41	6.38	2.25	4.13	3.29	0	0	0.19
1-2.c13253/1/1772	eukaryotic translation initiatio	148232020	NP_00108075	1.95	22.24	2.92	2.92	2.45	1.26	0	4.73	4.79	0	0	0
2-3.c3161/2/2554	CCR4-NOT transcription com	148228213	NP_00109065	17.55	0	3.74	0	0	0	0	0	0	19.88	0.01	23.68
3-6.c14935/1/3721	vigilin [Xenopus (Silurana) tr	55742112	NP_00100685	15.52	17.6	12.81	0.08	11.79	8.85	3.73	3.29	8.32	0	0	0
1-2.c29411/1/1857.	PREDICTED: polypeptide N	847131447	XP_0128192(	1.74	3.2	4.38	4.47	10.77	6.29	3.29	3.3	7.43	0	0.61	0
1-2.c35015/1/1773	--	--	--	4.78	0.89	0	0	0	0	0.03	0	5.66	1.75	16.85	11.51
1-2.c3425/3/1531	platelet-derived growth factor	71896169	NP_00102558	107.68	77.32	96.92	75.19	50.54	52.93	41.79	83.12	59.48	1.71	5.27	10.82
1-2.c3288/2/1291	egl-9 family hypoxia-inducib	148227604	NP_00108656	1.01	19.01	0	6.97	9.18	3.99	0	0	1.81	0.73	0	0
1-2.c47331/7/1846	collagen alpha-1(I) chain prec	58332412	NP_0010110(	0	0	0	0	0	0	0	0	0	11.25	14.4	1.26
2-3.c2584/3/2313	RecName: Full=Sarcoplasmic	9789732	Q92105.1	0	0	0	0	0	0	0	0	0	127.92	270.65	0
1-2.c27341/2/1620	PREDICTED: nebulin isofor	847166510	XP_01282661	118.79	36.55	124.16	227.16	181.58	362.1	181.96	287.9	135.21	43.57	7.48	31.95
2-3.c30358/2/1875	PREDICTED: interleukin enl	847169995	XP_01280854	5.07	7.27	0.41	2.94	0	0	8.63	10.24	0	32.4	48.96	32.71
1-2.c43137/1/1301	--	--	--	0.37	1.01	0.31	1.44	0	0.23	3.59	2.37	0	20.24	28	34.38
2-3.c3092/2/2361	heterogeneous nuclear ribonu	187607268	NP_00112002	0.11	101.43	0.12	0.07	25.72	51.4	0.09	16.16	0	0	0	0
1-2.c25668/2/1209	PREDICTED: 14-3-3 protein	602663371	XP_00743731	0	0	0	0	0	0	0	0	0	110.14	72.47	0
2-3.c18905/1/2213	PREDICTED: probable palm	512842773	XP_0049146(	0.84	0.14	0.13	0	0	0	0	0.73	0	5.57	1.14	3.89
1-2.c12883/1/1722	PREDICTED: 26S proteason	301612173	XP_00293561	0	0	0	0	0	0	0	0	0	35.37	35.96	0
1-2.c14958/1/1888.	--	--	--	89.18	23.06	74.81	33.75	29.86	0.96	9.5	34.74	24.12	0	0	0
2-3.c12222/1/2125	--	--	--	0	0.31	0	0	0	0	0	0	0	29.41	15.84	33.29
2-3.c2272/9/2556	PREDICTED: fibronectin typ	512827549	XP_00293967	24.15	5.41	24.14	13.75	19.39	13.35	7.09	15.09	12.67	0.93	1.08	2.33
2-3.c62692/2/2582	nicotinamide nucleotide trans	148231869	NP_0010877(	0	0	0	0	0	0	0	0	4.88	3.56	0.87	4.11
2-3.c1126/152/2512	PREDICTED: NADH-ubiqui	641774292	XP_00817072	37.33	15.95	41.51	33.19	39.6	18.44	16.35	23.86	28.24	2.44	1.63	5.35
2-3.c3322/8/2422	calpain-8 [Xenopus (Silurana	113931326	NP_0010391(	10.58	2.68	16.17	15.9	11.55	4.76	15.65	20.77	15.52	1.04	0	1.21
2-3.c54123/1/2257	RecName: Full=Collagen alp	18202034	O42350.1	30.83	363.97	101.73	69.92	121.17	273.55	35.87	29.35	60.76	7.53	5.68	5.29
1-2.c49308/1/4547.	endoplasmic reticulum-Golgi	47575764	NP_00100122	0	2.52	0.48	0.24	0	0	0.57	0.51	0.87	10.22	7.46	9.7
1-2.c35824/1/1272	PREDICTED: carnosine synt	847171322	XP_0049192(	10.92	5.11	21.27	8.48	13.72	25.02	4.93	9.02	9.95	0.97	1.12	1.25
2-3.c39567/1/2263	PREDICTED: transmembran	847111917	XP_0029323(	1.54	0	2.72	2.01	0	0	4.98	1.51	3.91	52.35	177.13	78.09
2-3.c20013/1/2011.	gamma-glutamyl hydrolase p	71896119	NP_0010255(	1.81	10.85	0.39	0.76	3.31	1.15	19.73	0.02	3.37	33.98	71.14	84.69
1-2.c32911/1/1410	ectonucleotide pyrophosphata	147899125	NP_00108052	0	11.57	0.19	0.1	0	0.93	0	0.84	0	34.19	7.7	25.39
2-3.c55655/1/2379	--	--	--	1.24	5.85	0.66	1.22	1.8	4.73	1.35	0.33	1	0	0	0.11
2-3.c15089/4/2714.	PREDICTED: NADH-ubiqui	641774292	XP_00817072	54.02	20.66	41.65	45.25	70.85	43.27	30.6	44.16	39.38	3.95	6.35	7.85
3-6.c18561/1/4282.	--	--	--	0	0	0	0	0	0	3.19	0	0	2.74	2.53	2.62
3-6.c6077/1/3143	glycogen phosphorylase, brai	50054411	NP_0010019(	0	3.59	2.14	0	7.09	8.99	0	1.47	0	0	0	0
1-2.c16828/1/1361	--	--	--	0	0	0	0	0	0	0	0	0	43.46	43.11	0
1-2.c23294/2/1270	LOC100170598 protein [Xen	189442022	AAI67684.1	0.31	0	0	0.06	0	0.02	0	0.06	0.28	9.53	7.64	7.36
2-3.c56811/1/2357	--	--	--	11.02	5.69	15.23	9.33	10.84	10.54	5.46	7.78	7.75	0.59	0.61	1.25
2-3.c2496/19/2100	PREDICTED: LOW QUALI	664723035	XP_0085186(	42.66	67.36	37.08	45.17	39.16	67.73	20.25	44.31	73.04	7.5	2.89	5.96
2-3.c28307/1/2130	uncharacterized protein LOC	148229417	NP_0010797(	0	0	0	0	0	0	0.22	0	0.48	3.79	5.5	1.41
2-3.c48038/1/2059.	PREDICTED: keratin, type II	677974099	XP_0090720(	733.81	653.38	604.54	479.88	1303.8	430.2	1315.08	665.91	423.8	16.5	13.03	18.55
3-6.c16716/1/3515	PREDICTED: myomesin-2 is	512841520	XP_0029352(	9.42	9.39	39.51	38.39	34.51	52.21	24.13	31.37	28.9	4.03	1.22	7.84
1-2.c6489/1/1389.1	--	--	--	0	0	0	0	0	0.13	0	0	1.94	17.54	12.58	12.98
3-6.c7697/1/3092	--	--	--	45.77	3.08	27.38	78.93	112.55	91.26	81.31	83.08	24.54	17.43	3.35	4.25
1-2.c27046/34/1901	PREDICTED: heat shock cog	512854037	XP_00293757	0	2.25	0	0	0	0	0	0	0	28.33	27.16	0.04
1-2.c24896/1/1227	--	--	--	2.75	0	0	0.32	0	0	0	1.85	0.25	11.96	16.97	9.41
1-2.c41246/1/1258	Dual specificity protein phos	678214136	KFV81115.1	6.2	8.6	0.59	4.38	3.81	3.62	9.02	7.56	14.82	122.57	186.19	80.32
3-6.c18884/8/2992	WD repeat-containing proteir	187608294	NP_00112039	1.02	6.87	0.76	0.8	1.76	3.44	0.68	0.54	0.49	0.07	0	0.06
1-2.c29706/1/1472	PREDICTED: pyruvate dehy	530574466	XP_00528163	33.25	16.99	1.69	56.7	42.94	25.26	20.17	49.06	32.31	2.49	4.94	5.84
3-6.c9454/1/3466.1	PREDICTED: isocitrate dehy	831547357	XP_01272691	406.37	274.58	366.08	279.42	334.36	329.41	231.58	270.05	261.8	39.69	40.98	34.52
1-2.c32641/1/6799	uncharacterized protein LOC	148232024	NP_00108962	11.33	2.58	4.02	3.72	0.16	0	8.7	5.34	5.69	71.37	81.34	35.31
1-2.c36201/3/1487	WD repeat-containing proteir	55742525	NP_00100678	37.66	20.28	7.66	12.49	0	0	29.53	10.49	0	159.39	80.29	148.42

2-3.c48583/1/2047	--	--	--	5.38	0	3.37	0.43	4.13	0.84	0	4.21	0.84	121.48	121.21	34.43
1-2.c45192/1/1712	PREDICTED: aspartate--tRN	847163066	XP_01282572	22.94	70.55	38.42	53.64	75.1	66.83	36.58	44.2	31.36	5.72	5.02	10.74
3-6.c14747/1/3100	E3 ubiquitin-protein ligase M	183986655	NP_00111691	0	1.13	0	1.5	2.13	0.66	0	0.71	0	0	0	0
1-2.c14699/1/1481	--	--	--	7.61	21.49	0.2	0	0	0	5.62	0	13.86	0	60.71	48.4
1-2.c5071/1/4048.1	PREDICTED: LOW QUALIT	803125841	XP_00401312	1.74	0	0	0	0.88	0	13.66	0	0	557.07	587.32	0
3-6.c8486/1/3731	PREDICTED: ribosome-bind	847127305	XP_01281832	2.14	12.58	0.52	1.44	1.45	5.58	0	0	1.28	0	0	0.02
1-2.c39104/1/1823.	RNA demethylase ALKBH5	148231788	NP_0010857(	0	3	0	0	0	2.35	13.03	0	0	94.27	167.32	38.15
1-2.c30592/1/1322	ran-binding protein 9 [Xenop	113205900	NP_00103797	0	0	0	0	0	0	0	0	0.35	34.93	45.63	1.08
1-2.c25094/1/1776	PREDICTED: choline/ethanc	733921552	XP_01072232	8.34	9.99	5.61	2.62	4.4	8.99	2.85	4.02	0.64	0	0	0.59
2-3.c39332/2/1950	--	--	--	0.49	1.84	0	0	0.56	0	5.35	1.19	1.7	10.77	5.99	17.62
2-3.c52464/1/2417	proliferation-associated prote	56605876	NP_00100843	0.86	37.02	12.52	9.85	10.4	30.67	11.93	9.5	0.48	0.69	0.89	0.36
3-6.c16114/1/3063	RecName: Full=Voltage-depe	46576355	O57483.1	3.81	1.68	6.55	5.65	2.29	2.94	3.22	0	0.13	0.66	0	0
3-6.c4216/1/2939.1	PREDICTED: vegetative cell	635140510	XP_00801711	10.45	4.24	0	0.26	0	0.65	13.48	5.87	0.05	25.76	43.12	27.79
3-6.c16673/1/3890	leucine--tRNA ligase, cytopla	62751875	NP_00101583	3.07	11.08	4.47	3.07	4.51	11.41	1.44	2.71	2.43	0	0.13	0.44
1-2.c45202/1/5025.1	PREDICTED: actin, alpha ca	803091489	XP_00401102	2.44	0	4.64	31.43	4.59	3.35	764.83	24.26	54.07	2049.47	1717.13	391.9
1-2.c48452/1/1358	biglycan precursor [Xenopus	147905193	NP_00108626	0	0	0	0	0	0	0	0	0.1	7.02	9	1.72
2-3.c62753/24/2291	methylcrotonoyl-CoA carbox	148229681	NP_00108606	6.77	14.6	0	9.31	5.71	12.19	5.15	0	0	0.62	1.42	0.33
3-6.c12316/22/2964	Glycogen phosphorylase, mu	465954282	EMP25833.1	59.21	2.15	55.62	56.76	55.47	42.98	47	71.3	44.52	14.73	1.46	2.61
1-2.c51825/255/155	Myozenin-1-like [Xenopus la	148237556	NP_00107945	1135.51	81.32	740.29	1134.01	1405.33	1042.46	421.74	1171.67	510.6	68.73	88.53	265.08
1-2.c13505/1/1288.	eukaryotic translation initiatio	148235981	NP_00108766	7.88	70.73	22.68	1.86	17.83	16.69	0	0	5.15	0	0	0
1-2.c1750/59/1533	PREDICTED: complement c	512820694	XP_00293686	36.82	94.22	31.65	31.51	25.19	49.86	14.67	13.79	25.68	3.55	3	4.25
2-3.c27424/1/2285	LOC548400 protein, partial [	59861981	AAH90369.1	0.82	3.47	13.83	30.49	9.41	4.56	49.04	44.15	35.18	443.24	672.38	244.2
1-2.c18866/1/1362	PREDICTED: splicing factor	637335070	XP_00811537	0	14.07	0	9.95	8.11	19.62	0	6	15.71	0	3.07	0
2-3.c62947/2/2242	--	--	--	0	0	0	0	0	0	1.4	0	0.09	0	18.47	22.7
2-3.c37639/2/1925	Methionine-R-sulfoxide redu	431892020	ELK02467.1	0	0	0	0.03	0	0	0	0	0	5.45	3.54	4.87
2-3.c22034/1/2250	PREDICTED: serine/threonir	847153661	XP_01282361	0.65	6.68	2.11	2.2	4.08	8.63	2.26	1.51	0	0	0.82	0
1-2.c24555/1/1365	--	--	--	30.28	8.41	73.06	44.73	80.59	140.74	63.79	60.61	64.93	13.5	3.05	12.28
1-2.c11440/2/1195	polyadenylate-binding protei	52346016	NP_00100505	0	0	0	0	0	0	15.16	0	0	209.05	497.96	9.99
1-2.c14090/1/1749.	claudin-3 [Xenopus (Silurana	54020932	NP_0010057(	0.67	0.27	0.24	0	0.1	0.59	0	0	1.39	23.98	10.4	18.75
1-2.c10848/4/1593.	--	--	--	1.86	6.99	0.85	1.98	2.18	3.81	8.75	1.36	1.67	106.29	118.45	78.28
2-3.c26218/1/1927.	--	--	--	0	0	0	0	0	0	0	0	0	9.54	11.09	15.68
2-3.c57287/6/2316	eukaryotic translation initiatio	209969740	NP_00112964	0	0	0	0	0	0	0	0	0	20.71	37.25	1.93
3-6.c12193/54/2756	glutamate dehydrogenase 1, r	58331978	NP_00101113	14.92	37.11	18.76	21.88	36.56	40.4	3.08	16.34	29.79	4.58	3.83	3.21
1-2.c35476/1/1581	PREDICTED: abl interactor 2	573898164	XP_00663681	1.5	5.53	2.75	0.75	2.59	3.91	1.08	3.4	2.26	0	0	0
1-2.c40039/1/1185	PREDICTED: glycogen phos	556954754	XP_00598898	72.83	9.9	91.91	142.56	165.85	84.28	131.92	162.86	233.33	3.6	11.99	29.52
2-3.c1599/26/2537	PREDICTED: protein phosph	847087415	XP_01281729	0	3.22	19.98	1.23	21.36	16.33	6.92	0	2.86	0.15	0	0
3-6.c13626/1/5444.1	PREDICTED: long-chain fatt	512839272	XP_00491424	1.49	3.38	1.25	3.27	0	0.74	4.98	0.71	2.2	41.31	32.27	51.34
3-6.c7204/1/2919	putative elongator complex p	147901297	NP_00108994	0.75	0.73	0.4	0.69	1.28	1.69	0.17	0	0.56	0	0.02	0
1-2.c32693/1/1336	PREDICTED: matrix metallo	847165172	XP_01282626	4.6	0.23	0.15	1.04	2.86	0.4	5.18	5.36	7.42	26.22	75.21	57.1
1-2.c41740/2/1404	uncharacterized protein LOC	148229755	NP_0010899(	1.67	31.32	0.38	0.54	3.39	5.33	1.5	1.2	0	0	0	0
2-3.c3701/1/2119	--	--	--	10.76	14.21	8.88	9.15	8.9	10.02	3.03	5.36	14.79	1.27	0.68	0.41
1-2.c46545/1/1478	alcohol dehydrogenase 5 (cla	148223732	NP_00108642	55.8	137.39	50.61	32.57	36.17	80.15	30.1	37.78	31.26	5.55	5.05	5.46
2-3.c3003/15/2117	Ptcd3 protein [Xenopus laevi	62739333	AAH94135.1	17.27	21.72	9.78	8.23	9.1	15.28	9.34	8.09	5.68	0.65	0.75	1.26
2-3.c45942/1/2583	4-hydroxyphenylpyruvate dic	118404104	NP_00107237	2.43	7.89	6.39	0.39	2.2	1.73	12.21	1.45	3.79	0	0	0
2-3.c32752/1/2342	PREDICTED: protein FAM1	530638059	XP_00530584	0	0	1.63	1.98	0.67	1.88	1.22	2.15	1.45	0	0	0
1-2.c19028/3/1285	PREDICTED: rRNA 2'-O-me	558189237	XP_00612907	10.89	51.53	5.27	11.95	10.47	27.95	11.47	10.86	3.87	1.57	0.82	1.49
1-2.c8743/1/1793	--	--	--	2.07	16.88	1.26	6.06	2.37	1.71	31.26	3.6	5.53	90.62	131.61	86.16
2-3.c43800/1/2127	Mimitin, mitochondrial precu	226372810	ACO52030.1	2.09	1.37	1.2	1.86	1.14	2.55	0.98	1.39	2.16	0	0	0
2-3.c16585/3/2247	AFG3-like protein 2 [Xenopu	118404624	NP_00107275	0	0.33	0	0	0	0	1.1	0	0	5.86	4.21	0.73
2-3.c2027/12/2325	PREDICTED: polyadenylate-	847099772	XP_01281235	57.35	30.34	57.29	39.7	46.84	67.23	34.97	30.3	50.28	4.56	6.2	7.35
2-3.c38485/5/2578	glycogen phosphorylase, brai	50054411	NP_0010019(	1.83	2.53	2.4	6.81	12.58	12.66	9.83	7.87	12.66	3.17	0	0
1-2.c10170/170/174	PREDICTED: alpha-enolase	675426353	XP_00892698	287.86	151.21	584.03	300.1	394.54	685.24	287.96	404.53	381.12	60.07	25.88	79.15
2-3.c34079/1/2556.1	ATP synthase subunit alpha,	71896075	NP_00102561	0	138.01	314.39	258.66	339.92	168.35	147.92	249.66	239.3	22.2	0	64.22
3-6.c2179/22/2953	PREDICTED: E3 ubiquitin-p	512842584	XP_00294018	0.34	11.6	0.68	0.55	1.03	2.8	0.13	0	0.38	0	0	0
1-2.c5552/3/1378	syntenin-1 [Xenopus (Siluran	55742362	NP_0010068(	7.01	8.06	1.35	0.2	1.88	1.79	7.35	0	3.64	56.49	68.03	20.39
1-2.c11988/1/1846.	uncharacterized protein LOC	147905311	NP_0010906(	0	0.87	0.71	3.7	0.3	5.05	0.63	6.43	1.26	112.21	134.89	82.7
1-2.c13540/6/1504	gamma-sarcoglycan [Xenopu	118404980	NP_0010729(	23.61	21.96	60.83	81.02	65.47	91.45	25.15	25.36	81.21	6.69	13.96	8.9
1-2.c9273/1/1520	--	--	--	5.48	8.39	1.18	3.3	0.85	5.18	9.43	4.63	4.84	88.98	90.91	89.19

3-6.c6870/1/3083	cell division cycle 25A protei	44895334	AAS48918.1	0.37	13.33	0.37	0.29	1.28	2.42	0.03	0.99	1.13	0	0	0
1-2.c45833/1/1581	PREDICTED: uncharacterize	512850405	XP_00293835	0.9	0.08	4.14	2.42	4.27	7.89	2.11	4.62	4.68	0.11	0.3	0
2-3.c46984/1/2028	--	--	--	0.06	12.2	5.49	2.16	0.36	3.38	3.12	2.06	1.43	0	0	0
3-6.c2902/5/3423	PREDICTED: catenin delta-1	847116240	XP_01281585	0.67	23.27	1.18	0	5.18	5.47	0	2.9	0	0	0	0
1-2.c38869/1/8486.	PREDICTED: heterogeneous	874483668	XP_01295842	63.42	274.72	55.86	47.9	66.75	86.56	25.92	56.54	22.84	0	0	17.19
1-2.c1381/110/1606	PREDICTED: aspartate amin	532002698	XP_0053456(	128.7	171.24	147.44	449.49	429.88	227.72	425.38	330.5	486.66	26.58	95.56	35.23
3-6.c16855/1/3259	periostin precursor [Xenopus	163915007	NP_00110637	3.43	15.16	10.02	7.69	7.71	9.88	6.13	6.93	7.06	1.08	0.87	0.62
1-2.c50069/1/1843	E3 ubiquitin-protein ligase ar	148228271	NP_00108982	0	0	0	0	0	0	4.38	0.7	0	2.15	7.32	2.84
3-6.c13732/1/2958	--	--	--	60.48	25.08	112.43	105.34	94.81	113.88	136.74	109.85	129.8	13.3	3.79	20.74
3-6.c3534/2/2899	PREDICTED: LOW QUALIT	512830904	XP_00293188	3.24	8.3	2.77	1.66	2.06	2.77	1.94	0	1.5	0	0	0.17
2-3.c1458/32/2465	PREDICTED: olfactomedin 4	847154698	XP_01282391	7.41	0.96	3.54	6.73	3.44	0.5	199.99	4.94	1.59	142.14	93.96	74.92
3-6.c9447/1/6228	PREDICTED: E3 ubiquitin-p	512821131	XP_00293523	0	0	0.23	1.17	1.45	0.75	0	0	0.24	0	0	0
2-3.c15817/1/2018	profilin-2 [Xenopus (Silurana	62859423	NP_00101609	4.39	9.02	8.2	4.46	7.61	8.98	2.77	6.26	6.44	0	0.2	1.16
1-2.c19093/2/1378	--	--	--	10.3	25.58	0	0	0	0	0	0	23.21	32.02	34.12	0
1-2.c17896/1/1525	--	--	--	0	0.13	0	0.04	0	0	0.97	0	0.74	7.07	7.44	3.35
1-2.c23241/1/1501	PREDICTED: protein ATP1I	301604441	XP_00293184	1.56	0.41	3.94	1.08	2.81	6.84	0.89	1.46	1.02	0	0	0
2-3.c14847/2/2642	DEAD (Asp-Glu-Ala-Asp) bo	148235819	NP_00108945	3.38	21.76	2.45	2.52	4.41	9.81	1.9	1.1	1.45	0.17	0.19	0.38
2-3.c39396/2/2763	transmembrane protein 182 p	118404178	NP_00107240	2.37	3.1	3.1	1.71	0.98	5.25	0	0	1.53	0	0	0
2-3.c21865/1/2082.:	PREDICTED: LIM domain-b	847142884	XP_01282173	24.53	19.16	159.73	72.58	133.89	210.01	61.79	60.04	121.24	7.16	3.35	32.14
3-6.c17854/23/3023	PREDICTED: choline transp	530641746	XP_0053076(	0	3.34	1.99	1.19	1.87	4.01	0	0.63	0	0.17	0.12	0
3-6.c3240/2/3594	Zinc finger protein 330 [Fukc	676271522	KFO26545.1	4.11	9.45	4.33	4.24	6.33	8.8	2.81	2.25	3.2	0.36	0.97	0.51
3-6.c6443/1/3207	PREDICTED: RNA-binding	847153751	XP_01282364	0	0	10.01	0	0	0	0	0	0	14.53	19.38	0
1-2.c2040/12/1672	tripartite motif-containing pro	147906184	NP_00107992	60.4	47.04	125	79.31	57.3	87.7	57.21	78.46	94.35	14.35	9.52	4.95
2-3.c62132/2/2712	serpin peptidase inhibitor, cla	147904661	NP_00108598	21.71	42.34	37.86	22.35	28.68	29.34	14.73	22.43	16.83	3.63	0.27	5.01
3-6.c10615/1/2832	PREDICTED: LOW QUALIT	847146731	XP_01282223	1.49	4.53	0.74	0.61	1.69	3.13	1.19	0.65	0.3	0	0	0.09
1-2.c52361/3/1244	PREDICTED: myosin-4 [Xer	847156920	XP_0128244(	2.11	0	26.12	22.77	15.59	0.32	29.31	19.41	16.01	0	0	0
2-3.c23272/1/2506	--	--	--	0	2.53	0	0.03	0.07	0	0	0.75	0	5.86	4.46	3.09
1-2.c27390/4/1348	PREDICTED: aldehyde oxid	847167054	XP_0029377(	8.08	5.46	5.03	12.59	4.81	7.4	18.53	5.08	11.07	237.67	230.58	207.63
1-2.c51301/2/1261	PREDICTED: LOW QUALIT	573880367	XP_00662838	7.99	59.05	169	165.18	158.56	123.52	191.46	245	114.02	42.57	9.81	7.04
3-6.c14787/1/2712	PREDICTED: myosin-4 [Xer	847156920	XP_0128244(	29.64	15.27	11.27	6.05	5.75	0	9.62	8.39	8.95	0	0	0
2-3.c42887/1/2256	PREDICTED: fat storage-ind	847092180	XP_00491081	0.22	0	1.24	2.02	1.77	2.53	0.45	0.83	0.77	0.11	0	0
1-2.c1511/13/1512	PREDICTED: pyruvate dehy	505846104	XP_00461653	282.55	74.41	260.78	114.93	223.45	234.26	85.71	192.97	93.99	17.19	43.41	18.19
1-2.c45065/1/1407	--	--	--	0.3	1.21	0.1	0.12	0.11	0.79	0.27	0	0.41	15.38	14.23	23.18
2-3.c34570/1/2062.:	--	--	--	3.65	2.15	0	10.03	0	0	0	0	11.57	103.52	206.06	82.68
2-3.c18392/933/211	pyruvate kinase PKM isoforn	118405080	NP_00107253	226.98	23.51	482.33	445.02	425.59	286.71	320.94	420.48	133.37	22.05	0	116.99
1-2.c4207/4/1836	PREDICTED: very-long-chai	847170095	XP_01280858	45.43	1.39	72.15	50.14	58.73	80.84	1.27	62.6	63.51	0	0	20.26
1-2.c36794/1/1539	phosphoglucomutase-1 [Cric	537225147	ERE83081.1	0.32	1.01	0	0	16.35	13.3	13.19	18.13	0	0	0	0
1-2.c34288/1/1519	--	--	--	0	0	0	0	0	0	0	0	0	33.74	46.5	0
1-2.c36844/1/1392	PREDICTED: erythrocyte ba	512857691	XP_00491668	0.09	0.09	0	0	0.04	0	4.04	0	0	14.45	4.49	34.22
2-3.c18599/13/2513	dihydrolipoamide branched c	147900987	NP_00108779	8.69	15.12	12.5	11.99	10.4	14.02	11.65	4.99	10.68	1.25	0.47	1.84
2-3.c28716/1/2439	PREDICTED: importin subu	301621730	XP_00294019	4.14	16.07	2.66	7.32	0	10.49	7.26	0.12	4.54	0	0	0
1-2.c52192/7/1661	protease, serine, 8 precursor [	148222777	NP_00108172	5.07	2.57	4.56	1.35	3.94	2.79	5.6	0.49	1.2	138.82	136.2	421.3
2-3.c34015/1/2802.	acidic leucine-rich nuclear ph	148228396	NP_00108315	6.94	12.72	13.3	6.03	15.83	9.8	4.76	2.96	2.68	0	0	1.72
2-3.c48219/1/2457.	PREDICTED: GMP reductas	597771204	XP_00724938	36.85	8.31	36.11	24.78	45.63	30.24	19.64	23.26	13.13	3.4	1.07	1.95
3-6.c15031/1/2778	--	--	--	0	0	0	0	0	0.15	1.3	0	0	3.23	4.13	6
1-2.c38125/1/1745.	ariadne RBR E3 ubiquitin pro	148237715	NP_00109024	7.55	8.42	0.84	3.4	13.64	11.57	0	4.57	3.6	0	0	0
2-3.c17535/1/2173	--	--	--	5.79	5.09	9.81	9.37	7.81	10.73	9.42	11.46	17.58	0.45	1.19	0.97
1-2.c49962/1/1597	alanyl-tRNA synthetase doma	148230330	NP_0010802(	0.35	2.61	0.9	1.21	1.6	4.42	0	0.65	0	0	0	0
1-2.c8948/5/1623	PREDICTED: heterogeneous	847142386	XP_0128215(	19.09	297.43	27.55	23.99	38.84	56.2	22.8	25.32	15.62	0	0.63	11.62
3-6.c13494/1/3225	ethanolamine-phosphate phos	147905107	NP_00108663	3.94	2.69	0.56	1.7	4.71	0	23.25	3.19	3.23	48.43	59.89	74.93
3-6.c2598/14/3419	PREDICTED: ubiquitin-prot	847099174	XP_01281214	2.82	0	4.39	2.02	3.2	4.59	0.66	3	0	0	0	0.56
1-2.c7357/1/1326	--	--	--	0.06	35.58	5.97	6.18	4.74	25.14	9.55	4.6	0	0	0	0
2-3.c60290/1/2304	PREDICTED: TPR and anky	847139365	XP_01282093	0.41	1.25	0.02	0	0	0	1.18	0	0	1.95	4.05	2.94
1-2.c18504/1/1816.	PREDICTED: actin, alpha ca	803091489	XP_00401102	6684.95	2303.66	16106.55	9933.69	10253.91	8370.97	6525.5	10907.14	8562.34	1472.55	596.57	1766.09
2-3.c10741/1/2517	--	--	--	10.13	19.58	26	7.27	7.67	10.31	4.52	8.94	12.46	0.67	1.17	0.64
1-2.c46014/1/1238	--	--	--	0.63	3.26	0	0.56	0	0.17	2.18	0	0	16.86	22.82	6.93
2-3.c50849/13/1905	PREDICTED: methylmalona	558137835	XP_0061178(	88.61	69.91	94.91	91.82	70.31	71.61	78.31	75.46	70.89	11.06	8.77	10.99
2-3.c10655/1/1942	PREDICTED: aryl hydrocarb	847107950	XP_01281382	0.4	5.09	0.42	0.37	0.08	0.58	0.52	0.34	0.41	10.45	20.36	12.52

2-3.c24029/1/2691	PREDICTED: collagen alpha	847156838	XP_01282444	3.12	32.15	10.49	6.08	7.94	15.7	2.25	4.35	6.4	0.6	0	2.05
1-2.c28809/2/1493	heterogeneous nuclear ribonu	188528957	NP_0011209(	0	0	0	0.38	0	1.37	3.74	0	2.88	20.38	22.85	22.29
1-2.c11192/1/1621	parvalbumin beta protein [Ra	20797085	CAC95153.1	21656.01	6639.1	41711.65	26311.28	37425.45	80449.06	31251.84	38625.53	33129.32	6698.85	4332.16	6065.22
2-3.c48490/1/2763	ubiquitin-like modifier activa	147902384	NP_00108398	0.1	35.51	0.11	4.35	5.64	7.61	2.54	2.78	3.52	0	1.8	0
2-3.c23312/1/2164.	FAS-associated factor 2 [Xen	113931576	NP_00103923	0	0	0	0	0	0	3.27	0	2.92	18.51	20.17	0
1-2.c29334/3/1213	Y-box-binding protein 3 [Xer	62751871	NP_00101585	214.32	148.36	256.71	156.76	190.2	379.35	179.13	251.4	244.63	0	0	82.03
2-3.c7287/5/2214	--	--	--	21.43	8.39	17.01	7.42	12.02	0.25	6.6	4.98	4.38	0	0	0
3-6.c6789/1/2831	PREDICTED: nebulin isofori	847166512	XP_01282661	2.88	0.12	0	0.05	16.43	10.31	4.32	3.1	0	0	0	0
2-3.c35251/1/2532	ryanodine receptor beta isofo	29501272	BAA04647.2	1.74	1.24	0	4.33	5.24	6.61	6.39	5.15	0	0	0	1.06
1-2.c36168/5/1492	LOC443630 protein, partial [	49118212	AAH73209.1	1.14	2.08	0.74	1.42	2.3	3.58	0.64	1.29	0.65	0	0	0
2-3.c2386/17/2552	RNA-binding protein 24-A [X	147907395	NP_00108099	47.34	54.84	42.82	38.46	69.74	68.86	38.17	45.17	52.68	9.4	6.39	7.83
2-3.c4996/4/2031	SET and MYND domain con	148225404	NP_00108546	1.64	0.44	0.93	1.05	3.58	6.36	0.2	0.73	2.9	0.15	0.08	0.06
2-3.c43362/1/2519	LOC399077 protein [Xenopu	111185534	AAH63720.2	1.81	1.24	4.14	2.21	3.51	3.29	0.63	1.41	1.16	0.17	0.13	0.11
1-2.c17294/2/1431	PREDICTED: UTP--glucose-	847127397	XP_01281836	0	0	0	0	0	0	0	0	0	91.55	188.09	0.09
2-3.c36298/1/2238.:	PREDICTED: ankyrin repeat	512839176	XP_00293509	56.41	1.37	24.12	26	34.23	3.09	9.65	37.9	40.66	0.56	0	0
2-3.c43657/1/2120	--	--	--	8.72	5.06	3.47	4.28	3.79	3.47	13.95	3.25	6.21	120.81	61.74	116.17
1-2.c13524/1/1580	PHD finger protein 1 [Xenop	148233497	NP_00108195	0	0	0	0	0	0	2.23	0	0	4.35	7.23	1.79
1-2.c8353/5/1478	matrix metalloproteinase-9TI	147901642	NP_00109130	0	0	0.21	0	0	0	1.16	0	0	36.46	52.64	0.05
2-3.c27019/1/2532	PREDICTED: uridine-cytidir	611978736	XP_00747556	16.39	2.05	17.63	9.45	11.3	3.04	11.29	9.66	20.09	0	0	1.62
1-2.c48859/2/1591	ER membrane protein comple	147906471	NP_00108517	0	0	0	0	0	0	0	0.75	0	5.48	4.9	2.82
2-3.c56390/1/2024	--	--	--	2.99	6.55	5.24	3.62	1.86	6.02	1.77	3.44	3.65	0	0.56	0
2-3.c59711/4/1930	PREDICTED: beta-enolase [.	637381761	XP_00812393	0	0	0	0	0	0	8.9	0	0.06	36.82	64.11	0.03
2-3.c55830/1/2472	PREDICTED: spermine oxid	557322059	XP_00603412	3.17	15.57	1.15	3.97	13.75	9.3	25.03	4.21	18.02	347.33	241.95	140.33
1-2.c14295/1/1176	fragile X mental retardation, a	89268870	CAJ81473.1	54.48	14.65	50.74	13.74	18.14	39.3	12.42	16.79	38.53	3.02	0.52	3.15
1-2.c18559/1/1428	PREDICTED: coiled-coil doi	641766368	XP_00816803	1.36	0.42	0	0	0	0	1.01	0	0	5.1	6.9	3.81
2-3.c15497/1/2007.	--	--	--	0.34	0	0	0	0	0	3.67	1.51	0	8.47	9.49	3.53
2-3.c1170/135/1969	ornithine decarboxylase 1 [X	148237898	NP_00108016	50.67	373.47	28.03	29.91	24.85	34.23	27.56	32.38	41.27	8.31	0.16	2.38
1-2.c25692/1/1624	PREDICTED: serine-rich coi	677907793	XP_00906869	0	2.16	0	0	0	0	0.57	0	0	4.17	7.2	1.36
1-2.c22957/1/1806	sorting nexin 9 [Xenopus laev	148237966	NP_00108661	4.67	0	0	0	0	0	0	0	0	1.17	7.11	5.72
3-6.c5993/1/2939.1	unnamed protein product [On	642115355	CDQ68211.1	2.78	4.48	4.61	8.02	5.32	17.62	7.21	2.95	7.33	1.02	0.59	1.05
1-2.c44729/1/1761	PREDICTED: protein transpo	847087089	XP_01281626	0.76	6.35	0.52	0.91	2.23	4.2	1.37	0.95	0.16	0	0.09	0
1-2.c5314/1/9256.2	PREDICTED: LOW QUALI	637377851	XP_00812323	35.62	7.07	131.28	183.4	161.65	72.02	58.18	101.4	239.91	9.27	5.26	37.91
2-3.c39462/1/2138.	PREDICTED: cytosolic non-	591359347	XP_00705465	75.52	6.05	0.33	24.1	71.54	3.38	37.5	46.19	74.06	851.38	985.56	761.42
1-2.c3696/2/1340	--	--	--	0	0	0	0	0	0	0	0	0	22.74	33.51	1.08
2-3.c51064/7/2467	myosin, heavy chain 4, skelet	47575800	NP_00100124	6.35	0.04	113.16	45.02	17.71	1.76	160.93	67.8	128.11	0	0	0
2-3.c10903/1/2540	solute carrier family 37 (gluc	148232489	NP_00108046	0	0	0	0	0	0	0.1	5.28	0.12	0	11.22	14.17
1-2.c18240/1/1332.	keratin 5, type II [Xenopus (S	118404108	NP_00107237	0.14	0	0.7	0.12	0.07	6.23	3.04	0.2	1.57	47.33	59.7	129.58
1-2.c52270/4/1243	PREDICTED: heat shock cog	512854037	XP_00293757	111.23	0.06	0	97.95	0.91	47	448.84	67.06	134.44	981.64	1650.89	898.97
1-2.c12693/2/1741	coatomer protein complex, su	148235681	NP_00108357	0	6.92	0	0	0	0	7.88	0.53	0.64	3.47	5.45	3.26
1-2.c19626/1/1231	--	--	--	2.61	0	0	0	0	0	0	0	2.52	4.32	7.17	6.99
1-2.c26228/1/1428	--	--	--	6.89	9.6	0.55	5.37	0	1.79	12.06	3.44	6.36	65.85	79.74	56.35
2-3.c13458/1/6442.:	PREDICTED: dihydrolipoyl	602630522	XP_00742217	13.73	1.71	7.05	24.76	14.3	11.41	3.22	4.66	0	0	0	1.66
2-3.c44152/1/2137	--	--	--	46.82	35.76	23.13	29.86	32.39	11.64	12.25	11.25	27.03	0	0	8.28
1-2.c23402/1/1550	xaa-Pro dipeptidase [Xenopu:	56118636	NP_00100809	15.64	0	5.83	0.03	0.3	0	0.11	10.11	9.59	40.46	3.84	52.44
1-2.c29487/1/1321	PREDICTED: conserved olig	847150844	XP_01282297	0	0	0	0	0	0	5.5	0	0	33.16	45.41	0
1-2.c24396/4/1524	MGC52527 protein [Xenopus	28838469	AAH47954.1	0.71	2.92	0.55	2.92	2.06	0.01	13.07	3.02	2.33	56.65	55.8	40.92
1-2.c25396/1/1280	zinc finger protein 207 [Xenc	89269088	CAJ81941.1	0.99	0	0	0	0	0	0	0	0	6.73	8.02	2.06
2-3.c16165/9/2218	PREDICTED: alanine--glyox	525026364	XP_00506063	17.28	6.04	13.65	11.88	11.25	5.25	6.49	11.13	10.57	1.25	1.14	0.65
2-3.c39785/1/2172.	PREDICTED: tRNA-dihydro	301615796	XP_00293736	0	0	0	0	0	0	1.2	0	0	8.06	10.32	4.49
2-3.c41905/1/2061	zygotic DNA replication licer	45361635	NP_989393.1	2.44	43.95	2.29	1.67	5.3	9.64	0.27	2.44	2.26	0	0	0.4
1-2.c34794/1/6686.:	PREDICTED: actin, alpha sk	664748946	XP_00853187	51.45	17.68	69.88	45.95	42.93	41.01	20.29	40.18	29.55	6.48	3.89	4.48
1-2.c51245/1/1838	propionyl CoA carboxylase, l	147904714	NP_00108365	9.07	39.49	25.3	23.18	25.53	51.89	10.2	8.54	19.98	6.71	2.06	3.64
1-2.c6736/1/1366	pre-rRNA-processing protein	148230961	NP_00108828	2.85	0	0	2.81	1.98	9.75	0	1.65	0	0	0	0
2-3.c62682/152/220	GDP dissociation inhibitor 2	148234617	NP_00108023	1.55	3.06	1.3	1.17	1.2	2.1	3.31	0.59	3.19	0	0	0
1-2.c38269/1/1282	--	--	--	0	1.5	3.67	0	0	0	10.15	0	2.42	49.72	32.54	0
1-2.c45883/1/1450.	PREDICTED: cathepsin L1-l	558221328	XP_00613595	297.79	27.22	57.06	160.42	151.03	28.85	281.09	183.59	207.57	3531.63	2419.31	2480.56
2-3.c10511/10/2259	PREDICTED: sodium-indepe	512873189	XP_00293735	0.92	0	0	2.69	3.63	2.57	0	0.8	1.78	0	0	0.32
2-3.c28070/2/2389	MGC82073 protein [Xenopus	147905808	NP_00108636	3.66	2.14	0.62	0.08	0	0	1.18	1.93	1.1	2.73	4.58	4.75

3-6.c3323/2/3013	PREDICTED: myomesin-2 is	847130012	XP_01281893	96.09	92.03	233.85	260.41	206.25	330.94	198.71	177.64	238.05	37.68	14.8	56.08
2-3.c6529/2/2284.1	aytl3 protein [Xenopus (Silur	58400917	AAH89229.1	0.73	0	3.59	0	0	0	0	0.01	2.52	6.32	1.31	6.47
2-3.c12593/1/2077	DDB1- and CUL4-associated	45360649	NP_988998.1	0	0	0	0	0	0	2.4	2.71	0	5.06	3.93	1.3
1-2.c14995/7/1720	cyclic AMP-responsive elem	163915079	NP_00110639	0	16.51	10.25	0	0	0.1	14.95	0	10.66	38.3	0	43.45
2-3.c4239/3/2531	Uba1a protein [Xenopus laev	28958137	AAH47256.1	0	0	0	0	0	0	0	0	0	18.11	12.89	0
2-3.c36966/1/2453	PREDICTED: host cell facto	543381231	XP_0055338(	6.24	3.11	1.72	2.19	1.87	1.18	9.83	3.28	3.25	42.75	57.8	42.58
3-6.c4674/1/3456.2	PREDICTED: F-actin-cappin	395539262	XP_0037715(	17.74	0	61.5	34.54	37.65	103.01	36.8	0	0	0	0	10.33
2-3.c45644/1/1887	PREDICTED: glutamate-rich	557258909	XP_00601514	11.47	24.8	3.04	5.96	5.8	14.62	7.88	4.05	7.98	1.22	0.24	0.71
2-3.c36235/1/2359	PREDICTED: unconventiona	847102432	XP_0128129(	20.24	12.62	17.14	9.79	9.54	16.25	6.42	18.95	11.07	4.06	0	0
2-3.c27776/1/2253.	PREDICTED: phosphatidylir	847137764	XP_0128207(	2.2	6.15	1.48	1.49	1.42	3.4	0.85	1.46	2.48	52.95	52.69	60.45
2-3.c63423/4/2514	signal transducer and activato	194018632	NP_0011234(	6.1	0	2.38	5.04	0	5.64	5.47	2.52	7.83	0	0	0
2-3.c63555/4/1963	regulator of G-protein signali	291290887	NP_00116747	0	0.77	0.42	0.25	1.23	1.92	1.44	0	1.33	32.11	25.75	37.65
1-2.c5228/1/1459.1	--	--	--	6.3	7.08	8.23	2.52	7.81	5.32	3.89	4.16	4.88	0	0	0
1-2.c7618/7/1774	voltage dependent calcium ch	11414922	BAB18553.1	52.2	12.89	79.37	61.62	75.31	59.01	44.31	62.55	52.26	9.41	2.75	13.75
1-2.c20852/1/1909.:	--	--	--	0.28	0.38	0.44	3.09	0.32	0	4.73	0.68	1.01	63.2	67.91	34.7
1-2.c50320/1/1555.:	--	--	--	21.97	14.23	0	0	3.97	5.41	43.7	29.07	19.25	308.77	1762.79	813.58
1-2.c25142/1/1760	LOC733292 protein [Xenopu	68534338	AAH98985.1	26.56	7.34	34.29	70.28	33.51	28.7	76.41	47.2	79.71	0	0.05	11.54
1-2.c27666/1/1202	--	--	--	2.9	0	0	0	0	0	1.06	0.11	1.8	3.3	9.68	4.99
1-2.c4429/2/1350	--	--	--	7.39	11.52	2.33	3.64	4.17	5.33	12.38	7.28	5.79	101.51	109.57	124.07
2-3.c20773/1/2231.	PREDICTED: myozenin-2 is	847087249	XP_01281675	9.61	15.27	24.54	9.58	9.57	26.36	9.09	12.39	10	0.99	1.06	1.69
2-3.c11649/15/1952	cystathionine-beta-synthase [	148228062	NP_00108682	26.74	71.4	24.36	18.94	17.9	39.21	24.17	14.44	14.04	3.35	3.86	2.49
2-3.c10656/7/2118	NADH dehydrogenase subun	146149047	YP_00116545	8.26	0	8.78	7.04	62.34	61.01	0	45.33	6.64	0	0	2.58
3-6.c9920/1/3061.1	--	--	--	1.35	4.09	0	1.33	0.51	3.74	8.57	0.34	2.83	123.27	47.68	52.33
2-3.c15861/1/1126(	--	--	--	4.33	43.63	16	22.92	14.1	15.46	3.81	16.25	8.12	1.71	1.73	1.37
1-2.c23369/3/1359	PRPS2 protein, partial [Homo	34192133	AAH30019.2	6.27	5.08	3.33	1.88	3.77	4.75	1.11	0.99	4.22	0	0	0.17
2-3.c45328/2/1866	PREDICTED: glycogen phos	556954754	XP_0059889(	115.87	6.82	125.49	173.23	153.01	74.43	139.77	175.69	154.54	29.46	12.15	18.03
2-3.c14473/3/2193	oxysterol-binding protein-rela	71895871	NP_0010256(	0	0	0.33	0	0	0.54	1.82	0	0	25.78	11.87	6.83
2-3.c63287/1/2282	staphylococcal nuclease and t	148234849	NP_0010796(	0	11.22	5.63	4.87	4.3	13.65	1.07	3.24	2.35	0	0	1.01
1-2.c40055/2/1460	MGC52527 protein [Xenopus	28838469	AAH47954.1	2.66	0.88	0.48	2.59	2.55	1.27	13.51	2.73	3.49	52.79	76.62	51.51
1-2.c40820/1/1427	--	--	--	11.11	34.56	2.4	16.07	7.46	53.86	42.08	11.22	19.68	522.12	735.1	469.36
1-2.c41879/1/1361	coiled-coil domain-containing	56118819	NP_00100817	0	0	0	0	0	0	0	0	0	29.03	86.92	10.94
1-2.c27765/1/1343	PREDICTED: myosin-4 [Xen	847156920	XP_0128244(	0	0	94.15	28.75	17.04	0	164.65	0	0	0	0	0
1-2.c21917/3/1901	PREDICTED: uncharacterize	512834006	XP_00491352	41.73	5.49	98.86	42.79	62.39	44.36	31.84	68.34	46.09	7.97	5.02	7.86
1-2.c22438/1/1149	RecName: Full=Collagen alp	18202034	O42350.1	0	0	0	0	0	0	0	0	0	39.83	29.63	0
2-3.c1273/120/2345	LOC398314 protein [Xenopu	80477501	AAI08429.1	88.34	51.24	78.32	78.06	79.21	72.18	64.12	70.97	81	9.66	10.55	12.95
1-2.c38760/1/7715.	--	--	--	0	0	0	0	0	0	0	0.58	0	77.36	127.76	0
2-3.c7135/13/2612	PREDICTED: ATP-depender	301608355	XP_00293375	0	1.01	0	0	0	0	0	0	0	2.88	1.16	2.94
2-3.c12102/7/2352	PREDICTED: apoptosis-indu	847154756	XP_01282393	4.54	8.82	9.75	5.03	4.77	0	0	3.52	0	0	0	0
2-3.c36368/1/2260	PREDICTED: autophagy-rela	512881983	XP_00294305	0	0	0	0	0	0	2.2	6.48	0	4.77	2.88	16.45
2-3.c35137/1/2478.	GDP dissociation inhibitor 2	148234617	NP_00108023	12.43	12.77	17.54	9.79	14.49	26.98	5.64	18.7	10.85	0	0	2.86
1-2.c4958/1/1619	PREDICTED: sarcalumenin i	847166841	XP_0128267(	16.71	3.33	27.93	14.9	27.78	34.56	53.28	41.4	32.98	9.36	0	0.34
3-6.c16974/1/2817	topoisomerase (DNA) II alph	148222806	NP_0010825(	0.42	25.57	0	0.5	2	2.41	0	0.64	0.64	0	0	0.1
1-2.c17858/1/1133	isocitrate dehydrogenase [NA	71896117	NP_00102559	0	0	0	0	0	0	2.1	0.55	0.68	11.05	27.51	4.85
2-3.c11468/6/2492	PREDICTED: probable threo	530582378	XP_00528503	10.57	1.02	10.53	7.41	7.09	35.7	3.66	4.65	3.84	0	0	0.06
2-3.c13072/1/6138.:	pyruvate kinase PKM [Xenop	148225037	NP_00108434	0	6.13	0	0	0	0	0	0	18.14	42.22	24.12	0
1-2.c21117/2/1689	obg-like ATPase 1 [Xenopus	148228515	NP_00107968	75.02	8.16	118.66	83.11	114.85	200.62	92.36	116.19	99.69	18.69	13.01	23.78
1-2.c22339/1/1441.	PREDICTED: actin-related p	573898892	XP_00663717	0	0	0	0	0	0	0	0	0	166.16	304.82	0
1-2.c11818/4/1447	PREDICTED: putative solute	512834811	XP_00491363	0	0	0	12.03	0	2.48	15.19	0.05	9.21	95.69	172.34	101.47
2-3.c54668/1/2064	PREDICTED: alpha-actinin-:	558140024	XP_00611831	19.04	5.59	36.58	33.74	28.55	31	24.89	25.14	30.98	4.04	3.5	4.95
3-6.c6884/1/3512	PREDICTED: collagen alpha	847166754	XP_01282667	2.24	21.38	6.02	3.79	4.77	24.6	3.25	1.74	3.45	0	0	0
2-3.c43189/1/2239.:	--	--	--	14.6	4.48	7.48	11.02	5.04	2.96	20.09	7.87	8.08	155.17	240.88	163.73
2-3.c29287/1/1937	PREDICTED: vitamin K-dep	530565501	XP_0052793(	0.04	15.54	0.04	0	0	0	0	0	12.95	16.66	21.9	0
1-2.c17195/5/1401	PREDICTED: RING finger p	847086255	XP_01281404	20.77	4.14	9.6	0	9.62	13.86	4.52	2.25	6.64	0	0	0
1-2.c2683/13/1137	myosin essential light chain f	52699568	AAU86906.1	7501.26	1517.79	13627.67	11838.16	12903.99	11766.81	11045.22	14223.59	9857.66	2038.63	1063.41	2329.9
1-2.c33564/1/4712.:	PREDICTED: cellular nuclei	594662064	XP_00717923	11.51	62.98	29.85	0.81	33.29	58.1	0	4.37	0	0	0	0
3-6.c13196/2/4133	PREDICTED: MICOS comp	512817128	XP_00293732	25.17	17.06	32.16	25	27.91	30.01	20.26	24.03	22.84	2.9	2.93	5.67
2-3.c21281/2/2602	--	--	--	0	0	0.03	0	0	0	18.51	0	0.99	21.34	0	44.29
2-3.c27211/3/2092	PREDICTED: uncharacterize	847119040	XP_01281681	0.82	22.99	2.53	0.67	9.57	8.86	0.63	0.82	1.43	0.12	0	0.08

2-3.c43847/1/2165	Dupd1 protein [Xenopus laev	68534444	AAH99351.1	0.82	0	2.37	1.54	1.99	2.35	3.73	1.01	1.81	0.05	0	0.04
2-3.c1590/14/2402	protein NipSnap homolog 2 [	113931288	NP_00103909	51.41	46.61	60.68	62.37	57.99	62.46	37.68	34.65	54.47	7.81	4.34	13.16
2-3.c29674/1/2159	--	--	--	62.55	54.91	117.04	45.12	50.46	156.02	32.29	42.04	49.96	1.35	0.58	5.93
2-3.c15516/1/2393.	PREDICTED: homocysteine-	847117414	XP_01281624	0	2.91	6.45	2.86	1.52	6.19	0	3.68	8.79	0	0	0
2-3.c2003/10/2542	PREDICTED: synaptopodin		512828724	XP_00293730	11.19	2.71	18	10.25	13.61	12.84	4.58	8.27	16.26	0.45	1.81	2.14
1-2.c39753/1/1273	PREDICTED: cysteine prote	847163930	XP_01282600	0	0	0	1.54	0	0	0	0.04	0	19.48	32.43	13.55
1-2.c25338/1/1542	--	--	--	19.46	26.79	8.89	18.7	12.01	11.29	78.91	14.06	13.24	292.48	353.48	333.47
3-6.c3048/7/3247	PREDICTED: mothers agains	512884416	XP_00492024	0.83	0	0.01	0.41	1.15	1.27	1.48	0	0	0	0	0
2-3.c62069/1/2481	Citrate synthase, mitochondri	808865946	KKF16719.1	0	0	0	0	0	0	0	0	6.44	9.47	0	10.21
1-2.c31032/1/1408	PREDICTED: sodium/potass	620963907	XP_00766720	0	0	0	0	0	0	0	0	0	14.99	15.95	0.43
2-3.c13636/1/2339.	PREDICTED: myocyte-speci	847084858	XP_01282407	4.25	6.79	4.12	4.28	7.29	10.77	5.31	2.36	4.43	1.5	0.37	0.15
2-3.c19105/1/2460	core histone H2A/H2B/H3/H	768197465	KJH53109.1	0	0	0	0	5.21	4.8	0	3.57	4.61	0	0	0
1-2.c13457/3/1595	--	--	--	110.92	50.8	158.37	193.31	176.71	169.09	232.32	225.97	206.48	35.28	2.18	39.08
2-3.c4345/1/2571	PREDICTED: eukaryotic tra	847099370	XP_01281221	72.41	95.62	46.64	20.28	44.55	44.34	3.84	17.02	47.11	0.79	0	12.7
1-2.c5473/1/2978.1	granulin precursor [Xenopus	147900546	NP_00108067	64.86	20.88	47	39.43	24.09	17.27	35.29	70.83	56.58	683.91	648.12	565.83
1-2.c3872/1/1886	PREDICTED: DTW domain-	569004435	XP_00652627	5.8	0	3.09	3.02	9.77	5.49	9.93	8.65	3.53	0	0	0
1-2.c44768/2/1734	cystathionine-beta-synthase [	148228062	NP_00108682	12.1	4.2	2.9	4.44	0.76	9.94	6.61	2.83	10.87	0	0	0
1-2.c51604/11/1487	PREDICTED: sarcalumenin i	512868462	XP_00293247	134.62	26.04	133.18	204.71	145.41	73.49	131.62	163.51	207.67	23.96	8.51	29.82
1-2.c6092/1/1609	PREDICTED: keratin, type I	698421871	XP_00981374	292.62	282.25	203.82	182.77	564.43	168.63	456.05	285.04	166.33	7.12	3.25	6.44
1-2.c9069/1/1775.1	--	--	--	0.92	6.26	0.78	1.7	0.87	2.39	0.98	1.75	1.73	90.42	104.14	25.61
3-6.c3475/2/3006.1	PREDICTED: tenascin isofo	512858291	XP_00491677	0	10.2	5.73	3.05	3.4	6.58	0.09	3.94	1.82	0	0.24	0.75
1-2.c28255/1/1294	inositol hexakisphosphate kin	62859747	NP_00101595	8.57	51.77	10.03	25.08	0	0.43	27.78	26.43	26.11	218.88	132.04	251.76
2-3.c10078/1/2189	uncharacterized protein LOC	148227048	NP_00109015	0	0	0.51	0	0	0	0	0	0	3.54	4.54	0.52
2-3.c20561/1/2487.	--	--	--	4.83	8.32	2.22	6.33	1.45	5.7	24.44	4.29	8.82	136.55	154.3	113.93
2-3.c40059/2/2298	PREDICTED: myosin-7B [X	512871888	XP_00293293	0	0	0	0	0	0	0	0	0.72	5.76	16.04	2.81
1-2.c40613/2/1359	PREDICTED: protein PFC07	556961274	XP_00599098	111.93	71.11	207.27	130.47	246.16	618.78	165.67	111.74	273.99	8.33	9.07	11.02
2-3.c3209/1/2397.2	--	--	--	0	0	0	0	0	0	2.68	0	0	75.45	150.61	11.51
2-3.c23132/1/2256	ethanolamine-phosphate phos	147905107	NP_00108663	0.22	0	0	0	0.12	0.12	0.62	0	0	2.84	7.51	6.18
1-2.c11925/3/1798	leucine-rich repeat-containing	194332463	NP_00112373	10.21	10.29	5.85	10.17	6.76	11.17	3.18	2.36	9.21	0.62	1.44	0.72
1-2.c13714/2/1299	uncharacterized protein LOC	148232024	NP_00108962	0	0	0	0	0	0	1	0	1.17	10.63	1.43	20.03
1-2.c3241/1/1389	PREDICTED: integrin alpha-	556963632	XP_00599173	1.28	8.77	0.61	0.98	0	3.33	0.23	0.9	2.01	39.02	27.28	49.72
1-2.c52346/1/1455	PREDICTED: isocitrate dehy	831547357	XP_01272691	1.6	0	105.35	165.54	99.95	114.01	187.92	137.82	244.19	16.31	14.48	25.23
1-2.c19337/1/1394	PREDICTED: phosphoglycer	831541829	XP_01272486	13.25	0.89	10.99	15.76	9.3	6.7	9	7.95	17.98	1.41	0	1.33
1-2.c29963/1/1482	epidermal growth factor recep	306922368	NP_00112010	0	0	0.19	0.17	0	0	0	0	1.9	7.65	9.91	3.47
1-2.c31198/1/1609	eukaryotic translation initiatio	187608058	NP_00112056	0	0	3.24	0	7.2	7.11	0	0	1.13	0	0	0
1-2.c38882/1/1604	ARP1 actin-related protein 1	148226057	NP_00108648	0	0	0	0	0	0	0	1.84	0	1.99	3.56	6.31
1-2.c42978/1/1527	--	--	--	4.46	71.67	3.81	3.04	2.74	17.25	7.19	3.78	2.8	128.39	142.94	218.91
2-3.c1492/54/2069	PREDICTED: interferon-rela	847109761	XP_01281444	2.72	0.12	0.96	6.47	0	10.28	0.3	4.87	1.05	0	0	0
1-2.c36358/1/1699	Unknown (protein for MGC:8	49257408	AAH73381.1	0	25.89	3.5	6.64	5.86	0	0	1.58	1.19	0	0	0
1-2.c30859/2/1704	pyruvate kinase PKM [Xenop	148225037	NP_00108434	0	0	0	0	0	0	0	0	0	15.76	17.15	0
3-6.c13035/2/2987	major vault protein [Xenopus	147903423	NP_00107993	0	3.35	0	0	0	0	0	0	0	11.64	8.49	0
2-3.c32790/1/2006.	PREDICTED: protein phosph	301603827	XP_00293153	0.17	2.44	1.42	1.71	1.67	1.14	0.83	0.41	1.78	28.12	35.08	58.75
1-2.c28066/1/1451.	--	--	--	1.51	12.33	0.32	0.87	0.46	0.62	1.91	0	0.96	23.78	38.66	12.87
3-6.c7494/1/2865.1	PREDICTED: mitogen-activ	512866147	XP_00491777	4.65	1.02	4.55	1.82	6.53	10.2	3.86	4.21	0.5	0	0.06	0.63
1-2.c4442/3/1736.1	PREDICTED: RNA-binding	126313602	XP_00136386	23.68	36.76	1.52	10.97	7.64	8.03	0	4	11.69	1.79	0	0
2-3.c11906/1/2335.	--	--	--	17.96	8.05	25.34	25.36	19.21	50.94	8.01	10.5	6.96	4.49	3.96	2.25
1-2.c2909/4/1339	Xgs protein [Xenopus laevis]	213623564	AAI69913.1	36.87	11.79	3.94	24.98	19.37	0	41.22	25.73	29.02	456.76	321.42	277.74
1-2.c7518/1/1741	--	--	--	0.49	0	0.7	0	0	0	1.2	0.89	1.87	6.82	1.08	3.64
1-2.c7587/3/1643	dusp1 protein [Xenopus (Silu	49257734	AAH74564.1	0	0	0	0	0	0	0	0	0	27.07	18.22	0
3-6.c6377/1/2921	H(+)/Cl(-) exchange transpor	113931518	NP_00103921	0.34	0	0	0.35	1.46	1.23	1.2	0.62	0	0	0	0
3-6.c9802/1/3496	PREDICTED: band 4.1-like	847168910	XP_01280820	11.12	24.79	10.26	5.31	7.46	2.19	1.93	5.07	3.6	0	0.5	1.07
1-2.c20966/1/1472	solute carrier family 3, memb	148229024	NP_00108024	0	6.55	5.8	32.46	14.29	1.37	13.28	0	25.6	0	0	0
1-2.c2657/8/1384	uncharacterized protein LOC	148232016	NP_00109117	0	0	0	0	0	0	0	0	0	49.88	96.73	0
2-3.c26979/1/1800	solute carrier family 2 (facilit	147902370	NP_00108560	0	1.5	0	0	0	0	0	0	0	6.32	49.86	16.83
1-2.c33276/1/1492	--	--	--	6.02	10.88	0	0	0	0	0	0	0	20.47	0.13	16.73
2-3.c3775/4/2100	PREDICTED: myomegalin-li	591373231	XP_00706131	2.17	8.25	8.76	0	7.67	13.95	0	2.59	0	0	0	0
3-6.c16971/1/2756	PREDICTED: neuroblast dif	847171283	XP_01280904	9.29	0	0.34	0.73	0	0	6.46	3.51	0	10.04	7.99	9.55
1-2.c4760/2/1412	PREDICTED: adipocyte enha	847115150	XP_01281556	0	0	3.22	0	0	0	3.1	0	0	2.48	6.07	5.63

1-2.c32963/2/1670	PREDICTED: eukaryotic trar	847089040	XP_01282181	0	0	0	0	0	0	0	0	0	2.12	6.86	2.25
1-2.c7952/1/1254.1	larval type I keratin [Rana cat	134140868	ABO61147.1	1383.21	225.25	311.26	727.83	849.96	147.37	656.89	310.24	219.68	20.51	40.8	23.55
2-3.c27671/1/2376.1	signal transducer and activato	148232026	NP_00108467	17.32	1.65	1.72	3.37	6.93	1.86	11.92	4.09	13.04	127.44	145.18	57.73
1-2.c13589/1/1526	myosin, light chain 1, alkali, j	374533792	AEZ53809.1	834.74	385.48	1921.93	1356.84	1516.35	2261.34	1706.92	2023.02	1313.45	299.45	198.13	297.55
2-3.c21143/1/2514.1	glyceraldehyde-3-phosphate d	300679438	ADK27487.1	0	0	0	0	0	0	0	0	0	1205.25	4121.26	0
1-2.c35656/1/1300	myosin, heavy chain 4, skelet	47575800	NP_00100124	0	0.41	25.82	10.66	8.48	0	11.09	0	0	0	0	0
2-3.c20801/1/2095	unnamed protein product [Ma	90083332	BAE90748.1	7.77	19.17	7.55	6.91	10.13	12.53	5.38	3.03	7.63	1.65	0.39	1.15
1-2.c38826/1/1418	PREDICTED: lysine-specific	637270584	XP_0081032C	0	0	0	0.21	0	0	1.08	1	0.13	8.28	7.71	5.86
1-2.c49435/1/1592	integral membrane protein Gl	187608073	NP_00112016	0	0	0	0	0	0	0	0	0	5.86	12.64	1.96
2-3.c28612/1/1899	--	--	--	1.77	0.74	4.23	10.55	8.43	1.36	5.42	2.84	5.72	0.29	0	0.17
1-2.c36703/1/1476	PREDICTED: guanine nuclec	620975525	XP_00766788	31.48	81.76	22.51	36.02	19.08	18	17.56	13.86	24.27	0	0	8.31
3-6.c16141/1/3594	PREDICTED: zinc finger CC	301609401	XP_00293425	5.41	2.93	2.77	3.63	5.04	3.71	2.17	2.23	2.44	0	0.41	0.78
1-2.c2670/3/1814.1	--	--	--	2.57	0.01	0.02	0	0.8	0.05	0.87	1.77	1.01	5.06	26.02	29.69
1-2.c6446/1/5773.1	--	--	--	2.83	12.43	0	1.48	0.41	4.32	6.15	1.45	1.95	61.18	96.3	30.51
1-2.c25313/1/1741.	PREDICTED: vitamin D3 hy	847119946	XP_01281704	0	0	0	0	0	0	0	1.91	0	28.01	62.55	4.96
1-2.c48959/1/1555.	--	--	--	2.88	18.33	0.73	6.32	3.7	1.83	17.96	11.65	0.69	41.88	150.51	173.98
2-3.c29689/1/1899	Keratin, type II cytoskeletal c	677541627	KFR03099.1	0	0	0	0	0	0	0	0	0	14.57	19.91	0
2-3.c59469/1/2000.	ORF2 [Acanthochelys spixii]	6576738	BAA88337.1	0.57	1.9	0.21	1.65	0.6	0.4	6.63	0	0.86	33.36	35.89	36.48
2-3.c59506/1/5587.	PREDICTED: H/ACA ribonu	543368307	XP_00552749	11.51	86.08	5.95	20.82	7.71	18.42	8.5	8.44	6.06	0.61	0.27	4.54
3-6.c1659/30/3423	PREDICTED: PDZ and LIM	847087387	XP_01281714	2.59	1.39	7.54	17.42	11.38	11.23	8.74	4.8	10.22	1.95	0.88	2.57
3-6.c7761/2/3419	PREDICTED: importin-5 iso	847100607	XP_01281263	5.76	8.64	6.33	4.22	5.26	6.75	3.91	4.15	4.01	0.44	0.62	0.74
3-6.c18515/1/2956	PREDICTED: myomesin-1 [	512850290	XP_00293895	3.5	1.68	16.37	10.12	13.69	17.68	10.73	9.06	9.57	1.11	0.15	3.71
1-2.c19906/3/1557	PREDICTED: aldehyde oxida	847167054	XP_00293776	5.44	9.34	5.93	8.68	11.87	11.58	23.65	14.88	15.61	134.39	202.4	352.56
1-2.c27892/1/5516.	LOC398627 protein [Xenopu	83318199	AAI08446.1	2.14	0	0	0	0	0	0	0.99	0.89	47	49.92	0
1-2.c30708/4/1460	PREDICTED: splicing factor	637335070	XP_00811537	0	0	0	10.01	13.42	23.86	2.16	4.34	18.49	3.19	1.77	0.64
1-2.c33601/1/10937	translation elongation factor 1	11228569	AAG33072.1	0	201.15	148.89	89.79	50.03	0	0	0	46.88	0	0	0
1-2.c8392/7/1247	LIM domain-binding protein	45360839	NP_989095.1	34.05	10.73	112.35	71.97	105.32	98.87	63.39	54.12	80.93	12.1	2.33	24.41
2-3.c11906/1/2335.	--	--	--	0.65	0	3.72	0.69	2.07	4.82	1.08	0.43	0	0	0	0
2-3.c30253/1/2123	alternative protein ATP1B1 [	440575751	CCO13684.1	0	0	0.27	0	0.53	0	0.9	1.99	0	3.99	13.79	8.48
2-3.c30837/1/2282	--	--	--	3.47	6.2	4.65	4.39	2.88	3.82	0.62	2.26	6.87	0	0.25	0.49
2-3.c39921/1/2459	PREDICTED: cytoplasmic dy	530648788	XP_00531093	0	0	0	0	0	0	0	0	0.2	8.89	9.62	0
2-3.c54323/1/2232	PREDICTED: protein phosph	591348122	XP_00705272	0	0	0	0	0	0	0	2	0	1.23	4.63	2.14
3-6.c16034/1/3528	periostin precursor [Xenopus	163915007	NP_00110637	0	4.1	9.64	0	5.54	3.93	0	0	2.29	0	0	0
2-3.c19233/1/2458.	PREDICTED: keratin-associa	767897549	XP_01150701	2.29	2.51	4.77	7.62	3.29	3.75	4.47	2.68	6.4	133.08	230.66	79.31
2-3.c1622/29/2043	PREDICTED: beta-taxilin [F	410960156	XP_00398666	0.48	4.91	23.46	32.63	45.55	18.93	0	28.93	0	0	0.44	12.32
1-2.c2790/3/1646	caveolin 3, gene 2 [Xenopus	156717622	NP_00109635	29.74	20.86	51.08	19.6	28.51	46.99	22.2	33.39	44.13	3.09	4.63	5.34
1-2.c5980/1/1544	--	--	--	3.65	1.51	0	2.63	0	0.54	7.86	3.41	9.05	28.46	30.99	32.58
1-2.c15012/1/1365	--	--	--	0	0	0	0	0	0	0	0	0	17.75	19.16	0
2-3.c60612/1/2582.1	--	--	--	20.8	7.77	0	3.54	0	3.27	13.68	0	0	75.42	86.07	31.94
2-3.c1591/42/1902	ATP synthase subunit alpha,	71896075	NP_00102561	1219.55	445.17	849.6	785.41	889.62	482.82	470.07	702.59	657	105.87	96.66	151.86
1-2.c18400/1/1340	heterogeneous nuclear ribonu	154147563	NP_00109375	0	0	0	2.42	0	0	14.29	0	4.15	38.86	45.14	11.81
1-2.c29585/1/14478	PREDICTED: elongation fac	700374727	XP_0099383C	0.79	9.6	4.04	6.74	4.07	3.18	4.05	4	3.83	0	0	0
1-2.c3892/1/1437	PREDICTED: suppressor of	847154706	XP_01282391	0	0	0	0	0	0	0	0	1.34	16.78	18.92	0
2-3.c36070/1/2036	--	--	--	16.66	5.06	45.25	17.42	21.44	35.72	19.21	19.28	23.01	3.94	2.5	3.87
1-2.c5430/5/1442	--	--	--	2.55	0	0	0	0	9.2	17.95	4.95	0	64.44	105.88	38.72
1-2.c32954/1/1318	cathepsin B precursor [Xenop	45361295	NP_989225.1	142.31	74.74	24.28	68.98	0	23.84	104.71	69.31	88.84	813.95	515.71	626.48
3-6.c15662/1/2682	PREDICTED: fermitin family	512861543	XP_00491721	14.38	15.49	17.54	12.15	9.79	9.9	6.42	8.08	9.32	0.82	1.27	1.96
2-3.c5225/2/1911	dermatopontin precursor [Xe	71896109	NP_00102559	12.35	22.47	27.24	19.25	18.75	34.27	15.5	7.81	18.16	5.99	0.48	3.2
1-2.c25758/1/1561	PREDICTED: septin 10 isofo	847099625	XP_0128123C	5.52	16.33	4.54	0	0.01	0	6.97	0	2.99	12.19	0	14.08
2-3.c34429/1/1925	PREDICTED: collagen alpha	591360730	XP_00705528	0.34	0	8.87	5.4	3.54	10.19	1.81	5.46	9.78	1.72	0	0
1-2.c11013/1/1407	proteasome activator complex	156717318	NP_0010962C	3.33	24.97	4.34	3.85	4.3	9.61	5.66	3.19	0	0.41	0	0.55
1-2.c16950/1/1772	aldehyde dehydrogenase fam	113931284	NP_00103908	3.67	6.71	3.29	4.3	5.68	2.29	0	1.99	2.12	0.76	0	0
2-3.c12028/1/2198	--	--	--	4.15	17.09	10.99	7.83	9.27	22.95	8.78	5.37	7.13	0	0.48	3.28
2-3.c43339/1/2437.	PREDICTED: ras-related pro	795342433	XP_01178278	0	2.14	0	1.86	0	2.66	0	0	12.51	24.53	32.64	52
3-6.c2301/16/2888	PREDICTED: probable threo	591363151	XP_00705648	2.62	2.23	4.54	0.89	2.22	1.64	0.3	1.65	0.4	0.14	0	0
1-2.c35504/3/1329	hedgehog acyltransferase-like	147901798	NP_00107949	0	3.28	0	0	0	0	0.03	5.8	0	18.89	23.33	0
1-2.c49784/1/1608	PREDICTED: heterogeneous	847142386	XP_0128215C	5.82	0	0	0	0	0	0	0	1.79	14.11	16.11	0
2-3.c1679/15/2396	heat shock 70 kDa protein 14	62751575	NP_00101578	0	17.05	1.19	0	4	4.9	1.55	0.91	1.76	0	0	0

2-3.c15478/1/2388.	sarcoplasmic/endoplasmic ret	383511148	AFH40438.1	7155.65	806.19	6426.29	7773.4	9682.35	5223.48	4200.18	7215.24	5571.86	820.94	486.97	2257.59
1-2.c31640/2/1222	PREDICTED: glucose-6-pho	704456039	XP_01008651	0	0	0	0	0	23.83	118.47	33.22	0	245.44	291.51	85.67
2-3.c19330/1/2401	--	--	--	5.58	3.03	7.92	4.59	4.8	13.15	6.24	1.63	0.59	0.75	0.79	0.52
1-2.c16396/4/1445	Short-chain specific acyl-Co/	226372490	ACO51870.1	11.52	5.94	9.83	6.49	7.46	6.25	2.96	10.56	5.86	1.58	0	0
2-3.c2672/3/2208	PREDICTED: collagen alpha	847156838	XP_01282444	2.98	22.5	3.73	1.58	0.2	2.55	1.62	1.22	3.18	0	0	0
1-2.c45689/1/1233	PREDICTED: heterogeneous	704176135	XP_01013362	0	60.74	3.64	0	10.37	16.9	0	1.69	0	0	0	0
2-3.c23478/1/5379	calsequestrin 1 (fast-twitch, s	148231736	NP_00108022	1843.97	280.28	3093.72	2285.41	2991.73	2565.74	1682.38	2494.75	2381.07	398.04	155.52	684.71
2-3.c47840/1/2313.	beta-hexosaminidase subunit	187607505	NP_0011206(	1.21	1.18	0.88	1.13	0.58	0.3	3.25	1.5	1.82	28.2	24.94	24.76
1-2.c4663/1/1466	heavy chain 6 cardiac muscle	388242704	AFK15622.1	0.99	6.96	1.56	3.92	0.24	3.56	1.8	0	2.57	0	0	0
1-2.c27092/95/139(	uncharacterized protein LOC	45361313	NP_989234.1	224.88	79.76	218.56	156.09	143.8	40.89	55.82	99.16	140.46	6.13	4.6	21.23
2-3.c4491/1/2343	RecName: Full=Low density	73921717	Q67FQ3.1	0	0.47	0	0	0	0	0	0.55	0	3.47	0.46	3.39
2-3.c8421/2/2274	PREDICTED: regenerating is	301610175	XP_00293462	6.47	0.12	5.85	14.17	7.02	10.38	5.6	4.38	0.12	0	0.09	3.26
1-2.c22994/1/1268	zinc finger protein 9 (a cellul:	89269563	CAJ82604.1	0	0	0	0	0	0	0	0	0	7.98	3.11	4.17
1-2.c6130/1/1349	PREDICTED: glycogen phos	512834332	XP_00293541	6.82	3.18	77.55	21.51	42.06	16.88	39.32	31.18	39.32	9.38	1.04	0
2-3.c46091/1/2509.	PREDICTED: blood vessel e	847126715	XP_01281814	14.94	17.53	18.79	16.76	10.08	30.56	9.51	8.58	11.67	1.91	2.07	3.21
1-2.c12033/3/1528	PREDICTED: dihydrolipoyl	602630522	XP_00742217	41.28	12.39	24.87	23.32	19.2	29.17	19.42	15.32	17.71	2.13	5.92	2.28
2-3.c44694/61/2377	Keratin, type II cytoskeletal c	697450143	KGL89199.1	132.35	156.62	82.79	83.74	304.91	82.9	175.84	141.55	83.88	1.34	1.5	1.96
1-2.c2952/17/1571	PDZ and LIM domain 7 (enig	148223185	NP_00108073	6.79	12.2	34.9	18.43	22.71	37.25	30.08	30.51	21.49	3.68	2.14	4.59
3-6.c15928/1/2713	PREDICTED: amyloid beta /	847099265	XP_01281217	0	9.5	6.54	0.01	4.64	3.76	0	0	2.15	0	0	0
1-2.c41590/1/1590	PREDICTED: protein PFC07	556961274	XP_00599098	1.96	0.44	5.77	2.33	4.15	9.91	2.49	1.68	2.97	0	0.18	0.46
2-3.c21891/1/2417	--	--	--	1.32	7.58	1.84	1.33	2.76	3.7	1.11	0.88	0.29	0	0	0.34
1-2.c39875/1/1400	PREDICTED: nuclear transcı	847086080	XP_01281352	0	0	0.9	0	0	0	0	0.79	0	5.14	6.18	2.45
1-2.c50556/2/1401	PREDICTED: histone acetylt	847162472	XP_01282552	0.23	12.45	0	0.72	0	0.08	2.42	3.6	6.83	15.93	13.78	8.42
3-6.c3547/3/3060	PREDICTED: CCAAT/enhar	847126903	XP_01281820	1.09	1.82	0.67	0.87	2.84	4.8	2.28	0	0.03	0.11	0	0.28
1-2.c50400/1/1690	--	--	--	0.64	3.62	0.28	1.15	0.47	0.93	3.27	0.84	1.07	17.46	15.57	37.45
3-6.c8716/3/3260	PREDICTED: cell division c	641789631	XP_0053004(	0.44	12.21	1.67	1.03	1.64	4.97	0	0.41	0.78	0	0	0.08
1-2.c21007/1/1751	--	--	--	11.64	4.58	0	6.66	0.39	3.27	20.73	14.24	12.07	70.57	77.19	81.74
1-2.c19922/1/1341	--	--	--	1.55	1.94	0	0.39	0	0.74	0	0.26	2.83	16.54	17.46	11.69
1-2.c22401/1/1500	cortexin-3 [Xenopus (Siluran	351542156	NP_00116515	5.54	10.11	10.52	4.17	6.67	10.09	6.74	4.4	6.44	0.27	0.31	0.22
1-2.c7064/4/1773.1	PREDICTED: monoamine o	847098632	XP_01281195	2.18	0.91	1.77	1.73	1.57	2.82	2.17	3.78	1.98	33.54	49.9	72.73
2-3.c27334/1/2262	--	--	--	1.38	15.02	1.86	2.21	1.36	6.49	5.1	0.65	3.08	57.51	86.64	69.47
2-3.c62865/3/2333	PREDICTED: zinc finger and	301618660	XP_00293871	2.66	7.87	3.33	2.92	5.57	4.42	0.01	2.56	2.52	0	0	0.87
1-2.c35572/1/9289.	novel aldo-keto reductase fan	113931624	NP_00103926	0	0	0	0	0	0	0	0	0	7.79	11.17	5.01
2-3.c32721/1/1021(	--	--	--	9.55	24.81	12.17	8.66	11.7	16.63	2.38	5.45	17.82	1.04	0.61	1.37
1-2.c49578/1/1822	RNA-binding protein VgRBF	147902659	NP_00108391	0	1.47	0	0	0	1.8	3.69	0	5.05	19.62	23.4	9.24
1-2.c6867/1/1642	PREDICTED: choline/ethanc	558142258	XP_00609261	0	0.08	0.97	0	0	0	0.83	0	0	1.97	6.14	3.06
2-3.c47700/1/2052.:	--	--	--	0	0	0	0	3.06	0.72	0	0	5.4	60.06	69.32	29.53
1-2.c29177/1/1201	PREDICTED: myomesin-2 is	847130012	XP_01281893	70.79	89.25	155.59	251.89	138.96	222.23	143.54	127.63	174.31	42.58	12.05	41.88
2-3.c60646/1/2634	PREDICTED: fermitin family	512861543	XP_00491721	2.13	3.62	7.28	2.07	2.88	3.06	1.94	1.98	2.01	0.13	0.09	0.26
1-2.c8211/2/1309	phosphatidylinositol transfer	147903972	NP_00108027	0	0	0	0	0	0	0	0	0	17.83	16.14	0
2-3.c39049/1/1894	--	--	--	0	0	0	0	0	0	0	0	0	3.81	4.79	0.59
1-2.c36260/1/1373	glutaryl-CoA dehydrogenase	148225687	NP_00108662	0	0	0	0	0	0	0	0	0	15.03	15.18	0
1-2.c21881/45/154(	PREDICTED: tartrate-resista	847170157	XP_01280861	1.94	0.71	0.27	0.82	0	0	0.5	0.31	0.75	7.96	16.68	13.22
1-2.c4313/1/1288	heterogeneous nuclear ribonu	89272515	CAJ81560.1	2.68	39.16	6.85	4.19	5.12	14.01	2.5	3.05	3.18	0.53	0.18	0.6
1-2.c46751/1/5313.	PREDICTED: ATP synthase	670982575	XP_00868189	0	0	0	0	0	0	0	0	0	129.82	311.75	0
3-6.c14511/1/4710	alpha 1 type I collagen [Rana	3242649	BAA29028.1	0.94	23.92	5.56	3.21	6.01	15.14	1.72	1.68	3.75	0.27	0	0.46
2-3.c19308/1/2127	PREDICTED: sodium-indepe	512873189	XP_00293735	1.67	0	0.16	0.44	0	0	1.12	3.31	1.94	7.38	5.12	9.7
2-3.c36455/2/2037	PREDICTED: LOW QUALIT	803125841	XP_00401312	11.66	1.92	12.92	17.92	27.46	10.57	17.97	12.61	11.11	2.92	0	4.69
2-3.c8521/4/2228	ubiquilin-1 [Xenopus (Silurar	45361287	NP_989221.1	5.82	0	0.71	3.12	3.11	3.37	0.21	0.24	0	0	0.7	0
2-3.c26945/1/2424.	uncharacterized protein LOC	147898849	NP_00109041	0.35	2.67	1.23	2.79	1.46	1.91	0	0.53	0.62	0	0	0
2-3.c12572/3/2352	PREDICTED: baculoviral IA	847099212	XP_01281215	19.02	7.43	7.2	7.45	0	0	14.74	8.45	22.13	39.02	54.46	74.02
1-2.c16946/1/1389	adenylosuccinate lyase [Xenc	148226240	NP_00108059	0	0	0	0	0	0	0	0	0	5.16	11.45	1.95
1-2.c43683/1/1584	PREDICTED: cold shock do	847087986	XP_01281925	0	0	0	0	0	0	0	0	0	14.41	12.41	0
2-3.c3663/2/2238	ppp1r3a protein [Xenopus (Si	117558559	AAI27365.1	4.44	0.36	4.15	5.49	3.63	2.05	2.58	0	3.4	0.58	0.35	0
3-6.c4883/1/3272	PREDICTED: glucocorticoid	847109993	XP_01281452	0.7	0	1.58	2.27	2.35	0	0	0	0	0	0	0
1-2.c40711/1/1869	Calnexin, partial [Anas platyı	483507247	EOA97896.1	0	0	0	0	0	0	6.39	0	0	15.34	7.55	1.16
2-3.c11984/1/1932	ppp1r3a protein [Xenopus (Si	117558559	AAI27365.1	8.88	2.39	7.04	10.24	9.98	6.35	3.53	0.13	9.04	0.09	2.99	0.39
3-6.c2265/17/3059	PREDICTED: large subunit (	507699078	XP_00464405	3.16	5.63	1.24	2.09	2.68	6.22	1.6	2.38	1.35	0	0.4	0.55

1-2.c23358/1/1262	cordon-bleu WH2 repeat prot	148230755	NP_00107964	22.68	18.04	6.59	0	8	12.06	0.13	0.71	0	0	0	0
2-3.c53737/1/2587	PREDICTED: protein FAM2	847109632	XP_0128144(	2.02	0	2.97	1.89	3.89	0.38	0	0.75	0	0	0	0
1-2.c16419/12/1901	PREDICTED: solute carrier f	823467263	XP_01242793	44.77	57.39	77.72	34.94	54.22	139.83	45.54	44.61	54.93	5.21	2.35	3.77
2-3.c54865/2/2324	PREDICTED: glycogen debr	847125952	XP_01281792	47.94	4.42	61.88	95.49	74.36	22.23	35.74	50.65	89.66	3.4	2.94	8.91
1-2.c10716/6/1445	--	--	--	25.34	0.48	21.16	44.2	22.46	13.33	25.08	26.96	0	0	0	4.85
3-6.c2694/10/3348	AP-1 complex subunit beta-1	189230240	NP_00112144	0.05	0.13	1.19	0	2.35	2.48	1.75	0	0	0	0	0
1-2.c27498/1/1550	protein kinase C substrate 80l	147900167	NP_00108712	0	0	0.31	0	0	0	7.74	0	0.89	4.82	3.94	2.94
2-3.c30396/1/2157	phytanoyl-CoA hydroxylase-	118403656	NP_00107232	1.58	1.21	0.23	1.16	0.45	0.93	0.95	0.16	2.36	17.74	11.3	34.67
1-2.c30483/1/7284.:	PREDICTED: beclin 1-assoc	512862076	XP_00293377	20.97	2.4	0.45	6.19	7.46	0	0	0	0	0	0	0
1-2.c25880/1/1518	PREDICTED: rho GTPase-ac	527274234	XP_00515487	0	0	0	0.35	3.42	2.34	0	0	2.81	0	0	0
1-2.c30367/2/1758	PREDICTED: RNA-binding	847135541	XP_01282024	0	24.17	0	0	0	0	0	0	0	14.28	20.74	0
2-3.c37792/1/2598	PREDICTED: myosin-4 [Xer	847156920	XP_0128244(	16.79	0	47.23	19.92	54.86	0	25.03	61.16	25.08	0	0	0
2-3.c29193/1/2252	PREDICTED: probable ATP-	348511436	XP_00344325	14.28	35.83	14.69	11.41	9.48	11.92	6.93	10.91	2.82	1.84	0	2.15
1-2.c36045/1/5902.	PDZ and LIM domain 7 (enig	148223185	NP_00108073	227.38	119.99	617.92	322.27	462.86	403.83	417.77	412.17	277.16	48.38	27.89	112.77
1-2.c15798/1/1397	WD repeat domain 13 [Xenoj	56118773	NP_00100805	0	0	0	0	0	0	0	0	3.17	7.5	16.53	2.24
2-3.c29386/1/2237	forkhead box protein J2 [Xen	349501020	NP_00100567	0	0	0	0	0	0	0	0	0	0.63	3.54	3.49
1-2.c32762/1/1251	PREDICTED: cyclin-L1 isof	594620350	XP_00716489	21.72	31.83	11.7	2.78	0	5.74	15.01	15.99	19.17	53.62	83.29	54.44
3-6.c15536/2/2623	proteasome activator comple:	156717318	NP_0010962(	2.77	19.55	2.46	1.42	6.47	11.18	0	0	3.88	0	0	0.45
2-3.c4500/1/2542	PREDICTED: nascent polyp	847099444	XP_01281224	27.16	34.42	24.77	19	13.45	32.74	17.12	12.84	15.97	3.32	4.14	2.63
3-6.c14405/1/3674	PREDICTED: SAFB-like tra	847162187	XP_01282542	0	0	1.03	2.59	2.03	0	0	0	0	0	0	0
2-3.c26614/1/1956.	aspartyl/asparaginyl beta-hyd	62751990	NP_00101573	8.33	3.66	12.18	9.85	10.89	20.12	9.6	10.4	8.47	0.85	0.85	2.89
3-6.c18230/1/2839.	GMP reductase 1 [Xenopus (	166158200	NP_00110729	53.17	4.49	39.24	37.98	42.13	39.27	23.12	39.18	49.98	3.74	3.58	10.83
2-3.c20605/1/2198	tripeptidyl-peptidase 1 precur	156717508	NP_00109629	0	0	0	11.11	15.96	0	0	3.65	0.54	0	0	0
3-6.c18532/1/2701	--	--	--	0	0	0	0	0	0	5.23	0	0	2.93	1.43	1.62
2-3.c53457/1/2303.	PREDICTED: DNA damage-	512818445	XP_0049115(	2.68	0.47	0.73	1.52	3.08	2	0.25	0.94	5.09	0	0.2	0
3-6.c5038/1/2692	RecName: Full=Sarcoplasmic	9789732	Q92105.1	6.36	0	4.81	6.98	7.33	3.85	5.35	5.64	5.22	0.33	1.74	0.35
1-2.c19417/1/1944.	--	--	--	1.58	2.08	2.18	4.31	4.11	3.07	2.2	0	1.39	0	0	0
2-3.c54769/1/2329	mitochondrial ribosomal prot	148224662	NP_00108778	4.63	7.42	4.14	4.44	3.87	7.35	1.77	3.1	3.1	0.79	0.31	0.43
1-2.c45721/1/1836.	PREDICTED: glycerophosph	847157563	XP_01282457	0	1.45	0.55	0.43	0.83	0.76	7.28	0	0.51	25.88	29.63	64.78
1-2.c33864/3/1887.:	--	--	--	4.56	0.88	8.17	4.52	9.79	5.87	2.81	1.09	1.15	0	0	0
1-2.c12985/2/1537	eukaryotic translation initiatic	148238002	NP_00108714	8.19	0	6.99	4.95	3.07	6.1	1.72	4.25	4.83	0	0.97	0
1-2.c39589/1/1277	PREDICTED: histone acetylt	637375986	XP_00812292	0.93	2.16	0	0	0	0	0.93	1.47	0.67	6.87	11.57	1.25
2-3.c48377/1/1944.:	--	--	--	8.14	14.03	1.31	7.18	9.6	5.6	23.55	21.61	9.55	165.78	227.95	133.71
1-2.c29826/1/1484	PREDICTED: inhibitor of gr	847109424	XP_01281433	0	0	0	0.36	0	0	3.65	0.38	2.15	8.15	9.92	7.23
2-3.c53140/1/2342.:	GJ17223 [Drosophila virilis]:	195386312	XP_00205184	7.81	158.55	45.39	19.01	8.43	21.49	13.84	10.34	22.46	0.3	0	1.55
3-6.c2706/7/2943	PREDICTED: transmembran	847171915	XP_01280927	0.87	0	0.83	0.53	2.89	1.04	0	0.74	0	0	0	0
3-6.c14449/1/2897	epithelial splicing regulatory	52346028	NP_00100505	0	3.57	6.74	1.86	0.3	0.88	0	0	0	0	0	0
1-2.c43902/1/1928	--	--	--	193.65	19.3	202.95	419.31	193.73	121.06	90.84	171.92	421.46	9.94	0	25.86
1-2.c20689/1/1547	--	--	--	26.82	14.46	24.32	27.37	28.28	37.8	34.48	42.04	64.9	2.2	11.03	2.37
1-2.c46057/1/1319	--	--	--	0	0	0	0	0	0	0	0	0	7.95	34.58	8.4
2-3.c17567/1/2301.	--	--	--	4.98	3.02	0.27	0	0.06	0	0	0.32	0	6.78	3.11	6.91
2-3.c40477/1/2179	PREDICTED: zinc transporte	301618652	XP_00293871	5.32	27.8	0.3	0	0	0	4.77	0	0	13.73	9.33	0
2-3.c46491/1/2259	PREDICTED: phosphorylase	847153425	XP_01282352	3.06	0.99	4.96	6.78	5.45	5.58	6.61	2.96	8.63	0.47	0.38	1.01
2-3.c49279/2/2197	PREDICTED: tubulin beta-4l	557016409	XP_00600863	0	0	0	0	0	0	0	0	0.38	11.26	8.21	0
2-3.c15731/1/3610.	calsequestrin 1 (fast-twitch, s	148231736	NP_00108022	125.87	0	831.98	428.62	605.93	159.35	340.93	691.79	386.82	0	0	104.93
2-3.c28969/1/1968.	PREDICTED: complement fa	847125968	XP_01281793	2.38	3.22	5.17	3.71	6.91	1.55	1.07	1.76	1.54	0.18	0	0
1-2.c30926/2/1310	--	--	--	4.9	0	8.55	2.85	4.18	1.48	2.6	4.49	5.62	0	0.13	0
1-2.c8309/1/1455	--	--	--	4.16	10.09	3.25	2.88	2.09	5.75	2.97	2.35	3.39	90.47	70.97	68.17
2-3.c27682/1/2331	--	--	--	3.84	0.37	4.13	4.39	4.81	1.41	1.73	4.22	1.36	0.91	0	0
2-3.c62973/3/2121	endothelin converting enzym	132424622	ABO33468.1	0	0	0	0	0	0	0	0	0	0.72	3.94	3.07
3-6.c3588/5/3338	transducin beta-like protein 3	62859901	NP_00101731	3.12	10.94	2.61	3	2.61	7.57	2.66	2.03	2.06	0.22	0.46	0.71
1-2.c17425/1/1487	fibroblast growth factor 6 pre	218664453	NP_00113629	1.36	4.07	3.19	2.07	2.87	3.24	1.26	1.38	2.32	0	0	0.1
3-6.c7657/2/3333	PREDICTED: alpha-protein l	512852902	XP_00491603	79.58	8.16	33.36	41.94	71.79	15.71	34.52	33.42	37.89	1.92	1.56	3.59
1-2.c11616/1/1684	--	--	--	0.94	0	0	0	0	0	2.35	0.76	0	0.38	5.79	4.98
2-3.c12718/1/1902	--	--	--	9.86	11.13	9.08	11.11	6.59	10.68	33.91	8.65	7.18	180.79	202.16	182.85
1-2.c18626/1/1432	PREDICTED: 1-acyl-sn-glyc	847152645	XP_01282327	1.5	1.82	0	0	0	1.78	5.78	3.09	0	19.32	19.4	15.1
1-2.c35069/1/1529.:	PREDICTED: kelch-like prot	149408781	XP_00150787	34.83	6.81	3.1	5	3.29	1.42	14.05	23.65	18.89	86.07	118.39	86.9
1-2.c19181/2/1350	--	--	--	0	4.03	0	0	3.06	0	14.08	3.25	4.07	23.89	41.56	25.83

3-6.c18031/1/3199	Mgc53960 protein [Xenopus	115528672	AAI24909.1	2.08	0.81	2.08	3.41	3.73	2.11	1.9	2.2	1.82	0.23	0.26	0.38
1-2.c29028/2/1830	Map2k3 protein [Xenopus lae	50604142	AAH77760.1	8.09	1.15	18.04	9.24	11.87	10.5	8.63	8.66	10.39	1.79	0.33	1.54
2-3.c12176/1/5964.:	PREDICTED: phosphoglycer	432887007	XP_0040749(	325.14	18.86	307.89	600.51	316.38	195.08	225.36	334.41	402.89	43.6	11	50.74
2-3.c7378/1/2151	PREDICTED: protein phosph	823396584	XP_01241593	0	1.53	1.39	1.1	1.04	2.17	0	0	0	0	0	0
1-2.c40637/1/1345	--	--	--	0	0	0	0	0	0	0	0	0	8.45	21.11	2.99
2-3.c21223/7/2347	PREDICTED: probable 28S r	301624894	XP_00294172	32.36	54.69	16.12	17.89	15.21	24.55	18.16	15.98	17.56	3.04	0.96	4.3
2-3.c60943/1/2539	eukaryotic translation initiatio	52346034	NP_00100506	9.75	23.57	14.2	16.97	12.81	14.65	7.44	12.27	7.18	2.71	0.45	3.16
2-3.c5330/1/2190.1	--	--	--	0	0	2.54	8.47	0	0	3.35	0.84	0.55	103.43	239.11	83.23
2-3.c26256/4/2183	Myozenin-1-like [Xenopus la	148237556	NP_00107945	7.7	1.53	13.46	12.06	12.03	12.67	16.85	10.36	23.47	2.17	1.13	1.82
1-2.c29621/1/1669	PREDICTED: phosphoribosy	847162833	XP_01282564	16.25	37.99	5.22	11.5	4.71	8.33	19.63	10.32	19.45	96.3	198.77	195.3
1-2.c44684/1/1472	granulins precursor [Xenopus	118403712	NP_00107216	44.61	21.24	6.67	3.18	0.19	2.88	5.32	0	6.64	0	0	0
2-3.c33274/1/2381.:	PREDICTED: actin, alpha ca	637243920	XP_00321452	1127.05	164.01	3153.35	1523.26	1183.13	1965.29	3075.6	3931.57	1521.52	266.72	229.42	310.03
1-2.c38912/1/1602	--	--	--	0.42	0.01	0	0	0	0	0	0.45	1.01	7.61	6.61	0.38
2-3.c8559/1/2485.1	--	--	--	2.22	2.47	1.35	2.11	2.77	2.8	0	2.74	3.39	112.45	59.17	46.19
1-2.c4368/1/1798	PREDICTED: calcium-bindi	847163610	XP_01282589	0	0	0	0	0	0	3.21	0	0	2.95	4.08	2.49
3-6.c8064/2/3110	breast cancer anti-estrogen re	147906605	NP_00108519	0.63	1.5	0.91	2.22	1.64	0.53	0.37	1.32	1.58	0.18	0	0
1-2.c14112/3/1436	PREDICTED: adenylate kina	530600033	XP_0052935(	1979.78	278.16	2899.27	3501.76	2864.31	1541.4	2529.65	2893.89	2570.23	457.74	435.15	559.41
1-2.c17517/2/1393	PREDICTED: pescadillo hon	847086285	XP_01281415	21.45	37.94	8.17	14.66	5.07	17.65	13.79	14.09	12.81	2.63	0.46	1.13
2-3.c1437/48/2329	PREDICTED: propionyl-Co/	301615335	XP_00293713	8.28	18.52	7.19	6.35	8.28	21.45	7.32	4.29	5.36	1.16	2.18	0
1-2.c24414/5/1753	reticulon-3-A [Xenopus laevi	148236603	NP_00108571	2.31	2.71	1.08	1.12	2.55	1	0	0	0	0	0	0
1-2.c4491/8/1453	PREDICTED: glycogen phos	556954754	XP_00598898	119.88	4.22	110.24	179.46	182.89	94.6	127.01	237.6	160.21	33.08	27.56	23.08
2-3.c9571/1/2047	--	--	--	3.59	0.82	5.62	6.35	4.38	8.38	0.65	1.1	0.87	1.41	0.36	0.3
1-2.c32648/1/1689	--	--	--	4.68	0	0	0	0	0	0	0	5.1	3.52	2.55	3.98
2-3.c41107/1/2432	hydroxyacyl-CoA dehydroge	148238090	NP_00108561	0	0.54	0	0	0	0	0.37	0	0.65	0	5.65	6.67
1-2.c41847/2/1471	PREDICTED: catenin beta-1	556987425	XP_00599905	0	0	0	0	0	0	0	0	0	25.84	49.45	0
2-3.c21599/1/2146	--	--	--	4.81	0.47	0.84	3.02	3.24	0	1.42	2.68	4.51	0	0	0
1-2.c7783/1/1587	Mapre1-prov protein [Xenop	27924168	AAH44954.1	0	0	0	0	0	0	0.77	0	5.54	11.25	13.49	0
1-2.c49951/1/1668	ZPR1 zinc finger [Xenopus la	189217508	NP_00112122	3.74	0	0	0	0	0	8.95	0	0	0	11.04	9.99
2-3.c49174/1/2348.	--	--	--	60.11	17.31	47.14	29.2	36.37	6.78	16.07	69.78	35.85	1.72	0.57	1.87
2-3.c53219/1/2491	--	--	--	0	1.42	1.06	0	0	0.5	1.6	0	0	7.06	6.37	6.54
3-6.c2861/5/3389	apelin preproprotein [Xenopu	284413797	NP_00116514	0.79	2.33	0.38	0.31	0.87	1.11	0.11	0	0.49	0	0	0
3-6.c3252/3/3154	lysyl oxidase homolog 2 prec	213982721	NP_00113552	3.42	28.3	14.32	10.11	15.24	24.49	5.92	5.49	5.29	1.71	0.17	5.2
2-3.c53842/1/2100	tripeptidyl-peptidase 1 precur	156717508	NP_00109629	0	1.04	3.38	5.14	3.68	0	3.92	0.23	7.26	0	0	0
1-2.c40635/1/8999.	PREDICTED: nuclease-sensi	594676952	XP_00718627	0	0	0	0	0	0	0	0	0	0	28.68	26.3
1-2.c45931/1/1365	PREDICTED: hydroxysteroi	847099649	XP_01281231	0.58	1.93	0.26	0	0.84	1.75	7.77	0.31	0	13.47	20.81	42.27
1-2.c9600/9/1592	PREDICTED: ras-related pro	573880320	XP_0066283(	0	0	3.22	0.81	8.06	3.7	0	0	0	0	0	0
2-3.c15125/1/2502	--	--	--	0.42	0	0.45	0.63	1.01	0	3.07	1.32	1.94	11.43	9.48	23.9
2-3.c21894/1/6385.:	--	--	--	9.84	5.02	0.03	0	5.98	9.29	25.77	14.95	31.14	116.51	117.3	114.87
2-3.c5585/1/2068	PREDICTED: circadian-asso	512875732	XP_00491897	0	1.47	0.1	0	0	0	0	0	0	3.17	5.87	0.74
1-2.c22950/1/1271	eukaryotic translation initiatio	113931184	NP_00103903	1.57	0	0	0	0	0	0.88	0.43	0.55	4.72	8.03	2.37
1-2.c27975/1/1490	--	--	--	3.07	6.35	0.21	0	0	0	3.6	2.99	0	8.18	5.29	1.17
1-2.c26081/1/1305	secreted frizzled-related prote	148226186	NP_00108066	0	42.72	0	0	0	0	0	0	2.21	2.12	3.79	11.62
1-2.c33269/1/1310.:	--	--	--	42.79	31.86	4.57	4.84	10.5	10.91	38.14	28.89	9.15	212.83	261.57	140.84
1-2.c35234/1/1459	granulins precursor [Xenopus	118403712	NP_00107216	0	0	1.27	0	0	0	1.83	0	2.46	44.68	103.4	0
2-3.c13370/1/1964.	--	--	--	11.87	7.7	6.2	9.84	7.47	5.87	14.5	8.68	9.99	102.37	148.18	212.4
2-3.c44940/3/2359	PREDICTED: glycogen debr	847125952	XP_01281792	0.62	0	11.02	11.14	11.23	2.83	17.41	6.1	9.51	0.29	0.29	1.75
2-3.c32130/1/2463	cysteine--tRNA ligase, cytopl	58332582	NP_00101136	0	3.91	0	0	0	0	0	0	0	2.51	2.58	1.29
1-2.c24296/1/1760	splicing factor proline/glutam	189230206	NP_00112142	8.82	0.71	19.17	0	0	0	19.6	2.27	0	9.54	9.01	0
1-2.c28231/1/1565.:	--	--	--	3.81	4.55	3.02	3.95	2.03	4.71	9.58	6.29	6.68	52.24	151.8	87.86
2-3.c22840/1/2323.	uncharacterized protein LOC	147901083	NP_0010845(	0	0	0	0	0	0	0.11	0	0	2.25	3.98	1.32
2-3.c27284/2/1994	--	--	--	6.07	35.29	0.03	0	0.04	0	0.09	0.08	0	23.99	0	16.85
3-6.c15308/1/4354	collagen, type 1, alpha 2 prec	147898763	NP_00108072	21.17	1033.2	180.18	99.87	36.15	0	55.88	68.88	83.48	0.06	0	0
2-3.c20686/5/2784.:	--	--	--	47.68	54.73	0.26	0.16	0.32	0	111.59	0.38	0.41	5.74	270.66	157.03
1-2.c51503/3/1275	uncharacterized protein LOC	147903581	NP_00108456	31.19	8.76	13.36	16.19	16.1	18.72	8.28	20.15	14.74	0.59	0	5.18
1-2.c41378/1/1425	--	--	--	0	0	1.24	0.29	0	1.18	8.65	0	1.44	158.58	207.35	8.11
2-3.c14966/2/2019	calcium/calmodulin-depender	166795935	NP_00110772	0.59	0	0	1.92	1.91	2.06	0	0	3.65	0.15	0	0
2-3.c2960/13/2370	cadherin-13 precursor [Xeno	156718012	NP_00109654	3.32	7.98	5.92	5.01	8.02	9.18	4.98	5.31	5.78	0.88	0.29	1.49
1-2.c8709/2/1204	uncharacterized protein LOC	351542224	NP_00112019	4.26	7.26	4.2	6.24	4.46	5.64	26.68	5.13	4.77	106.01	122.31	109.39

1-2.c25727/1/1406	--	--	--	6.47	10.75	4.53	4.82	1.16	4.3	5.93	4.69	5.48	89.31	74.6	55.45
3-6.c9799/1/2553	PREDICTED: host cell factor	543381231	XP_0055338(	0.19	1.53	0.32	0.62	0.19	0	1.77	0	0	5.77	7.68	13.08
3-6.c15325/1/3418	cGMP-dependent 3',5'-cyclic	118404742	NP_0010726(	25.8	28.29	11.55	10.77	8.67	18.58	2.48	2.66	11.32	3.44	1.42	1.19
2-3.c13458/1/6442.:	PREDICTED: dihydrolipoyl	512830506	XP_00293316	115.64	52.34	78.94	95.88	96.75	78.54	41.91	55.31	69.88	10.45	14.46	17.19
1-2.c18682/1/1404	glycine--tRNA ligase [Xenop	55926180	NP_00100749	11.03	19.47	4.39	0	0.05	1.39	11.19	3.72	2.88	15.86	24.17	7.47
3-6.c14381/1/3475	PREDICTED: collagen alpha	591360730	XP_00705528	0	17.07	0.5	4.05	0	8.84	0	3.03	0	0	0	0
1-2.c15706/1/1237	predicted protein [Nematoste	156405864	XP_00164095	5.48	0.28	0.64	1.19	2.84	0.46	2.09	3.99	0.93	53.35	29.42	34.44
3-6.c10742/1/3522.:	--	--	--	2.32	3.8	2.77	5.79	5.66	7.19	13.77	3.73	5.76	104.91	189.83	97.45
1-2.c30027/1/1481.	PREDICTED: gastric intrinsi	637248235	XP_00810889	0.55	0.25	0	0.72	4.37	9.17	13.11	4.56	4.31	74.88	67.09	188.56
2-3.c28174/1/2164	PREDICTED: DCN1-like pro	564336204	XP_00623232	6.29	3.61	3.47	3.68	4.58	8.54	4.37	2.39	2.24	0.67	0	0.91
3-6.c13464/1/2771	importin-9 [Xenopus (Siluran	148235030	NP_00109064	2.63	4.79	2.1	2.08	3.65	1.99	0.56	2.28	1.37	0.15	0	0.37
1-2.c45199/1/1078	Transposable element Tc3 tra	294896790	XP_00277572	2.65	0	0	0	0	0	1.39	0	2.93	15.83	0	14.64
1-2.c51362/2/1231	PREDICTED: protein PFC07	556961274	XP_00599098	8.95	4.38	12.87	14.05	24.8	62.96	20.56	7.89	21.57	3.74	0.34	0.58
2-3.c34296/1/2538	PREDICTED: collagen alpha	847166756	XP_01282667	9.71	87.71	16.29	9.24	14.86	51.49	3.65	8.72	7.76	0.04	0.14	0.55
1-2.c14503/1/1299.	--	--	--	0	0	0	0	0	3.88	0.3	0	0	66.1	32.25	31.01
2-3.c30736/5/2679	PREDICTED: histone-argini	301627458	XP_00294288	0.37	2.19	0	1.93	1.08	0.3	0.52	0.96	0.34	0	0	0.04
2-3.c62299/1/2324	MGC84250 protein [Xenopus	148225296	NP_0010879(	107.86	8.06	121.95	224.28	120.82	75.22	96.43	116.85	176.65	19.47	5.05	24.91
1-2.c48740/1/1509	NIMA-related kinase 6 [Xeno	148228408	NP_00108065	2.72	2.81	7.57	5.82	8.88	1.73	4.46	2.65	3.74	0	1.07	0
1-2.c28764/1/6859.:	PREDICTED: cold-inducible	837829631	XP_00459569	699.82	1169.03	495.97	248.1	513.72	467.84	78.49	257.29	135.83	0	0	191.98
3-6.c16522/1/3292	PREDICTED: fat storage-ind	847092180	XP_00491081	0.52	0.97	1.6	2.38	1.75	3.03	1.65	1.2	1.63	0.25	0.08	0.23
2-3.c19435/1/2623	RNA-binding protein 24-A [X	147907395	NP_00108099	3.16	1.8	5.67	4	4.51	11.18	0	4.29	5.04	1.49	0	0.84
1-2.c21166/1/1351	uncharacterized protein LOC	148227144	NP_00108893	0	0	0	0	0	0	0	0	0	17.11	25.94	0
2-3.c61614/11/2077	--	--	--	0	0	0.17	2.98	3.88	0	0	0	3.5	0	0	0
1-2.c31224/1/1217	--	--	--	4.14	0	0	0.7	0	2.03	8.03	4.97	3.08	30.83	24.37	19.07
1-2.c25693/1/1894	--	--	--	19.4	19.05	4.29	6.81	5.5	7.18	31.92	7.09	5.91	119.89	173.02	92.51
1-2.c30602/1/1614	--	--	--	0	0	0	0.85	0	0	0	0	0	10.66	15.55	10.93
1-2.c45922/1/1643	alpha1 type II collagen [Cync	5360532	BAA82043.1	0	12.22	0	0	0	0	0	2.51	0.62	3.84	3.2	2.84
1-2.c45083/1/1247	--	--	--	8.59	56.39	8.06	10.05	7.18	17.71	2.75	5.42	16.16	2.75	0.75	0.36
2-3.c33444/1/2308	PREDICTED: ubiquitin-asso	847089063	XP_0128218(	0	0	0	0	0	0	0	0	0	6.86	6.11	0
2-3.c11939/1/2565.	PREDICTED: 28S ribosomal	847132351	XP_01281932	11.2	10.1	5.2	9.4	4.81	8.87	2.38	4.37	3.76	0.25	0	1.31
3-6.c3603/3/2868.1	PREDICTED: serine/threonin	655880357	XP_00827209	1.4	0.7	0.7	0.57	0.38	1.54	0.47	0.25	1.34	22.11	31.59	29.41
1-2.c40546/1/1217	PolyADP-ribose glycohydrol	226371920	ACO51585.1	0	0	0.16	1.13	0	0	4.37	2.05	1.24	21.99	11.3	11.8
2-3.c23181/1/2198	glycosylphosphatidylinositol	62752867	NP_0010158(	0	0	0	0	0	0	0.22	0	0	6.81	6.54	0
2-3.c26656/1/2017.	PREDICTED: cofilin-2 isofo	558137250	XP_00611773	217.8	173.06	311.89	169.4	221.85	535.97	281.28	248.99	249.9	24.84	60.59	40.14
1-2.c6272/8/1727	inositol hexakisphosphate kin	62859747	NP_00101595	2.22	12.86	2.38	0	3.58	4.61	0	0	0	0	0	0
1-2.c29927/2/1244	--	--	--	22.37	208.1	13.32	17.89	15.99	96.82	16.54	13.53	26.77	648.62	809.75	793.46
2-3.c36027/2/2343	PREDICTED: glypican-4 [Xe	301606027	XP_00293263	0	4.99	0.78	1.81	1.28	0.16	0	2.24	0.98	0	0	0
1-2.c6866/1/1354	tripartite motif-containing pro	56118797	NP_00100818	102.16	77.2	131.87	79.91	87.9	122.65	81.83	115.76	96.56	15.03	26.18	13.05
1-2.c39201/1/1520.	--	--	--	2.41	3.53	1.2	2.39	1.35	3.53	1	0.77	3.34	71.06	52.19	90.6
1-2.c47345/2/1255	periostin precursor [Xenopus	163915007	NP_00110637	17.62	52.09	32.17	27.79	27.79	30.78	24.9	16.65	29.17	7.88	1.37	4.02
1-2.c2420/19/1330	lumican precursor [Xenopus (	58332414	NP_0010110(	25.75	72.98	64.88	34.57	34.78	76.07	22.59	29.22	24.06	7.18	2.63	12.66
2-3.c10654/1/2168	PREDICTED: UPF0668 prot	557321340	XP_00603378	0	0	0	0	0	0	0.12	0	3.23	1.29	3.12	2.98
2-3.c39555/1/1988	PREDICTED: very-long-chai	847170095	XP_01280858	5.96	4.63	32.26	18.54	21.85	35.5	48.67	23.08	16.67	5.69	2.58	4.05
2-3.c41520/1/2520.	uncharacterized protein LOC	147905311	NP_0010906(	0	0	0	0	0.21	0	0	0	0.27	9.57	7.01	16.08
2-3.c59496/1/2513.	PREDICTED: MAP/microtul	823462103	XP_01242959	0	0.65	1.39	1.01	1.39	3.14	0.53	2.54	0	0	0	0
1-2.c8014/1/1956	periostin precursor [Xenopus	163915007	NP_00110637	4.24	61.69	12.19	10.59	8.8	14.63	9.36	3.11	8.29	2.91	0	1.68
1-2.c34981/1/1232	HLA-B associated transcript	49522510	AAH75571.1	0	1.5	0	0	0.58	1.25	1.09	0	1.16	20.07	21.62	15.41
1-2.c49695/1/1802	hypothetical protein LOTGIL	676426511	XP_00904464	6.88	28.25	1.46	1.91	3.19	2.31	4.33	4.01	0	39.92	72.04	45.53
2-3.c7976/22/2622.:	--	--	--	9.79	10.64	5.92	23.42	17.29	6.88	130.88	6.59	9.27	303.77	456.07	213.96
2-3.c43136/1/2698.:	slc35b2 protein [Xenopus (Si	39794512	AAH64205.1	0	4.6	0	7.65	0	0	10.76	0	9.1	59.63	58.57	55.11
3-6.c8838/3/2879.1	--	--	--	1.72	0	0	0	0	0	1.12	0	0	4.23	4.97	7.44
2-3.c60220/1/2537.	--	--	--	0.04	1.13	0.12	0	0.03	0.12	0.04	0.03	0.04	3.56	3.54	6.53
2-3.c42851/1/2381	uncharacterized protein LOC	148234478	NP_00108457	0	0	0	0	0	0	0.95	0	1.07	1.5	5.71	1.75
2-3.c43638/1/2572	transcription factor jun-B [Xe	113205822	NP_00103795	0	0.78	0	0	0	0	0	0	31.45	85.91	29.96	0
1-2.c9280/1/4430	desmin [Xenopus laevis]>gi|1	148224383	NP_00108017	0	0	0	0	0	0	0	0	0	14.36	14.12	0
1-2.c6234/8/1765	translation initiation factor eI	148231181	NP_00108641	0	0	0	0	9.5	22.25	0	0	0	0	0	0
1-2.c47110/1/1568	eukaryotic translation initiatic	62859649	NP_00101672	0	0	0	0	0	0	0	0	0	23.02	45.73	0
2-3.c53727/2/2028	transmembrane protein 65 [X	187607455	NP_0011203(	2.95	0	1.02	1.59	5.14	3.61	1.88	1.7	3.76	0	0	0.63

2-3.c34023/1/2276.	--	--	--	1.37	1.38	0.55	1.39	0.33	0.06	7.45	0.9	1.21	21.23	14.58	19.21
2-3.c14894/1/2269.:	--	--	--	0.44	6.25	0	0	0.67	0	0	0.43	0.16	22.82	34.53	19.69
1-2.c20969/1/1629	polypyrimidine tract-binding	118404352	NP_00107247	0	0	0	0	0	0	0	0	0	3.82	1.76	4.21
2-3.c60976/1/2329	--	--	--	0	0	0	0	0	0	0.21	0	0.88	0.75	10.89	5.57
1-2.c20287/4/1618	matrix metalloproteinase-9TH	147901642	NP_00109130	1.18	0	0	0	0	0	2.23	0	0	76.7	2.26	277.76
1-2.c50854/2/1477	PREDICTED: intracellular hy	847086348	XP_01281437	5.78	13.69	4.72	3.14	4.84	9.04	5.06	5.77	3.82	0	1.55	0
2-3.c26211/1/2402.:	--	--	--	9.09	8.53	2.27	4.09	3.81	1.55	19.79	12.54	7.77	59.42	85.08	83.83
2-3.c10337/1/2481	--	--	--	31.47	34.4	23.91	32.22	27.11	36.37	15.45	12.91	22.36	5.45	3.02	7.82
2-3.c55297/1/2435.	calcium uniporter protein, mi	194332791	NP_00112369	13.06	12.8	14.75	8.78	13.7	19.17	3.54	4.89	6.75	1.25	0.74	2.57
2-3.c26740/1/2340	PREDICTED: LOW QUALI	558146048	XP_00611959	0.93	0.22	5.46	2.54	4.54	1.74	0.6	2.07	0.43	0.68	0	0
1-2.c24763/4/1663.	PREDICTED: E3 ubiquitin-p	847107025	XP_01281364	9.47	6.03	2.17	0	1.58	4.02	7.49	7.02	13.17	53.33	31.06	36.63
3-6.c14914/1/2992	--	--	--	3.35	7.86	3.8	1.51	1.76	2.33	0.32	2.31	3.86	0.34	0	0
2-3.c8768/1/2445.2	--	--	--	2.5	2.45	0.19	0.9	1.31	4.01	3.71	0.66	3.8	60.24	88.8	53.27
1-2.c27908/1/1639	mitochondrial ATP synthase	148223359	NP_00108012	910.73	347.83	795.13	642.95	705.2	337.79	527.43	601.2	476.2	92.8	60.89	164.88
3-6.c13558/1/3921	uncharacterized protein LOC	148222755	NP_00108958	5.47	19.04	3.82	2.9	5.61	6.03	4.53	3.08	2.51	0.8	0.61	0.64
3-6.c18827/1/3169	double-stranded RNA-bindin	350606338	NP_00101137	0	1.59	0	1.94	0	1.93	0	0	1.06	0	0	0
1-2.c11532/1/1238	PREDICTED: LOW QUALI	296197462	XP_00280672	9.24	11.14	13.93	25.06	0	0	21.17	1.24	8.67	116.76	165.94	206.27
1-2.c46837/5/1616	PREDICTED: tropomyosin a	847143250	XP_01282187	0	0	0.13	0.23	0.3	0	0	0.4	0	91.14	110.67	0
2-3.c45717/1/2572	PREDICTED: striatin-interac	847114855	XP_01281550	3.24	5.14	4.14	2.17	3.4	8.15	1.12	1.36	3.09	0.4	0.68	0.18
2-3.c47497/1/1942.	--	--	--	16.72	7.09	35.79	30.13	29.15	28.99	30.53	19.75	31.11	3.16	6.29	5.16
1-2.c23339/1/1762.	uncharacterized protein LOC	148232016	NP_00109117	0.01	0	45.88	46.2	156.03	2.19	89.28	82.31	33.27	0	0	0
3-6.c15931/1/4367	collagen, type 1, alpha 2 prec	147898763	NP_00108072	1.05	9.89	0.66	1.29	2.2	3.54	0	1.33	0.21	0.72	0.04	0
1-2.c28914/2/1725	PREDICTED: junctophilin-2	301607262	XP_00293323	59.56	41.57	122.61	71.44	67.68	136.74	72.77	81.03	89.37	13.28	12.52	22.86
2-3.c30327/2/2348	RecName: Full=Voltage-dep	46576355	O57483.1	4.55	2	7.97	1.93	7.16	11.49	3.09	4.6	3.4	0	0.19	1.02
3-6.c3462/4/4186	uncharacterized protein LOC	148223037	NP_00108484	30.17	37.35	22.51	17.69	22	25.06	14.8	17.45	18.36	2.75	4.18	4.77
1-2.c17477/1/1373	LOC100145612 protein [Xen	170285152	AAI61369.1	0	0.43	0	0	0	0.09	0.99	0	2.89	4.17	11.79	3.05
1-2.c29894/1/3175.:	PREDICTED: malate dehydr	768365243	XP_01157989	479.61	186.28	467.81	431.39	508.42	177.85	231.1	328.84	377.43	53.08	17.71	128.65
2-3.c13830/1/2302	eukaryotic translation initiatio	148232020	NP_00108075	0	0	5.16	0	0	0	0	0	0.05	6	6.04	0
2-3.c42158/3/2603.:	PREDICTED: protein Shroor	558149116	XP_00612025	1.51	4.85	2.73	1.31	1.08	0.66	0.23	1.09	0.89	28.21	69.63	32.71
1-2.c13981/1/1373	--	--	--	7.96	11.45	17.4	7.73	11.2	9.07	4.33	11.16	14.75	0.53	1.09	1.31
2-3.c54729/1/2158	PREDICTED: keratin, type II	543747793	XP_00551465	26.98	7.03	22.28	16.75	51.48	10.18	55.41	20.29	16.71	0.11	0.93	0
2-3.c4502/3/2620	Geneb-a protein [Xenopus lae	213623820	AAI70263.1	0	6.06	0.29	0.11	0.27	1.66	1.44	0.2	0.14	11.78	8	24.85
2-3.c59445/1/2342	--	--	--	6.58	7.98	7.69	9.84	13.23	7.51	6.21	8.27	12.51	1.3	0.71	2.4
1-2.c32568/1/1568	MGC84445 protein [Xenopus	51703940	AAH81189.1	0	4.67	0	0	0	1.86	0.42	0	0	11.17	16.28	20.76
1-2.c17943/1/1365	nucleolin [Xenopus laevis]>g	148226518	NP_00108155	42.99	264.41	33.23	34.21	65.46	141.37	39.74	33.82	37.98	5.31	3.95	11.04
2-3.c5492/1/1976	ryanodine receptor beta isofo	29501272	BAA04647.2	10.11	12.36	54.02	31.75	32.21	34.96	50.05	44.18	56.3	8.29	1.21	7.26
1-2.c9865/11/1341	actin, alpha cardiac muscle 2	45361557	NP_989355.1	5.08	0.23	21.65	8.29	6.96	13.03	12.55	21.2	10.68	1.27	1.09	0.76
2-3.c15687/1/2537.:	transforming growth factor, b	147906015	NP_00108870	39.34	25.61	51.57	34.83	38.33	24.56	30.54	41.93	23.77	4.02	1.48	8.61
2-3.c23873/1/1947	--	--	--	5.27	4.84	1	3.11	1.52	0.96	3.01	4.74	3.77	40.37	41.76	36.94
2-3.c8669/1/2354	tubulin, gamma complex asso	148230420	NP_00108761	1.28	7.34	1.69	2.71	1.2	1.89	1.76	1	0.9	0.21	0	0
1-2.c11081/10/1599	keratin 5, gene 1 [Xenopus (S	54020785	NP_00100563	2.92	0	0	0	3.57	3.92	0	0.02	0	0	0	0
2-3.c48907/1/2161.	PREDICTED: ras-related pro	641749811	XP_00816483	0	6.68	2.26	0	0	0	1.14	4.27	0	2.08	3.02	6.19
1-2.c19268/3/1667.	--	--	--	1062.26	1228.62	1675.64	730.73	1050.06	325.85	1554.38	1018.37	903.67	268.86	46.33	50.46
1-2.c19667/1/1423	thioredoxin domain-containir	118405096	NP_00107253	0	0	0	0	0	0	0	0	0	24.94	6.94	4.2
1-2.c11476/1/1711.	--	--	--	0	1.47	4.35	15.67	4.33	6.76	0	0	4.97	0	0	0.84
1-2.c23763/1/1810	parvalbumin beta protein [Ra	20797085	CAC95153.1	292.92	90.7	574.91	405.49	604.74	1409.19	527.01	565.48	632.16	115.7	71.02	93.56
1-2.c11848/1/1583	fructose-1,6-bisphosphatase 2	291290927	NP_00116749	11.77	4.07	8.94	22.93	9.14	12.87	17.43	15	22.93	1.68	4.62	0.45
1-2.c46671/1/1365	LOC100144989 protein [Xen	165971120	AAI58328.1	19.87	22.19	6.49	10.88	5.2	14.2	4.98	3.04	0.89	0.72	3.23	0
1-2.c36683/1/1466	PREDICTED: serine/arginine	700322225	XP_00992747	0	0	10.47	0	0	0	0.78	0	0	36.59	87.1	0
2-3.c5145/8/2501	gsn protein [Xenopus (Silurar	159155320	AAI54876.1	54.71	60.57	61.87	52.66	79.49	53.66	40.7	58.41	54.27	6.57	0	25.28
2-3.c59275/1/2666	Unknown (protein for IMAG	197246230	AAI68800.1	3.94	0.77	3.73	3.54	2.4	1.67	0	4.1	3.03	0	0	0.51
1-2.c42493/226/16	ubiquinol-cytochrome c reduc	148222361	NP_00108668	583.01	199.71	311.83	317.52	449.59	234.67	271.7	282.38	246.57	90.41	18.57	83.28
1-2.c34068/1/1339	--	--	--	3.35	7.71	9.34	12.35	6.31	4.95	2.9	4.14	4.46	0.96	0.91	0.5
2-3.c45394/2/1931.	--	--	--	1.64	2.11	0.61	2.22	0	0.65	0	1.89	2.18	18.61	19.69	39.14
1-2.c12249/1/1519	--	--	--	18.28	4.68	23.81	11.17	17.65	7.79	59.52	0	35.98	202.92	297.02	195.36
2-3.c19255/1/2595	ribosomal protein S6 kinase,	39794417	AAH64239.1	0.87	7.89	0.12	0	2.81	5.04	2.33	1.42	0	0.01	0	0
1-2.c41582/2/1799	high mobility group protein 2	55742128	NP_00100676	0.7	3.53	1.21	1.56	0.52	2.33	1.42	0.45	0	0	0	0
2-3.c1422/76/2342	kinectin [Xenopus (Silurana)	194018594	NP_00112338	0.71	0	0	0	0	0	0	0	0	0	4.81	5.22

1-2.c48064/5/1310	actin, alpha skeletal muscle B	820145793	KKX13565.1	12.99	1.65	12.75	16.2	21.2	9.6	13.13	14.4	8.29	1.94	1.17	3.39
1-2.c11329/1/1537	uncharacterized protein LOC	147905311	NP_0010906(	0	0	0	0	0	0	2.29	2.96	0	13.41	0	26.68
2-3.c25435/5/2426	PREDICTED: Fanconi anem	301621609	XP_00294013	1.02	7.03	1.5	2.55	2.36	3.38	1.77	0.85	1.99	0.61	0	0
1-2.c36045/1/5902.	--	--	--	0	15.77	0	0	0	0	0	0	0	49.29	33.37	0
1-2.c29268/2/1598	--	--	--	3.29	5.01	2.42	0	1.74	3.13	0	0.8	1.26	32.14	50.09	22.67
1-2.c38662/1/1731.	--	--	--	1.4	0	0	0	0	0	0	0.83	0	5.41	3.88	3.89
2-3.c8132/1/2615	RecName: Full=Transitional	257051069	P23787.3	0	0.01	0	0.09	0	0	0	0	0	0	16.61	17.59
1-2.c38843/1/1295	--	--	--	4.41	0.57	0.72	2.16	3.59	1.82	15.07	4.04	3.97	45.4	68.66	52.23
1-2.c34363/1/1710	PREDICTED: glycine dehyd	512813230	XP_00293422	0	0	0	0	0	0	3.09	0.17	0.04	7.31	0	10.83
1-2.c50616/1/1305	serpin peptidase inhibitor, cla	147899187	NP_00108698	2.07	11.29	3.54	0	4.31	5.55	0	0	0	0	0	0
2-3.c12268/1/6515.	calsequestrin 1 (fast-twitch, s	148231736	NP_00108022	19.15	1.97	53.06	30.96	38.04	39.63	34.54	36.09	42.58	11.44	3.79	3.57
2-3.c16250/1/1939	--	--	--	0	0	0	0.01	0	0	0	2.66	0	5.88	0	8.2
2-3.c4368/1/2565	LOC398050 protein [Xenopu	126362056	AAI31885.1	5.72	3.85	10.75	5.75	5.9	1.32	1.75	2.41	6.07	0.01	0.37	0.78
2-3.c47583/1/2542	phospholipid-transporting AT	71896237	NP_00102556	0.35	1.04	1.11	1.11	0.51	0.91	6.77	0.89	0.31	10.99	13.11	30.02
1-2.c4810/2/1417	--	--	--	10.15	2.68	0	5.15	16.9	1.02	22.57	22.09	5.91	91.91	213.44	153.64
1-2.c2762/21/1410	--	--	--	43.35	43.76	91.72	60.06	118.74	361.23	76.43	51.82	128.64	1.17	1.81	5.38
1-2.c29299/1/6568.	translation elongation factor I	211907091	ACJ12080.1	50.1	0	0	2.93	0	108.77	195.65	97.81	170.45	531.52	929.28	423.82
1-2.c34897/1/1681	uncharacterized protein LOC	147906566	NP_00109117	0	0	0	0	0	0	0	0	0	8.55	11.34	0
1-2.c27548/1/1312	--	--	--	0	0	0	0	0	0	0	0	0	20.68	54.77	3.33
2-3.c54690/1/2116	carnitine O-acetyltransferase	73853812	NP_00102748	2.29	1.58	2.6	6.91	4.8	4.12	4.7	2.32	3.41	0.55	0.17	0.95
1-2.c39955/1/1222	--	--	--	117.21	132.71	279.97	190.63	322.71	1026.27	251.44	160.2	392.66	4.6	11.69	12.88
3-6.c5011/1/3331	cGMP-dependent 3',5'-cyclic	118404742	NP_00107260	2.18	2.83	1.05	0.59	0.57	1.02	0	1.26	0.07	0	0	0
3-6.c15513/1/2974	PREDICTED: transforming a	847151927	XP_01282315	7.94	4.53	15.44	11.79	12.61	4.07	6.46	11.99	12.72	0	5.3	0.29
2-3.c28740/1/2167	Na-K-2Cl cotransporter 1 [X	147904042	NP_00109133	1.44	1.66	0.31	0.8	1.18	0.64	3.11	1.4	0.55	19	15	25.37
2-3.c14168/4/2428	gnl3-prov protein, partial [Xe	38649400	AAH63220.1	29.31	18.22	20.6	14.11	10.82	21.42	15.1	15.02	12.41	2.28	1.81	3.31
1-2.c35673/1/1491	--	--	--	2.6	4.41	0	1.44	2.56	2.25	0	2.56	0.21	30.26	63.9	40.14
3-6.c7532/1/2819	aryl hydrocarbon receptor nu	154147739	NP_00109368	0.27	6.58	0.34	0.46	0.27	0.44	0.26	0.3	0.15	7.67	14.59	8.36
3-6.c8035/1/3000	PREDICTED: sarcalumenin i	641794149	XP_00816075	22.98	2.36	18.29	26.87	19.62	8.02	17.63	19.14	32.98	2.34	2.73	3.98
1-2.c27528/1/1411	--	--	--	3.31	5.38	0	2.8	1.73	3.9	3.44	4.91	3.63	57.43	44.09	64.06
1-2.c33876/2/1279	PREDICTED: nebulin isofor	847166510	XP_01282661	0	0	0	0	0	0	0	0	0	15.84	11.32	0
1-2.c25614/1/1758	--	--	--	0	0	0	0	0	0	0	0	0	2.97	7.82	1.56
2-3.c59623/1/2618	casein kinase I isoform alpha	820135221	KKX08385.1	0	2.11	0	1.86	1.81	0	0.5	0	0	0	0	0
2-3.c34956/1/2515.	PREDICTED: zinc finger pro	847170543	XP_01280875	0	1.93	0	1.49	0	0.86	0.05	0	0.27	19.7	27.26	11.26
1-2.c13291/1/1454	PREDICTED: keratin, type II	699641559	XP_00987834	39.25	31.79	30.49	22.86	78.83	25.95	72.18	39.08	30.17	0.49	0.96	2.43
2-3.c45713/1/2007	oxysterols receptor LXR-beta	118403952	NP_00107285	0	1.29	0.01	0.07	0	0.07	0	0	0	5.11	2.96	4.76
3-6.c16452/1/3721	NODAL modulator 3 precurs	189230238	NP_00112144	2.19	13.1	2.31	0	4.68	12.23	0	0	0.1	0	0	0
2-3.c35848/1/2139	--	--	--	10.88	3.54	31.85	19.42	20.42	19.57	20.41	20.06	19.69	4.1	1.99	4.08
2-3.c13579/1/7723.	calsequestrin 1 (fast-twitch, s	148231736	NP_00108022	7.43	0.82	29.86	19.62	31.74	20.91	16.93	18.5	27.35	0.71	0	7.69
1-2.c19610/1/1363	zygotic DNA replication licen	212286112	NP_00113103	0.42	15.27	1.45	0.35	3.2	3.14	0	0.68	0.89	0	0	0
1-2.c24202/1/1778	--	--	--	0.14	27.27	0.15	0.14	0.05	0.37	0	0	10.13	66.63	76.01	0.73
1-2.c35007/1/1540	PREDICTED: pyruvate dehy	512866108	XP_00293466	1.34	0	1.05	3.03	2.55	1.18	1.6	0.3	0.13	0.14	0	0
2-3.c19990/3/2040	PREDICTED: voltage-depen	823417697	XP_01241959	10.77	9.56	14.42	21.8	19.43	21.45	14.37	7.05	13.45	8.1	0	2.56
1-2.c2057/25/1807	desmin [Xenopus laevis]>gi|1	148224383	NP_00108017	117.82	69.13	113.95	49.29	100.36	99.55	67.33	82.95	64.7	21.82	20.86	7.92
1-2.c28418/3/1441	voltage-dependent anion-sele	148225606	NP_00108939	37.2	2.03	26.97	27	28.37	20.44	22.13	32.09	17.96	4.81	3.24	4.76
2-3.c17554/1/2470.	beta-enolase [Gallus gallus]>	46048765	NP_990450.1	1087.49	57.65	1958.02	2906.51	2334.43	655.75	2303.17	2159.92	3342.82	216.64	38.96	319.54
2-3.c6406/2/2258	PREDICTED: angiopoietin-r	847161289	XP_01282520	0.73	0	1.31	0	0	0.3	0	1.61	2.41	6.87	4.63	3.07
1-2.c49295/1/1348	--	--	--	1.22	1.67	0	2.07	1.57	2.43	0.12	0.62	0.4	0	0	0
1-2.c11669/1/1812.	--	--	--	19.26	35.42	14.69	13.78	15.27	27.09	4.16	12.07	19.14	0.77	0.91	1.6
2-3.c6683/1/2259.1	PREDICTED: LOW QUALIT	543714928	XP_00549874	9.85	2.97	12.47	9.42	6.87	6.37	0	3.65	0	0	3.14	0
2-3.c6736/1/2605	PREDICTED: collagen alpha	512859351	XP_00491692	4.93	19.13	10.92	6.99	8.69	7.85	2.04	3.8	5.59	0.83	1.43	1.36
2-3.c9365/6/2428	alpha 1 type I collagen [Rana	3242649	BAA29028.1	22.37	693.8	89.69	46.6	40.86	191.76	26.49	21.63	42.91	1.93	0	1.52
1-2.c7916/1/1691	PREDICTED: fumarate hydr	512842641	XP_00293586	30.8	47.52	54.2	45.33	25.08	33.64	23.68	48.55	0	4.93	4.85	8.59
1-2.c16868/1/1351	--	--	--	0	0.55	0	3.15	0	0	9.1	2.24	2.51	54.19	58.02	12.52
2-3.c35520/1/2048.	fast troponin I [Rana catesbei	58397794	AAW73073.1	3547.28	981.64	7323.49	4942.62	5737.54	6030.37	5585.69	6829.51	4374.82	1257.94	841.84	1256.12
2-3.c40893/1/1959	--	--	--	23.83	4.29	19.75	14.97	12.59	7.63	7.31	4.91	18.78	0.4	3.87	1.7
2-3.c16341/1/2504	eukaryotic elongation factor 2	187607479	NP_00112014	3.79	2.66	2.12	8.02	7.4	2.5	4.65	0.97	1.76	0.37	0.3	1.65
2-3.c20378/1/3092	PREDICTED: glucosylceram	557017449	XP_00600913	0	2.88	0	6.39	0	2.42	9.72	0	0.88	61.02	98.48	33.83
1-2.c8698/2/1160	calreticulin precursor [Xenop	148230491	NP_00108076	0	0	0	0	21.12	59.12	0	0	0	0	0	0

1-2.c24235/2/1712	--	--	--	0	0	0	0	0	0	0	0	2.85	2.3	3.7	2.96
1-2.c32790/1/1501	phosphatidylinositol 3,4,5-tris	183986707	NP_00111694	0.95	0	0	0	0	0	0.86	0	0	5.12	3.3	1.68
2-3.c6000/5/2118	thrombospondin-4 precursor		118404528	NP_00107267	8.08	10.79	23.68	8.73	4.17	22.72	8	10.78	12.9	0	0	0.94
1-2.c34593/1/1637	PREDICTED: ATP-binding c	699653275	XP_00989689	0	0	1.58	1.55	0	0	1.21	0	1.88	22.52	32.67	7.65
2-3.c14094/1/2005	PREDICTED: pyridine nucle	512851997	XP_00293648	0.85	2.88	1.41	1.25	1.57	0.95	0.49	0.4	0.47	0	0	0
2-3.c3842/4/1963	cAMP-specific 3',5'-cyclic ph	118403894	NP_00107286	4.94	5.31	1.82	3.03	4.64	0	0	0.25	0	0	0	0
1-2.c47233/3/1591	PREDICTED: rho GTPase-ac	512862460	XP_00491733	0	0	0	0	0	0	0	0	0	8.5	7.49	0
2-3.c20719/5/2123	PREDICTED: non-syndromic	847139923	XP_01282102	232.98	4.89	29.83	116.18	172.16	0.86	19.63	87.05	90.07	0.16	0	2.3
2-3.c13194/2/2160	PREDICTED: talin-1 isoform	768398998	XP_01159535	0	0	0	0	0	0	0.83	0	1.07	45.42	0	19.75
1-2.c25728/1/1353	PREDICTED: elongation fac	573878959	XP_0066277(	67.81	130.93	124.92	31.63	14.72	20.49	31.98	14.38	21.33	11.25	0	0
2-3.c14172/11/2182	YTH domain family protein 1	431894607	ELK04407.1	0	0.55	0	0	0.51	0.97	12.23	0	2.08	11.11	16.35	9.85
2-3.c40760/1/2202.	pyruvate kinase PKM isoform	118405080	NP_00107253	216.44	0	180.79	342.75	280.5	0	112.85	266.29	346.97	8.13	0	0
2-3.c61595/4/2205	PREDICTED: protein PRRC	512859823	XP_00294134	4.85	41.12	17.82	0.04	0.02	0	0	0.63	0	0.17	23.87	16.32
3-6.c10887/2/2945	eukaryotic translation initiatio	148232020	NP_00108075	0	0.95	0	0.28	0.92	1.21	0.47	0.47	0	0	0	0
2-3.c34444/1/2620.:	--	--	--	110.28	9.78	24.26	144.98	54.26	29.65	253.43	97.74	152.5	1162.86	1884.88	1090.96
3-6.c18459/1/3360	PREDICTED: chloride chanr	874464599	XP_01295201	6.22	0.08	3.47	2.74	6.44	4.77	2.24	3.72	6.76	0	0	1.69
1-2.c44663/1/1360	--	--	--	4.61	4.94	15.47	11.63	8.31	35.65	11.67	7.68	9.15	1.31	0.18	0.49
1-2.c27329/2/1774	agmatine ureohydrolase (agm	147900416	NP_00108921	28.07	14.17	44.88	42.36	34.37	29.83	30.84	30.24	37.58	4.39	6.84	8.62
1-2.c14017/1/1749	PREDICTED: schwannomin	847126596	XP_0128181(	1.31	0	0	0	0	0	1.83	0	0	4.84	3.26	0.67
2-3.c24654/2/2447	--	--	--	25.74	11.61	46.16	37.24	31.86	48.1	31.76	28.42	37.66	7.13	5.35	9.41
2-3.c54289/1/2248.	PREDICTED: G protein-cou	765112974	XP_01147901	0	0.24	0	0	4.06	3.98	0	0	0.63	0	0	0
2-3.c62672/1046/24	calsequestrin 1 (fast-twitch, s	148231736	NP_00108022	20.58	1.85	60.87	20.76	40.59	22.37	14.42	46.69	30.65	7.05	4.23	4.91
3-6.c2699/13/3594	PREDICTED: polyadenylate-	696972430	XP_00955615	31	22.51	8.72	10.69	14.24	9.22	1.58	9.71	7.17	2.5	1.4	2.25
1-2.c7395/2/1275	--	--	--	0	0	0	1.19	0	1.66	2.51	0	0	31.88	25.37	14.18
3-6.c10939/1/4150.	sarcospan [Xenopus laevis]>g	147902306	NP_00108856	19.96	18.63	27.25	17.99	24.76	35.94	12.39	14.43	20.84	3.36	2.49	6.48
2-3.c39066/1/2144	PREDICTED: nebulin-relatec	699588075	XP_00986535	3.95	1.87	8.33	4.17	4.55	4.71	1.24	2.82	3.33	0.34	0.56	0.56
1-2.c1797/58/1597	claudin-3 [Xenopus (Silurana	54020932	NP_0010057(	1.95	0.44	0	0	0.43	0.52	0	1.83	0	12	8.35	32.44
1-2.c5202/1/5612.3	platelet-derived growth factor	71896169	NP_00102558	34.04	6.76	28.96	8.64	8.14	0	0.09	13.01	0	0	0	0
1-2.c52245/4/1435	PREDICTED: endoplasmin is	847110350	XP_01281465	0	0	0	0	0	0	0	2.52	4.44	14.38	24.98	0
2-3.c58866/1/1993.	--	--	--	16.58	35.44	20.45	13.34	16.25	31.28	8.31	14.16	14.19	3.87	2.02	2.37
2-3.c19813/1/2100	PREDICTED: interleukin enl	847169995	XP_01280854	7.94	2.95	0	0	8.94	5.02	0	0	3.21	0	0	0
3-6.c6658/1/6035.1	PREDICTED: sarcoplasmic/e	847174969	XP_01281038	2659.1	263.16	15.4	2720.88	3603.4	2169.71	2111.72	2983.87	18	388.45	327.78	977.86
1-2.c31258/1/1390	PREDICTED: CCR4-NOT tr	557006273	XP_00600468	5.06	7.4	1.26	4.87	0	1.4	1.04	0.27	0	48.33	50.87	32.34
1-2.c48828/1/1729.	--	--	--	17.41	4.84	11.94	10.44	12.62	4.27	39.11	2.63	16.15	159.26	230.87	120.2
2-3.c13721/2/2489	RecName: Full=Voltage-depe	46576355	O57483.1	5.5	0.8	17.04	6.81	14.08	9.13	6.59	9.87	11.41	0.84	0.69	3.09
1-2.c17529/2/1762	polypyrimidine tract-binding	118404352	NP_00107247	4.37	16.92	4.44	2.62	5.43	12.72	4.81	3.16	4.5	0	0	0.94
1-2.c35432/1/1298	uncharacterized protein LOC	187608717	NP_0011204(	0	0	0	0	0	0	0	0	0	9.52	9.24	0
2-3.c44036/2/2082	OTTXETP00000002159 [Xe	89272520	CAJ83768.1	0.17	7.16	0.12	0	2.18	2.65	0	0	0	0	0	0
2-3.c2189/9/2415.1	PREDICTED: protein FAM1	512881655	XP_00294317	0.36	0	2.64	1.77	3.86	1.33	0.56	0	0	0	0	0
1-2.c35935/1/1404	--	--	--	2.02	20.38	4.05	2.56	0.74	2.36	0.09	1.44	0.95	0	0	0
2-3.c56473/1/2099.	3-hydroxyisobutyrate dehydro	71896099	NP_0010256(	9.78	8.15	16.94	10.65	17.61	14.19	7.99	11.48	7.87	0	0.69	1.93
3-6.c3226/2/3320	ubiquinol-cytochrome-c redu	147900400	NP_00108188	4.94	7.38	3.33	9.33	12.04	5.73	3.45	2.49	6.93	1.31	0.84	2.41
1-2.c38168/1/10757	--	--	--	16.09	0	2.45	1.3	6.76	5.33	19.44	7.81	13.44	128.86	154.25	41.61
1-2.c4709/2/1650	--	--	--	4.9	4.83	0	0.25	0	0.65	2.19	0	0.92	13.34	8.19	8.49
2-3.c20770/1/2039	LOC443692 protein, partial [	49119610	AAH73734.1	3.33	0	0.19	0	2.44	2.58	2.29	2.27	1.14	0	0	0
1-2.c12280/2/1606	PREDICTED: citrate synthas	617649484	XP_00753343	26.45	85.77	73.59	126.5	73.79	58.23	141.04	82.81	18.15	6.37	4.28	36.53
1-2.c38321/1/1328.	aspartyl/asparaginyl beta-hyd	62751990	NP_00101573	3.83	3.7	8.47	1.24	8.09	21.72	4.19	5.71	7.04	0	0	0
3-6.c9746/1/3536.1	--	--	--	5.9	13.01	0.11	0	0	0	0	4.63	4.6	0	7.54	8.49
3-6.c2766/6/3194	PREDICTED: semaphorin-3I	395539080	XP_0037715(	2.12	28.57	3.07	1.55	6.13	5.21	0.66	1.02	3.09	0.68	0.24	0.76
1-2.c12992/2/1651	activating transcription factor	147902692	NP_00108699	27.4	44.65	32.41	34.88	33.14	39.92	22.98	14.95	31.51	6.59	3.24	9.43
1-2.c28729/1/1766	hedgehog acyltransferase-like	147901798	NP_00107949	89.3	11.98	77.1	101.65	112.42	53.41	32.58	62.68	54.78	13.72	23.03	19.24
1-2.c32609/3/1985.	PREDICTED: keratin, type I	512874187	XP_00293908	837.47	1165.36	1476.59	894.62	1314.54	75.1	1211.48	839.72	803.52	37.59	3.28	6.75
1-2.c20912/1/1738	provirus ancestral Env polypr	808862307	KKF13889.1	31.92	72.28	5.29	17.34	19.17	11.17	102.18	12.55	28.56	390.46	279.19	173.1
1-2.c50838/1/1446	--	--	--	0	7.93	3.11	5.65	7.14	0	0	0.04	5.94	0	0	0.04
2-3.c52817/1/5822.:	--	--	--	0	25.79	0	0.08	0	0	0	0	23.33	31.19	22.49	0
1-2.c44470/1/1816	PREDICTED: polyadenylate-	847099772	XP_01281235	37.02	18.26	26.85	31.65	21.65	54.25	32.99	44.29	27.92	7.49	10.44	3.1
1-2.c26092/2/1884	uncharacterized protein LOC	182765469	NP_00111683	0	2.48	0	0	2.38	2.8	0	0	0	0	0	0
3-6.c18863/7/2944.:	eukaryotic translation initiatio	148232020	NP_00108075	77.31	133.31	38.54	48.59	56.48	93.34	47.47	41.55	45.16	6.16	0.17	27.09

1-2.c22005/2/1452	PREDICTED: interferon regu	847127915	XP_01281852	4.34	0	0.11	1.98	0.01	1.8	7.31	2.39	5.53	22.31	36.63	25.5
2-3.c5438/1/2150.2	--	--	--	0	55.1	2.67	0	18.12	16.01	0	0	0	0	0	0
1-2.c8924/1/1459	PREDICTED: adenosine kina	874462866	XP_01295145	6.35	14.12	14.62	8.18	5.89	7.57	2.11	1.8	2.47	0	0	2.04
2-3.c26102/1/2052.	--	--	--	4	0.61	2.9	2.81	6.43	4.47	1.33	1.59	14.16	0.29	0.95	0.14
2-3.c41114/1/2024.:	--	--	--	0	0	0	0	0	0	1.79	0	1.9	3.66	22.3	8.34
2-3.c56643/1/2338.	reverse transcriptase, partial [	347227	AAA49027.1	0.83	45.13	0	0	1.28	3.66	4.23	0.77	0.05	44.41	69.13	19.34
2-3.c777/43/2538	succinate dehydrogenase [ubi	148237169	NP_00108347	44.19	9.38	53.92	65.56	71.12	31.23	20.28	22.06	70.84	20.6	4.55	9.35
3-6.c4687/1/6482	--	--	--	1.46	52.84	3.73	1.82	3.14	6.13	0.83	1.81	2.77	0.26	0.42	0.64
2-3.c22148/1/1883.	--	--	--	0	0	0	0	0	0	1.57	0	0	10.73	7.73	0
2-3.c42309/1/2269.	PREDICTED: zinc transporte	301618652	XP_00293871	0	0	0.38	0	2.66	2.65	0	0	0.6	0	0	0
3-6.c5530/1/2861	PREDICTED: myosin-4 [Xe	847156920	XP_01282446	73.67	32.18	38.63	15.45	26.29	4.97	18.7	35.12	13.14	0.56	2.24	0.39
1-2.c42918/2/1803	PREDICTED: sorting nexin-:	704154961	XP_0101301(	0	0	0	0	0	0	1.26	0	0	6.47	5.84	0
2-3.c56018/1/2335	microtubule-associated protei	148232096	NP_00108054	1.53	0	0.03	1.67	1.67	0	1.99	6.24	2.01	35.26	54.3	14.64
2-3.c53963/1/2408.	PREDICTED: rho GTPase-ac	512868524	XP_00491809	0	0	0	0	0	0	0	0	0	7.2	7.78	0
2-3.c36466/1/2194	uncharacterized protein LOC	73853872	NP_00102751	22.07	11.75	9.88	8.6	12.21	3.45	3.59	5.62	5.04	0	4.22	0
2-3.c4691/1/2170	LOC398524 protein [Xenopu	169642411	AAI60682.1	0	2.86	0	2.38	0	2.28	1.56	0	0	0	0	0
2-3.c58988/1/2501.:	--	--	--	5.2	1.04	1.36	1.08	1.65	1.25	15.95	3.06	0.77	33.55	53.42	18.23
1-2.c11573/2/1220	--	--	--	0	4.48	0	2.99	1.62	1.44	3.93	0.77	2.27	51.04	36.97	40.06
1-2.c25420/2/1700	tetranectin precursor [Xenopu	58332698	NP_00101142	5.8	3.17	9.41	4.61	14.31	5.58	7.62	0	6.08	0.5	0.17	0.85
1-2.c3082/5/1609	nucleosome assembly protein	118404866	NP_0010729(	11.44	32.34	7.26	5.37	3.18	26.72	3.86	4.22	3.85	0	0	0
1-2.c50141/1/1411	ubiquinol-cytochrome c redu	147902934	NP_00107971	0	0	0	0	0	0	0	0	0	50.65	143.48	0
3-6.c5030/1/4113	PREDICTED: serine/threonin	591351354	XP_00707012	1.81	0.43	1.4	1.68	3.11	0	1.29	0.86	0	0	0	0
1-2.c3127/1/1441	--	--	--	0.31	1.99	3.1	0	0	0	0	0	0	34.03	85.45	0
2-3.c23026/1/2322	--	--	--	0.7	0.66	0.31	0.63	0.93	1.48	4.86	0.5	0	0	0	0
2-3.c63590/1/2160	PREDICTED: beta-enolase [	637381761	XP_00812393	2.52	0	7.11	9.61	6.81	2.59	5.9	4.23	14.7	1.34	0.77	0.63
1-2.c45861/1/1508	PREDICTED: protein CDV3	847134819	XP_01281997	5.98	14.81	3.52	2.91	4.43	5.15	1.99	3.92	4.14	0.86	0	0
1-2.c44134/1/1563.	tissue factor pathway inhibito	62751741	NP_00101576	0	14.29	2.38	0	6.05	4.66	0	0	0	0	0	0
1-2.c29985/1/1566	PREDICTED: tropomyosin a	847143250	XP_01282187	1.07	53.08	0	0	3.06	7.8	4.71	0	0	82.53	62.22	46.54
1-2.c2586/6/1298	actin, alpha cardiac muscle 2	45361557	NP_989355.1	2.77	0	9.71	14.12	4.84	7.32	7.42	9.73	3.42	1.54	0.2	1.27
2-3.c32812/1/2039	far upstream element-binding	148232337	NP_00109083	0.94	15.67	3.56	8.02	4.52	5.51	0	0	9.48	2.41	0	0
3-6.c10528/1/3030	PREDICTED: cytoplasmic F.	512821479	XP_00491186	2.74	10.69	2.64	2.29	5.3	3.39	1.96	3.26	0	0	0.51	0.83
1-2.c24551/1/1340	PREDICTED: ubiquitin carb	847119228	XP_01281688	1.78	5.03	1.06	0	0	1.85	4.62	2.04	1.51	20.5	22.87	6.47
2-3.c55982/2/2655	--	--	--	0	0	40.01	9.66	42.45	14.76	16.73	28.36	13.2	1.62	0	0
1-2.c42392/3/1731	PREDICTED: aspartate--tRN	847163066	XP_01282572	0	0	0	0	0	0	0	0	0	0	6.23	5.95
2-3.c59034/1/2311.	--	--	--	0.67	0	0	0	0	0	2.77	0	1.93	13.47	0	9.69
1-2.c38137/1/1569	high density lipoprotein bindi	160420251	NP_00108016	78.44	93.35	114.21	74.99	93.96	68.54	60.9	72.64	80.12	9.4	3.57	31.55
3-6.c11062/1/2776	--	--	--	4.36	131.09	9.09	4.86	4.46	15.47	2.95	3.45	7.8	0.44	0.19	0.74
1-2.c11764/1/1710	PREDICTED: filamin-A-inte	301613504	XP_00293624	8.5	10.21	3.95	1.74	3.35	9.61	1.17	0.59	6.95	0	0.24	0.1
2-3.c12144/2/2509	PREDICTED: protein prune	847161780	XP_01282533	4.04	6.94	4.76	7.05	4.05	11.72	2.04	3.27	5.48	1.72	0.64	1.04
1-2.c4839/8/1710	PREDICTED: protein PFC07	556961274	XP_00599098	33.34	29.64	90.31	57.22	124.33	378.14	68.19	53.54	148.03	1.24	5.61	2.86
2-3.c25673/1/1889	--	--	--	2.16	2.36	4.11	1.09	3.34	3.46	0.88	0.47	4.8	0	0.49	0
2-3.c3027/2/2604	transmembrane protein 65 [X	187607455	NP_0011203(	4.29	0	0.77	2.97	1.96	0	1.96	1.88	4.92	0	0	0
2-3.c6634/8/2301	calsequestrin 1 (fast-twitch, s	148231736	NP_00108022	5.9	0.58	10.28	8.2	10.1	8.65	6.06	8.34	9	0.52	1.2	2.43
1-2.c32951/1/1200.:	--	--	--	9.25	8.26	14.83	43.49	18.95	17.06	24.75	9.23	24.03	1.44	3.04	4.41
1-2.c8423/1/1407	--	--	--	0	0	0	0	0	0	0	0	0	12.19	8.58	0
2-3.c3280/1/2453	eukaryotic translation initiatic	156717262	NP_00109617	49.78	69.06	29.08	24.16	26.1	25.63	19.72	19.59	15.01	3.04	0	10.25
2-3.c37041/1/1972	PREDICTED: constitutive cc	564265499	XP_00627158	3.93	22.56	4.22	4.29	8.47	11.1	3.54	4.28	4.18	0	0.69	2.42
3-6.c6910/3/4151	paxillin [Xenopus (Silurana)	213983203	NP_0011355(	2.75	6.65	4.55	2.57	4.57	4.49	1.72	1.69	1.38	1.29	0.19	0.29
3-6.c8001/2/2753	Unknown (protein for IMAG	197246230	AAI68800.1	9.34	0	10.09	4.07	4.45	1.19	2.95	3.87	12.52	0	1.35	0
1-2.c1895/41/1826	hedgehog acyltransferase-like	147901798	NP_00107949	90.89	0	17.08	91.54	104.67	48.1	28.27	15.73	39.68	8.86	17.25	24.7
1-2.c13325/2/1764.	tetranectin precursor [Xenopu	58332698	NP_00101142	13.76	0	8.28	8.55	6.12	1.72	4.52	9.81	14.43	1.31	0	0
2-3.c45406/2/2534	PREDICTED: ATP-binding	512830490	XP_00491305	2.7	0.88	0.47	2.19	1.15	0.27	0.68	0.9	0.5	24.93	22.83	24.32
1-2.c17270/1/1413.	Three prime repair exonuclea	226372920	ACO52085.1	20.33	0.69	4.56	1.34	2.26	1.59	67.65	8.63	1.69	65.47	40.31	32.68
2-3.c42924/1/1876	primase, polypeptide 1, 49kD	89267400	CAJ82378.1	0.29	9.33	1.03	0.88	2.79	3.77	0.22	0.44	1.32	0	0	0.29
3-6.c6086/1/4223	angiotensin-converting enzyn	183986763	NP_00111688	0.52	0.62	0.31	0.56	0.53	0.45	0.07	0.53	0.51	0	0	0
2-3.c59102/1/2341	--	--	--	0.84	4.2	0.38	0.74	1.28	2.89	0.63	0.62	0	0.18	0	0
3-6.c15508/1/6944.	RecName: Full=Sodium/pota	30923213	P30714.2	22.11	22.82	24.26	12.17	29.38	11.82	4.82	2.38	8.67	2.94	0.51	3.65
2-3.c24498/3/2053	PREDICTED: ubiquitin-like	736263084	XP_0107903(	0.04	0	0	0	0	0	1.21	0	0.49	8.81	5.78	0

2-3.c7423/2/2264.1	ferritin H [Bufo gargarizans]	89515094	ABD75379.1	3584.05	640.21	1203.45	3661.76	2630.3	360.13	5154.6	2371.11	3497.09	32708.02	57229.96	25622.39
2-3.c15845/1/1839.	--	--	--	28.59	84.19	49.28	46.51	33.18	53.14	29.57	24.79	41.89	9.24	4.1	10.15
2-3.c23963/1/7119.:	Plaa protein [Xenopus laevis]	68534804	AAH98975.1	0.69	1.12	0	1.38	0.53	2.14	8.17	7.51	3.31	42.6	23.91	19.84
2-3.c33369/1/1927	PREDICTED: flotillin-2 isofo	847099593	XP_01281229	0	0	2.66	0.02	0	0	0	0	6.62	12.32	1.03	6.2
2-3.c5782/1/2440	cytochrome c oxidase subunit	146149044	YP_00116544	381.05	1271.43	505.59	940.11	154.92	1081.7	248.48	11.09	516.15	66.32	38.01	57.66
2-3.c60447/1/2136	mitochondrial import recepto	156717970	NP_00109652	16.34	15.28	9.6	5.12	15.24	17.96	7.95	14.95	7.84	0	1.26	4.69
2-3.c35209/1/2283.	--	--	--	14.75	7.83	8.45	8.84	9.13	3.19	10.53	4.31	12.98	0.36	1.79	0.42
2-3.c5979/1/2279	NADH dehydrogenase subun	146149047	YP_00116545	11.88	32.9	22.04	13.76	19.47	19.94	11.45	16.47	18.09	1.76	1.35	6.07
1-2.c7693/5/1490.1	--	--	--	1105.12	1554.67	1861.27	1259.44	1440.65	107.25	1857.63	1219.12	1274.41	76.12	14.55	10.99
2-3.c58473/84/2353	ATP-binding cassette sub-fan	676264017	KFO20588.1	25.71	57.33	16.95	15.36	18.16	43.41	19.02	14.93	11.55	4.31	0	8.54
3-6.c13633/1/7136	LIM domain-binding protein	45360839	NP_989095.1	3.3	4.82	12.68	16.11	17.9	23.38	19.22	8.12	18.92	2.93	1.67	6.05
1-2.c23432/2/1509	PREDICTED: cyclin-depend	847153765	XP_01282364	28.54	0	9.58	0	2.87	3.8	3.83	8.48	11.59	0	0	0
2-3.c41062/1/1984	--	--	--	2.43	1.14	5.28	1.91	4.29	7.32	1.19	0.95	1.15	0.18	0.25	0.89
2-3.c10632/2/1897	pyruvate kinase PKM isoforn	118405080	NP_00107253	43.28	0	47.41	58.94	50.03	24.91	72.52	65.13	55.84	0	0	23.89
1-2.c18813/9/1438	replication factor C (activator	148233376	NP_00108067	4.6	18.01	1.25	1	6.62	2.27	0.59	1.89	0	0	0	0
2-3.c5103/1/2515	PREDICTED: MBT domain-	641798037	XP_00816220	2.95	1.56	0.78	1.04	1.24	0.36	2.2	0	2.01	0	0	0
2-3.c1183/85/2296	pescadillo homolog [Xenopus	148236909	NP_00108055	15.69	29.59	7.36	3.7	6.76	19.16	4.64	7.68	5.54	0.1	0.58	0.35
2-3.c53621/1/2597	PREDICTED: aminopeptidas	847168456	XP_01280803	0	0	0.81	0	0	0	0	1.16	0.74	3.12	0	3.8
1-2.c13203/1/1389.	PREDICTED: 40S ribosomal	109104395	XP_00109708	102.3	38.69	72.15	63.74	58.16	117.49	82.58	73.99	17.68	9.92	9.46	19.25
2-3.c14999/1/2012	histone-lysine N-methyltrans	148229543	NP_00108865	0.86	0	0	0	0	0.02	1.45	0.39	0.94	2.31	2.98	1.71
2-3.c1881/16/1855	PREDICTED: T-complex pro	530627264	XP_00530314	57.64	79.84	40.07	28.47	44.37	104.79	40.54	51.22	27.22	8.3	3.57	7.81
2-3.c35445/3/2106.:	PREDICTED: E3 ubiquitin/IS	847172880	XP_01280964	59.75	2.48	45.42	60.15	58.3	34.5	31.58	44.98	59.13	9.5	8.54	10.08
2-3.c25574/1/2351	PREDICTED: nuclear pore co	525010339	XP_00505279	0	0	0	0	0	0	1.62	0	2.55	4.94	9.86	0.49
2-3.c59173/1/2212.	PREDICTED: carnosine synt	847171322	XP_00491926	4.95	4.16	6.81	3.69	5.99	11.68	2.99	3.38	4.85	1.03	0.8	1.03
3-6.c10153/1/3016	PREDICTED: myosin-3 [Xen	847172863	XP_01280963	2.45	8.62	3.33	3.32	0.67	11.84	1.49	2.75	1.69	0	0	0
2-3.c58330/29/2046	PREDICTED: cell division co	301604583	XP_00293192	0.46	8.72	0.57	0.41	1.23	2.95	0.08	0.37	0.77	0.06	0	0
2-3.c40516/1/2183	--	--	--	4.68	0	6.4	2.48	5.46	8.92	3.22	0	0	0.17	0.25	1.52
1-2.c42098/4/1367	V-type proton ATPase 16 kD	45360509	NP_988893.1	0	0	0	0	0	0	0	0	4.77	0	85.48	35.81
1-2.c38888/1/1502.	--	--	--	2.1	24.01	2.01	5.13	3.97	26.44	5.72	3.13	3.64	156.35	221.72	218.47
1-2.c11100/2/1313	PREDICTED: double-strande	847135111	XP_01282007	24.64	34.71	25.19	12.17	25.55	28.62	10.38	7.55	21.56	5.14	0	5.54
1-2.c14922/2/1369	selenide, water dikinase 2 [Xe	373432635	NP_00107289	0	0	0	0	0	0	0	0	0	11.15	8.18	0
1-2.c52182/4/1402	GDP-mannose 4,6-dehydratas	147899928	NP_00108035	0	7.88	0	0	0	0	2.51	0	0	0	7.84	7.77
2-3.c3561/2/2310	PREDICTED: coiled-coil don	573895397	XP_00663544	0	1.5	0	0	0	0	0.06	0.12	2.1	2.89	0.89	2.57
1-2.c11602/1/1382	RNA polymerase-associated	147900849	NP_00108528	0.12	22.79	0	0	0	0	0	5.67	3.92	10.79	15.61	0
1-2.c3637/1/1620.1	PREDICTED: adenylate kina	847099206	XP_01281215	31.3	31.11	34.87	37.32	29.46	43.52	19.64	29.7	26.1	5.66	2.35	10.8
2-3.c18320/15/2610	DNA replication licensing fac	148232250	NP_00108146	6.14	63.38	4.05	3.47	8.42	13.32	1.29	2.41	5.13	1.01	0	2.65
2-3.c42231/1/2616	PREDICTED: uncharacterize	847139520	XP_01282096	0	0.67	0	0	0	0	0	0	2.67	2.55	1.2	1.39
2-3.c57515/3/2301	endothelin converting enzyme	132424622	ABO33468.1	0	9.5	1.92	0	0	3.9	7.61	5	4.99	11.18	19.78	35.42
2-3.c8864/1/2049	PREDICTED: transmembran	512852461	XP_00293601	0.17	0	0	0	0	0.97	2.61	0	2.34	10.62	9	6.72
3-6.c19091/8/3352	myosin, heavy chain 4, skelet	47575800	NP_00100124	0.92	0.53	19.25	12.84	9.92	0.09	19.56	6.9	9.14	0.1	0.13	0.1
1-2.c6523/1/1653	3-hydroxyisobutyrate dehydro	147898679	NP_00108475	10.99	5.71	7.42	11.79	7.92	2.83	10.52	9.06	6.4	0.55	1.99	0.28
2-3.c27177/2/2165.	--	--	--	8.06	13.03	6.75	7.41	6.01	0	2.64	3.81	2.82	0	0	0
1-2.c46586/2/1608	PREDICTED: dimethyladeno	512842699	XP_00491468	2.43	1.77	1.08	2.06	4.09	2.06	0.38	0.28	2	0.42	0	0
1-2.c29298/1/1769.	deoxynucleotidyltransferase,	148227738	NP_00108539	0	10.57	1.93	0	0	0	3.56	0	0	15.58	10.14	0
1-2.c43301/1/1666	Mark2-prov protein [Xenopus	27694575	AAH43730.1	0	0	0	0	0	0	0	0	0	5.91	0	5.44
1-2.c47084/1/1454	--	--	--	0	4.38	0	0.19	0	0	0	0.17	0.42	2.61	10.06	4.52
2-3.c47041/1/2434	PREDICTED: collagen alpha	512859351	XP_00491692	1.79	4.82	3.04	2.91	3.57	2.72	1.6	1.49	2.1	0.46	0.18	0.29
2-3.c54978/1/2225	Mimitin, mitochondrial precu	226372810	ACO52030.1	0	0	0	0.55	1.64	0.94	1.04	0	0	0	0	0
3-6.c3318/4/3339	H(+)/Cl(-) exchange transpor	113931518	NP_00103921	0.63	0.98	0	1.19	1.66	0	0	0	0	0	0	0
1-2.c49523/1/1370	hypothetical protein T265_11	684410742	XP_00917617	38.4	14.79	1.52	2.16	2.27	5.34	44.01	24.03	3.19	54.21	63.9	64.9
3-6.c2748/6/2872.1	transmembrane BAX inhibito	147899637	NP_00108928	3.3	0	3.32	3.45	5.54	1.36	1.2	3.95	0	0	0	0.67
2-3.c41116/1/2724.	uncharacterized protein LOC	147905311	NP_00109060	0.13	0	0.36	1.06	0.51	1.08	2.35	0.16	0.47	20.36	17.85	36.36
2-3.c40646/2/2051	--	--	--	0.46	3.43	0.16	0	0	0	2.85	0	0	0.13	3.34	3.36
1-2.c44486/1/1589	--	--	--	2.48	5.88	5.62	2.92	2.8	7.46	2.2	2.66	3.36	0.33	0.22	0.45
3-6.c4119/2/3033.1	RecName: Full=Heat shock p	338817950	P30946.2	0	0	0	0	0	0	4.57	0.07	0.97	19.41	14.56	0
1-2.c32116/2/1616	beta tubulin, partial [Chionod	10242188	AAG15329.1	0	0	0.09	0	0	0	0	0	2.33	10.69	33.62	3.13
2-3.c35184/1/2025	PREDICTED: LOW QUALI	641766329	XP_00816801	0	0	0	0	0	0	0	2.14	0	8	14.9	0.13
2-3.c56290/2/2399	--	--	--	1.92	0	0	0	0	0	0	0	0	0	3.41	3.9

1-2.c1065/49/1485	PREDICTED: voltage-depen	530564480	XP_00527881	1980.39	1043.21	1596.03	1422.44	1606.6	1050.15	1138.03	1188.2	1366.66	322.74	205.69	363.86
1-2.c19102/2/1399	--	--	--	9.32	0	7.36	6.92	0.56	1.87	10.65	6.88	5.14	48.68	76.11	52.82
1-2.c27694/1/4507	PREDICTED: cytoplasmic dy	637327354	XP_0081139	0	1.6	0.43	0	0	0	0	0.87	2.25	9.47	3.4	1.26
2-3.c59258/1/2379.	PREDICTED: glutathione S-	558227591	XP_00613781	20.93	65.94	32.29	26.48	16.4	66.52	30.58	27.35	31.08	4.22	1.98	2.63
1-2.c31183/1/1459	--	--	--	2.32	0.7	1.34	2.61	1.82	0.98	0.96	2.1	3.23	42.02	36.8	32.44
2-3.c8644/1/2308.2	--	--	--	111.73	62.7	101	113.46	76.18	3.46	9.78	88.85	59.21	1.04	0	1.62
2-3.c9334/8/2414	RNA-binding protein 28 [Xe	194018610	NP_00112339	1.19	0.47	1.05	0.98	0.98	3.15	1.01	0.58	0.81	0.2	0	0
1-2.c43254/1/1302	PREDICTED: immunoglobul	847130950	XP_01281911	1.75	4.17	0.56	4.17	3.17	6.13	0.82	4.23	4.6	0.18	0.19	0.39
1-2.c48648/1/1246	PREDICTED: enoyl-CoA hy	432903775	XP_00407722	20.11	60.15	21.46	27.43	34.95	78.55	24.65	29.56	25.82	10.12	9.23	7.53
2-3.c23376/1/2651	synaptojanin 2 binding protei	148232060	NP_00108525	0	0	1.22	0	0	0	0	0	0	3.55	3.5	0
2-3.c52467/1/2029	PREDICTED: tropomyosin-2	755867989	XP_0112915	1.34	0.59	0	0	0.4	0.12	0	0.69	0.42	9.85	8.06	2.73
1-2.c19814/6/1816	PREDICTED: monocarboxyl	704293877	XP_01016065	29.99	0.82	28.7	82.7	87.2	9.59	27.34	35.66	61.29	3.58	3.33	2.49
1-2.c4650/1/1476	RecName: Full=Cyclin-A2 [	1345741	P47827.1	0.81	37.45	0.53	0.66	2.49	7.26	0	1.17	1.79	0	0	0
1-2.c40238/1/1387	F-box/LRR-repeat protein 15	148225152	NP_00107974	0	0	0	0	0	0	0	0	0	3.95	5.42	2.68
2-3.c61900/1/2215	Saf-A protein [Xenopus laevi	54311369	AAH84742.1	0	0	0	0	6.36	3.6	0	0.17	0	0	0	0
1-2.c19681/3/1296	LOC494840 protein, partial [	52789300	AAH83011.1	0	33.79	0	0.12	0	0	26.78	0	0	71	164.75	0
3-6.c9299/1/2983.1	P2Y purinoceptor 1 [Xenopus	156717364	NP_00109622	1.79	1.96	3.23	0.76	0.94	3.87	0	0.9	1.71	0	0	0
2-3.c14228/1/2155	PREDICTED: adenylyl cycla	301606235	XP_00293272	0	5.32	0	0	0	0	9.93	0	0	0	3.89	5.4
3-6.c10412/1/4439.	PREDICTED: collagen alpha	602636099	XP_0074249	39.92	1112.56	205.53	125.4	163.09	558.41	72.27	62.08	135.14	9.13	5.51	10.94
1-2.c8877/1/1726	collagen, type 1, alpha 2 prec	147898763	NP_00108072	0	0	0	0	0	0	0	0	0	0	4.82	6.24
1-2.c11992/9/1486	PREDICTED: 3-hydroxyisob	591350996	XP_00706995	4.83	17.63	5.94	4.63	5.85	14.05	3.02	4.53	5.18	1.32	0.48	1.03
1-2.c6536/2/1545	--	--	--	21.76	1.03	19.1	0.09	0.13	0.16	0.22	0.22	27.68	36.44	0.17	37.22
2-3.c9845/1/2538.1	PREDICTED: tyrosine-protei	847138365	XP_0049153	8.21	11.96	10.1	6.36	7.42	4.26	4.08	9.15	6.74	0	0	1.38
2-3.c4307/1/2287	--	--	--	3.83	0.17	0.32	1.6	1.38	0.41	4.98	0.58	0.84	24.41	19.96	23.98
2-3.c12470/2/2420.	--	--	--	0.62	0	1.08	0	0	0	0	0	0.09	7.04	3.72	1.32
1-2.c42521/373/13	secreted protein acidic and ric	42407294	BAD10858.1	1.17	22.75	3.9	4.33	3.62	11.45	1.83	2.03	3.02	0	0	1.65
1-2.c47470/3/1551	PREDICTED: cytoplasmic dy	637327354	XP_0081139	14.01	22.21	5.35	2.72	0	5.34	4.73	0.1	4.62	45.36	47.22	46.48
1-2.c7903/1/1636	PREDICTED: LOW QUALIT	847103725	XP_0128131	1.3	2.83	2.73	1.68	2.96	1.83	1.51	2.09	2.36	0	0.19	0
2-3.c42323/3/2552	--	--	--	9.49	3.2	5.33	11.24	16.39	4.78	7.49	0.73	3.6	2.33	0	2.92
1-2.c28458/1/1700	--	--	--	1.63	11.66	0.63	2.85	1.67	6.61	2.2	2.21	2.47	55.99	75.18	56.08
1-2.c40764/1/1392	PREDICTED: ankyrin repeat	512839176	XP_00293509	38.16	1.29	6.19	17.44	19.78	0.84	10.41	30.37	20.77	0.99	0	0
2-3.c40892/1/2313	PREDICTED: laminin subun	512830498	XP_00293314	0	0	0.78	3.63	2.19	0	0	0	0	0	0	0
1-2.c25890/1/1686	PREDICTED: calcium/calmo	847142006	XP_01282144	8.46	5.05	4.74	10.97	9.53	13.18	5.94	2.51	5.2	0	0	4.65
2-3.c36681/1/2213	MAP/microtubule affinity-reg	148232710	NP_00108512	0	0	0	0.57	1.99	6.41	0	3.89	0	0	0	0
1-2.c41962/4/1387	actin, alpha cardiac muscle 2	45361557	NP_989355.1	8.62	0.91	8.49	10.69	14.77	14.49	12.48	7.88	10.69	2.28	1.29	2.54
2-3.c7138/1/2274	calsequestrin 1 (fast-twitch, s	148231736	NP_00108022	50.2	7.97	89.97	73.67	89.46	70.89	65.13	80.48	83.8	18.18	7.86	23
2-3.c29535/1/1907	Histone deacetylase 1, partial	676771369	KFP00125.1	0	0	0.77	0	0	0	1.66	0	0.25	0.44	2.44	5.22
2-3.c8666/1/1939	PREDICTED: uridine-cytidir	611978749	XP_0074755	0.75	0	7.29	3.91	2.52	0	0.31	2.6	0	0	0	0
2-3.c30901/1/2306.	phospholipid transfer protein	349732136	NP_0010877	21.61	32.03	19.18	11.6	4.89	17.25	10.04	16.36	14.12	177.85	269.6	119.33
2-3.c13579/1/7723.	calsequestrin-1 precursor [Xe	45360503	NP_988894.1	1301.36	143.51	2256.45	1654.59	2049.32	1228.35	1029.6	1723.47	1608.55	326.96	185.64	554.3
2-3.c2427/7/2322	fragile X mental retardation p	147898943	NP_0010899	4.88	19.71	1.8	0.76	4.19	1.19	0.96	1.28	0	0	0	0
1-2.c38857/1/1391	casein kinase 2, alpha prime p	148235727	NP_0010851	0	0.2	0	0	0	1.33	5.52	0	3.68	13.84	20.34	7.54
1-2.c17036/1/1918	PREDICTED: vacuolar fusio	847163331	XP_0128258	0	0	0	0	0	0	0	0	0	11	9.82	0
2-3.c13579/1/7723.	calsequestrin 1 (fast-twitch, s	148231736	NP_00108022	653.62	68.95	673.89	328.46	991.73	1086.48	752.92	963.27	836.25	181.75	125.54	206.89
2-3.c55975/2/2013	uncharacterized protein LOC	148236097	NP_0010894	0.17	1.6	0	0	0	0	0	0	4.23	8.27	5.36	0
1-2.c40488/1/1684	PREDICTED: transmembran	634827293	XP_00795314	0	0	0	5.98	0	0	5.81	13.25	5.19	40.09	46.34	29.02
1-2.c46373/1/1406	PREDICTED: DNA polymer	847117617	XP_01281632	1.32	4.56	0.98	1.02	1.87	2.43	0	0	0	0	0	0
2-3.c25039/8/2243	novel protein containing thre	89269825	CAJ81625.1	0	39.22	0	0	1.77	2.63	0	0	0	0	0	0
2-3.c51481/29/2171	LOC100038258 protein [Xen	166796565	AAI58918.1	1.46	26.98	6.34	6.46	10.44	4.71	3.74	6.31	9.7	0.41	2.28	0.98
3-6.c13921/1/3620	LIM domain-binding protein	45360839	NP_989095.1	4.79	1.41	17.11	15.31	16.88	15.96	19.32	9.95	19.65	2.91	1.15	5.23
1-2.c10270/19/146	RAD9 checkpoint clamp com	147905155	NP_0010826	13.34	26.1	11.21	9.97	12.17	16.37	11.5	12.68	7.06	2.81	3.2	0.58
2-3.c24441/1/2410	PREDICTED: cyclin-depend	847146277	XP_0128221	0	1.02	0	1.66	0.6	0.41	0	2.63	0.99	0	0	0
3-6.c8866/1/2769	MGC84250 protein [Xenopus	148225296	NP_0010879	5.97	0.47	7.21	15.61	7.88	5.91	9.18	8.98	4.51	3.01	1.35	1.23
3-6.c5830/1/3433.2	--	--	--	1.23	14.2	0.79	3.92	1.09	5.11	14.28	1.66	2.17	41.07	62.32	88.52
1-2.c20364/1/1361	PREDICTED: male-specific	512874083	XP_0029402	0	0.12	2.49	0	0	0	0	4.61	3.96	7.21	0	11.53
1-2.c42490/5/1626	DBIRD complex subunit ZN	349585177	NP_00101608	0	0	0	0	0	0	7.95	0	4.92	9.93	17.7	0
2-3.c21624/1/6482.	PREDICTED: beta-enolase-li	573878367	XP_00662741	1504.65	92.24	2789.49	4552.27	3214.19	701.47	1770.31	2150.31	3310.83	169.96	46.71	285.75
2-3.c30755/1/1949	PREDICTED: protein archea	669281586	XP_0086333	1	0	0	0	0	0.62	5.15	0	0.1	13.02	3.76	5.69

2-3.c63202/3/2163	folylpolyglutamate synthase,	156717746	NP_00109641	2.7	12.1	1.7	3.73	3.71	10.08	0.95	1.95	0.46	0.63	0.73	0.95
3-6.c4386/1/2742	--	--	--	2.13	11.15	0.69	3.74	2.05	0.72	20.44	2.45	3.89	28.41	50.46	37.05
3-6.c10120/1/3260	PREDICTED: eukaryotic tran	847153589	XP_01282358	0.49	0.4	0.49	0.31	0.54	1.13	0.17	0.77	0.56	0	0	0
3-6.c13582/1/2762	PREDICTED: tetratricopepti	512843038	XP_0029345(	1.56	2.17	0.65	0.76	0.65	3.51	0.46	0.73	0.66	0	0	0
1-2.c3351/1/1682	novel protein containing thre	89269825	CAJ81625.1	0	0	0	0	0	0	0	0	0	9.8	17.97	0
1-2.c27670/1/1245	ER lumen protein-retaining re	58332334	NP_00101104	0	0	0	0	0	0	0	0	0	16.6	32.49	0
2-3.c21229/1/2070	PREDICTED: glycogen phos	426369041	XP_0040515(	0.1	3.51	13.59	11.96	93.58	51.31	18.92	112.91	67.46	0	5.72	0
1-2.c43880/1/3449.1	PREDICTED: elongation fac	573878959	XP_0066277(	23.24	0	0	35.91	0	38.9	233.13	49.81	142.05	359.73	419.36	360.65
3-6.c13579/1/11992	--	--	--	108.08	0	4.2	88.24	63.36	2.77	108.85	80.2	70.82	785.93	1168.66	670.46
1-2.c17033/2/1311	PREDICTED: protein FAM3	612057466	XP_00750695	0	0	0	0	0	0	0	0	0	8.76	11.79	0
1-2.c44228/1/1880	PREDICTED: notchless prote	530616974	XP_00529822	4.12	11.24	1.42	2.85	3.7	10.24	3.8	1.53	1.78	0.57	0.19	0.55
1-2.c25597/1/1369	PREDICTED: 14-3-3 protein	821027291	XP_01235685	0	0	0	0	0	0	0	0	0	18.46	39.82	0
2-3.c44477/1/2652	PREDICTED: myosin-4 [Xer	847156920	XP_01282446	1.06	0.01	24.66	15.04	11.14	0.08	27.54	12.75	13.06	0.19	0	0.15
2-3.c27237/1/1911	PREDICTED: 14-3-3 protein	821027291	XP_01235685	55.9	23.71	56.62	74.93	59.29	70.18	54.88	40.08	38.56	0	16.02	26.37
2-3.c45408/3/2351	PREDICTED: beta-enolase-li	573878367	XP_00662741	0.26	0	0	1.55	1.04	0.14	0.17	0	1.37	0	0	0
1-2.c40367/1/1528	synaptophysin-like protein 2	54606873	NP_00100611	66.54	69.71	142.7	82.68	129.54	397.24	99.08	114.64	118.14	10.3	2.13	9.05
2-3.c48949/1/2025	phosphoethanolamine methyl	351720746	NP_00109627	0.95	1.27	1.16	0	1.95	2.08	0	0.37	0	0	0	0
1-2.c42795/38/1549	ATP synthase subunit alpha,	71896075	NP_00102561	74.43	72.95	135.83	105	178.88	157	121.45	71.51	161.05	31.14	46.67	24.18
1-2.c40827/1/1753	PREDICTED: ADP/ATP tran	829867054	XP_01264039	6.95	1.58	5.75	8.3	5.37	7.39	4.5	2.51	3.46	0.6	2.07	0.49
2-3.c16307/1/2245	PREDICTED: integral memb	543742020	XP_00551187	1.66	0	0	0.18	0	0	2.41	0	0	45.12	86.07	0
1-2.c34319/1/1302	PREDICTED: uncharacterize	658888975	XP_00842733	6.61	12.04	1.48	5.46	3.48	2.11	18.01	2.86	4.9	103.73	67.82	54.02
2-3.c49278/1/2455.	--	--	--	0	44.55	0	2.75	1.96	4.16	0	0	0	56.9	77.15	34.09
3-6.c15160/1/2801	PREDICTED: cTAGE family	847155129	XP_01282405	3.49	0	1.43	2.46	2	2.08	3.76	0.65	1.4	0.59	0	0
1-2.c41255/1/1571	PREDICTED: collagen alpha	641767075	XP_00816827	6.52	26.23	9.38	12.36	6.91	17.55	7.46	7.05	11.05	3.09	1.18	1.59
2-3.c63596/1/2512	proteasome activator complex	156717318	NP_00109620	0.49	2.66	0.25	0.83	0.96	0.8	0	0	0	0	0	0
2-3.c7709/4/2461	RecName: Full=Myb-related	6226654	P52551.2	1.12	2.72	0.71	0.35	1.16	1.16	0.54	0.88	0.38	0	0	0
1-2.c49329/1/1659.	--	--	--	0.18	4.23	0.01	3.76	3.48	0	22.09	3.84	0	59.95	50.17	48.54
1-2.c7569/2/1696	--	--	--	3.29	4.46	1.35	1.07	3.03	0.62	1.26	1.02	2.43	18.5	33.06	42.52
1-2.c46152/1/1510	N-terminal kinase-like protein	148225212	NP_00108530	3.5	6.34	0	0	0	0	2.36	0.2	0.5	1.6	1.98	5.96
1-2.c19087/1/6532.	LOC100125139 protein [Xen	140832681	AAI35415.1	15.36	9.82	9.73	3.98	3.4	8.01	3.46	16.84	0	0	0	0
1-2.c10850/11/1728	LOC100144989 protein [Xen	165971120	AAI58328.1	20.1	25.34	10.24	11.14	8.88	12.93	9.28	6.48	7	1.48	2.06	1.93
1-2.c17145/1/1938.	PREDICTED: beta-enolase [.	637381761	XP_00812393	569.1	39.38	778.55	1460.02	1042.33	298.87	976.88	965.32	1198.82	73.98	10.34	170.13
3-6.c18211/1/2784.	PREDICTED: collagen alpha	556973045	XP_00599461	2.01	37.68	10.3	4.4	1.15	6.89	1.94	2.42	4.9	0	0.14	0.17
1-2.c34277/1/3900.	PREDICTED: choline-phosp	675418748	XP_00892276	0.42	0.4	1.05	5.91	9.09	5.63	0.42	4.57	0.71	0.47	0.56	0.9
1-2.c6546/2/1254.1	3-oxo-5-beta-steroid 4-dehyd	71896073	NP_00102560	0	0.27	0	0.09	4.79	8.5	0	0	3.97	0	0	0
1-2.c1857/19/1873	SET and MYND domain con	148225404	NP_00108546	0	0	0	0	21.38	84.75	0	0	39.02	0	0	0
1-2.c7258/17/1550	succinyl-CoA ligase [ADP-fo	45361311	NP_989233.1	10.4	0	8.3	13.58	9.17	16.36	3.5	8.96	14.06	0	1.33	4.52
2-3.c21934/1/2107	PREDICTED: myosin-bindin	768914292	XP_01160075	1.82	1.26	4.64	3.55	2.44	2.2	1.51	4.51	2.92	0.35	0.41	0
2-3.c2236/16/2034	ras-related GTP-binding prote	820152711	KKX16856.1	0	4.7	0.32	1.2	3.49	0.5	1.2	0.85	0	0	0	0
1-2.c20127/3/1727	periostin precursor [Xenopus	163915007	NP_00110637	16.11	0	39.41	16.83	11.85	6.7	8.52	11.41	26.07	0	3.92	2.52
2-3.c6306/1/2092	--	--	--	2.1	1.63	0	0.29	0	0.68	0.7	2.24	0.67	7.44	10.21	7.7
1-2.c28375/1/1750	PREDICTED: diacylglycerol	301610039	XP_00293457	0	4.61	3.4	2.34	2.65	1.76	1.98	2.15	0	0.28	0	0
2-3.c19452/1/2530	PREDICTED: LOW QUALIT	641766329	XP_00816801	14.06	21.59	35.91	32.82	18.29	13.79	14.06	8.67	17.2	1.54	2.72	8.44
2-3.c49645/1/1859.	PREDICTED: DENN domain	847169838	XP_01280847	0.17	0	0	0.95	0.16	0.2	5.54	0	0	52.66	64.58	278.44
1-2.c5975/1/3903.3	PREDICTED: NEDD8-conju	847163368	XP_01282581	6.31	11.31	4.97	2.41	7.5	11.82	0.07	0	5.29	0	0	0
2-3.c56289/2/2079	calsequestrin 1 (fast-twitch, s	148231736	NP_00108022	9.51	0.82	17.09	13.1	17.04	12.31	13.21	16.84	12.4	2.97	0.66	4.01
1-2.c48250/1/1363	Sec23 homolog B (S. cerevisi	89266855	CAJ83841.1	2.41	0	3.97	3.14	2.69	0	16.66	5.32	1.04	43.91	41.66	32.91
1-2.c7004/2/1535	--	--	--	0	0	0	0	0	0	0	0	0.39	9.88	9.66	0
1-2.c13020/1/1405	ER lumen protein-retaining re	45361615	NP_989381.1	0	0	0	1.94	0	0	0	2.99	0	14.87	23.64	13.06
2-3.c53509/1/6356.:	--	--	--	0	0	2.07	0	0	0	0	0	0	15.58	27.32	0
3-6.c2992/3/3198	nicotinamide nucleotide trans	148231869	NP_00108770	38.19	22.59	29.45	28.99	30.49	23.04	25.25	32.6	31.42	7.72	3.52	6.5
2-3.c3061/1/2339.1	PREDICTED: uncharacterize	847164282	XP_01282609	2.12	0.21	5.16	3.27	4.46	8.79	3.2	5.03	8.92	0.69	0.64	0.33
1-2.c4358/1/1830	--	--	--	0.94	2.23	0	0	0	0	0	0.77	0.58	2.73	3.31	1.35
1-2.c27458/1/1705	polypeptide N-acetylgalactosa	147900163	NP_00108341	1.63	1.27	1.26	0.87	0	0.13	7.46	0.17	1.02	8.45	8.62	12.95
1-2.c33801/1/1212	PREDICTED: hypothetical p	297293110	XP_00280419	2.15	0	0	0	0	0	0.49	0	4.17	12.79	22.65	0
1-2.c14191/1/1604	--	--	--	0	0	0	0	0	0	0	0.45	0	5.04	0	4.99
2-3.c22823/1/2025	PREDICTED: LOW QUALIT	803125841	XP_00401312	0	2.57	1.09	0	0	0	0.41	0	0	3.72	1.06	1.69
2-3.c48271/1/2321	PREDICTED: myosin-7B [X	512871888	XP_00293293	2.38	5.38	5.48	3.6	5.39	34.71	3.24	2.14	4.25	0	0	0

1-2.c51741/5/1474	PREDICTED: alpha-actinin-	558140034	XP_00611831	538.55	147.12	689.32	728.87	582.17	687.91	577.83	618.25	600.7	157.41	106.79	182.66
3-6.c18560/2/2925	myosin, heavy chain 4, skelet	47575800	NP_00100124	0.77	0	1.55	2.35	1.47	0	1.93	2.29	0.52	0	0	0
1-2.c46143/1/1833	--	--	--	13.82	2.26	10.75	48.24	34.34	10.9	13.92	17.39	27.07	2.37	3.51	5.23
2-3.c40999/1/1986.	MGC84105 protein precursor	147904058	NP_00108618	0	8.9	2.34	0.95	1.36	3.83	0.68	0	1.03	0	0	0
2-3.c42247/1/2475	four and a half LIM domains	55742067	NP_00100670	4.53	18.62	6.03	5.53	6.25	10.7	2.38	4.84	1.88	0	0	3.18
1-2.c11213/3/1769	probable threonine--tRNA lig	156717366	NP_00109622	0	28.16	0.08	0	0	0	0	2.75	9.16	5.09	4.87	0
2-3.c9371/1/2437	PREDICTED: SUMO-activat	706145739	XP_01020496	0	0	0	0	0	0	0	0	0	3.81	1.14	0.87
2-3.c54417/1/2126	PREDICTED: myosin-4 [Xer	847156920	XP_01282446	0.19	1.1	129.08	56.63	17.93	0.72	183.28	0	0.13	0	0.05	0
2-3.c20472/1/2390	heterogeneous nuclear ribonu	147904138	NP_00108421	0	0	0.92	0	0	0	0	0	0	8.81	0	5.16
2-3.c27428/3/2377	PREDICTED: laminin subun	512844752	XP_00293635	26.68	28.09	22.29	16.72	21.44	44.04	18.52	24.47	23.09	5.16	5.99	5.81
3-6.c17069/1/3326	Hermansky-Pudlak syndrome	148226823	NP_00108241	13.23	5.78	11.34	8.38	12.21	6.34	4.12	8.19	11.15	1.36	1.18	2.57
3-6.c11132/1/2948.	PREDICTED: PDZ and LIM	847087387	XP_01281714	3.75	0.37	14.44	23.36	13.86	9.2	4.94	10.39	10.49	1.34	0.15	5.67
2-3.c15131/1/2112	PREDICTED: fibroblast grov	558127197	XP_00611551	0	0	0	0	0	0	0	0	0	6.34	1.42	1.62
2-3.c19420/1/2212	zinc finger protein 326 [Xenc	213624144	AAI70704.1	0	0	0	0	0	0	0	0	0	6.13	4.1	0
2-3.c23967/2/2044.	--	--	--	2.33	0.36	4.4	3.57	2.47	0.68	2.43	0	7.15	0	0.34	0
2-3.c44973/22/2595	LOC443574 protein, partial [	48734648	AAH72270.1	46.76	4.13	46.8	53.05	60.35	24.84	34.17	37.79	50.12	11.6	5.05	13.71
3-6.c14239/1/1545.	PREDICTED: glutamic acid-	780154032	XP_01168211	5.69	8.2	3.37	5.3	5.09	11.78	19.02	4.04	5.12	97.54	145.37	110.35
2-3.c41298/2/2036	--	--	--	3.47	8.89	3.66	3.22	2.73	5.09	9.41	4.5	3.99	59.57	68.23	51.98
1-2.c26587/28/1348	secreted protein acidic and ric	42407294	BAD10858.1	64.39	741.97	221.8	136.91	173.11	382.62	162.44	91.49	108.06	43.17	14.31	83.7
1-2.c39308/1/1549	--	--	--	0.1	11.02	0.01	0	3.62	2.77	0	2.88	0.18	0	0	0
1-2.c44177/1/1133	aspartate aminotransferase 2		148232563	NP_00108025	0.48	67.95	0.43	157.82	135.12	84.38	159.68	114.8	185.22	0	100.79	0
2-3.c40433/1/2650	--	--	--	14	2.6	0.52	0.34	0.03	0	15.57	1.42	9.18	1.19	15.75	20.92
3-6.c9503/1/2768.1	--	--	--	1.43	4.81	0	0.59	0.66	2.39	0	0	1.05	22.81	28.38	28.24
1-2.c9579/1/1347.1	PREDICTED: mucin-4-like i	847172448	XP_01280947	46.62	12.84	43.28	14.35	28.58	7.01	11.63	26.36	10.98	2.42	0.26	1.32
2-3.c41066/1/2498	PREDICTED: dynein heavy	632937914	XP_00790151	0.28	0	0.29	0	1.41	1.4	0	0	0.02	0	0	0
1-2.c45523/1/1441	REST corepressor 1 [Xenopu	148233554	NP_00108214	0	2.72	0	0	0	0.09	1.14	0	0	5.37	3.53	5.05
1-2.c32703/1/5049.	larval type I keratin [Rana cat	134140868	ABO61147.1	14.32	4	4.7	5.64	12.61	3.14	9.33	2.4	7.86	0.87	0	0
1-2.c6334/2/1277	PREDICTED: dnaJ homolog	847168000	XP_01282709	1.04	0.19	3.16	1.94	6.98	1.26	0	0.96	0	0	0	0
2-3.c36097/1/2475	Putative transposase [Dicentr	317419774	CBN81810.1	19.16	9.96	30.7	22.82	28.36	53.93	22.84	23.02	32.44	9.46	6.52	6.56
2-3.c7528/3/2575	ena/VASP-like protein [Xenc	147905674	NP_00108902	4.05	1.68	4.25	4.56	6.22	2.24	4.56	2.19	7.14	0.59	0.43	0.85
1-2.c12585/4/1522	PREDICTED: nicotinamide r	542206422	XP_00547665	35.21	25.14	22.8	6.46	29.39	13.45	2.32	20.76	42.69	0.51	2.31	0.27
1-2.c13547/1/1435	PREDICTED: nebulin isofor	847166512	XP_01282661	3.1	0	0	5.13	12.32	11.31	6.16	4.68	0	2.5	1.58	0
1-2.c37916/3/1339	gamma-sarcoglycan [Xenopu	118404980	NP_00107290	0	5.74	0	0	0	0	0	0.85	0	4.45	4.81	1.3
2-3.c32226/1/2467	transmembrane 9 superfamily	147898405	NP_00108858	0	0	0	0	7.79	3.86	0	0.07	0	0	0	0
2-3.c22641/2/2177	fatty acyl-CoA reductase 1 [X	148229551	NP_00107959	1.07	4.52	0.46	1.88	1.64	1.32	1.21	2.74	0.88	0	0.21	0
1-2.c49216/1/1273	RecName: Full=mRNA cap g	82114931	Q9I8S2.1	0	0	0	0	0	0	0	0	0	2.03	3.69	5.24
3-6.c15679/1/3575	PREDICTED: alpha-actinin-	556954562	XP_00598892	0.39	0	0.98	0.77	0.49	0.38	0.2	0.51	0.73	0	0	0
2-3.c44158/2/2112	Uba1a protein [Xenopus laev	28958137	AAH47256.1	0	0	0	0	0	0	0	0	0	4.97	3.78	0
1-2.c42210/2/1570	Novel Ubiquitin carboxyl-ter	89272870	CAJ81966.1	1.38	8.34	1.26	4.5	0.66	0.58	4.97	0.96	3.12	32.7	53.19	24.07
3-6.c17218/10/3324	PREDICTED: titin-like [Allig	564226854	XP_00625789	67.8	50.64	142.39	86.31	131.91	222.96	108.7	107.73	217.36	22.83	6.37	61.76
1-2.c14317/1/1307	PREDICTED: casein kinase I	847117966	XP_01281643	0.46	2.63	0	0	0	0	1.46	0.5	1.1	7.6	24.66	3.05
2-3.c56448/1/1995	PREDICTED: NADH-ubiqui	697482752	XP_00967305	104.31	24.27	99.72	91.72	62.33	24.13	55.4	80.59	83.92	0	3.45	19.76
1-2.c9252/2/1326	--	--	--	9.42	4.6	1.18	12.32	0.06	0.68	24.77	10.45	6.33	90.29	84.49	52.44
1-2.c14190/1/1477.	--	--	--	30.31	15.2	9.95	16.19	1.86	9.95	55.96	7.32	31.1	244.25	167.29	103.57
2-3.c34850/1/1970.	PREDICTED: meprin A subu	542223736	XP_00545132	0.87	0.89	0	0	0	0	0	0	0	4.41	5.96	0.81
3-6.c9499/1/3485	ryanodine receptor alpha isof	538246	BAA04646.1	73.66	31.62	145.06	79.84	109.27	140.38	57.65	83.73	86.18	16.09	27.87	30.98
1-2.c30360/1/1757	--	--	--	2.35	2.84	2.6	0	0	0	0	0	1.89	3.45	1.34	2.73
1-2.c3552/3/1500	glutamyl-prolyl-tRNA synthe	189217812	NP_00112134	0	0	0	0	0	0	0	0	0	0.06	22.17	11.68
2-3.c56691/1/2471	PREDICTED: nebulin isofor	847166512	XP_01282661	0.35	0.14	0	1.29	0.43	0.79	0.88	0.99	0	0	0	0
1-2.c5014/2/1358	PREDICTED: collagen alpha	637250524	XP_00811284	44.53	231.28	44.06	45.34	40.67	116.66	29.96	39.09	50.33	19.26	10.96	8.3
2-3.c49008/1/2174.	Na-K-2Cl cotransporter 1 [X	147904042	NP_00109133	0.17	0	0	0	0	0	0	0	0	1.59	2.43	2.97
2-3.c56843/1/1977	PREDICTED: HIRA-interact	512880191	XP_00294081	0.63	4.22	0.36	0	1.65	2.15	0	0.2	0.53	0	0	0
1-2.c28773/1/1648	--	--	--	8.52	5.67	2.3	3.53	3.47	2.88	10.95	4.97	3.39	44.89	84.59	42.64
1-2.c34351/1/1306	uncharacterized protein LOC	147902936	NP_00108858	1.29	2.33	0	0	0	0.14	8.71	3.58	0.65	7.69	7.77	0.83
1-2.c45466/1/1326	--	--	--	0	0	0	0	0	0	0	0.78	0	8.44	6.69	0
3-6.c6106/1/7096	PREDICTED: transmembran	847095053	XP_01281107	4.59	2.38	1.55	1.03	3.57	1.37	1.88	3.39	2.44	0	0.06	0.25
1-2.c29347/1/2721.	larval type I keratin [Rana cat	134140868	ABO61147.1	3093.57	497.45	1232.53	2384.93	2108.81	554.74	2548.23	1752.34	1689.95	241.89	384.73	174.34
2-3.c16695/1/2583	RecName: Full=Sarcoplasmi	9789732	Q92105.1	0.93	0.34	3.83	3.42	2.68	1.63	2.4	3.5	2.48	0	0	0.69

1-2.c27882/2/1701	WD repeat and SOCS box-co	45361625	NP_989387.1	7.48	20.32	12.97	4.06	12.7	13.2	9.44	3.92	6.15	0	0	4.21
2-3.c56455/1/2014.	PREDICTED: Kruppel-like f	363744516	XP_0036430(	4.7	3.6	0.7	7.39	5.02	0.83	98.58	4.83	4.8	79.14	72.24	94.13
1-2.c34254/1/1421	--	--	--	96.58	11.6	183.53	120.98	156.92	150.47	113.9	132.58	148.12	36.05	20.42	38.16
3-6.c18923/8/3235	PREDICTED: DNA ligase 1	641783051	XP_00817393	0.57	6.35	0.31	0.31	1.69	0.96	0.36	0.24	0.7	0	0	0.08
2-3.c29142/1/2065	WD repeat-containing protein	55742525	NP_00100678	8.95	13.25	6.47	0	0	0	0	3.49	0	5.86	0	4.16
3-6.c14138/2/4879	PREDICTED: myosin-4 [Xe	847156920	XP_0128244(	0	0	0.65	0.61	0.79	0	0.64	0.49	0.81	0	0	0
2-3.c26791/1/2238	--	--	--	1.74	0	0	1.58	0	1.6	0.79	0	0	0	0	0
3-6.c7744/1/4048	PREDICTED: laminin subun	512844752	XP_00293635	0.63	0.94	1.79	0	1.24	2.51	0	0.71	2.96	0	0	0
2-3.c8534/1/2725.1	hypothetical protein CAPTEI	443682880	ELT87315.1	1.8	41.04	0.06	0	1.63	3.05	0.4	0	2.01	45.48	55.83	15.78
1-2.c43312/2/1802	--	--	--	11.82	32.99	54.72	41.84	41.35	130.67	71.22	46.87	63.25	6.89	3.84	9.48
2-3.c40890/1/2553.	clusterin preproprotein [Bufo	393717589	AFN21430.1	19.93	0.6	4.18	20.97	0.44	0	28.62	13.83	8.37	161.75	109.91	84.87
1-2.c36805/1/1598	--	--	--	3.76	18.17	11.43	9.17	4.04	13.02	8.01	4.88	15.91	0.57	2.41	1.07
2-3.c49926/1/2414.	PREDICTED: complement c	512820694	XP_0029368(	0	18.87	0	0	13.21	30.86	7.47	3.83	0	0	0	0
2-3.c58936/1/2126	PREDICTED: ran-binding pr	543278705	XP_00542741	0.62	0	0.32	0	0	0.11	1.14	1.06	0	4.61	3.62	1.36
3-6.c18497/1/4128	splicing factor 3B subunit 1 [	147905376	NP_00108415	2.57	0	1	0	0.74	0.78	0.54	1.95	2.35	0	0	0
1-2.c46666/1/1244	cathepsin D-like precursor [X	218847782	NP_00113637	3.45	0	0	0	0	0	0	0	2.82	10.69	18.04	0
2-3.c54007/1/2369.	--	--	--	4.18	0	0	3.46	0	0.57	16.75	0	5.16	26.09	22.43	57.9
2-3.c42433/1/2065	PREDICTED: laminin subun	512844752	XP_00293635	10.63	17.1	8.34	4.92	4.83	11.8	4.35	6.14	5.25	1.03	0.05	1.91
3-6.c3874/1/3278	PREDICTED: ATP-depender	795120083	XP_01183217	0.42	5.18	0.78	0.64	0	1.11	1.85	0.12	0.5	13.04	15.4	4.55
1-2.c34250/1/1402	hydroxyacid oxidase 2 (long	148234656	NP_0010861(	7.21	0.76	8.5	8.63	4.57	1.7	5.8	5.68	4.4	0	0	0.7
2-3.c14844/1/2111	Kruppel-like factor 11 [Xeno	148222922	NP_00108601	22.76	8.68	1.82	5.66	1.6	0.94	19.69	33.1	12.88	20.21	93.26	90.34
2-3.c36164/2/1964	PREDICTED: uveal autoanti	512827341	XP_00491264	3.28	2.06	6.38	8.24	4.67	5.13	1.99	3.34	4.56	1.54	0	0.98
2-3.c36837/1/2221.	--	--	--	5.14	10.13	3.8	1.96	3.58	6.03	1.19	1.33	4.96	0	0	0
2-3.c19300/1/2390.	--	--	--	6.83	12.27	1.11	2.44	2.68	9.46	3.09	0.59	4.52	0	0	0.41
1-2.c43607/1/1622	electron transfer flavoprotein-	54020771	NP_00100563	6.44	8.9	0	0.19	0	0	0	0.1	14.02	0.29	14.82	20.67
3-6.c16427/1/3493	PREDICTED: laminin subun	512844752	XP_00293635	6.77	3.47	6.07	1.6	4.98	3.78	0.11	5.87	3.47	0.23	0	1.17
1-2.c12109/2/1319	PREDICTED: T-complex pro	700396110	XP_00993867	1.58	30.27	0	5.82	0	0	44	8.06	12.76	39.51	48.44	25.18
1-2.c39934/1/1859	--	--	--	0.14	0.72	0.87	0.92	0.43	2.21	0	0.47	0	0	0	0
2-3.c1509/25/2579	PREDICTED: methylmalony	512845229	XP_0029336(	22.49	22.24	19.61	23.85	22.11	14.79	14.79	12.36	21.41	3.82	3.24	5.88
2-3.c11789/7/2510	autocrine motility factor rece	147901912	NP_00108315	7.47	0	1.51	2.05	3.96	0	0	3.71	1.53	0	0	0
2-3.c4729/1/2425	PREDICTED: probable glob	847092406	XP_0128080(	0.45	11.56	1.61	1.08	4.66	2.86	0.41	2.47	4.04	0	0	0.78
2-3.c62044/11/2308	PREDICTED: myosin-bindin	564233250	XP_0062609	0.33	3.17	0	28.69	9.05	0	40.62	8.35	60.65	0	0	0.01
2-3.c13759/1/6896.	PREDICTED: serine/arginine	556960768	XP_00599082	0	0	0	0	0	0	5.97	0	0	62.78	16.67	8.95
2-3.c16675/12/216	aryl hydrocarbon receptor nu	156717516	NP_00109629	2.03	14.28	0.93	2.1	1.53	4.01	2.26	0.84	2.29	30.66	38.13	51.47
3-6.c8168/1/2699	PREDICTED: sarcalumenin	861486921	XP_01292099	6.92	1.35	6.57	6.74	6.87	3.5	9.93	7	11.33	0.58	0.85	1.46
1-2.c50517/1/1681	SET and MYND domain con	148225404	NP_0010854(	0	0	0	0	1.92	2.05	0	0	0.04	0	0	0
2-3.c49598/1/2045	Casp9-A protein [Xenopus la	213623754	AAI70178.1	0	0	0	0	0	1.55	0.21	0	0	7.64	15.41	17.59
3-6.c3325/8/3377	PREDICTED: exportin-2 iso	847167837	XP_0128270(	4.25	22.37	6.37	3.73	6.96	7.89	3.94	3.64	3.77	0.66	0.58	1.96
1-2.c25797/3/1313	MGC52527 protein [Xenopus	28838469	AAH47954.1	14.88	3.28	3.56	12.64	11.96	4.42	66.81	8.25	6.25	121.79	164.76	173.57
1-2.c44247/1/1329	PREDICTED: retinal dehydr	348572972	XP_0034722(	0	0	0	0	0	0	0	0	0	30.74	13.61	0
1-2.c4672/1/1548	periostin precursor [Xenopus	163915007	NP_00110637	2.24	1.53	1.12	1.45	0.87	2.13	0.24	0.24	1.63	0	0	0
2-3.c26877/1/1989	PREDICTED: myomegalin-l	573893936	XP_00663471	1.44	1.02	3.43	1.12	2.26	5.77	1.01	2.18	1.27	0.13	0	0.25
1-2.c24317/1/1371	keratin 5, gene 1 [Xenopus (S	54020785	NP_00100563	1.86	0	0	1.86	0	0	5.12	0.12	7.87	16.49	21.9	10.25
1-2.c36522/1/1461	--	--	--	0	0	0	0	0	0	0	1.14	0	4.37	5.13	0.7
2-3.c35092/1/2101	replication factor C subunit 1	313760612	NP_00118649	0	0	0	0	1.56	2.33	0	0	0.81	0	0	0
3-6.c18647/2/3287	LIM domain-binding protein	45360839	NP_989095.1	13.08	7.61	36.99	36.01	39.96	52.97	44.19	20.87	50.92	11.37	2.45	14.03
3-6.c16786/1/3080	PREDICTED: extracellular n	512865174	XP_00294261	0	0	0.13	0	0	0	1.48	0.14	0	2.41	2.96	0
1-2.c33418/1/1575	--	--	--	1.87	5.89	5.69	2.7	16.13	11.04	0	6.28	13.42	0	0	1.86
3-6.c8762/1/3240	PREDICTED: titin isoform X	847165031	XP_01282625	4.38	4.63	10.14	7.11	5.57	20.63	8.79	3.95	7.95	0.4	0.6	1.57
1-2.c33122/1/1377	PREDICTED: eukaryotic tra	847099370	XP_01281221	0	0	0	0	0	0	0	0	0	13.5	28.58	0.01
1-2.c37933/2/1785	PREDICTED: arf-GAP doma	847163505	XP_01282586	0	0	0	0	0	0	0	0	0	3.84	0	4.04
1-2.c49467/1/1545	MGC81975 protein [Xenopus	148223179	NP_00108767	0	0	0	1.59	0	0	5.1	0	9.48	9.88	16.83	14.67
2-3.c22976/1/1986	PREDICTED: beta-enolase is	837827704	XP_01278517	0	0	0.87	2.98	1.17	0.35	0	1.07	0.89	0	0	0
2-3.c4097/35/2421	tRNA methyltransferase 1 ho	147899382	NP_00109025	2.71	3.17	1.34	0	1.59	2.79	0.45	1.13	0.9	0	0	0
2-3.c43589/1/2404	PREDICTED: dolichyl-diphc	591383939	XP_00706642	7.8	0	8.42	8.21	13.62	22.42	4.55	0.04	1.42	3.25	1.67	3.68
2-3.c61126/1/2466	PREDICTED: nascent polype	847099448	XP_01281224	0.26	1.37	1.42	1.71	1.32	0.39	0.64	0.21	1.87	0	0	0.08
1-2.c11274/1/1199	PREDICTED: laminin subun	512850282	XP_00293418	15.51	19.74	9.08	22.99	7.63	17.72	46.97	16.33	19.12	246.64	257.39	222.5
2-3.c7616/2/2430.1	beta-hexosaminidase subunit	187607505	NP_0011206(	30	15.83	10.99	26.14	14.02	11.47	33.43	18.9	20.4	232.25	249.82	304.11

2-3.c41053/1/2526.	PREDICTED: abhydrolase do	301613203	XP_00293609	0.1	1.8	0.2	0.4	0	0.23	0	0.13	0.11	6.97	4.25	8.64
2-3.c53210/1/2595	PREDICTED: neurogenic loc	512877935	XP_0049193(	3.61	13.76	3.67	4.62	1.12	6.46	1.65	3.69	3.53	0	0.27	0.94
3-6.c2443/15/3739	PREDICTED: alpha-actinin-3	602667068	XP_0074391(	164.69	9.15	190.41	158.77	274.7	211.58	231.35	264.79	245.81	59.75	32.38	60.29
1-2.c3899/1/1490	histone-binding protein RBBI	160420243	NP_0010803(	0	0	0	0	0	0	0.01	0	0	1.73	5.77	1.58
2-3.c14016/1/2229	--	--	--	0	33.24	0	0	0	0	0	0	10.55	34.1	12.16	0
1-2.c32948/1/1450.	--	--	--	16.9	11.97	6.35	9.8	12.01	4.84	44.67	11.59	10.49	123.25	137.54	164.53
1-2.c43402/1/1454	--	--	--	0.56	0	0.24	0	0	0	2.09	0	0.6	3.31	2.13	4.31
1-2.c48748/1/4054.	PREDICTED: creatine kinase	126344225	XP_00136422	871	136.58	1208.24	1218.88	1023.33	548.77	471.69	835.99	1087.16	38.75	18.46	544.97
2-3.c11160/186/256	MGC84250 protein [Xenopus	148225296	NP_0010879(	443.29	24.68	419.93	853.57	432.41	227.95	263.28	398.58	468.31	61.34	13.32	75.59
3-6.c18511/1/2726.	PREDICTED: nebulin isoforr	512865746	XP_00491772	0.34	0.93	0	0	1.91	2.46	0.94	0	0	0	0	0
2-3.c23227/2/2303	PREDICTED: nucleolar prot	847087222	XP_01281669	2.12	4.76	0.88	2.06	1.69	3.92	1.23	0.28	0.34	0	0.27	0.39
2-3.c41745/1/2678	cyclic AMP-responsive elem	163915079	NP_00110639	0	6.21	0	0.16	0	0.08	10.07	0.15	6.7	36.75	0	28.75
2-3.c49988/1/1863	RecName: Full=60S ribosom	82186247	Q6P2Z6.1	42.98	56.72	22.59	18.31	17.92	38.91	25.3	18.68	14.28	4.86	4.65	5.89
2-3.c17993/1/2728	PREDICTED: NACHT, LRR	699666029	XP_00988359	0	1.09	0	0	0	0.02	7.71	0	0	7.64	0	16.27
1-2.c8694/2/1314.1	dehydrogenase/reductase SDI	166158027	NP_00110742	0	7.42	3.22	0	5.92	11.52	6.77	0	0	0	0	0
2-3.c51321/85/2577	WD repeat-containing protein	55742525	NP_00100678	0	11.07	3.57	7.31	21.42	44.31	9.61	4.55	9.85	0.41	5.18	0.25
2-3.c24391/1/2078	N-alpha-acetyltransferase 15,	282165843	NP_0010170(	7.8	9.53	3.25	3.15	4.61	7.75	0.66	3.22	2.7	0.31	0.67	1.1
1-2.c14903/1/1151	PREDICTED: phosphate carr	742180842	XP_01088807	0	2.2	0	0	0	0	0	0	0	73.9	22.87	2.44
2-3.c48401/1/2467.	--	--	--	5.4	3.95	0.85	2.98	5.06	0.93	13.52	7.23	6.15	51.91	51.33	51.99
1-2.c30224/1/1451	PREDICTED: short-chain de	683927591	XP_00909386	0	0	0	0	0	0	0	0	0	11.07	6.65	0
2-3.c34151/1/2124	zygotic Mcm3 protein [Xenoj	693576206	BAP63985.1	0.37	12.09	0.01	0.95	0.84	3.32	0.1	0.58	1.37	0.11	0.12	0
3-6.c4380/2/3270.1	nuclear factor 7, ovary [Xeno	284447304	NP_00107969	0.95	2.52	0.86	0	1.03	1.33	0.22	0.48	1.05	0	0	0
2-3.c22454/1/2571	serine/arginine-rich splicing f	55926178	NP_00100748	0	0	0.65	1.11	0	1.43	0.91	0	0	0	0	0
3-6.c16738/1/3191	PREDICTED: muscular LMI	512844542	XP_0029349(	39.99	2.41	13.08	18.11	20.97	6.28	8.52	13.57	18.47	1.94	5.89	2.96
2-3.c39725/1/2382	PREDICTED: transmembran	847111917	XP_0029323(	0	4.27	0.13	0	0.21	2.9	0.5	0	0.21	37.51	57.69	11.77
3-6.c2412/8/2920	origin recognition complex, s	148224764	NP_0010818(	0.59	10.46	0.39	0.4	2.04	2.32	0	0.97	0.46	0	0	0.27
1-2.c13865/1/1478	PREDICTED: actin, alpha ca	803091489	XP_00401102	4.09	0.24	17.03	14.49	8.29	9.46	19.41	21.17	9.57	1.75	1.16	2.27
3-6.c2487/27/3469	incenp-prov protein, partial [	54038417	AAH84167.1	0	5.94	0.28	0.35	0.91	2.12	0.21	0.49	0.85	0	0.17	0
2-3.c55924/1/2251	nucleolar and coiled-body ph	89886147	NP_00103481	15.44	34.39	4.44	9.52	10.1	15.1	7.1	9.33	4.45	1.56	3.2	2.18
1-2.c48929/1/1479	PREDICTED: CCR4-NOT tr	847103286	XP_01281312	0	0	0	0	0	0	0	0	0.52	2.71	5.42	0.99
2-3.c62226/22/2429	ribosome biogenesis protein t	156717538	NP_0010963(	8.62	27.82	4.34	4.79	6.52	13.58	5.31	4.85	4.39	1.18	2.72	0.85
1-2.c1984/28/1918	PREDICTED: UTP--glucose-	847127397	XP_01281836	131.21	14.5	53.73	92.56	107.07	25.02	28.85	88.35	79.04	20.98	10.14	6.45
3-6.c6481/1/3099	PREDICTED: uncharacterize	672020665	XP_00877445	2.79	0	1.23	0	0.76	0.44	0	0	0.89	8.99	7.22	9.15
3-6.c9111/1/3781	PREDICTED: ubiquitin-prote	847099174	XP_01281214	6.75	0.92	5.94	4.87	6.26	6.82	3.14	0.97	4.45	1.65	0.77	1.03
1-2.c34192/2/1617	PREDICTED: intercellular ad	847169795	XP_01280845	17.87	2.78	8.41	11.36	13.81	3.68	1.97	10.84	10.17	0.36	0.7	3.67
2-3.c46671/1/2124	PREDICTED: transmembran	602626865	XP_00742038	4.95	19.92	6.74	0	1.45	2.14	0	0	4.27	0	0	0
1-2.c3964/1/1248	--	--	--	27.92	0	0	16.04	3.96	7.4	35.45	64.29	41.03	465.91	1240.26	314.65
2-3.c55632/1/2196.	cytidine monophospho-N-ace	148227318	NP_00109075	0.34	1.38	1.13	0.71	2.54	1.06	0.03	0	3.65	0	0	0
1-2.c24998/1/1762	isocitrate dehydrogenase [NA	45361551	NP_989352.1	108.43	15.1	47.81	93.84	71.62	47.11	58.71	21.48	32.28	14.03	0.11	31.71
2-3.c61366/8/2558	PREDICTED: anaphase-prom	530617968	XP_0052987(	0.73	1.97	0.4	0.95	1.12	1.99	0	1.11	1.57	0	0.19	0
2-3.c16796/5/2676	LOC414460 protein, partial [	46250336	AAH68818.1	4.68	19.16	2.86	3.39	4.45	10.4	4.73	2.63	3.07	1.06	1.02	0.99
2-3.c16350/1/2354.	--	--	--	26.31	13.44	3.32	16.77	0.9	6.31	25.03	14.99	37.04	159.65	181.84	92.74
2-3.c34115/1/2116.	--	--	--	0	4.54	1.52	0	0	0	0	0	0	3.22	3.24	1.88
1-2.c30817/1/1152	solute carrier family 7 membe	45361265	NP_989210.1	30.72	6.92	13.55	61.89	9.21	0.94	28.9	59.83	48.02	256.33	542.45	318.51
2-3.c13511/1/2113.	conserved hypothetical protei	170067775	XP_00186861	7.34	0.16	0.39	2.48	7.24	0.13	23.34	3.31	4.82	94	51.11	50.7
2-3.c21618/1/1967.	PREDICTED: ras-related pro	768342989	XP_01159391	0	0	0	0	0	0	0	0	0	68.34	216.57	0
1-2.c11480/1/1682.	LOC733374 protein [Xenopu	76779620	AAI06554.1	216.25	109.1	97.13	106.98	63.11	54.8	40.06	100.09	103.95	17.69	18.72	13.82
2-3.c30661/1/2621	--	--	--	20.02	28.34	19.94	11.52	18.94	33.1	22.6	14.02	22.84	2.71	4.9	5.93
1-2.c41119/1/1557.	von Willebrand factor A dom	148228482	NP_00107996	20.73	21.83	18.07	16.04	21.01	12.21	29.4	13.29	19.09	228.2	231.85	332.83
2-3.c40469/1/2089	mothers against decapentaple	55926152	NP_00100748	0	15.43	1.35	1.01	1.14	0.81	0	1.98	0.2	0	0	0
2-3.c49992/1/2073.	--	--	--	0	3.18	0	0	0	0	0	0	2.98	10.04	12.48	0
1-2.c24386/5/1731	PREDICTED: protein PFC07	556961274	XP_00599098	3.74	2.8	9.69	4.49	5.52	22.97	5.3	4.44	7.73	0.25	0	0.21
1-2.c15630/1/1562	ubiquitin-like domain-contair	148231101	NP_00108786	2.94	3.26	0.55	1.64	0.86	1.41	3.19	1.98	3.1	25.01	34.78	15.6
1-2.c36842/2/1375	--	--	--	11.88	198.12	12.75	14.92	13.87	38.05	7.88	12.39	19.62	4.77	2.28	5.58
2-3.c17272/18/1977	PREDICTED: alpha-aminoac	597777684	XP_00725249	12.84	14.06	22.79	5.85	22.08	42.28	16.86	10.2	12.26	2.01	2.93	0
1-2.c41160/1/1501.	--	--	--	1.3	1.44	0.51	0.84	1.12	0.34	0.48	0.63	0.95	44.33	26.2	22.35
2-3.c9619/3/2210.1	E3 ubiquitin-protein ligase M	62858193	NP_00101691	7.5	9.39	6.88	4.62	7.79	5.99	0.92	3.18	3.08	0	0	1.76
3-6.c7025/1/3178	--	--	--	12.01	2.41	14.93	8.73	18.36	41.62	13.45	11.23	11.25	2.36	1.99	1.96

1-2.c40202/1/1606	Serine/threonine-protein phos	677443430	KFQ38846.1	1.78	2.49	0	0	0	0	0.25	0.49	0	4.97	1.66	1.44
1-2.c4317/1/1346.1	PREDICTED: target of rapan	847163389	XP_01282582	0	0	0	0	0	0	0	0	0	23.75	43.84	0.09
2-3.c53370/1/2190.	--	--	--	0	0	0	0	1.91	2.59	1.35	0	0	0	0	0
3-6.c15037/1/5322	staphylococcal nuclease and t	148234849	NP_0010796(	2.47	0	0	0	0	0	0	0	0	1	1.24	2.85
2-3.c20184/1/1791	PREDICTED: adipocyte enha	557297871	XP_00603123	0	27.4	0	0	0	0	0	0	7.26	10.09	5.62	0
1-2.c12917/2/1731	--	--	--	0.78	3.15	0.72	0.85	0.58	2.54	0	0	0	0	0	0
1-2.c29263/1/1833	--	--	--	11.9	5.4	10	7.57	11.38	6.12	9.16	8.9	8.5	2.17	2.2	0.47
2-3.c1464/77/1952	PREDICTED: voltage-depen	823417697	XP_01241959	88.9	37.76	74.38	60.4	68.81	106.22	73.47	82.67	64.3	13.32	11.07	28.1
1-2.c19914/1/1673.	PREDICTED: serine/arginine	768366525	XP_01158055	0	0	0	0	0	0	0	0	0	27.81	70.27	0
1-2.c49552/1/1183	PREDICTED: titin-like [Pyth	602631486	XP_00742264	6.56	0.13	100.6	28.65	51.18	83.12	31.43	38.97	18.04	0	0	30.63
1-2.c26986/2/1646	PREDICTED: microtubule-a	831279932	XP_01267097	3.14	28.36	1.05	0.89	0.01	1.57	0	0	3.18	17.58	16.96	14.52
1-2.c35145/1/1621	PREDICTED: polyadenylate-	696972430	XP_00955615	36.61	37.09	23.72	29.71	40.79	16.43	19.32	20.04	34.33	4.19	5.49	9.54
2-3.c42919/1/2172	PREDICTED: glucose-6-pho	847118565	XP_01281665	0.92	3.89	0.46	0.82	0.5	1.66	6.19	0.63	0.44	15.42	10.78	25.33
3-6.c13045/2/2981	PREDICTED: serine/threonin	512819921	XP_00491168	1.03	0.59	0.26	0.93	0.38	0.4	14.76	1.15	0.49	14.75	10.09	9.09
2-3.c12584/5/2220	PREDICTED: schwannomin	847126599	XP_0128181(	2.33	3.21	5.64	0.95	4.29	17.61	1.18	4.6	5.12	0	0	0
2-3.c29453/1/2784	PREDICTED: uncharacterize	847159899	XP_01282497	3.44	0.02	1.04	1.78	2.51	0.45	2.99	0	0	0	0	0.3
1-2.c26140/1/1459	protein archease [Xenopus (S	62857601	NP_00101679	0	0	0	0	0	0	0.22	0	0	4.14	10.63	1.25
2-3.c29827/1/1848.	PREDICTED: tenascin isofor	512858291	XP_00491677	19.89	72.22	42.67	20.54	25.12	40.47	19.1	18.75	23.55	10.56	3.49	3.83
1-2.c39101/1/1218	tomm22 protein [Xenopus (S	169642622	AAI60473.1	15.66	21.64	8.47	7.6	5.94	11.9	0.57	0.32	5.8	0.51	0	2.51
1-2.c51053/5/1453	protein disulfide isomerase fa	148226947	NP_00108005	0	0	0	0	0	0	3.82	0	0	21.63	58.92	0
1-2.c13765/1/1210	--	--	--	114.42	79.63	176.84	110.57	118.74	272	137.51	160.25	154.52	46.06	28.74	40.26
1-2.c24570/3/1494	PREDICTED: methionine-R-	640823407	XP_00806872	0	0	0	0	0	0	0	0	0	5.07	5.84	0
2-3.c34629/1/2074	--	--	--	4.7	5.42	4.68	12.27	7.38	6.33	7.12	5.9	6.85	1.43	2.52	1.06
1-2.c16804/1/1505	PREDICTED: atrial natriuret	742130720	XP_01087616	0	17.22	3.07	0	0	0	7.1	0	13.03	16.56	0	8.61
1-2.c31003/1/1397	--	--	--	2.73	0.46	8.17	3.51	3.09	2.07	2.23	3.37	3.6	0.39	0	0
1-2.c35832/1/1303	PREDICTED: LOW QUALI	700363815	XP_00992652	0	794.57	0	0	0	0	54.7	0	944.15	121.27	98.02	1770.21
1-2.c46263/1/6247.	larval type I keratin [Rana cat	134140868	ABO61147.1	4229.17	786.65	1022.45	2490.96	2269.79	421.38	2452.1	1186.35	1052.22	142.64	225.67	171.43
1-2.c51758/3/1422	ATP synthase subunit alpha,	71896075	NP_00102561	0	0	0	0	0	0	3.97	0.06	0	21.86	0.03	71.12
2-3.c24449/1/2240	tripeptidyl-peptidase 1 precur	156717508	NP_00109629	0	0.15	1.1	8.66	3.64	0	1.77	0.23	2.17	0	0	0
2-3.c37395/1/2492	microtubule-associated protei	148232096	NP_00108054	1.83	0.64	0.57	0.94	0.97	0	4.36	3.71	0	34.23	37.44	6.13
2-3.c32298/2/2537	PREDICTED: ralBP1-associ	847128049	XP_01281857	0.12	4.92	6.29	7.08	11.06	6.58	0.63	7.33	5.58	0	1.83	2.86
2-3.c43021/1/2102	PREDICTED: polynucleotide	512854490	XP_00293389	0	0	0	0	0	0	2.06	0	0	2.62	2.72	0.49
1-2.c49081/1/1290	PREDICTED: tetratricopepti	512840241	XP_0029315(	2.02	0.84	1.82	0.59	1.44	1.41	10.21	0.12	1.56	25.77	24.44	24.52
3-6.c13351/1/3275	elongation factor Tu GTP bin	148225911	NP_00107953	7.94	17.4	4.16	3.01	5.91	6.45	1.8	3.05	2.38	0.92	0.44	1.31
2-3.c34000/1/2484	--	--	--	4.31	12.14	4.72	2.07	3.19	11.86	0	1.17	0	0.29	0	0
1-2.c32267/11/1442	calsequestrin-2 precursor [Xe	45360999	NP_989136.1	258.21	73.88	638.77	264.16	316.27	79.27	426.34	131.21	391.29	56.63	46.45	26.41
1-2.c12339/1/1403	tropomodulin 4 (muscle) [Xe	148233551	NP_00108736	241.63	129.02	489.05	349.39	348.08	414.4	303.42	341.07	404.11	117.81	51.22	97.85
1-2.c8721/2/1375	protein FAM192A [Xenopus	187608361	NP_00112024	6	12.64	5.83	2.8	5.5	6.61	3.3	5.77	1.31	0.21	0.28	0.83
1-2.c34731/1/1599	ADP-ribosyltransferase 1 pre	160420185	NP_0011042(	20.07	6.57	18.26	20.39	37.24	17.99	3.49	30.56	22.37	2.7	0	12.24
1-2.c33213/1/1521	--	--	--	0	2.56	0	0	0	0.4	1.46	0.4	0.17	2.06	10.45	6.11
2-3.c12588/1/2552.	PREDICTED: asparagine syn	301603748	XP_00293157	0	0	0	0	0	0	0.52	0.25	0	4.58	7.35	0
1-2.c41256/1/1477	vasohibin-1 [Xenopus (Silura	213982977	NP_00113565	0.32	3.22	0.82	0.55	0.39	0	1.19	0.55	0.83	9.97	13.8	6.24
3-6.c8592/1/3030	tax1-binding protein 1 homol	45361339	NP_989247.1	6.5	4.68	7.17	5.51	8.99	4.48	3.57	4.95	3.97	0.66	0.46	2.27
1-2.c15735/1/3362.	PREDICTED: DCN1-like pro	847098455	XP_01281189	0	0	0	0	0	0	0	0	0	10.13	5.42	0.54
1-2.c12815/1/1636	PREDICTED: dedicator of cy	700433889	XP_0099558(	0	0	0	0	0	0	1.91	0	0.19	4.33	4.83	0
1-2.c17657/1/1218	PREDICTED: rho GTPase-ac	847148811	XP_01282258	1.45	1.35	0.83	1.06	0	0	1.3	0	2.72	11.34	31.07	10.26
2-3.c14572/2/1965	PREDICTED: myosin-4-like	301611451	XP_00293525	0	0	5.59	1.38	1.87	0	0.97	1.88	0.71	0	0	0
2-3.c33357/1/2167	uncharacterized protein LOC	148222298	NP_00108502	0.22	1.71	1.89	0	1.27	1.76	0	0	0.92	0	0	0
2-3.c9416/1/2462	PREDICTED: translocation p	831212036	XP_01265989	0	0	0	0	0	0	0	0	0	2.63	0	4.29
1-2.c19076/3/1506	RecName: Full=UPF0489 pro	123893185	Q28H30.1	4.7	10.6	4.8	4.27	4.99	9.6	1.49	2.84	3.07	1.12	0.06	1.05
2-3.c14240/1/2490.	secreted protein acidic and ric	42407294	BAD10858.1	97.8	883.51	246.33	179.09	245.63	500.28	226.45	108.12	137.68	74.64	27.58	96.25
2-3.c6626/1/2125	--	--	--	2.34	4.6	0.58	3.09	0	0.74	2.21	2.48	2.93	29.46	20.09	18.72
2-3.c27015/1/2555.	--	--	--	0	1.01	1.47	1.67	2.33	2.93	1.71	1.16	0.94	42.93	76.65	36.57
1-2.c8562/2/1334	matriptase a [Xenopus laevis]	148228853	NP_00108106	16.72	5.35	11.81	13.46	3.59	2.22	25.97	7.36	15.65	91.97	116.69	94.23
1-2.c44212/1/1259.	--	--	--	64.82	6.29	18.05	30.28	37.63	4.22	53.93	64.68	72.88	330.75	435.71	336.92
2-3.c11765/26/2074	U3 small nucleolar RNA-asso	147905015	NP_00108016	3.09	9.64	2.2	2.9	2.7	5.84	2.79	1.46	1.18	0.55	0.15	0.57
2-3.c4253/1/2522	PREDICTED: creatine kinase	126344225	XP_00136422	15689.85	1857.43	39160.21	27110.1	29684.49	15442.67	18055.52	36723.1	30214.51	67.71	52.61	15858.65
1-2.c21603/1/1337	baculoviral IAP repeat contai	148228259	NP_00108673	117.89	18.99	18.51	41.28	13.22	17.83	73.86	123.07	43.13	321.67	515.32	232.61

2-3.c14953/1/2242	--	--	--	8.47	288.36	17.68	38.53	13.37	71.57	30.46	20.21	46.54	485.93	862.33	363.26
2-3.c47350/1/2139	PREDICTED: LOW QUALI	512830904	XP_00293188	3.71	14.36	0	3.77	1.42	18.41	1.45	0	0.07	0	0	0
1-2.c14384/1/1556	alpha-actinin-4 [Xenopus (Sil	55741892	NP_00100681	3.96	3.36	2.27	10.15	0	6.9	25.35	2.23	15.75	133.74	226.56	60.89
2-3.c30934/1/1851	PREDICTED: protein PFC07	556961274	XP_00599098	0.31	0	0	0.42	0.95	2.19	0.23	0.42	0.69	0	0	0
2-3.c6292/1/2537	--	--	--	1.38	35.84	7.37	2.46	1.02	5.58	3.29	4.24	3.39	0.26	0.18	0.18
2-3.c7762/3/2228.2	--	--	--	0.21	42.16	0	4.35	5.39	15.42	7.12	4.42	6.68	136.5	213.38	66.38
2-3.c43189/1/2239.	cingulin [Xenopus laevis]>gi	148237354	NP_00108197	9.59	2.63	4.35	6.14	5.97	1.89	12.5	5.21	3.03	59.22	68.98	89.37
1-2.c25517/3/1770.	PREDICTED: leukotriene B4	512817971	XP_00293437	17.24	26.97	6.04	14.91	7.01	6.68	26.23	12.95	15.75	178.23	159.26	155.88
1-2.c17190/1/1919	PREDICTED: protein SET is	847153649	XP_0128236(	32.06	112.66	32.21	14.83	24.51	27.53	16.9	15.39	23.14	0	3.03	10.43
1-2.c2092/29/1416	PREDICTED: 2-oxoisovalera	512841749	XP_00293625	23.47	30	25.19	21.35	19.62	25.73	19.1	14.84	21.27	4.43	5.53	4.05
3-6.c10780/1/2885	PREDICTED: PDZ and LIM	847087387	XP_01281714	0.66	0.43	1.1	3.79	2.97	4.56	2.55	1.72	2.35	0.43	0.25	0.98
2-3.c9059/2/2298	succinate dehydrogenase [ubi	148237169	NP_00108347	0	7.56	0.24	0	0	0	0	0	0	0	12.51	6.25
2-3.c1703/41/2564	von Willebrand factor A dom	148228482	NP_00107996	45.37	17.96	33.35	33.11	47.39	20.6	32.02	25.91	22.76	6.73	6.06	11.27
2-3.c4247/1/1873	tln2 protein [Xenopus (Silura	166796755	AAI59153.1	58.72	34.59	54.54	40.6	34.7	41.77	56.6	36.23	37.69	1.06	9.95	15.42
1-2.c7224/1/1644	--	--	--	21.06	0	0	0	11.37	0	4.9	51.18	11.03	130.76	63.6	308.83
3-6.c4847/1/2806	Glycogen phosphorylase, mu	465954282	EMP25833.1	216.14	4.33	252.43	328.45	164.99	85.76	103.7	203.5	168.64	0	2.99	49.33
1-2.c41960/1/1601	PREDICTED: pyruvate dehy	530574466	XP_00528163	103.17	67.69	152.07	26.01	90.09	78.65	65.1	24.99	96.7	25.07	3.53	16.61
2-3.c27242/1/2599	MGC84250 protein [Xenopus	148225296	NP_0010879(	51.84	2.96	40.61	98.39	65.23	39.74	71.23	75.14	99.7	26.92	14.43	10.85
2-3.c33014/1/2256.	PREDICTED: carnosine synt	847171322	XP_00491926	3.95	5.78	5.41	3.99	5.19	12.88	3.45	3.2	4.64	1.41	0.51	1.23
1-2.c29999/2/1408	PREDICTED: NADP-depenc	301618178	XP_0029385(	19.61	0.93	2.53	9.51	4.69	8.81	14.88	9.73	9.54	81.04	98.85	150.32
2-3.c16068/5/2313	CMYA4 protein, partial [Xen	51261725	AAH80138.1	0.27	0	1.76	0.98	1.2	6.5	0	0.28	0	0	0	0
1-2.c21179/1/1630	DNA replication licensing fac	147898891	NP_00108075	2.06	25.67	2.05	0.96	4.4	7.46	0.27	0.66	1.73	0.16	0	0.57
3-6.c18437/1/3149.	--	--	--	13.19	4.49	3.42	1.98	7.51	5.04	14.69	11.02	7.18	94.17	83.48	76.99
1-2.c19791/3/1563	phosphorylase phosphatase [X	963087	CAA56714.1	11.51	14.92	0	0	0	4.71	4.78	4.83	0	17.2	25.67	32.17
2-3.c7237/5/2079	PREDICTED: calpain-3 isofo	512862211	XP_00293538	6.08	15.56	9.77	9.59	11.39	43.86	6.88	3.25	1.26	0.56	0	1.16
1-2.c2794/3/1695	ubiquitin carboxyl-terminal h	54020791	NP_00100564	7.15	1.04	2.07	0	5.09	2.95	3.11	0	1.32	0	0	0
1-2.c31478/1/1144	V-type proton ATPase subun	52345764	NP_00100492	13.36	8.56	0.12	12.09	12.85	5.19	2.69	5.08	0.12	0	0	3.96
1-2.c43290/1/1412.	PREDICTED: lysosome-asso	847098867	XP_01281203	11.93	9.75	0.68	21.45	8	0.85	0	6.12	7.89	0	0	0
2-3.c14397/1/2304.	--	--	--	3.48	1	5.53	3.23	3.55	9.54	1.79	2.44	1.71	0.14	0.16	0.55
2-3.c50318/1/2527.	PREDICTED: WD repeat-co	512828612	XP_00491281	1.47	11.1	1.47	1.22	2.84	6.74	1.25	1.29	0.82	0.36	0.33	0
2-3.c7688/1/2006	PREDICTED: myosin-4 [Xe	847156920	XP_0128244(	108.79	6.88	110.43	13.93	55.68	0	0	303.11	96.06	0	0	0.01
3-6.c14902/1/3185	PREDICTED: U5 small nucl	512827234	XP_00293258	0.85	10.37	0.7	0.55	1.83	2.54	1.65	1.05	2.22	0	0	0.36
1-2.c5194/1/1341	PREDICTED: dystrobrevin b	557318474	XP_00603242	0	0	0	0	0	0	0.26	0.13	0	1.83	2.36	5.33
2-3.c1223/82/2519	lamin B1 [Xenopus (Silurana	89272234	CAJ83381.1	0.36	0	0.4	0	3.05	1.8	0	0	0.01	0	0	0
1-2.c29009/1/3551.	--	--	--	104.16	72.86	107.8	147.94	347.64	937.79	393.23	172.88	442.49	30.11	22.65	8.03
2-3.c50127/1/2236.	--	--	--	0.97	14.9	0.93	1.23	1.13	5.82	3.67	0.63	1.86	56.7	42.15	27.77
1-2.c34000/1/5930.	PREDICTED: sarcalumenin i	847166841	XP_0128267(	2.47	1.28	4.03	3.63	7.46	2.07	3.26	4.48	4.04	0	0	1.24
2-3.c32678/1/2482	PREDICTED: protein tyrosin	149638430	XP_0015067(	20.62	10.19	15.06	17.48	12.94	4.47	5.36	9.25	9.72	0.31	0	4.68
2-3.c34714/1/1985.	--	--	--	0	0	0	0	0	0	0	1.77	0.73	20.59	14.3	5.41
1-2.c12734/5/1417	PREDICTED: histone deacet	557268092	XP_00601941	2.63	25.51	1.48	0.99	0	1.6	3.22	1.9	0.57	16.38	19.49	16.95
2-3.c48237/1/2174	phosphoglucomutase-1 [Xeno	47575814	NP_00100125	0	1.3	0	0	26.44	8.89	21.86	28.96	0	0	0	0
1-2.c45042/1/1418	PREDICTED: LOW QUALI	637377851	XP_00812323	42.85	28.37	81.25	74.05	92.76	42.63	18.57	52.87	153.4	4.91	2.15	39
1-2.c43448/1/1398	PREDICTED: uncharacterize	512834006	XP_00491352	8.84	0.83	78.02	3.58	39.72	31.9	21.64	31.09	7.44	0	1.55	2.24
2-3.c23886/1/2264.	--	--	--	0.52	20.42	0.7	0.95	0.3	3.32	2.75	0.68	0.95	22.78	21.55	28.88
1-2.c33214/2/1561	PREDICTED: isocitrate dehy	847172685	XP_01280957	28.6	7.93	40.67	16.32	43.93	20.34	26.52	38.05	30.32	8.03	5.96	3.12
2-3.c17501/1/2025.	--	--	--	3.13	0	0.66	1.57	0	1.42	11.19	9.37	2.4	32.69	34.83	21.06
3-6.c3539/3/3225	PREDICTED: uveal autoanti	512827341	XP_00491264	61.02	72.58	108.73	94.4	90.46	94.24	47.15	58.14	92.57	16.58	20.85	31.37
2-3.c26092/1/2252.	deoxyribonuclease II, lysosor	147904975	NP_00108667	4.83	1	2.76	4.55	3.77	0.68	3.05	4.62	4.06	44.34	66.8	51.59
2-3.c52164/1/2212	PREDICTED: phosphoribosy	847162833	XP_01282564	5.7	14.98	2.53	4.14	1.99	2.79	7.02	3.95	7.47	27.13	41.43	66.96
1-2.c19896/1/1318	PREDICTED: cathepsin F [E	742212064	XP_01089668	13.42	0.01	0	0	0	0	0	9.5	3.97	9.44	0.15	5.86
1-2.c4874/5/1366	HLA class II histocompatibili	308193352	NP_00118403	8.5	5	7.11	5.03	7.23	4.24	2.77	3.45	2.94	1.4	0	0.51
3-6.c13269/1/7498.	--	--	--	26.07	9.03	0.1	0.28	5.41	3.4	23.92	13.07	6.69	40.48	55.62	60.73
1-2.c21935/5/1755	transglutaminase 2 (C polype	147903280	NP_00108541	0.94	0	0	0.51	0	0	0	0	0.61	8.94	7.07	3.3
3-6.c14296/1/2681.	15 kDa selenoprotein precurs	30231240	NP_840079.1	0	5.29	0.63	0.16	2.23	5.91	0	0.09	0.52	0	0	0
1-2.c13946/9/1454	PREDICTED: LOW QUALI	803125841	XP_00401312	8.33	5.65	12.27	10.56	17.32	8.51	10.08	10.03	7.3	2.97	0.64	3.02
1-2.c17753/1/1235	hypothetical protein [Plasmoc	156094450	XP_0016132(	12.75	1.88	9.11	13.22	16.42	6.93	19.46	10.94	22.11	3.04	2.31	1.32
2-3.c16433/1/8592.	transcription factor IIIB 90 kI	307344666	NP_00118255	0.49	1.55	0	0.65	0.92	1.07	0.35	0	0.19	0	0	0
3-6.c7352/1/2719	--	--	--	1.68	18.76	7.31	9.39	10.42	41.14	6.72	2.88	6.36	0.25	0	1.42

2-3.c2293/6/2187	PREDICTED: acyl-coenzym	512837953	XP_00491407	0	0	3.52	0	2.07	1.5	0	0	0	0	0	0
1-2.c32774/1/1421	PREDICTED: transmembran	612054824	XP_00750467	233.12	88.91	175.32	114.19	195.12	32.43	70.69	273.67	94.52	10.23	6.91	8.93
2-3.c3712/2/2280	glyceraldehyde-3-phosphate	300679438	ADK27487.1	1.37	0	11.78	8.9	13.34	3.84	1.35	2.4	8.14	2.32	2.62	0.78
2-3.c45390/3/2360.	Fos-related antigen-2 [Xenop	213626733	AAI69992.1	11.94	31.38	2.1	5.24	3.11	1.84	17.47	11.48	3.97	51.83	57.02	64.05
3-6.c4836/1/2998	PREDICTED: polypyrimidin	847118922	XP_01281677	0	6.03	0.99	0.59	0.3	1.07	0	0	0	0	0	0
1-2.c15790/2/1471.	FXYD domain-containing io	226372508	ACO51879.1	30.88	20.68	26	33.18	27.58	28.14	46.25	28.42	24.38	251.38	598.27	416.93
2-3.c15842/1/3437.	--	--	--	8.64	4.87	2.72	8.53	4.87	2.11	10.12	10.84	9.5	80.87	92.51	87.23
1-2.c34792/1/1524.	PREDICTED: titin [Strongyl	780140340	XP_01168055	0.94	0.82	1.75	4.17	2.35	3.88	1.91	2.15	1.45	0	0.38	0
2-3.c16370/4/1979	protein kinase C and casein k	194018590	NP_00112338	1.45	3.01	0.41	0.65	1.29	1.09	0	0	0	0	0	0
3-6.c18349/1/3028	PREDICTED: bifunctional g	512844004	XP_00293606	10.96	13.08	9.53	6.74	7.67	16.62	6.56	8.18	13.17	2.33	2.02	2.21
3-6.c4952/1/3593.1	--	--	--	0.09	1.11	0.96	0.39	0.68	1.59	0.62	0.83	1.87	0	0	0
2-3.c29736/1/2669	PREDICTED: cullin-2 [Zono	542142665	XP_00548043	5.21	8.47	3.74	4.56	6.56	8.43	3.86	2.99	3.44	1.4	0.2	1.86
3-6.c15968/1/2729	uncharacterized protein LOC	163915221	NP_00110658	2.29	0.65	1.27	0.93	2.5	0.46	0.83	2.07	1.3	0	0	0.06
3-6.c8242/2/3196	epithelial membrane protein	58331827	NP_00101111	5.58	21.82	3.66	4.64	5.99	6.1	1.24	5.42	2.7	1.75	0.24	1.13
1-2.c19804/2/1648	transforming growth factor-b	118403632	NP_00107232	0	12.75	0	5.63	0	3.17	4.04	0	0.2	0	0	0
1-2.c26063/3/1878	Uncharacterized protein B04	677429359	KFQ30293.1	1.38	0	0.74	0.94	1.22	0.26	3.94	0.49	0.7	15.64	25.05	8.65
2-3.c6424/12/1923	phosphatidylethanolamine bi	148232176	NP_00108562	17.54	54.45	33.84	15.45	26.28	65.03	17.98	21.42	28.85	6.7	0.87	3.29
1-2.c18013/1/1256	regulator of G-protein signali	187608191	NP_00112012	0	0	0	0	0	0	0	0	0	12.74	7.54	0
2-3.c59331/1/2215	PREDICTED: lysosome men	696995434	XP_00956446	1.63	0.12	1.23	0.16	0.48	0.64	2.7	1.52	0.19	11.2	4.73	11.42
2-3.c9807/1/2350.2	PREDICTED: NFU1 iron-sul	847146530	XP_01282218	32.06	17.62	28.88	26.68	27.69	19.55	14.24	20.03	20.61	2.87	3.13	8.65
1-2.c51219/5/1501	LOC397919 protein [Xenopu	213625217	AAI70091.1	40.06	175.82	28.96	25.94	40.33	83.15	46.22	32.15	42.3	12.16	7.34	14.18
2-3.c21324/1/2288.	RNA-directed DNA polymer	808880956	KKF28667.1	5.58	7.48	1.77	3.36	1.66	1.75	6.58	6.03	3.65	38.23	46.06	27.81
2-3.c30193/1/2397.	PREDICTED: alpha-2,8-sialy	847084430	XP_01281233	1.16	2.51	2.97	2.06	2.89	8.09	3.08	1.78	1.6	0.43	0.93	0
1-2.c12399/1/1906	PREDICTED: myomesin-2 is	847130012	XP_01281893	49.98	26	61.74	102.29	83.65	146.16	101.18	91.02	106.89	39.84	19.79	22.91
2-3.c24276/1/2130	3-hydroxyisobutyrate dehydr	71896099	NP_00102560	0	2.64	0.25	0	2.64	6.44	0	0	0	0	0	0
1-2.c32903/1/1219	calsequestrin 1 (fast-twitch, s	148231736	NP_00108022	16.18	1.9	29.47	23.57	31.76	23.82	17.38	26.81	25.87	4.65	3.69	8.36
2-3.c38624/8/2139	aspartyl/asparaginyl beta-hyd	62751990	NP_00101573	1.64	0.67	2.31	2.27	3.29	5.41	2.19	2.64	1.56	0.55	0	0.73
1-2.c1792/30/1431	PREDICTED: enoyl-CoA de	847134418	XP_01281985	21.26	30.75	16.21	18.39	23.83	24.66	10.1	18.16	20.4	5.62	2.36	5.81
1-2.c22076/1/1323	kunitz-type protease inhibitor	113931260	NP_00103907	16	9.39	14.59	9.93	3.59	3.52	30.21	12.97	18.01	71.86	85.77	96.99
1-2.c2779/14/1330	fast troponin I [Rana catesbei	58397794	AAW73073.1	102.06	116.9	127.46	138.92	158.09	419.76	316.99	104.09	167.07	21.76	78.39	37.81
2-3.c16882/2/1922	PREDICTED: uncharacterize	768943650	XP_01161148	1.2	2.79	1.04	0.79	2.64	6.18	0.47	1.21	0.23	0	0	0.26
1-2.c17307/1/1378	PREDICTED: creatine kinas	126344225	XP_00136422	31.55	6.01	67.12	427.38	625.19	231.04	37.92	33.41	776.82	144.75	155.44	59.02
2-3.c26211/1/2402.	--	--	--	10.79	9.45	2.69	4.59	4.73	2.41	20.75	13.29	10.71	49.4	52.01	92.38
2-3.c58065/6/2127	calsequestrin 1 (fast-twitch, s	148231736	NP_00108022	22.51	4.82	39.83	34.2	39.47	30.27	24.63	33.45	29.95	8.7	7.08	9.43
2-3.c33251/1/2219.	--	--	--	0.35	5.71	0	2.56	0	1.59	2.96	0	3.93	33.7	38.23	23.96
1-2.c26192/1/1561	aconitase 2, mitochondrial [X	147904130	NP_00108626	101.2	30.48	85.55	81.47	97.68	56.58	63.59	74.63	65.8	12.21	13.88	30.05
2-3.c46682/1/2358.	--	--	--	2.73	25.58	3.19	3.42	3.84	6.65	12.01	3.04	2.34	75.39	90.4	49.45
2-3.c59547/1/2155	PREDICTED: sodium-indepe	512873189	XP_00293735	1.01	0	0	0	0	0	0	0	0	0.87	1.57	2.96
2-3.c32641/1/2664.	PREDICTED: keratin, type II	795536447	XP_01190357	66.36	0	0	0	20.06	12.89	0	0	0	0	0	0
3-6.c13040/2/3940	LIM domain-binding protein	45360839	NP_989095.1	1.9	5.77	25.92	9.41	19.94	52.07	18.31	9.54	13.54	0.74	0	3.2
1-2.c20205/1/1455	WD repeat and SOCS box-co	45361625	NP_989387.1	1.57	4.36	2.99	2.91	0	2.26	3.01	0	0	0	0	0
2-3.c55486/1/2083	PREDICTED: exostosin-like	530594220	XP_00529069	1.17	7.11	2.42	1.85	1.41	5.57	1.63	2.71	1.58	0.55	0	0.06
1-2.c47974/155/184	PREDICTED: LOW QUALI	803125841	XP_00401312	184.31	21.18	217.06	218.65	232.95	122.08	224.11	120.36	8.72	108.85	29.65	16.94
1-2.c44450/1/1420	--	--	--	0	15.14	0	0.87	0	0	0	3.09	0	26.78	32.06	2.98
1-2.c11766/1/1259	THUMP domain-containing	45360771	NP_989059.1	2.5	12.34	2.22	2.32	4.28	7.51	3.68	1.72	1.08	0.6	0.18	0.38
2-3.c35127/1/2286.	PREDICTED: sarcalumenin i	733909809	XP_01071766	0	0	6.22	6.63	5.97	3.26	4.6	7.66	3.03	0.98	0	0.97
2-3.c3861/2/2061	--	--	--	23.42	0.56	17.53	10.51	26.46	23.38	14.13	15.98	0	0	3.46	9.45
2-3.c8610/1/2391	--	--	--	79.78	4.79	20.34	37.55	42.56	10.22	35.14	19.06	29.5	4.7	8.29	4.8
2-3.c16487/7/2622	wdr18-prov protein, partial [	56971199	AAH88047.1	5.58	23.55	3.04	3.59	5.6	8.32	3.93	2.56	3.43	0.48	1.1	1.57
1-2.c13606/1/1310	MGC82089 protein [Xenopu	51950276	AAH82410.1	5.49	4.35	0.15	2.86	0	1.4	3.57	6.49	7.76	27.24	21.4	28.79
2-3.c8841/1/10846.	propionyl CoA carboxylase,	148227451	NP_00108929	0.97	0	0	0	0	0	2.14	0.67	1.87	5.99	2.54	3.59
2-3.c46002/4/2152	PREDICTED: diacylglycerol	301610039	XP_00293457	1.28	0	0	0.11	0	0	0	0.6	0.91	1.95	4.42	2.79
2-3.c53771/1/2303	TC1-like transposase [Cyprin	357575246	AET85182.1	0	0	0.11	0.59	0	1	2.31	0	0.48	8.71	12.35	9.51
2-3.c5157/1/2333.1	--	--	--	2.09	0	1.96	1.94	0	3.8	5.25	8.67	7.96	21.85	53.62	23.93
2-3.c13762/1/2290	transposase [Rana pipiens]>g	37678182	AAP49009.1	0.86	0	0	0.2	0.15	0	2.33	0	2.05	6.57	13.95	3.05
2-3.c1327/46/2417	myc proto-oncogene protein	45361629	NP_989390.1	29.24	33.3	18.74	17.46	18.09	21.8	27.06	17.78	28.92	7.28	2.66	3.46
1-2.c26161/1/1443.	--	--	--	0	0	0	0	0	0	0	0	0	16.66	39.59	3.01
1-2.c6864/1/1165	Tubulin alpha-1C chain [Lari	808859777	KKF11870.1	1.4	63.83	0	2.51	0	19.34	33	26.08	8.17	121.78	76.54	90.56

2-3.c46474/1/2345	TFIIH basal transcription fact	56118508	NP_00100813	3.97	7.53	0.07	0	0	0.1	2.96	0	0	6.81	8.45	0.19
1-2.c26277/3/1463	mindbomb E3 ubiquitin prote	189217792	NP_00112133	0	2.39	0.41	1.97	0	1.29	1.75	2.69	0	35.3	24.35	8.51
2-3.c52334/1/2434.	--	--	--	3.09	1.46	1.84	2.74	1.18	1.49	4.55	1.19	3.47	36.36	54.01	20.23
1-2.c19078/1/1665	--	--	--	3.48	1.41	1.32	6.8	1.39	0.92	4.97	4.18	1.3	46.71	36.12	56.39
2-3.c32369/1/2639.	PREDICTED: clathrin intera	301620587	XP_00293964	1.15	0.48	0.37	1.29	0	0	4.1	0.46	0	20.22	24.92	4.31
1-2.c33573/1/1443.	PREDICTED: TSC22 domai	847153187	XP_01282344	2.83	0	0	1.08	0	0	0.69	0	0	114.9	193.73	0
2-3.c57097/2/2045	PREDICTED: myosin-4 [Xe	847156920	XP_01282446	2.18	0	24.01	15.79	14.21	0.09	26.7	11.89	14.53	0.33	0.68	0
2-3.c59991/1/2138	PREDICTED: ataxin-3 isofo	847154356	XP_01282385	0	1.05	0.36	0.71	1.48	0.45	0.38	0.87	0	0	0	0
2-3.c15409/1/2262	PREDICTED: exocyst compl	558179412	XP_00612692	0	0.2	0.21	0.04	0	0	0.86	0	0.29	2.73	2.49	1.16
2-3.c33480/1/2145	PREDICTED: integral memb	695151462	XP_00950344	2.54	9.26	0	0	0	0	6.76	0	0	8.83	4.41	0
2-3.c45677/1/2334	vigilin [Xenopus (Silurana) t	55742112	NP_00100685	56.31	58.93	73.99	50.11	57.72	43.45	54.01	66.35	47.85	1.68	1.6	30.19
1-2.c34794/1/6686.	Actin, alpha cardiac muscle 1	440908718	ELR58708.1	12.77	1.19	56.79	20.18	25.3	37.31	62.26	60.34	38.16	8.75	6.52	4.01
2-3.c22719/1/2715	electron transfer flavoprotein	54020771	NP_00100563	5.13	0.82	4.27	11.24	7.63	3.45	1.44	5.89	1.26	3.49	0.79	0.56
1-2.c48560/1/1269	PREDICTED: spermatid peri	471370382	XP_0043757(	0.48	0	0	1.15	0	0	1.33	2.01	4.2	10.23	7.35	16.18
2-3.c38835/1/2382	--	--	--	4.83	4.95	8.47	4.19	9.44	9.68	5.97	3.3	6.4	1.84	0.22	2.3
2-3.c47937/1/2574.	predicted protein [Nematoste	156353049	XP_00162289	4.99	8.87	0.7	2.44	2.48	2.34	1.91	3.54	4.51	35.03	56.86	36.98
3-6.c15531/1/11745	myosin heavy chain, partial [	4249701	AAD13772.1	0.67	0	7.17	4.06	3.6	0	3.7	2.34	4.2	0	0	0.13
3-6.c6427/1/3121	Ribosome-binding protein 1 [	449268937	EMC79765.1	3.97	0.23	1.92	2.23	5.59	1.02	2.17	1.47	1.03	0	0	0.25
3-6.c6672/1/3709.1	PREDICTED: atrial natriuret	512816528	XP_00491126	0.48	7.96	1.1	0.59	0.78	2.17	0	0.46	0.66	0.15	0	0
2-3.c29586/1/2080.	ORF2 [Acanthochelys spixii]	6576738	BAA88337.1	2.4	12.33	2.27	1.85	2.43	6.88	10.6	2.44	3.8	60.71	75.92	66.05
2-3.c61430/1/1892	alpha 1 type I collagen [Rana	3242649	BAA29028.1	0	5.8	1.19	0.74	0.67	3.68	0.78	0	0.64	0	0	0
2-3.c38140/63/2537	arrestin domain-containing p	118404458	NP_00107269	8.3	10.68	0.79	4.68	1.28	1.06	9.38	9.91	5.07	18.95	38.74	46.36
2-3.c54016/1/2486	PREDICTED: 5'-nucleotidas	768395961	XP_01159374	7.67	5.81	9.38	9.69	7.57	10.28	9.38	6.76	11.76	0.33	0.76	4.11
1-2.c27330/3/1729	PREDICTED: N-lysine meth	573875669	XP_00662614	32.44	5.96	35.8	24.2	17.45	13.96	25.55	29.46	24.41	2.32	5.99	4.49
1-2.c43885/1/1689	--	--	--	4.07	1.92	3.83	2.41	3.12	5.14	4.61	4.98	2.72	0	0.15	0.85
2-3.c42257/1/1873	PREDICTED: phosphogluco	847117435	XP_01281625	32.3	9.26	56.87	20.52	13.73	30.94	20.59	4.09	41.34	2.56	9.08	3.89
2-3.c34539/1/2618	tyrosine aminotransferase [X	55742474	NP_00100679	3.38	0.81	1.65	1.83	1.46	1.4	16.02	1.78	1.73	0	0.39	0
1-2.c25284/1/4147.	Malate dehydrogenase, cytop	226372118	ACO51684.1	38.8	59.23	74.13	81.7	42.25	75.29	81.5	61.83	82.91	16.92	23.42	10.74
1-2.c34646/1/1313	--	--	--	0.12	0.46	0	0	0	0	0	0.29	1.1	4.55	6.16	0.04
1-2.c39392/1/1756	myosin X [Xenopus (Siluran	89268944	CAJ81959.1	2.64	7.5	2.36	2.59	1.73	5.82	3.26	2.76	5.76	47.78	42.55	53.21
1-2.c19142/1/1395	actin, alpha skeletal muscle B	820145793	KKX13565.1	581.72	105.55	1907.31	857.22	669.37	1164.74	2158.99	2149.94	1054.1	241.11	237.52	223.89
1-2.c23118/1/1751	PREDICTED: uncharacterize	847137252	XP_01282064	4.97	8.03	1.66	3.56	2.19	1.81	2.1	2.74	4.97	18.13	58.54	43.03
2-3.c35240/1/2155	low density lipoprotein recep	148225746	NP_00108256	0	0	0	0	0	0	1.77	0	0	3.29	0.5	1.65
1-2.c10976/1/1595	activating transcription factor	147902692	NP_00108699	9.64	20.1	6.95	11.9	7.49	4.79	1.14	2.47	1.87	0	2.42	1.92
1-2.c13781/1/1613	PREDICTED: nesprin-2 isof	847157071	XP_01282449	14.14	11.79	6.31	8.83	7.86	4.19	12.36	8.98	8.72	82.99	110.01	107
2-3.c48517/1/1908	PREDICTED: titin-like [Che	591350793	XP_00706984	0	0	0	1.43	0	1.73	0.27	0.14	0	0	0	0
1-2.c18270/1/1323.	keratin 14, type I [Xenopus la	262050515	NP_00115991	78.46	15.52	97.89	62.69	34.51	1.17	29.39	71.05	35.87	1.17	0	0
2-3.c36046/3/2226	PREDICTED: non-canonical	512834531	XP_00293741	0.82	8.44	2.34	2.42	2.09	4.48	1.76	1.79	2.75	0.32	0.35	0.38
2-3.c26540/1/2476	GI17572 [Drosophila mojave	195116485	XP_00200278	0.16	60.38	20.95	13.63	19.85	33.5	7.42	17.62	0	0	3.34	11.16
2-3.c3284/1/2142	PREDICTED: ectonucleotide	847130990	XP_01281912	3.03	14.81	0.8	2.98	2.13	4.17	1.42	1.77	3.07	44.01	50.12	40.2
1-2.c25258/1/1493	--	--	--	0.85	0	1.34	1.5	0.37	0	0	0	1.87	21.67	8.73	11.18
1-2.c33032/1/1342	NPL4 homolog, ubiquitin rec	147898562	NP_00108042	0	6.86	0.56	5.65	0.86	6.36	21.64	1.3	11.45	68.59	84.63	36.47
2-3.c2106/43/2489	MGC83764 protein [Xenopus	148237259	NP_00108656	2.64	12.3	3.18	2.4	3.55	3.44	1.28	1.87	1.47	0.68	0	0.56
1-2.c20637/1/1516	--	--	--	1.47	0	0	2.24	0.33	0.39	2.14	1.25	4.64	17.59	28.29	12.84
2-3.c47226/1/1917	tripeptidyl-peptidase 1 precu	156717508	NP_00109629	0	0	0	0	0	0.05	8.46	0	0	46.6	17.07	0.09
1-2.c13011/1/1207	BCL2-like 1 [Xenopus (Silur	53749666	NP_00100542	0.33	0.94	0.87	0	0.25	0	0	0	0	8.66	7.7	3.74
1-2.c39771/1/1345	serpin peptidase inhibitor, cla	56118698	NP_00100793	0	2.11	1.2	0.47	0	0	2.19	0	1.82	1.76	8.1	11.71
1-2.c43400/1/1608	LOC494840 protein [Xenopu	83318459	AAI08848.1	0	11.31	0.16	0	0	0	7.58	0	0.08	14.39	38.71	0
2-3.c18940/1/2571.	PREDICTED: vacuolar prote	847157353	XP_01282453	1.55	0.27	0	0	0	0	0.86	0	0	7.03	9.02	0
2-3.c27201/1/2774	CCR4-NOT transcription cor	148228213	NP_00109065	1.48	5	3.72	0	0	0	1.37	0	0	0.31	4.62	15.06
2-3.c15206/1/2410	PREDICTED: myosin-4 [Xe	847156920	XP_01282446	0.36	0.08	1.93	1.22	2.14	0	1.42	2.16	1.84	0	0	0
2-3.c20244/1/2388	--	--	--	10.71	7.42	11.31	15.07	8.03	20.05	15.95	6.92	15.12	3.63	4.63	1.89
2-3.c59393/1/1897	PREDICTED: tyrosine-prote	847087706	XP_01281833	0.46	6.45	0.53	3.57	4.01	4.46	0.24	2.15	0	0.55	0	0.89
2-3.c30924/2/1922	ATP-dependent RNA helicas	565321822	ETE72588.1	44.37	34.08	11.38	23.83	22.35	23.15	9.97	11.47	0.46	5.09	7.97	4.11
1-2.c24128/4/1485	--	--	--	54.97	7.59	8.9	29.62	9.93	5.88	57.57	28.34	25.66	231.11	396.87	132.17
2-3.c13982/1/1939	PREDICTED: keratin, type I	698421871	XP_00981374	45.12	56.95	31.44	27.22	156.15	35.01	85.58	62.1	33.13	0.95	0	0.74
2-3.c41197/1/2392	S-adenosyl-L-methionine-de	148224710	NP_00108996	0.79	2.1	0.65	1.17	1.56	2.79	1.07	0.35	0.36	0.42	0	0
2-3.c54148/1/2398.	--	--	--	0.05	0	0.95	0.27	0	0	2.06	0	0	8.04	12.77	7.53

1-2.c24309/1/1541	--	--	--	5.3	4.84	1.81	11.57	5.47	4.29	4.42	4.19	4.16	1.16	1.1	1.19
3-6.c4733/1/4374	Dnmt1 protein [Xenopus laev	49117032	AAH72774.1	1.19	4.56	1.38	0.87	1.81	1.87	0.43	1.13	1.14	0.08	0.08	0.36
1-2.c26359/1/1805.	--	--	--	16.48	16.44	3.57	5.2	3.55	9.38	95.19	12.06	6.27	108.17	122.66	46.56
1-2.c51460/338/181	mitochondrial ATP synthase	148223359	NP_00108012	430.14	256.33	339.14	330.45	438.75	261.33	241.33	357.5	466.53	62.96	110.5	106.5
1-2.c14204/1/2470.	--	--	--	29.6	38.98	77.04	49.27	102.75	362.93	59.42	39.16	130.1	2.07	5.44	1.88
1-2.c52125/1/1442	lumican precursor [Xenopus	58332414	NP_00101100	3.9	1.41	5.76	5.13	2.83	4.69	2.51	3.42	0	0	0	1.11
1-2.c1667/70/1628	adenylosuccinate synthetase i	148236946	NP_00109001	636.41	147.94	569.89	592.17	566.4	169.16	470.89	528.38	658.11	124.36	51.57	134.97
2-3.c52773/1/4411.	--	--	--	56.36	1.7	49.9	124.85	57.43	28.56	43.36	54.87	24.69	3.28	0.46	10.63
2-3.c10634/1/2132	PREDICTED: myb-binding p	512820645	XP_0049117	0.78	0	0	1.59	0.36	0.74	0.21	0.21	0	0	0	0
1-2.c39795/1/1840	1-acylglycerol-3-phosphate C	148222154	NP_00108527	0	5.36	0	0	0	0	0	0	3.04	5.69	11.22	0
1-2.c50654/3/1755	X-box binding protein 1 [Xer	147905550	NP_00108052	0	0	0	0	0	0	0	0	0	6.43	13.19	0
2-3.c20949/1/2254.	--	--	--	0	0.02	0.3	0	0	2.4	0	0	0	9.5	30.32	14.86
2-3.c39072/1/2271.	glutamate oxaloacetate transa	333805525	BAK26556.1	185.99	12.49	85.66	149.97	133.44	48.73	80.73	108.79	145.87	27.74	12.86	44.61
2-3.c15003/1/2253	PREDICTED: vacuolar prote	847111178	XP_0128148	0	0	0	0	0	0	0	0	0	3	0	2.34
1-2.c50142/1/1422	--	--	--	5.55	31.59	7.05	10.33	8.12	17.16	6.45	5.1	8.31	2.52	1.83	2.3
1-2.c14282/2/1384	--	--	--	6.84	114.67	36.41	20.53	6.8	38.34	20.53	22.53	26.93	3.93	3.13	0.82
2-3.c1801/24/2518	PREDICTED: ATP-binding	641776196	XP_0081713	2.37	3.43	0	0	1.68	1.16	2.46	1.58	0.38	0	0	0
1-2.c48991/1/1427	72 kDa type IV collagenase [	148231336	NP_00108069	3.52	24.34	2.42	0	0	0	10.02	3.38	0	40.9	0	15.14
2-3.c52304/1/2352	spastin [Xenopus laevis]>gi|8	148231546	NP_00108672	0.94	0	0	0.55	0	0	3.2	2.15	1.72	5.81	5.81	4.51
1-2.c30026/1/1350	peroxisome proliferator-activ	148233022	NP_00108131	1.94	2.05	2.38	1.88	2.08	0.53	1.33	2.17	2.21	0	0	0
1-2.c44744/1/1769	coenzyme Q-binding protein	148235208	NP_00108658	3.56	0	0	0	0	0	0	0	1.8	0.51	2.91	3.01
2-3.c7703/2/2065	PREDICTED: E3 ubiquitin-p	512842584	XP_0029401	0	0.3	0	0	1.16	1.71	0	0	0	0	0	0
1-2.c7804/7/1612	POLD3 protein, partial [Xeno	50415212	AAH77426.1	0	0	2.31	0	0	0	13.27	5.88	0	0.62	6.94	21.49
1-2.c13557/1/1455	--	--	--	0	3.08	0.48	0	0.06	0.07	0.37	0	0	4.6	4.96	3.28
1-2.c6174/1/1447	annexin A1 [Xenopus laevis]	147900738	NP_00108236	2.44	0	0	3.17	2.08	0	0	0	0	0	0	0
1-2.c45896/1/1223	--	--	--	2.85	2.59	0.55	1.17	1.51	2.81	1.43	2.75	3.65	37.28	27.23	27
2-3.c38158/13/2296	delta(3,5)-Delta(2,4)-dienoyl-	54020920	NP_00100570	1.74	8.55	0.53	1.49	0	3.04	0.58	4.34	0.89	0	0	0
1-2.c2460/5/1468	GDP dissociation inhibitor 2	148234617	NP_00108023	0	0	0	0	0	0	0	0	0	2.44	1.16	4.7
1-2.c47115/2/1204	crystallin, lambda 1 [Xenopus	148224534	NP_00109120	0	0	0	7.61	0.89	0	9.38	11.3	2.87	41.36	47.92	49.33
2-3.c61869/1/2045	inner-ear cytokeratin [Rana c	6980088	AAF34720.1	26.17	22.16	19.97	13.04	14.79	32.24	16.46	16.7	21.59	3.05	4.66	5.96
2-3.c21937/1/2475.	--	--	--	0	0	0	0	0	0	0	0	0	8.88	12.06	0
2-3.c60080/1/2119	--	--	--	8.58	17.01	9.56	9.99	8.15	19.35	9.86	4.07	9.18	2.78	2.91	2.46
1-2.c5155/1/1647.2	--	--	--	7.44	13.04	4.23	6.98	4	4.43	8.41	4.52	6.04	72.25	101.19	80.33
1-2.c15459/1/1398	PREDICTED: Down syndror	512823845	XP_0029415	0	1.17	0	0	0	0	0.69	0	0	2.66	1.47	5.01
1-2.c30336/1/3382.	Y-box-binding protein 3 [Xer	62751871	NP_00101585	1425.6	1049.68	1531.75	1231.93	1533.4	4096.78	1133.39	1482.47	1301.27	399.92	195.87	454.9
2-3.c19034/2/2239	bystin [Xenopus (Silurana) tr	62752053	NP_00101583	2.35	3.79	0.59	1.71	0.79	3.03	1.65	1.86	1.37	0	0.44	0
2-3.c30092/1/2403.	hypothetical protein N312_01	676249687	KFO13559.1	1	2.68	0.77	0.59	0.77	1.85	22.87	1.04	1.2	13.49	22.23	24.51
2-3.c34905/1/2313.	ORF2 [Acanthochelys spixii]	6576738	BAA88337.1	1.29	4.97	1.56	1.71	1.24	2.69	6.18	2.15	1.33	35.91	34.07	33.84
1-2.c34945/2/1665	tln2 protein [Xenopus (Silura	166796755	AAI59153.1	0	0	0	0	0	0.01	4.44	0	0	5.62	10.67	0
1-2.c41072/1/1360	--	--	--	0	7.93	0	0.23	0	0.32	0.89	2.27	5.59	16.75	13.75	1.47
1-2.c31272/1/1332	synapse associated protein 1	147899972	NP_00108504	5.87	0.46	3.62	7.06	0	5.85	11.74	6.67	9.72	64.29	70.25	50.36
2-3.c61212/1/2146	--	--	--	6	13.4	4.39	11.76	9.68	10.79	4.94	5.46	9.9	2.73	2.14	2.16
2-3.c14127/1/2577	MGC82791 protein [Xenopus	147906957	NP_00108591	0	0	0	1.09	0	2.02	0	0.61	0	0	0	0
1-2.c7608/1/1220	T-cell, immune regulator 1, A	60422792	AAH90359.1	2.06	2.07	0	1.3	0	0.03	1.5	2.43	0.53	9.73	17.34	10.39
2-3.c29408/1/2079	--	--	--	52.99	3.92	28.14	100.09	105.08	8.1	277.09	88.79	93.31	892.2	901.6	1084.06
2-3.c39976/1/2109.	--	--	--	0	0	0	0	0	0	0	0	2.21	17	38.5	0
1-2.c21596/3/1468	staphylococcal nuclease and t	147905764	NP_00108050	21.39	81.66	14.62	16.25	18.17	35.73	7.31	9.49	15.99	0	1.38	12.55
1-2.c24007/1/1310	ST3 beta-galactoside alpha-2,	147899298	NP_00108946	0.84	14.24	0.83	1.44	2.12	7.11	1.96	1.01	1.86	54.74	51.15	46.56
3-6.c2838/10/3582	solute carrier family 25 meml	147905724	NP_00109049	53.75	10.78	42	52.43	31.81	12.15	24.15	44.27	41.35	4.19	4.92	4.83
2-3.c43219/1/1970	fibronectin precursor [Xenop	148237139	NP_00108127	160.23	387.28	104.8	87.34	71.78	85.46	114.81	83.7	85.18	964.76	804.56	1318.49
1-2.c26171/1/1387	uncharacterized protein KIAA	166157929	NP_00110737	0	0.44	0	3.42	0	3.09	4.25	0.14	5.22	38.28	47.75	17.56
2-3.c1656/27/2260	ATPase family AAA domain	47498020	NP_998849.1	5.01	9.69	3.3	5.56	11.7	11.59	4.95	3.9	6.62	2.17	2.08	2.03
1-2.c13529/5/1615	CDK5 regulatory subunit-ass	58332734	NP_00101144	9.55	15.85	7.81	9.06	6.45	13.41	6.01	5.59	5.4	1.42	2.37	1.82
1-2.c40892/1/4041.	alpha-cardiac actin, partial [M	553858	AAA37165.1	66876.38	9995.89	92150.49	66368.86	83817.09	69615.86	53212.6	91864.33	56266.36	21610.39	11275.31	24241.46
2-3.c43293/1/2455.	MGC78980 protein [Xenopus	147901711	NP_00108539	0	0.24	0.51	0	0	3.18	11.48	2.09	7.14	30.82	29.76	16.65
2-3.c60794/2/2103	PREDICTED: ETS transloca	564254896	XP_0062664	4.46	14.17	4.85	2.95	6.02	10.23	1.2	1.49	6.06	0	1.28	2.05
2-3.c18947/4/2004	PREDICTED: calcium-bindi	664741920	XP_0085282	8.61	12.15	0.01	4.84	8.96	8.55	3.15	1.54	0	4.35	0	0.07
2-3.c25592/1/2547	glutamyl-prolyl-tRNA synthe	189217812	NP_00112134	5.05	6.29	7.44	4.97	4.6	8.41	3.47	4.22	7.38	1.09	1.13	1.25

2-3.c46954/1/2338.	--	--	--	31.84	13.51	14.23	26.11	12.95	6.07	45.6	35.54	23.72	224.02	187.69	194.11
2-3.c53822/1/2185	PREDICTED: fibronectin typ	512827549	XP_00293967	45.92	13.25	37.16	27.61	31.6	22.1	21.97	33.43	28.5	5.76	10.71	5.25
2-3.c48169/1/2278.	--	--	--	1.71	4.77	1.6	2.87	2.46	3.57	13.29	1.99	2.54	46.85	54.66	47.94
2-3.c59915/1/2270.	zinc finger protein-like 1 [Xe	147907246	NP_00109117	0	0	0	0	0	0	1.31	0	0.54	5.28	7.57	0
2-3.c25665/1/1969	PREDICTED: glucosidase, b	847088564	XP_01282071	12.86	31.18	6.68	11.04	8.68	15.98	9.37	4.04	3.02	1.71	1.96	3.7
1-2.c12067/11/1910	PREDICTED: splicing factor	641783658	XP_0081742(	4.58	46.79	5.27	0	1.65	3.23	0	1.61	2.73	0	0	0
2-3.c2164/13/2368	PREDICTED: translation fac	301607837	XP_0029334(	2.72	5	1.99	1.9	5.86	6.58	1.74	1.45	3.57	0.58	0.42	1.31
2-3.c8247/12/1975	PREDICTED: 3-ketoacyl-Co	847088369	XP_0128203(	16.75	35.71	20.02	27.15	20.78	35.87	19.79	13.12	12.68	3.92	2.69	12.44
1-2.c13235/5/1851	PREDICTED: probable ATP-	847108646	XP_0128140(	10.93	26.64	4.74	8.19	7.62	17.45	9.46	5.21	4.13	2.45	2.9	1.7
1-2.c27244/25/1464	lumican precursor [Xenopus (	58332414	NP_0010110(	16.45	6.99	10.27	10.57	18.22	5.38	1.6	4.97	10.85	2.55	0	3.69
1-2.c15638/4/1681	Myozenin-1-like [Xenopus la	148237556	NP_00107945	18.87	0.21	20.11	23.02	20.21	10.49	22.37	18.51	19.57	2.94	3.47	5.93
1-2.c20352/1/1504	PREDICTED: glutamine syn	541978789	XP_00544205	114.73	50.51	28.78	175.79	62.06	1.44	6.79	79.99	78.45	0	0	0.74
1-2.c23240/1/1661	--	--	--	7.75	106.1	4.33	7.84	3.63	41.71	7.41	9.35	13.94	280.81	112.51	236.78
1-2.c30283/1/1375	PREDICTED: collagen alpha	512850191	XP_00293872	0.03	0	0.51	5.01	2.65	0	6.49	3.09	4.49	0	0	0
1-2.c45263/1/1636	PREDICTED: trafficking pro	847136021	XP_01282041	1.45	1.06	3.85	1.46	2.26	0	0.02	0	0	0	0	0
1-2.c47868/56/1528	PREDICTED: intracellular hy	847086348	XP_01281437	3.98	9.78	3.84	5.53	4.69	6.61	4.54	2.14	4.01	0.73	0	1.42
1-2.c7256/1/1317	--	--	--	0	34.06	2.47	2.24	0	6.18	7.91	1.55	3.93	42.8	114.65	43.94
2-3.c11560/2/2306.	PREDICTED: protein FAM1	847092350	XP_00293564	0	0.74	2.05	1.51	0.78	0.19	0.78	0.45	1.83	0	0	0
2-3.c36846/1/2727	PREDICTED: ras-GEF doma	847087760	XP_0128184(	2.46	0	0.68	0	0	0	1.95	1.47	0	3.98	1.94	0.16
3-6.c16219/1/3172	voltage dependent calcium ch	11414922	BAB18553.1	0.1	0.7	0.36	1.84	1.13	4.17	2.38	1.26	1.02	0	0	0.82
1-2.c25996/1/1331	PREDICTED: enoyl-CoA hy	821486130	XP_0124057(	6.3	88.93	11.2	7.28	6.8	13.24	4.2	5.76	6.53	2.2	0.82	1.61
1-2.c8712/1/1530	legumain precursor [Xenopus	54020950	NP_00100572	67.52	20.65	15.38	33.64	28.62	11.21	49.07	36.39	55.59	336.17	240.01	389.44
2-3.c40482/1/2104	calsequestrin 1 (fast-twitch, s	148231736	NP_00108022	4.46	0.83	5.19	4.73	3.08	3.75	3.89	3.46	4.17	0.23	1.11	0.45
1-2.c18353/1/1783	tropomodulin 4 (muscle) [Xe	148233551	NP_0010873(	46.67	22.52	79.96	52.51	51.48	76.66	44.86	58.79	59.91	23.36	16.78	8.42
1-2.c6580/5/1363	UBA domain containing 1 [X	148235257	NP_00108937	7.72	0	3.66	9.31	6.35	7.75	2	2.69	0	1.52	2.77	0
1-2.c2878/14/1585	ribonucleotide reductase m2 p	89268988	CAJ81953.1	4.48	20.29	0.59	5.02	7.71	16.35	3.85	2.54	9.61	3.16	2.82	0
2-3.c33225/1/2406	PREDICTED: ataxin-2 isofor	512811031	XP_0049105(	0	0	0	0	0	0	0.43	0	0	1.04	0.54	3.07
2-3.c39106/2/2450	HEAT repeat-containing prot	58332728	NP_00101143	7.15	7.14	5.03	3.78	5.53	7.74	2.59	4.07	3.97	0.59	1.03	1.52
2-3.c44096/1/1962	aspartyl/asparaginyl beta-hyd	62751990	NP_00101573	3.22	1.85	5.29	3.1	5.46	9.97	4.15	4.39	4.53	0.98	1.85	0.63
1-2.c38748/1/1522	--	--	--	7.17	6.65	4.59	4.51	4.81	6.79	12.7	0.9	1.86	66.16	62.2	89.32
1-2.c4420/1/1487.1	--	--	--	0	0	0	0	0.39	0.64	0	0	0.31	7.71	17.43	8.37
3-6.c13094/2/2812	structural maintenance of chr	147900881	NP_00108374	0	0	0	0	0	0	0	0	0	1.61	0.03	2.91
1-2.c38549/1/1415	protein phosphatase 1 regulat	820146588	KKX13916.1	11.77	30.38	26.76	10.66	11.85	53.72	8.94	5.1	7.92	0.24	0.73	0.59
1-2.c35667/1/1583	--	--	--	3.04	0	0	1.82	1.41	1.56	0.07	1.01	1.94	0	0.17	0
1-2.c43909/1/1400	PREDICTED: protein kinase	685612499	XP_00919443	0	0	0	5.15	0	0	1.34	3.45	1.73	33.09	41.23	14.57
1-2.c5097/2/1899	dehydrogenase/reductase SDI	166158027	NP_00110742	20.56	8.37	20.07	16.5	14.27	10.85	12.69	8.16	20.15	0.48	0.82	7.23
1-2.c32192/101/146	voltage-dependent anion char	147906224	NP_00108535	202.43	134.99	136.81	135.43	110.24	63.49	79.6	102.18	102.3	23.47	5.82	48.59
1-2.c13616/2/1421.	--	--	--	6.64	12.86	7.33	9.12	6.37	14.13	14.46	4.02	10.42	119.38	144.58	143.56
2-3.c15871/11/2666	PREDICTED: elongation fac	301607385	XP_00293328	6.26	6.38	3.14	5.4	6.56	10.04	2.9	2.26	3.05	1.52	0.93	2.06
2-3.c63546/3/2210	myosin heavy chain, partial [l	4249697	AAD13770.1	0	0	4.17	1.34	1.06	0	2.56	0.25	1.22	0	0	0
2-3.c2630/2/2575.2	--	--	--	34.62	32.57	0.09	1.03	0	0	76.87	0	0	0	254.57	140.55
2-3.c5324/4/2056	PREDICTED: tenascin isofor	512858291	XP_00491677	1.63	0.51	7.86	1.53	2.99	0	1.62	1.11	1.23	0.01	0	0
3-6.c14911/1/2839.	--	--	--	9.35	51.35	8.98	6.64	7.82	9.43	16.27	7.69	11.23	146.64	130.57	59.63
2-3.c5710/2/1883	PREDICTED: RAC-alpha sei	301622901	XP_00294076	3.65	9.72	1.68	2.16	1.98	3.18	0.31	0.41	0.79	0	0.17	0.38
1-2.c14515/1/1815	PREDICTED: elongation fac	803119291	XP_01204081	338.49	333.5	234.32	110.16	219.58	314.94	138.61	158.1	188.46	59.87	25.21	79.84
1-2.c22857/1/1624	embryonal Fyn-associated sul	148235363	NP_00108837	0	0	0	0.4	0	0	1.32	3.11	0	8.79	7.44	1.2
1-2.c46046/1/1421	--	--	--	4.16	10.44	0	0	0	0	0	3.95	5.44	0	28.66	11.94
2-3.c48914/3/2386.	PREDICTED: protein LZIC [	704504142	XP_01007875	2.83	0.86	3.46	2.92	2.48	1.92	1.08	0.73	0.44	0.25	0	0.39
2-3.c49018/1/2587.	Mimitin, mitochondrial precu	226372810	ACO52030.1	6.13	8.88	6.86	7.37	4.25	5.76	5.17	5.9	4.82	0.4	1.23	1.49
3-6.c14157/1/2691	Cysteine--tRNA ligase, cytop	676706853	KFO84757.1	11.4	14.79	10.43	7.17	11.12	15.37	8.94	9.96	8.57	2.07	0.57	4.59
2-3.c19888/1/2406.	--	--	--	0	0.38	0.15	0	2.5	1.63	0	1.59	0	0	0	0
2-3.c27193/1/2161.	PREDICTED: transmembran	301612255	XP_00293563	3.34	2	1.48	1.38	0.68	0.61	0	3.39	3.76	42.06	31.07	7.31
1-2.c39431/2/1389	--	--	--	5.83	10.34	2.07	4.68	1.43	6.43	6.72	4.64	7.16	44.47	48.72	78.83
1-2.c49606/1/1305	--	--	--	0	4.35	1.31	0	2.19	3.82	0	0	0	0	0	0
1-2.c5788/18/1677	PREDICTED: nucleolar com	847086891	XP_01281575	4.82	16.74	2.32	2.86	3.54	7.52	3.59	4.19	2.48	0.78	0.48	0.6
2-3.c36998/1/2078	Transmembrane BAX inhibit	225708156	ACO09924.1	0.06	16.39	0.12	0	0.03	0	0	0	26.81	19.56	8.73	0
2-3.c55627/1/2144.	PREDICTED: poliovirus rec	513222218	XP_00494808	0	4.19	1.79	2.37	1.94	3.2	3.15	0.68	1.72	0	0	0.14
3-6.c13530/4/4075	protein-arginine deiminase ty	156717898	NP_00109649	7.49	0.64	12.18	16.56	17.69	12.16	22.5	1	1.47	1.87	8.09	3

3-6.c19073/1/3623	PREDICTED: DNA-directed	291241363	XP_00274058	0.32	3.41	0	0.86	2.7	3.36	0	0.57	1.05	0	0.65	0.44
2-3.c48135/1/2112	--	--	--	3.13	3.18	1.74	1.52	2.17	0.62	6.06	0.61	0.8	20.2	11.07	34.9
1-2.c3595/4/1873	eukaryotic translation initiatio	187608058	NP_00112056	38.13	60.95	26.48	19.37	18.98	40.8	17.77	24.13	20.27	5.51	4.36	8.75
1-2.c9606/1/1525.1	PREDICTED: calcium uptake	847172778	XP_01280960	2.43	3.89	8.66	4.23	8.17	4.82	2.02	3.56	2.73	0.61	0	0.26
1-2.c29178/1/11718	tripartite motif containing 54	147905260	NP_00108509	65.24	12.79	21.08	20.97	40.44	26.48	25.79	42.17	18.87	2.09	10.94	5.69
2-3.c32781/1/2469	3-hydroxyisobutyrate dehydro	71896099	NP_00102560	7.82	2.69	4.16	7.37	3.85	1.49	7.05	5.33	3.71	0.86	0.19	0.14
1-2.c1713/18/1645	PREDICTED: fumarate hydra	512842641	XP_00293586	27.07	0.68	0	0.25	28.3	27.23	10.18	0	44.23	1.15	0.89	0
2-3.c37219/1/2338	--	--	--	0.18	0	0	0	4.16	7.23	0.42	0	7.65	0	0.19	0
2-3.c58217/38/2132	high density lipoprotein bindi	160420251	NP_00108016	30.62	35.13	31.71	34.39	23.84	25.52	42.49	13.44	27.71	3.25	0	15.81
1-2.c30722/2/1799	15 kDa selenoprotein precurs	30231240	NP_840079.1	8.74	15.85	5.97	3.41	6.83	8.28	1.86	2.52	3.77	0	0.51	2.36
1-2.c32601/2/1413	--	--	--	7.78	0.44	5.76	84.94	0	5.71	105.78	41.34	43.57	324.64	365.69	478.36
2-3.c57925/3/2302	PREDICTED: 60 kDa heat sh	768339285	XP_01157440	0.2	1.39	0	0	1.39	1.07	0	0	0	0	0	0
1-2.c25403/1/1833	PREDICTED: protein tyrosin	149638430	XP_00150670	29.56	1.61	11.52	22.9	36.09	9.8	13	11.4	14.4	0	0	10.82
2-3.c48822/1/2116.	--	--	--	32.83	3.45	14.21	16.52	15.96	9.72	9.18	9.93	17.58	1.83	4.56	2.68
2-3.c5120/2/2021	--	--	--	4.57	0	8.9	25.17	6.93	0.07	0	0.83	4.71	0	0	0
3-6.c16923/1/3292	voltage dependent calcium ch	11414922	BAB18553.1	12.44	7.08	15.33	15.29	15.12	24.79	20.2	15.88	15.41	3.73	4.3	5.78
2-3.c42447/1/2277.	von Willebrand factor A dom	148228482	NP_00107996	4.54	6.9	2.56	2.45	5.65	11.54	10.48	3.23	3.31	59.77	81.82	107.48
2-3.c10111/4/2472	PREDICTED: long-chain fatt	512839272	XP_00491424	1.03	0	1.68	2.29	0.35	0.69	0	0	0	0	0	0
2-3.c48426/1/4415	PREDICTED: beta-enolase [	637381761	XP_00812393	0	0	0.02	0	0	0	0	0	0	71.25	17.54	0
1-2.c28936/2/1245	nucleolar protein family A, m	89271893	CAJ82850.1	9.15	55.6	8.45	10.79	10.89	31.68	19.73	7.16	6.36	4.14	2.51	2.96
1-2.c36779/1/1350	transforming, acidic coiled-co	147905121	NP_00109042	4.87	13.2	1.65	0.01	0	0	4.68	2.47	0	2.59	1.24	4.67
2-3.c2248/10/1935	rrp1b protein [Xenopus (Silu	60552498	AAH91594.1	34.8	66.49	22.54	18.2	18.75	37.07	24.06	19.97	16.83	5.09	10.62	3.53
1-2.c50413/1/1221	PREDICTED: ubiquitin-prote	637309248	XP_00811049	3.72	0.59	0.17	1.43	0.76	0	4.53	1.28	2.01	26.99	8.52	13.67
2-3.c13202/2/2332	aquaporin [Hyla japonica]	148356713	BAF63030.1	39.06	16.69	11.71	16.96	31.76	0.24	24.2	18.93	14.12	0	0	0.62
2-3.c41661/1/2205	PREDICTED: serine/threonin	512844020	XP_00491484	4.29	3.54	4.65	4.24	3.63	5.06	1.93	3.06	4.73	0.58	0.34	1.07
3-6.c7691/1/3994	translocating chain-associated	147905532	NP_00108662	1.58	1.98	1.19	1.9	1.45	1.87	0.17	0.79	0.64	0.35	0	0.34
1-2.c34246/1/1383	MAM domain-containing pro	118403772	NP_00107284	0	0	0	0.38	0	0	0.96	0.52	0	7.91	5.26	5.14
2-3.c48540/1/1999	zyxin [Xenopus laevis]>gi|18	148886688	NP_00109215	14.34	29.93	5.64	9.9	5.45	6.96	21.15	10.44	6.47	112.04	116.48	69.77
2-3.c25377/1/2261.	--	--	--	0.97	2.31	0	1.68	0	1.78	0	0	1.79	0	0	0
1-2.c51625/6/1680	GI12770 [Drosophila mojave	195125345	XP_00200713	1.78	3.31	0	0	0	3.99	0.9	0.77	0	15.66	23.56	20.07
1-2.c2716/15/1806	PREDICTED: solute carrier f	512851875	XP_00293681	8.21	2.07	5.01	1.66	4.02	1.69	4.19	1.93	4.02	0.29	0	0.24
2-3.c25610/1/2248.	PREDICTED: liprin-beta-2 is	697434073	XP_00967843	0	4.07	5.03	0	2.87	0	5.16	6.71	10.33	17.84	23.26	18.74
1-2.c1840/18/1740	cystathionine gamma-lyase [X	148238287	NP_00108798	103.05	25.28	49.54	68.13	121.71	25.39	131.89	139.94	119.73	6.24	12.32	8.02
2-3.c19950/1/2474.	PREDICTED: ubiquitin-asso	556949722	XP_00598744	0	0	2.54	5.8	1.59	2.01	1.54	1.36	2.15	0.31	0	0
3-6.c11523/1/3006.	PREDICTED: acidic leucine-	768401936	XP_01159692	5.98	24.37	9.68	6.53	12.99	9.21	2.05	3.61	3.49	1.3	1.7	2.89
1-2.c27381/2/1371	acyl-CoA-binding domain-co	577861032	NP_00107282	0	2.12	1	0	0	0	2.73	0	0.01	3.59	4.89	0.65
2-3.c3893/2/2463.3	--	--	--	1.52	5.41	0	3.21	0.73	2.67	6.85	1.28	0.45	39.76	49.08	53.47
2-3.c35192/1/2163	--	--	--	0.2	1.07	0.07	0.57	0.23	0.36	2.19	0.15	0.48	10.04	5.77	8.37
1-2.c36197/1/1377	cullin-3 [Xenopus (Silurana)	134085433	NP_00101597	0	0.17	0.51	1.39	0	0	6.22	0	1.74	12.18	12.77	9.16
2-3.c39062/1/2545	PREDICTED: unconventiona	847102432	XP_01281298	11.49	0.02	7.13	0.31	15.31	4.4	0	0	2.09	0	0	0
2-3.c56794/1/2394	DNA polymerase epsilon [Xe	11493740	AAG35631.1	0	7.14	0.4	0.1	0.98	1.22	0.07	0.09	0.33	0	0	0
1-2.c25105/2/1389	heterogeneous nuclear ribonu	147905111	NP_00108132	17.96	51.24	14.24	16.34	19.8	30.72	9.09	11.57	0	0	3.91	10.41
3-6.c6979/1/3014	PREDICTED: GSK-3-bindin	512846394	XP_00491520	0.39	0.2	0	0	0	0	0.26	0	0.14	0.86	1.91	0.69
1-2.c13856/1/1283	--	--	--	0.88	2.84	1.74	2.89	0	1.59	6.81	6.42	4.5	26.75	30.3	21.46
2-3.c34147/3/2254	transmembrane BAX inhibito	147899637	NP_00108928	0.04	21.49	0	0	0	0	0	0	9.79	3.6	2.41	0
1-2.c32562/2/1570	PMCA1bx [Rana catesbeiana	12963455	AAK11272.1	13.6	29.41	0.05	0	0.45	23.51	20.64	4.9	0	81.16	80.87	118.03
1-2.c2379/7/1573	PREDICTED: dimethyladeno	512842699	XP_00491468	0	2.11	1.48	1.98	1.25	4.31	0	1.38	0.78	0	0	0.41
2-3.c27589/1/2256	--	--	--	2.43	10.04	1.88	2.72	2.87	7.48	3.13	1.62	1	0.85	0.37	0.86
1-2.c38391/1/1234	--	--	--	20.33	0.36	3.88	17.2	4.71	14.25	29.44	15.25	23.38	159.2	219.92	100.31
2-3.c20226/1/2163	PREDICTED: protein Shroom	847100721	XP_01281266	2.52	1.03	0.68	0.85	1.09	1.52	0.84	0.09	0.11	13.33	14.84	24.86
2-3.c56465/1/2009.	uncharacterized protein LOC	148222510	NP_00108474	0	0.41	0	0	0	0	0	0	0	8.06	17	0
1-2.c26095/1/1421	PREDICTED: exosome comp	560908898	XP_00618076	2.4	17	2.57	3.27	3.82	6.94	2.47	1.73	2.27	0.81	0.62	0.25
2-3.c43426/1/2453	PREDICTED: tubulin polygl	847153386	XP_01282351	1.48	3.22	0.27	1.24	1.07	0.7	3.34	1.08	1.36	16.87	19.3	13.27
1-2.c41750/1/1608	PREDICTED: protein phosph	823396584	XP_01241593	0	15.36	0	0	1.5	2.6	0	0	0	0	0	0
2-3.c29258/1/1823	DNA replication licensing fac	148236609	NP_00107906	2.71	28.24	1.63	1.2	4.83	9.65	0.14	1.76	2.16	0.36	0.21	0.42
1-2.c20667/1/5740.	PREDICTED: basic leucine z	641711076	XP_00814469	138.49	102.22	49.2	37.6	84.76	63.19	43.46	32.51	45.13	38.67	0	2.86
1-2.c30567/1/1501	ubiquinol-cytochrome c reduc	147902934	NP_00107971	143.38	101.86	177.86	158.13	108.49	32.77	58.38	61.58	78.21	0	0	34.88
1-2.c36723/1/1293	Putative uncharacterized tran	449282618	EMC89440.1	0.28	0.19	0	0.19	0	0	0	0	0.32	8.87	11.23	0.5

1-2.c50474/1/1101	sodium/potassium-transportir	52346048	NP_00100506	68.83	25.1	34.39	28.33	25.95	10.15	5.37	30.05	14.96	4.82	3.17	6.24
2-3.c63373/15/2144	PREDICTED: glycogen phos	505849981	XP_00461844	0	0	0	0	0	0.18	12.1	0	17.35	12.23	17.03	0
2-3.c3061/1/2339.2	--	--	--	4.18	1.31	11.11	10.78	12.45	23.78	10.61	12.49	21.52	3	3.11	1.82
2-3.c15583/1/2134	UTP18 small subunit (SSU)	147898751	NP_00109005	9.63	34.02	5.64	6.44	8.6	15.88	8.85	5.69	5.35	0.92	1.29	4.03
1-2.c40948/1/1437	PREDICTED: glutamic acid-	780154032	XP_01168211	1.88	5.03	0.68	1.84	1.14	3.41	5.82	1.55	1.32	23.82	32.9	37.77
1-2.c27020/27/1611	PREDICTED: lupus La prote	591363494	XP_00705665	17.47	113.4	11.7	15.25	21.37	53.65	20.5	14.16	18.3	5.81	5.46	4.72
1-2.c9042/2/1267	--	--	--	13.26	14.64	8.08	11.04	10.69	14.46	8.74	7.82	8.71	2.45	3.17	1.65
2-3.c20037/1/2462	--	--	--	4.11	0	7.76	9.23	10.71	0	0	4.8	5.66	0.67	0	0
2-3.c29896/1/2588	--	--	--	17.72	4.29	17.74	16.21	10.85	7.72	11.33	10.61	19.49	4.42	2.56	1.83
2-3.c9385/1/2521.1	--	--	--	1.57	7.42	2.02	2.29	2.59	3.72	4.01	2.14	1.27	0.61	0.16	0
3-6.c4323/1/2780	PREDICTED: spectrin beta c	512862388	XP_00293694	11.86	20.75	10.75	5.68	7.87	19.11	4.59	7.09	9.47	1.71	3.33	1.78
1-2.c26338/1/1639	PREDICTED: endothelial lip	847089272	XP_01282219	0.78	3.89	1.69	0.12	3.44	3.57	0.71	0.26	0.7	0	0.31	0
1-2.c4860/1/1038	PREDICTED: family with se	847117708	XP_01281635	5.86	4.62	3.07	3.01	7.21	1.89	6.67	5.78	3.26	52.5	51.85	90.35
2-3.c39397/3/1976	aspartyl/asparaginyl beta-hyd	62751990	NP_00101573	2.19	5	7.64	5.18	7.38	24.27	5.01	5.65	6.68	1.02	0	0.86
1-2.c24835/1/1678.	potassium channel subfamily	187607255	NP_00112042	3.57	2.81	1.66	4.07	3.89	2.15	2.48	2.75	4.85	59.2	39.7	60.92
1-2.c45212/1/1917.	--	--	--	7.94	4.22	7.39	4.41	4.16	0.55	10.43	6.09	6.15	0	0	0
1-2.c35048/7/1569	histone-binding protein RBBI	89886120	NP_00101139	2.16	36.1	2.4	2.8	5.05	6.86	1.53	4.27	2	0	0	1.78
2-3.c27095/1/2698	PREDICTED: polypeptide N	847131447	XP_01281920	0	0	0.18	0	9.29	8.21	0	0.83	8.05	0	0.6	0
3-6.c18468/1/2862	formin-binding protein 1 [Xe	118404564	NP_00107266	3.04	0	4.17	3.76	4.45	1.56	1.69	2.31	0	0.58	0	1.02
3-6.c9383/2/3808	adaptor-related protein compl	147900121	NP_00108530	12.44	6.1	6.69	4.39	8.69	3.09	1.51	5.3	1.91	0.45	0.53	2.44
2-3.c60255/1/2399.	--	--	--	3.99	12.83	4.06	4.83	3.22	5.03	1.1	2.72	3.36	0.96	0.41	0.2
1-2.c11463/1/1346	PREDICTED: peroxiredoxin-	573894288	XP_00663489	136.18	110.62	64.32	135.68	115.27	45.48	312.23	89.82	96.65	1016.99	1570.37	1234.02
1-2.c29446/1/1669	PREDICTED: lamina-associa	847176929	XP_01281093	0	13.46	0	1.24	0	0.1	0	0	2.26	13.48	23.88	5.37
3-6.c16117/1/3156.	signal transducer and activato	148232026	NP_00108467	10.86	0.19	2.41	3.44	4.15	1.18	5.62	6.7	5.41	48.82	34.92	42.05
1-2.c14124/2/1303	Tubulin alpha-1C chain [Lari	808859777	KKF11870.1	13.29	0	0	1.29	0	12.4	18.55	4.02	7.23	51.97	83.35	42.01
2-3.c26785/1/2444	--	--	--	7.03	3.79	20.18	14.14	20.66	17.16	13.39	9.98	16.95	3.64	0.99	7.6
3-6.c4999/1/3536	collagen alpha-1(I) chain pre	58332412	NP_00101100	24.8	597.92	128.04	72.61	119.25	424.5	48.61	40.4	83.21	7.89	4.83	6.11
2-3.c24403/1/2592.	--	--	--	1.52	0	0	1.51	0.25	0	3.95	0.23	0	16.67	20.84	8.8
1-2.c7347/2/1466	--	--	--	0	0	0	0	0	0	0	0	0	3.14	4.16	0.7
3-6.c10550/1/3567.	RecName: Full=Sarcoplasmi	9789732	Q92105.1	1.48	0.36	2.41	2.34	3.13	1.72	2.38	2.52	1.77	0	0.16	0.35
2-3.c60674/1/4149	PREDICTED: protein phospl	512847586	XP_00491535	0	1.53	0	0	0	0	0.38	0	0	0	6.24	18.83
3-6.c14134/1/3117	PREDICTED: eukaryotic trai	734641821	XP_01074981	0	11.45	4.31	5.2	5.88	10.91	9.77	2.85	0	5.1	0	0
2-3.c25918/1/1985.	Unknown (protein for MGC:	120538440	AAI29682.1	2.02	0	0.67	2.27	3.61	0	13.52	2.19	12.23	34.61	17.57	65.16
3-6.c3223/2/2906	TGF-beta-activated kinase 1	147905935	NP_00109076	0	0.82	0.4	0.24	0.79	0.79	0	0.04	0.43	0	0	0
2-3.c2343/27/2592	PREDICTED: translation init	512840815	XP_00293518	8.96	17.07	3.86	4.2	6.65	16.54	4.64	3.63	0.79	0.62	0	2.58
2-3.c32721/1/10210	--	--	--	7.2	15.38	11.28	6.87	10.97	12.99	4.79	5.66	11.53	1.05	0.31	0.89
2-3.c5582/1/2418	--	--	--	25.27	31	24.02	16.68	20.16	23.17	18.11	16.21	22.04	4.61	3.3	6.97
3-6.c18530/1/3121	ZZ-type zinc finger-containin	678212196	KFV79175.1	0	3.67	0.61	0	0.79	1.52	0.63	0	0	0	0	0
1-2.c27491/1/1562.	--	--	--	20	6.9	5.59	16.03	5.1	2.39	24.95	15.54	13.83	115.93	148.12	111.47
2-3.c2615/19/1916	Unknown (protein for IMAG	76779686	AAI06679.1	34.89	25.35	29.66	17.56	20.45	6.42	61.19	13.02	20.89	3.61	3.54	3.99
1-2.c10827/5/1557	glycogen phosphorylase, brai	50054411	NP_00100190	11.83	20.26	10.48	53.84	32.92	34.33	32.79	15.81	41.81	15.55	9.55	7.61
1-2.c24777/1/1365	PREDICTED: glycogen debr	847125952	XP_01281792	8.55	2.6	5.89	23.68	15.21	9.21	14.32	13.53	30.41	4.36	4.88	2.27
2-3.c36416/2/2367	PREDICTED: PAB-depender	512822114	XP_00293457	0	3.62	0.74	0.7	0.97	0.59	0.83	0.52	0	0	0	0
2-3.c44825/161/250	dual specificity mitogen-activ	56118586	NP_00100805	55.27	66	50.67	18.34	41.85	53.37	16.05	19.6	16.67	7.43	5.7	15.75
2-3.c29832/1/2400.	--	--	--	0	0	0	0	0	0	0	0	0	25.55	0	13.95
2-3.c51277/184/224	PREDICTED: nucleolar GTP	512849296	XP_00293522	17.7	10.11	17.61	11.39	3.15	18.65	3.43	9.37	11.04	4.45	0	0
2-3.c8471/7/2213	pyruvate kinase PKM isoforn	118405080	NP_00107253	3.02	0.99	12.28	8.17	8.73	4.58	3.1	8.58	7.95	2.19	1.05	1.45
3-6.c16944/1/3404	tax1-binding protein 1 homol	45361339	NP_989247.1	0	0.18	0	0	0	0	0	0	0	1.77	0.2	1.01
1-2.c4143/14/1638	cleavage stimulation factor su	194332803	NP_00112370	0	0	0	0	2	1.45	0	0	0	0	0	0
2-3.c2868/11/2363	agmatine ureohydrolase (agm	147900416	NP_00108921	20.63	9.91	28.12	23.84	23.68	23.2	18.7	16.67	21.7	5.6	8.96	4.81
2-3.c54205/1/2507.	--	--	--	1.26	2.77	6.54	0.31	4.35	9.13	1.93	1.35	4.24	0	0	0
1-2.c36338/1/1758.	--	--	--	0	0	0	0	0	0.19	0	0	0	5.49	11.56	2.41
1-2.c33609/1/10505	PREDICTED: actin, alpha ca	327288034	XP_00322873	8832.52	1536	16941.28	12918.23	12995.92	9205.59	8650.7	13191.73	10153.76	2765.41	1491.55	5364.32
2-3.c1148/118/2211	glycine amidinotransferase (I	89272889	CAJ82904.1	0	39.92	0	10.35	0.23	54.9	3.64	0	1.8	0	0	0
3-6.c16496/1/2877.	--	--	--	62.92	26.18	8.6	32.24	22.07	13.52	39.22	17.36	23.53	299.74	571.26	197.29
1-2.c49811/1/1411	stearoyl-CoA desaturase (delt	147901446	NP_00108780	0	0.38	0.58	0	0.53	0.54	1.03	0.31	0.48	13.89	8.98	5.83
2-3.c50147/1/2073	--	--	--	4.84	6.59	4.98	8.34	4.2	2.01	21.2	5.58	5.94	68.62	48.27	76.4
2-3.c8493/2/1960	PREDICTED: ankyrin-1 isofo	847110220	XP_01281460	20.13	1.36	15.93	52.29	11.76	26.48	52.31	11.96	27.83	4.9	5.73	0

1-2.c27738/1/1672	PREDICTED: collagen alpha	664740187	XP_00852739	2.63	26.56	9.59	8.95	0.89	2.35	0.08	5.47	1.87	0	0	0
1-2.c7319/1/1713	reverse transcriptase, partial [	347227	AAA49027.1	2.75	6.28	1.9	1.14	1.02	1.73	3.86	1.37	0.74	18.91	24.21	27.58
1-2.c12407/5/1663	PREDICTED: protein SET is	847153649	XP_01282360	10.18	26.56	8.19	0.86	8.23	2.12	0.36	5.48	0	0	0	0
2-3.c11600/3/2314	--	--	--	3.94	29.36	3.23	1.69	2.28	16.43	0.87	2.08	0	0	0	0
3-6.c9838/1/4837	tetratricopeptide repeat protei	148224016	NP_00108657	2.5	3.25	1.54	1.07	1.39	3.45	1.03	1.13	1.01	0.52	0.21	0.27
1-2.c28173/1/1300	--	--	--	0	2.28	0	0	1.3	0	0.49	0	1.72	13.26	13.09	9.64
2-3.c14683/1/2360	--	--	--	0	2.24	0	2.09	1.19	0	0	1.1	0	0	0	0
1-2.c17756/1/1804	DBIRD complex subunit ZNI	349585177	NP_00101608	3.61	18.27	2.45	3.05	10.44	10.31	3.7	0.84	1.76	1.04	1.11	2.43
1-2.c34011/1/1250	alpha-actinin-3 [Xenopus (Sil	213983245	NP_00113551	831.69	182.88	1047.65	1126.8	1010.7	1265.15	1158.25	1057.01	1063.2	381.55	262.82	323.09
1-2.c36944/1/1370.:	--	--	--	5.08	70.4	10.8	13.27	4.78	5.32	28.89	23.31	27.77	187.65	110.38	63.32
1-2.c51854/1/1686	PREDICTED: serine--tRNA	602647281	XP_00743006	35.57	96.07	42.39	33.19	27.04	87.22	32.28	32.35	30.8	5.44	8.79	13.2
2-3.c36746/1/2056	PREDICTED: protein-tyrosir	847100370	XP_01281254	0	0	0	0.29	0	0	2.49	0	0.42	5.27	4.76	2.41
2-3.c42938/1/1892.	PREDICTED: saccharopine (	512842692	XP_00293153	4.95	28.43	4.51	1.11	6.93	7.71	0	4.77	0	0	0	0
3-6.c15981/1/2918	--	--	--	3.73	8.52	2.86	11.66	2.19	7.94	2.35	4.56	5.09	1.04	1.47	0.5
3-6.c5648/1/3103.2	PREDICTED: N-alpha-acety	602661561	XP_00743643	1.15	5.35	3.4	1.85	1.83	6.6	0.21	2.51	2.62	0	0.21	0.16
1-2.c2708/15/1716	PREDICTED: tubulin beta-5	558136461	XP_00611755	33.58	86.58	10.82	17.87	18.4	35.16	10.29	18.23	10.74	9.04	0.62	7.21
2-3.c9373/3/2072	OTTXETP00000002159 [Xe	89272520	CAJ83768.1	0.46	8.03	0	2.98	0.45	2.93	5.77	2.69	3.37	31.33	33.06	23.72
1-2.c21044/1/1472	PREDICTED: LOW QUALI	512849980	XP_00294276	3.58	4.48	0	0	0	0	3.05	2.62	0	5.93	11.61	0
2-3.c7732/3/2477	PREDICTED: spectrin beta c	512862388	XP_00293694	10.18	35.32	17.56	5.65	8.07	13.03	5.31	14.39	8.23	0	1.83	3.91
1-2.c13076/1/1464	--	--	--	0.65	4.74	0.68	0.83	0.35	1.23	3.46	0.41	1.9	14.16	23.17	7.83
2-3.c34489/1/2472	PREDICTED: myoferlin isof	847141448	XP_01282127	0	0.97	0	1.28	0.81	0	1.36	0.37	0	0	0	0
1-2.c18725/2/1523	PREDICTED: cytosolic 5'-nu	301624828	XP_00294170	11.79	1.21	6.47	25.58	22.34	6.9	13.39	9.63	12.5	2.68	6.04	5.19
2-3.c21510/1/2540	Nek2A [Xenopus laevis]	4760557	BAA77339.1	0	0.14	0	0	1.05	2.09	0	1.23	0	0	0	0
2-3.c42098/1/2682	PREDICTED: calcium unipo	847175711	XP_00492048	25.01	22.45	23.27	17.69	20.13	27.09	12.45	14.16	14.11	3.5	4.29	8.63
2-3.c25484/4/2544	RNA helicase II/Gu [Xenopu	147899364	NP_00108203	16.34	261.89	50.69	0.02	0.68	73.67	44.37	54.09	56.06	0	0	0
2-3.c62711/5/2197	ATP-binding cassette sub-fan	148226825	NP_00108764	0	0.16	16.24	22.28	0.14	0.06	22.55	19.37	15.16	0	0	0
1-2.c20946/1/1362	--	--	--	0	0.69	24.7	30.71	0	0.14	45.47	28.9	19.33	0	0	0
2-3.c36559/1/2016.	--	--	--	134.01	13.29	776.57	284.44	674.28	24.82	1306.57	1788.63	1198.09	22.36	21.22	26.28
1-2.c13270/1/1732	RecName: Full=Large prolin	317374854	A4IH17.1	0	0	10.53	14.48	0	0	13.53	14.03	14.17	0	0	0
3-6.c9618/1/5618	myosin, heavy chain 4, skelet	47575800	NP_00100124	1.97	0.22	20.25	10.61	8.56	0.06	23.81	13.03	16.14	0.54	0.23	0.04
1-2.c32537/27/1410	periostin precursor [Xenopus	163915007	NP_00110637	54.1	0	38.17	88.45	21.88	0	84.8	77.69	126.87	5.56	0	0
3-6.c9552/1/4253.1	myosin-binding protein C, ca	163915017	NP_00110637	63.23	2.21	97.85	211.78	138.55	0	147.26	102.2	186.53	0.88	0.24	9.23
1-2.c1981/40/1644	PREDICTED: 2-oxoisovalera	847152594	XP_01282325	0	21.68	10.46	11.49	0.22	0	10.81	8.03	9.47	0	0	0
3-6.c17820/2/3854	PREDICTED: myosin-4 [Xe	847156920	XP_01282446	1.01	0.06	9.24	5.7	3.2	0.07	12.48	8.18	8.83	0.01	0.07	0.46
2-3.c20612/1/2325	abhydrolase domain-containi	349501031	NP_00100677	0.12	0	3.52	3.35	0.07	0.08	4.71	5.09	6.07	0	0	0
2-3.c35777/1/2117.	--	--	--	0.79	86.91	0.65	0.45	0.66	26.77	0.85	0.17	0.96	121.57	51.14	135.76
1-2.c7335/1/1871	PREDICTED: reticulon-4 iso	847127971	XP_01281854	0	8.25	0	0	0	7.7	0	0	0	12.18	18.12	14.46
3-6.c19096/10/2961	PREDICTED: myosin-bindin	564233250	XP_00626099	130.77	3.9	241.13	247.47	119.81	0	201.39	133.05	167.88	9	5.22	7.3
3-6.c16742/1/2778	PREDICTED: myosin-bindin	701407466	XP_00999782	82.24	0.02	38.86	157.98	83.39	0	148.81	80.66	187.8	6.27	0	11.09
1-2.c52068/1/1916	PREDICTED: lymphocyte ar	301620254	XP_00293949	0	0.84	9.55	15.48	0	0.38	18.01	10.93	17.88	0.71	0	0.23
1-2.c42924/2/1448	PREDICTED: 2-oxoisovalera	847152594	XP_01282325	0.2	13.17	5.06	8.58	0.13	0	9.18	6.66	8.27	0	0	0
2-3.c35753/1/2219	--	--	--	3.25	0	16.92	8.72	1.18	0	4.78	8.59	7.54	0	0	0.16
2-3.c50575/1/2194.	nitrogen fixation cluster-like	147903789	NP_00108070	23.24	0.42	44.11	0.22	36.76	24.09	18.8	16.64	13.28	1.43	0	0
2-3.c13340/1/2000	PREDICTED: valine--tRNA	512859726	XP_00293951	0.05	0	3.69	3.89	0.04	0.01	9.03	4.38	3.02	0	0	0
2-3.c16992/1/1989	PREDICTED: lymphocyte ar	301620254	XP_00293949	17.52	53.92	9.34	8.43	32.88	140.84	14.73	4.22	8.97	466.67	597.46	570.38
2-3.c7869/1/2116	--	--	--	11.22	0.05	0.04	0.1	14.85	110.29	0.12	0.05	0.18	23.12	19.37	17.62
2-3.c1891/31/2009	kelch-like protein 41 [Xenopu	45361273	NP_989214.1	50.87	136.44	117.72	39.89	69.76	328.6	57.67	59.29	84.6	2.09	8.09	2.67
2-3.c33847/3/2274	--	--	--	2.94	5.09	6.96	3.63	5.74	47.04	3.81	4.18	6.05	0.09	0	0
1-2.c29294/1/1849	PREDICTED: tropoelastin 2	847162855	XP_01282565	186.5	1.72	80.1	157.23	42.1	0.39	174.4	288.63	212.35	21.7	4.32	19.68
2-3.c6342/1/2749	PREDICTED: ankyrin repeat	512839176	XP_00293509	37.23	3.03	13.37	13.78	21.54	0.65	13.25	25.4	22.89	0.88	1.6	0.97
1-2.c20717/1/1367	chaperonin containing TCP1,	148232533	NP_00108038	480.04	25.92	2514.53	823.93	1767.23	52.94	505.84	768.69	498.63	49.29	7.49	66.87
1-2.c20014/5/1404	PREDICTED: creatine kinase	847154911	XP_01282398	12.78	6.9	23.99	28.49	18.19	0.39	49.14	39.84	20.15	3.82	0	1.55
2-3.c9170/2/2393	LOC446249 protein, partial [	50418068	AAH78090.1	9	0.66	0.1	0	3.43	4.25	5.42	12.17	8.7	0	0	0.34
1-2.c7655/1/1844	--	--	--	12.27	0.59	38.15	34.77	7.98	0.27	17.7	34.27	27.83	3	0.85	0.5
1-2.c33303/3/1401	PREDICTED: valine--tRNA	512859726	XP_00293951	0.11	0	4.53	3.67	0	0	9.29	4.97	4.57	0	0	0
3-6.c16111/1/4289	myosin-binding protein C, ca	767806579	NP_990447.2	15.86	0	39.21	14.65	12.99	0	12.22	10.47	12.98	0	0	1.72
1-2.c17930/1/1384	plasminogen activator inhibit	47575766	NP_00100122	0	219.29	136.15	0.01	105.77	116.87	74.68	95.61	158.46	0.96	0	19.08
3-6.c8043/1/2777.1	uncharacterized protein LOC	194332743	NP_00112366	811.39	41.64	3809.8	1711.11	3050.95	72.73	3310.86	5812.12	3318.67	155.18	55.94	136.71

1-2.c25247/1/1321	RecName: Full=Large prolin	317374854	A4IH17.1	11.58	12.14	0	0	1.51	11.52	2.27	0	0	48.03	68.17	46.53
3-6.c17084/1/7453	myosin-binding protein C, ca	767806579	NP_990447.2	26.82	0.57	0.44	51.54	33.27	4.35	68.81	38.13	72.92	5.07	4.53	4.38
1-2.c31108/1/1528	PREDICTED: myosin-4 [Xen	847156920	XP_01282446	275.41	6.97	408.64	229.4	293.81	0.93	337.82	352.98	353.89	20.35	19.19	45.74
1-2.c20658/2/1592	--	--	--	0	13.18	0.64	43.04	3.5	0	10.99	8.45	27.18	0	0	0
3-6.c3344/2/3579	PREDICTED: myosin-4-like	301611451	XP_00293525	163.81	5.89	227.26	127.92	165.26	1.33	243.04	246.22	224.81	12.12	18.77	30.92
1-2.c48927/1/1618.	uncharacterized protein LOC	194332743	NP_00112366	1589.89	56.96	9491.02	3491.42	5942.57	93.13	2876.26	5086.85	3116.36	189.16	25.39	265.38
2-3.c51093/3/2154.	PREDICTED: beta-enolase [	637381761	XP_00812393	1073.05	66.64	2429.69	4350.59	2514.65	638.64	1872.93	1860.06	3596.95	234.32	12.63	361.6
3-6.c15132/1/2685	PREDICTED: myosin-4-like	301611451	XP_00293525	1.64	0.26	16.6	10	6.79	0	13.3	6.78	7.39	0.44	0.44	0.37
1-2.c48018/53/1468	PREDICTED: beta-enolase [	637381761	XP_00812393	140.16	0	22.06	90.19	58.17	18.3	431.92	242.14	218.92	53.03	32.88	0.2
2-3.c26384/1/2382.	PREDICTED: ankyrin repeat	512839176	XP_00293509	8	2.07	2.45	3.69	4.43	0	4.06	4.75	7.76	0	0	0.28
1-2.c40274/1/1440	--	--	--	0	5.63	3.18	1.56	0	0	0	0	0	13.16	15.91	6
2-3.c13240/1/2285	myosin, heavy chain 2, skelet	55742222	NP_00100680	17.78	0.63	25.99	12.1	21.05	0.22	29.9	36.01	21.97	1.51	2.28	2.94
2-3.c24309/1/2344	--	--	--	1.27	64.39	2.21	1.16	0.55	19.51	0.35	0.75	1.43	38.56	31.54	60.53
1-2.c50845/5/1507	--	--	--	15.76	4.95	21.26	93.46	42.83	4.98	82.1	64.86	71.27	9.84	0	7.28
2-3.c17603/1/2111	abhydrolase domain-containi	349501031	NP_00100677	0	0	3.81	5.84	0.02	0	3.01	3.23	3.33	0	0	0
2-3.c9545/1/2343.2	--	--	--	48.73	10.5	5.84	7.33	0	0	0	0	0.12	36.33	30.48	12.84
1-2.c35180/1/7192.	uncharacterized protein LOC	194332743	NP_00112366	3719.61	149.71	17741.66	7230.35	12951.88	229.82	6655.07	10108.39	5762	709.5	127.96	678.45
3-6.c13888/1/2922.	PREDICTED: myosin-bindin	733889284	XP_01070973	47.33	0	58.07	148.22	59.79	0	116.93	62.97	120.7	5.69	4.46	16.67
1-2.c12422/1/1659	--	--	--	3.06	20.99	31.07	17.76	24.77	106.5	53.14	18.94	34.69	2.95	0.68	3.33
3-6.c2723/1/3922	myosin, heavy chain 4, skelet	47575800	NP_00100124	0.27	0	3.45	1.18	0.74	0	3.48	1.56	1.3	0	0	0.06
1-2.c18770/1/1544	Unknown (protein for MGC:	213626057	AAI70388.1	46.02	0	45.97	63.79	16.63	0	23.67	25.77	37.59	3.56	0	2.6
2-3.c17128/1/2579	PREDICTED: myosin-4 [Xen	847156920	XP_01282446	158.54	61.85	92.26	40.97	67.32	8.62	58.63	71.68	55.98	7.18	11.48	2.49
2-3.c54300/1/2079.	probable C->U-editing enzym	55741924	NP_00100672	38.82	61.75	80.95	35.88	50.39	267.75	31.3	41.57	50.88	3.33	2.99	3.41
2-3.c27739/1/1741	breast cancer anti-estrogen re	147906605	NP_00108519	2.35	2.59	0	0	2.77	1.13	3.05	4.85	2.1	0	0	0
3-6.c3860/2/4633	myosin, heavy chain 4, skelet	47575800	NP_00100124	1.44	0	5.28	1.73	2.39	0	3.27	4.48	3.5	0.16	0	0.39
3-6.c3365/2/3324	collagen, type 1, alpha 2 prec	147898763	NP_00108072	11.8	161.14	43.36	39.17	41.65	315.78	46.06	31.92	50.46	5.99	3.6	3.85
2-3.c20093/1/1862	PREDICTED: lymphocyte ar	301620254	XP_00293949	0.11	0	3.3	6.96	0	0	9.4	3.63	5.91	0.32	0	0.18
1-2.c3756/7/1301	APG4A protein [Xenopus lae	213626921	AAI70397.1	12.64	1.78	3.48	1.29	11.22	0.16	4.15	4.06	7.02	0	0	0
2-3.c24303/1/2299.	PREDICTED: beta-enolase [	637381761	XP_00812393	297.14	25.9	869.63	1540.29	876.68	182.03	761.25	586.06	1184.84	135.2	34.09	116.47
1-2.c45413/1/1426	mitochondrial 2-oxoglutarate	71895777	NP_00102568	126.01	35.02	140.03	151.66	73.17	23.96	46.04	83.39	82.77	5.92	4.17	11.84
3-6.c3464/2/3022	PREDICTED: collagen alpha	847166754	XP_01282667	3.75	52.7	8.23	5.39	0	17.95	1.67	4.19	2.62	0	0.12	0
2-3.c7871/6/2737	PREDICTED: CD99 antigen-	301623490	XP_00294104	12.81	0	0	8.94	0	0	9.38	13.48	11.34	0	0	2.39
1-2.c16487/6/1579	--	--	--	0	0	0	0	0	4.72	0	0	0	9.01	11.55	5.76
2-3.c10107/21/2517	Unknown (protein for IMAG	197246230	AAI68800.1	325.06	6.02	230.65	493.05	307.98	29.68	181.81	262.7	316.14	18.71	14.78	55.95
1-2.c18043/3/1368	PREDICTED: complement c	512820694	XP_00293686	6.46	0	0	0	6.17	0	10.74	8.01	4.41	0	0	0.46
2-3.c25818/1/2190.	PREDICTED: collagen alpha	847138882	XP_01282086	13.32	0.49	29.96	29	5.58	0	17.46	31.85	25.63	3.46	0.2	1.52
2-3.c36786/1/2098.	uncharacterized protein LOC	148232016	NP_00109117	380.96	12.62	82.59	291.34	298.11	28.71	161.1	305.04	344.83	0.01	103.79	2.77
1-2.c21168/1/1364	LOC100037225 protein [Xen	125858810	AAI29772.1	77.37	81.54	116.02	76.96	23.39	20.13	40.92	84.02	58.49	5.52	2.41	3.47
1-2.c1846/27/1308	PREDICTED: tenomodulin [	847171214	XP_01280901	46.74	172.8	65.57	24.49	20.02	101.73	21.85	31.88	31.56	2.33	3.9	1.72
1-2.c48429/1/1775	PREDICTED: myosin-bindin	524998837	XP_00504717	78.86	0	26.47	151.89	82.64	0	92.12	60.18	133.48	18.63	5.45	9.76
1-2.c18849/1/1796	phosphatidylethanolamine bi	148232176	NP_00108562	0.14	1.86	1.36	0	1.4	4.67	3.64	1.85	3.9	0	0	0
2-3.c22369/1/2361.	PREDICTED: mRNA-decapp	512826563	XP_00293814	0	0	0	4.08	0	0.56	0	0	0	4.76	12.29	5.5
2-3.c36559/1/2016.	uncharacterized protein LOC	194332743	NP_00112366	793.38	47.57	4469.58	1495.21	2854.04	73.99	2694.28	5840.58	2881.9	59.21	20.36	90.88
2-3.c39771/1/2258	--	--	--	0.65	0	0	2.76	0	0	0	0	0	6.7	2.03	6.48
3-6.c13952/1/5500.	PREDICTED: myosin, heavy	847172850	XP_01280963	32.06	4229.42	30.68	35.28	27.17	24866.5	29.21	19.58	26.62	4.86	3.38	0.56
1-2.c29423/2/1350	--	--	--	2	0	0	0	0	10.68	0	0	0.51	31.73	39.02	12.23
1-2.c22130/1/1428	--	--	--	0	0	2.45	2.95	0	0	4.58	3.43	3.88	0	0	0
1-2.c4006/1/1598	PREDICTED: nebulin isofor	847166508	XP_01282661	50.43	26.36	4.33	64.49	0	0	24.23	15.41	29.38	1.84	0.9	3.27
2-3.c54140/1/2109	PREDICTED: myosin-4 [Xen	847156920	XP_01282446	824.62	26.86	884.1	804.22	701.34	6.04	1045.01	1111.32	944.94	133.92	133.74	161.83
1-2.c52026/3/1548	--	--	--	21.36	72.74	34.59	22.57	12.39	68.07	21.17	31.97	31.19	5.91	1.33	1.53
2-3.c36849/1/2078	PREDICTED: target of Nesh	847100396	XP_01281255	3.79	20.24	4.34	4.53	4.03	30.75	3.46	4.74	5.97	0	0.24	0.21
2-3.c42999/1/2072	nicotinamide nucleotide trans	148231869	NP_00108770	37.76	29.97	0.38	58.81	0.47	0.19	25.56	51.04	24.97	2.35	0.36	0
2-3.c50513/2/2030	myosin heavy chain, partial [	4249697	AAD13770.1	0	0.32	12.89	4.77	4.82	0.09	9.44	2.7	5.05	0.47	0	0
3-6.c5852/1/3083	claudin 4 [Bufo japonicus]>g	407031622	AFS68371.1	19.09	4.96	0	17.52	0	0	20.82	24.89	12.23	0	0	5.93
2-3.c36986/3/2484	PREDICTED: collagen alpha	591360730	XP_00705528	4.76	49.33	9.29	4.01	4.87	28.25	3.11	5.12	7.34	0	0	0.71
2-3.c34195/1/2653.	PREDICTED: G0/G1 switch	704472928	XP_01007157	4.82	30.19	6.25	18.76	14.17	29.66	9.21	5.03	8.09	225.84	218.83	242.59
3-6.c16458/1/6542	myosin, heavy chain 4, skelet	47575800	NP_00100124	2.15	0	9.17	5.51	4.8	0	9.26	5.81	5.19	0.46	1.29	0.18
2-3.c6649/1/1974	solute carrier family 41, mem	147907304	NP_00108547	35.69	51.07	55.37	26.46	34.22	122.96	49.8	36.55	56.11	7.27	6.28	4.45

3-6.c18298/1/3309	calpain 1, large subunit [Xeno	61889135	NP_00101363	9.28	0.63	3.62	0.72	8.44	6.03	2.79	6.33	5.89	0.68	0	0.38
2-3.c19688/1/2393.	isovaleryl-CoA dehydrogenas	58332612	NP_00101138	23.37	8.86	11.14	8.35	0.66	3.2	11.1	9.63	13.42	0	3	0
1-2.c25617/1/1485	--	--	--	0	3.09	0.22	0.43	0.39	3.3	0.25	0	0.74	18.81	20.3	18.88
1-2.c50742/1/1284	periostin precursor [Xenopus	163915007	NP_00110637	28.17	0	0.13	36.71	4.77	0	28.6	33.62	29.92	0.96	4.21	4.81
2-3.c32515/1/2162	PREDICTED: H/ACA ribonu	602655291	XP_00743339	16.93	0.41	14.13	0	12.32	29.89	14.19	15.4	11.74	1.88	2.91	0
1-2.c17584/1/1489	PREDICTED: tropoelastin 2	847162847	XP_01282565	168.73	3.63	71.73	96.93	56.63	1.01	176.95	252.61	185.74	33.72	30.53	29.83
1-2.c48185/2/1779	PREDICTED: myosin-4 [Xen	847156920	XP_01282446	923.63	27.09	983.84	1013.07	816.66	7.54	1399.17	1326.36	1163.81	206.48	196.08	206.34
2-3.c62798/10/2219	PREDICTED: integrin beta-l	512821557	XP_00293713	8.75	0	8.71	0.5	3.51	0	3.56	4.88	4.77	0.38	0.22	0
1-2.c13485/3/1428	--	--	--	12.02	2.29	0	3.34	0	9.62	3.76	9.38	11.6	1.14	0	0
1-2.c37153/7/1450	--	--	--	6.84	50.48	7.94	1.17	4.01	48.79	8.97	5.61	6.47	0.84	0	0
1-2.c50366/1/1808.	uncharacterized protein LOC	147906248	NP_00108473	0.48	14.39	0.95	1.08	0	0	0	0	0	6.74	3.22	8.32
1-2.c8908/13/1602	PREDICTED: pyruvate dehy	530574464	XP_00528162	14.82	3.43	27.86	8.2	22.73	4.29	14.92	10.13	12.21	1.4	0	1.56
1-2.c10740/6/1348	glioma tumor suppressor canc	148228700	NP_00109002	79.3	124.94	66.51	82.23	0.95	0.18	90.74	75.73	65.43	0.35	0	28.21
2-3.c41897/1/2274	myosin, heavy chain 2, skelet	55742222	NP_0010068(	12.17	6.39	8.42	2.7	6.99	1.29	5.66	7.35	2.85	0.38	0.54	0
1-2.c19900/1/1332	ectonucleotide pyrophosphata	147901695	NP_00109016	1.5	35.65	0	0.08	0	8.58	1.32	0	1.33	39.55	24.96	34.92
3-6.c15820/1/2998	PREDICTED: myomegalin-li	847014351	XP_01280435	17.05	30.46	21.62	9.37	23.59	57.5	15.04	22.6	12.13	2.9	1.66	2.03
2-3.c45527/1/2271	PREDICTED: myosin-4 [Xen	847156920	XP_01282446	1.88	0	8.46	7.68	7.92	0	5.84	8.44	7.47	0.43	0.59	0.74
1-2.c13297/4/1763	PREDICTED: chordin-like pr	847153124	XP_01282342	13.32	1.35	16.27	4.64	11.3	1.54	7.99	5.5	7.1	0.02	1.23	0
2-3.c11963/1/2084	--	--	--	0	0	0	0.04	3.13	3.53	3.15	4	3.86	0	0.43	0
1-2.c34152/1/1435	LOC100101331 protein [Xen	148921625	AAI46634.1	6.06	0.01	5.82	0	0.32	0.46	0	0	0	8.77	4.69	7.81
2-3.c19423/1/6971.	PREDICTED: beta-enolase [/	637381761	XP_00812393	775.67	71.59	1492.86	3194.74	1659.55	453.69	1279.76	1126.69	2261.9	299.76	117.16	325.19
3-6.c19043/1/2909	myosin, heavy chain 4, skelet	47575800	NP_00100124	0.98	0.37	4.91	2.29	1.75	0	5.21	2.59	1.86	0.19	0	0.2
2-3.c55363/1/2084	--	--	--	2.98	5.97	5.02	0	2.9	5.81	2.37	2.32	4.12	0.14	0	0
3-6.c18354/1/3309	PREDICTED: myozenin-2 is	847087249	XP_01281675	15.39	29.16	25.28	10.41	12.96	40.92	19.05	20.58	18.77	3.88	1.94	2.62
2-3.c5846/1/2071	PREDICTED: protein Wnt-5a	821108369	XP_01237937	0.2	0.02	0	0	0	0.99	0	0	0	3.93	2.92	6.94
1-2.c7623/1/1812	--	--	--	0	0	2.81	5.07	0	0	2.78	2.64	4.15	0.1	0	0
2-3.c33743/1/2566	--	--	--	0	0	0	1.25	0	0.22	0.01	0	0	3.83	2.75	4.09
2-3.c9413/1/1914	PREDICTED: tropoelastin 2	847162839	XP_01282564	49.39	4.05	43.77	99.91	9.97	0.75	71.95	85.19	110.08	6.56	3.22	29.09
1-2.c17576/1/1462	PREDICTED: LOW QUALIT	696973019	XP_00955636	0	0	0.08	5.57	4.05	0	0.01	0	0	35.38	30.37	25.36
2-3.c51195/2/2196	PREDICTED: glycogen debr	847125942	XP_01281792	27.27	2.06	18.7	57.86	34.38	8.89	29.2	29.82	60.81	6.33	4.6	5.75
3-6.c15947/1/3380.	myosin, heavy chain 2, skelet	55742222	NP_0010068(	64.29	34.14	33.34	13.42	26.32	2.24	18.56	33.66	15.09	1.92	0	0.21
2-3.c55224/1/2053	PREDICTED: glucocorticoid	847109993	XP_01281452	2.36	3.22	2.64	0.84	0	3.03	3.6	1.71	1.7	0	0	0
2-3.c60141/1/2047	MGC84135 protein precursor	148228231	NP_00108619	0	0.69	0	0	1.24	5.52	0	0	1.45	22.34	22.49	10.77
2-3.c59709/1/2634.	uncharacterized protein LOC	147905311	NP_00109060	0.11	2.37	0	0	2.48	3.27	0	0.32	0	6.12	10.65	17.08
2-3.c20067/2/2196	PREDICTED: collagen alpha	701292594	XP_01002219	9.08	191.84	22.02	10.03	16.35	113.54	16.49	26.26	24.81	8.69	0	1.04
3-6.c12471/173/377	RecName: Full=Sarcoplasmic	9789732	Q92105.1	905.22	371.15	591.84	519.54	696.89	642.54	1345.42	860.84	978.31	64.09	0	421.88
1-2.c1272/230/1630	uncharacterized protein LOC	148232016	NP_00109117	1073.96	32.41	119.87	566.2	477.44	45.17	1186.48	775.36	705.34	134.53	282.93	77.8
1-2.c20135/1/1549	PREDICTED: myosin-3-like	847172866	XP_01280963	2.15	477.2	2.58	3.27	2.49	2904.74	3.45	2.35	4.26	0.04	0	0
3-6.c13190/2/3804	RecName: Full=Alcohol dehy	113364	P22797.1	7.47	33.41	16.55	3.21	6.17	26.46	2.03	3.75	3.73	0.37	0	0.3
1-2.c13194/1/1379	epiphycan precursor [Xenopu	54020763	NP_00100562	5.25	18.24	18.74	14.52	2.86	2.09	7.94	20.31	14	1.18	0.49	0.28
2-3.c39649/1/2357.	myosin heavy chain, partial [	4249697	AAD13770.1	56.73	4.86	763.91	469.41	243.54	6.45	924.67	238.3	344.31	4.43	6.61	4.16
1-2.c16610/3/1383	--	--	--	1.04	1.52	0	1.17	0	0	0	0	0	9.06	4.42	10.12
1-2.c30525/1/1433	myosin heavy chain, partial [	4249695	AAD13769.1	62.53	0.95	78.09	92.62	60.86	0.42	156.09	118.72	117.27	19.41	26.01	20.39
1-2.c49882/1/1439	--	--	--	15.42	0.32	38.31	32.64	10.72	0.4	28.14	35.77	39.53	7.19	5.4	2.4
1-2.c28080/1/3919.	glyceraldehyde-3-phosphate c	300679438	ADK27487.1	24689.39	1262.15	19346.98	20729.1	26959.91	3442.12	15946.37	20298.43	23019.78	2990.8	1041.54	5625.32
1-2.c52493/1/1209	myosin heavy chain, partial [	4249703	AAD13773.1	34.55	4977.22	31.49	46.69	34.37	29892.13	34.22	21.69	27.92	7.25	2.6	0.64
2-3.c29621/1/4590.	PREDICTED: uncharacterize	847117050	XP_01281612	0	1.13	0	1.74	0.41	0.03	0	0.43	0	7.38	12.02	10.73
3-6.c16357/3/3055	LIM domain binding 3 [Xenc	147903867	NP_00108023	1.98	10.76	17.31	15.25	23.46	92.06	34.28	17.06	19.79	1.44	0.2	7.84
3-6.c6802/2/3280	PREDICTED: DDB1- and CU	512862186	XP_00293537	7.22	9.68	1.14	4.36	5.05	0.7	6.51	3.37	7.69	0.32	0.35	1.01
1-2.c51337/6/1392	PREDICTED: pyruvate kinas	564240659	XP_00627715	231.32	0	329.54	400.08	292	0	269.39	304.27	422.81	73.5	0	91.56
2-3.c49297/1/2496	solute carrier family 38, mem	147900233	NP_00108707	3.83	6.68	4.65	4.35	25.92	0.72	8.59	2.86	7.91	0.54	0.7	0.5
1-2.c37690/6/1591	collagen, type II, alpha 1, par	374533626	AEZ53726.1	18.03	445.47	109.73	54.12	20.08	152.45	53.83	52.07	70.07	13.52	8.97	6.8
1-2.c45632/2/1804	PREDICTED: alpha-actinin-3	558140024	XP_00611831	47.45	37.69	28.54	23.12	10.43	27.62	155.44	83.92	147.63	25.51	4.54	32.77
3-6.c2417/1/3782	PREDICTED: DNA-directed	291241363	XP_00274058	1.54	1.55	1.63	0.5	0.95	0	1.43	1	0.61	0	0	0
1-2.c40213/1/1738	Phytanoyl-CoA hydroxylase-	678148140	KFV17291.1	0.69	4	1.01	1.49	1.06	6.56	1.09	1.24	1.55	21.92	54.13	39.61
1-2.c35594/1/9297.	PREDICTED: myosin bindin	847128061	XP_01281857	48.57	29.47	0	93.81	154.24	204.73	306.69	220.07	259.38	69.77	43.74	19.14
1-2.c34119/1/1516	unnamed protein product [On	642019941	CDQ95350.1	12.16	19.58	0.92	39.53	31.97	29.58	106.2	78.11	59.63	24.16	3.5	12.87
1-2.c30374/1/1204	PREDICTED: probable E3 ul	847147395	XP_01282233	0	0	0	0	0	2.13	0	0	0	19.59	24.15	3.89

2-3.c30410/1/6158.	PREDICTED: myosin-4 [Xei	847156920	XP_01282446	18.99	4.38	608.76	428.14	200.75	2.78	811.68	251.43	323.14	13.67	15.17	12.6
2-3.c61486/3/2434	periostin precursor [Xenopus	163915007	NP_00110637	11.96	0	16.68	12.97	13.49	0	9.91	9.32	7.25	1.65	0	1.22
2-3.c25467/2/2494	--	--	--	1.43	0.11	5.06	1.89	1.32	7.72	4.33	4.25	3.08	0.35	0.12	0.24
1-2.c29603/1/1517.	troponin T [Hyla chrysoscelis	69219560	AAZ04163.1	1184.56	155.77	2502.06	2198.22	1677.28	119.94	2669.35	2302.93	1995.19	420.4	300	553.63
1-2.c4923/1/1137	tropomyosin alpha, partial [H	69219511	AAZ04161.1	111.27	11.23	251.79	208.96	167.27	30.26	108.37	175.79	139.61	34.78	30.56	15.07
1-2.c24376/3/1290	tyrosine 3-monooxygenase/tr	89515082	ABD75373.1	42.51	66.77	24.47	66.42	0	0	65.2	18.95	48.65	0	0	11.18
2-3.c58783/1/2350.	--	--	--	0.21	3.68	0.34	50.86	0	0.1	0.34	100.07	95.65	0	0.08	0
3-6.c17965/1/3534	PREDICTED: myosin-4 [Xei	847156920	XP_01282446	2.95	0	5.99	5.11	4.13	0.05	4.51	6.08	4.85	0.32	1.02	0.53
1-2.c34265/1/5396.	larval type I keratin [Rana cat	134140868	ABO61147.1	1941.06	462.95	1476.77	1202.38	772.7	216.62	1301.2	1671.4	1444.84	252.9	383.68	240.83
3-6.c15288/3/6232.	PREDICTED: myosin-4 [Xei	847156920	XP_01282446	2.07	0	12.67	10.39	8	0	9.62	6.84	7.64	1.69	0.18	0.29
2-3.c35770/1/2386	PREDICTED: collagen alpha	591360726	XP_00705528	0.01	36.14	9.07	5.12	4.03	23.67	5.67	4.38	5.26	1.28	0	0
1-2.c50267/3/1430	PREDICTED: sarcalumenin i	512868459	XP_00491808	5.43	3.4	7.65	8.36	1.71	2.22	7.34	7.91	5.24	1.29	0	0
1-2.c25674/1/1296	PREDICTED: glypican-5-like	301607624	XP_00293342	0	33.51	0	0	0	14.31	3.26	0.28	0	34.3	47.08	22.75
2-3.c41073/1/2023	PREDICTED: ubiquitin carbo	847100581	XP_01281262	1.53	0	0	0.65	1.92	0	2.84	1.57	1.46	0	0	0
1-2.c20368/1/1284.	--	--	--	0.22	0	0	0	0	13.35	0.18	0.32	0	171.89	257.6	36.11
2-3.c61166/1/2522	PREDICTED: nuclear recepto	847100804	XP_01281269	2.38	10.43	0.71	1.09	2.56	2.16	2.36	5.17	6.69	0	1.45	0.01
1-2.c34000/1/5930.	PREDICTED: sarcalumenin i	847166841	XP_01282670	61.89	0	34.47	17.69	84.17	0	92.9	96.32	69.99	17.97	17.18	13.99
2-3.c29850/1/2612.	--	--	--	0	0	4.57	1.56	0	0	8.7	3.87	8.15	0	0	0
1-2.c13847/1/1263	uncharacterized protein LOC	187608167	NP_00112012	17.32	1.7	18.21	17.78	18.18	2.61	14.45	18.68	22.04	2.03	3.19	1.44
3-6.c10833/1/3027	PREDICTED: collagen alpha	591360726	XP_00705528	0.38	8.78	2.96	1.08	0.58	9.12	1.4	2.2	1.71	0.17	0	0
3-6.c18811/1/2977	myosin, heavy chain 4, skelet	47575800	NP_00100124	0.52	0	1.89	0.65	0.18	0	2.34	1.64	1.15	0	0.12	0
2-3.c42079/1/2305.	PREDICTED: tyrosine-protei	847094360	XP_01281088	1.45	11.69	0.47	0	0.26	2.24	0.33	0.32	0	9.79	9.72	12.86
1-2.c6800/1/1622	nuclear export mediator facto	537152771	ERE72278.1	0.86	2.94	0.81	0.81	1.29	0.92	0	0	0	2.42	6.21	7.84
1-2.c35872/1/1500.	--	--	--	0.6	6.43	0.61	75.29	0.9	0	0	157.57	143.33	0	0	0
1-2.c19159/1/1431	PREDICTED: large neutral a	532055639	XP_00537053	52.1	0	24.6	75.8	91.95	0	47.21	35.05	44.51	0	0	17.83
1-2.c6516/1/1608	--	--	--	0.89	64.35	1.44	1.35	0.95	24.75	1.68	1.08	1.86	33.81	40.04	43.47
2-3.c32705/1/2033	PREDICTED: myosin-4 [Xei	847156920	XP_01282446	0.96	0	7.3	5.21	3.57	0	5.92	2.79	3.37	0.44	0	0.13
1-2.c31601/6/1344	PREDICTED: NAD(P) transl	706104795	XP_01019849	10.89	9.91	10.32	13.6	3.87	0.29	17.23	7.74	5.79	1.84	0	0
1-2.c43348/1/1359.	PREDICTED: nuclear recepto	847100804	XP_01281269	35.13	177.77	39.42	17.14	110.55	19.69	21.57	28.02	51.39	6.69	3.51	4.27
2-3.c20492/1/2499	PREDICTED: general vesicu	512815393	XP_00491113	0	0	0	0	0	1.63	1.53	1.86	0.94	0	0	0
2-3.c26550/1/4673	--	--	--	11.13	12.99	19.13	11.19	13.12	1.97	20.24	6.25	11.72	1.91	0.15	1.51
3-6.c3337/6/3018	protein unc-45 homolog B [X	297591951	NP_00117205	2.73	10.39	1.14	0.15	1.17	30.67	2.03	0.37	1.32	0	0	0
1-2.c6890/1/1639	PREDICTED: dnaJ homolog	847134514	XP_01281987	1.92	0	2.17	3.17	2.91	0	0	0	0	12.81	17.34	2.6
2-3.c27188/1/2344	PREDICTED: target of Nesh	847100396	XP_01281255	0	14.98	4.72	0.63	1.9	18.14	1.13	1.52	2.08	0	0	0
1-2.c35872/1/1500.	--	--	--	0.51	3.1	0.26	79.08	0.22	0	0.66	131.75	129.01	0	0	0
3-6.c9671/2/3157	RecName: Full=Aquaporin F	1703359	P50501.1	5.93	12.67	4.04	5.48	11.41	22.39	0.36	0.59	3.08	35.77	14.63	34.64
1-2.c35227/1/8313.	RecName: Full=Actin, alpha	113288	P10994.1	0	0	0	35.65	0	0	0	0	0	109.09	18.75	62.51
2-3.c59770/1/2338.	PREDICTED: phosphorylase	847163876	XP_01282599	0	0.88	2.53	2.23	1.22	3.04	0	0	0.44	13.87	18.88	8.56
2-3.c60695/1/2472.	PREDICTED: mitochondrial	326666498	XP_00266141	3.06	10.59	2.03	1.83	1.97	10.3	3.31	3.5	7.62	0.33	0	0.56
3-6.c8576/1/3834	PREDICTED: myosin-4 [Xei	847156920	XP_01282446	1.19	0	2.4	2.14	1.85	0	3.11	2.74	1.95	0.21	0.2	0.24
1-2.c23378/1/1329.	PREDICTED: mitochondrial	301626778	XP_00294256	42.23	1.96	22	33.3	50.02	1.84	87.88	36.86	45.46	2.5	9.31	15.07
2-3.c45849/1/2087	--	--	--	36.67	10.82	29.98	21.14	20.09	4.46	19.74	31.12	30.17	4.56	3.51	6.34
3-6.c7451/1/2842.1	RecName: Full=Collagen alp	18202034	O42350.1	39.08	1297.31	200.62	131.64	129.35	561.17	53.28	57.55	120	11.87	8.91	11.38
1-2.c41428/1/1311	--	--	--	0	0	0	0.84	0	0	0	0	0	9.3	4.11	6.46
2-3.c63592/1/2016	alpha 1 type I collagen [Rana	3242649	BAA29028.1	158.82	4023.54	525.27	297.81	297.79	3160.37	396.06	236.51	483.38	120.1	44.86	65.1
3-6.c5318/1/3951.1	PREDICTED: endogenous re	831566752	XP_01273347	0	0	0.66	0	0.36	1.68	2.57	1.4	1.18	0	0	0
1-2.c43062/1/1671.	Htra1 protein [Xenopus laevis	56270034	AAH87471.1	1.04	0	0.9	2.35	3.27	7.15	2.59	0.25	0.77	42.67	40.94	20.82
2-3.c37366/1/2450	hypothetical protein LOTGIE	676430378	XP_00904590	2.13	0.51	0	1.46	0	0.27	0	0	0	1.46	4.12	3.61
3-6.c3238/2/2848	kelch-like protein 30 [Xenopu	156717430	NP_00109625	8.75	15.34	8.94	5.03	7.65	42.11	5.26	5.83	5.39	0.37	0.99	0.67
3-6.c16484/1/4818	PREDICTED: transmembran	847095050	XP_01281107	4.04	2.7	1.77	0.19	1.99	2.1	2.55	3.77	4.59	0	0	0.54
1-2.c49046/1/1592.	--	--	--	1.98	22.7	2.06	2.51	2.22	15.68	4.88	2.76	2.6	96.56	77.74	67.24
3-6.c18821/1/2932	PREDICTED: myosin-4 [Xei	847156920	XP_01282446	1.86	0	4.81	3	2.85	0.04	4.49	3.84	3.09	0.44	0.26	0.33
1-2.c19370/1/6020.	PREDICTED: nuclease-sensi	532064616	XP_00531817	0	0	0	50.55	0	0	0	154.67	157.98	0	0	0
1-2.c17348/3/1795	PREDICTED: asparagine--tR	512809930	XP_00293448	18.99	0.77	0.09	0.22	4.51	22.53	0.36	0.15	0.18	15.26	35.97	14.99
1-2.c1947/17/1359	calsequestrin-2 precursor [Xe	45360999	NP_989136.1	218.79	59.46	8.03	214.68	268.29	46.09	230.92	439.71	294.23	27.91	51.06	84.85
2-3.c33303/2/2304	ryanodine receptor beta isofo	29501272	BAA04647.2	3.12	3.31	21.12	10.17	12.55	14.99	18	15.5	21.8	6.68	0.61	2.27
3-6.c5570/1/3274	--	--	--	9.54	0.49	7.44	15.82	15.99	1.11	4.39	5.93	10.65	1.66	0.64	0.8
3-6.c16332/1/3513.	myosin, heavy chain 2, skelet	55742222	NP_00100680	31.49	5.09	15	6.94	18.52	0	8.48	7.15	15.56	1.07	0.65	0.59

1-2.c1161/47/1713	phosphoglycerate kinase 1 [X	62858027	NP_00101654	661.22	64.4	659.36	999.62	716.43	195.89	582.27	614.93	833.03	163.41	82.15	187.49
2-3.c393/429/2195	transketolase-like protein 1 [	45361299	NP_989227.1	60.71	544.77	51	26.02	50.26	169.91	32.76	39.15	42.19	6.81	4.9	10.36
2-3.c51503/2/2736	periostin precursor [Xenopus	163915007	NP_00110637	3.44	0	47.18	15.01	22.68	2.63	22.37	7.48	10.2	0	0	2.62
1-2.c37398/1/1661	PREDICTED: 6-phosphofruc	594036326	XP_00604282	0	0	0	3.74	0.48	0	4.08	0.61	2.4	0	0	0
2-3.c10563/1/1916	Unknown (protein for IMAG	197246230	AAI68800.1	216.81	8.24	146.72	384.39	200.97	45.78	181.57	174.77	273.35	59.55	24.19	50.03
1-2.c41161/1/4386.	keratin, type I cytoskeletal 47	147899009	NP_00108243	3136.78	562.95	2082.86	2317.74	1657.67	536.14	2263.64	2585.54	2809.59	484.52	820.68	369.94
2-3.c21738/1/2493	PREDICTED: myosin-bindin	556961300	XP_00599099	2.76	10.91	4.7	3.34	2.18	259.5	2.2	6.09	5.48	0.04	0.32	0.72
1-2.c6102/2/1412	homogentisate 1,2-dioxygena	148230238	NP_0010875	6.18	3.5	1.82	3.5	0	3.2	5.59	4.67	8.48	0.44	0.09	0.53
1-2.c34161/1/1654	--	--	--	0	13.59	0	10.43	0	5.03	0	0	0	76.32	2.44	51.86
3-6.c18716/1/2695	myosin, heavy chain 4, skelet	47575800	NP_00100124	1.6	0.02	6.87	3.95	4.68	0	3.49	6.35	3.53	0.5	0.44	0.53
3-6.c10359/1/3816	hexokinase 1 [Xenopus laevis	160420247	NP_00109665	1.85	0	1.89	0.07	3.83	3.88	5.9	4.87	8.34	0	3.54	0
2-3.c53852/1/2381	PREDICTED: dual specificit	847088131	XP_01281959	0.85	1.53	0.77	0	1.01	2.92	3.58	1.1	1	0	0	0
1-2.c16775/1/1866	serine protease HTRA1 precu	350276150	NP_00108879	5.28	155.73	2.82	3.92	4.23	48.94	2.47	7.27	5.07	78.98	80.24	124.98
1-2.c15912/30/1420	--	--	--	2.55	5.13	5.69	3.68	8.89	28.43	5.67	4.7	11.51	0.59	1.15	0.15
2-3.c47911/1/2529.	regulator of cell cycle RGCC	156717456	NP_00109626	543.81	60.5	439.04	653.34	790.14	31.89	995.01	494.08	366.81	64.41	153.7	58.87
2-3.c52177/1/6339.	PREDICTED: myosin-4 [Xen	847156920	XP_01282446	5.19	0.78	43.02	42.75	18.43	0	48.12	17.97	26.35	6.75	0	2.01
2-3.c10462/1/2112.	--	--	--	338.55	8.58	84.73	103.72	242.32	4.23	215	305.34	211.99	48.19	20.94	68.91
1-2.c32802/1/4797.	PREDICTED: actin, alpha ca	874446319	XP_01295408	620.52	206.42	1524.5	1415.16	718.82	1424.04	2566.03	2510.56	1097.33	307.25	406.62	397.15
2-3.c11688/3/2609	--	--	--	16.48	0.15	10.61	25.73	15.42	1.83	11.54	10.53	13.82	3.21	1.4	1.57
2-3.c25244/2/2645	myosin, heavy chain 4, skelet	47575800	NP_00100124	456.07	21.74	1557.31	902.72	1226.32	11.89	877.27	1635.47	977.16	172.38	110.85	246.84
2-3.c13378/2/1998	PREDICTED: segment polari	847126719	XP_01281815	0	1.63	0	0	0	2.77	0	0	0	5.99	1.79	3.14
2-3.c28216/1/1972	PREDICTED: collagen alpha	701292594	XP_01002219	12.39	115.65	29.88	26.52	15.39	109.99	11.26	13.9	28.31	0.75	0.02	4.4
3-6.c2685/8/3619	PREDICTED: myosin-4 [Xen	847156920	XP_01282446	52.01	1.51	21.99	57.22	27.37	0.34	34.46	52.51	27	6.77	4	9.12
2-3.c34127/1/2521	PREDICTED: nebulin-like is	557018863	XP_0060098	14.96	3.93	14.01	5.8	1.57	2.8	21.59	13.04	11.48	0.62	5.08	2.98
1-2.c24401/1/1580	pre-mRNA cleavage complex	194018660	NP_00112341	4.76	14.69	3.84	2.26	1.72	5.99	0	0.6	0	7.6	14.06	12.52
2-3.c43869/1/2241.	--	--	--	2.32	12.73	9.29	1.66	3.26	17.02	1.13	2.76	3.53	0.06	0	0.09
1-2.c23600/1/1565	--	--	--	3.94	66.03	1.52	5.97	2.11	10.16	2.56	1.62	2.42	41.82	63.11	34.29
2-3.c10627/10/2082	lysyl-tRNA synthetase [Xeno	147898923	NP_00108005	30.61	22.69	11.6	6.1	6.9	7.27	28.34	14.81	23.14	6.26	3.02	3.09
1-2.c13992/6/1370	unnamed protein product [On	642126608	CDQ60726.1	23.36	5.34	36.61	31.16	21.3	4.77	18.36	26.8	25.15	0.74	1.11	8.93
1-2.c2352/9/1630	PREDICTED: cytochrome P4	602675207	XP_00744308	0	0.54	0.07	0	0	3.17	0.09	0	0.64	22.78	14.88	10.72
1-2.c5402/1/1526	alpha1 type II collagen [Cync	5360532	BAA82043.1	10.05	235.7	79.06	31.17	9.28	86.69	27.22	23.57	24.78	5.31	0.85	6.69
3-6.c7024/2/3169	alpha 1 type I collagen [Rana	3242649	BAA29028.1	2.93	84.8	10.83	9.21	7.04	72.86	10.97	7.07	16.93	2.76	0.91	2.45
1-2.c26203/2/1584	PREDICTED: nucleolar GTP	512849296	XP_00293522	17.84	30.54	0	24.04	0	1.93	26.46	17.75	17.03	4.05	6.26	1.17
1-2.c40189/1/1472	PREDICTED: collagen alpha	194224629	XP_00191509	7.6	12.11	23.29	8.58	3.54	6.64	12.53	9.25	16.99	3.13	1.07	1.21
2-3.c55586/1/2472	--	--	--	109.39	4.5	24.85	32.08	75.51	0.65	62.7	93.43	63.69	17.56	10.03	21.82
2-3.c9965/4/2240	PREDICTED: serine-rich coi	847147000	XP_01282228	14.41	0.85	5.44	7.39	11.56	0	12.78	6.23	5.87	1.48	0.26	1.56
1-2.c50878/2/1422	tyrosine 3-monooxygenase/tr	89515082	ABD75373.1	0	0	34.98	0	4.92	0	0	0	0	83.65	0	138.15
3-6.c7488/1/3425	myosin heavy chain, partial [	4249701	AAD13772.1	0.05	0.05	2.76	1.76	1.15	0	4.84	1.92	1.14	0.12	0	0
1-2.c41161/1/4386.	larval type I keratin [Rana cat	134140868	ABO61147.1	1716.14	452.29	990.88	788.12	474.86	41.92	866.49	767.21	566.21	161.09	151.52	203.16
3-6.c8993/1/2680	PREDICTED: nexilin isoforn	512837277	XP_00491397	35.01	75.35	40.04	22.34	35.26	175.21	19.94	27.74	37.57	2.09	11.03	6
2-3.c22138/1/2331.	--	--	--	36.27	10.18	49.03	44.73	37.7	6.08	57.5	29.5	46.23	14.9	3.48	5.77
1-2.c4233/4/1280	tetraspanin-2 [Xenopus (Silur	163915095	NP_00110653	31.54	134.42	74.83	27.74	71.71	340.13	33.74	46.29	43.19	11.78	6.09	6.97
3-6.c3497/3/2851	matrilin-2 precursor [Xenopu	45361321	NP_989238.1	11.56	12.65	4.69	4.74	2.45	14.99	4.22	8.21	6.09	0	0.47	2.02
2-3.c59107/1/2602.	--	--	--	8.08	9.75	7.21	2.77	3.11	7.9	28.09	4.45	15.44	0.37	0.22	0
2-3.c33887/1/1925	PREDICTED: ATP-binding	847161447	XP_01282525	0.15	1.03	0	0.48	0	0	0	0	0	4.5	2.12	4.43
2-3.c36298/1/2238.	PREDICTED: ankyrin repeat	512839176	XP_00293509	9.21	0.06	4.53	4.08	3.98	0.75	9.15	7.94	20.36	0	3.45	0.5
3-6.c17414/1/2742	PREDICTED: myosin-4 [Xen	847156920	XP_01282446	0	0	2.31	0.96	0.67	0	1.83	0.89	0.78	0	0	0
3-6.c16042/1/3035.	PREDICTED: myosin-4 [Xen	847156920	XP_01282446	1.33	0	3.16	2.74	2.91	0	4.67	2.26	1.85	0.23	0	0
2-3.c20452/1/2029.	PREDICTED: triosephosphat	558167186	XP_0061242	3009.12	438	3851.57	3503.49	3060.06	684.46	3752.26	3100.59	4640.99	1130.73	890.18	729.04
2-3.c46431/1/2168	PREDICTED: serine--tRNA	301622150	XP_0029404	0	2.44	0.59	2.96	0	0	2.27	1.03	1.25	0	0	0
2-3.c50941/2/2381.	--	--	--	0.59	0	2.61	0.85	3.14	14.59	1.28	2.23	2.21	0	0	0
1-2.c52410/1/1354	collagen, type 1, alpha 2 prec	147898763	NP_00108072	192.9	2278.91	535	338.81	303.56	3225.02	306.77	249.59	457.38	109.55	43.36	81.54
3-6.c18258/1/2976	PREDICTED: caveolin-2-like	301611463	XP_00293525	6.17	6.37	0.12	3.69	0	4.1	1.23	3.57	2.58	0.25	0.25	0.01
2-3.c54531/1/1984	--	--	--	11.92	3.59	53.86	22.33	5.69	0.15	12.06	33.37	24.56	2.38	0.26	2.8
3-6.c16873/1/3147	myosin, heavy chain 4, skelet	47575800	NP_00100124	0.58	0	2.7	2.17	1.47	0.09	2.61	1.5	1.98	0	0.15	0.18
2-3.c5210/3/2110	--	--	--	8.28	62.05	20.28	17.03	20.85	94.22	19.52	16.72	20.58	3.78	0.26	6.19
2-3.c59218/1/2521	Brg1 [Xenopus laevis]	56474875	AAV91782.1	1.97	15.92	1.99	1.96	0	0.31	0	0.08	0.73	7.99	6.88	11.39
1-2.c36994/4/1771	Basic leucine zipper and W2	677309152	KFP91580.1	44.1	1.38	26.28	10.55	22.31	4.84	11.97	17.4	11.58	0	5.41	2.06

1-2.c30442/1/1583.	--	--	--	37.25	6.42	22.24	21.57	24.9	2.35	56.65	28.16	26.69	7.14	9.71	6.03
2-3.c30410/1/6158.:	PREDICTED: myosin-4 [Xer	847156920	XP_0128244(	0	0.5	7.45	5.21	1.19	0.07	5.96	0.97	4.24	0.16	0	0.14
1-2.c28349/1/1495	TRH4 protein [Xenopus laevi	148226933	NP_0010839(	2.64	0	0	5.98	0	0	0.87	0.55	2.64	29.45	43.33	20.19
1-2.c40465/1/3661.	actin, alpha skeletal muscle B	820145793	KKX13565.1	4.88	17.99	2.37	9.16	14.93	50.36	7.08	4.29	19.43	171.9	89.57	219.2
1-2.c11974/3/1520	PREDICTED: ornithine amin	768381361	XP_01158831	25.41	10.87	16.3	18.08	3.52	9.42	28.36	18	22.19	7.09	5.6	0.79
2-3.c27287/1/2364	hypothetical protein CAPTEI	443682880	ELT87315.1	1.75	47.73	2.77	2.82	1.69	6.99	3.29	0	4.56	44.78	62.99	28.38
1-2.c7105/4/1683	transketolase-like protein 1 [X	45361299	NP_989227.1	0	0	0	10.34	0	18.16	7.26	6.61	9.19	0	3.26	0
2-3.c55918/1/2505	myosin, heavy chain 2, skelet	55742222	NP_0010068(	1.52	0.16	3.39	1.11	2.63	0	1.82	3.55	1.44	0	0	0.24
2-3.c45694/1/2236	unnamed protein product [On	642130802	CDQ57637.1	1.52	12.15	0.82	1.73	1.18	4.63	0.29	1.41	1.17	20.79	21.22	23.08
3-6.c16047/1/3033	PREDICTED: collagen alpha	847166756	XP_01282667	8.63	73.36	18.34	8.57	12.6	53.53	5.24	10.54	7.17	1.79	0.75	1.57
1-2.c16133/380/150	mitochondrial inorganic phos	22653426	AAN04052.1	164.23	113.79	120.43	221.81	148.83	139.69	135.59	226.17	282.96	46.65	71.98	45.45
1-2.c40861/2/1438	alpha 1 type I collagen [Rana	3242649	BAA29028.1	5.82	147.08	18.13	10.22	10.59	117.08	12.51	8.98	21.74	2.87	0.71	2.77
1-2.c28747/1/1357.	--	--	--	3.36	29.7	3.15	4.38	1.76	8.83	2.06	2.27	4.83	93.94	70.54	48.86
1-2.c35444/1/1643	--	--	--	11.42	5.05	11.14	8.36	7.45	5.16	17.2	8.61	10.12	1.44	3.31	1.32
1-2.c24923/1/1601	probable threonine--tRNA lig	156717366	NP_00109622	19.34	1.34	19.72	12.58	16.18	89.95	25.67	9.05	9.46	0.13	0	2.27
2-3.c24676/1/2629	alpha 1 type I collagen [Rana	3242649	BAA29028.1	210.91	5562.29	726.64	447.07	519.22	4587.38	413.12	284.85	604.26	154.48	61.12	103.29
2-3.c56150/1/2330.	ORF2 [Acanthochelys spixii]	6576738	BAA88337.1	2.47	21.7	2.97	2.77	2.49	4.04	0.8	0.22	0	21.03	26.15	11.33
3-6.c8248/1/2910	methionine--tRNA ligase, cyt	283945504	NP_0010170(	3.44	26.42	4.67	2.99	5.07	55.26	13.72	6.4	4.11	0.71	0	0.95
3-6.c7840/2/3476	PREDICTED: nebulin isofori	847166510	XP_01282661	2.57	0.12	5.15	6.12	0	0	4.23	12.05	8.3	0	0	1.51
2-3.c10758/1/2030	--	--	--	0	0	0.8	1.12	0	0	1.67	1.5	1.12	0	0	0
2-3.c38690/2/2654	collagen, type 1, alpha 2 prec	147898763	NP_00108072	28.64	670.87	157.95	120.19	93.47	1038.32	97.85	42.27	99.88	21.34	7.44	26.03
3-6.c14577/2/3047	eukaryotic translation initiatio	148232020	NP_00108075	1.9	0	19.43	1.31	0	0.54	0	0	0	18.2	18.5	0
1-2.c51845/2/1449	--	--	--	7.21	5.75	17.37	10.26	1.16	3.61	11.17	7.3	2.27	0	0	0.74
1-2.c11185/2/1325	--	--	--	268.79	8.49	145.51	426.23	250.39	147.1	307.75	326.81	335.33	133.53	43.72	66.5
3-6.c17958/3/2910	PREDICTED: myosin-4-like	301611451	XP_00293525	0.62	0.05	3.05	1.96	1.73	0	2.52	3.04	1.56	0.32	0.03	0.17
1-2.c43801/1/1761	PREDICTED: fructose-bisph	530658308	XP_00531468	158.74	4.55	125.18	158.21	174.43	7.79	124.64	124.78	193.81	33.38	28.89	44.98
1-2.c18977/1/1550	--	--	--	17.57	0.63	9.6	15.5	17.23	4.1	20.93	25.85	28.19	0	13.47	3.34
1-2.c5405/1/1572	PREDICTED: paired amphip	847088488	XP_01282055	2.02	0.33	0.23	5.33	0.8	1.26	0	0	0	5.31	4.53	3.92
1-2.c6549/3/1869	myosin, heavy chain 4, skelet	47575800	NP_00100124	921.16	45.88	3850.3	2374.4	3210.27	5.79	1508.02	1748.93	924.53	318.24	212.94	523.7
2-3.c47157/1/2706.	--	--	--	0	123.83	2.19	10.17	0	7.93	0	0	9.73	88.42	111.45	50.87
3-6.c18644/4/3577	PREDICTED: myosin-4 [Xer	847156920	XP_0128244(	0.52	0	7.68	1.83	2.47	0.12	2.66	1.02	3.14	0.24	0.22	0.13
1-2.c24322/1/1583	PREDICTED: paraplegin [X	301608280	XP_0029337(	0	1.03	0	1.64	0.81	0	0.38	0	0	7.8	11.21	5.08
1-2.c38496/1/1234	synaptophysin-like protein 2	54606873	NP_00100611	65.33	16.16	58.06	85.53	60.7	208.84	126.37	89.43	115.64	31.42	22.36	25.22
2-3.c55609/1/2067	PREDICTED: nebulin isofori	847166510	XP_01282661	60.41	24.85	165.27	125.25	55.71	1.82	92.14	114.47	89.39	16.97	6.73	45.45
2-3.c21582/1/2184	Keratin, type II cytoskeletal c	677541627	KFR03099.1	918.22	25.07	788.42	580.59	614.37	76.89	607.28	628.02	572	179.3	64.31	207.63
1-2.c17608/1/1986	PREDICTED: actin, alpha ca	803091489	XP_00401102	7.2	4.09	35.01	13.1	6.4	20.93	52.04	51.25	29.49	15.24	9.16	6.69
1-2.c52214/1/1542	RecName: Full=Voltage-depe	46576355	O57483.1	19.22	18.22	89.39	35.74	44.9	45.13	36	45.51	71.46	8.44	7.63	17.97
1-2.c28775/1/1440	PREDICTED: sarcalumenin i	847166841	XP_0128267(	89.99	7.62	116.33	176.26	90.3	27.8	28.12	58.46	68.96	0	0	24.82
1-2.c39217/1/1601	PREDICTED: ADP/ATP trar	829867054	XP_0126403	16.03	2.44	17.92	20.44	21.27	10.49	29.2	18.85	24.44	6.23	9.52	0.64
1-2.c29435/1/1242	--	--	--	20.6	694.22	67.72	25.77	46.56	412.74	55.21	19.73	46.62	16.58	2.83	5.7
3-6.c11334/1/2761	PREDICTED: obscurin isofo	847141066	XP_0128211	20.97	14.44	24.53	16.64	16.68	26.12	44.21	40.2	71.42	22.98	8.31	7.66
1-2.c24693/1/702	myosin essential light chain f	52699568	AAU86906.1	11202.45	3553.47	20626.33	15488.03	19497.57	18092.79	21451.4	20247.19	18746.27	6268.58	5089.03	4527.12
2-3.c43665/1/2600	dehydrogenase/reductase SDI	166158027	NP_00110742	9.33	10.55	10.22	7.26	9.55	15.96	21.54	9.48	16.68	1.4	1.94	5.92
3-6.c11995/2/3177	PREDICTED: ectonucleotide	847135215	XP_01282011	0.23	4.77	0.76	0	0.76	1	0	0	0.32	6.3	1.7	5.76
2-3.c11756/5/2320	PREDICTED: schwannomin	847126593	XP_0128181(	0.76	0.17	2.04	0.97	1.03	0	0.26	1.75	1.72	0	0	0
2-3.c9294/1/2336	PREDICTED: eukaryotic trai	847089031	XP_0128217	0.26	0	0	1.25	0	0	0	0	0	4.99	2.24	1.24
3-6.c3069/5/2948	PREDICTED: myosin-4 [Xer	847156920	XP_0128244(	1.63	0	3.16	1.56	2.68	0	2.04	3.47	1.35	0	0.08	0.31
2-3.c61064/1/2551	--	--	--	21.14	0.53	5.05	4.01	11.6	0.99	9.62	6.87	12.4	0	4.13	1.57
2-3.c34950/2/2605	dehydrogenase/reductase SDI	166158027	NP_00110742	59.58	23.29	45.96	48.9	43.12	42.17	87.36	42.96	82.48	10.8	12.84	26.69
3-6.c14479/1/2867	--	--	--	1.93	23.04	16.74	4.3	4.89	105.17	1.02	2.09	6.07	0	0	0
3-6.c18237/1/2784	PREDICTED: protein FAM1	512816747	XP_0049112	17.67	0	14.31	6.2	12.9	2.66	8.07	5.99	13.67	0	2.91	2.37
1-2.c45531/1/1634.	--	--	--	0	2.49	2.04	1.82	1.95	0	0	0	0	16.7	23.11	3.56
1-2.c18325/5/1432	PREDICTED: WNT1-inducil	512845805	XP_0029395(	2.66	23.3	4.18	3.36	4.77	21.58	2.97	3.54	7.65	75.21	87.54	71.39
3-6.c13175/2/3112	kinectin [Xenopus (Silurana)	194018594	NP_00112338	9.31	0	8.33	0.02	10.63	4.56	10.72	9.47	9.47	2.43	4.39	0
1-2.c23459/2/1495	hedgehog acyltransferase-like	147901798	NP_00107949	102.02	36.59	280.04	135.68	124.14	104.84	204.28	264.03	266.88	98.83	55.64	42.37
2-3.c49774/1/2087	PREDICTED: guanine deami	512813022	XP_00491082	0.62	0	0.23	0.1	0.44	0.93	0.09	0	0.1	5.85	1.65	7.54
1-2.c12973/2/1297	PREDICTED: AMP deamina	697003755	XP_0095680(	49.33	20.2	17.32	9.1	13.09	26.87	49.1	23.65	60.81	5.45	18.39	7.17
1-2.c24854/1/1866	--	--	--	49.8	29.78	74.32	46.93	47.24	4.96	84.21	26.36	49.52	13.68	2.68	9.52

3-6.c3321/2/2731	uncharacterized protein LOC	148232016	NP_00109117	33.6	0.75	0.5	7.15	13.71	0.22	15.76	17.35	13.08	1.6	8.42	1.42
2-3.c9855/11/2246	disks large-associated protein	213982915	NP_00113562	1.42	1.69	1.35	0	0.44	4.37	1.38	1.57	1.05	0	0	0
2-3.c43334/1/8595.	PREDICTED: AMP deamina	847091857	XP_01282629	22.2	0	26.99	5.87	15.4	7.64	17.92	10.38	17.44	0	2.18	1.22
2-3.c46968/1/2099.	--	--	--	16.18	3.15	6.34	22.1	14.19	2.79	9.26	11.16	12.08	207.86	195.98	125.41
2-3.c19815/4/2170	PREDICTED: nebulin isofor	847166512	XP_01282661	83.01	40.89	284.93	64.06	0	0	143.98	90.11	216.42	32.14	31.55	48.99
2-3.c52988/1/2462	--	--	--	0	53.59	0.33	0.32	0.12	14.16	0.52	0.09	0.14	28.13	5.28	31.02
2-3.c12461/1/2048	--	--	--	39.05	190.15	29.76	33.66	20.84	100.15	32.99	26.47	47.13	524.68	444.66	521.28
2-3.c22138/1/2331.	--	--	--	3.22	2.07	7.22	4.64	7.08	0.97	7.15	3.48	8.19	0.56	0	0.58
2-3.c8411/1/2519	PREDICTED: filamin-A-inte	512841708	XP_00491457	5.44	4.65	3.43	1.87	1.45	9.14	0.84	2.38	3.1	0.22	0	0.1
1-2.c43099/1/1631	PREDICTED: titin-like [Che	591350793	XP_00706984	1.11	0	0.8	0.04	0.94	0.11	2.42	1.93	1.2	0	0	0
1-2.c16800/2/1415	alpha 1 type I collagen [Rana	3242649	BAA29028.1	0	0	0	0	0	129.08	0	0	0	41.94	12.14	9.46
2-3.c22261/2/2050	alpha 1 type I collagen [Rana	3242649	BAA29028.1	37.44	1006.54	112.28	84.82	59.48	735.14	85.22	56.81	128.94	32.49	19.03	17.61
1-2.c29226/1/9331.	PREDICTED: 14-3-3 protein	831273285	XP_01267899	80.42	14.95	1.55	36.2	1.36	0	34.18	40.01	40.09	0	17.37	0
1-2.c39671/1/1383	--	--	--	5.68	21.88	5.46	4.61	5.72	14.45	6.15	4.62	6.54	78.73	74.68	117.22
2-3.c11566/9/2336	dehydrogenase/reductase SDI	166158027	NP_00110742	70.48	26.65	58.69	60.94	50.31	45.78	92.61	49.53	105.85	13.66	13.77	32.59
3-6.c10238/4/3210	PREDICTED: F-box-like/WI	705676379	XP_01011698	3.55	4.73	1.44	0	0.64	0.96	1.14	0.84	0.68	0	0	0
3-6.c8757/1/3647	PREDICTED: protein CDV3	847134819	XP_01281997	0	11.59	1.45	3.89	0	3.2	2.55	1.09	1.78	0	0.47	0
1-2.c11682/1/1235	Receptor-transporting protein	304434768	NP_00113458	22.76	6.94	21.04	29.3	15.95	4.69	29.92	15.32	34.01	4.51	7.42	4.24
1-2.c27511/1/1632	--	--	--	101.41	8.8	24.79	40.83	41.25	13.13	51.51	36.01	37.56	9.94	17.09	4.74
1-2.c34954/1/6762.	PREDICTED: fructose-bisph	731506121	XP_0105968(	1630.23	14.48	705.15	482.24	1275.25	147.58	1334.5	1110.94	1845.5	417	447.75	283.01
1-2.c41169/1/1446	--	--	--	13.45	65.3	8.24	11.54	7.85	18.21	9.23	9.21	12.24	161.97	165.96	130.69
2-3.c7363/15/1872	tropomodulin 4 (muscle) [Xe	148233551	NP_00108736	50.19	23.35	84.05	60.52	56.1	67.15	55.22	73.07	83.82	18.99	16.84	19.08
3-6.c18549/1/2690	myosin heavy chain, partial [	4249697	AAD13770.1	0.72	0	3.36	2.5	2.02	0.09	3.65	2.91	2.29	0.68	0.16	0.12
1-2.c17304/1/1684	PREDICTED: collagen alpha	847130150	XP_01281895	4.89	66.37	28.61	14.27	1.16	3.6	14.59	3.88	14.51	2.86	0.31	0.82
1-2.c46371/1/1797	PREDICTED: tropomyosin a	847143254	XP_01282187	7.1	0	6.11	7.53	2.35	0	5.06	3.54	18.61	0	0	0
3-6.c3051/7/2950	kinectin [Xenopus (Silurana)	194018594	NP_00112338	43.95	10.71	21.35	15.49	31.76	19.16	31.35	33.18	32.94	12.28	8.39	4.9
1-2.c17885/2/1573	periostin precursor [Xenopus	163915007	NP_00110637	18.08	351.55	35.08	0.65	13.11	116.16	28.3	16.52	23.94	5.84	1.07	6.81
1-2.c22457/1/1698	myosin, heavy chain 4, skelet	47575800	NP_00100124	4.31	0	7.18	4.63	7	0	6.19	8.76	4.87	0.36	0.34	1.66
1-2.c51822/7/1689	PREDICTED: myosin-4 [Xe	847156920	XP_0128244(	137.31	23.99	2420.63	1479.71	772.02	19.61	3905.99	922.63	1288.43	46.88	36.82	26.93
2-3.c60498/1/2566.	--	--	--	10.63	1.1	27.11	23.9	8.24	2.07	13.29	30.77	23.37	7.86	2.28	1.99
2-3.c30807/1/2242	--	--	--	1.65	5.74	2.4	0.49	3.25	0	0.77	1.98	4.94	0	0	0
3-6.c19112/1/3714	myosin heavy chain, partial [	4249701	AAD13772.1	4.62	0.14	8.16	5.63	6.17	0.04	6.01	11.3	7.51	2.32	2.16	1.25
3-6.c17411/1/3023	PREDICTED: obscurin isofo	847141062	XP_01282119	23.42	13.65	31.36	36.46	30.14	38.68	59.08	53.35	82.14	17.41	17.82	16.56
1-2.c37266/1/1382	PREDICTED: nebulin isofor	847166510	XP_01282661	234.09	42.48	387.26	330.33	190.89	178.84	270.66	398	313.1	118.56	46.5	99.83
1-2.c44007/1/1492.	--	--	--	0.47	0	1.85	1.83	1.81	0	3.95	4.37	6.3	0	0	0
2-3.c9333/5/2420	abhydrolase domain-containi	349501031	NP_00100677	0.82	2	0	0	0.95	0.44	0	0	0	2.07	2.96	2.41
2-3.c23039/1/2333	--	--	--	14.08	1.92	15.31	4.95	14.61	0.45	31.44	35.9	50.29	0	9.38	18.78
2-3.c56611/1/2203.	PREDICTED: matrix Gla pro	301612298	XP_00293565	21.46	54.88	224.99	0	149.91	7.08	122.66	23.89	125.08	0	5.39	9.56
3-6.c14807/1/3174	phosphofructokinase, muscle	148224874	NP_00108692	26.85	0	2.34	28.96	15.46	1.63	11.82	18.57	16.29	8.22	0.51	2.4
1-2.c30144/1/1287	--	--	--	7.13	10.61	5.81	2.62	10.33	11.93	3.85	3.3	1.79	72.55	46.95	39.97
1-2.c19898/1/1180	endophilin-A2 [Xenopus (Sil	45360901	NP_989126.1	0	0	0	2.32	0	0	0	0	0.32	13.33	6.01	7.62
1-2.c18541/1/1911.	RecName: Full=Gelsolin; Alt	121119	P14885.1	0	64.91	42.65	20.01	0	0	0	35.46	41.85	0	0	0
1-2.c18852/3/1040	PREDICTED: tropomyosin a	831547267	XP_01272688	26065.16	1187.26	40659.96	30607.13	34230.61	855.77	31324.35	37199.42	25119.95	8768.28	9305.26	8479.72
1-2.c48528/1/1622	--	--	--	0	29.53	0	0.4	0	1.58	0	0	0	15.86	10.8	1.25
3-6.c13597/1/3044	mitochondrial 2-oxoglutarate	71895777	NP_00102568	1.68	0	4.52	2.47	2.9	1.87	6.11	4.2	8.42	0	0.49	2.56
2-3.c33216/1/4581.	--	--	--	454.24	36	270.47	869.35	1013.26	509.78	2442.02	1262.42	2788.25	684.78	656.86	390.45
2-3.c56842/1/2665.	--	--	--	0.43	6.13	0.44	2.31	2.52	8.76	2.93	1.86	1.92	35.08	31.21	64.58
1-2.c38894/1/1686.	--	--	--	31.17	20.01	18.09	71.53	23.25	1.54	6.41	31.47	24.57	0	0.93	0.46
2-3.c38261/113/238	PREDICTED: ras GTPase-ac	558228880	XP_00613832	114.3	130.79	75.38	50	61.25	184.57	54.22	55.07	25.68	14.6	6.55	13.5
2-3.c19426/1/2158	phosphoglucose isomerase [D	20067633	CAC83783.1	594.49	92	548.43	749.4	745.04	179.2	481.16	616.03	705.45	176.09	100.3	212.43
1-2.c14466/1/1247	--	--	--	3.22	3.19	1.52	0.46	3.25	4.76	0	1.42	2.26	26.54	42.21	17.73
1-2.c28632/1/1999	Myozenin-1-like [Xenopus la	148237556	NP_00107945	85.23	4.12	98.2	90.9	93.61	83.16	99.82	84.98	103.46	20.63	21.69	31.94
1-2.c40683/1/1740	cebpd, CCAAT/enhancer bin	89273345	CAJ81462.1	0	0	0	3.07	0	0.93	24.72	12.84	0	0	0	0
2-3.c25619/1/1937	--	--	--	3.63	11.72	9.41	8.26	6.36	33.68	12.42	3.79	8.15	0.79	0.51	2.18
3-6.c11376/2/3083	PREDICTED: long-chain fatt	512839272	XP_00491424	0	0.6	0.11	0.2	1.72	1.3	0	0.28	0	4.26	2.92	6.82
1-2.c50641/1/1491	PREDICTED: actin, alpha ca	803091489	XP_00401102	199.98	143.05	424.36	206.93	206.18	192.76	192.47	260.37	268.43	83.06	45.48	68.44
2-3.c24429/1/2023	autocrine motility factor recep	147901912	NP_00108315	0	0	2.86	0	2.98	0	1.71	0.93	1.67	0	0	0
2-3.c3894/5/2641	solute carrier family 38, mem	58332320	NP_00101105	59.89	38.36	58.23	34.19	43.65	40.86	36.59	45.74	49.86	12.06	7.51	14.45

2-3.c62846/1/2652	phosphofructokinase, muscle	148224874	NP_00108692	20.56	0.9	8.73	33.15	20.67	3.92	17.52	19.28	20.19	7.07	0	5.92
1-2.c52348/2/1575	alpha 1 type I collagen [Rana	3242649	BAA29028.1	9.48	295.55	38.78	38.22	23.73	270.47	38.41	34.95	80.76	13.6	9.09	9.04
2-3.c56664/1/2240	Transposable element Tcb1 tı	317419272	CBN81309.1	20.78	5.52	19.41	11.23	24.09	9.8	17.76	32.52	20.33	7.78	2.8	6.43
2-3.c16384/1/2164.	PREDICTED: collagen alpha	512841609	XP_00293490	1.19	0	3.57	1.66	0.04	0	2.6	2.86	2.81	0	0	0.31
1-2.c41069/1/4687.	PREDICTED: tenomodulin [	847171214	XP_01280901	19.31	134.35	32.86	7.39	5.68	78.73	5.98	10.58	10.46	0.54	0.97	0
2-3.c54780/1/2294	--	--	--	1.76	1.21	2.31	2.19	1.89	1.87	2.86	2.14	5.56	0.2	0.38	0.42
2-3.c35881/1/2098	--	--	--	9.85	3.63	13.69	14.04	19.43	3.48	14.84	18.49	23.65	2.93	5.34	5.08
2-3.c13811/2/2143	PREDICTED: RNA-binding	512850834	XP_00294294	10.37	3.72	10.71	7.67	8.04	4.34	12.46	6.81	8.63	1.94	1.87	0.96
2-3.c27449/1/2134	pecanex-like protein 4 [Xeno	148228914	NP_00108266	0.53	0	2.25	1.05	0.51	0.83	0	0	0.12	2.66	5.93	3.32
1-2.c51966/42/1541	PREDICTED: myosin-4 [Xe	847156920	XP_0128244(	8104.8	176.65	14502.74	11822.71	11262.28	55.03	6946.93	14122.91	11306.84	3808.04	2510.81	3153.79
1-2.c26750/6/1428	eukaryotic translation elongat	148231139	NP_00108844	6.83	27.34	12.95	10.89	6.54	34.71	8.32	7.79	12.32	0	0	3.55
1-2.c26283/2/1257	transgelin [Xenopus (Silurana	71896187	NP_00102557	21.62	23.84	10.9	19.79	27.67	16.35	19.4	10.09	21.01	269.42	145.85	257.75
3-6.c18762/4/3676	myosin heavy chain, partial [	4249701	AAD13772.1	1.42	0.1	5.04	3.75	3.2	0	3.87	3.35	3.12	0.76	0.34	0.52
2-3.c10570/1/2121	--	--	--	2.19	26.88	1.42	2.62	1.02	5.18	2.2	1.65	2.71	28.35	39.44	37.97
1-2.c29283/1/1662	calsequestrin 1 (fast-twitch, s	148231736	NP_00108022	196.44	26.38	339.23	249.64	337.05	279.46	303.78	334.4	328.1	94.8	62.98	111.99
3-6.c18307/1/3608	PREDICTED: myosin-4 [Ail	301771630	XP_00292123	1.86	0.12	3.67	2.92	3.75	0	1.81	3.02	3.53	0.5	0.42	0.28
2-3.c2689/6/2296	PREDICTED: phosphogluco	847117435	XP_01281625	15.54	17.28	23.7	7.47	10.23	93.67	8.29	13.07	16.58	3.01	3.12	2.5
1-2.c1815/52/1651	PREDICTED: F-actin-cappin	525020623	XP_00505782	195.57	65.3	224.59	190.02	191.68	180.45	156.24	243.18	160.08	51.75	3.58	93.24
1-2.c12059/1/1401.	--	--	--	18.35	28.99	24.36	5.04	16.75	10.18	6.01	5.11	4.77	75.55	54	129.27
2-3.c18648/2/2420.	--	--	--	80.95	12.64	57.16	73.35	65.51	53.47	51.78	74.63	94.78	19.52	15.75	20.81
2-3.c60691/1/2168.	Putative uncharacterized trans	449282618	EMC89440.1	0.81	115.53	0	13.75	2.33	37.6	8.1	8.15	0.73	115.8	160.47	85.51
2-3.c20637/1/1909	--	--	--	0.35	0	0.29	0	0.52	0	0	0	0	8.5	5.53	31.75
1-2.c43170/1/1459	--	--	--	2.53	3.63	0	0	1.84	2.75	0.24	0.84	1.19	19.86	16.72	19.04
1-2.c16454/4/1563	glioma tumor suppressor canc	148228700	NP_00109002	0	0	0	0	0	13.02	0	0	0	26.2	33.63	0
1-2.c4799/1/1485.1	PREDICTED: matrix Gla pro	301612298	XP_00293565	34.25	18.52	70.75	24.03	48.65	6.2	87.32	24.31	63.44	11.17	14.56	8.35
2-3.c5036/3/2673	autocrine motility factor recep	147901912	NP_00108315	3.37	1.69	0	1.44	0	0	0	0	0	1.04	3.27	1.92
2-3.c16237/5/1967	voltage dependent calcium ch	11414934	BAB18559.1	20.8	43.43	17.6	11.45	10.75	91.54	5.06	13.32	23	1	0	1.03
2-3.c27016/3/2200	PREDICTED: mothers agains	558224886	XP_00613681	0	2.57	0.9	2.35	0	0	1.47	0.84	1.36	0	0	0
2-3.c17616/1/2253	PREDICTED: polyadenylate-	847099772	XP_01281235	38.45	15.37	19.58	13.35	8.59	9.91	10.04	20.91	13.04	1.12	2.87	4.09
1-2.c28311/1/1870	collagen, type II, alpha 1, par	374533628	AEZ53727.1	0	0	0.01	0	0	6.44	0	6.93	7.02	0	0	0
1-2.c46162/1/1377.	PREDICTED: protein FAM2	512809591	XP_00294112	0	1.12	0.64	0.76	1.5	0	0.83	0	0	12.99	18.63	6.02
2-3.c9923/1/2342	interferon-related developmer	148227722	NP_00109024	0	0	2.02	0	0	6.04	11.3	4.65	0.62	0	0	0
3-6.c10236/1/2796	alpha1 type II collagen [Cync	5360532	BAA82043.1	4.62	106.08	14.08	3.44	2.46	23.11	3.52	8.1	5.12	0.7	0.2	1.36
1-2.c45202/1/5025.	actin, alpha skeletal muscle B	820145793	KKX13565.1	8109.88	2538.92	15521.19	10206.97	12636.96	12546.45	11456.8	16400.85	11023.89	5663.65	3384.59	2384.91
2-3.c62222/3/2164	RecName: Full=Voltage-depe	46576355	O57483.1	29.49	12.96	14.48	1.04	29.45	36.29	20.45	27.95	39.24	8.96	0.63	11.85
1-2.c45071/1/1432	PREDICTED: collagen alpha	663272682	XP_00849548	0	0	0	0	3.04	3.07	0	0	0	3.6	3.16	5.86
2-3.c34375/1/4375	transforming growth factor-be	118403632	NP_00107232	56.69	20.15	66.62	58.74	41.39	29.23	55.03	68.54	55.64	20.6	9.41	19.25
2-3.c4015/4/1943	cysteine-rich secretory protei	73853836	NP_00102749	0	3.18	0.47	10.03	13.34	2.1	21.04	10.75	15.47	0.39	11.42	0.42
3-6.c9821/1/2700.1	--	--	--	0	6.16	0.8	0	0.08	6.39	0	0	0	2.4	1.92	4.49
1-2.c3263/1/1649	nuclear receptor subfamily 4,	147903779	NP_00108015	65.79	249.91	26.51	37.97	82.5	19.33	33.53	53.07	76.05	23.25	17.87	5.63
3-6.c5415/1/3098	PREDICTED: glycogen phos	512834332	XP_00293541	2.91	0	1.93	1.63	0.64	0.5	1.49	1.69	1.96	0	0.43	0
1-2.c14999/1/1522	larval type I keratin [Rana cat	134140868	ABO61147.1	2.48	0.67	2.34	0.95	2.12	0.31	2.08	2.17	1.26	0	0	0
1-2.c29748/1/1749	--	--	--	17.65	6.78	13.28	12.12	10.04	6.51	25.56	13.17	16.1	4.32	5.99	2.62
3-6.c19130/1/3587	PREDICTED: myosin heavy	831281475	XP_01267182	1.14	0.14	4.33	3.67	3.35	0	3.14	3.6	3.39	0.34	0.54	0.75
2-3.c26151/2/2484	PREDICTED: E3 ubiquitin-p	512849807	XP_00293652	10.82	16.53	17.81	8.22	8.36	6.27	10.76	10.1	20.92	2.61	0	6.3
3-6.c4959/2/3186	ubiquitin-conjugating enzyme	1381181	AAB02656.1	0.01	0	1.15	0.41	0	0	2.65	5.6	0.77	0	0	0
3-6.c10070/1/3331	alanyl-tRNA synthetase [Xen	189217804	NP_00112134	17.72	60.85	22.2	15.02	16.76	74.38	22.68	19.3	14.04	3.69	4.34	6.66
1-2.c2623/12/1821	IMP (inosine 5'-monophosph	148222892	NP_00108241	282.34	104.02	173.91	121.71	136.03	84.82	99.27	140.99	184.4	33.75	65.65	30.83
1-2.c22377/1/1530	popeye domain containing 2 [	148222019	NP_00108796	6.81	1.56	3.69	0	2.34	5.58	2.08	2.64	7.64	0	0	0.19
1-2.c20819/1/4155.	PREDICTED: actin, alpha sk	795473616	XP_01189147	0	0	0	0	0	50.18	29.08	2.89	34.52	0	1.26	0
3-6.c7302/1/2638	PREDICTED: glypican-5-lik	512866823	XP_00491786	75.7	6.62	46.08	66.97	55.3	9	52.98	42.35	84.47	10.68	19.61	20.41
1-2.c11857/1/6131.	PREDICTED: myosin bindin	847128061	XP_01281857	38.65	43.3	136.97	87.93	0	5.83	159.41	85.96	89.48	31.98	22.6	20.77
1-2.c16104/49/1495	PDZ and LIM domain 7 (enig	148223185	NP_00108073	25.6	76.88	42.32	66.67	31.9	96.32	99.64	98.31	42.86	35.09	24.22	9.85
3-6.c4130/2/4538	collagen, type 1, alpha 2 prec	147898763	NP_00108072	18.75	0	21.82	9.43	56.78	384.05	18.43	15.05	55.69	0.09	0	1.69
1-2.c3251/2/782	myosin light chain, phosphor	148225444	NP_00108400	54118.92	10066.39	85775.33	65433.59	79178.71	65694.52	70435.63	88657.82	66214.14	27276.37	19717.48	21593.25
1-2.c16998/1/1254	PREDICTED: phospholipid-t	301614897	XP_00293692	10.47	0.55	11.84	9.82	9.05	0.31	5.59	11.67	8.79	1.56	0.36	1.3
1-2.c2512/9/1569	LOC100145085 protein [Xen	165971391	AAI58516.1	12.05	7.3	10.87	19.01	14.57	3.8	13.5	8.11	15.1	0.7	2.5	3.63
2-3.c62312/2/2410	PREDICTED: myosin-4 isofo	632943616	XP_00788704	1.9	0	4.13	2	3.55	0	3.04	3.8	3.11	0.43	0.37	0.38

1-2.c25999/1/1500	diacylglycerol O-acyltransfer	147901247	NP_0010832	0.11	0.2	0	0.14	0.18	0.05	0	0	0.05	4.5	1.54	7.94
1-2.c21512/33/1379	calsequestrin 1 (fast-twitch, s	148231736	NP_00108022	1982.77	297.69	2724.14	2315.88	3049.35	2869.18	2746.11	3165.15	2794.53	1001.12	658.31	966.07
1-2.c52264/8/1347	PREDICTED: myosin-4 [Xe	847156920	XP_0128244	137.61	26.52	2612.32	2146.33	1051.88	14.96	5462.36	1251.06	1522.71	70.28	49.13	0
3-6.c8036/1/2732	--	--	--	30.16	2.04	10.06	25.84	15.46	2.81	18.29	16.57	19.9	3.35	2.73	7.55
1-2.c46410/1/1648	PREDICTED: sialic acid syn	301612838	XP_00293591	1.89	0	0	3.67	2.37	2.2	0	0	0.36	7.84	8.29	3.88
1-2.c44248/1/1429	PREDICTED: cytosolic non-	591359345	XP_00705465	27.68	0	3.66	40.22	6.28	0	0	20.33	11.23	204.2	110.82	135.55
3-6.c15514/1/3134	PREDICTED: angio-associat	602656532	XP_00743399	0.35	4.17	0.2	0.33	0.27	0.56	1.69	1.84	1.61	0.27	0	0.1
1-2.c52471/1/1434	Myozenin-1-like [Xenopus la	148237556	NP_00107945	576.91	44.31	668.79	774.85	814.46	621.95	828.81	792.86	784.05	264.47	233.52	236.76
1-2.c43476/1/1419	ubiquitin thioesterase zranb1	183986621	NP_0011169	0	14.62	27.91	36.85	24.01	1.15	14.67	7.45	19.34	7.9	0	0.08
1-2.c22654/3/1153	troponin T [Hyla chrysoscelis	69219560	AAZ04163.1	10122.26	2073.04	17965.94	14147.51	14757.52	11129.84	13493.94	17624.33	12549.12	5273.14	3959.14	4385.19
2-3.c13804/1/2225	PREDICTED: nebulin isofor	847166512	XP_01282661	11.15	0	36.25	2.49	15.18	0	47.98	17.58	0	0	0	0
3-6.c8447/1/6433.3	--	--	--	10.51	177.42	5.42	25.46	5.4	24.73	11.36	4.77	10.78	161.06	295.8	123.95
2-3.c39659/1/2162	PREDICTED: UTP--glucose-	847127397	XP_01281836	198.01	14.37	97.18	183.91	155.72	23.49	174.48	159.15	184.01	52.9	42.17	58.82
1-2.c25374/1/1478	PREDICTED: rho GTPase-a	847148808	XP_01282258	3.25	10.44	1.95	5.89	2.18	4.92	3.9	3.5	3.18	43.72	65.83	50.32
2-3.c13258/1/2172	PREDICTED: cytoplasmic dy	530648788	XP_00531093	0	0.63	0	1.01	0.99	0.46	0.65	0	0	9.44	4.43	6.52
3-6.c2720/1/3163	myosin heavy chain, partial [	4249697	AAD13770.1	1.27	0	4.48	3.18	3.38	0	3.8	3.51	3.79	0.72	0.77	0.45
1-2.c27238/36/1638	Hepatoma-derived growth fac	226372168	ACO51709.1	0	0	0	0	0	2.02	0	0	0	15.27	0	15.97
1-2.c17518/1/1484	PREDICTED: UAP56-intera	512859648	XP_00491695	12.79	12.04	17.34	22.3	18.44	73.95	40.54	15.67	23.18	4.7	10.43	4.83
2-3.c3125/5/2074	PREDICTED: myosin-4 [Xe	847156920	XP_0128244	49.69	17.41	0.89	35.76	25.31	2.82	61.61	76.61	56.17	14.72	44.77	4.72
2-3.c51962/2/2315	tln2 protein [Xenopus (Silura	166796755	AAI59153.1	16.48	6.8	20.23	21.01	15.83	11.84	22.75	15.33	24.53	9.05	4.54	3.15
3-6.c16377/1/3543	myosin, heavy chain 4, skelet	47575800	NP_00100124	1.22	0	3.15	2.26	1.15	0.04	2.3	1.55	1.74	0.16	0.13	0.25
2-3.c4190/2/2570.1	--	--	--	0	0	0	0	0.64	0	0	0	0	2.65	8.78	13.12
3-6.c14606/1/3830	myosin, heavy chain 4, skelet	47575800	NP_00100124	1.38	0.12	3.23	1.91	2.21	0	1.17	3	2.19	0.29	0.11	0.42
2-3.c5041/1/2291	PREDICTED: collagen alpha	847166754	XP_01282667	5.65	79.3	18.6	13.42	13.81	108.81	19.12	11.15	17.8	6.53	2.57	2.89
2-3.c50572/2/2187	--	--	--	11.8	18.8	8.48	6.28	0	0	0	0	0	10.89	0	10.96
1-2.c13300/5/1445	PREDICTED: chondroadheri	512871774	XP_00491854	37.37	30.31	61.06	47.24	15.89	22.7	23.62	47.41	36.05	14.39	11.69	4.76
1-2.c6247/1/1538	LOC100144989 protein [Xen	165971120	AAI58328.1	3.46	0.42	1.96	2.55	1.09	1.77	8.17	5.44	4.31	1.59	0	0.68
2-3.c36665/1/2620.	von Willebrand factor A dom	148228482	NP_0010799	3.85	3.52	1.91	1.08	3.27	3.58	4.76	1.46	1.33	25.1	29.78	49.2
3-6.c10857/1/3453.	--	--	--	53.88	3.2	5.23	17.63	25.33	0	14.83	35.39	41.28	0	0	13.93
1-2.c24095/1/6125.	isocitrate dehydrogenase 2 (N	148227256	NP_00108032	246.57	126.31	221.86	212.81	166.21	200.52	272.81	182.83	260.56	61.43	89.2	58.35
1-2.c2224/21/1361	LOC100126641 protein [Xen	213625404	AAI70536.1	83.34	12.28	71.04	82.33	67.31	46.34	55.75	71.79	73.02	21.15	21.34	16.28
3-6.c18664/2/3307	PREDICTED: C-Maf-inducin	512834466	XP_00293818	4.16	10.99	5.17	2.88	6.59	2.58	0.6	0	1.26	8.78	13.02	9.68
1-2.c24920/1/1755	PREDICTED: phosphorylase	512838308	XP_00293171	0.97	0	0.9	0.68	1.98	0	2.15	2.55	3.08	0	0.5	0
3-6.c5151/1/2988.1	--	--	--	12.47	1.03	3.3	19.38	5.14	1.18	11.09	3.84	6.92	1.71	0.38	1.35
2-3.c3096/1/2595	--	--	--	12.57	1.29	1.53	5.26	4.34	0.44	1.87	6.23	5.7	0.2	0.98	0.39
2-3.c7037/1/2274	exocyst complex component	189217627	NP_0011212	5.43	0.71	4.46	1.57	1.65	0	1.22	3.08	3.08	0	0	0.5
2-3.c40175/1/2071	serine hydroxymethyltransfer	148231095	NP_00108035	2.29	16.56	6.17	3.1	3.49	0	3.13	2.27	4.22	0.95	0	0
1-2.c22016/2/1687	phosphofructokinase, muscle	148224874	NP_00108692	66.86	7.06	19.64	71.16	39.36	17.24	70.87	45.28	89.85	17.21	19.06	22.66
1-2.c45402/1/6314.	PREDICTED: actin, alpha ca	637243920	XP_00321452	129.93	16.16	184.13	331.63	190.68	751.4	425.39	344.31	258.08	71.17	27.27	183.01
2-3.c35042/1/1961.	--	--	--	1.1	0	0	0	0	2.95	0	0	0	8.61	13.13	1.55
3-6.c18588/1/3218	PREDICTED: myosin-4 [Xe	847156920	XP_0128244	0.89	0	2.89	1.81	1.82	0	2.27	2.19	1.83	0.23	0.14	0.32
1-2.c5812/2/1262	--	--	--	18.66	30.12	13.83	18.1	16.88	63.05	13.91	18.51	19.93	237.62	282.82	156.67
2-3.c63531/2/2181	PREDICTED: myomesin-2 is	847130012	XP_01281893	3.71	2.35	4.82	6.2	4.74	9.97	8.96	5	11.09	2.81	0.79	1.45
3-6.c18765/1/2900	myosin heavy chain, partial [	4249701	AAD13772.1	7.44	0.06	14.11	8.89	11.04	0.11	7.87	14.66	10.66	3.47	2.33	2.94
1-2.c36266/1/1185	PREDICTED: cathepsin L is	847145572	XP_01282199	1.89	45.25	0.17	3.11	1.14	64.27	0.88	0.89	1.92	38.62	22.29	16.06
1-2.c7055/2/1286	hexokinase 2 [Xenopus laevis	148230649	NP_0010906	0	0.08	0	0	0	0.41	0	0	0	4.03	5.49	4.44
1-2.c52446/3/1680	PREDICTED: myosin-4 [Xe	847156920	XP_0128244	4545.7	115.76	8029.94	6320.53	6440.73	47.91	4541.1	8667.25	5312.41	2052.55	1302.31	1499.33
2-3.c32335/1/1823	Chain B, Structure Of The Ci	198443360	3EB6	2.91	4.33	1.43	0.68	0.71	0.68	2.44	2.12	4.48	0.69	0	0
2-3.c50438/1/2064.	--	--	--	0.97	2.2	1.7	0.32	0.3	0	0.37	1.72	2.4	0	0	0
2-3.c45452/2/2478	PREDICTED: phosphorylase	847120381	XP_0128171	5.6	1.61	2.48	13.29	5.69	2.27	8.07	6.38	10.65	1.48	2.74	1.56
3-6.c16262/1/3102	--	--	--	24.65	0.99	18.47	36.51	40.87	4.27	11.8	21.95	35.22	4.54	2.15	5.82
1-2.c36782/1/1589.	Keratin, type I cytoskeletal 1	678146122	KFV15273.1	1525.24	432.52	1017.38	1398.28	525.66	312.37	1266.33	1403.73	1096.37	584.44	272.73	339.58
1-2.c46183/1/1981	actin, alpha cardiac muscle 2	45361557	NP_989355.1	0	0.5	2.67	0.37	2.09	0	1.79	3.42	1.49	0	0	0
1-2.c5100/9/1726	plasminogen activator inhibit	47575766	NP_00100122	377.45	38.59	287.35	334.22	198.05	308.35	324.37	219.91	353.34	96.44	114.52	73.86
3-6.c19042/4/3303	alanyl-tRNA synthetase [Xen	189217804	NP_00112134	4.96	12.26	3.47	1.51	2.81	14.42	4.09	2.73	2.17	0.23	0.2	0.84
1-2.c39495/2/1625	PREDICTED: laminin subun	512844752	XP_00293635	32.15	32.31	29.61	24.33	13.71	26.69	21.24	30.1	15.45	7.02	2.18	7.96
2-3.c28428/1/1913	PREDICTED: tropoelastin 2	847162839	XP_01282564	60.97	1.05	37.3	82.95	25.88	0.34	57.1	48.47	30.1	13.73	11.43	14.58
1-2.c46644/1/1707.	cytosolic 5'-nucleotidase 3A [	187607205	NP_00112013	13.9	1.79	25.52	49.67	29.72	15.56	39.24	32.93	39.94	2.62	6.41	18.93

1-2.c50494/1/1564	PREDICTED: retinoic acid r	847104579	XP_01281331	6.57	45.93	7.53	9.03	3.33	14.67	10.66	5.33	9.21	91.5	119.81	106.7
2-3.c26792/1/2570	LOC734179 protein [Xenopu	84570116	AAI10785.1	154.01	173.49	142.2	108.04	123.5	148.59	108.3	114.18	134.52	35.34	23.55	49.68
2-3.c48518/1/2135	PREDICTED: LOW QUALI	731286412	XP_0106115(	0	0	0	0	2.59	7.73	0	4.71	4.35	0	0	0
2-3.c48098/1/1874	PREDICTED: band 4.1-like	847168904	XP_0128081	9.59	0.16	8.84	1.34	0.99	0	7.22	3.19	7.67	0	0	2.43
1-2.c35435/1/7811.	uncharacterized protein LOC	148226670	NP_0010797	11576.77	210.62	13763.67	16985.47	10917.86	576.36	7839.12	8821.66	18449.94	3988.51	1522.33	2571.07
2-3.c16841/1/2252.	PREDICTED: metastasis-ass	512877662	XP_0049192(	0	0	3.28	1.52	0	1.08	0.86	0	0	5.64	10.39	8.55
2-3.c42759/1/2146	--	--	--	13.06	168.2	29.22	21.78	15.51	62.56	16.56	19.71	25.62	7.86	4.67	4.49
1-2.c20805/3/1418	tripartite motif-containing pro	56118797	NP_0010081	22.06	14.74	24.81	23.39	19.69	39.49	32.82	33.03	28.44	8.31	16.96	2.7
1-2.c26073/8/1390	thrombospondin-4 precursor	118404528	NP_0010726	65.13	76.06	81.75	45.44	34.2	47.18	31.65	48.47	50.36	11.62	13.78	12.47
1-2.c6780/1/1630	PREDICTED: uncharacterize	847117050	XP_01281612	0.28	1.58	0	0.03	0.05	2.22	0	0	1.27	10.72	10.58	12.13
2-3.c39403/1/2577.	tubb4 protein [Xenopus (Silu	39794463	AAH64270.1	9.05	3.45	0.75	3.44	1	0	5.4	4.45	0	0	0	0
1-2.c45716/1/1583	PREDICTED: flotillin-2 isof	847099593	XP_0128122	20.57	0	9.9	5.76	4.47	0.17	7.85	10.68	17.21	0	0	6.64
2-3.c2925/1/2146.1	--	--	--	5.95	6.22	4.68	4.88	8.75	10.31	7.88	8.24	13.76	1.25	2.26	1.14
1-2.c8806/8/1658	LRRN4 C-terminal like [Xen	163914763	NP_0011066(	7.91	13.63	14.28	21.65	21.59	10.81	32.41	9.72	15.45	226.26	226.84	217.3
1-2.c39777/1/1466.	carbonyl reductase 3 [Xenopu	160420331	NP_0010853(	1.2	0	3.3	0	1.14	0	7.2	1.69	1.91	0	0	0
3-6.c13122/2/3182	PREDICTED: collagen alpha	512847530	XP_0049153	2.73	0	5.15	3.74	0.61	0	3.57	9.01	3.02	0	0	0.34
1-2.c20977/1/1258	PREDICTED: aminoacyl tR	847087804	XP_0128186	34.66	81.13	68.35	28.34	46.5	262.48	44.21	31.71	34.15	12.41	5.42	11.04
1-2.c52443/4/1318	PREDICTED: complement f	847125972	XP_0128179	0	0	1.31	0.69	0.85	1.71	1.08	0	0	6.66	11.29	16.68
3-6.c13377/1/3989	PREDICTED: obscurin isofo	847141081	XP_0128212(	15.02	5.08	23.88	21.96	18.5	25.24	21.97	19.75	31.81	6.35	6.1	9.29
1-2.c43239/1/1284	PREDICTED: serine/threoni	795092231	XP_0118279(	0	0	0	6.93	0	0	0	0	0	4.82	5.32	3.2
2-3.c24246/1/2185	PREDICTED: sarcalumenin	733909809	XP_0107176(	4.01	0	0.86	1.91	0.96	0.56	1.71	2.3	4.22	0.38	0	0.35
2-3.c4983/1/2188	microtubule-associated protei	147902447	NP_0010822	15.4	1.52	9.48	7.83	10	0.64	3.57	10.18	13.13	1.62	2.35	0
2-3.c54427/1/2105.	S-adenosylmethionine mitoc	52345544	NP_0010048	4.23	5.59	1.45	0	0.38	2.16	1.73	1.32	2.09	0	0	0
2-3.c55056/1/2345	--	--	--	4.68	1.66	5.55	6.11	3.95	3.52	3.19	6.56	6.87	0.55	0.41	2
1-2.c49998/1/1339	immunoglobulin superfamily	148226668	NP_0010906	0.57	2.2	0.3	0.85	0.35	1.18	0	0.83	0	14.45	13	5.5
2-3.c40633/1/2734	--	--	--	0.16	0	0.02	1.24	0	0	0	0	0	2.69	0.76	2.02
2-3.c63445/11/1962	myosin heavy chain, partial [	4249697	AAD13770.1	11.8	3.68	432.58	328.89	111.81	0	845.98	182.31	296.92	11.66	18.62	8.87
1-2.c44796/1/1824	olfactory receptor family C s	329130754	AEB77813.1	1.56	0.18	1.21	0.38	0.67	1.18	1.14	2.4	2.95	0	0	0.27
3-6.c19155/1/4056	PREDICTED: myosin-4-like	557022470	XP_0060114	1.62	0.14	3.96	2.88	3.08	0	3.04	4.26	2.85	0.93	0.69	0.5
1-2.c32939/1/1565	--	--	--	1.19	4.67	1.22	1.57	2.98	1.95	2.3	1.32	1.05	21.39	17.17	36.63
1-2.c43414/1/1140	coronin, actin binding protein	148230198	NP_0010837	3.22	12.75	5	11.75	6.08	10.01	11.15	0.85	8.47	113.07	76.42	70.3
1-2.c29211/1/1556.	nucleolar protein 16 [Xenopu	147902850	NP_0010857	0	11.53	0.2	9.69	0	0	0	9.98	8.71	0	0	0
3-6.c4057/1/3596	PREDICTED: proline-rich nu	224048441	XP_0021980	15.42	5.96	5.61	6.72	8.61	1.91	3.96	9.35	7.47	2.44	0.76	1.73
1-2.c4205/2/1195.2	PREDICTED: dual adapter f	847087333	XP_0128170	16.7	15.45	2.13	9.39	0	6.05	8.65	0.57	0	62.42	35.31	100.95
2-3.c21559/1/2315.	--	--	--	19.21	227.27	13.84	24.04	21.66	92.82	26.89	26.8	34.18	427.38	345.02	311.6
2-3.c22471/1/2756	PREDICTED: uncharacterize	672020667	XP_0087744	3.56	0.04	1.86	0	1.77	2.08	0.03	0	3.53	16.61	12.59	21.85
2-3.c34931/1/2250	PREDICTED: collagen alpha	847130150	XP_0128189	2.02	30.37	6.88	3.45	0.86	0	3.81	3.24	2.94	1.01	0.3	0
3-6.c6699/1/2765.1	PREDICTED: glycerol-3-ph	637265166	XP_0081021	246.6	11.34	179.34	349.57	215.93	74.31	156.61	187.27	273.18	66.2	35.13	90.32
1-2.c40054/1/1441	--	--	--	0	10.68	3.81	1.47	3.51	4.55	0	0	0.82	10.82	8.48	9.63
2-3.c3107/1/2035	--	--	--	7.39	66.55	3.63	5.32	9.07	26.24	5.51	4.82	7.28	84.66	55.53	78.22
1-2.c39075/1/2153	calsequestrin 1 (fast-twitch, s	148231736	NP_0010802	24.45	3.19	41.87	26.55	42.83	36.44	35.48	40.78	40.68	15.31	11.94	8.36
1-2.c41615/1/4512.	--	--	--	27.36	170.96	23.75	35.19	21.9	121.07	78	34.16	38.51	673.05	656.58	442.33
3-6.c14818/1/3070	PREDICTED: myomegalin-l	847014351	XP_0128043	2.15	2.99	3.6	4.47	2.14	6.46	2.39	6.2	7.11	0.62	1.05	1.4
1-2.c28878/1/1600	PREDICTED: caveolin-2-lik	301611463	XP_0029352	3.36	4.48	6.53	0.91	2.66	14.81	2.08	2.5	2.7	0.17	0.13	0
1-2.c14110/1/1280	PREDICTED: myosin-4-like	301611451	XP_0029352	7.69	0.55	11.58	8.75	9.91	0	7.46	12.57	11.94	2.29	1.85	2.01
1-2.c41790/1/1707	myosin heavy chain, partial [	4249701	AAD13772.1	25.56	0	59.93	50.96	46.42	0	31.94	53.81	48.01	20.14	10.82	10.62
3-6.c14798/1/3775	actinin, alpha 4 [Xenopus lae	147901835	NP_0010870	0	7.16	1.92	3.05	0	0	4.63	2.6	1.74	1.66	0	0
2-3.c33756/1/1891	PREDICTED: phospholipase	512861567	XP_0029369	0.64	0	0	0	0	0.94	0.01	0.14	0	16.59	26.87	2.89
1-2.c48539/1/1240	Map2k3 protein [Xenopus la	50604142	AAH77760.1	0	0	0	3.3	0	0	0	0	0	8.37	16.03	2.08
3-6.c11195/1/2917	--	--	--	25.85	1.18	12.8	23.41	18.18	4.73	16.55	16.46	21.93	5.03	2.93	7.49
2-3.c43740/1/2261	GTP-binding protein 2 [Xeno	118404418	NP_00107271	36.35	9.39	14.93	15.06	34.81	5.86	38.76	31.35	44.66	9.62	13.28	12.28
1-2.c43822/1/1783.	--	--	--	29.69	0.87	7.89	14.78	13.94	3.54	11.04	18.61	21.54	2.59	1.86	3.02
1-2.c28122/2/1552	G protein-coupled receptor ki	54606877	NP_00100611	12.96	6.25	2.21	8.3	0.12	0	4.02	0	0	21.78	13.82	26.99
3-6.c13602/2/3485	alpha 1 type I collagen [Rana	3242649	BAA29028.1	0.46	7.66	1.17	0.84	1.06	7.73	1.12	0.57	1.46	0.13	0	0
2-3.c60332/1/2006.	--	--	--	10.98	250.46	12.84	13.18	15.42	286.82	16.38	7.59	20.08	204.24	225.61	103.98
1-2.c14958/1/1888.	--	--	--	415.2	170.23	256.39	376.95	186.57	11.03	50.41	262.73	200.22	6.82	4.67	10.71
1-2.c2795/3/1437	PREDICTED: cold-inducible	837829631	XP_0045956	0	13.34	5.44	0	0.31	7.14	0	0	0	22.26	35.31	0
2-3.c23031/1/2056.	PREDICTED: LOW QUALI	637377851	XP_0081232	854.83	122.56	1834.94	1809.22	1690.84	833.33	804.33	1508	1831.44	289.31	238.58	783.15

2-3.c34033/1/2055	PREDICTED: filamin-B isof	512839159	XP_00491422	0	0	0	1.06	0	1.1	1.16	0	0	8.6	11.49	6.41
2-3.c4581/11/1993	tyrosinase-related protein 1 p	148223219	NP_00108049	1.6	3.85	3.53	0.85	1.62	1.26	5.33	0.9	2.61	0	0	0.21
2-3.c53835/1/2033	semaphorin-4A precursor [X	166158256	NP_00110731	0	0	0	0	0	1.21	0	0	0	9.96	10.64	0
3-6.c4089/3/3443	arginase-2, mitochondrial [X	118404488	NP_00107268	38.48	9.97	33.42	7.83	42.58	0.97	53.87	31.32	27.28	0.03	5.73	26.15
1-2.c28006/1/1862	PREDICTED: zinc finger pro	558191053	XP_00612944	0	2.53	0	0.47	0.62	0	0.06	0.05	0	4.61	4.33	2.36
2-3.c28093/2/2423	PREDICTED: PHD finger pr	301618658	XP_00293871	0.39	0.02	0	0	0	3.49	1.28	1.64	3.11	0	0.56	0
2-3.c54547/1/1876.	--	--	--	1.56	16.06	0	0.78	0.64	1.77	0	0	0	12.92	26.59	5.71
1-2.c8228/2/1609	myozenin-1 [Xenopus (Silura	55742226	NP_00100671	29.77	5.09	44.72	43.65	41.66	33.3	46.68	40.63	46.02	11.3	12.03	16.21
3-6.c10187/1/3370	predicted protein [Nematoste	156383368	XP_00163280	3.97	1.19	4.87	4.75	7.32	3.55	5.65	6.5	6.55	1.48	1.61	1.59
2-3.c16797/3/2253	matrin-3 [Callithrix jacchus]>	327365358	NP_00119216	0	0	0	0	0.76	0.72	0	0	0	2.39	1.6	2.64
2-3.c46230/1/2482	PREDICTED: LIM domain a	847100858	XP_01281271	1.27	1.28	0	1.52	0.08	0.67	0	0	0.95	3.68	6.95	11.08
1-2.c18000/1/1618.	PREDICTED: tubulin alpha	847163580	XP_01282588	0	103.6	0	0	83.65	305.9	260.05	126.93	0	0	1.5	0
3-6.c8118/1/3064	PREDICTED: E3 ubiquitin-p	821474903	XP_01240099	11.29	0.06	0.08	2.93	5.49	0.25	5.01	6.8	5.53	0.99	3.2	0.29
2-3.c32273/1/2544	PREDICTED: integrin alpha-	530603879	XP_00529368	0.5	5.27	1.12	0	0.57	0.8	0.11	0.52	0.05	3.85	5.09	10.44
2-3.c27373/1/2014.	PREDICTED: glycerophosph	723552948	XP_01029893	4.91	2.92	1.96	2.44	5.51	0.71	3.41	3.88	0.59	29.25	26.56	64.09
1-2.c23180/2/1443	TPA_inf: tropoelastin 2a [Xe	296040422	DAA12508.1	5.64	0.18	3.92	7.42	1.59	0	4.28	7.7	7.08	1.17	0.27	1.38
1-2.c34298/1/1277	PREDICTED: plexin-B2 isof	847110120	XP_01281457	5.35	6.95	0	2.77	1.99	3.58	1.97	0	0	20.97	12.88	10.2
1-2.c15042/2/1694	PREDICTED: glycogen debr	847125952	XP_01281792	10.07	0	8.2	18.91	6.25	2.37	5.73	7.13	15.11	1.57	2.36	1.52
2-3.c53647/1/1961	--	--	--	9.49	16.74	9.14	7.16	2.88	3.99	3.56	6.86	5.7	1.35	0.78	0.7
2-3.c23205/6/2632	PREDICTED: uridine-cytidir	611978749	XP_00747556	30.24	0.84	20.03	15.55	17.44	0.43	8.43	16.46	19.37	2.54	3.81	6.09
2-3.c26640/1/2181	--	--	--	1.9	2.68	1.81	0.92	1.6	1.59	4.56	2.59	4.73	0	0.15	1.29
1-2.c4105/2/1321	PREDICTED: vimentin A2-l	512841962	XP_00293220	311.7	6.5	290.73	254.46	140.29	0.13	271.63	308.18	239.39	112.63	168.61	17.37
2-3.c24094/1/2637.	PREDICTED: F-box only pro	512825033	XP_00491234	27.58	1.16	12.28	21.96	27.91	5.59	16.03	18.81	16.5	5.02	3.93	5.61
2-3.c61732/1/2281	--	--	--	33.72	20.78	36.51	37.44	51.06	14.87	50.42	56.89	83.32	17.49	16.49	26.14
1-2.c10711/3/1356	cordon-bleu WH2 repeat prot	148230755	NP_00107964	32.66	4.13	10.41	16.02	24.08	7.95	21.96	12.35	19.77	0	11.54	2.69
1-2.c29873/1/1706.	--	--	--	0	0	0.93	0	1.36	0	0	0	0	13.86	8.4	8.35
3-6.c10794/1/3009	PREDICTED: RNA-binding	512850834	XP_00294294	0.61	0	0.9	0.48	0.74	0.56	4.24	1.84	1.01	0	0.34	0.14
1-2.c11618/11/1377	Ras-related associated with d	148236071	NP_00108621	10.99	85.95	6.96	1.49	6.22	24.24	1.41	2.29	2.95	36.42	40.86	26.61
3-6.c5455/1/4108	PREDICTED: collagen alpha	512867463	XP_00293207	8.51	60.49	19	9.37	16.36	65.29	7.71	12.68	8.6	2.66	2.17	3.63
1-2.c19967/2/1694	PREDICTED: nebulin isofor	847166504	XP_01282661	15.93	8.31	88.3	54.85	0	0	63.55	42.51	30.33	18.16	4.41	17.52
3-6.c15944/1/2780	PREDICTED: LOW QUALI	512830904	XP_00293188	2.01	9.99	1.03	1.67	2.8	20.98	2.73	0.47	2.81	0.28	0	0.12
1-2.c22406/1/1175	alpha 1 type I collagen [Rana	3242649	BAA29028.1	0	0	0	0	0	67.06	0	0	0	9.96	30.54	5.9
1-2.c14613/1/1438	PREDICTED: myosin-4 [Xe	847156920	XP_01282446	59.62	4.64	508.5	236.81	390.54	0	171.33	193.68	109.3	53.24	31.27	68.77
2-3.c53936/1/1898	adenylosuccinate synthetase i	148236946	NP_00109001	17.72	5.77	13.29	16.84	16.79	4	12.26	12.03	19.66	3.95	4.48	3.32
2-3.c39576/2/2309	--	--	--	1.59	0	0	0.53	0	3.49	2.61	1.74	1.52	0	0.17	0.22
2-3.c22993/1/2150	PREDICTED: multidrug resi	512867941	XP_00293226	11.84	10.71	10.02	8	9.18	6.48	7.18	11.08	10.28	1.77	2.81	2.58
1-2.c49269/1/1268	--	--	--	2.18	64.05	4.02	2.67	5.62	15.91	5.93	7.11	4.67	0.66	0.52	0.92
1-2.c15193/2/1736	F-box only protein 31 [Xeno	118405062	NP_00107252	118.51	5.23	24.77	63.8	39.17	6.45	34.29	50.4	52.91	10.6	21.37	12.81
3-6.c14638/1/2869.	--	--	--	55.5	31.41	28.63	38.57	53.63	101.1	176.79	100.32	121.28	46.73	45.32	33.25
2-3.c12114/1/2207	UBA domain containing 1 [X	148235257	NP_00108937	1.02	0.91	1.33	1.5	1.57	0.59	2.52	1.01	1.76	0.24	0	0
1-2.c29231/1/1405	PREDICTED: microtubule-a	847101702	XP_01281288	2.1	0	0	3.02	0	0	0	0	0	24	42.31	0.25
2-3.c29740/2/1873	ryanodine receptor beta isofo	29501272	BAA04647.2	0	0.41	0	0	0	0	1.97	1.29	0.5	0	0	0
2-3.c45754/1/2163	WD repeat and SOCS box-co	45361625	NP_989387.1	9.03	45.92	10.35	10.26	8.62	20.29	13.18	14.8	13.6	1.75	3.2	6.02
1-2.c30305/1/1448	--	--	--	6.1	0	0	0.8	0	7.95	6.57	0.95	0	26.85	46.04	32.2
3-6.c12338/5/2777.	PREDICTED: uncharacterize	755867960	XP_01129149	0	11.34	5.91	0.11	1.47	6.02	0.15	0	0.24	93.66	129.63	0.39
2-3.c54707/1/2384	serine/arginine repetitive mat	89269017	CAJ83937.1	0	1.14	1.13	1.42	0.79	2.68	0.17	0	0	2.84	7.12	2.46
2-3.c9829/10/1911	PREDICTED: dihydrolipoyl	699633727	XP_00990847	27.74	17.91	24.72	31.03	37.16	15.37	19.11	33.43	36.72	9.52	3.48	13.07
2-3.c62155/4/2317	--	--	--	63.89	27.16	25.32	120.72	24.75	21.37	34.25	46.02	121.7	5.09	0.68	5.13
1-2.c36134/1/1827.	--	--	--	6.55	61.87	5.04	6.53	4.51	9.82	6.24	3.95	8.04	70.41	112.32	54.54
2-3.c20854/1/2262.	PREDICTED: alpha N-termi	301608515	XP_00293383	0.31	1.47	1.51	2.57	1.91	10.19	3.46	1.48	2.4	0.18	0.07	0.15
2-3.c2148/11/2244	PREDICTED: zinc finger pro	847155335	XP_01282413	7.67	0	1.34	3.09	0.77	0	0	2.68	3.76	0	0	0
1-2.c22227/1/1315	PREDICTED: RNA-binding	847153751	XP_01282364	0	0	0	4.91	0	0	0	4.58	5.43	0	0	0
1-2.c50117/1/1669.	--	--	--	9.51	4.48	0	6.3	6.3	4.93	5.88	5.21	3.2	53.41	60.16	81.97
2-3.c35670/1/2476.	--	--	--	30.3	9.12	26.01	36.51	16.04	4.47	28.17	23.14	42.67	6.74	5.55	5.71
1-2.c22683/1/1296	--	--	--	3.87	4.26	19.48	11.96	10.03	12.17	16.2	14.23	22.15	6.89	1.92	3.79
2-3.c34496/1/2623	--	--	--	2.3	0.87	3.97	1.31	3.81	10.53	2.46	1.82	3.02	0.14	0.39	0.31
1-2.c30327/1/1573	--	--	--	4.62	22.72	0.89	5.52	5.02	11.61	5.85	3.9	1.5	38.71	34.42	72.34
2-3.c42249/1/2138	PREDICTED: glycogen phos	556954754	XP_00598898	9.97	0.21	4.8	7.25	7.31	2.19	7.53	8.77	11.54	2.42	2.09	2.02

1-2.c29452/1/1285	myosin heavy chain, partial [	4249695	AAD13769.1	885.57	298.65	348.99	301.68	293.15	36.55	378.25	468.87	399.53	130.86	308.72	31.37
2-3.c15942/5/2592	protein SDA1 homolog [Xen	148236679	NP_00108249	7.83	13.03	3.47	1.6	4.15	10.6	4.93	4.26	4.02	0.2	0.93	1.32
2-3.c62459/4/2260	--	--	--	0	18.7	0	0	0.22	42.41	0	0.51	2.75	18.1	17.07	15.14
2-3.c34560/1/1945	predicted protein [Nematoste	156380608	XP_00163186	0.22	5.55	0	4.41	3.34	5.18	0.69	2.16	1.06	26.18	50.2	20.07
2-3.c34816/2/2585	--	--	--	6.42	5.2	8.18	8.41	11.89	8.11	7.21	11.19	11.15	0	1.37	5.87
1-2.c19643/3/1539	RecName: Full=Iroquois-clas	263432223	O42261.2	4.54	31.4	3.13	3.97	3.87	13.23	8.71	2.54	6.44	65.95	68.8	77.39
2-3.c53345/1/2484	PREDICTED: ATP-depender	512873634	XP_00491868	0.73	1.01	0.62	1.06	0	1.03	1.06	1.26	0.37	0	0	0
3-6.c14961/1/2834	alpha-catulin [Xenopus laevis	148237000	NP_00108661	1.59	3.07	5.16	1.92	2.88	1.06	1.78	1.83	3.85	0.28	0	0.6
2-3.c43309/1/2482.	transmembrane protein 63A [	147906330	NP_00108875	0	0	0	0	3.66	0.9	0	3.73	3.29	0	0	0
3-6.c16722/1/3197.	PREDICTED: transmembran	512856155	XP_00294200	5.85	8.38	0	0.02	0	4.98	0	0	0	0	7.07	7.97
2-3.c12478/1/2395	PREDICTED: collagen alpha	194224629	XP_00191509	0	10.52	1.49	2.1	0.22	0.73	2.86	0	3.01	0	0	0
2-3.c25794/1/2191	cysteine-rich secretory protei	73853836	NP_00102749	0.12	2.4	0.13	6.08	8.43	0.61	15.45	7.57	9.47	0	8.89	0
2-3.c14285/2/2291	alpha1 type II collagen [Cync	5360532	BAA82043.1	2.65	101.28	18.81	9.14	1.8	19.83	5.36	4.45	5.88	0.64	0.77	1.51
2-3.c22247/1/2406	N-acetylglucosamine kinase [	207028218	NP_00112869	0.21	0.34	0.13	0.28	0.37	0	0.41	0	0	5.96	3.4	5.25
1-2.c21055/4/1363	kelch-like protein 41 [Xenopu	45361273	NP_989214.1	19.82	24.26	0	8.24	6.92	69.42	36.06	20.18	32.87	5.84	18.33	3.27
2-3.c35028/1/2147	fragile X mental retardation s	147899444	NP_00108178	35.89	21.35	25.5	6.59	4	8.42	11.62	9.77	10.9	1.74	0.7	5.13
2-3.c40760/1/2202.	pyruvate kinase PKM [Xenop	148225037	NP_00108434	0	0	0.86	0	0.25	6.57	3.4	0	0	32.68	54.92	17.23
2-3.c60503/1/2316	PREDICTED: RAC-alpha se	301622901	XP_00294076	9.69	9.55	4.75	3.73	4.38	2.34	1.81	5.22	4.11	0	1.63	0.25
1-2.c12427/1/1791.	PREDICTED: glucose-6-pho	327288680	XP_00322905	129.29	1.95	37.58	76.07	0.94	12.84	60.64	36.18	73.37	1.99	8.78	37.89
2-3.c11851/8/2182	PREDICTED: collagen alpha	591360728	XP_00705528	3.68	66.52	8.95	3.16	2.11	28.13	0.8	1.6	3.64	0	0.27	0
2-3.c39422/1/2230	PREDICTED: collagen alpha	688603631	XP_00929327	5.66	1.11	4.63	3.79	7.08	1.28	2.66	5.13	6.66	0.49	1.18	1.01
2-3.c27411/1/2321	glycerophosphodiester phosp	187607081	NP_00112059	3.3	3.19	0	2.65	0	0.63	0.2	1.4	0	15.56	12.32	5.94
1-2.c46467/1/4679.	muscle creatine kinase [Xeno	147899942	NP_00108007	16399.49	1495.38	11744.97	11879.68	12243.56	6365.12	10657.14	10084.84	10078.52	4723.24	2327.34	3414.03
2-3.c36572/1/2031.	PREDICTED: suppressor of	847154706	XP_01282391	2.92	6.34	1.91	0.6	2.56	2.98	2.54	1.87	1.71	21.22	40.09	28.63
1-2.c7427/1/1839	--	--	--	0	3.88	0.37	4.82	0	0	5.44	0.95	3.99	0	0.41	0.42
2-3.c24282/1/2441.	--	--	--	21.86	249.75	12.93	26.75	16.54	34.45	20.61	18.29	22.51	226.79	393.38	179.47
1-2.c26920/1/1233	--	--	--	22.26	26.69	62.95	37.66	1.63	5.03	50.2	29.1	3.49	1.63	0	0
3-6.c3426/3/3257	hypoxia-inducible factor 1 al	166343757	ABY86629.1	4.08	5.91	0.47	4.71	5.37	2.7	8.67	3.14	4.45	1.19	0.85	1.67
3-6.c15179/1/6486.	myosin, heavy chain 4, skelet	47575800	NP_00100124	0.3	0	1.82	1.35	1.12	0	1.77	1.69	1.42	0.21	0.08	0.2
2-3.c61980/1/2022.	--	--	--	1.03	0.46	1.32	2.57	2.25	1.29	2.92	0.82	0.79	17.61	39.22	24.71
2-3.c11442/25/1920	lysine--tRNA ligase [Xenopu	47575822	NP_00100125	116.6	167.4	76.5	58.22	66.81	128.32	125.81	67.29	83.54	30.77	14.92	42.28
2-3.c45772/1/2259	pentraxin 3, long precursor [	148226508	NP_00109115	3.15	0.34	1.68	3.96	0.97	0.08	3.19	1.47	0.97	0.2	0	0.15
3-6.c10214/1/3383	PREDICTED: LIM domain-b	847142931	XP_01282174	1.49	0.13	6.25	2.63	3.01	2.12	1.93	2.94	4.91	0.18	0.22	1.37
2-3.c11813/2/2417	--	--	--	4.86	5.55	2.43	4.46	3.4	2.15	2	2.96	3.57	37.77	30.95	38.37
3-6.c2890/2/3785	PREDICTED: hamartin [Xen	512863241	XP_00294468	65.7	5.97	30.81	40.5	41.85	5.28	33.35	33.95	39.03	8.24	11.33	15.73
2-3.c7806/1/2314.1	PREDICTED: GMP reductas	847133898	XP_01281969	11.08	3.42	12.6	19.53	10.72	4.48	10.92	13.07	10.15	5.08	1.37	1.54
1-2.c43472/1/1865.	--	--	--	2.42	60.05	9.88	3.78	0	8.13	7.21	4.38	10.46	0	0	0
3-6.c4428/2/3338	uncharacterized protein LOC	148225160	NP_00108922	8.14	61.81	26.31	10.53	11.21	55.25	13.1	4.73	13.96	1.23	2.47	4.92
1-2.c24233/1/1527	protease, serine, 8 precursor [	148222777	NP_00108172	0	0	0	0.34	0	0.64	0	0	0	0.52	4.06	5.82
2-3.c2051/20/2321	heat shock 70kDa protein 9 (	148228693	NP_00108016	2.45	10.62	3.64	9.04	0.08	14.31	7.73	5.97	6.98	0	0.71	3.57
3-6.c12948/3/3596	PREDICTED: RNA polymer	637246018	XP_00810503	4.54	11.51	3.96	4.49	4.59	0.22	2.61	4.09	3.63	0	0	1.95
3-6.c11524/1/4750	PREDICTED: myosin-4-like	301611451	XP_00293525	2.96	0	5.39	4.97	4.91	0.08	5.77	6.83	4.33	2.74	1.29	0.98
2-3.c22311/1/2239	myozenin-3 [Xenopus (Silura	58332250	NP_00101127	0.82	7.19	5.26	0.6	1.05	39.01	1.83	0.76	1.88	0.07	0.05	0
2-3.c19084/3/2254.	leucine-rich repeat-containing	194332463	NP_00112373	0.32	0	0	0.83	0	0	4.44	0	5.68	0	0	0
1-2.c48481/1/1551	myosin, heavy chain 2, skelet	55742222	NP_0010068(	9.43	2.11	6.01	2.28	2.34	0.06	2.72	3.84	2.83	0.19	0.01	0.48
1-2.c36910/1/1866	PREDICTED: LOW QUALI	617600050	XP_00752244	0.59	5.25	1.28	0	0	1.12	3.42	0	3.35	0	0	0
2-3.c20294/1/2099.	--	--	--	4.68	59.64	4.25	9.47	3.59	12.73	6.39	3.67	6.62	72.55	102.49	48.33
2-3.c43208/1/2196	nuclear pore complex protein	148228663	NP_00108744	2.05	0	0	0	0	0	3.48	0	2.8	0	0	0
3-6.c11772/5/2807	collagen, type 1, alpha 2 prec	147898763	NP_00108072	16.76	0	0.03	0.03	1.28	81.86	21.3	11.73	12.91	14.18	0	0
1-2.c2629/9/1645	PREDICTED: nebulin isofor	847166508	XP_01282661	0	0.4	0.59	0.93	1.01	0	1.66	0.81	1.92	0	0	0
1-2.c31345/1/1465	PREDICTED: zinc finger BE	641785470	XP_00531296	1.12	86.91	5.9	8.47	6.13	18.2	2.11	3.97	10.24	78.74	107.22	63.2
2-3.c12321/2/1890	PREDICTED: LOW QUALI	697487074	XP_00967463	1.1	4.39	25.36	8.47	21.73	16.67	11.08	17.44	12.44	2.4	0	7.96
2-3.c12633/1/2379	--	--	--	3.24	10.63	3.52	1.68	1.89	8.29	0.98	1.16	1.21	13.67	10.55	25.32
1-2.c49741/1/1814	PREDICTED: NAD(P) transl	768393841	XP_01159260	138.52	154.92	97.47	219.84	108.19	83.81	192.99	228.2	122.15	70.72	73.84	54.16
1-2.c22029/3/1756	PREDICTED: glucose-6-pho	327288680	XP_00322905	254.95	84.96	243.48	464.04	340.73	83.04	255.93	252.17	503.78	121.7	47.12	168.89
2-3.c29321/1/2507.	PREDICTED: homocysteine-	602651855	XP_00743230	0	0	0	0	0	4.6	0	0	0	42.89	32.97	0
3-6.c13804/1/8671	myosin, heavy chain 4, skelet	47575800	NP_00100124	0.18	0	0.64	0.34	0.42	0	0.22	0.59	0.56	0	0	0
2-3.c10198/5/1962	PREDICTED: dual specificit	847134985	XP_01282003	15.48	23.16	0.06	0.8	4.5	14.43	2.79	8.16	8.69	2.96	0.67	0

3-6.c18172/1/2871	PREDICTED: myosin-3-like	557329602	XP_0060377(	0.12	6.96	1.41	0.8	0.43	58.63	0.68	1.1	0.35	0	0	0
2-3.c32898/1/2173.	--	--	--	0.48	7.57	1.66	4.08	1.16	7.83	0.92	1.87	1.74	24.09	61.49	52.87
2-3.c55241/1/2063	PREDICTED: DBH-like mor	512842438	XP_0029363€	0.01	0	0	0.6	1.37	0.51	0.21	0	0	3.59	3.85	4.65
2-3.c24749/7/2346	PREDICTED: dual specificit	524976634	XP_0050881	10.75	42.31	7.55	11.12	16.28	16.02	10.66	8.04	8.01	65.73	114.22	114.67
1-2.c25639/2/1346	actin, alpha sarcomeric/skelet	45360805	NP_989076.1	25.2	9.42	35.3	27.72	32.85	29.07	24.85	37.39	25.77	10.9	4.06	11.27
2-3.c12986/6/1975	T-complex protein 1 subunit	56118448	NP_00100787	74.01	128.8	62.68	43.98	39.95	129.6	55.41	63.64	30.91	26.26	23.02	5.64
2-3.c16642/1/2190	PREDICTED: cytoplasmic dy	847163111	XP_0128257	1.95	0	2.65	2.66	0	0	3.24	0	2.59	0	0	0
3-6.c15004/1/2682	PREDICTED: myosin-4 [Xer	847156920	XP_0128244€	1.76	0	2.44	1.28	1.12	0	1.31	1.16	1.4	0	0	0.16
2-3.c15470/1/2345	PREDICTED: E3 ubiquitin-p	821474903	XP_0124009	4.68	0.22	0	0.68	2.11	0.39	4.69	3.09	2.82	0.09	1.69	0.11
2-3.c58784/1/2454.	--	--	--	456.76	19.06	346.43	1465.39	555.65	135.67	860.62	604.03	999.71	427.23	213.92	210.8
1-2.c27518/1/1050	GTP-binding nuclear protein	678196368	KFV63350.1	215.88	361.38	156.61	112.94	156.91	166.04	148.93	147.39	128.32	44.5	32.74	64.17
3-6.c19150/1/2859	--	--	--	17.74	3.59	52.73	39.37	45.5	26.66	37.97	23.86	50.87	13.6	4.87	17.36
1-2.c46418/1/1352	PREDICTED: LOW QUALI	637377851	XP_0081232	117.68	15.44	233.03	244.89	241.96	123.97	105.66	210.04	249.18	50.37	38.31	103.41
1-2.c33004/1/3717.	actin, alpha cardiac muscle 2	45361557	NP_989355.1	17672	2925.13	25457.44	20359.02	23430.02	19339.6	17185.39	26544.81	18558.93	8634.64	5465.71	8510.04
1-2.c38142/1/1542	isocitrate dehydrogenase [NA	71896117	NP_0010255	72.2	19.56	48.44	38.52	51.05	36.71	29.73	43.94	34.11	10.56	11.31	12.97
2-3.c7616/2/2430.2	beta-hexosaminidase subunit	187607505	NP_0011206(	9.97	8.35	4.66	15.2	10.82	2.95	10.48	4.01	5.95	72.33	57.18	125.41
2-3.c16203/1/1780.	predicted protein [Nematoste	156353049	XP_0016228	2.53	12.82	4.77	5.02	1.1	6.61	4.14	2.84	4.78	36.21	89.15	51.91
2-3.c49559/1/2240	PREDICTED: starch-binding	301617462	XP_0029381	0.87	0	0	2.1	0.36	0	3.11	0.3	1.3	0	0	0
2-3.c30607/1/2473	--	--	--	8.83	4.69	13.06	9.83	35.36	6.24	13.85	8.21	26.1	2.5	5.2	1.93
2-3.c47252/1/2237	PREDICTED: bifunctional gl	512844004	XP_0029360€	18.34	42.38	26.21	17.47	19.15	27.35	18.87	19.55	24.11	5.31	4.24	9.53
1-2.c46430/1/1373	Ras association (RalGDS/AF	157426943	NP_0010987	0.99	3.7	1.4	3.32	0	0	0	1.23	0	13.42	11.83	9.98
1-2.c2165/15/1553	isovaleryl-CoA dehydrogenas	58332612	NP_0010113	75.74	50.9	64.54	86.27	64.29	40.96	51.24	51.18	67.29	17.06	23.9	17.21
1-2.c39980/1/9175.	PREDICTED: phosphate carr	847109028	XP_0128141	29.34	27.41	21.18	64.65	20	13.99	20.67	47.71	66.08	16.91	9.26	4.85
1-2.c49938/1/1557	PREDICTED: protein phosph	675610817	XP_0089340	6.5	0.01	8.9	4.78	6.44	7.96	2.23	0.04	2.32	20.12	30.56	16.33
1-2.c32094/53/1813	PREDICTED: fragile X ment	637372583	XP_00322991	122.39	91.91	111.96	82.65	137.97	182.29	145.65	87.35	115.7	19.84	29.1	66.14
1-2.c33054/1/1457	Sodium/potassium-transporti	565315033	ETE67008.1	0.77	2.78	0.8	1.13	0.56	4.12	0	0	0	11.64	9.97	0
1-2.c25421/1/3503.	serum paraoxonase/arylestera	148232543	NP_0010806	0.94	6.68	1.41	0.11	0.02	7.18	1.04	1.75	0.51	20.89	21.7	22.61
1-2.c24290/2/1780	--	--	--	6.69	43.42	3.12	2.7	4.8	19.25	11.38	2.07	6.44	72.96	78.8	65.57
3-6.c16779/1/3663	--	--	--	9.37	4.93	20.18	10.65	15.28	25.02	14.63	13.18	20.09	3.48	5.44	6.59
1-2.c8407/12/1662	SET and MYND domain-con	148235066	NP_0010856	50.86	89.61	45.41	42.35	34.15	37.59	38.42	37.01	45.9	16.01	10.42	13.3
2-3.c17756/1/1941	--	--	--	18.34	5.06	11.51	18.54	12.01	16.35	13.55	12.84	18.72	2.06	5.52	5.44
2-3.c18517/21/2109	PREDICTED: aldehyde dehy	729766577	XP_0105804	40.19	22.87	104.81	29.54	98.67	67.56	64.12	88.29	61.34	31.53	24.78	21.14
1-2.c14116/2/1473	growth hormone inducible tra	148231426	NP_0010800	35.17	0	0.9	0.5	9.97	11.97	10.93	14.87	11.96	0	0	8.3
1-2.c52252/13/1418	NADH dehydrogenase subun	146149047	YP_0011654	338.96	158.6	157.43	112.15	109.43	112.86	268.38	179.26	212.92	104.78	41.45	82.59
2-3.c15844/1/2603	collagen, type III, alpha 1 (El	147905732	NP_0010835	13.53	130.09	28.65	15.5	46.64	301.62	27.1	11.32	20.03	8.47	2.38	6.92
1-2.c25839/1/1782	72 kDa type IV collagenase [	148231336	NP_0010806	0.99	51.09	3.01	6.28	6.41	25.06	9.21	0.64	11.86	72.4	65.11	91.85
1-2.c30814/1/2510.	PREDICTED: LOW QUALI	637377851	XP_0081232	147.36	14.31	130.05	102.63	112.08	57.57	105.59	126.17	115.53	35.4	27.8	45.79
1-2.c49804/2/1328	--	--	--	0	0	0	0	0	0.31	0	0	0	48.78	119.87	0
3-6.c13467/1/3144.	serine protease PC5-A [Pelop	23266416	AAN10146.1	12.38	33.67	3.58	11.49	6.47	24.49	11.36	10.46	12.21	136.81	164.39	112.3
3-6.c14952/1/3047	PREDICTED: myomegalin-li	297279729	XP_0028017	1.86	5.15	4.73	2.37	5.93	12.22	4.91	4.49	4.33	0.9	1.1	1.24
3-6.c16868/1/3032	myosin heavy chain, partial [	4249701	AAD13772.1	1.54	0.06	3.56	2.67	2.8	0	2.28	4.13	2.24	0.57	0.13	0.84
1-2.c1985/36/1470	PREDICTED: 28S ribosomal	847132351	XP_0128193	29.15	47.48	17.94	15.48	13.47	25.65	18.41	19.04	21.5	5.11	9.87	2.95
2-3.c12253/1/1913	PREDICTED: serine/threonir	663259256	XP_0084914	3.89	6.03	0	0.56	0.8	2.29	0.01	0.78	0	5.82	4.18	12.42
3-6.c13919/1/3394	PREDICTED: myosin heavy	765166248	XP_0114919	4.26	0	7.15	5.33	6.05	0	5.35	8.38	5.13	2.89	1.06	1.43
2-3.c43226/1/2634	PREDICTED: talin-2 [Tinam	719733077	XP_0102179(	31.02	18.83	36.4	20.16	28.04	14.56	22.65	23.61	21.84	5.58	5.49	10.91
3-6.c18847/9/2929	PREDICTED: titin-like [Allig	564226854	XP_0062578	416.41	195.23	817.61	584.2	617.19	844.82	575.85	566.76	723.17	192.87	126.48	335.27
1-2.c43599/1/1472.	--	--	--	40.59	55.34	21.5	30.76	16.08	25.57	27.06	16.75	49.21	16.15	2.13	2.51
3-6.c5778/1/4745	myosin, heavy chain 4, skelet	47575800	NP_0010012	1.88	0	3.1	3.39	2.22	0.08	3.07	3.23	2.81	0.74	0.6	0.9
3-6.c18857/2/4593	myosin, heavy chain 4, skelet	47575800	NP_0010012	2.88	0	4	3.34	3.52	0	2.44	4.21	3.1	0.95	0.52	1
2-3.c15367/1/2233	--	--	--	3.01	0.09	6.05	2.71	0.93	0.08	2.51	1.18	3.01	0	0	0.54
3-6.c4654/1/3909	succinate dehydrogenase cytc	218847754	NP_0011363	12.28	10.51	20.9	13.73	15.44	7.77	16.08	7.24	14.38	3.54	1.61	6.14
2-3.c48842/1/4288	calsequestrin 1 (fast-twitch, s	148231736	NP_0010802	248.5	35.13	319.37	322.99	425.54	396.31	423.82	467.64	351.53	199.48	153.5	117.63
1-2.c11973/1/1605	--	--	--	0	0.72	0.21	1.01	1.53	8.73	0.16	2.43	4.51	0	0	0
2-3.c22693/1/2530	--	--	--	119.57	8.43	89.4	61.59	42.4	18.19	43.41	54.57	69.57	18.89	33.63	10.9
2-3.c19016/2/2318	--	--	--	0.77	22.02	1.46	3.09	0.92	7.9	3.61	1.58	3.91	41.76	77.3	31.98
2-3.c32849/1/11549	--	--	--	27	5.22	52.2	128.65	29.96	23.97	113.42	73.82	103.64	35.84	23.08	36.16
3-6.c10073/1/3198	PREDICTED: laminin subun	301607003	XP_0029331(	27.84	17.85	20.32	8.52	21.18	14.09	15.26	16.72	21.92	5.05	5.83	6.98
1-2.c25815/1/1333.	--	--	--	7.38	36	4.79	9.65	7.47	12.32	10.62	7.79	9.59	121.33	98.76	108.66

2-3.c55553/1/2548.	dual specificity mitogen-activ	56118586	NP_00100805	0	0	0	5.36	0	0	5.29	4.95	4.11	1.41	1.27	0
1-2.c23401/1/5855.	malate dehydrogenase, cytopl	148223868	NP_00108333	64.58	46.52	55.78	42.64	75.53	84.1	46.92	67.64	66.44	21.31	7.57	28.16
2-3.c43652/1/2096	--	--	--	3.12	48.03	1.52	1.77	2.48	17.05	5.35	3.74	3.8	36.27	54.25	56.01
3-6.c10137/1/3017	transforming acidic coiled-co	148235419	NP_00108196	0	0.26	1.63	0.88	5.95	5.55	4.1	3.4	3.36	0	2.73	0
2-3.c61198/3/2604	PREDICTED: protein PRRC2	512859823	XP_00294134	34.42	43.87	48.34	45.99	19.32	13.13	28.64	42.84	49.58	14.32	11.43	16.67
1-2.c11933/1/1564	--	--	--	5.45	1.34	7.11	3.27	3.94	1	2.25	5.69	3.77	0.64	0.36	0.46
2-3.c28219/1/2543	myosin, heavy chain 4, skelet	47575800	NP_00100124	752.54	24.16	1582.73	867.35	1229.02	6.31	759.32	1914.36	1079.6	160.16	101.44	285.79
2-3.c31633/218/220	solute carrier family 16 (mon	148232306	NP_00108030	122.62	13.08	55.81	258.74	174.23	16.97	144.8	74.82	180.14	39.11	28.71	67.94
2-3.c12268/1/6515.	calsequestrin-1 precursor [Xe	45360503	NP_988894.1	27.45	4.6	71.34	18.67	63.32	59.94	34.83	57.11	31.01	11.76	10.35	13.44
2-3.c6735/1/2573.1	--	--	--	28.56	25.8	14.76	15.45	9.82	12.88	27.53	17.14	13.06	6.06	5.98	5.25
1-2.c38610/1/3837.	PREDICTED: NADH dehydr	847118057	XP_01281647	281.77	90.18	183.55	202.8	200.5	129.47	152.58	192.29	200.66	46.5	69.88	82.37
1-2.c14792/1/1198	mesoderm-specific transcript	54262191	NP_00100578	2.78	1.65	2.94	2.18	5.37	4.14	4.11	4.99	3.43	0	1.2	0
1-2.c52378/3/1323	PREDICTED: myosin-4-like	847172870	XP_01280964	2.05	177.95	2.24	6.04	4.33	1146.28	4.46	6.86	2.87	0.47	0.94	0.5
2-3.c6101/1/2670	PREDICTED: mitogen-activa	847103308	XP_01281312	0	0.75	0.19	0.23	0	0.94	0.05	0.05	0	2.63	3.1	1.96
2-3.c8673/1/2757.1	--	--	--	10.71	1.04	4.69	6.22	3.24	2.01	4.23	3.33	6.37	0	1.2	1.7
3-6.c8630/1/2912	eukaryotic translation initiatio	148232020	NP_00108075	19.52	2.34	6.38	8.19	10.82	1.23	12.75	8.37	7.48	2	2.54	3.75
3-6.c11310/1/3133	PREDICTED: caveolin-2-lik	301611463	XP_00293525	1.23	13.74	14.96	1.71	4.98	30.04	3.2	1.74	3.54	0	0.57	0.9
1-2.c4011/6/1368	--	--	--	49.52	1394.44	126.9	111.01	73.89	786.52	96.53	82.86	163.24	65.49	32.85	24.59
1-2.c26347/1/1293	--	--	--	1.51	9.09	1.17	4.82	3.41	4.58	0	0	4.62	16.07	29.03	25.62
1-2.c19873/3/1329	--	--	--	0.09	0	0.57	0	0	2.84	0	0	0	10.34	13.77	0.14
2-3.c21899/1/1926	PREDICTED: peptidyl-proly	742150246	XP_01088046	3.48	0.43	5.65	4.4	3.59	5.99	8.43	5.08	7.4	2.11	0.72	1.76
2-3.c52553/1/2131.	PREDICTED: DNA-directed	589959545	XP_00699235	50.75	21.06	50.99	37.78	34.9	35.05	19.8	30.92	45.35	11.01	12.75	8.89
1-2.c45720/1/1318	--	--	--	44.12	23.97	61.62	61.79	51.34	62.17	106.1	86.75	69.42	49.07	15.2	27.01
2-3.c15608/1/2363	--	--	--	9.53	0.46	6.54	7.46	7.3	0.89	5.72	3.05	3.83	0	0	2.04
2-3.c20705/7/1948	phosphoglucomutase-1 [Xeno	47575814	NP_00100125	21.97	36.24	13	12.53	322.16	73.22	264.73	349.61	15.56	6.68	0	2.18
2-3.c41102/1/2363	--	--	--	9.78	0.07	1.51	5.65	5.58	0	6.11	2.16	2.15	0.43	0.83	0.3
1-2.c29138/2/1426	protein FAM134B [Xenopus	187608054	NP_00112044	11.58	0.33	1.67	5.58	7.92	0.12	6.08	3	4.09	0.16	1.33	0.2
2-3.c56659/1/2309	N-acetylglucosamine kinase [	207028218	NP_00112869	2.78	0	5.02	6.55	1.37	0	0	1.76	2.25	0	0	0
2-3.c45841/1/1987	--	--	--	2.62	2.94	2.07	0.75	0	0.67	0.27	1.09	2.6	0	0	0
2-3.c55480/1/2251	voltage dependent calcium ch	11414922	BAB18553.1	36.38	10.61	36.15	36.15	43.51	46.44	41.05	33.24	34.49	13.56	12.57	11.93
2-3.c25443/3/2326	--	--	--	4.58	7.11	7.13	5.37	6.98	5.53	4.96	9.92	6.41	0	0.32	4.63
1-2.c49544/1/3724.	PREDICTED: actin, alpha ca	874446319	XP_01295408	2.41	2.68	12.94	14.93	2.75	8.61	12.4	13.39	6.13	0.23	0.53	5.5
1-2.c6471/3/1383	PREDICTED: solute carrier f	847120590	XP_00293828	4.79	5.88	5.17	4.41	3.91	13.04	7.9	6.78	6.03	1.42	1.7	0.81
2-3.c34043/1/2095.	PREDICTED: aldehyde oxida	847167054	XP_00293776	2.29	1.97	1.07	2.32	5.93	1.78	3.18	4.96	0.32	31.24	48.4	97.09
2-3.c47134/1/2266	PREDICTED: glycogen [star	512855195	XP_00293927	15.98	0.38	13.39	21.05	16.16	2.04	8.68	13.21	8.72	1.36	1.67	5.41
2-3.c28854/1/2246	--	--	--	11.85	16.72	7.9	10.57	32.86	2.6	26.63	5.69	14.48	2.19	2.49	0.98
2-3.c50338/1/1853	--	--	--	0.75	0.35	1.27	1.53	0	0	0.17	0	0.33	4.77	8.06	4.98
2-3.c40900/1/2240	--	--	--	1.13	7.9	0.76	0.51	0.85	2.07	1.72	0.54	0.89	19.24	12.36	13.05
1-2.c46929/2/1290	phosphoglycerate kinase 1 [R	190701025	AAG33069.2	204.55	23.16	61.11	202.78	181.97	71.64	240.94	257.91	216.33	129.23	91.08	53.11
2-3.c50698/2/2094	--	--	--	0	0	0	0	0	0.71	0	4.83	3.28	0	0	0
2-3.c57948/4/2591	--	--	--	24.59	2.52	61.86	63.09	71.93	37.14	60.71	30.73	71.49	17.52	7.35	29.19
1-2.c8286/1/1512.1	--	--	--	0	0	0	0.31	0	0	0	0	0	4.09	4.86	6.15
2-3.c47722/1/2319	PREDICTED: myosin-4 [Xen	847156920	XP_01282446	5.63	0.59	10.45	7.96	9.62	0	10.2	14.55	9.69	6.18	3.43	1.45
3-6.c10702/1/4698	PREDICTED: LOW QUALIT	697487074	XP_00967463	230.08	102.37	462.24	327.84	366.54	510.11	340.01	349.61	445.54	122.18	78.73	208.91
3-6.c18147/1/2680.	PREDICTED: myosin-4 [Xen	847156920	XP_01282446	62.98	0	396.07	220.42	397.37	0.64	195.11	170.81	86.95	33.56	16.44	94.33
1-2.c50791/2/1166	--	--	--	10.73	2.85	0.03	5.03	1.26	0	1.59	6.09	6.73	0	0	1.24
2-3.c6840/1/2164	PREDICTED: pre-mRNA-sp	512835126	XP_00293762	2.03	6.81	1.77	5.45	0	2.75	1.26	1.85	0.96	16.2	24.35	16.17
1-2.c7729/8/1536	PREDICTED: ankyrin repeat	733897457	XP_01071290	17.26	2.54	19.84	23.29	19.37	40.07	20.81	9.41	13.71	294.26	208.21	101.7
3-6.c18441/1/3652	myosin, heavy chain 4, skelet	47575800	NP_00100124	1.64	0	4.78	3.22	4.34	0	2.35	3.95	2.53	0.36	0.07	1.39
1-2.c17124/1/1453	--	--	--	1.77	1.74	0.34	7.55	4.7	2.82	5.93	3.7	8.53	0.34	0.29	2.15
2-3.c46113/1/1953.	--	--	--	1.62	31.58	0.99	2.9	0.85	5	1.95	1.45	2.14	26.84	52.6	21.75
2-3.c17674/1/2363.	delta-1-pyrroline-5-carboxyla	156717284	NP_00109618	7.69	10.58	8.16	5.43	5.14	5.69	6.56	3.92	5.62	1.25	0.23	1.43
2-3.c56279/1/4020.	PREDICTED: SH3 domain-c	847102032	XP_01281293	1.7	0.47	7.28	1.4	3.41	2.43	3.21	1.31	5.54	0.32	0.71	0.27
2-3.c9907/1/2583.1	--	--	--	2.92	12.02	1.2	3.65	3.38	8.58	10.74	2.61	3.14	46.16	71.74	61.44
2-3.c55477/1/2355.	muscle creatine kinase [Xeno	147899942	NP_00108007	5.66	5.84	13.21	8.49	3.49	23.03	6.2	4.09	7.39	2.27	0	0.33
3-6.c9244/1/3049	C-cadherin [Xenopus laevis]	3150273	AAC16910.1	0.23	0	0.26	0.14	0.27	0	0	0	0	2.05	0.77	1.19
2-3.c26751/1/2371	--	--	--	6.13	24.26	1.92	3.6	5.05	9.44	3.6	4.06	3.25	46.08	24.31	51.32
2-3.c5673/1/2081	topoisomerase (DNA) II alph	148222806	NP_00108250	2.4	49.36	1.85	1.63	2.55	12.41	0.89	2.34	2.76	0.11	0.29	0.11

2-3.c39984/1/4356.	Y-box-binding protein 3 [Xer	62751871	NP_00101585	30.02	20.16	24.98	28.68	45.09	153.65	40.54	41.75	41.13	16.05	18.74	10.56
2-3.c19544/2/2433	PREDICTED: neogenin isofo	847142486	XP_0128216(	0	2.19	0	0	2.97	0	0.06	1.99	2.36	0.02	0	0
1-2.c3728/1/1533	ectonucleoside triphosphate d	147898505	NP_00108573	0.19	3.89	0.56	1.65	2.25	3.88	1.73	1	1.11	16.82	21.07	20.47
1-2.c8915/23/1694	PREDICTED: glutaryl-CoA (	847170161	XP_01280861	77.22	58.07	81.86	98.13	67.22	51.66	68.76	45.77	89.2	14.43	21.44	34.05
2-3.c59324/1/2606	PREDICTED: ankyrin repeat	301608278	XP_0029337(	2.12	0	0.72	0	0	0	0	1.54	1.58	0	0	0
1-2.c35951/2/1435	--	--	--	0	0	0.1	0	0.28	5.57	0.57	0	0.99	9.52	17.28	8.06
1-2.c28127/1/1346	PREDICTED: cortactin-bindi	847114286	XP_00491304	4.14	1.5	0.98	3.55	5.71	2.8	1.95	1.36	3.07	23.22	24.21	39.93
3-6.c18049/1/3361	PREDICTED: ralBP1-associa	395535050	XP_00376954	7.94	2.31	0.56	0.72	0	0	3.24	1.14	3.24	1.42	0	0
1-2.c22941/1/1350	--	--	--	3.33	2.35	1.14	3.91	1.21	1.14	1.21	2.36	1.47	24.14	30.17	22.14
1-2.c39356/1/4357.	keratin, type I cytoskeletal 19	485049501	NP_990340.2	566.47	127.21	496.9	712.09	332.9	140.69	660.95	632.65	620	376.84	216.37	144.56
2-3.c21231/1/2380	eukaryotic peptide chain relea	349585151	NP_00101580	0.19	0.84	0.16	0.09	0.47	0.78	2.81	2.79	2.57	0.38	0.62	0.29
1-2.c45258/1/1396	PREDICTED: dnaJ homolog	557275761	XP_00602105	0	0	1.3	0.77	1.71	1.77	0	0	0	3.25	0.94	5.68
1-2.c14386/1/1617	PREDICTED: F-actin-cappin	426227627	XP_00400791	27.67	92.74	39.66	30.23	49.63	92.34	56.52	90.78	116.7	23.05	21.98	48.71
2-3.c26373/2/2383	PREDICTED: paraplegin [Xe	301608280	XP_0029337(	45.82	11.28	15.17	9.31	14.34	5.64	13.66	23.73	27.71	8.05	6.07	8.26
2-3.c52177/1/6339.	myosin heavy chain, partial [	4249697	AAD13770.1	0.76	0	3.96	4.63	1.56	0	2.21	3.62	3.74	1.56	0	0
3-6.c16507/1/3416	PREDICTED: myosin-4 [Xer	847156920	XP_0128244(	3.27	0	3.63	3.67	2.73	0	2.86	7.3	4.68	1.11	1.19	0.86
2-3.c7669/1/2100.1	ATP synthase subunit gamma	226371866	ACO51558.1	710.34	356.86	570.96	508.06	599.01	403.84	485.82	510.57	535.83	195.38	174.63	205.01
1-2.c44451/1/1218	bromodomain-containing pro	148230679	NP_00108259	0	5.14	0	0	0	3.57	2.8	0.95	1.94	0	0	0
1-2.c43157/1/3783	PREDICTED: actin, alpha ca	803091489	XP_00401102	1.01	0.61	5.27	5.46	1.95	3.66	3.47	4.81	2.77	0.64	1	0.95
2-3.c4995/2/1739	bone morphogenetic protein 7	147900035	NP_00108086	2.22	11.21	3.71	3.99	0	0.25	1.93	1.18	0	17.72	12.08	17.39
1-2.c34207/1/6288.	PREDICTED: endoplasmic re	817323826	XP_01233262	8.25	16.48	2.44	15.6	1.83	17.24	11.33	4.51	5.27	110.13	113.73	61.05
1-2.c1327/147/1569	NADH dehydrogenase (ubiqu	148225797	NP_00108982	323.98	195.67	298.89	350.38	304.93	178.82	262.5	299.88	289.82	78	142.6	110.46
2-3.c9247/1/2402.1	PREDICTED: synaptotagmin	847099495	XP_01281226	4.95	3.33	13.86	2.69	13.43	1.67	13.7	7.76	2.59	0	1.61	0.82
1-2.c4228/6/1678	uncharacterized protein LOC	148235485	NP_00108971	4.83	0	5.18	3.16	3.17	1.37	3.07	5.36	2.54	0.54	0	0.74
2-3.c61115/1/2424.	hypothetical protein A306_0(	449277216	EMC85474.1	0.6	27.47	7.76	14.85	3.04	1.89	3.15	1.86	2.57	51.78	56.5	22.51
3-6.c4364/1/3186	alpha1 type II collagen [Cync	5360532	BAA82043.1	0.96	48.53	8.14	3	0.59	15.53	3.95	5.53	1.98	1	0	1.03
1-2.c12928/1/1320	--	--	--	0	0	0	0	0	0.68	0	0	0	10.85	15.43	0
3-6.c16843/1/3371	myosin, heavy chain 4, skelet	47575800	NP_00100124	2.03	0.11	4.27	2.78	2.24	0	1.54	3.92	3.15	0.43	0.6	0.82
1-2.c19321/1/5450.	larval type I keratin [Rana cat	134140868	ABO61147.1	31.3	4.7	9.45	12.04	10.87	0.86	9.89	9.57	5.56	1.78	0.8	1.54
2-3.c30940/1/1947	--	--	--	8.17	376.34	13.56	10.45	5.54	301.26	5.1	11.58	13.58	2.94	3.28	2.63
2-3.c17104/1/2122.	myosin heavy chain, partial [	4249701	AAD13772.1	3.05	0.51	3.69	2.36	3.11	0.1	2.86	2.89	2.86	0.37	0	0.11
2-3.c43395/2/2569	PREDICTED: eukaryotic elo	847162478	XP_01282552	2.6	6.48	5.42	20.39	6.74	5.57	3.63	7.44	8.48	0.43	0.46	3.94
1-2.c26129/1/1279	--	--	--	39.65	3.03	4.41	56.13	11.85	4.33	38.2	13.92	19.54	7.03	8.16	6.46
2-3.c33048/1/2158	ubiquitin-conjugating enzyme	1381181	AAB02656.1	0	0	0	1.57	0	0	0	0	0	7.22	11.52	0.01
2-3.c27487/1/2534	solute carrier family 38, mem	58332320	NP_00101105	7.05	4.1	7.06	3.96	4.58	5.3	3.07	4.46	5.08	0.91	0	1.62
2-3.c60711/1/4163	PREDICTED: myosin-4 [Xer	847156920	XP_0128244(	15.88	0.72	29.55	27.13	25.26	0	16.07	31.4	19.7	9.58	6.37	7.62
2-3.c13232/2/2569	vacuole membrane protein 1 [	56118368	NP_00100787	4.43	3.01	1.49	9.08	2.6	0	5.49	2.4	4.53	1.23	0	1.24
2-3.c55678/1/2219.	--	--	--	1.7	5.15	1.62	2.79	1.49	2.15	3.42	1.3	1.76	34.69	60.59	28.27
2-3.c36881/1/2214	--	--	--	42.66	0.62	4.64	19.45	13.44	0.29	3.22	8.7	13.69	0.3	1.95	1.3
1-2.c29389/1/1381	PREDICTED: protein kinase	699699565	XP_00990692	2.63	29.57	4.82	4.44	0	0	1.7	0	3.61	30.7	23.99	18.78
1-2.c26034/2/1285	Nucleophosmin [Rana catesb	226372338	ACO51794.1	119.51	308.82	68.82	66.11	58.59	98.62	62.66	64.97	61.55	17.7	23.36	26.39
1-2.c41445/1/1900.	--	--	--	4.08	4.67	0	1.95	2.59	1.97	1.46	0	0.33	12.45	20.66	9.58
1-2.c47447/2/1379	sodium channel subunit beta-	58332472	NP_00101131	0.5	0	3.44	1.15	2.02	0.74	2.4	0.88	1.75	0	0	0
2-3.c5429/1/1976	--	--	--	0	4.99	0	0.3	0	0	0	0	0	6.11	0	10.27
2-3.c9582/1/2637	glutamate dehydrogenase 1, r	58331978	NP_00101113	61.58	59.15	41.98	26.02	48.51	33.93	38.84	40.03	24.86	9.51	0	25.07
1-2.c34055/1/1363.	PREDICTED: ectonucleotide	847130990	XP_01281912	1.35	7.93	0.49	1.03	0.73	2.05	0.42	0.75	0.33	16.05	15.81	15.07
1-2.c44648/1/1545	--	--	--	1.13	3.75	0.16	1.21	0.51	2.1	0	0.35	0.51	19.06	21.23	3.71
2-3.c63371/4/2142	PREDICTED: fibronectin iso	768383139	XP_01158925	3.11	11.87	3.36	2.01	2.15	4.1	2.85	2.34	2.38	22.41	21.25	43.15
2-3.c4018/2/2373.1	PREDICTED: LOW QUALIT	847136111	XP_01282043	4.45	5.77	5.01	5.6	4.62	3.6	7.86	2.81	4.89	41	60.4	65.1
2-3.c61208/7/2271	myosin, heavy chain 4, skelet	47575800	NP_00100124	842.85	7.97	0.06	5.16	0.07	3.69	0	1790.41	1021.46	0	16.89	0
3-6.c8023/1/3947	phosphofructokinase, muscle	148224874	NP_00108692	1.88	0	0.85	0	1.98	0	0	0.9	1.41	0	0	0
1-2.c18811/1/1666	SPRY domain-containing SO	148224848	NP_00108883	0	0	9.35	0	6.39	4.55	9.41	6.44	8.18	2.82	3.56	0
1-2.c11248/1/1530.	--	--	--	0	47.71	2.02	7.82	1.58	10.62	7.3	1	0	99.74	242.7	74.12
1-2.c45163/1/949	voltage dependent calcium ch	11414934	BAB18559.1	96.27	134.76	105.86	34.36	61.03	181.94	44.62	39.2	41.5	6.88	20.09	13.91
1-2.c33078/1/1583	--	--	--	6.98	8.28	0	4.73	2.61	0	0	0.41	0.22	12	4.58	5.12
1-2.c40819/1/1483.	--	--	--	22	17.67	37.53	13.28	17.03	1.92	24.71	12.18	28.36	4.78	2.24	2.45
3-6.c13599/1/2904.	PREDICTED: endothelin-con	669276853	XP_00863068	10.71	30.42	9.66	18.45	4.41	12.34	16.84	11.39	12.58	129.16	158.19	133.19
1-2.c40990/1/1483	--	--	--	61.88	24.68	68.93	64.91	89.11	17.89	122.07	115.6	164.07	57.89	46.91	46.66

3-6.c10316/1/3600	myosin, heavy chain 4, skelet	47575800	NP_00100124	0.16	0.14	1.63	0.74	0.41	0.09	1.86	0.29	0.83	0	0	0.07
2-3.c13468/1/1885	--	--	--	4.83	0	4.37	0.11	21.43	148.33	11.93	4.54	5.51	3.74	1.65	0
3-6.c13706/1/3455	alpha 1 type I collagen [Rana	3242649	BAA29028.1	10.58	289.66	33.57	18.67	17.76	365.16	25.14	17.69	32.96	13.07	5.64	8.4
1-2.c30688/1/1582	PREDICTED: DBH-like mor	512842438	XP_0029363	4.94	2.41	2.25	4.14	3.73	2.43	3.17	3.78	1.29	31.84	39.41	33.12
1-2.c39644/1/1321	--	--	--	1.35	0	1.01	4.62	0	0	2.42	1.17	0	17.69	40.26	17.79
2-3.c12071/1/2342.	--	--	--	2.02	24.57	2.6	2.87	1.72	3.18	3	0.65	3.61	28.24	47.13	26.81
2-3.c26498/1/1913	transgelin-2 [Bufo gargarizan	635377487	AHZ89387.1	5.59	6.93	3.45	5.08	5.35	0.81	7.91	0.8	3.64	39.86	54.57	37.14
2-3.c59597/1/2079.	--	--	--	189.49	4.64	60.97	66.87	124.19	2.89	50.01	122.11	94.61	35	23.81	22.89
1-2.c26093/1/1332	inner-ear cytokeratin [Rana c	6980088	AAF34720.1	31.41	0.42	38.95	52.98	35.29	3.13	41.86	48.49	59.49	29.04	22.8	5.24
1-2.c49159/1/1781	--	--	--	60.61	46.94	45.13	58.13	50.25	58.34	50.96	40.21	59.68	18.66	11.31	23.08
3-6.c9894/1/3570	myosin heavy chain, partial [	4249697	AAD13770.1	1.16	0	2.02	1.79	1.12	0	1.41	1.47	1.26	0.11	0.07	0.29
2-3.c53927/1/1976.	sestrin-1 [Xenopus (Silurana)	194018650	NP_00112341	31.62	2.24	6.94	24.94	21.54	1.91	42.4	37.98	31.14	1.8	6.81	27.19
1-2.c16545/2/1344	--	--	--	1.11	11.81	0.56	1.15	0.26	3.54	0.79	0.99	0.69	10.78	19.99	15.69
2-3.c12698/1/2305.	--	--	--	0	6.9	0.37	0	0	1.21	0.36	0	0	3.89	13.23	5.55
2-3.c54921/1/2055	dehydrogenase/reductase SDI	166158027	NP_00110742	13.38	10.96	19.02	12.9	16.28	25.87	25.89	10.3	24.34	5.69	5.4	8.29
1-2.c2094/32/1682	PREDICTED: medium-chain	224058541	XP_00218952	86.36	50.41	75.45	46.85	101.03	48.3	73.31	79.05	87.74	21.41	36.57	33.12
3-6.c4577/2/2844.2	PREDICTED: talin-2 isoform	557294565	XP_00602972	21.42	11.08	24.22	18.31	17.08	18.33	16.98	16.69	18.89	5.05	1.04	6.59
1-2.c49223/1/1373	PREDICTED: nucleolar prot	556975779	XP_0059954	31.13	83.43	15.05	32.43	18.68	36.86	41.31	27.41	23.77	10.19	16.21	6.11
1-2.c12162/1/1582.	actin, alpha cardiac muscle 2	45361557	NP_989355.1	218.03	46.4	402.78	294.84	325.8	445.45	295.56	360.33	370.48	142.47	87.59	161.68
2-3.c37034/1/2383.	cadherin-13 precursor [Xenop	156718012	NP_00109654	3.26	5.02	5.1	2.39	5.29	5.68	3.69	4.19	6.89	1.56	0.19	1.39
2-3.c55977/1/2165.	MGC82982 protein [Xenopus	148236271	NP_00108598	2.12	13.46	1.1	2.65	1.68	4.26	1.69	1.54	2.16	23.6	32.2	19.83
2-3.c7346/1/2652.1	PREDICTED: solute carrier f	301613957	XP_00293647	0.81	0.36	0.45	0.2	0.35	0.48	0	0.36	0.34	4.65	3.34	10.12
2-3.c19149/10/2416	cirhin [Xenopus (Silurana) tr	113931428	NP_00103916	3.95	5.4	1.06	0.08	0.14	0.35	1.15	1.56	2.29	0.19	0.31	0
3-6.c18773/1/3218	PREDICTED: myosin-4 [Xe	847156920	XP_0128244	3.85	0	6.26	4.34	4.44	0	2.72	5.59	3.88	1.81	0.72	0.75
2-3.c10416/1/2538	--	--	--	9.93	9.61	14.94	9.75	12.38	16.08	11.95	9.13	12.68	4.28	1.49	4.48
1-2.c19686/1/1354	tyrosine 3-monooxygenase/tr	148224700	NP_0010864	0	0	0	0.19	0	0	0	0	0	8.87	11.77	0
2-3.c32729/1/2033	PREDICTED: arf-GAP with	641793969	XP_00817812	0	7.26	3.31	1.42	0.18	0.42	0	0	0	5.86	0	10.3
1-2.c20264/1/1615	rab-like protein 3 [Xenopus (	156717510	NP_00109629	5.87	4.38	2.13	0.79	3.44	0.3	0.31	2.44	2.68	0	0	0.19
2-3.c16061/1/2182	calsequestrin 1 (fast-twitch, s	148231736	NP_00108022	14.21	1.82	24.86	22.54	27.72	30.97	31.54	32.77	31.39	16.21	11.05	8.42
1-2.c32995/1/2922.	glyceraldehyde-3-phosphate	300679438	ADK27487.1	25267.62	1406.63	18300.74	27490.3	27987.01	3711.51	23168.97	21521.97	29375.34	9611.21	9644.47	9901.33
1-2.c26301/1/1840	PREDICTED: myosin-4 [Xe	847156920	XP_0128244	0	0	131.94	249.42	286.72	0	21.44	6.3	0	100.08	48.56	118.99
2-3.c15207/1/2631	kelch-like protein 40 [Xenop	147902760	NP_0010904	1.23	0	0	0.02	1.42	18.14	0	0	0	0	6.59	4.6
1-2.c26001/1/1351	--	--	--	8.54	16.83	5.31	9.18	5.72	7.04	12.61	5.16	5.58	72.93	89.66	80.36
2-3.c50353/1/1729	Unknown (protein for MGC:	80477042	AAI08785.1	16.87	33.27	11.3	8.41	10.77	47.31	14.7	13.61	12.87	2.98	1.45	7.2
2-3.c47933/1/2328	--	--	--	5.33	5.18	0.5	5.78	2.26	2.08	1.32	1.54	0.91	16.75	17.78	15.61
2-3.c61788/1/2164	calsequestrin 1 (fast-twitch, s	148231736	NP_00108022	3.39	0.93	8.5	5.54	8.29	8.43	4.58	7.45	6.54	1.86	1.07	1.85
2-3.c47381/1/2109	unnamed protein product [On	641929461	CDR19102.1	5.89	22.68	2.35	3.98	4.3	11.59	2.8	3.95	6.33	47.54	59.79	35.7
1-2.c4121/1/1332	--	--	--	1.74	3.34	0.07	1.75	0	0.56	0	0.69	0.5	8.99	11.48	11.62
1-2.c34076/1/8436.	PREDICTED: elongation fac	543731520	XP_0055068	3144	6000.08	2846.39	2178.68	2477.28	3895.67	2085.38	2107.33	2775	879.12	537.03	1221.47
2-3.c54334/1/2805	myozenin-3 [Xenopus (Silura	58332250	NP_00101127	1.97	17.3	12.24	2.52	4.85	102.55	4.05	2.09	2.58	0	0.17	1.28
2-3.c10302/1/2178.	--	--	--	4.98	47.09	2.44	4.23	3	7.86	3.7	2.57	7.18	55.19	81.82	36.39
2-3.c53355/1/1810	PREDICTED: calreticulin [A	557281027	XP_0060234	157.45	143.68	150.15	63.58	169.73	45.55	130.31	101.27	27.51	25.29	1.41	9.98
2-3.c47479/1/2159.	--	--	--	41.93	33.56	33.07	107.14	66.43	128.25	145.35	98.38	120.3	66.45	48.09	26.12
2-3.c52396/1/2150	PREDICTED: myosin-4 [Xe	847156920	XP_0128244	741.86	0.36	804.14	700.14	436.35	16.39	0	2.41	28.49	250.76	99.58	129.67
1-2.c17867/1/1078	PREDICTED: keratin, type I	602673215	XP_0074421	35.48	52.37	34.96	18.69	22.78	47.81	22.09	37.58	32.29	27.54	1.25	1.78
1-2.c2767/10/1803	Map2k3 protein [Xenopus la	50604142	AAH77760.1	31.8	52.27	56.55	26.4	47.67	58.1	38.06	37.57	27.8	18.86	9.44	9.5
1-2.c3323/1/1591.1	envelope polyprotein [Chloro	80550713	ABB52640.1	0.72	14.66	2.29	3.28	2.9	3.73	2.37	1.09	4.1	21.74	38.94	36.45
1-2.c5738/7/1372	--	--	--	3.14	3.49	7.48	5.98	1.21	2.38	8.76	6.04	1.07	0.11	0	0.72
2-3.c48283/1/2226	PREDICTED: acetyl-coenzy	301616096	XP_0029374	2.27	1.74	1.57	0	0.74	2.33	1.22	1.21	0.19	0	0	0
2-3.c53425/1/2653	--	--	--	0.41	1.93	0.8	2.28	0	4.05	1.88	0	1.39	18.01	9.52	12.29
2-3.c13656/1/2478	--	--	--	8.01	9.26	7.51	6.02	4.77	4.45	4.47	5.21	4.16	0.44	2.07	0.81
1-2.c37945/1/1560.	PREDICTED: protein Wnt-5	512839216	XP_0049142	0	9.28	0.52	1.89	0	0.53	0.33	0.26	0	6.38	8.4	12.65
2-3.c11611/4/2303.	PREDICTED: translational a	847168784	XP_0128081	18.68	11.76	13.89	16.95	16.81	11.74	11.72	12.62	18.71	5.22	2.61	4.36
1-2.c18499/1/1483	--	--	--	3.68	0.69	8.13	6.35	6.07	1.28	4.74	2.29	7.68	1.79	0	0.41
2-3.c43276/1/2280	ryanodine receptor alpha isof	538246	BAA04646.1	65.02	24.96	72.4	51.49	59.52	87.61	52.7	60.68	68.54	24.67	26.55	20.36
2-3.c39151/1/2622	PREDICTED: F-box only pro	847127821	XP_0128184	2.87	0.46	0.54	3.32	4.22	0.59	12.04	6.45	3.26	1.22	1.05	1.87
2-3.c41770/1/3030.	myosin heavy chain, partial [	4249697	AAD13770.1	908.12	28.17	1711.88	1774	1478.63	6.52	1371.08	1830.98	978.14	789.65	462.83	478.02
2-3.c41009/1/2497.	--	--	--	2.97	6.57	0.65	1.54	1.36	3.17	3.17	0.59	1.19	21.92	22.34	17.06

3-6.c15535/1/3720	RecName: Full=Sarcoplasmi	9789732	Q92105.1	2.15	0.42	2.98	1.96	2.39	1.58	2.41	2.68	3.75	0.4	0.69	1
2-3.c46043/1/2257	--	--	--	7.31	6.77	16.32	12.79	16.75	24.67	20.87	11.94	21.11	6.46	4.33	7.23
1-2.c29299/1/6568.	PREDICTED: elongation fac	573878959	XP_0066277	1621.64	3008.1	1403.47	1430.43	1268.08	1906.68	1259.14	1269.07	1405.83	576.93	361.26	613.53
1-2.c16743/1/1269	--	--	--	128.58	8.13	20.18	38.25	50.89	21.44	46.68	71.46	41.96	21.27	31.14	10.97
2-3.c21884/4/2601	alpha 1 type I collagen [Rana	3242649	BAA29028.1	6.63	219.04	20.6	16.89	15.98	149.44	18.73	12.79	24.19	9.53	6	4.48
2-3.c38731/2/2598.	uncharacterized protein LOC	148226767	NP_00108484	9.53	7.99	17.55	52.95	25.72	0	28.57	18.35	31.43	4.11	10.25	11.36
3-6.c13236/1/2824	potassium channel tetrameriz	148232084	NP_00108844	2.29	8.29	1.16	1.95	1.82	3.43	2.14	1.43	2.32	25.31	26.22	14.68
3-6.c18324/1/3082.	uncharacterized protein LOC	148225843	NP_00107989	0	0	0	0.97	0	0	0	0	0	4.52	6.36	1.98
2-3.c11480/9/2043	Methionine-R-sulfoxide redu	431892020	ELK02467.1	8.36	12.57	5.7	5.76	5.51	25.76	6.26	5.83	4.97	1.39	3.32	0
2-3.c61453/2/2316	PREDICTED: splicing factor	847091403	XP_01282544	0	0	8.08	5.4	0	3.85	2.44	3.77	7.63	0.24	1.85	0.73
2-3.c29278/1/2208	--	--	--	4.27	0.09	1.47	3.92	4.84	0.62	1.66	3.99	5.87	1.42	0.67	0.36
1-2.c44380/1/1354	--	--	--	19.21	22.67	27.18	43.4	18.29	21.86	21.27	25.09	27.28	8.39	8.09	7.64
1-2.c24461/1/1725	PREDICTED: integrin beta-l	512821557	XP_0029371	10.05	40.89	7.34	3.61	4.09	31.86	3.72	7.93	5.6	3.46	0.67	0
2-3.c39059/1/1897.	--	--	--	2.52	4.67	2.74	3.3	2.6	1.55	4.92	1.97	2.04	45.11	25.26	45.2
2-3.c48697/1/2159	--	--	--	0	2.11	0.49	0.96	0	1.15	0.84	0.33	0	7.84	8.84	6.47
3-6.c1957/1/4234	myosin, heavy chain 4, skelet	47575800	NP_00100124	1.34	0	2.69	2.3	1.85	0	2.36	1.93	1.72	0.67	0.24	0.36
2-3.c47652/1/2646	PREDICTED: tenascin isofo	512858291	XP_0049167	0.81	0	0	0.06	0	0.57	2.95	0.48	3.2	0.14	0	0.39
2-3.c49998/1/2377	--	--	--	6.44	0	3.94	1.73	5.09	1.51	1.6	5.09	4.62	0.18	2.3	0
1-2.c52188/11/1541	PREDICTED: keratin, type I	326919555	XP_00320604	3.66	0	183.05	159.86	32.78	0	139.8	106.95	194.07	53.54	30.63	80.22
3-6.c18240/1/3482	Ca2+-ATPase 1 [Rana clamit	12055497	CAC20853.1	3.2	0.81	3.66	2.37	3.16	1.74	3.68	4.01	4.26	1.15	1.17	1.02
1-2.c46271/1/1181	troponin T [Hyla chrysoscelis	69219560	AAZ04163.1	278.34	309.26	311.76	255.79	224.08	1243.14	495.55	341.61	255.4	139.65	183.41	120.79
3-6.c18369/1/2791.	PREDICTED: lethal(3)malig	512819552	XP_00491164	2.63	4.79	3.72	1.99	1.9	1.89	2.47	2.66	1.16	0	0.73	0.26
1-2.c24672/2/1442	--	--	--	29.98	64.67	18.34	24.59	0.98	7.94	9.07	10.92	11.62	118.25	91.48	108.46
2-3.c40223/1/2454.	--	--	--	0	0.44	0	0.14	0.13	2.79	0.11	0	0.26	4.82	7	4.26
3-6.c14118/1/3010.	hypothetical protein CAPTEI	443727653	ELU14322.1	1.59	8.37	2.31	2.59	1.01	2.69	1.51	1.01	3	20.97	45.59	28.84
1-2.c18264/2/1898	acetylcholine receptor subuni	148227704	NP_00108091	2.71	6.41	5.46	3.25	1.88	13.04	1.73	4.02	4.92	0.46	0.32	0.89
1-2.c22009/3/1530	PREDICTED: blood vessel e	847126715	XP_01281814	0.19	0	0	0	2.44	6.59	2.89	3.55	0	0	0	0
1-2.c38440/1/1475	acyl-CoA dehydrogenase fam	148228760	NP_00107991	9	10.39	6.58	9.12	5.24	4.47	8.44	5.64	8.65	2.19	1.81	1.18
2-3.c6564/21/2418	PREDICTED: beta-enolase [	637381761	XP_0081239	6.82	0	1.47	5.5	7.76	0	3.79	6.49	10.89	4.99	0	0.23
3-6.c3028/8/3407	PREDICTED: nuclear recept	512881896	XP_0029404	48.61	30.6	36.82	28.11	29.68	11.33	14.9	21.99	26.55	6.49	3	13.34
1-2.c8824/1/5102.2	PREDICTED: keratin, type I	543376583	XP_0055314	1072.71	244.14	837.78	1223.32	414.84	204.04	1042.51	1071.03	871.02	590.63	381.71	261.13
1-2.c25784/1/1258	PREDICTED: molybdenum	512844256	XP_0029429	5.23	6.7	0	7.3	0	2.08	2.61	3.5	3.14	42.83	56.33	20.29
1-2.c1620/94/1522	novel aldo-keto reductase fan	113931624	NP_0010392	16.29	7.5	45.98	45.53	44.25	45.43	45.08	50.89	46.59	20.88	23.74	11.4
1-2.c12891/6/1502	PREDICTED: prostaglandin	641764723	XP_0081674	35.33	36.56	23.37	19.82	18.7	24.06	15.76	21.52	20.82	6.45	7.73	5.69
1-2.c43690/1/1778	major facilitator superfamily	153792409	NP_0010933	0	0	2.43	1.08	0.66	0.05	3.5	0	2.38	0	0	0
2-3.c12301/1/2069	PREDICTED: nascent polyp	847099435	XP_0128122	17.07	42.38	15.09	9.74	10.57	24.51	12.2	10.66	14.45	4.7	4.5	3.19
2-3.c8140/1/2107	--	--	--	4.45	12.63	1.75	1.97	2.52	4.42	4.89	3.1	2.27	49.77	39.85	21.88
2-3.c11965/2/2367	PREDICTED: coagulation fa	554576773	XP_00588021	2.29	0	1.69	2.48	0.58	0	2.33	4.05	2.63	0.72	0	0.77
2-3.c8741/1/2049	ornithine decarboxylase 1 [X	148237898	NP_0010801	41.95	242.31	17.87	33.56	16.5	25.41	11.68	26.27	32.94	9.02	8.77	7.57
2-3.c20773/1/2231.	--	--	--	75.38	82.7	55.82	48.28	44.63	139.5	87.16	92.5	81.91	30	42.25	17.42
2-3.c55886/1/2096	--	--	--	5.61	24.51	3.03	4.53	6.92	15.16	4.52	3.31	3.84	35.31	35.93	49.73
2-3.c59443/1/2244	--	--	--	0.46	10.24	1.95	1.66	1.01	2.41	0	0.08	0	1.49	1.83	4.46
1-2.c48439/1/1637	glyceraldehyde-3-phosphate	300679438	ADK27487.1	40.96	12.53	40.09	32.11	24.91	21.59	32.29	39.64	24.32	8.24	8.91	17.41
2-3.c23550/6/2037	spermidine synthase [Xenopu	147902014	NP_0010848	9.86	41.4	4.18	7.07	5.26	7.38	14.46	7.82	7.29	3.62	2.77	2.56
1-2.c10838/3/1848	patatin-like phospholipase do	118404478	NP_0010726	2.32	0	0.76	4.22	0.96	0.17	1.71	0.51	1.01	0	0	0
1-2.c25860/1/1686	39S ribosomal protein L45, n	148235359	NP_0010795	0	0	0	0	3.19	4.18	2.34	2.51	2.75	0	0.77	0
2-3.c19323/1/2447	PREDICTED: phosphatidyli	344267740	XP_0034057	29.49	28.81	21.25	14.82	19	18.91	19.68	16.15	20.64	6.58	9.71	4.91
1-2.c6626/1/7057.1	muscle creatine kinase [Xeno	147899942	NP_0010800	70.39	6.61	93.26	71.86	91.93	49.31	44.17	87.45	76.41	31.85	26.86	17.36
1-2.c31015/2/1388	PREDICTED: multidrug resi	847126537	XP_0128180	11.91	10.61	6.95	11.55	10.61	2.87	17.96	5.58	8.27	96.66	147.34	69.99
1-2.c33917/1/1268	PREDICTED: sodium-coupl	612052686	XP_0075039	1.98	7.32	0.97	2.3	0	0.15	4.44	1.65	5.27	1.02	0.12	0
1-2.c14775/3/1310	--	--	--	22.53	0.26	11.25	20.41	9.84	46.51	34.6	24.61	48.18	16.98	12.26	9.18
2-3.c19947/1/2007.	--	--	--	8.65	56.34	47.63	24.27	18.63	231.1	40.34	17.35	23.51	7.39	4.58	11.75
1-2.c49544/1/3724.	PREDICTED: actin, alpha ca	726987387	XP_0103900	0	0.53	1.29	0	0.42	1.64	0.78	3.28	1.12	0	0	0
1-2.c12208/4/1387	PREDICTED: tensin-1 [Xen	847165849	XP_0128263	12.3	0.01	5.79	19.98	12.9	6.07	1.75	2.92	0	24.33	28.46	15.73
1-2.c8282/7/1408.1	PREDICTED: histone-lysine	635044876	XP_0079980	1.07	1.63	1.69	2.1	1	2.68	4.81	1.19	4.01	0	0.31	0
1-2.c10731/8/1845	PREDICTED: methylcroton	542168352	XP_00549274	26.09	27.39	16.93	20.35	17.29	20.92	15.62	14.05	15.83	3.71	5.85	5.51
1-2.c14679/5/1223	uncharacterized protein LOC	118404006	NP_0010721	0.98	2.22	1.25	1.28	0.55	0.82	0.78	0.98	0.59	10	9.61	25.39
2-3.c60402/1/2629	--	--	--	0	10.38	0	0	0	0	0	1.83	2.67	0.05	0	0

1-2.c44779/1/1324	paxillin [Xenopus (Silurana)	213983203	NP_0011355(	14.7	13.01	11.43	7.07	6.42	13.21	4.8	6.59	14.07	0.45	1.62	2.88
2-3.c52497/1/1916	PREDICTED: four-jointed bo	847123236	XP_01281754	0.48	3.28	0	0.13	0.45	0	0	0	0	6.15	3.65	0.21
3-6.c3020/5/4043.2	PREDICTED: aldehyde oxid	847167054	XP_0029377(	0.61	1.39	1.92	3.3	3.85	5.47	6.25	4.86	3.79	30.26	56.7	82.53
1-2.c44959/1/1535	PREDICTED: something abo	847167797	XP_01282698	19.04	38	4.84	7.82	5.52	8.49	9.59	16.42	7.93	2.54	1.78	5.09
1-2.c2132/14/1601	LOC100036924 protein [Xen	120538285	AAI29668.1	13.66	9.83	12.11	9	8.27	4.45	8.75	8.55	9.31	2.26	3.11	1.89
1-2.c5034/6/1449	PREDICTED: secernin-3 [Ch	530621714	XP_00530048	11.59	4.12	11.58	10.6	10.53	6.64	7.48	10.08	9.88	2.51	2.44	2.16
1-2.c23339/1/1762.	--	--	--	880.65	25.27	123.26	661.19	615.19	59.33	1285.95	839.43	895.96	363.03	860.06	72.86
1-2.c29344/2/1805	myosin heavy chain, partial [	4249701	AAD13772.1	0.97	0.6	2.82	0.85	1.39	0	2.8	0.54	1.11	0	0	0.12
2-3.c42150/1/2086.	--	--	--	0.15	1.76	0.64	12.71	0	0	5.69	0	2.13	45.11	31.36	22.94
2-3.c8550/11/2598	PREDICTED: pre-rRNA-pro	768932342	XP_01160682	5.04	25.69	5.56	3.75	5.64	17.62	5.02	4.05	3.58	1.35	0	1.5
1-2.c19990/2/1403.	--	--	--	0.38	0.97	0.59	2.16	1.64	0.46	0.35	1.32	0.98	27.77	17.01	38.93
1-2.c25545/1/1405	PREDICTED: palmdelphin [	512835762	XP_00293701	0	0	1.17	2.61	0	0	0.66	0.33	2.26	17.81	24.64	8.3
2-3.c23790/1/2240.	--	--	--	9.42	33.97	10.4	13.08	8.85	7.47	6.45	9.58	13.67	4.23	2.82	1.66
1-2.c17225/2/1260	PREDICTED: 2',5'-phosphod	558198612	XP_0061309(	0	9.42	4.5	2.98	0.23	3.91	3.81	0.76	4.02	24.33	49.83	27.82
1-2.c50160/1/1668	PREDICTED: poly [ADP-rib	847173218	XP_01280975	1.18	0.87	0.61	0.14	0.3	0.29	2.2	2.48	0.34	0	0	0.19
2-3.c39820/1/2575.	--	--	--	8.85	13.43	10.36	5.35	10.87	14.28	10.47	7.92	11.67	2.59	2.07	2.59
2-3.c49368/1/2153	PREDICTED: choline transp	530641746	XP_0053076(	3.53	1.29	4.01	0.92	1.5	1.05	2.51	1.75	2.98	0	0	0.69
1-2.c17349/1/2846	PREDICTED: actin, alpha sk	326920308	XP_00320641	4.74	0.31	4.47	2.35	3.29	2.31	4.04	4.1	4.2	0.8	0.79	1.33
2-3.c8480/1/2382	acidic leucine-rich nuclear ph	148228396	NP_00108315	15.61	14.48	15.7	15.95	17.4	6.63	7.89	13.99	11.73	2.1	1.96	6.79
2-3.c17471/2/2575	pentraxin 3, long precursor [	148226508	NP_00109115	1.54	0	13.46	2	6.9	0.49	0.49	1.76	3.05	0.34	0	0
2-3.c28056/1/2679	PREDICTED: collagen alpha	194224629	XP_00191509	7.7	61.43	19.63	7.45	2.47	1.67	4.94	12.38	3.47	0	0	0.71
1-2.c19329/1/1859	uncharacterized protein LOC	148232016	NP_00109117	32.08	0.88	0.3	7.41	14.96	0.83	17.98	21.28	11.02	5.25	13.06	1.31
3-6.c14525/1/7300	PREDICTED: alpha-actinin-	602667068	XP_0074391(	22.22	5.55	25.9	26.72	40.49	31.42	44.11	32.16	24.6	14.31	7.17	17.26
2-3.c33112/2/2350	PREDICTED: protein RCC2,	859758337	XP_0129039(	3.02	0.33	2.03	2.36	3	8.82	7.09	0	0	18.36	22.68	30.26
1-2.c28376/1/1287	--	--	--	14.67	41.56	12.48	11.53	11.49	20.97	9.94	8.08	18.89	4.35	1.68	3.55
3-6.c19183/1/3388	PREDICTED: titin-like [Pyth	602631486	XP_00742264	25.6	11.98	45.12	37.36	35.64	54.19	35.16	34.1	44.21	13.54	10.55	19.99
2-3.c23827/1/2044	--	--	--	7.14	0	3.46	5.27	2.05	0.3	2.06	6.07	8.18	0.63	2.86	0
2-3.c19571/1/2065.	--	--	--	12.8	0	2.87	1.9	5.52	2.61	4.38	4.57	2.13	0	0	1.16
2-3.c42569/1/2275.	pancreatic progenitor cell diff	604159143	AHV78314.1	566.32	1440	297.31	991.12	378.36	645.12	1001.15	375.81	554.51	5161.82	6545.91	5098.11
1-2.c20750/1/1839	PREDICTED: uncharacterize	847158215	XP_0128246(	2.02	17.12	1.88	4.14	1.16	3.81	1.37	0.61	2.04	16.95	25.23	9.95
1-2.c18249/1/1704	--	--	--	3.54	3.81	15.94	19.31	13.72	14.86	28.92	7.57	16.64	1.99	1.1	7.52
1-2.c36153/1/1633	--	--	--	12.93	5.7	35.49	16.08	16.96	23.38	19.88	35.86	34.11	16.4	4.86	12.03
2-3.c8820/1/2273.1	--	--	--	0.68	2.1	0.86	2.66	0.9	1.99	0.45	0.15	0.65	14.14	18.82	4.12
3-6.c18162/1/2809	PREDICTED: echinoderm m	847154147	XP_01282378	3.28	8.35	2.69	0	1.79	0.96	0.02	0	0	5.68	0	10.04
2-3.c10677/1/2224.	hypothetical protein CAPTEI	443682880	ELT87315.1	7.32	55.27	3.66	8.25	4.83	14.15	7.79	8.88	11.67	77.93	148.89	82.23
2-3.c45029/13/2081	uncharacterized protein LOC	147906566	NP_00109117	52.12	122.31	56.15	46.85	40.78	141.4	40.66	43.61	45.15	17.32	14.64	18.71
2-3.c16800/2/2127.	--	--	--	6.71	21.18	3.11	5.51	0	2.96	10.49	6.22	5.44	1.73	0	1.94
2-3.c17159/2/2231.	--	--	--	5.33	116.12	11.55	18.58	11.57	59.27	16.6	9.75	20.84	150.6	200.45	109.55
2-3.c21944/2/2127	PREDICTED: myosin-3 [Xe	847172863	XP_01280963	3.91	14.34	7.2	5.31	4.33	69.53	7.5	3.23	5.5	2.16	0.97	0.86
3-6.c15051/1/3387	FXYD domain containing ior	147902838	NP_0010864(	6.17	8.47	7.16	4.29	3.21	3.72	6.24	4.17	2.43	2.23	0.05	1.28
2-3.c14687/1/1981	--	--	--	8.84	7.38	11	11.26	8.09	2.95	7.29	6.99	11.97	1.96	3.68	2.5
3-6.c18506/1/2622	collagen alpha-1(III) chain pr	218847780	NP_00113637	20.68	160.38	29.25	18.47	55.36	446.27	40.05	14.06	28.68	16.73	2.85	10.58
1-2.c19745/1/1622	Sperm-associated antigen 1,	676584923	KFO74026.1	0	0	0.61	0	0	0	2.09	0	2.08	0	0	0
2-3.c37303/2/2551	transmembrane protein 182 p	118404178	NP_0010724(	0	0.84	0.52	0.69	0.41	7.95	1.46	1.76	0	0	0	0
1-2.c11344/1/1759	succinyl-CoA ligase [ADP/G	71896153	NP_00102558	71.7	46.79	113.94	120.92	109.38	73.55	111.65	107.45	134.32	30.97	92.15	30.21
2-3.c23763/1/2466	PREDICTED: uncharacterize	301617369	XP_00293811	0.03	0	0	0	0.45	0.55	1.34	1.01	0	0	0	0
1-2.c4450/2/1272	--	--	--	0	0	0	0	0	0	0	2.35	3.62	0	0	0
1-2.c52047/1/1687	PREDICTED: serine--tRNA	602647281	XP_0074300(	12.85	30.99	7.4	5	6.82	44.15	23.28	11.05	11.85	5.05	4.68	5.28
2-3.c12858/2/2655	PREDICTED: myosin-7B [X	512871888	XP_00293293	23.5	41.1	18.29	21.44	16.42	127.54	19.04	16.99	13.12	7.17	4.97	6.07
1-2.c34179/1/1524	PREDICTED: periplakin [Xe	512869040	XP_0029410(	11.4	7.96	12.72	1.86	11.21	11.87	0.02	0	0.92	10.15	27.04	8.77
2-3.c26482/2/2492	--	--	--	2.06	5.06	4.56	0	1.75	9.62	1.27	3.48	4.67	0	0	1.54
2-3.c53385/1/2333.	PREDICTED: uncharacterize	512848095	XP_00491541	2.27	25.45	2.05	3.71	1.35	6.4	2.8	1.64	3.48	28.39	44.49	23.79
2-3.c60333/2/2139	--	--	--	5.97	0	0	0	1.51	0	3.67	0	5.8	0	0.01	0.03
2-3.c60667/1/2143	tRNA-dihydrouridine(47) syn	147903417	NP_0010796(	13.2	22.48	5.05	5.85	0	0	8.85	8.29	7.24	6.36	1.36	0
2-3.c25964/1/2469.	--	--	--	0	0	0	0	0	1.58	0	0	0	6.57	18.84	4.68
1-2.c33438/1/1568	--	--	--	9.98	15.88	5.68	6.7	6.11	4.97	11.6	0.05	4.85	60.14	61.93	37.65
2-3.c8551/1/1879	SEC62 homolog [Xenopus la	148234873	NP_00108684	0	0	0	0.4	0	1.47	0	0	0	6.1	10.09	0
1-2.c28315/1/1394.	--	--	--	77.63	31.62	141.17	72.72	65.05	6.06	145.47	83.21	105.51	57.69	7.15	41.92

1-2.c51096/9/1414	ureidopropionase, beta [Xeno	148234835	NP_0010875(	4.91	10.58	10.21	16.49	9.79	16.6	15.43	12.61	11.07	2.73	3.37	5.06
1-2.c2443/2/1468.1	c-fos induced growth factor ('	189217641	NP_0011212(	9.6	9.05	6.01	4.05	4.89	4.27	4.01	5.74	5.63	71.97	52.77	57.47
1-2.c8815/2/1731	phosphoglycerate kinase 1 [X	62858027	NP_00101654	14.04	0	7.61	17.04	7.79	0.8	11.38	9.44	10.77	0	2.82	6.1
2-3.c54343/1/2347	PREDICTED: dual specificit	634861343	XP_00794372	0	0	0	1.14	0	0.11	0.26	0	0	5.04	4.3	1.99
2-3.c62822/4/2266	uncharacterized protein LOC	147905311	NP_0010906(	0	0	0	0.95	0	0	0	1.12	1.32	0	0	0
3-6.c2423/20/3512	PREDICTED: very low-dens	301609344	XP_00293422	0	0	0.45	0	0.6	0.43	0	0.69	0.81	0	0	0
1-2.c24478/1/1602.	--	--	--	4.41	31.4	2.72	2.58	1.95	7.67	0	1.19	1.07	32.65	76.36	20.35
3-6.c10674/1/2961.	--	--	--	2.17	2.62	3.01	1.34	1.34	3.6	1.18	2.91	4.24	0.19	0.33	0
2-3.c33579/1/2157	PREDICTED: succinate dehy	847123585	XP_01281758	5.92	2.79	4.24	10.45	10.07	4.5	8.12	6.7	10.43	1.87	1.92	3.61
2-3.c42427/1/2811	--	--	--	3.41	253.48	8.7	3.62	4.8	170.29	2.12	4.39	6.32	2.73	0.23	0.39
1-2.c14456/3/1825	--	--	--	0.57	0.99	1.19	0	2.12	0	2.63	0	0	14.21	6.74	14.68
2-3.c6906/1/2785	PREDICTED: metastasis-ass	847154043	XP_01282374	0.95	6.32	0.7	0.24	0.11	0	0.09	0.58	1.2	0	0	0
2-3.c40136/1/1855	molybdate-anion transporter j	189217675	NP_0011212(	0	5.84	0	0	0	0	1.59	1.46	0	0	0	0
2-3.c48796/1/2002	PREDICTED: myosin heavy	657587209	XP_00829788	13.8	0.33	28.57	18.66	23.18	0.39	11.13	26.86	23.65	8.95	6.84	7.69
1-2.c48353/1/1474	--	--	--	4.94	22.63	4.16	4.7	3.19	5.55	3.37	3.02	3.01	38.98	26.33	38.94
1-2.c31225/1/1129	--	--	--	0	4.62	0	0	0	5.28	0	5.06	0	26.64	32.74	20.24
3-6.c4220/1/4081	PREDICTED: myosin-3 [Xe1	847172863	XP_01280963	1.41	6.69	3.85	1.7	2	60.53	1.45	2.78	1.2	0.13	0.12	0.7
2-3.c16217/1/2133	PREDICTED: synaptopodin	512828724	XP_0029373(	2.06	2.11	0	2.47	1.67	2.97	4.65	3.91	5.89	2.5	0.3	0.66
2-3.c2636/32/2205	PREDICTED: 60 kDa heat sl	768339285	XP_0115744(	9.28	44.32	3.99	7.73	21.33	55.42	18.98	7.18	6.68	3.24	0.86	2.46
3-6.c13051/2/3182	forkhead box protein K2 [Xe1	213982945	NP_00113563	29.45	29.62	15.47	23.32	12.75	7.63	9.16	24.55	27.16	7.15	5.5	6.13
2-3.c2446/2/2299	WD repeat and SOCS box-co	45361625	NP_989387.1	1.99	4.09	1.21	2.18	2	1.11	1.6	2.66	2.98	0.62	0.31	0.22
1-2.c10819/13/1575	PREDICTED: keratin 19, typ	847167891	XP_01282703	34.77	11.15	25.25	31.35	13.84	8.37	25.34	27.62	20.03	13.36	5.81	7.61
2-3.c49481/1/2244	PREDICTED: creatine kinas	126344225	XP_00136422	1.68	0	1.81	1.24	1.35	0.65	2.77	0.5	1.79	0.2	0.17	0.1
1-2.c14263/1/1364	reverse transcriptase, partial [	347227	AAA49027.1	1.19	3.69	0	0.23	0.73	2.42	1.32	0.41	0	23.75	23.37	4.93
2-3.c12469/1/6062	--	--	--	0	27.04	0.17	11.59	0	0	0	11.16	12.59	0	0	0.17
3-6.c5644/1/5515.1	glycogen synthase kinase-3 b	148226891	NP_00108375	0.06	0	0	0	0	0	0.96	1.54	0.25	0	0	0
2-3.c2857/1/2285	--	--	--	13.95	1.51	10.92	7.41	2.86	2.02	4.37	5.63	1.59	0.8	0.46	0.71
1-2.c33457/1/4870.	PREDICTED: LOW QUALI	637377851	XP_00812323	83.31	11.48	104.78	81.48	114.71	60.45	81.4	103.94	112.73	37.66	29.81	38.67
1-2.c1978/16/1820	beta-hexosaminidase subunit	187608414	NP_00112045	15.61	2.88	2.4	7.09	8.04	5.52	11.14	6.86	5.68	70.09	59.62	94.17
2-3.c5996/1/2143	glutamate oxaloacetate transa	333805525	BAK26556.1	141.92	43.9	104.77	122.98	48.52	38.63	105.83	40.9	67.25	19.69	23.01	38.99
1-2.c44455/1/1718	PREDICTED: bcl-2-like prot	847177164	XP_00492107	21.16	16.77	26.24	26.4	26.77	7.78	17	16.03	28.74	3.45	8.12	10.12
3-6.c11324/1/2740	PREDICTED: nascent polyp	847099448	XP_01281224	7.98	4.76	6.24	3.21	2.5	14.75	3.04	4.41	3.99	1	1.09	0.91
2-3.c9494/1/2611	PREDICTED: ATP-depender	795120083	XP_01183217	0.58	13.6	0.6	1.43	0.73	3.31	1.26	0.38	1.49	12.82	24.38	9.7
1-2.c18995/1/1256	--	--	--	106.39	4.37	5.16	37.01	22.12	2.09	22.74	36.37	32.64	6.27	26.75	4.09
1-2.c36758/1/6899.	nuclease-sensitive element-bi	147903761	NP_0010793(	971.79	4882.91	965.89	1207.16	1008.67	2057.8	961.47	1058.23	1428.72	428.73	196.88	726.75
1-2.c49364/1/1192	PREDICTED: eukaryotic init	821477144	XP_01240194	0	1.37	0	0	0	2.69	0	0	0	3.26	5.15	2.87
2-3.c6297/2/2080	--	--	--	0.3	15.19	2.98	1.03	2.54	8.33	2.2	1.44	1.16	24.75	8.33	22.59
3-6.c14882/1/4946.	PREDICTED: calpain-7 [An	327274957	XP_00322224	0.07	0.97	0.55	2.1	0	2.35	0	0	0	2.37	0.22	4.63
2-3.c62194/3/2047	PREDICTED: actin-related p	663283393	XP_00849925	0	9.41	2.74	0	0	0	1.32	3.59	0.52	0	0	0
3-6.c6424/1/3069	PREDICTED: ectonucleotide	847135208	XP_01282011	0.07	24.95	0.43	0.17	0.72	8.35	1.89	0.1	0.53	13.08	4.64	18.21
3-6.c17950/7/3451	myosin, heavy chain 4, skelet	47575800	NP_00100124	0.45	0	1.35	0.87	0.22	0	0.9	0	0.76	0	0	0
3-6.c17076/1/2738.	--	--	--	0	6.96	0	23.01	22.35	4.43	20.58	27.68	36.16	0	27.67	0
1-2.c15720/1/1380	UBA domain containing 1 [X	148235257	NP_00108937	9.8	4.02	6.59	7.31	7.84	1.45	10.3	5.57	17.95	1.04	2.46	5.04
1-2.c30288/1/1350	--	--	--	65.08	11.93	41.39	67.3	55.41	27.23	70.46	46.93	87.08	32.02	26.75	22.62
1-2.c2105/17/1262	transmembrane protein 182 p	118404178	NP_0010724(	12.42	42.12	29.45	15.61	15.22	157.62	13.03	10.32	12.25	4.14	3.8	2.21
1-2.c48651/1/5600.	muscle creatine kinase [Xeno	147899942	NP_00108007	25.55	2.06	13.28	8.83	9.33	6.28	23.28	14.12	11.34	4.57	3.81	3.53
1-2.c9303/1/1473	--	--	--	85.12	0.69	24.72	28.88	54.72	0.98	22.51	55.01	31.47	11.79	5.23	7.26
2-3.c4505/1/2004	PREDICTED: glycogen [star	327281085	XP_00322528	11.53	0.73	10.7	20.23	18.7	1.95	8.21	10.75	10.15	1.6	1.5	5.3
3-6.c18246/1/3720.	hypothetical protein, conserv	557144680	CDI75148.1	3.83	4.51	6.66	6.89	7.52	17.39	9.51	6.89	9.05	2.28	2.97	3.69
2-3.c46635/1/2202.	PREDICTED: solute carrier f	301613957	XP_00293647	0.22	1.46	0.57	1.01	1.43	0.43	0.08	0.25	0.33	7.11	7.52	8.97
3-6.c4669/1/4636.1	myosin heavy chain, partial [	4249701	AAD13772.1	1.59	0	2.03	1.1	1.64	0	1.4	1.38	2.1	0.15	0.27	0.38
2-3.c55773/1/1954.	--	--	--	12.12	5.44	10.65	37.18	14.83	16.3	25.57	9.6	23.01	6.51	5.31	5.98
3-6.c14815/1/3319	PREDICTED: myosin-bindin	557258509	XP_00601494	1.86	14.28	7.14	6.41	2.35	279.36	2.87	8.87	3.79	0	0	0.94
2-3.c59609/1/1968	PREDICTED: CD99 antigen-	301623490	XP_00294104	0	0	0	0	0	0	1.59	0	2	0	0	0
1-2.c29489/3/1675	PREDICTED: thrombospond	847131343	XP_01281919	0.86	4.4	0.99	2.35	1.5	11.22	5	0.05	0.72	30.44	21.84	12.41
1-2.c38076/1/3768.	muscle creatine kinase [Xeno	147899942	NP_00108007	45.33	4.37	60.72	42.23	49.04	28.31	38.57	49.28	49.9	11.98	14.42	24.96
2-3.c41744/1/2519.	--	--	--	0.45	13.77	0.89	1.15	1.31	2.42	0.61	0.41	1.99	15.35	22.82	8.43
2-3.c49103/1/2458.	--	--	--	28.65	69.05	12.23	14.33	14.41	14.74	19.86	17.39	14.78	182.13	227.67	102.39

3-6.c15880/1/5573	myosin, heavy chain 2, skelet	55742222	NP_00100680	3.75	0.12	7.55	6.06	5.14	0.1	3.76	7.24	5.01	2.19	1.12	1.63
1-2.c18950/2/1384	PREDICTED: zinc finger prc	847139787	XP_01282101	10.4	17.42	4.98	5.12	2.87	5.54	3.12	4.59	5.91	0.61	1.33	0.48
1-2.c42887/6/1598	PREDICTED: myosin-4 [Xer	847156920	XP_01282446	1794.78	31.28	1102.64	1087.89	1077.07	6.76	984.09	3503.95	2795.11	477.95	342.84	488
2-3.c37066/1/2329	cellular retinoic acid-binding	25453404	NP_058940.1	5.8	11.28	2.85	5.8	7.37	3.1	8.02	3.28	5.91	57.08	64.92	41.1
2-3.c59054/1/2659	rcc1 protein [Xenopus (Silura	171846900	AAI61455.1	0	0.92	0.35	0	0	0.61	1.39	0	1.04	0	0	0
2-3.c38881/1/2380	PREDICTED: protocadherin	512816337	XP_00294091	0.84	32.14	0.9	0.62	1.1	8.57	3.74	2.02	2.74	0.63	0	0.76
1-2.c19267/1/1414	trimeric intracellular cation cl	148237167	NP_00107953	16.16	3.54	25.87	17.42	17.91	2.82	3.28	12.19	12.13	0	0	3.87
3-6.c16660/1/3265	--	--	--	7.36	6.32	8.63	4.01	7.32	2.33	5.1	6.55	5.08	1.56	0.38	3.15
1-2.c32987/1/1430	--	--	--	12.11	4.09	29.57	33.32	22.51	7.19	37.51	39.66	25.13	8.92	1.41	25.52
2-3.c39805/1/2408	PREDICTED: sodium/potass	620963907	XP_00766720	1.47	2.56	0	3.18	0	0.85	3.01	0	0.71	12.83	11.15	17.06
2-3.c37339/1/2537	PREDICTED: nuclear recepto	512881896	XP_00294049	49.62	24.11	33.17	27.82	16.48	2.77	9.23	14.91	28.77	2.65	3.93	6
1-2.c46225/1/1329	--	--	--	1.27	0.92	0.37	1.41	1.26	1.09	1.81	0.98	0.14	14.37	20.27	13.67
1-2.c49914/1/1729.	PREDICTED: proline-rich tra	597733803	XP_00722921	20.06	4.3	20.89	8.44	8.77	1.94	2.06	8.8	10.01	0.26	0.61	0.22
2-3.c14430/1/1901	--	--	--	4.33	0.28	7.43	4.52	4.04	0.84	4.04	6.27	5.32	1.38	1.5	1.11
2-3.c27183/1/2627	--	--	--	1.26	20.47	0.82	2.24	1.69	3.67	1.55	1.53	2.56	19.27	33.02	14.01
2-3.c53510/1/2445	ubiquitin carboxyl-terminal h	183986733	NP_00111695	0	0	0	0	0.4	0	0	0	0	2.95	0	2.65
1-2.c12240/1/1300	PREDICTED: alpha-aminoac	699658970	XP_00989810	2.25	3.64	15.26	0	5.23	29.09	16.04	13.72	17.22	6.17	10.08	0.24
2-3.c1948/2/2285	PREDICTED: adenylyl cycla	301606235	XP_00293272	14.03	7.63	14.21	9.29	11.66	15.67	6.82	11.36	10.43	3.55	3.88	2.44
3-6.c10531/1/3124	PREDICTED: talin-2 isoform	847147373	XP_01282232	15.91	8.75	21.19	11.46	17.31	14	15.22	14.06	13.78	2.46	2.45	6.52
1-2.c22567/1/1576	PREDICTED: transmembran	847095050	XP_01281107	45.03	58.63	16.59	16.96	25.08	19.24	25.28	39.57	30.38	11.9	11.29	13.99
1-2.c49257/1/1239	--	--	--	81.15	159.03	37.66	82.6	44.81	104.78	140.12	63.05	91.75	792.13	1144.82	648.76
2-3.c10834/1/2448.	PREDICTED: putative nucle	512821917	XP_00491191	1.09	11.88	0.75	4.34	0.76	2.18	1.91	0	0.86	5.85	21.11	17
2-3.c41391/1/2449	--	--	--	2.31	11.6	14.04	5.76	5.79	63.87	8.21	4.44	5.59	1.45	0.58	2.94
3-6.c4001/1/3035.1	Transcription factor BTF3 4,	676423020	KFO64485.1	277.95	606.5	264.78	225.57	210.92	640.68	325.39	237.84	239.34	105.85	113.22	113.91
1-2.c12548/1/1636	PREDICTED: nebulin isofor	847166512	XP_01282661	14.72	13.03	71.5	65.65	14.33	0	22.93	54.13	46.95	26.98	0	14.27
1-2.c24824/1/1485.	--	--	--	16.41	15.03	3.12	6.46	2.76	31.81	9.94	7.09	7.82	101.86	105.18	50.32
2-3.c43754/2/2456	PREDICTED: LOW QUALIT	543714928	XP_00549874	42.22	9.04	54.29	33.2	28.98	43.2	32.79	34.6	59.52	11.2	12.4	26.24
1-2.c24232/1/1263	PREDICTED: protein transpo	847087082	XP_01281625	0	3.9	0.53	1	0	2.32	0	0	0	9.34	16.32	0
1-2.c3100/2/1546	rCG19955, isoform CRA_a [	149032951	EDL87792.1	2.2	0	0	0	0	5.57	1.31	0	0.56	15.71	9.07	5.62
2-3.c52659/1/2150	PREDICTED: bifunctional 3'	831298598	XP_01268117	0.12	0	0	0	0	0	1.32	1.52	0	0	0	0
3-6.c5040/1/3392	PREDICTED: band 4.1-like p	847168906	XP_01280820	21.78	6.2	20.96	14.38	13.59	4.15	11.24	15.39	13.76	4.51	6.45	5.18
2-3.c26996/1/2078	vitamin K epoxide reductase	55742523	NP_00100692	15.83	245.94	16.62	11.86	14.64	72.65	24.88	8.93	16.76	137.97	159.93	172.33
3-6.c15406/1/2790.	glucosidase, alpha; neutral AI	148232724	NP_00109123	0	0	0	0	0	0.69	1.8	0	1.28	0	0	0
3-6.c8920/1/3848	PREDICTED: protein CDV3	847134819	XP_01281997	12.98	11.16	8.06	3.03	11.17	18.37	6.25	4.37	4.21	0	0	4.15
1-2.c13673/7/1307	PREDICTED: actin, cytoplas	795093282	XP_01181546	10.42	3.35	14.36	12.51	17.97	7.62	17.94	10.84	9.42	2.58	1.55	6.38
1-2.c25074/1/1720.	--	--	--	3.96	3.2	3.72	5.29	2.39	2.05	3.17	3.03	4.11	36.87	45.95	44.8
2-3.c20986/6/2085	PREDICTED: thymopoietin i	847109041	XP_01281420	1.26	0.79	0	0.45	0	0	0	0	0	0	3.85	3.34
2-3.c2306/20/2235	6-4 photolyase [Xenopus laev	147906624	NP_00108142	10.53	24.12	13.89	4.71	6.36	6.23	8.02	6.69	9.85	3.86	2.31	1.73
2-3.c17398/1/2387.	basigin [Xenopus (Silurana) t	89268702	CAJ83018.1	459.77	112.91	170.79	368.9	387.88	94.65	109.55	211.18	287.74	86.25	24.7	119.24
1-2.c8938/2/1432	inositol hexakisphosphate kin	62859747	NP_00101595	0.02	8.67	0	0	0.49	0	0	0	0	5.01	0	7.76
1-2.c27349/1/1732	TPA_inf: tropoelastin 2a [Xe	296040422	DAA12508.1	2.18	0	1.04	0.93	1.17	0	2.45	3.15	1.02	0	0.8	0
2-3.c15881/1/2060.	--	--	--	14.83	61.72	9.21	16.28	6.6	14.42	10.85	8.43	10.81	102.9	141.85	57.15
2-3.c62964/1/2466	cyclin I [Xenopus laevis]>gi|	148228521	NP_00108357	30.8	49.67	33.89	25.43	23.3	14.39	12.07	21.29	19.8	3.88	0.79	14.21
1-2.c9731/1/1808	major facilitator superfamily	153792409	NP_00109335	2.25	8.96	1.92	0.14	0	2.74	0	1.28	1.75	0	0	0
1-2.c40900/1/1278	PREDICTED: kelch-like prot	301610740	XP_00293489	74.27	16.86	42.41	36.49	65.33	56.42	61.85	45.56	88.52	12.93	63.97	9
2-3.c48925/1/2195	PREDICTED: protein transpo	301609492	XP_00293429	0	3.1	2.11	2.21	2.8	1.03	5.13	1.44	2.52	1.06	0	0.41
3-6.c15016/1/3195	PREDICTED: myosin-4 [Xer	847156920	XP_01282446	0.7	0.07	2.52	1.47	1.93	0	1.2	2.36	1.8	0.16	0.21	0.56
2-3.c26922/1/2290.	--	--	--	9.48	34.27	4.92	6.12	8.42	13.06	10.5	7.66	11.71	102.92	99.2	90.13
1-2.c8269/1/1291	--	--	--	2.73	1.68	4.32	3.88	2.32	3.81	4.89	3.98	7.14	1.03	0.86	0.92
1-2.c12187/4/1338	PREDICTED: nucleolar GTP	395539819	XP_00377186	82.91	86.59	51.9	24.74	35.07	50.82	39.62	41.86	56.43	17.87	6.88	26.58
2-3.c53140/1/2342.	--	--	--	12.02	109.24	37.72	23.05	11.79	21.67	18.46	11.59	35.55	8.08	7.05	3.15
1-2.c22200/3/1727	umps-prov protein, partial [X	56789550	AAH88513.1	16.25	44.68	5.91	13.23	9.58	10.18	12.75	10.25	12.16	4.74	2.82	3.79
2-3.c23394/3/2655	PREDICTED: protein Shroor	847100721	XP_01281266	2.76	7.69	1.14	1.59	2.56	2.82	0.87	2.1	2.52	15.08	12.51	32.4
1-2.c44317/1/1236	MGC86492 protein [Xenopus	147904348	NP_00108781	8.54	51.81	5.79	11.59	6.05	21.77	10.23	7.78	13.23	99.16	102.94	94.19
1-2.c16583/2/1367	ribosomal protein L3-like [Xe	148223643	NP_00108803	2.7	3.48	2.41	1.44	2.08	3.35	2.38	6.88	4.68	0.36	2.09	0.26
1-2.c34127/1/1458	Uroplakin-3a, partial [Colum	449273315	EMC82838.1	21.47	10.08	18.57	9.48	18.41	4.68	10.44	10.68	3.96	1.58	0.69	4.29
1-2.c13419/1/1831	PREDICTED: glypican-5-lik	847167197	XP_01282679	0	0	0	0	0	1.07	0	0	0	7.72	15.64	0
1-2.c21967/8/1387	serine palmitoyltransferase sr	62857651	NP_00101721	2.36	0.59	0	4.89	3.78	0	2.37	2.05	3.81	27.1	30.52	36.7

2-3.c54284/1/2147	PREDICTED: myb-binding p	512820645	XP_0049117(	3.34	10.67	1.96	1.34	2.78	6.93	5.58	1.98	1.82	0.47	0.56	0.52
1-2.c17594/1/2370.	Myozenin-1-like [Xenopus la	148237556	NP_00107945	377.96	35.87	546.63	489.68	358.36	319.02	769.99	423.07	943.12	319.4	343.48	249.13
1-2.c39919/1/1290	PREDICTED: LOW QUALIT	637377851	XP_0081232:	24.19	6.75	46.34	43.31	35.18	14.01	29.72	27.8	54.12	4.85	4.37	29.13
2-3.c23423/1/2433	PREDICTED: unconventiona	847091238	XP_0128252(	14.21	4.71	10.72	0	11.74	10.71	6.18	8.23	8.84	2.01	5.59	0.9
3-6.c7796/1/4300	uncharacterized protein LOC	148225160	NP_00108922	2.31	64.82	14.4	3.71	11.92	62.96	2.34	6.45	2.49	0	0	0.74
2-3.c12775/1/2529	C4 protein [Xenopus laevis]	213626287	AAI70422.1	4.32	0.47	2.61	3.38	1.3	0.29	1.95	2.41	3.28	0.51	0.01	0.77
1-2.c4205/2/1195.1	dual adapter for phosphotyros	62751355	NP_00101572	66.17	25.34	40.21	48.11	54.34	15.64	62.03	51.8	41.39	445.21	502.94	466.44
2-3.c10474/1/2554	extracellular sulfatase Sulf-2	54020831	NP_00100566	0.97	0	0	0	1.12	0	0.06	0.89	1.89	0	0	0
2-3.c59938/1/2047	--	--	--	2.49	1.83	3.23	2.79	2.74	1.41	4.73	2.23	1.77	0.5	0	0.82
3-6.c17332/2/3093	--	--	--	0	0	0	0	1.24	0.07	0	0	0	3.81	0	8.89
3-6.c18499/1/6403.:	--	--	--	12.13	175.49	7.55	23.32	11.05	26.2	13.44	11.81	19.61	217.38	358.59	120.13
1-2.c2558/10/1433	PREDICTED: telethonin [Xe	301622194	XP_00294041	22.57	0.94	24.46	2.9	0.32	0.66	0	2.39	4.88	0	0	0
2-3.c7633/1/2691	--	--	--	1.05	1.27	6.61	13.73	3.92	4.93	8	4.15	13.66	1.37	2.83	3.1
3-6.c2973/3/3877	PREDICTED: proline-rich nu	224048441	XP_00219808	12.16	7.31	6.43	6.45	7.5	1.27	3.48	8.56	8.32	1.44	0.07	4.48
1-2.c35071/2/1356	PREDICTED: serine/threonin	617644733	XP_00753235	5.32	16.75	1.93	2.03	2.92	3.5	0.79	1.46	0	8.51	14.73	15.93
1-2.c26807/2/1700	alpha 1 type I collagen [Rana	3242649	BAA29028.1	6.07	198.31	18.08	15.32	12.04	126.92	15.28	10.35	22.55	8.25	4.52	4.13
1-2.c6874/4/1447	PREDICTED: LOW QUALIT	821485492	XP_01240543	4.38	204.6	24.12	17.6	0	121.38	8.23	12.42	12.43	0	2.51	6.87
1-2.c45824/1/1230.	--	--	--	1.73	13.18	3.33	3.73	4.63	4.37	3.66	2.4	3.68	31.27	45.72	62.63
1-2.c4837/1/1479.1	PREDICTED: tropomyosin a	831547267	XP_01272688	346.98	8.66	563.83	488.96	399.62	9.28	467.48	440.67	356.01	208.09	204.9	146.94
1-2.c17851/1/6739.:	eukaryotic translation elongat	62859813	NP_00101669	1201.02	2614.09	1029.88	806.76	734.99	1029.2	728.04	822.23	870.92	261.12	74.27	635.72
1-2.c5375/11/1442	PREDICTED: LOW QUALIT	803125841	XP_00401312	16.77	1.97	13.65	18.36	24.12	13.14	13.04	14.96	14.34	4.58	1.66	6.99
1-2.c25795/1/4418.:	PREDICTED: actin, alpha ca	803091489	XP_00401102	132.81	100.59	431.26	530.5	83.65	130.62	489.7	457.15	223	182.66	137.77	173.58
2-3.c12430/4/2306	protein tweety homolog 2 [X(	45361675	NP_989411.1	23.35	66.17	6.43	11.4	12.95	20.56	12.41	9.38	10.45	84.42	107.32	99.28
2-3.c14018/1/2514	PREDICTED: LOW QUALIT	512836507	XP_0029347(	0.53	0.48	0.89	0	0	0	1.19	1.09	0	0	0	0
2-3.c20256/1/2003.	--	--	--	10.53	20.54	1.39	6.09	9.51	11.36	15.12	5.28	5.2	83.56	169.81	74.69
2-3.c2893/1/2597.1	basic leucine zipper and W2 (	147906588	NP_00108029	106.8	60.92	43.42	30.61	53.05	50.32	65.09	32.02	34.15	0.12	0	42.79
2-3.c20935/1/2615	LOC496301 protein, partial [	76780301	AAI06222.1	4.46	4.8	2.47	2.46	2.36	1	2.56	2.26	2.7	0.46	0.43	0.53
1-2.c22508/1/1458	tubulin, beta, 2 [Danio rerio]	834400287	NP_00103248	119.75	0.63	67.92	34.03	23.59	71.85	30.74	47.34	30.07	13.59	1.5	25.05
1-2.c44857/1/1364	--	--	--	0.67	5.37	0.77	2.13	0	14.08	1.27	1.27	0.85	34.62	61.72	15.08
2-3.c6620/4/2296	glutamine-dependent NAD(+	187608643	NP_0011204(	4.46	2.07	3.86	5.24	3.37	2.15	2.7	3.48	3.84	0.31	0.72	1.04
2-3.c4478/15/2180	PREDICTED: retinoic acid re	301614423	XP_00293668	2.33	14.43	0	2.53	3.74	3.89	2.29	0.37	0.63	11.23	11.91	16.02
3-6.c9142/1/3104.2	--	--	--	2.16	7.27	0	2.3	1.86	4.02	1.6	2.89	2.15	27.54	30.94	25.83
1-2.c40310/1/1404	chloride intracellular channel	148224931	NP_00108268	0.31	0	3.46	2.41	0.12	0	2.6	2.19	2.38	0	0	0.39
3-6.c18822/1/2734	PREDICTED: myosin-4 [Xen	847156920	XP_0128244(	0.25	0	1.59	0.96	0.72	0	1.62	0.83	0.69	0.05	0	0.13
2-3.c60184/1/2170	--	--	--	0	1.32	1.6	4.04	0	0	5.07	0	2.33	0	0	0
2-3.c46375/1/2538.	--	--	--	0	1.49	0.62	1.88	0.28	0.41	2.43	0.27	1.51	0	0	0
2-3.c2883/1/2560	eukaryotic translation initiatio	148237458	NP_0010800(	3.74	1.24	6.85	4.21	4.77	2.08	4.1	5.84	5.02	0.64	0.44	2.93
2-3.c13541/1/2607	LOC398149 protein [Xenopu	60649448	AAH91650.1	4.99	0.85	2.02	1.96	3.91	1.08	3.95	2.67	1.02	17.92	26.29	31.92
1-2.c24789/1/1540	PREDICTED: ATP synthase	564257576	XP_00626773	216.06	98.52	123.37	169.3	234.29	187.25	194.69	192.1	185.34	66.09	64.53	110.71
3-6.c4153/1/2908.1	PREDICTED: ankyrin repeat	591350883	XP_00706989	4.4	0.29	0.18	3.91	2.88	1.52	3.39	3.68	2.09	0.08	0	0.74
2-3.c15691/1/2295.	--	--	--	0.47	1.7	0.38	1.09	0.19	0.71	0.62	0.64	1.26	5.86	16.72	12.07
2-3.c46123/1/2302.	--	--	--	0.36	7.8	0.23	0.36	0.51	2.6	0.37	0.12	0.75	7.2	11.67	7.5
1-2.c30329/1/1717	--	--	--	4.37	12.07	2.35	5.44	2.08	3.69	2.71	2.68	2.97	25.81	34.19	30.3
2-3.c62437/2/2508	PREDICTED: arrestin domai	847088847	XP_0128212(	1.74	1.09	1.2	0	1	2.55	1.29	1.04	1.06	0	0.23	0
1-2.c32859/2/1541	PREDICTED: periostin isofo	560975750	XP_00621017	31.57	0.29	39.32	49.54	14.3	9.51	15.08	47.08	46.94	16.76	6.2	5.42
1-2.c39980/1/9175.:	reticulon-2 [Xenopus (Siluran	166795979	NP_00110773	32.16	112.86	62.71	26.41	33.17	141.62	47.35	43.12	39.04	16.14	24.22	11.47
2-3.c63476/5/2036	PREDICTED: adenylyl cycla	704180660	XP_0101352(	5.43	0	1.48	0	1.76	0	8.42	2.07	2.41	0	0.8	0
1-2.c27312/9/1373	--	--	--	73.61	50.02	153.41	108.78	5.75	23.09	123.68	86.73	6.64	3.48	0	5.76
1-2.c44631/1/1192	PREDICTED: neurofilament	301617086	XP_00293797	19.6	27.88	37.83	26.89	26.41	124.53	59.98	26.98	37.48	15.53	21.7	11.41
1-2.c13843/2/2305	ras homolog family member ,	148225697	NP_00107972	26.2	3.27	0.56	18.64	0.82	0.53	0	17.58	0.26	62.49	58.15	67.29
2-3.c4811/1/2177	ring finger protein 20, E3 ubi	163914497	NP_00110633	0	0	0	0	0.33	0	0	0	0	0	2.69	2.85
2-3.c12262/2/2062	mannosidase, alpha, class 2A	148226300	NP_0010855(	2.67	0.16	1.96	0	2.59	1.28	0	0	0	2.9	1.51	0.91
2-3.c2130/8/2346	PREDICTED: 5-beta-cholest:	512849887	XP_00491563	0.69	1.14	0.28	1.89	0	0.7	0.68	0.82	2.17	0	0	0.12
1-2.c14950/1/4597.	PREDICTED: keratin, type I	697020374	XP_00957168	5.75	1.47	9.61	0	0.02	0	4.66	7.83	0	0	0	0
2-3.c54604/1/2355	--	--	--	1.56	34.69	1.7	4.16	1.84	7.15	3.25	2.71	4.36	34.45	58.94	24.02
3-6.c14765/1/4012	PREDICTED: reticulon-4 iso	847127949	XP_01281853	37.05	6.62	32.54	20.57	25.45	3.55	14.43	21.34	17.37	8.38	3.94	9.39
2-3.c37841/4/2152	ectonucleoside triphosphate d	55742043	NP_00100679	1.15	4.12	0	3.72	1.06	3.68	0.52	1.21	1.38	12.48	14.46	14.59
1-2.c45701/1/1902	twinfilin-2 [Xenopus (Siluran	194018662	NP_00112341	129.11	7.39	37.28	26.75	74.11	18.31	125.08	56.63	39.17	10.36	34.37	13.88

1-2.c11033/1/6975.	muscle creatine kinase [Xeno	147899942	NP_00108007	16.79	1.48	20.42	21.5	22.36	11.4	19.33	18.2	26.34	8.31	6.2	7.96
3-6.c5889/1/6501	profilin-2 [Xenopus (Silurana	62859423	NP_00101609	12.95	21.49	16.05	10.04	7.35	7.18	7.88	11.21	4.11	2.08	0.5	3.13
2-3.c20900/1/2411	acetyl-CoA acetyltransferase	148234082	NP_00108602	20.62	1.12	1.45	2.38	1.93	0.81	13.78	12.85	0	0	0.31	0.41
1-2.c5521/1/1688	tyrosinase-related protein 1 p	148223219	NP_00108049	1.37	4.01	1.63	0.75	1.26	1.01	4.75	1.09	1.3	0.23	0	0
2-3.c27312/1/2649	PREDICTED: cationic aminc	847153149	XP_01282343	75.68	104.07	19.15	24.56	42.66	28.87	97.59	64.05	48.49	39.1	25.52	26.03
1-2.c10949/6/1831	PREDICTED: NAD-depende	530626779	XP_00530291	27.46	32.21	19.22	24.5	21.8	20.68	12.9	22.62	25.33	5.53	5.09	12.01
2-3.c47018/1/2447.	--	--	--	1.13	0.19	0.31	0	0.23	0	0	0.06	0	11.17	0	8.03
2-3.c44606/5/2291	PREDICTED: forkhead box p	543358692	XP_00552324	0	10.14	2.8	3	2.5	2.15	1.13	4.23	5.36	0.73	1.2	0
3-6.c7510/1/7734.2	--	--	--	0.67	1.27	0.58	1.17	0.98	0.77	0.56	0.49	0.81	8.13	8.22	16.45
2-3.c27825/1/2538	PREDICTED: nebulin isofor	847166493	XP_0128266(	5	7.55	11.43	2.16	1.28	8.62	1.58	2.86	3.5	0.68	0.55	0.44
1-2.c22295/1/1424.	--	--	--	0.75	6.45	4.37	2.72	2.15	21.08	1.59	2.66	0.67	0	0	0
2-3.c52319/1/2076	myozenin-3 [Xenopus (Silura	58332250	NP_00101127	0	0	0	0	0	0	1.9	1.08	0	0	0	0
3-6.c18408/1/3097	PREDICTED: collagen alpha	591360730	XP_00705528	1.01	30.33	5.97	0.85	0.59	3.69	0.44	2.28	3.54	0.05	0.47	0
1-2.c48433/1/1581	PREDICTED: nucleolar com	847148668	XP_0128225(	5.23	14.93	3.55	3.67	3.92	7.23	6.43	3.32	3.57	0.35	1.32	0.9
2-3.c33718/1/2168.	--	--	--	0.78	0	0	0.53	0	0	0	0	0	13.43	12.55	0
1-2.c25899/1/1338.	--	--	--	1.72	64.14	1.15	3.03	1.54	5.8	0.9	1.84	3.91	28.03	47.97	27.02
1-2.c39070/1/3637.	fructose bisphosphate aldolas	98979411	ABF60004.1	47.52	0.29	28.07	42.13	32.29	4.29	33.51	35.11	40.9	17.3	13.01	11.61
1-2.c22972/1/1524	--	--	--	7.81	6.56	20.41	2.36	30.13	2.9	18.07	6.09	21.61	4.48	6.23	4.6
1-2.c49176/1/1504	--	--	--	3.99	19.3	1.74	5.48	3.15	9.12	2.56	0	0	10.99	46.48	92.42
2-3.c14492/1/2202	MLK-like mitogen-activated	525507075	NP_00126758	10.87	8.58	10.38	7.5	9.53	17.14	7.72	9.21	7.96	4.27	3.58	1.25
3-6.c17376/1/2728	myosin, heavy chain 4, skelet	47575800	NP_00100124	4.23	0.18	7.93	7.41	5.65	0.04	4.33	8.49	6.44	2.45	2.36	1.92
2-3.c13861/1/1947	--	--	--	8.25	87.35	4.84	6.75	2.01	15.87	4.75	3.74	6.13	50.13	58.89	32.23
1-2.c48758/1/1648	--	--	--	402.57	12.5	65.68	127.14	224.53	5.52	319.03	336.05	260	148.72	111.89	144.06
1-2.c7884/1/2480.2	Homogentisate 1,2-dioxygena	677297600	KFP80028.1	0	7.23	9.58	0.56	1.37	4.22	11.32	0.66	4.33	0.08	0	0
2-3.c40208/1/2528	--	--	--	8.07	4.67	2.18	1.9	2.55	1.16	8.98	7	2	2.68	0.88	0.2
3-6.c13212/1/3747.	Ca2+-ATPase 1 [Rana clamit	12055497	CAC20853.1	3.01	0.61	1.41	1.48	3.06	1.43	2.09	2.19	1.99	0	0.37	0.55
3-6.c15080/1/2723	--	--	--	0.2	2.42	0.12	0.99	1.51	0.49	0.98	0.28	0.2	5.88	9.49	5.36
2-3.c56093/1/1951	WD repeat and SOCS box-co	45361625	NP_989387.1	6.02	28.35	9.79	5.93	8.47	17.83	10.01	9.57	9.59	3.4	3.06	3.32
2-3.c21000/4/2668	--	--	--	35.62	55.29	35.52	21.76	25.44	50.12	33.03	31.83	39.54	7.3	23.18	14.79
1-2.c41371/1/1189	PREDICTED: keratin, type II	512822642	XP_00293578	165.35	156.94	428.8	289.94	0	93.17	554.28	74.85	258.8	15.55	12.74	22.54
1-2.c46748/2/1769	--	--	--	0.55	6.94	3.25	0	0	29.26	16.71	0	5.66	0	0	0
1-2.c14135/6/1641	BCL2/adenovirus E1B 19 kD	62751435	NP_00101585	9.04	2.92	6.29	6.69	11.32	0.41	5.37	9.56	5.4	0	0	4.86
1-2.c5895/1/1586	--	--	--	4.23	30.58	3.91	4.04	2.94	12.28	4.93	3.62	6.41	59.54	41.68	42.73
3-6.c16222/2/2954.	PREDICTED: putative pre-m	591366635	XP_00705817	25.07	51.18	11.86	10.43	8.12	12.73	9.53	12.17	5.67	3.44	4.33	1.67
1-2.c36886/1/1315	PREDICTED: junctophilin-2	557262533	XP_00601678	23.23	15.5	57.39	31.81	0.01	36.1	15.12	31.45	24.94	11.75	6.62	8.85
1-2.c19359/2/1645	carboxymethylenebutenolidas	148233964	NP_0010882(	0	9.14	1.64	4.11	1.03	1.71	7.03	3.44	4.54	0	2.36	1.11
2-3.c23983/1/2290.	--	--	--	0	0	2.09	0	0.18	0	0	0	0	9.5	10.38	0.19
3-6.c17185/4/2926	myosin heavy chain, partial [	4249701	AAD13772.1	0.11	0	1.65	1.25	0.43	0	2.08	0.46	0.78	0.06	0.13	0.06
3-6.c15150/1/3265	PREDICTED: ceruloplasmin	301615846	XP_00293737	1.01	12.91	1.25	0.39	0.94	2.81	0.68	1.18	1	0.2	0.04	0
1-2.c40819/1/1483.	--	--	--	46.94	16.63	59.65	34.66	41.92	4.75	87.07	24.82	78.17	32.27	24.47	7.85
3-6.c2662/14/3358	nuclear factor, erythroid 2-lik	147900041	NP_00108001	136.79	26.92	45.06	76.95	103.53	13.79	117.07	101.9	87.14	34.32	29.41	66.24
2-3.c34853/1/2318.	--	--	--	33.1	4.67	49.13	85.24	88.88	9.75	96.42	67.23	87.15	36.16	5.38	53.34
2-3.c1588/34/2495	PREDICTED: cyclin-L2 isof	847146354	XP_01282212	16.43	27.69	3.91	7.3	3.64	7.5	8.53	8.13	11.98	0.08	5.77	4.49
1-2.c36883/1/9679.	MGC84445 protein [Xenopus	51703940	AAH81189.1	9.08	10.42	9.38	2.44	2.91	0	8.23	2.85	1.65	0	0	0
2-3.c42563/1/2383	cellular retinoic acid-binding	25453404	NP_058940.1	3.32	134.33	6.07	1.62	7.01	22.15	42.07	14.77	33.1	3.97	0	26.96
2-3.c29868/1/2078.	--	--	--	23.24	2.66	28.27	31.03	26.78	4.4	44.96	21.52	40.55	27.43	6.14	6.34
3-6.c7728/1/3338	PREDICTED: myosin-3 [Xer	847172863	XP_01280963	1.95	7.04	4.12	0.64	1.45	74.6	1.57	1.87	1.68	0.96	0	0
1-2.c2245/23/1297	LOC548400 protein, partial [	59861981	AAH90369.1	318.1	37.5	44.81	61.74	100.3	18.6	86.09	78.23	63.44	501.54	694.05	754.53
2-3.c10262/2/2337	transcription factor Dp-1 [Xe	148222844	NP_00108805	0.46	0	0	0.55	0	1.91	0.39	1.46	0.44	0	0	0
2-3.c54510/1/2290	4-aminobutyrate aminotransf	189230071	NP_00112152	2.93	0	1.2	2.68	5.49	0.31	5.99	6.5	5.06	0.87	1.9	2.49
3-6.c7775/1/3739	PREDICTED: bcl-2-like prot	847177164	XP_00492107	30.19	10.66	19.82	33.62	19.52	9.42	15.18	15.89	20.52	5.61	2.87	11.63
2-3.c4393/1/2332	--	--	--	2.55	53.44	3.32	2.94	2.92	16.72	2.2	2.86	4.44	32.71	14.43	43.65
2-3.c7650/1/2206	--	--	--	0	9.9	3.29	0.03	4.33	3.73	0	0	0	2.93	2.72	0
2-3.c9525/1/2034	PREDICTED: myotubularin-	847089882	XP_01282302	2.42	3.37	0.92	3.35	0.78	3.22	6.05	1.47	2.05	34.75	30.81	26.97
2-3.c7319/1/2423	cGMP-dependent 3',5'-cyclic	118404742	NP_0010726(	0	0	0	0.96	0	0	1.45	3.09	0.2	0	0	0
2-3.c35193/1/2278.	--	--	--	6.2	0.5	0.06	31.38	7.84	1.3	14.46	9.97	13.29	7.23	1.25	2.64
1-2.c14512/1/1673	PREDICTED: cytochrome P4	512845316	XP_00293364	3	0	0	1.59	1.69	1.04	2.44	2.62	1.49	0	0.67	0
3-6.c3167/1/3572	PREDICTED: myosin-bindin	564233250	XP_00626099	0.38	0.18	31.44	0	0	0	14.96	3.58	0	0	0	0

1-2.c52466/1/1252	LOC397919 protein [Xenopu	213625217	AAI70091.1	112.74	553.45	89.37	94.3	112.85	258.94	121.92	80.87	112.1	50	37.74	44.53
1-2.c17928/1/1743	--	--	--	0	0.18	0	0	0	0.22	0	0	0	0.49	6.35	3.1
2-3.c42443/1/2247.	--	--	--	2.07	16.25	0.75	1.7	1.77	4.81	1.48	1.25	1.85	17.28	29.52	11.58
3-6.c16028/1/2660	breast cancer anti-estrogen re	147906605	NP_00108519	0.21	0.62	0.9	0	0	1.36	3.57	2.38	1.21	0	0.91	0.54
3-6.c18431/1/2995	PREDICTED: cardiomyopath	512810079	XP_00491044	27.63	4.01	37.87	37.76	43.17	5.53	32.45	45.35	34.35	13.79	7.54	25.44
1-2.c18863/1/1308	--	--	--	5.9	7.89	1.1	2.14	2.26	5.84	4.45	1.67	2.67	31.87	43.53	20.61
1-2.c1919/31/1776	zinc finger, AN1-type domai	148234811	NP_00108039	9.26	28.66	4.48	7	7.01	4.07	0	0	5.46	22.81	11.98	23.78
1-2.c2539/4/1292	--	--	--	39.65	147.73	57.18	31.3	9.21	13.75	19.61	39.06	55.5	25.74	5.68	10.36
1-2.c47206/12/1506	PREDICTED: calsequestrin-	821480671	XP_01240345	277.22	31.52	281.17	293.29	428.37	381.12	460.75	482.01	424.73	229.91	219.26	175.15
2-3.c42752/2/1879	PREDICTED: LOW QUALI	695159509	XP_00950788	1.48	0	0.79	0	1.09	2.63	0	0	0	0	3.05	4.86
1-2.c32947/4/1746	--	--	--	9.03	7.67	4.1	1.47	0.48	1.22	4.8	4.74	8.26	2.41	1.76	0.66
2-3.c46796/1/2073.	--	--	--	4.43	20.31	2.81	2.91	2.38	3.15	4.47	1.82	2.15	29.42	32.9	30.85
1-2.c52372/7/1426	tripartite motif containing 54	147905260	NP_00108509	487.5	34.02	188.75	250.28	334.06	44.67	260.29	298.99	300.56	93	239.23	77.1
1-2.c51455/108/162	glycogenin 1 [Xenopus laevis	148232896	NP_00109023	355.71	25.72	133.37	117.95	154.52	46.01	76.27	152.6	119.96	22.51	98	44.47
2-3.c23256/1/2186.	PREDICTED: myb-related tr	847129062	XP_01281880	0.49	15.1	0.27	0.35	0.31	1.33	0	0	0.29	6.04	24.36	8.26
3-6.c18604/1/3111	myosin heavy chain, partial [	4249701	AAD13772.1	2.09	0	3.93	3.98	3.38	0	2.35	4.28	3.12	0.78	1.18	0.88
3-6.c15710/1/4011	PREDICTED: DNA damage-	847123732	XP_00293768	13.56	13.48	10.68	10.8	16.96	10.89	9.99	12.84	9.3	3.53	1.14	7.56
2-3.c27849/1/2268	ras homolog family member	148225697	NP_00107972	1.51	1.32	0.15	1.38	0.15	0.27	0.33	0.8	0	4.68	9.13	6.5
1-2.c13042/1/1557	PREDICTED: coiled-coil dor	641766364	XP_00816802	0	0	0	0	0	1.86	0	0	0	4.39	0.54	2.58
1-2.c25020/2/1328	Sprouty2 [Xenopus (Silurana	52352549	AAU43764.1	1.18	0	0	0	0.34	7.03	0	0	0	6.74	4.98	0
2-3.c3410/1/2051.2	--	--	--	24.36	14.57	17.18	7.81	13.77	5.32	20.77	19.33	24.28	8.71	3	4.72
2-3.c8815/1/2097	--	--	--	2.61	71.66	2.57	10.45	1.65	0	2.4	4.36	6.33	43.26	50.25	32.92
2-3.c39831/1/2085	PREDICTED: kelch-like prot	847095166	XP_01281109	0.35	5.27	0.66	1.88	1.98	2.06	0	0.86	0	5.49	7.26	6.39
2-3.c55108/1/2351	--	--	--	1.41	10.73	6.01	4.33	2.26	43.34	2.68	3.27	7.35	0	2.73	0.77
1-2.c3245/1/1571	PREDICTED: serine/threonin	597759458	XP_00724140	0	0	0	0	0.02	0	7.03	2.69	0	0	0	0
1-2.c10915/1/10690	PREDICTED: LOW QUALI	558146048	XP_00611959	2.76	0.39	0	0.02	0	0.36	0	2.1	3.01	0	0	0.06
2-3.c15520/6/2195	PREDICTED: nuclear recept	694856246	XP_00946969	1.34	0.92	0.46	0.82	0	2.33	0.12	0.29	0.13	3.18	7.1	3.87
3-6.c4976/1/3855	PREDICTED: nebulin isofor	847166506	XP_01282661	0	0	3.31	0.4	0	1.54	1.96	1.48	0.27	0	0	0.29
3-6.c12875/10/3715	RecName: Full=Sarcoplasmi	9789732	Q92105.1	3.07	0.11	3.76	5.46	3.76	2.6	2.78	4.4	3.41	1.83	0.38	1.09
2-3.c42381/1/2121.	Hypothetical protein EH28_0	808880242	KKF28166.1	1.76	32.32	1.31	5.41	1.09	4.02	1.5	1.87	3.26	37.03	30.47	18.26
1-2.c29265/1/1451	--	--	--	3.42	22.25	1.35	3.39	1.02	2.63	2.15	1.76	2.49	23.18	32.05	18.39
2-3.c43926/1/2676.	Chain A, Catalytic And Struc	21730859	1LUW	111.01	96.51	109.69	99.19	106.09	136.27	122	105.68	111.78	51.86	46.69	35.28
2-3.c52694/1/2565	PREDICTED: E3 ubiquitin-p	847087434	XP_01281732	0	3.77	0.09	0	0.55	0	0	0	0	1.29	0.62	2.13
2-3.c55630/1/2547	PREDICTED: pre-mRNA-pr	847162749	XP_01282561	0	0	0	0	0.49	0.61	0	1.02	0.86	0	0	0
2-3.c16746/1/2131	--	--	--	1.43	14.9	1.28	1.03	0.76	7.84	2.04	1.36	2.38	15.68	15.9	28.11
2-3.c39688/1/2478	cat eye syndrome chromosom	58331875	NP_00101108	1.26	0.99	0.37	0.35	0.59	0.1	1.68	0.85	0.6	0.14	0	0
3-6.c14023/1/3707	PREDICTED: calcium chann	557294828	XP_00602984	47	8.47	33.13	49.02	79.58	4.48	46.91	42.62	55.84	14.09	13.86	33.24
3-6.c3245/2/3337	SH3 domain-binding protein	349501002	NP_989213.2	0.7	5.56	1.76	0.18	3.56	0	1.22	0.65	0	0	0	0
1-2.c25677/1/1638	Unknown (protein for IMAG	197246590	AAI68801.1	0	0	1.39	0.16	0	0	0	0	0	0	4.6	3.77
1-2.c30420/1/1364	--	--	--	1.42	13.91	0	4.53	0.09	5.61	6.59	4.16	1.11	0.99	0.67	0
1-2.c13095/1/1686	uncharacterized protein LOC	351542170	NP_00108826	25.61	21.71	32.73	25.36	29.29	21.52	26.19	31.1	29.29	13.85	12.01	10.06
2-3.c6458/1/2667.1	--	--	--	12.01	1.52	13.07	8.79	9.87	5.44	1.87	0.84	7.03	20.34	24.79	42.76
3-6.c15817/1/4121	PREDICTED: nidogen-2 [Tri	471361161	XP_00437114	1.84	46.44	10.93	4.37	4.24	21.47	0.83	2.59	3.28	0.54	0.11	0.73
2-3.c1868/17/2266	PREDICTED: adenosine kin	874462866	XP_01295145	5.42	7.07	3.86	4.38	5.49	1.99	4.72	3.35	3.46	1.08	0	1.51
1-2.c48187/2/1389	LOC398623 protein, partial [	32450751	AAH54264.1	33.75	35.72	24.61	25.21	18.16	12.38	27.38	29.66	25.69	0	8.5	21.89
1-2.c7315/1/1789	--	--	--	9.75	33.18	15.39	15.11	16.74	45.17	24.1	18.03	13.3	5.11	0.57	13.65
2-3.c40509/1/2336	--	--	--	0	0	1.1	0	2.14	0	1.73	0.93	0	0	0	0
1-2.c2118/33/1787	aldose reductase [Xenopus (S	58331962	NP_00101113	14.95	92.05	34.51	37.43	36.7	72.24	35.62	32.47	30.15	14.3	6.71	18.32
1-2.c45934/1/1325.	--	--	--	18.25	14.84	22.26	12.63	15.55	3.11	16.66	8.26	15.33	2.75	4.79	0.96
2-3.c48791/1/2648.	PREDICTED: metastasis-ass	512877662	XP_00491926	1.13	10.66	1.18	0.56	4.73	0	0	1.08	2.12	0	0	0
1-2.c2462/2/1377	--	--	--	2.41	89.88	1.62	6.46	4.48	45.11	8.84	4.12	4.6	70.19	49.9	163.61
1-2.c36725/1/1635	SH3 and cysteine-rich domai	55926168	NP_00100750	14.05	33.16	23.8	9.58	15.34	51.64	18.02	15.78	13.07	5.84	0.25	9.87
3-6.c4989/1/3762	sarcoplasmic/endoplasmic ret	383511148	AFH40438.1	10.58	0	7.82	8.02	17.21	10.07	9.48	11.75	9.57	3.03	1.49	7.22
2-3.c47277/1/2145	--	--	--	0	0	0	4.82	0.17	0	0	10.79	6.8	0	0	0.16
2-3.c11775/5/2064	55 kDa erythrocyte membran	56118338	NP_00100815	4.43	0.99	0.45	0.24	0.49	0.34	0.8	2.36	0.4	0	0	0
1-2.c22240/1/1719.	PREDICTED: ribosomal RN	432843220	XP_00406557	3.32	6.78	5.87	0	0.54	3.33	4.49	0	3.01	0	0	0
1-2.c30016/1/1498.	--	--	--	10.6	14.65	10.53	7.49	5.63	2.98	3.48	6.56	10.19	1.8	0.17	0.88
2-3.c6918/4/2294	PREDICTED: matrilin-4 isof	512873848	XP_00491871	0.75	4.13	2.45	3.55	0.92	0.43	0.76	1.41	3.28	0.55	0	0

1-2.c11418/2/1526	--	--	--	12.49	12.74	21.85	4.89	8.19	2.21	4.71	9.96	14.27	3.74	6.37	0
1-2.c39487/1/1359	PREDICTED: sodium/hydro	847167859	XP_01282701	0.99	4.56	0.53	0.62	0	1.22	0.47	0	1.8	13.8	14.95	7.52
1-2.c41168/1/1415	--	--	--	0.45	0	0.63	0.65	0.11	0.28	0.56	0	0	0.36	5.64	9.67	5.92
1-2.c52394/5/1494	vdac1 protein [Xenopus (Silu	138520005	AAI35546.1	40.37	45.94	49.43	45.59	42.77	74.62	80.93	54.48	67.24	38.68	31.7	18.8
3-6.c18314/1/3879	pentraxin 3, long precursor [X	148226508	NP_00109115	1.81	0.44	3.46	1.2	1.38	0.12	1.29	0.81	1.28	0	0.22	0.25
1-2.c38229/1/1714	gltscr2-prov protein, partial [.	56789334	AAH88785.1	1.92	2.09	0	0	4.96	13.93	0	0	0	3.45	0	3.11
2-3.c24341/1/2050.	--	--	--	1.88	31.61	1.41	2.94	1.19	6.31	3.29	2.03	3.82	29.7	53.3	21.63
1-2.c24544/1/1472	--	--	--	0	0	0	0.5	0	0	0	0	0	1.19	3.57	2.91
2-3.c33937/1/2356	PREDICTED: N-acetylgalact	301608268	XP_0029337(	3.12	0	0.92	0	0.42	0	0	1.26	0.97	0	0	0
1-2.c49810/2/1367	muscle creatine kinase [Xeno	147899942	NP_00108007	1.69	0	4.25	6.34	4.09	3.44	3.91	7.66	2.67	0	1.64	0.63
1-2.c20748/1/1825	PREDICTED: transmembran	847095053	XP_01281107	2.37	5.4	0.96	3.12	2.26	1.99	4.01	4.47	1.73	0.44	0.45	1.04
1-2.c22375/8/1504	PREDICTED: uncharacterize	768435294	XP_01155908	9.33	64.27	10.09	4.19	14.56	43.87	9.35	5.7	8.68	0	2.25	3.96
2-3.c19749/1/1999	PREDICTED: lysosomal alpl	512873200	XP_0029325(	0	1.42	1.26	0	2.65	0.31	1.71	0.99	0	0	0	0
1-2.c43050/1/1553	Geneb-a protein [Xenopus la	213623820	AAI70263.1	0	6.72	0	0	0	3.03	1.17	0	0.14	22.91	4.08	39.98
1-2.c7763/2/1119	PREDICTED: cytosolic non-	591359345	XP_00705465	51.14	0	7.18	46.92	72.6	0.08	39.01	50.99	36.91	309.27	282.3	479.34
2-3.c20139/1/2023	--	--	--	0.51	5	1.16	0.34	1.59	3.44	1.58	0.56	0.7	13.35	10.78	11.24
1-2.c49121/1/1455	PREDICTED: NAD kinase 2	847088841	XP_01282125	0	0	0	0.48	0	0	0	0	0	3.59	3.68	0.43
1-2.c50205/1/1671	PREDICTED: cellular nuclei	594662064	XP_00717923	95.43	181.71	75.84	70.47	70.4	259.21	93.54	64.13	71.02	31.41	39.33	30.31
3-6.c14757/1/2938.:	--	--	--	3.55	7.36	1.43	3.07	1.6	3.36	3.29	2.07	2.84	46.26	58.84	37.36
1-2.c1958/39/1177	nucleolar protein NO38 [Xen	147902126	NP_0010797(	390.58	879.25	260.23	235.75	210.17	337.34	273.83	254.25	272.19	97.14	149.65	119.74
1-2.c41495/1/5068.	PREDICTED: fructose-bisph	530658308	XP_00531468	43.7	0	19.91	31.05	27.13	1.94	17.36	26.26	29.76	10.93	7.58	8.69
2-3.c10907/2/2583	gsn protein [Xenopus (Silurai	159155320	AAI54876.1	19.24	42.84	20.24	17.37	19.15	20.14	11.26	11.14	19.37	7.83	0.55	7.38
3-6.c14123/1/3297	PREDICTED: sialomucin coi	73973725	XP_532256.2	0.31	0	0	0.28	11.14	5.11	7.84	6.03	5.74	5.08	2.61	0
2-3.c57408/1/2154	eukaryotic translation initiatic	148237458	NP_0010800(	16.8	0.65	9.43	10.8	7.14	1.14	8.14	10.71	8.77	0.66	9.49	1.18
2-3.c35589/1/2062.	hypothetical protein L798_06	646716259	KDR19588.1	1.71	15.02	1.86	3.88	1.39	2.75	2.2	1.2	2.58	34.12	54.25	15.74
3-6.c8209/1/3015.1	--	--	--	1.29	0	4.55	12.95	10.28	4.68	16.18	9.48	14.49	6.21	2.4	5.56
2-3.c34660/1/2540	eukaryotic translation initiatic	148232020	NP_00108075	0	0.93	0.06	1.72	0.44	0.69	0	0	0	1.8	0	2.41
1-2.c39887/1/1748	PREDICTED: LOW QUALI	548522118	XP_00569813	7.19	4.15	8.53	4.83	4.31	4.39	2.2	5.06	5.58	0	0.43	2.22
1-2.c36814/1/1323	cystathionine-beta-synthase [	148228062	NP_00108682	22.88	68.35	21.72	16.66	11.37	41.3	32.29	26.53	19.37	9.06	18.23	5
2-3.c53002/1/2182	PREDICTED: succinate dehy	847123585	XP_01281758	12.65	5.51	8.49	13.35	12.38	4.61	7.8	7.75	11.63	2.76	2.26	4.34
2-3.c56434/1/2284	PREDICTED: coiled-coil doi	641766364	XP_00816802	0	0	0	0	0.57	0.08	0	0	0	0.23	1.91	2.45
3-6.c16732/1/4896.	PREDICTED: elongation fac	557264210	XP_00601757	532.46	611.95	310.33	387.49	394.4	489.56	332.88	415.87	454.15	214.75	95.59	232.53
1-2.c18004/1/1247	--	--	--	31.58	26.55	77.44	47.12	2.19	7.72	55.16	37.04	2.19	0.75	0	3
1-2.c41259/1/1211	dipose differentiation-related	89266853	CAJ82576.1	26.58	18.28	0.35	36.69	17.1	5.22	39.14	14.36	10.44	155.12	186.03	191.24
1-2.c23812/1/4040.:	Myozenin-1-like [Xenopus la	148237556	NP_00107945	8.35	0	5.66	7.07	5.74	4.39	3.93	5.86	5.56	0	0.32	2.32
1-2.c12069/1/1653	PREDICTED: myosin-bindin	556950430	XP_00598765	0.78	0	1.05	1.67	1.1	0.83	0.45	2.29	1.17	0	0	0.1
2-3.c23623/1/2574	--	--	--	10.27	11.1	4.15	4.18	4.62	7	15.24	5.5	4.03	2.09	0.63	0.62
2-3.c59921/1/2215	--	--	--	3.36	4.73	1.16	3.12	1.75	2.09	2.76	1.15	1.32	20.19	18.17	15.81
1-2.c2584/9/1436.1	PREDICTED: uncharacterize	847175141	XP_01281044	76.56	5.14	11.65	43.32	56.15	3.09	49.89	56.19	45.17	519.93	323.19	442.6
2-3.c39807/2/1874	PREDICTED: UBX domain-	301619867	XP_0029393(	0	0.75	0	0.2	0	0.44	0	0	0	3.01	3.32	0
2-3.c34128/1/2609	AMP deaminase 3 [Xenopus	71895767	NP_00102568	28.66	0.65	12.24	13.64	25.15	0.25	7.13	20.93	11.64	3.45	0.86	2.59
2-3.c47283/1/2648.:	PREDICTED: elongation fac	557264210	XP_00601757	727.64	1584.29	640.33	490.95	544.14	628.08	406.41	375.07	584.85	141.01	125.4	310.36
2-3.c62272/69/2529	cyclin I [Xenopus laevis]>gi|:	148228521	NP_00108357	39.49	45.5	30.15	20	23.54	17.39	11.03	22.37	25.14	7.5	4.4	12.03
3-6.c11189/1/3345	PREDICTED: filamin-B isof	512839151	XP_00491422	2.13	5.47	1.38	1.36	0.95	0.6	1.86	1.17	1.11	9.19	10.22	21.43
1-2.c32962/1/1359	--	--	--	8.08	88.02	6.12	10.5	6.08	18.27	8.46	5.97	12.3	77.88	142.57	60.93
2-3.c16494/1/2040.	--	--	--	9.7	8.42	14.92	5.12	5.33	17.25	6.27	9.62	16.94	1.93	4.08	1.18
1-2.c16850/6/1326	PREDICTED: MKI67 FHA c	327266746	XP_0032181(	0	65.8	35.25	24.18	17.72	19.77	23.89	29.52	84.99	14.4	0.87	0
1-2.c23282/1/1451	tropomodulin 4 (muscle) [Xe	148233551	NP_0010873(	10.07	3.9	26.76	17.11	14.55	19.17	23.37	19.57	24.3	13.14	7.24	6.14
2-3.c16255/1/2324	hypothetical protein N340_0:	678134232	KFV03414.1	4.29	61.95	4.71	9.22	4.49	12.56	7.56	4.76	8.93	52.37	92.11	43.45
2-3.c40106/1/6028.	calsequestrin 1 (fast-twitch, s	148231736	NP_00108022	63.41	8.95	93.57	98.2	130.18	83.51	121.78	117.42	122.78	65.73	56.58	45.72
1-2.c25887/1/1332	--	--	--	4.25	0.73	0.25	3.73	0.97	5.25	2.94	0	4.24	35.09	29.76	13.26
1-2.c5924/1/4751.2	PREDICTED: fructose-bisph	731506121	XP_0105968(	26.55	6.28	33.44	52.01	31.32	4.79	24.41	28.92	60.5	10.02	18.84	7.57
2-3.c19378/1/1970	PREDICTED: non-syndromi	847139923	XP_01282102	262.8	4.26	39.02	119.08	158.19	1.65	97.38	140.7	132.43	47.41	34.44	82.67
2-3.c23308/1/2203	--	--	--	0.23	0	0.09	0	0.12	0	1.08	1.32	0	0	0	0
3-6.c15842/1/3726.:	Ca2+-ATPase 1 [Rana clamit	12055497	CAC20853.1	11.07	2.03	19.79	9.41	13.81	12.22	7.74	11.25	14.02	1.08	0.37	6.3
1-2.c43968/1/1614	--	--	--	4.77	15.87	5.3	6.59	3.6	3.11	4.77	4.52	8.65	2.62	0.27	1.66
3-6.c2673/9/3888	isoleucine--tRNA ligase, cytc	148231881	NP_00109069	31.9	66.9	25.13	24.46	22.6	48.39	23.59	18.74	21.48	5.56	7.13	14.12
2-3.c33808/1/2453.	von Willebrand factor A dom	148228482	NP_0010799(	4.64	5.21	5.24	4.42	10.97	9.87	11.55	4.44	6.33	45.48	42.55	97.77

1-2.c41097/1/5164.	PREDICTED: actin, alpha ca	803091489	XP_00401102	31692.48	5125.53	40180.95	36051.11	43549.69	40700.32	36770.55	49983.84	34909.23	24663.33	17659.18	14719.77
2-3.c39789/1/2655.	--	--	--	5.51	15.49	18.74	7.28	6.35	6.67	3.39	6.81	12.94	2.08	1.27	1.44
2-3.c37179/1/2366	PREDICTED: nascent polype	847099444	XP_01281224	26.28	0	0.91	11.17	13	0.25	4.44	15.51	13.39	0.24	4.83	2.81
1-2.c22974/1/5869.	collagen alpha-1(I) chain prec	58332412	NP_00101100	0	7.7	0	0	0	359.09	0	0	0	32.95	0	19.56
1-2.c37322/10/1793	BTB (POZ) domain containir	147900907	NP_00109018	23.89	21.58	18.31	16.34	20.05	8.51	9.41	17.15	16.79	4.25	2.77	8.91
2-3.c54875/1/2178.	mitochondrial pyruvate carrie	148235923	NP_00108884	464.57	157.89	357.51	383.2	451.35	222.14	376.71	368.03	432.81	176.25	173.09	164.34
2-3.c61046/1/2203.	PREDICTED: E3 ubiquitin-p	512849811	XP_00491562	40.32	28.91	34.56	37.5	21.22	24	18.8	35.49	41.47	11.24	8.43	20.16
3-6.c12854/3/2832	myosin heavy chain, partial [	4249701	AAD13772.1	2.98	0.41	40.02	33.37	22.41	0.14	41.66	11.32	10.49	2.37	1.5	0.64
1-2.c25049/1/1527.	--	--	--	2.09	43.86	3.64	7.36	2.36	11.07	2.72	2.5	7.3	42.99	87.15	36.11
1-2.c21994/4/1529	lymphocyte cytosolic protein	350994415	NP_00108021	43.62	3.77	8.28	35.84	35.39	2.18	21.7	30.8	36.53	275.84	266.61	213.24
1-2.c33476/1/1359	plasminogen activator inhibit	47575766	NP_00100122	30.37	36.78	20.7	22.83	18.24	22.77	17.98	20.89	24.74	7.46	7.18	9.85
1-2.c4153/45/1348	ribosomal protein L3-like [X	148223643	NP_00108803	415.51	20.1	306.18	353.31	217.74	163.41	254.78	215.05	189.78	118.84	139.02	57.71
2-3.c3839/2/2213.1	--	--	--	0	0	0	1.3	0	0	0	0.33	0	11.06	3.91	3.94
1-2.c15642/1/1567	--	--	--	8.26	19.62	38.24	27.76	17.81	49.37	39.18	20.33	49.88	25.34	9.12	10.27
2-3.c63424/1/1885	calsequestrin 1 (fast-twitch, s	148231736	NP_00108022	3.01	0.75	9.86	6.37	9.67	8.45	8.86	7.29	8.37	2.79	1.53	3.5
3-6.c16571/1/3739	Ca2+-ATPase 1 [Rana clamit	12055497	CAC20853.1	4.54	0.47	5.12	4.54	4.13	2.8	3.19	4.32	4.77	1.01	1.9	1.45
3-6.c2260/20/2990	PREDICTED: splicing factor	847091403	XP_01282544	21.99	24.22	9.5	9.48	14.21	10.51	5.38	12.31	9	2.96	2.46	5.04
2-3.c37060/1/2562	--	--	--	2.14	4.38	4.41	2.94	2.56	2.87	3.65	3.02	3.44	1.26	0.42	0.91
3-6.c4339/2/3095	PREDICTED: protein DGCR	847085100	XP_01281098	1.17	2.61	1.47	1.26	0	0	0.88	1.2	1.27	0	0.25	0.12
1-2.c44314/1/5036.	actin, alpha cardiac muscle 2	45361557	NP_989355.1	8662.19	1575.19	11703.18	11194.84	12611.92	11777.41	9631.32	13753.84	11230.27	6853.91	4273.78	5319.49
3-6.c6918/1/2848	--	--	--	132.33	339.06	158.86	178.09	173.2	251.44	149.65	164.38	95.46	78.5	62.82	54.37
3-6.c15910/1/3113	transmembrane protein 131 [	213982761	NP_00113555	2.3	1.97	2.03	1.15	2.57	2.49	1.99	1.7	1.88	0.59	0	0.44
1-2.c3233/1/1824.1	--	--	--	1.34	4.45	0.67	1.51	0.56	1.37	1.35	0.63	1.67	16.78	9.62	24.06
3-6.c13176/3/2921	PREDICTED: muskelin [Xer	301604525	XP_00293189	4.96	4.34	3.64	5.54	5.52	2.98	9.38	5	4.91	1.45	0.78	4.06
1-2.c39108/2/1326	PREDICTED: heme-binding	512861457	XP_00491719	0.65	1.87	0.18	0.9	0	0.42	1.44	0	0	10	10.44	8.75
1-2.c45479/1/1278	SEC62 homolog [Xenopus la	148234873	NP_00108684	1.88	7.17	1.42	0.15	0.33	4.41	1.09	1.09	2.02	0	0	0
3-6.c8715/1/2816.2	--	--	--	4.75	7.15	2.18	4	1.79	3.53	3.88	2.71	3.34	30.91	40.26	40.53
1-2.c10941/19/1720	etoposide-induced protein 2.4	62857461	NP_00101718	0	21.62	0	0.21	0.54	0.23	0.23	14.21	18.79	0.7	0.54	0.14
2-3.c15779/2/2246.	--	--	--	2.06	2.12	6.43	5.92	6.1	4.8	5.42	7.32	4.65	1.23	0	0.56
2-3.c31475/89/2147	5'-aminolevulinate synthase 1	148232986	NP_00108605	30.31	51.47	31.84	20.21	41.94	32.57	29.23	14.59	32.42	9.76	14.49	8.46
3-6.c19030/1/2763	PREDICTED: uncharacterize	827537520	XP_01254756	0	9.69	4.02	0	6.67	4.56	0	0	0.06	7.9	5.03	0
2-3.c42951/1/2353	inhibitor of Bruton tyrosine k	147902812	NP_00108492	4.38	6.6	4.93	2.29	5.14	6.7	4.96	2.67	5.35	1.49	0.85	1.23
1-2.c16867/1/1547	PREDICTED: collagen alpha	512835852	XP_00491377	6.41	74.34	12.44	5.08	9.09	37.38	4.12	5.37	4.95	0.71	0	2.24
1-2.c14379/1/1183	muscle creatine kinase [Xeno	147899942	NP_00108007	14.44	2.12	25.82	18.39	15.37	6.81	16.82	14.55	17.45	1.05	2.72	11.5
1-2.c33729/4/1837	PREDICTED: junctophilin-2	557262533	XP_00601678	24.33	23.08	47.34	14.98	18.6	36.37	37.98	21.45	33.7	5.29	7.45	23.8
2-3.c4534/1/2519	transcription factor 25 (basic	148237729	NP_00109140	0.22	6.79	0.42	0.8	0.48	1.36	0	0.24	0.4	8.06	14.65	3
2-3.c7381/1/2402	PREDICTED: L-lactate dehy	675698937	XP_00275513	74.39	6.32	67.6	190.34	127.42	35.98	73.27	61.83	155.69	42.86	34.91	35.74
2-3.c52702/1/2622.	--	--	--	0	0	0.34	2.17	0.64	0	4.2	1.78	0	0	0	0
3-6.c17927/1/3814	myosin heavy chain, partial [	4249697	AAD13770.1	0.08	0.04	0.21	0.35	0.42	0	0.49	0.44	0.26	0	0	0
2-3.c13088/1/2345.	--	--	--	8.24	55.62	5.35	9.33	6.63	15.9	22.69	7.91	6.77	119.55	118.74	115.08
1-2.c2610/4/1667	cysteine conjugate-beta lyase	148228780	NP_00108074	22.05	13.76	15.2	19.89	17.65	7.57	12.72	13.36	16.46	4.61	6.27	5.23
2-3.c1643/25/2088	arginine--tRNA ligase, cytopl	45361659	NP_989403.1	30.94	53.73	18.73	14.24	23.74	34.19	17.8	19.65	14.4	5.31	2.32	12.44
2-3.c2162/2/2024	4-aminobutyrate aminotransf	189230071	NP_00112152	7.14	0	2.57	4.46	3.62	1.19	5.44	3.55	3.44	0.51	1.15	1.44
2-3.c2075/12/2429	PREDICTED: transcription f	512866157	XP_00491777	0.21	0.56	0	0	0.47	0.41	0	0	0	2.51	1.09	0.67
2-3.c35761/2/2251	--	--	--	0	0.19	0	0	0	0.85	0.41	1.02	0.6	0	0	0
1-2.c33891/1/13293	uncharacterized protein LOC	148226670	NP_00107979	87.53	1.43	71.1	93.8	88.76	8.39	53.87	56.17	79.46	36.66	23.03	17.97
1-2.c3962/1/1460	PREDICTED: actin, alpha ca	803091489	XP_00401102	569.51	639.82	2568.28	4537.68	1706.55	1487.03	2770.22	1723.85	4251.09	1788.14	991.87	1226.11
2-3.c26155/1/2321	fragile X mental retardation s	147899444	NP_00108178	68.65	36.98	59.93	24.11	31.95	64.98	27.04	24.36	63.28	6.26	8.66	17.81
3-6.c15452/1/3111	PREDICTED: transcription e	512819310	XP_00491160	1.32	10.38	1.14	0.83	0.57	7.52	0.79	0.45	2.15	0.03	0	0.13
3-6.c17352/1/3759	Ca2+-ATPase 1 [Rana clamit	12055497	CAC20853.1	1.42	0.7	3.33	3.7	2.28	0.94	2.12	2.54	3.96	0.86	0.45	1.2
3-6.c3531/2/3282	Disintegrin metalloproteinase	213626841	AAI70225.1	0	3.49	0.88	0	3.09	1.18	0	0	0	1.51	0	2.61
2-3.c16381/1/2420	--	--	--	0	0	0	0	1.26	0	1.25	0	0.82	0	0	0
2-3.c36656/1/2219.	--	--	--	2.83	10.24	1.31	0.82	5	1.86	0.92	1	2.59	20.53	20.04	30.75
3-6.c4258/1/7410.2	--	--	--	0.86	4.86	1.31	2.37	1.14	2.59	2.32	0.98	2.82	34.64	37.13	29.13
2-3.c60391/6/2486	PREDICTED: aspartate--tRN	847163066	XP_01282572	166.02	117.03	119.35	117.25	109.59	117.76	82.14	71.38	102.89	46.61	27.78	42.04
1-2.c6930/1/1716	PREDICTED: myb-related tr	847171315	XP_01280905	0.08	6.48	0.28	0.37	0.44	0.48	0.4	0.22	0.1	3.61	9.82	4.48
2-3.c2766/2/1833	acetylcholine receptor subuni	148227704	NP_00108091	12.76	9.59	15.75	7.63	4.46	21.05	4.55	5.89	6.67	1.9	0.35	2.4
1-2.c39909/1/1309	NADH dehydrogenase (ubiqu	148225889	NP_00108733	140.85	116.54	120.27	116.42	137.84	121.28	95.23	111.74	121.72	46.7	63.3	43.96

1-2.c47054/4/1688	unnamed protein product [Ho	221040588	BAH11971.1	0	0	0	0	0	0.12	0	0	0	0.43	1.66	4.33
1-2.c38704/2/1288	PREDICTED: microtubule-as	847168440	XP_01280802	9.1	1.37	7.46	7.63	7.2	0	5.3	10.09	15.51	3.65	6.57	0.52
2-3.c43680/1/1897	calsequestrin 1 (fast-twitch, s	148231736	NP_00108022	6.46	1.02	11.09	8.91	11.83	9.34	9.56	9.84	11.77	4.3	3	3.9
1-2.c33241/1/1423.	--	--	--	7.72	9.28	1.3	2.5	2.71	4.06	2.93	2.51	6.36	52.59	45.71	35.29
2-3.c52987/1/2184.	--	--	--	14.66	35.7	3.09	11.35	6.12	5.97	7.31	8.87	5.96	78.45	134.84	52.93
3-6.c16410/1/2950	Unknown (protein for IMAG	197246230	AAI68800.1	1.38	0	0	0	0	0.66	1.11	0	2.13	0	0	0
1-2.c9838/3/1695	PREDICTED: LOW QUALI	507933757	XP_00467811	3.66	30.08	15.5	13.82	5.88	13.02	18.22	7.77	14.47	1.79	3.23	8.79
2-3.c23168/1/2591	PREDICTED: glycogen phos	564238421	XP_00627608	0.37	1.59	0.73	1.08	0.88	0	0	0.22	0	3.64	2.05	10.68
1-2.c35516/1/1250.	--	--	--	1.28	0.41	1.8	2.48	1.35	1.18	4.14	0	0.51	34.34	28.13	12.08
2-3.c33167/1/2448	PREDICTED: collagen alpha	512835856	XP_00491377	1.9	16.44	5.72	2.2	3.57	11.79	1.47	3.4	2.15	0.33	0.66	0.51
1-2.c36250/1/1731	--	--	--	1.13	2.42	0	3.9	0	0	0	2.18	6.07	0	0	0
1-2.c24591/3/1918	PREDICTED: cold shock do	847087980	XP_01281925	27.48	10.19	16.22	17.71	32.47	12.43	9.26	22.77	17.09	2.26	2.13	11.12
2-3.c63586/1/2000	calsequestrin 1 (fast-twitch, s	148231736	NP_00108022	13.42	2.02	24.97	22.35	24.02	19.41	24.91	26.45	24.71	12.06	10.59	10.45
2-3.c14998/1/2208.	--	--	--	100.53	37.55	60.13	81.04	97.3	62.43	89.56	94.02	87.5	53.19	39.71	28.98
1-2.c2556/21/1512	polymerase delta-interacting	62860016	NP_00101709	73.98	44.2	42.47	47.02	44.58	37.29	43.99	44.63	44.94	15.04	25.27	19.26
2-3.c3619/4/2319	PREDICTED: CCR4-NOT tr	847135304	XP_01282014	5.29	1.9	0.71	3.59	0.7	0	3.69	3.63	2.57	0	2.97	0
2-3.c46119/1/2026.	PREDICTED: cytoplasmic d	573907129	XP_00664128	10.19	0	4.56	0	0.16	0	0	9.58	4.55	0	0	0
3-6.c16264/1/3574.	PREDICTED: sacsin-like [X	847149439	XP_01282272	4.2	0.09	1.71	0	2.42	0.73	0	0	0	4.95	2.33	0
2-3.c40455/1/2250	--	--	--	0.91	7.76	0.32	0.71	0.13	6.92	0.29	0.85	0.52	6.71	8.47	8.75
2-3.c21327/1/2387	--	--	--	45.34	2.56	27.13	22	10	2.66	11.04	10.3	18.46	4.01	8.78	4.12
2-3.c42864/1/2052	PREDICTED: zinc finger pr	821456582	XP_01240918	0	0	0.92	0.45	0	0	0	0	0	3.28	2.33	0
1-2.c1763/54/1497	PREDICTED: LOW QUALI	554568265	XP_0058784	195.54	308.3	116.7	120.57	112.15	182.78	129.27	107.77	125.13	52.98	38.41	71
2-3.c56842/1/2665.	--	--	--	0.65	5.48	0.55	1.83	2.57	6.89	1.58	2.17	2.64	24.73	15.39	46.14
2-3.c60219/1/2684	--	--	--	1.82	8.71	0.65	1.43	1.43	3.52	3.26	0.69	1.71	18.66	29.05	13.16
3-6.c12012/4/3156.	PREDICTED: calpain-7 isof	847134772	XP_01281995	3.61	1.71	2.88	0	3.67	0	2.31	3.53	2.63	0	2.71	0
3-6.c16629/1/3186	hypothetical protein CAPTEI	443682880	ELT87315.1	3.99	63.61	3.55	7.35	3.46	11.48	5.52	3.69	9.28	52.43	98.02	42.06
1-2.c23673/1/1619	RecName: Full=Sarcoplasmi	9789732	Q92105.1	3.13	0.43	3.09	5.01	6.65	3.45	4.32	5.01	3.39	1.58	1.01	0.37
2-3.c8618/1/2477	PREDICTED: protein phosp	557024824	XP_0060125	4.11	0.44	0	4.66	3.3	0.12	3.77	1.2	1.13	0.35	0.58	0
2-3.c23264/1/2196	--	--	--	8.13	10.6	8.48	11.31	9.53	15.78	23.86	9.6	13.76	117.08	130.43	129.38
1-2.c3566/2/1828	DEAD (Asp-Glu-Ala-Asp) b	148228442	NP_00108267	5.84	13.11	3.81	4.12	1.3	0.35	9.97	6.21	9.48	1.78	2.13	4.37
1-2.c38627/7/1404	PREDICTED: LOW QUALI	637377851	XP_0081232	8.1	1.14	7.02	5.4	3.46	2.7	9.58	8.35	5.57	3.77	0.41	2.39
1-2.c41326/1/1283	lysozyme g [Ictalurus furcatu	308322267	ADO28271.1	1.96	9.29	6.92	4.96	6.57	3.74	3.76	4.99	5.39	1.08	0.8	0.65
2-3.c10121/1/2337	hypothetical protein LOTGII	676427143	XP_0090448	4.85	5.73	2.58	2.07	3.76	3.57	3.48	2.12	2.92	14.2	35.46	29.02
2-3.c12010/1/2021.	--	--	--	3	11.36	1.9	1.2	2.97	5.84	3.4	2.52	2.78	26.18	63.05	29.2
2-3.c36258/1/2897	--	--	--	1.45	0.95	0	3.11	1.59	0.98	5.14	2.22	0	48.89	55.35	16.96
2-3.c37376/1/2525	--	--	--	0	0.05	0	0	0	2.44	1.1	0	0.84	0	0	0
3-6.c16635/1/3634	ATPase, Ca++ transporting, c	148685413	EDL17360.1	2.05	0.37	3.27	2.62	3.17	2.21	2.38	2.56	2.9	0.33	0.2	1.57
3-6.c16672/1/3633	RecName: Full=Sarcoplasmi	9789732	Q92105.1	8.37	1.64	8.47	7.35	7.18	5.07	6.72	8.34	10.56	4.3	3.73	2.81
3-6.c7455/1/2766	--	--	--	3.17	4.15	8.56	2.36	2.22	12.72	3.86	2.16	4.17	0.4	0.76	1.52
1-2.c22044/5/1312	DIM1 dimethyladenosine tra	148233697	NP_0010896	5.14	20.05	6.27	5.67	4.05	19.77	7.68	3.25	3.41	0.54	1.14	0.97
1-2.c41975/4/1473	--	--	--	5.74	7.88	0	2.3	3.36	1.15	7.24	7.92	2.55	0.03	0	3.87
1-2.c24583/1/1504	PREDICTED: FYVE, RhoGl	847121379	XP_01281725	0.92	6.88	1.23	2.52	1.52	0.99	2.7	0.44	1.5	20.99	20.87	11.02
1-2.c38359/1/7801.	--	--	--	11.85	2.75	28.65	17.2	23.27	16.79	21.98	16.66	26.24	4.77	0	7.71
2-3.c4331/1/2018	myosin, heavy chain 4, skelet	47575800	NP_00100124	5.08	0	5.53	2.89	8.55	0	2.27	6.54	8.02	1.35	2.96	1.2
1-2.c11308/1/1339	PREDICTED: collagen alpha	847135925	XP_01282037	17.05	455.87	63.97	43.67	38.41	508.48	48.79	34.21	67.09	30.14	16.91	18.18
2-3.c56092/1/2135	PREDICTED: myosin-7B [X	512871888	XP_0029329	1.64	1.01	0.94	0.39	0.64	11.7	3.22	0.81	1.19	0.4	0	0.16
1-2.c24587/4/1759	PREDICTED: sorting and as	543379494	XP_0055329	145.46	121.95	122.08	126.64	102.31	97.59	102.12	109.52	107.57	51.66	50.62	48.6
1-2.c11455/1/1975	--	--	--	0	0	0	1.02	0	0.75	0	0.7	0	192.63	616.42	13.87
3-6.c7163/2/3453	uncharacterized protein LOC	147905311	NP_0010906	0	0	0	0.78	0	0.19	0	0.14	0.02	0.92	1.78	4.39
1-2.c46475/1/1217	PREDICTED: glutamine-rich	667335925	XP_0085930	0	37.12	0.21	0.21	0	0.29	0.81	12.39	19.26	0	0.6	0.86
2-3.c28936/1/6835.	methylcrotonoyl-CoA carbox	148229681	NP_0010860	4.62	2.08	2.67	4.74	1.29	3.95	2.97	3.49	8.72	0	0.24	0.45
3-6.c4391/1/2941	--	--	--	28.6	5.55	18.4	11.24	18.81	12.73	8.22	17.83	10.82	4.23	5.21	5.32
1-2.c17541/1/1368.	--	--	--	6.14	3.77	0.44	1.9	0.1	3.92	4.04	2.24	0.57	27.68	42.98	29.05
1-2.c36629/1/6877.	muscle creatine kinase [Xeno	147899942	NP_0010800	51.8	3.56	43.75	36.65	41.36	24.78	47.7	46.34	43.99	21.03	22.07	15.91
2-3.c3831/1/2068.1	--	--	--	3.68	70.27	3.09	7.75	2.68	14.18	6.25	3.9	9.57	62.66	115.87	46.06
1-2.c19985/1/1758	predicted protein [Nematoste	156353049	XP_0016228	0.16	0.25	0.46	0.16	0.29	0.26	0	0	0	1.45	1.83	2.79
1-2.c13598/1/1324.	PREDICTED: nuclear transc	512829742	XP_0029381	5.41	22.72	8.56	0.68	3.84	2.28	2.34	1.62	2.5	0	0	0
2-3.c32571/1/4608.	myosin heavy chain, partial [	4249701	AAD13772.1	0.41	0	1.96	1.58	1.18	0	2.37	1.44	2.51	0.34	0.59	0

1-2.c3586/2/1614.1	PREDICTED: cell cycle prog	847112286	XP_01281505	5.01	0	0	4.05	0.9	1.33	1.12	3.41	3.69	0.45	0	0
2-3.c46960/1/5277.	PREDICTED: alanine amino	410923795	XP_0039753	15.09	5.82	6.48	12.56	11.33	4.45	6.51	4.78	11.34	2.59	3.88	2.19
3-6.c3103/5/3177	segment polarity protein dish	183986681	NP_00111692	34.33	8.22	11.96	20.36	26.18	9.65	6.55	15.59	19.53	6.65	5.36	5.4
2-3.c15525/3/2302	PREDICTED: sarcalumenin [	701384503	XP_0099938	0	2.16	0	0	0	0	0	0	0	8.09	3.59	0
2-3.c22628/1/2040.	--	--	--	2.41	24.36	4.22	3.4	4.85	8.22	5.39	2.79	7.63	34.86	57.42	47.92
1-2.c50262/1/7274.	calsequestrin 1 (fast-twitch, s	148231736	NP_00108022	8.41	3.19	13.91	7.23	15.72	11.6	13.4	14.64	13.74	2.54	0	3.64
2-3.c55098/2/2255	Mimitin, mitochondrial precu	226372810	ACO52030.1	11.69	3.56	12.03	8.01	7.05	3.96	8.33	8.84	6.32	2.71	3.33	2.73
2-3.c30065/3/2184	PREDICTED: dnaJ homolog	301610031	XP_00293455	1.75	0	0.21	0	0	2.39	0	0	0.22	2.21	1.1	5.31
2-3.c5923/1/2107	PREDICTED: fructose-bisph	530658308	XP_00531468	195.26	4.26	70.3	133.6	118.22	9.25	59.37	101	99.57	45.65	26.17	49.94
1-2.c52360/19/1350	PREDICTED: myosin-4 [Xe	847156920	XP_0128244	6005.93	157.74	7513.13	6890.47	7658.41	43.96	5268.66	12600.73	7273.63	3321	2499.21	2079.81
1-2.c12166/2/1717	aldehyde dehydrogenase clas	4586546	BAA76412.1	56.15	42.33	38.01	43.26	35.99	40.66	36.12	43.39	34.04	13.45	14.49	22.17
2-3.c13944/3/2237	uncharacterized protein LOC	147906566	NP_00109117	4.5	14.33	8.69	5.12	2.78	14.32	7.43	6.96	6.28	3.48	1.34	2.39
3-6.c8858/1/3128	PREDICTED: unconventiona	847091229	XP_0128252	13.79	8.25	19.39	10.28	16.52	21.08	11.46	15.89	13.67	5.03	6.98	6.25
2-3.c5383/1/2075	PREDICTED: collagen alpha	664740187	XP_00852739	0	6.22	3.23	1.27	1.36	2.46	2.91	3.96	1.71	1.9	0	0
3-6.c13676/1/4353	PREDICTED: myosin-7 isof	657571574	XP_0082893	1.63	0	3.01	1.84	2.57	0.08	1.92	3.08	1.87	0.72	0.89	0.64
1-2.c34465/1/1777	myosin, heavy chain 4, skelet	47575800	NP_00100124	2.87	0	4.25	2.13	4.89	0	1.01	4.32	6.91	0.51	0.34	0.51
2-3.c1851/13/2369	Unknown (protein for IMAG	195539698	AAI68131.1	6.32	5.37	7.35	3.98	6.92	1.04	2.8	4.62	3.56	1.4	0.52	1.09
2-3.c30173/1/2544.	nuclear factor of kappa light	148230967	NP_00108699	29.1	0	25.61	20.07	19.85	15.36	0	0.07	12.65	46.95	53.7	43.74
2-3.c20383/2/2501.	--	--	--	3.24	6.59	5.3	3.61	2.11	3.38	0.75	2.75	4.05	0.63	0.3	0
2-3.c42072/1/2158.	PREDICTED: rho GTPase-a	847148811	XP_01282258	1.5	4.61	0.53	1.32	0.68	1.65	1.14	0.88	0.75	9.55	12.57	12.54
2-3.c20450/2/2598	PREDICTED: probable E3 u	847147382	XP_01282233	46.97	12.48	31.62	31.66	48.22	20.11	32.67	37.32	67.1	24.86	14.19	23.42
1-2.c23021/1/1237	PREDICTED: protein transp	301609492	XP_00293429	1.32	0	0	2.17	0	0	0.4	0	1.15	13.83	12.28	4.05
1-2.c27010/6/1628	PREDICTED: cytosolic 5'-nu	327275093	XP_0032223	20.02	7	26.08	18.74	38.06	5.49	10.19	30.7	23.73	7.39	1.93	8.3
1-2.c30051/1/1239	--	--	--	3.24	43.15	4.65	0.84	5.36	20.33	1.83	0	3.43	13.1	18.76	28.02
1-2.c44265/1/1354	PREDICTED: phosphate-reg	669271014	XP_0086274	2.96	8.34	1.23	2.27	1.11	10.37	6.74	1.25	3.99	33.29	43.93	33.18
2-3.c63488/11/2093	nucleolar RNA helicase 2 [X	113931550	NP_00103922	15.17	71.86	15.25	26.43	12.29	78.99	13.55	28.36	41.24	0	0	21.53
1-2.c34954/1/6762.	PREDICTED: fructose-bisph	530658308	XP_00531468	807.55	32.68	125.86	212.03	829.44	48.15	445.32	525.76	169.09	53.72	89.28	182.75
1-2.c25024/2/1300	--	--	--	5.85	74.73	8.38	5.77	7.26	5.87	4.93	7	6.19	3.04	1.17	0.42
1-2.c52062/2/1259	RecName: Full=Voltage-dep	46576355	O57483.1	140.86	58.62	159.57	148.36	162.49	208.18	257.74	183.51	219.04	119.79	127.41	72.07
2-3.c46041/1/2575	mitochondrial intermembrane	147905047	NP_00108467	14.75	19.56	7.55	13.59	12.37	12.79	8.5	5.13	12.97	3.32	2.83	3.88
1-2.c2043/25/1577.	homeobox protein DLX3 [El	46484683	AAS98251.1	3.49	14.09	5.99	5.83	2.62	13.95	17.65	4.19	5.49	51.11	58.64	122.17
1-2.c25710/1/1341	PREDICTED: cytochrome P	847139669	XP_0128209	0	0	0	0	0	0	0	0	0	7.22	15.48	0
1-2.c27894/1/1457	PREDICTED: protein FAM1	847095670	XP_0128111	21.01	9.68	16.46	10.19	10.25	7.97	7.94	12.9	12.97	1.94	5.99	4.46
2-3.c26529/2/2131	PREDICTED: chloride intrac	530655848	XP_00531393	0.07	0.26	1.19	1.15	0.15	0	1.5	1.08	1.17	0	0	0.22
1-2.c28400/2/1715.	Keratin, type II cytoskeletal c	678188672	KFV56394.1	13.32	5.22	7.98	7.68	7.28	0	5.95	4.96	5	2.07	0.67	0.19
2-3.c47102/1/2525	PREDICTED: trafficking pro	847136021	XP_01282041	0.4	0	0.24	0	0	0.2	0	0	0	2.11	0	1.75
2-3.c1841/28/2245	PREDICTED: peptidyl-proly	847168271	XP_01282723	7.24	137.99	8.08	5.37	15.93	86.62	11.78	7.33	7.4	6.86	3.81	0
2-3.c6401/1/2556.1	--	--	--	5.9	3.97	2.15	3.76	4.36	2.44	1.84	2.43	3.98	1.08	1	0
2-3.c13434/4/2534	PREDICTED: nebulin isofor	847166510	XP_01282661	0	0	16.78	0	0	0	0	0	0	1.91	1.91	0
1-2.c34728/1/9427.	PREDICTED: serine/threoni	831290265	XP_01267661	0	0	0	0	0	0	0	0	0	26.38	3.42	9.04
1-2.c1820/38/1504	PREDICTED: elongation fac	573886958	XP_0066312	0.18	25.11	1.42	2.12	0.71	10.15	2.77	1.18	2.73	0.37	0	0.16
1-2.c23496/2/1367	--	--	--	1.98	0	3.5	0	3.92	12.24	14.39	0.01	0	41.57	41.08	40.56
1-2.c39494/1/1466	--	--	--	20.14	0.66	11.46	17.14	18.48	2.44	11.86	14.03	18.27	2.28	6.68	7.71
3-6.c16351/1/2840.	hypothetical protein N332_0	677416451	KFQ22048.1	37.67	10.53	26.47	35.79	33.12	18.16	27.41	33.07	37.6	12.35	12.82	13.12
2-3.c30680/2/2103.	--	--	--	9.88	5.88	16.69	4.6	13.15	3.27	4.08	11.58	5.19	0	1.08	1.67
1-2.c18074/1/1389	--	--	--	3.73	10.9	1.6	4.25	1.79	6.48	2.67	2.98	2.93	22.53	39.82	26.74
2-3.c7778/1/2540.2	--	--	--	2.08	1.82	0	0	3.41	4.46	0	0	0	0	19.22	16.63
2-3.c33655/1/2412	--	--	--	2.78	95.18	5.61	13.62	4.44	26.43	12.2	6.02	19.35	146.85	328.94	126.32
1-2.c25734/1/6191.	PREDICTED: creatine kinas	556963392	XP_00599165	17.09	2.68	25.47	23.38	25.36	10.32	18.6	18.16	26.55	7.53	7.33	9.05
2-3.c47638/1/1882	PREDICTED: lamina-associ	847176929	XP_01281093	1.5	11.8	1.31	1.83	1.32	4.85	0.71	0.87	1.76	18.34	27.99	7.92
2-3.c54359/1/2621	--	--	--	2.72	19.09	1.65	2.41	1.65	3.97	1.71	1.35	1.4	15.74	21.29	8.45
1-2.c33181/1/5736.	MGC64482 protein [Xenopu	54261690	AAH84349.1	16306.93	468.08	9576.89	15362.97	15070.11	1318.89	13619.22	12840.7	17769.58	8221.06	7591.35	5796.72
2-3.c32221/1/1889	PREDICTED: periodic trypt	301618674	XP_00293873	8.23	16.12	4.77	2.87	4.07	10.46	5.59	4.17	3.06	0.73	0.61	1.83
3-6.c13151/2/2853	--	--	--	9.26	7.06	6.45	6.95	4.05	2.15	8.36	4.61	6.83	2.11	2.14	2.94
1-2.c49343/1/1553	PREDICTED: SPRY domain	558216658	XP_00613488	0.41	6.25	0.5	0.5	0.64	1.93	1.14	0.52	0.42	11.67	8.24	10.38
1-2.c29961/1/1540	larval type I keratin [Rana ca	134140868	ABO61147.1	3.61	0.97	5.48	2.12	1.91	0.34	1.84	6.27	5.32	2.1	0.81	0.6
1-2.c32925/1/1281	PREDICTED: mRNA turnov	641759304	XP_00816553	19.24	44.23	13.61	10.37	12.5	28.14	17.71	10.02	10.42	4.42	4.69	3.99
2-3.c38913/1/2265	mannosidase, alpha, class 2A	148226300	NP_00108556	0	9.25	2.54	0.33	1.5	15.69	2.72	0.76	4.32	1.03	0	0

1-2.c9285/1/1396	Proteasome activator comple	226372158	ACO51704.1	5.91	10.37	3.49	2.88	4.31	5.67	5.41	4.69	3.29	1.95	0.76	0
3-6.c7531/1/3246.2	--	--	--	17.57	5.55	9.29	12.58	15.16	7.85	25.01	10.97	14.64	169.52	127.56	149.34
2-3.c46822/1/2176.	PREDICTED: G-protein cou	512840252	XP_00293169	4.64	1.97	3.44	3.02	1.11	0.99	1.51	2.37	2.72	21.74	20.91	36.25
1-2.c50276/1/1755	PREDICTED: unconventiona	602734247	XP_00745249	0	0	0	0	0	0	0	0	0	4.56	2.88	0
1-2.c6280/10/1525	methylglutaconyl-CoA hydra	186910214	NP_00111953	35.46	32.7	23.66	32.03	27.52	23.76	26.51	27.59	28.93	9.76	8.54	16.26
1-2.c1861/39/1628	PREDICTED: dnaJ homolog	847134514	XP_01281987	58.49	59.19	41.95	41.82	36.1	0	0	23.29	8.95	0	0	3.34
1-2.c1924/24/1739	PREDICTED: cytosol amino	847087076	XP_01281622	0	0	0	3.32	0	0	0.3	0	0	1.24	4.96	4.29
1-2.c2309/3/1780	PREDICTED: hypothetical p	297293110	XP_00280419	0	0	0	1.62	0.28	0	0.42	1.46	0	2.97	1.7	3.43
1-2.c2378/27/1457	plasminogen activator inhibit	47575766	NP_00100122	1.05	170.9	105.58	104.48	0	0	0	0	0	0	0	23.76
1-2.c2474/13/1790	PREDICTED: zinc finger prc	847114219	XP_01281539	2.22	4.89	0	0	0	0	1.55	0	0	0	0	0
1-2.c2726/9/1437	PERP, TP53 apoptosis effect	147901359	NP_00108623	10.11	37.74	6.21	2.87	7.72	13.73	1.79	2.93	9.41	2.95	0	1.6
1-2.c2787/5/1778.1	PREDICTED: phosphatidyle	641720285	XP_00814978	1.06	9.8	5.03	0	5.92	2.23	0	0.2	0	0	0	0
1-2.c2807/5/1288	NADP-dependent leukotriene	226371914	ACO51582.1	3.48	11.18	4.91	1.47	3.93	1.71	4.56	1.24	1.3	0.28	0	0.23
1-2.c3023/1/1623	hypothetical protein CAPTEI	443723767	ELU12037.1	9.56	8.05	5.97	28.42	19.17	2.83	45.51	16.41	21.75	181.98	205.76	105.28
1-2.c3107/8/1674	SUMO-activating enzyme sul	148227698	NP_00108525	0	0	0	0.03	7.6	17.79	7.85	0.12	7.85	0	14.32	0
1-2.c3276/2/1636	PREDICTED: exostosin-like	602651973	XP_00743236	0	0	0	0	0.93	0.12	0	1.14	0.9	6.26	3.1	2.57
1-2.c3522/1/1244	PREDICTED: keratin, type II	852796039	XP_01288789	0	0	0	0	1.24	9.32	4.14	0	2.23	16.28	28.7	99.03
1-2.c3783/2/1431	beclin-1 [Xenopus laevis]>gi|	147903463	NP_00108575	0	0.99	0	2.6	0	0	2.88	0	0.54	11.34	16.56	7.98
1-2.c4116/2/1427	--	--	--	0	0	0	0	14.17	15.72	12.6	18.92	27.27	0	32.78	0
1-2.c4603/1/1001	Eukaryotic translation initiati	226372362	ACO51806.1	360.22	812.52	383.72	43.7	299.49	366.47	235.1	30.9	398.19	0	0	77.4
1-2.c4896/1/1495	PREDICTED: BMP-2-induci	512815037	XP_00293405	0	0	0	2.84	0	0	0	0.41	1.15	4.13	7.3	7.04
1-2.c5056/1/1837	--	--	--	4.16	2.88	0.92	0	0.22	0	1.01	0.03	0.56	0	0	0
1-2.c5277/1/1281	keratin, type I cytoskeletal 18	148234658	NP_00108081	91.39	246.52	74.16	16.52	0	0	0	0	0	0	0	0
1-2.c5359/8/1332	39S ribosomal protein L45, m	148235359	NP_0010795	14.74	21.13	9.7	11.11	4.09	4.76	2.18	3.51	1.98	2.35	0.37	1.4
1-2.c6293/1/1210	transgelin-2 [Bufo gargarizan	635377487	AHZ89387.1	20.21	2.01	6.63	42.19	2.42	11.45	85.08	12.75	32.13	277.79	275.8	160.02
1-2.c6592/3/1172	PREDICTED: 60S ribosomal	742249727	XP_01086661	0	800	143.51	13.4	0	0	0	0	0	0	0	0
1-2.c6649/7/1612	cdc42 effector protein 2 [Xen	113931434	NP_00103916	0	0	0	3.46	0.52	3.06	2.84	1.01	0	14.45	0.42	14.04
1-2.c6700/1/1307	F-box only protein 46 [Xenop	348605211	NP_00110647	0	0	0	5.68	0	0.97	0	0	9.57	5.04	5.04	15.14
1-2.c7025/2/1815	PREDICTED: nucleolar GTP	512849296	XP_00293522	0	0	0	7.32	0	0	0	2.41	0.11	9.22	10.76	9.13
1-2.c7305/6/1464	DET1- and DDB1-associated	147903603	NP_0010885	1.21	3.84	4.26	3.4	0.75	0	1.74	0	0	0	0	0
1-2.c7625/1/1347	--	--	--	2.67	0	0	3.6	2.49	4.27	9.19	4.35	7.76	50.01	52.34	35.84
1-2.c7698/2/1539	dual specificity mitogen-activ	56118586	NP_00100805	0	0	0	4.81	0	0	21.68	8.2	11.91	24.73	17.65	8.51
1-2.c8008/7/1320	Tubulin alpha-1C chain [Lari	808859777	KKF11870.1	0	0	0	11.44	0	0	13.73	0	11.2	25.81	11.65	20.03
1-2.c8010/2/1543	--	--	--	0	0	0	1.2	2.55	0	4.26	0	14.42	0	58.12	20.5
1-2.c8126/2/1605	RecName: Full=MMS19 nuc	408407685	Q0V9L1.2	0	0	0	0	5.28	8.07	0.45	6.2	5.63	2.77	14.89	0.51
1-2.c8201/1/1713	PREDICTED: protein FAM1	759159550	XP_01137198	0	0	0	8.52	0	0.1	0	7.48	8.55	21.5	0	0
1-2.c8242/1/1734	PREDICTED: collagen alpha	847166754	XP_01282667	2.36	15.36	2.56	0.15	4.37	18.86	0	0	2.27	0.18	0	0
1-2.c8435/1/1348	E3 ubiquitin-protein ligase ar	183986725	NP_00111695	0	0.75	0	1.37	0	0	0	0.9	1.26	15.95	10.19	5.21
1-2.c8459/1/1276	non-POU domain containing,	148235052	NP_00108073	0	0	0	0	0	0.57	1.8	0.57	2.27	14.62	15.26	0
1-2.c8693/1/1427	PREDICTED: uncharacterize	847110867	XP_01281479	2.64	7.11	1.1	0	1.09	0	0	0	0	0	0	0
1-2.c8731/2/1724	--	--	--	7.48	6.61	3.83	20.76	13.6	5.76	99.83	5.8	7.77	158.12	110.61	113.87
1-2.c8818/2/1270	myelin expression factor 2 [X	62859395	NP_00101603	6.06	18.61	0	0	0	0	0	0	0	0.1	0	0
1-2.c8928/1/1605	PREDICTED: canalicular mu	847167781	XP_01282697	2.67	0	0	3.9	2.94	0	15.41	3.35	0	24.77	28.6	10.58
1-2.c9409/1/1412	--	--	--	7.4	0	0.91	11.01	7.86	18.42	17.29	11.31	18.06	55.97	74.49	50.26
1-2.c9415/2/1438	PREDICTED: glycine amidir	874493879	XP_01296181	21.15	0.94	13.17	0.08	3.64	0	0.39	3.88	0	0.16	0	0
1-2.c9519/1/1861.1	actinin, alpha 1 [Xenopus lae	148224373	NP_00108429	0	9.71	0	7.89	0	0	30.44	0.73	2.25	70.57	75.14	16.33
1-2.c9541/1/1457	--	--	--	5.46	0	2.14	11.91	6.22	14.92	23.55	4.81	5.21	48.14	63.08	52.35
1-2.c9850/1/1409	DEAD (Asp-Glu-Ala-Asp) bo	148228442	NP_00108267	1.02	0	0	0	2.69	1.67	0	0	9.83	19.91	22.56	10.15
1-2.c10634/12/1405	E3 ubiquitin-protein ligase K	148224862	NP_0010857	4.28	1.96	1.03	0.01	2.12	0	0	0.83	0.5	0	0	0
1-2.c11312/1/1587	--	--	--	0	0	0	0	0	0.47	5.19	1.12	4.19	2.69	14.31	5.01
1-2.c11348/3/1430	--	--	--	0.98	1.29	0	3.01	0	0.3	3.9	1.5	3.88	9.66	19.08	23.1
1-2.c11718/1/1384	PREDICTED: myosin-4 [Xer	847156920	XP_0128244	76.43	27.63	54.98	0	19.01	2.61	0	0	0	0	0	0
1-2.c11779/1/4901.	solute carrier family 35 meml	742627585	NP_989845.2	15.84	4.63	8.68	9.7	2.87	2.2	4.18	7.96	4.12	0	0	0
1-2.c11964/1/1183	60S ribosomal protein L4 [Xe	284009776	NP_0011650	6.13	6.21	5.22	1041.26	4.14	6.27	1156.09	933.43	1120.28	990.56	1706.83	0.95
1-2.c13126/2/1430	glucocorticoid receptor [Xenc	456374	CAA54804.1	0	0	0.07	4.02	0	0	5.39	0	1.3	10.22	4.67	6.32
1-2.c13137/1/1634	--	--	--	3.5	0.27	1.86	4.71	0	0.15	2.24	0.02	0.4	0	0	0
1-2.c13449/3/1457	PREDICTED: succinate dehy	847123585	XP_01281758	46.05	16.04	35.8	0.2	0	0	0	0	0	0	0.3	0
1-2.c13866/1/1614	--	--	--	6.68	3.84	6.63	1.66	3.96	3.89	2.33	0	5.09	1.13	0	0

1-2.c13998/1/6518.	LOC431836 protein, partial [	62739321	AAH94088.1	18.38	14.58	11.94	13.85	0.08	0.09	0	11.3	0.09	0	0	0
1-2.c14179/1/1285	--	--	--	0	106.61	35.24	0	0	0	0.26	0	14.29	0	0	0
1-2.c14278/2/1296	LOC398627 protein [Xenopu	83318199	AAI08446.1	0	0	0	16.09	0	0	5.87	0.53	14.56	66.7	85.87	0
1-2.c14632/1/1526	zinc finger protein 326 [Xeno	213624144	AAI70704.1	0	0	0	0	0	2.13	0	1.13	0	8.86	24.38	2.18
1-2.c14766/1/1683	--	--	--	0	0	0	4.94	0	3.07	3.63	3.11	0.54	6.83	10.62	4.88
1-2.c14799/1/6602.	basic leucine zipper and W2 (	147906588	NP_00108029	11.51	14.63	0.03	0	18.34	6.3	8.84	2.37	0	0	0	0
1-2.c14883/2/1782	--	--	--	2.38	25.58	8.75	1.81	1.22	9.87	0.43	1.24	1.6	1.92	0.95	0
1-2.c15670/2/1498	PREDICTED: COP9 signalos	512840748	XP_00491444	9.89	0	6.6	0	4.61	0	0	0	0	0	0	0
1-2.c16496/3/1779	PREDICTED: RNA-binding	686617738	XP_00928583	3.54	14.52	2.68	2.16	1.61	4.28	1.77	1.56	1.97	0.06	0	0.67
1-2.c16856/1/1239	PREDICTED: choline-phosp	817290393	XP_01231551	0.16	0	0.12	19.27	17.73	13.91	0.23	16.85	0	42.12	35.01	0
1-2.c17151/3/1672	PREDICTED: serine/threonir	512855246	XP_00293928	0	0	0	0	0.89	0	1.25	0	0	3.99	0	5.56
1-2.c17296/1/1770	PREDICTED: endogenous re	831566752	XP_01273347	5.56	0	0	2.37	0.98	7.02	31.81	8.28	7.5	38.51	51.23	15.6
1-2.c17563/1/1509	uncharacterized protein LOC	163914535	NP_00110635	0	0.93	0	2.95	2.6	11.09	0.25	1.28	2.15	9.79	10.55	8.44
1-2.c17578/1/1449	--	--	--	0	0	0	0	0	1.04	0	0	0.62	12.28	0	10.97
1-2.c18047/1/1277	TPA: hypothetical protein BC	296473158	DAA15273.1	6.34	24.88	4.36	10.24	10.37	47.81	125.53	31.77	42.55	212.68	177.99	141.3
1-2.c18085/1/1797	PREDICTED: G-rich sequen	847162878	XP_01282565	1.36	13.64	7.38	0	2.09	0	0	0	0	0	0	0
1-2.c18218/1/1799	PREDICTED: immunoglobul	847130950	XP_01281911	0.69	4.67	0.95	1.13	2.14	0	0	0.95	2.93	0	0	0
1-2.c18232/7/1354	high mobility group nucleoso	284795352	NP_00108076	0	0	0	18.68	0	0	14.47	0	0	72.67	112.7	4.33
1-2.c18236/1/1755	CCR4-NOT transcription con	147903571	NP_00108665	0	0	0	0.52	0	0	0	3.03	2.35	2.86	0.12	4.67
1-2.c18764/5/1645	PREDICTED: N-acetyltransf	301620503	XP_00293960	2.48	5.88	1.1	1.8	0	1.28	0.28	0	1.87	0	0	0
1-2.c18786/1/1542	PREDICTED: E3 ubiquitin-p	847166327	XP_01282653	0	0	0	0	0	0.3	0.58	0	0	4.03	7.2	0.77
1-2.c19243/1/1384	--	--	--	0.58	0	0	0.31	0	1.78	8.66	4.06	1.47	32.4	75.98	14.4
1-2.c19308/2/1529	E3 ubiquitin-protein ligase RI	56118680	NP_00100801	0	0	0	2.24	4.19	0.82	3.57	0	11.81	7.25	25.06	4.49
1-2.c19588/2/1553	PREDICTED: spectrin alpha	847152581	XP_01282324	0	0	0	0.8	0	0	4.52	2.49	6.47	16.08	13.47	0
1-2.c19787/2/1191	--	--	--	1.78	0	0	0	1.92	4.1	9.01	10.46	0	72.86	108.55	13.79
1-2.c19845/1/1595.	trifunctional enzyme subunit	45361293	NP_989224.1	31	58.47	18.59	67.57	51.67	73.88	91.5	57.09	63.74	512.15	1037.87	454.32
1-2.c22059/2/1586	RAD9 checkpoint clamp com	147905155	NP_00108260	0	0	0	0	1.89	7.88	0	0	3.17	10.7	5.55	6.74
1-2.c22096/2/1329	--	--	--	0.8	0	0	5.44	0.45	3.18	2.84	0	4.32	15.03	23.19	16.53
1-2.c22108/3/1709	uncharacterized protein LOC	73853872	NP_00102751	0	0	0	0	0.11	0	0.42	1.73	1.28	2.7	2.46	2.86
1-2.c22413/1/1891	PREDICTED: PHD finger pr	301606656	XP_00293290	7.49	6.36	6.49	2	3.6	2.72	3.44	0	3.09	0.44	0	1.07
1-2.c22776/1/1189	serine/threonine kinase 25 [X	148234581	NP_00108494	0	0	0	2.15	0	0.03	0	2.46	0.66	6.39	21.09	6.08
1-2.c22893/1/1518	--	--	--	28.8	2.99	1.51	109.15	0	35.38	46.83	34.69	96.82	300.37	406	166.73
1-2.c23192/1/1245	--	--	--	0	0	0	0.9	0	0	2.93	0	0	12.1	4.6	3.08
1-2.c23384/3/1171	ribonuclease H2, subunit A [	301171214	NP_00118033	9.87	33.11	8.99	5.39	6.58	12.59	6.26	6.58	5.21	1.76	1.69	1.13
1-2.c23519/1/1716	--	--	--	0.14	0	0.28	3.01	0	0.72	3.14	1.36	0.08	6.97	8.43	10.73
1-2.c24124/1/1306	PREDICTED: protein phosph	512847586	XP_00491535	0	0	0	0.71	0	0	0.55	1.53	0	9.17	11.77	0
1-2.c24192/1/1350	hypothetical protein H696_0	694544462	XP_00949791	0	0	0.17	0	1.59	4.52	4.66	1.37	0	0	40.64	31.22
1-2.c24437/1/1525	--	--	--	0.94	0	0.26	2.2	1.21	5.44	4.01	0.96	0	19.61	22.68	6.59
1-2.c24509/1/1612	solute carrier family 17 (acidi	189217614	NP_00112125	0	0.37	0	0	1.02	0.6	0	1.79	1.73	12.75	6.78	4.17
1-2.c24521/3/1543	RecName: Full=Vimentin-4 [	138532	P24790.1	59.73	36.15	28.9	24.18	0	3.56	0.96	2.15	0	1.32	0	0
1-2.c24732/1/1241	RNA-binding motif protein,	148237346	NP_00108498	0	0	0	0.98	0	0	1.62	0	0	17.35	28.28	2.12
1-2.c24904/1/1269	PREDICTED: 5-azacytidine-	530611118	XP_00529716	0	0	0	2.88	0.23	0	1.31	0	1.38	8.94	5.07	7.68
1-2.c25077/2/1228	--	--	--	0	0	0	0	0	33.18	9.39	9.7	0	10.39	5.8	0.76
1-2.c25343/1/4701.	NADH dehydrogenase subun	146149047	YP_00116545	0	0	0	2.41	15.13	109.38	0.03	130.9	114.66	7.83	151.04	7.27
1-2.c25645/1/5760.	PREDICTED: 26S protease r	768377728	XP_01158642	5.44	0	0	0	18.97	12.23	34.35	22.43	5.13	44.84	124.44	75.39
1-2.c25714/1/1398	PREDICTED: ubiquitin-prot	768369251	XP_01158196	6.03	2.73	2.28	1.37	0	0	0	0	0	0	0	0
1-2.c26009/1/1234	dermatopontin precursor [Xe	71896109	NP_00102559	1.09	3.8	4.29	0.33	1.3	2.82	0.73	0.91	0.8	0	0	0
1-2.c26194/1/1655	PREDICTED: atrial natriuret	512816528	XP_00491126	3.54	23.62	3.47	2.41	2.23	4.88	1.12	2.29	4.5	0.73	0.62	0.61
1-2.c26226/1/1835	PREDICTED: mortality facto	847109511	XP_01281436	0	0	0	0	0	0.34	3.44	0	0	7.29	6.43	0
1-2.c26261/1/1853	PREDICTED: eukaryotic trai	821476296	XP_01240160	0	6.81	1.73	0.56	0	0	1.2	4.11	0	0	0	0
1-2.c27359/4/1544	PREDICTED: hydroxyacid-o	847134641	XP_01281992	1.9	0.73	2.64	0	2.3	1.55	0.09	1.95	0	0	0	0
1-2.c28446/1/1507	--	--	--	3.8	0	0	2.31	4.75	1.86	4.41	2.83	5.16	23.27	38.34	26.18
1-2.c28556/1/1698	--	--	--	0	9.79	4.65	2.09	0	0	0	0	0	0	0	0
1-2.c28765/1/1262	--	--	--	3.25	0	0	0	4.7	9.85	9	5.31	4.99	34.85	31.86	24.57
1-2.c29523/1/3650.	PREDICTED: engulfment an	847168078	XP_01282713	0	1.41	0.35	5.25	0	1.26	0	0.43	3.71	15.68	11.48	11.06
1-2.c29797/1/1694	--	--	--	0	0	0	3.69	0	0	0.57	0	0.22	0	6.25	10.36
1-2.c29852/1/1728	unnamed protein product [On	642108707	CDQ72316.1	2.23	0	0.78	2.96	8.14	29.87	18.32	6.47	20.15	65.16	64.51	52.41
1-2.c30108/1/1128	Y-box-binding protein 3 [Xe	62751871	NP_00101585	0	0	0	0	7.73	114.05	119.72	71.14	33.53	0	120.64	0

1-2.c30194/1/1610	PREDICTED: U2 small nucl	301617734	XP_0029383(	0.18	3.82	1.94	2.41	0	0	0	0	0.98	0	0	0
1-2.c30199/1/1680	PREDICTED: glycylpeptide	301612194	XP_0029355(	9.74	0	4.08	0	0	0	0	0	0	0	0	0
1-2.c30266/1/1483	--	--	--	0	0	0	4.02	1.18	1.64	14.79	5.23	4.8	9.51	5.27	0.28
1-2.c30311/1/1646.	PREDICTED: creatine kinase	126344225	XP_00136422	640.51	302.77	58.61	7776.97	8647.92	4826.56	717.59	31.21	10004.96	9545.14	7987.52	13.59
1-2.c30417/2/1610	--	--	--	0	0.08	0	0.87	0.72	0	0	0	1.51	14.43	21.88	2.38
1-2.c30861/1/1442	clusterin preproprotein [Bufo	393717589	AFN21430.1	0.58	0	0.9	3.44	5.07	1.19	18.42	0.28	2.11	17.48	17.68	25.01
1-2.c30862/1/1528	PREDICTED: sushi, von Wil	847092934	XP_01280886	7.07	23.94	5	4.96	3.86	9.66	1.85	5.04	6.01	0.73	0.79	1.31
1-2.c31022/1/1501	--	--	--	0.19	0	0	0	0	2.45	1.71	0.24	1.29	5.28	9.64	3.19
1-2.c31270/2/1503	beta-secretase 2 precursor [X	213982845	NP_00113559	0	0	0	5.77	0.48	1.63	4.15	3.17	0.24	6.41	8.05	7.23
1-2.c31468/1/1716	--	--	--	0	1.71	0.08	0.93	1.6	1.07	6.67	1.87	2.55	22.32	15.24	19.21
1-2.c32549/2/1578	aldose reductase [Xenopus (S	58331962	NP_00101113	0	7.07	4.14	0	0	0	0	0	0	0	0	0
1-2.c32622/4/1245	PREDICTED: clathrin heavy	803113303	XP_0120406(	3.46	0	3.95	13.86	0.64	17.12	29.06	15.97	9.12	116.99	145.59	68.59
1-2.c33542/1/5773.	60S ribosomal protein L3 [X	187607061	NP_00112007	24.04	0	0	43.44	0	49.61	323.77	110.9	169.18	442.07	954.65	318.07
1-2.c33808/1/1498	exportin-7-B [Xenopus laevis	148234429	NP_00108934	0	0	0.05	3.83	0	0.34	5.77	0.73	1.06	12.82	15.54	8.81
1-2.c34613/1/1531.	tubulin folding cofactor A [X	148229733	NP_00108536	0	0.13	0	11.05	0	0	4.23	16.63	3.98	37.7	41.46	10.19
1-2.c34868/1/1336	--	--	--	8.91	0	0	27.32	0	1.45	48.51	34.74	58.52	227.69	455.94	89.25
1-2.c34956/1/1639	--	--	--	0	0	0	17.59	0	0.82	7.62	0	6.98	40.46	10.19	0.62
1-2.c35050/2/1772	PREDICTED: inositol-tetraki	847154713	XP_01282392	2.19	5.37	0	2.01	0	0	0	3.13	0	0	0	0
1-2.c35382/2/1625	--	--	--	0	0	0	0	0	0.33	2.13	0	0	3.85	4.75	0
1-2.c35508/1/1439	transposase [Salmo salar]	157285978	ABV31710.1	0	0	0	1.02	0	0	1.17	0	0	7.69	4.91	0
1-2.c35759/1/3663.	deoxyribonuclease II, lysosor	147904975	NP_00108667	0	0.4	0	1.6	0.58	0.36	1.15	0	2.56	13.69	20.29	11.68
1-2.c36312/1/1263	extended synaptotagmin-3 [X	58332580	NP_00101136	0	0	0	0.19	0	0	1.9	0.74	1.11	6	9.36	0.58
1-2.c36321/1/1256	--	--	--	0.29	0	0	0.9	0.28	1.43	5.42	2.96	0	7.08	9.13	6.91
1-2.c36512/1/1490	coatomer protein complex, su	148235681	NP_00108357	1.15	0	4.42	4.46	0.87	7.08	2.64	8.64	5.27	43.59	58.98	36.61
1-2.c36592/1/1195	PREDICTED: heterogeneous	847118781	XP_01281673	2.46	0	0	3.46	0	1.49	8.31	3.08	0	32.09	18.7	12.95
1-2.c36865/1/1469	UBA-like domain-containing	58331988	NP_00101114	0	11.75	3.55	3.05	0	0	0	0	0	0	0	0
1-2.c37145/5/1652	PREDICTED: fragile X ment	637372583	XP_00322991	154.07	146.23	153.56	118.83	0	1.5	0.34	126.12	0.06	0	0	0
1-2.c37965/1/5025.	uncharacterized protein LOC	89886093	NP_988922.2	0	0	0	4.97	0	2.88	5.19	9.86	7.79	33.28	66.62	11.92
1-2.c38197/1/1445	Vasculin [Tupaia chinensis]	444725091	ELW65670.1	0	0	0	0	0.78	0.45	4.22	2.27	5.52	9.71	7.23	5.12
1-2.c38283/1/1458	PREDICTED: insulin recepto	301615358	XP_00293713	0	10.53	2.85	2.25	0.22	0.12	1.4	0	0	0	0	0
1-2.c38353/2/1557	NADH dehydrogenase subun	146149047	YP_00116545	0	204.54	25.42	0	0	0	0.86	0	0	0	0	0.16
1-2.c38552/1/3604.	PREDICTED: creatine kinase	532105376	XP_00533827	0	0	0	6938.98	6970.2	3900.09	0	0	6148.8	6457.75	7620.4	0
1-2.c38635/1/1503	--	--	--	0	1.91	0.4	0.75	38.91	1.13	50.34	40.67	0.54	29	56.03	38.4
1-2.c38664/1/1698	elongation of very long chain	148235901	NP_00108037	0	0	0	5.87	4.11	1.67	4.29	0	4.04	12.87	31.15	4.92
1-2.c38716/1/1772	PREDICTED: myomegalin-li	591373231	XP_00706131	0	0.01	0	7.45	0	0	11.39	0.04	14.92	0	0	2.43
1-2.c38746/1/1248	PREDICTED: protein polybr	594652736	XP_00717481	0	0	0	0	0.29	0	0.78	0.67	0.07	2.48	4.68	6.61
1-2.c38760/1/7715.	--	--	--	5.89	0	0	10.1	3.71	5.08	25.33	15.73	12.93	109.19	213.01	49.52
1-2.c38766/1/1595	sorting nexin-30 [Xenopus la	148227022	NP_00108952	0	0	0	0	2.56	2.02	0.19	2.27	4.71	4.42	0	8.16
1-2.c39024/1/1701	PREDICTED: integral memb	514711338	XP_00501156	1.84	0	0	3.86	0.79	3.54	4.26	6.37	2.4	20.28	14.62	19.89
1-2.c39406/1/1345	PREDICTED: LOW QUALI	696993200	XP_00956364	0	0	0	4.13	0	0	0	0	5.53	17.17	18.37	3.35
1-2.c39407/1/5416.	PREDICTED: zinc finger pro	847116590	XP_01281596	7.26	12.12	5.23	1.98	6.95	2.41	0.32	0	0.11	0	0	0
1-2.c39929/1/1297	PREDICTED: non-syndromic	847139928	XP_01282103	0	0	0	60.26	0	0	69.67	0	55.38	0	145.21	50.43
1-2.c39959/1/1174	--	--	--	10.49	19.02	0	1.36	1.11	0	0.9	0.03	0	0	0	0
1-2.c40139/1/1298	oxidative-stress responsive 1	148228567	NP_00108693	0.01	0	0	4.92	0	0	8.14	0	2.46	22.23	31.26	6.52
1-2.c40217/1/1423	PREDICTED: myosin-4 [Xe	847156920	XP_0128244(	0	0	0	0	0	0.55	21.82	0	0	13.69	0	25.98
1-2.c40396/1/1396	serine/threonine-protein phos	58332004	NP_00101115	2.07	0	2.4	5.6	3.61	1.65	6.92	1.88	4.73	36.79	46.05	20.19
1-2.c40418/1/1465	--	--	--	1.35	0	0	1.7	0	2.55	3.96	1.8	7.52	15.06	19.71	13.38
1-2.c40426/1/1602.	developmentally regulated G	89269049	CAJ81597.1	0	0	0	2.5	0	0	14.97	2.74	0	29.83	52.84	7.76
1-2.c40458/1/3483.	eukaryotic translation initiatio	45360615	NP_988980.1	0	0	0	7.67	0	13.8	19.24	9.68	18.29	53.62	133.04	32.91
1-2.c40803/2/1179	bhmt protein [Xenopus (Silur	58618891	AAH89235.1	0	0	0	2.7	0	0	1.02	0	5.62	19.97	14.62	10.43
1-2.c41257/1/1743	cytosol aminopeptidase [Xen	58331950	NP_00101112	3.95	11.46	1.29	0	0.94	5.83	0	4.64	0	0	0	0
1-2.c41649/1/1198	PREDICTED: filamin-A isof	637264717	XP_00810209	59.89	35.83	37.79	121.3	51.07	51.45	252.81	67.04	91.67	781.12	1269.13	623.49
1-2.c41668/1/1292	MHC class I antigen [Rana pi	6090957	AAF03409.1	5.82	2.1	1.41	2.9	0	0	0	1.13	0	0	0	0
1-2.c42987/1/1355	PREDICTED: phosphatidylir	301607217	XP_0029332(	0.01	0	0	0	0.83	0	0.48	0	1.57	4.49	9.15	2.7
1-2.c43180/1/1416	splicing factor 3A subunit 3 [	62859201	NP_00101711	1.09	19.69	3.4	0	3.81	1.65	0	0	0	0	0	0
1-2.c43595/1/1680	PREDICTED: protein TFG is	847099504	XP_01281226	0.07	0	0	0	5.12	4.14	1.61	3.87	3.12	3	0	0
1-2.c44170/2/1801.	Three prime repair exonuclea	226372920	ACO52085.1	0	0	0	0	3.72	0	1.62	4.79	3.83	0	0	21.14
1-2.c44192/1/1786	Na-K-2Cl cotransporter 1 [X	147904042	NP_00109133	0	0	0	0	0.65	0.17	3.9	0.4	0.28	10.51	3.72	27.65

1-2.c44229/1/1777.	PREDICTED: hyaluronidase-	525010756	XP_00505300	10.25	1.79	2.85	6.8	11.27	1.19	17.01	5.44	8.54	62.43	50.32	162.46
1-2.c44441/1/1501	YTH domain-containing fam	45361633	NP_989392.1	0	0	0	0	0	0.94	2.83	0	0	14.26	30.98	0
1-2.c45680/1/1480	PREDICTED: isoprenoid syn	847135289	XP_01282013	6.97	5.69	3.28	1.99	0	2.87	2.38	1.32	0	0.76	0	0
1-2.c45772/1/1388	PREDICTED: catenin delta-1	847116258	XP_01281585	3.91	8.53	3.29	11.08	11.55	6.19	30.65	3.85	16.03	89.72	112.59	77.27
1-2.c45907/1/1245	PREDICTED: F-actin-cappin	733918801	XP_01072126	0	0	0	22.86	3.16	25.93	34.61	23.18	43.1	129.94	135.27	19.83
1-2.c46482/1/1381	PREDICTED: max dimerizat	847110099	XP_01281456	0	0	0	0.95	0	2.02	0	0	1.61	11.86	13.77	0.48
1-2.c46592/1/1839.	PREDICTED: collagen alpha	847135925	XP_01282037	0	0	0	0	0	618.47	48.94	40.77	46.28	60.64	39.94	46.44
1-2.c46701/2/1318	heat shock protein 90kDa alp	62858821	NP_00101628	0	0.46	0.77	3.17	0	8.97	11.26	0.42	2.71	30.81	12.56	43.03
1-2.c46780/1/1502	glycine amidinotransferase (L	89272889	CAJ82904.1	0.74	15.3	4.58	0	0.88	21.7	0	1.77	1.13	0	0	0
1-2.c46829/2/1770	aconitase 2, mitochondrial [X	147904130	NP_00108626	0.01	0	0	0	0	0.22	0.49	0	0	8.92	0	5.51
1-2.c47081/2/1303	platelet-derived growth factor	71896169	NP_00102558	0	0	0	4.66	0	0	2.73	2.15	2	8.24	2.4	3.75
1-2.c47384/2/1221	uncharacterized protein LOC	89886093	NP_988922.2	0	0	0	2.04	0	0	3.51	3.96	2.97	11.43	18.62	13.68
1-2.c47456/2/1214	PREDICTED: ATP synthase	557324504	XP_00603528	0	0	0	44.35	0	0	101.36	10.53	71.37	113.21	126.96	130.89
1-2.c47899/5/1554	PREDICTED: ras-related pro	73968729	XP_851250.1	0	0	0	3.75	0	0	0	0	5.23	19.5	18.23	14.91
1-2.c48260/1/1383	--	--	--	2.12	0	0	4.7	12.78	9.45	20.42	0.89	15.97	25.73	42.97	18.94
1-2.c48323/1/1761	PREDICTED: leucine-rich re	847134249	XP_01281979	0	0	0.01	1.03	2.6	0.58	2.27	0.41	3.23	4.93	3.25	9.14
1-2.c48994/1/1754	--	--	--	0	0	0	0	17.18	30.04	28.1	16.19	18.17	0	45.09	0
1-2.c49346/1/1508	heterogeneous nuclear ribonu	45361501	NP_989327.1	10	38.48	4.72	11.33	7.2	2.88	0.55	1.33	8.04	0	0	3.69
1-2.c49385/1/1794	protein MON2 homolog [Xer	148222438	NP_00108578	0	0	0	1.03	0	0	0.9	3.11	1.49	1.03	0	0
1-2.c49487/1/1590	Tubulin alpha-1C chain [Lari	808859777	KKF11870.1	3.28	0.93	1.65	12.41	4.28	0.46	21.31	2.49	2.38	72.21	13.97	56.8
1-2.c49530/1/1152.	inositol monophosphatase 3 [	147899181	NP_00108451	0	0	0	0.43	0	0.65	0	0	1.78	36.13	47.55	0
1-2.c49532/1/1231	growth hormone inducible tra	148231426	NP_00108009	0	0	0	5.56	0	0	11.03	0.07	5.53	5.5	3	5.25
1-2.c49644/1/1803	uncharacterized protein LOC	148234787	NP_00109077	2.46	3.02	1.63	1.54	0	0.05	0.12	1.14	0	0	0	0
1-2.c49682/1/1278	--	--	--	0	5.81	2.59	0.03	2.63	31.36	1.21	0	1.37	0	0	0
1-2.c49930/1/1754	protein PBDC1 [Xenopus (Si	62858545	NP_00101636	0.79	12.56	1.28	0.28	0.23	0	0	0.01	0	0	0	0
1-2.c50028/1/1631	PREDICTED: STAM-bindin	847108531	XP_01281402	0	0	0	2.67	0	0	3.24	0	1.46	4.74	5.07	0
1-2.c50082/1/1656	PREDICTED: poly(rC)-bindi	847099224	XP_01281215	51.32	41.81	0	0	0	0	1.3	3.72	0	0	0	0
1-2.c50137/1/1939	--	--	--	0	0	0	0.07	0.06	0	0.04	0.18	0	3.48	4.06	1.34
1-2.c50171/1/1882.	PREDICTED: filamin-A-inte	301613504	XP_00293624	2.39	1.68	1.38	0.42	0.56	3.18	0.4	2.07	0	0	0	0
1-2.c50280/1/1366	--	--	--	2.21	1.65	0	1.87	1.55	1.96	5.42	4.17	7.03	28.34	18.16	28.83
1-2.c50528/1/1412	PREDICTED: semaphorin-3I	612053402	XP_00750416	14.69	83.41	15.08	12.34	9.09	13.5	4.59	8.19	16.84	4.71	1.86	1.81
1-2.c50594/1/1535	Ras suppressor protein 1, part	677553639	KFR13401.1	0	0.83	0	0	14.26	21.54	0	0	15.1	1.07	33.67	33.68
1-2.c50644/1/1355	--	--	--	6.42	30.82	13.35	4.79	3.48	38.51	1.25	5.34	3.49	0	1.19	1.32
1-2.c50942/4/1445	Complement factor B [Xenop	213625177	AAI69984.1	0	0	0.16	0	3.43	7.51	0.14	0	8.4	9.3	12.82	2.06
1-2.c50953/9/1302	PREDICTED: protein disulfi	697460798	XP_00966706	0	0	0	47.04	0.65	0	18.05	40.22	47.53	136.44	78.63	39.79
1-2.c51067/2/1543	Unknown (protein for IMAG	189442732	AAI67693.1	0	0	0	0.82	6.53	8.67	0	2	0.42	0	0	21.16
1-2.c51308/10/1472	PREDICTED: myosin-4 [Xer	847156920	XP_01282446	0	0	0	9.08	41.76	0	1.55	125.94	216.2	19.73	7.33	0
1-2.c51922/5/1347	keratin, type I cytoskeletal 18	148234658	NP_00108081	0	8.13	0	35.1	0	0	50.74	0	49.03	238.84	532.89	164.26
1-2.c52054/3/1322	lactate dehydrogenase A [Xer	148227690	NP_00108070	37.1	0	0	620.24	0	0	267.39	0	507.04	281.13	228.11	153.17
1-2.c52185/9/1457	uncharacterized protein LOC	147905311	NP_00109060	0	0	1.66	4.54	2.99	2.24	6.91	2.85	0.91	25.39	14.34	34.65
1-2.c52328/2/1324	RING finger protein 10 [Xen	148231949	NP_00108983	0	0	0	5.14	0	0	1.89	1.31	0	22.47	39.1	0
1-2.c52366/8/1595	PREDICTED: tubulin beta-5	558136461	XP_00611755	2.47	34.12	5.75	0.59	0	4.23	0	0	0	0	2.89	3.48
1-2.c52455/2/1393	Keratin, type II cytoskeletal c	676246754	KFO10626.1	0	0	0	87.94	0	0	0	76.12	61.55	94.44	46.18	0.01
2-3.c1310/75/2380.	Copz1 protein, partial [Xenop	29126980	AAH47988.1	2.1	4.81	1.6	0	0	0	0	0	0	0	0	0
2-3.c1485/31/2623	nuclear pore complex protein	47498074	NP_998879.1	5.35	21.5	2.3	2.37	3.97	9.52	1.62	2.71	1.92	0.77	0.38	1.09
2-3.c1543/8/2309	PREDICTED: serine/threonir	821122370	XP_01238372	0	0	0	3.61	0	0	4.16	0.15	3.85	3.5	0	0
2-3.c1606/43/2098	heat shock 70kDa protein 9 (r	148228693	NP_00108016	6.18	7.78	0	0	11.51	1.56	0	0	0	0	0	0
2-3.c1632/54/2057	heterogeneous nuclear ribonu	45361501	NP_989327.1	13.58	25.51	12.68	0	10.86	0	0.23	4.45	0	0	0	0
2-3.c1880/8/2155	protein tyrosine phosphatase	56605916	NP_00100846	0	0	0	0	8.76	12.89	0	7.31	0	5.65	3.97	0
2-3.c1994/19/2575	probable lysosomal cobalami	118403984	NP_00107218	0	0	0	0	4.51	1.9	0	3.32	0	4.75	4.99	0
2-3.c2145/23/2018	PREDICTED: pre-mRNA 3'-	847088059	XP_01281946	10.02	13.75	3.94	3.41	0.55	0.21	3.01	0.89	0	0	0	1.42
2-3.c2790/3/2291.1	uromodulin-like protein type	802176571	BAR42254.1	1.65	0	1.31	2.71	3.83	0	74.38	1.53	0	42.96	13.53	84.52
2-3.c3209/1/2397.1	NPL4 homolog, ubiquitin rec	147898562	NP_00108042	18.77	0	7.64	1.08	13.63	1.22	0	3.15	0.83	0	0	0
2-3.c3339/1/2486	PREDICTED: myocilin [Xen	512836045	XP_00293419	0	0	0.23	0	14.28	0	13.99	15.06	0	2.22	10.5	11.56
2-3.c3631/1/1939	--	--	--	14.86	5.97	18.01	21.88	0	0	0	1.44	0	0	0	0
2-3.c4256/1/2408	--	--	--	0	2.87	1.53	2.5	1.18	0	0	2.61	0	0	0	0
2-3.c5010/1/2465.1	von Willebrand factor precurs	372273431	NP_00124321	0	0	0	0.16	0	0	0	0.53	0.41	2.37	5.26	2.77
2-3.c5642/2/2090	glutamyl-prolyl-tRNA synthe	189217812	NP_00112134	6.66	10.83	10.61	8.39	0.01	20.11	0.08	16.64	18.21	2.4	0	0

2-3.c6388/1/2165.1	mitochondrial ribosomal prot	148236950	NP_00107977	2.76	10.04	6.27	4.15	0.93	3.41	0	0	3.23	0	0	0
2-3.c6414/1/2370	--	--	--	7.76	27.99	3.9	5.07	3.86	54.69	1.51	2.82	3.55	0.54	1.35	1.52
2-3.c6552/3/2122	PREDICTED: A-kinase anch	512820714	XP_00293685	4.17	6.01	0.9	0.03	0	0	0	4.73	0	0	0	1.13
2-3.c6723/3/2362	splicing factor proline/glutam	189230206	NP_00112142	15.35	23.33	8.79	0	0	0	2.7	0	0	0.01	1.72	0.28
2-3.c6821/1/2213	lysyl oxidase homolog 1 prec	156717522	NP_0010963(	1.91	7.47	2.3	0	0.7	0.04	0	0.62	0.08	0	0.01	0.02
2-3.c7532/1/1940	PREDICTED: nebulin isofor	847166508	XP_01282661	57.87	34.67	42.49	5.62	107.39	0.77	114.58	51.98	16.33	3.15	4.58	9.37
2-3.c7552/1/2102.1	protein archease [Xenopus (S	62857601	NP_00101679	1.92	1.96	1.07	1.25	3.65	0	0	0.81	0	0	0	0
2-3.c7590/1/1927	PREDICTED: CXXC-type zi	847088379	XP_01282033	1.89	5.27	1.36	0	1.14	0.74	0.55	0.64	0.3	0.23	0	0
2-3.c7867/2/2274	PREDICTED: F-box only pro	543358887	XP_00552332	0	0	0	0	2.8	1.74	2.66	1.83	4.52	1	2.02	6.42
2-3.c9076/1/2475.2	--	--	--	0	0	0	0	1.64	0.74	0	3.63	0	13.98	67.64	12.98
2-3.c9310/4/2058	PREDICTED: guanine nucle	560925767	XP_0061890(	2.96	1.66	1.47	0	0.24	0.66	0	0	0	0.53	0	4.2
2-3.c9385/1/2521.2	PREDICTED: D-beta-hydrox	301616891	XP_00293787	2.69	13.27	6.03	2.55	2.54	6.17	3.96	4.05	2.06	0	0	0.39
2-3.c9995/2/2333	--	--	--	0	0	0	0.68	0.46	0	3.26	0.78	0	9.38	7.45	1
2-3.c10008/1/1996.	ethanolamine-phosphate phos	147905107	NP_00108663	1.24	0.36	1.81	4.14	10.7	4.45	20.7	6.4	1.14	40.79	31.14	65.5
2-3.c10257/1/2247	protein phosphatase 2, regula	148234757	NP_00108413	0	0	0	1.53	0	0	0.56	0	0.75	2.93	3.69	0
2-3.c10445/16/2077	zinc finger protein 36, C3H1	147901333	NP_00108188	0	37.89	8.47	0	4.67	0	0	0	0	0	0	0
2-3.c10508/2/2043.:	--	--	--	12.35	0	0.79	21.27	19.98	2.72	238.64	10.77	4.2	695.42	82.99	1276.27
2-3.c11803/12/2529	angiopoietin-related protein 7	156717416	NP_00109624	1.79	17.53	8.13	0.65	1.98	28.55	0.26	1.23	1.97	0	0.39	0.57
2-3.c11954/1/2403	--	--	--	1.17	6.12	0.42	1.44	0.17	0	0	0.68	0	0	0	0
2-3.c11958/6/2586	low-density lipoprotein recep	156717580	NP_00109633	0.53	4.46	2.67	0	0	1.27	0.69	0	0.11	0	0	0
2-3.c12055/5/2190	collagen, type 1, alpha 2 prec	147898763	NP_00108072	30.08	269.22	94.54	38.67	55.43	827.66	0	0	0	0	0	4.66
2-3.c12206/1/2066	PREDICTED: nuclear recepto	512881896	XP_00294049	8.89	55.22	22.85	5.31	46.31	10.52	0	24.49	3.62	1.61	1.04	5.22
2-3.c12215/1/2021.	--	--	--	29.21	11.47	13.94	19.97	47.42	6.96	81.74	62.8	53.54	268.3	133.18	433.03
2-3.c12436/4/2491	RecName: Full=Voltage-depe	46576355	O57483.1	0	0	0	0	0	1.07	0	4.02	6.36	4.69	1.75	0
2-3.c12507/1/2168	--	--	--	0	0	0	1.22	0	0	1.56	0.8	1.65	3.3	1.4	0
2-3.c12567/1/2031	PREDICTED: CTP synthase	512819329	XP_0029394(	0.56	4.57	1.52	0	0.42	5.82	0	0	0	0	0	0
2-3.c12758/1/2311	PREDICTED: interleukin enh	847169995	XP_01280854	25.47	81.46	20.45	8.14	10.64	23.77	13.86	9.61	4.84	0	7.47	6.07
2-3.c12765/1/2745	PREDICTED: semaphorin-3I	395539080	XP_0037715(	1.71	13.69	2.76	1.52	2.27	0.84	0	0.25	0	0	0.62	0
2-3.c12821/1/2495.	PREDICTED: hydroxysteroid	847099649	XP_01281231	0.46	2.81	2.58	2.77	5.14	6	10.19	1.34	3.05	65.4	29.77	141.76
2-3.c13263/2/1862	PREDICTED: stromal intera	686589298	XP_00928915	6.41	8.42	0	6.52	14.97	13.16	3	15.51	15.19	50.02	88.71	68.46
2-3.c13360/1/1950.	septin 6 [Xenopus laevis]>gi|	148230675	NP_00108704	0.2	0	0	0	0	0.82	0	0.61	0	4.7	5.81	6.66
2-3.c13546/1/2174.	PREDICTED: 28S ribosomal	847132351	XP_01281932	5.06	6.89	4.7	1.54	6.87	0	2.65	0	0	0	0	0.45
2-3.c13686/1/2392	LOC733424 protein [Xenopu	80476880	AAI08821.1	0	0	0	0	0	0.47	1.68	0.21	3.06	2.67	3.76	2.29
2-3.c13758/1/2368	PREDICTED: immunoglobul	847130950	XP_01281911	2.21	5.63	3.87	0.27	1.02	2.79	0	1.53	0.92	0.09	0	0
2-3.c13887/2/2221.	upf3b protein [Xenopus (Silu	165971526	AAI58354.1	2.39	0	1.94	0.44	3.37	0.8	0	0	0	0	0	0
2-3.c14169/2/2285	PREDICTED: junctophilin-2	301607262	XP_00293323	0	0	0	2.74	0	2.55	6.73	0.57	9.42	0	6.95	0
2-3.c14596/3/2272	PREDICTED: laminin subun	847130567	XP_01281902	1.84	0	1.2	0.15	0.62	6.08	0	0	0	0	0	0
2-3.c14617/1/2506	--	--	--	2.29	16.36	3	0	1.54	8.87	0	2.79	1.73	1.34	0	0
2-3.c14945/1/2586	PREDICTED: LOW QUALI'	831302925	XP_01268353	1.1	1.99	2.47	0.94	0	0	0	0	0	0	0	0
2-3.c15293/2/2023	phospholipid-transporting AT	71896237	NP_0010255(	0	0	0	0.46	0	0	0.67	0.55	0	1.9	0.91	3.81
2-3.c15345/1/2164.	regulator of G-protein signali	291290887	NP_00116747	0.05	0	0	1.02	0	0	3.22	0.22	0	10.54	16.55	0
2-3.c15590/1/1982	alpha1 type II collagen [Cync	5360532	BAA82043.1	2.37	42.67	10.69	5.98	0	9.68	2.62	7.47	1.36	1.17	0	0
2-3.c15840/1/2166.:	--	--	--	9.26	7.55	1.64	0	4.59	0	0	0.56	3.64	0	0	0
2-3.c15966/1/2331.	ubiquitin-like modifier-activa	148225200	NP_00108972	0	0	0	0.03	0	0	1.54	0	0	0	7.38	6.54
2-3.c16436/1/1810.	vacuolar proton-ATPase E-su	111924355	BAF02791.1	3.76	3.42	6.38	0	9.56	1.33	0	3.47	0	0	0	0
2-3.c16565/1/2108.	--	--	--	0	17.32	3.26	0	2.41	0	0	0	0	0	0	0
2-3.c16573/4/2106.	PREDICTED: 5-azacytidine-	512848342	XP_00491543	2.27	7.1	4.91	2.26	5.4	0.11	0	0	5.55	0	0	0
2-3.c16756/3/2049.	syntaxin-binding protein 6 [X	54020954	NP_00100571	1.04	7.25	1.82	0.57	0.23	0	0	0.53	1.99	0	0	0
2-3.c16848/2/2264	PREDICTED: tetratricopepti	530607046	XP_00529521	0	0	0	0.5	4.04	0.91	0	3.83	3.62	0.97	0	1.57
2-3.c16911/1/2137.	PREDICTED: band 4.1-like p	847168906	XP_0128082(	5.96	0	2.97	2.13	0.06	2.1	0	2.08	0	0	0	0
2-3.c16950/2/2118	PREDICTED: large subunit (	301616900	XP_00293788	4.28	6.94	2.4	0	0	0.07	3.22	0	2.28	0	0	1.45
2-3.c16955/1/1968	PREDICTED: homeobox pro	847152600	XP_01282325	2.44	2.27	2.81	0.91	0	0	0	0	0	1.79	0	0
2-3.c17248/1/2116	PREDICTED: NADPH-depe	874459990	XP_01295045	1.69	2.6	1.2	0.83	0	0.91	0.65	0	0.17	0	0	0
2-3.c17604/1/2593.	claudin-1 [Xenopus (Silurana	62751357	NP_0010157(	1.49	14.9	3.44	0	0	0	0	0	0	0	7.64	0
2-3.c17631/1/2468	kinesin-like protein KIF2C [X	147904322	NP_00108031	1.43	7.71	2.88	0.37	1.41	3.09	0.88	0.84	1.51	0	0.25	0.06
2-3.c17684/3/2163	--	--	--	7.19	12.69	0	1.68	0	0	0	0	0	0	0	9.97
2-3.c17879/6/2441	PREDICTED: serine/threonir	512866570	XP_0029399(	2.5	11.05	3.83	1.56	1.93	18.67	3.08	0.56	1.56	0.41	0.2	0.25
2-3.c17883/2/2474.	--	--	--	2.73	3.59	2.35	0	0	0	1.8	0	0	0	0	0

2-3.c17958/4/2526	hypothetical protein MGC75	38174052	AAH61314.1	0.12	2.38	1.26	0	0.35	0.14	0.68	0	0.86	0	0	0
2-3.c17991/3/2208	dual specificity mitogen-activ	56118586	NP_00100805	0	0	0	20.58	0	0	14.62	14.99	20.55	11.53	3.69	6.26
2-3.c18641/3/2291	PREDICTED: protein sel-1 h	301618001	XP_00293841	0	0	0.16	0.79	0.67	0	0.9	0.17	1.93	8.33	1.93	5.59
2-3.c18673/4/2352	PREDICTED: guanine nucle	395821567	XP_0037841(	0.97	6.42	1.43	0.21	2.01	0	0	0	0	0	0	0
2-3.c18991/1/1982	PREDICTED: filamin-A-inte	301613504	XP_00293624	8.54	5.38	3.46	1.15	2.16	7.85	0.75	5.97	1.59	0.56	0.58	0.28
2-3.c19040/1/2287	(XCAP-C) [Xenopus laevis]	213626071	AAI70550.1	0.92	5.71	1.32	0	0.45	1.6	0	0	0.94	0	0	0
2-3.c19080/1/2229	PREDICTED: probable ubiq	558212499	XP_00613401	4.71	11.9	1.55	0	1.77	0	0	0.91	0	0	0	0.6
2-3.c19571/1/2065.	--	--	--	2.97	0.41	0	23.26	40.1	13.31	25.85	25.22	57.04	83.11	94.08	11.25
2-3.c19638/1/1991.	PREDICTED: calponin-1 [Tr	471381154	XP_00437837	0	0	0	0	0	1.58	0	0	6.6	4.03	15.25	8.94
2-3.c19890/1/2237	--	--	--	1.48	2.34	1.84	0.64	0.26	0	0	0	0	1.16	0	0.64
2-3.c20127/2/2026	PREDICTED: tetratricopepti	620948097	XP_00765381	0	0	0	0	0	1.7	0	0	3.18	2.56	1.39	2.17
2-3.c20238/3/2197.	Unknown (protein for MGC:	120538440	AAI29682.1	0	3	0	1.21	2.97	0.45	0	8.46	0	21.47	34.46	33.82
2-3.c20444/1/2184	probable lysosomal cobalami	118403984	NP_00107218	0	0	0	0	2.53	0.75	0	1.14	0	4.39	4.04	0
2-3.c20888/1/2302.	Complement factor B [Xenop	213626825	AAI70192.1	0	0	0.12	0	12.21	11.89	0.05	0.1	5.89	35.42	45.16	5.59
2-3.c20942/1/2065	uncharacterized protein LOC	58332520	NP_00101133	2.77	5.34	1.48	0	0	1.33	0.44	0.77	0	0	0	0
2-3.c20966/1/2286.	--	--	--	0	0	0	0	0.37	0.53	7.23	0	0.97	17.42	34.63	5.35
2-3.c21088/1/2549	periostin precursor [Xenopus	163915007	NP_00110637	1.7	41.01	7.44	0	3.34	75.99	1.79	0.73	9.14	0.08	0	0
2-3.c21133/1/2588	PREDICTED: synaptopodin	301612611	XP_00293581	2.28	13.7	2.42	1.72	1.29	15.98	0.11	1.2	0.14	0	0	0.11
2-3.c21471/1/2157.	--	--	--	1.13	1.2	2.53	0	0.21	0	0	1.98	0	0	0	0
2-3.c21589/1/1874	--	--	--	1.37	0	0	1.66	1.87	0.17	3.29	1.45	0.75	9.9	12.01	16.41
2-3.c21687/1/2129	protein unc-45 homolog B [X	297591951	NP_00117205	3.71	25.53	4.43	4.6	3.21	57.85	1.22	1.34	2.8	1.31	0.39	0.19
2-3.c21772/8/1947	TATA box-binding protein-a	148225402	NP_00107945	2.27	0	1.73	0.66	0	1.26	0.35	0	1.04	0	0	0
2-3.c21804/1/2220.	PREDICTED: zinc finger pro	602653218	XP_0074453(	0.12	0	0	0.43	0.46	0	1.24	0	0.63	4.09	7.27	2.35
2-3.c21859/1/2039	staphylococcal nuclease and	148234849	NP_0010796(	3.46	18.41	2.81	0	5.57	10.82	1.28	6.04	1.06	0	0	1.57
2-3.c21882/2/2312	uncharacterized protein LOC	148226767	NP_00108484	0	0	0	15.57	0	2.19	28.82	16.71	30.15	49.34	45.12	59.19
2-3.c22004/9/1913	LOC431836 protein, partial [	62739321	AAH94088.1	26.67	23.19	16.23	22.3	0.7	0.26	0.91	19.52	0.43	0.38	0.42	0.47
2-3.c22347/2/2177	CDK5 regulatory subunit ass	148235437	NP_00109049	0	0.81	0	4.14	0.15	0.57	2.99	2.25	0	9.9	7.08	6.88
2-3.c22370/2/2244	PREDICTED: semaphorin-3I	395539080	XP_0037715(	2.44	13.1	3.99	0	2.8	2.66	1.15	1.55	4.08	0.01	0.61	0.44
2-3.c22509/1/2050	PREDICTED: microtubule-a	847168426	XP_01280801	4.98	3.24	1.86	0	3.09	2.1	1.13	3.21	0.54	0	0	0.13
2-3.c23100/1/2426.	PREDICTED: hydroxysteroi	847099649	XP_01281231	0.7	0.14	0.76	1.09	0.77	1.99	2.24	0.48	1.21	10.27	11.98	22.32
2-3.c23427/2/2340	eukaryotic translation initiati	156717262	NP_00109617	0.01	0	0	5.1	3.17	3.92	0.84	1.84	1.77	2.43	0	0.83
2-3.c23976/1/2487	fragile X mental retardation s	147899444	NP_00108178	0.49	0	0	13.07	11.91	35.48	0.29	18.81	0	11.68	7.97	0
2-3.c23997/2/2654	--	--	--	3.24	7.31	5.3	0.06	6.32	25.55	0.37	0.36	4.52	0.2	0	0.98
2-3.c24083/1/2474.	serine/arginine-rich splicing f	55926178	NP_00100748	0.79	18.63	5.24	0.84	2.58	4.87	0	1.41	2.27	0	0	0.27
2-3.c24098/1/2296	PREDICTED: H(+)/Cl(-) exc	847163797	XP_01282596	0.52	0	0.13	0.57	0.06	0.93	1.45	0	1.19	7.17	5.81	6.7
2-3.c24401/3/2334	PREDICTED: transcription e	847101864	XP_0128129(	8.9	0	4.84	1.64	0	0.22	0	0	0	0	3.5	0
2-3.c24516/2/2345	PREDICTED: collagen alpha	847169802	XP_01280845	0.41	4.91	1.65	0.84	0.04	2.36	0	0	0	0.66	0.03	0
2-3.c24541/3/2527	thrombospondin-4 precursor	118404528	NP_00107267	5.73	11.35	9.86	0	2.48	5.02	0	2.63	3.13	1.1	0.55	0
2-3.c25331/5/2067	--	--	--	0.77	0.8	0.65	2.74	1.93	2.4	6.25	1.92	2.39	16.76	18.87	9.31
2-3.c25387/2/1940	--	--	--	1.02	16.12	4.39	0	0.02	1.43	0	2.02	0	0	0	0
2-3.c25434/3/2326	PREDICTED: LOW QUALI	512830904	XP_00293188	8.79	5.36	3.96	0.64	0	1.79	0	0.87	0.18	0	1.58	0
2-3.c25875/2/2644	--	--	--	0	0	0.33	0.76	0	0	3.73	1.15	2.14	4.13	4.43	5.97
2-3.c26473/1/2141.	--	--	--	0.24	0.84	0.25	6	5.68	1.71	0	4.13	7.76	20.33	3.03	20.95
2-3.c26771/1/2264.	PREDICTED: collagen alpha	556973054	XP_00599461	1.66	31.16	9.43	0.49	2.2	4.97	2	1.73	4.21	0.26	0	0.07
2-3.c26883/1/2274	PREDICTED: dipeptidyl pep	847108105	XP_01281387	0	0	0	1.92	2.57	0.46	3.35	1.62	2.32	3.16	0.71	0.88
2-3.c27062/2/2124	Plaa protein [Xenopus laevis]	68534804	AAH98975.1	0	1.35	0	1.83	0	0	9.53	0	0	2.51	12.57	10.97
2-3.c27096/1/2572.	--	--	--	0	2.75	0.63	3.59	3.89	3.75	1.83	0.12	6.65	22.86	73.93	38.84
2-3.c27113/1/2210	--	--	--	0.03	0	0	5.61	0	0	68.55	0	5.68	16.15	5.68	13.04
2-3.c27248/1/2300	heterogeneous nuclear ribonu	218749891	NP_00108247	3.99	3.86	0.04	0	0	0	0	0.53	0	0	0	0
2-3.c27621/1/2398	uncharacterized protein LOC	148233247	NP_00108367	1.37	2.74	1.35	0.14	0	0.32	1.3	0	0	0	0	0
2-3.c28099/2/2252	pyruvate carboxylase, gene 1	148227386	NP_00108322	0.08	2.16	0.47	1.99	2.78	7.53	8	0.66	0.81	7.88	31.77	45.36
2-3.c28424/1/2004	PREDICTED: tight junction	512813087	XP_00491083	0.1	0	0	1.66	0	0	0.13	1.29	1.58	4.31	1.77	5.66
2-3.c28445/4/1875	chaperonin containing TCP1,	89273814	CAJ81480.1	18.93	15.31	8.79	0	6.87	5.66	4.8	2.46	1.82	0	0	3.91
2-3.c28586/1/2642	MGC64589 protein, partial [	27735407	AAH41516.1	2.37	0.82	0.84	0.31	0.22	0.18	0.49	0.33	0.84	0	0	0
2-3.c28812/3/2259.	angiopoietin-related protein 7	156717416	NP_00109624	3.03	22.21	6.66	2.03	0.68	16.35	1.29	2.14	0.59	0.1	0.12	0.91
2-3.c29286/1/2231	PREDICTED: myomegalin-li	591373231	XP_00706131	0.07	0	0.07	15.07	0	0	23.41	0	26.24	0	0	2.31
2-3.c29329/1/2325	--	--	--	0.98	0.72	0	0.57	0.65	2.53	2.88	0	1.06	17.23	15.36	6
2-3.c29517/3/2121	PREDICTED: ubiquitin carb	301614077	XP_00293651	0	0	0	1.76	0.91	0	4.69	6.56	0.83	0	0	9.83

2-3.c29549/1/2470	PREDICTED: LOW QUALI	512830904	XP_00293188	21.92	27.21	13.77	7.73	6.37	50.95	5.33	0	7.53	2.09	0	2.86
2-3.c29611/1/6284.:	--	--	--	0	5.83	3.24	177.42	123.98	64.73	74.11	4.89	168.11	103.15	442.25	94.04
2-3.c29815/1/2642.	PREDICTED: G-protein cou	512882444	XP_0049199(	0.61	1.96	0.57	0.67	13.17	6.32	7.43	23.8	20.2	12.01	13.4	0.59
2-3.c29976/2/2445	Histone H3.3 [Harpegnathos	307204446	EFN83153.1	0	0	0	1.65	3.09	2.12	0	6.15	3.71	10.26	7.3	3.19
2-3.c30252/1/2102	--	--	--	0	0.03	0	2.87	0	0	2.01	2.1	1.38	3.38	1.72	0
2-3.c30719/1/2233	transcription factor Dp-1 [Xe	148222844	NP_00108805	0.67	4.84	0.91	0.99	0.4	0.65	0.39	0	0	0	0	0
2-3.c31514/4/2364	PREDICTED: collagen alpha	847130150	XP_01281895	1.44	39.44	14.64	8.87	0	0	5.59	0	4.95	0	0	1.32
2-3.c31715/2/2199	guanine nucleotide binding p	148231283	NP_00108051	44.7	103.16	4.65	0	2.61	0	13.88	0.01	0	0	0	2.19
2-3.c31982/1/2222	Na-K-2Cl cotransporter 1 [X	147904042	NP_00109133	0	0	0.08	0.35	0.75	0	1.32	0	0.9	3.96	2.61	10.47
2-3.c32079/1/2215	PREDICTED: myomegalin-li	573893936	XP_00663471	0	0	0	1.38	0.17	0.33	3.17	0.36	1.78	0	0	0.68
2-3.c33669/1/4992.:	uncharacterized protein C17o	52345942	NP_00100501	0	0	0	0.68	0	0	0.88	0	1.87	2.1	3.79	3.3
2-3.c33715/1/2171	--	--	--	0.34	0.72	0	0	0.64	1.54	0.98	0.31	0.62	8.22	4.51	10.06
2-3.c33875/1/1894	--	--	--	0	0	0	0	0.38	2.61	3.16	0	0	5.66	8.91	0
2-3.c34018/2/1900	--	--	--	0	0	0	4.4	1.84	0	4.9	2.37	4.15	0.64	2.38	1.84
2-3.c34088/1/2108	PREDICTED: myotubularin-	847116167	XP_01281582	0.83	4.04	1.02	1.01	0	0.75	0.06	0	1.11	0	0	0
2-3.c34802/1/2277.	--	--	--	0	29.35	10.01	0.9	6.25	3.54	0	3.68	12.46	0	0	0
2-3.c35295/1/2173	--	--	--	0	0	0	2.19	2.06	3.75	1.59	1.12	3.12	3.26	9.13	16.43
2-3.c35318/1/2144	--	--	--	0	0	0	0	0.91	2.98	0	1.81	0	12.46	13.97	24.99
2-3.c35542/1/2172	--	--	--	0.06	0	0	0.06	3.56	2.78	2.06	2.4	1.64	0	1.28	0
2-3.c35788/1/2229	netrin 1 precursor [Xenopus (	118404128	NP_00107238	0.71	3.19	0.86	0.25	0	1.42	0.15	1	0	0	0	0
2-3.c35913/1/2033	--	--	--	0	0	0	0	4.61	4.13	0	4.63	0	14.79	0	17.63
2-3.c36813/3/2290	Activated RNA polymerase I	226372244	ACO51747.1	0	0	0	0	0.51	0	2.73	0	0	6.09	3.34	1.53
2-3.c37126/1/2373.	PREDICTED: RNA polymer	301607791	XP_00293347	3.14	2.88	0.2	0	3.47	0	0	0	0	0	0	0
2-3.c37162/1/2260	segment polarity protein dish	147902637	NP_00108609	7.46	6.01	4.78	0.92	2.33	0	3.11	0	0.99	0	0	1.03
2-3.c38674/2/2550	uncharacterized protein LOC	148223037	NP_00108484	0	0	0	0.96	0	0	4.87	4.61	0	0	3.58	0
2-3.c39182/2/2306	collagen alpha-1(III) chain pr	218847780	NP_00113637	0	100.53	16.19	0	0	0	0	0	0	0	0	0
2-3.c39770/1/1895.	ataxin-10 [Xenopus (Silurana	62751857	NP_00101582	5.31	8.24	1.42	0.27	1.66	2.62	0.25	0.92	0.83	0	0.19	0
2-3.c39827/1/2344	PREDICTED: transcription e	512819310	XP_0049116(	0.47	8.14	1.79	0	0.3	6.09	0.51	0.53	0.73	0	0.26	0
2-3.c40334/1/2160.	ethanolamine-phosphate phos	147905107	NP_00108663	0	0	0.68	3.42	13.33	2.75	22.64	5.61	1.88	15.37	36.26	60.49
2-3.c40397/1/2078	PREDICTED: spectrin beta c	542194400	XP_00547213	0	0.12	0	1.82	0	0	3.48	1.78	0	1.6	2.88	7.14
2-3.c40415/1/1972	phosphatidylinositol 4-kinase	148228714	NP_00108705	0.73	3.26	1.12	0.2	4.19	0.96	0	1.28	0.37	0	0	0
2-3.c40679/1/2407.	dual specificity mitogen-activ	56118586	NP_00100805	0	0	0	2.44	0	0.22	3.47	2.96	3.42	4.64	1.52	0
2-3.c40682/1/2371	PREDICTED: far upstream e	847117382	XP_01281622	1.59	2.03	0.89	0.23	1.44	0.06	0	0	0.35	0	0	0
2-3.c40754/1/2314	YTH domain family protein 1	431894607	ELK04407.1	3.89	4.67	0	2.44	1.2	0	0	0	0	0	0	0
2-3.c40857/1/1998	PREDICTED: endogenous re	831566752	XP_01273347	0	4.2	1.17	2.14	1.11	0	7.21	0	1.31	0	0	0
2-3.c41000/1/2484	--	--	--	0	0.26	0	3.87	0.34	0	2.83	2.71	2.68	2.95	1.4	0
2-3.c41305/1/2020	zinc finger protein 703-B [Xe	148223708	NP_00108444	6.74	1.17	10.91	0	3.94	0	0	0	0	0	0	0
2-3.c41514/1/2631.:	PREDICTED: telethonin [Xe	301622194	XP_00294041	57.9	18.4	61.92	0	37.97	64.19	4.2	9.7	3.27	1.56	4.38	0.43
2-3.c41584/1/2318.:	PREDICTED: mixed lineage	301604167	XP_00293171	5.26	10.67	7.55	1.38	8.03	1.79	0	6.37	2.11	0	0	0
2-3.c41735/1/1948	--	--	--	2.85	12.72	0.42	0.04	0.89	2.41	0.35	2.93	0.02	0.13	0	0
2-3.c42007/1/2299	--	--	--	0.94	14.61	5.84	0	2.11	64.62	1.35	0	0.82	0	1.25	0
2-3.c42029/1/2500	PREDICTED: protein O-man	512860787	XP_0029389(	0	0	0	0.28	0	0	0.42	2.18	1.11	0	0	3.43
2-3.c42063/1/2352	PREDICTED: elongation fac	557323742	XP_00603492	390.76	1326.95	439.07	331.79	236.61	0	55.45	68.6	297.6	0	0	209.67
2-3.c42404/1/2038	PREDICTED: serine/threonir	734601995	XP_01072793	1.07	9.14	1.67	0	0	0	0	0	2.29	0	0	2.77
2-3.c42423/1/2242	heterogeneous nuclear ribonu	89272515	CAJ81560.1	0	30.91	3.72	0	1.59	4.14	0.78	0.79	0	0	0	0
2-3.c42568/1/2167	PREDICTED: leucine-rich re	847138470	XP_01282083	0.86	2.61	1.59	0.56	1.87	0.24	2	0	0.12	0	0	0
2-3.c43109/1/2341.	PREDICTED: AP-2 complex	821005491	XP_01236599	0	0	0	1.6	0	5.51	2.75	1.17	5.92	37.02	39.41	7.03
2-3.c43548/1/2227	WD repeat and SOCS box co	148222751	NP_00109025	0	0	0	0	2.48	0.69	1.24	0	2.82	8.89	7.18	4.92
2-3.c43664/1/1854	PREDICTED: methionine an	704281225	XP_0101567(	4.08	13.61	7.84	1.83	2.92	9.15	1.96	0	0.96	0	0	0.75
2-3.c43699/1/2329	--	--	--	0	5.02	1.53	0	1.16	0	0	0	0	0	0	0
2-3.c43769/2/1884	fragile X mental retardation s	147899444	NP_00108178	0.47	0.39	0.25	15.4	21.62	61.82	0.67	16.06	12.15	13.83	14.23	0
2-3.c43935/1/2533	--	--	--	2.28	10.67	4.34	0	5.87	10.44	0	0	0.08	0	3.56	2.65
2-3.c45337/2/2129	PREDICTED: receptor-type t	704177475	XP_01013411	0.96	0	0	2.25	0	4.56	1.18	3.14	0.93	12.37	9.06	13.18
2-3.c46191/1/2155.	--	--	--	0	0	0	0	6.82	0	0	5.91	0	111.42	0	55.32
2-3.c46636/1/2250	--	--	--	0.17	7.04	2.69	0.44	0	0.88	0.46	0.78	0.69	0.09	0	0.07
2-3.c46871/1/2764	Glycogen phosphorylase, mu	465954282	EMP25833.1	0	0.76	0	4.41	8.51	1.34	46.16	4.16	44	17.39	0.07	13.83
2-3.c47242/1/2412.	PREDICTED: zinc finger prc	558191053	XP_00612944	2.84	5.26	0	1.94	0.67	0	0	0.53	1.27	0	0	0
2-3.c47351/1/2315.	PREDICTED: rab GDP disso	573874500	XP_00662563	0	7.61	1.85	0	1.21	0	0	0	0.5	0	0	0

2-3.c47496/1/2533	PREDICTED: zinc finger prc	591357490	XP_00705381	0.89	2.05	0.64	0	0	0.61	0	0.09	0.06	0	0	0
2-3.c47765/1/2613	probable ATP-dependent RN	118405026	NP_00107251	0	0	0	0.79	0	0	0.17	0	0.9	5.32	5.71	4.8
2-3.c47898/1/2398	polypyrimidine tract-binding	118404352	NP_00107247	2.7	7.01	0	1.87	2.75	0	0	1.22	0	0	0.1	0
2-3.c47969/1/2158	leucine-rich repeat-containing	45361617	NP_989386.1	0.65	0	0	1.36	0	1.09	2.12	0	0.14	7.36	8.18	9.03
2-3.c48264/1/2191	PREDICTED: fructose-bisph	591365782	XP_00705775	0	0	0	0.61	0.35	0	0.36	0.88	0.59	4.36	8.27	0
2-3.c48716/1/2707.:	--	--	--	0	0	0	0.24	1.95	0	2.78	0.44	3.43	9.74	12.49	6.69
2-3.c48879/1/2436	--	--	--	0.91	0	1.08	3.91	5.97	0	15.54	4.79	4.51	13.6	8.07	27.98
2-3.c49000/1/2204.	PREDICTED: ras-specific gu	363740538	XP_00364234	0.61	0.25	0.63	1.41	2.2	3.07	4.22	2.73	2.23	11.3	17.29	15.71
2-3.c49383/1/2447	PREDICTED: probable ubiqu	558212509	XP_00613401	0.21	3.16	0	5.2	0	5.15	10.29	1.26	4.17	20.86	20.89	19.44
2-3.c50403/1/2339	PREDICTED: huntingtin-inte	512820820	XP_00293359	6.61	23.85	8.07	1.19	3.87	3.68	2.81	1.95	5.13	1.79	1.2	0.44
2-3.c50765/5/2056	alpha1 type II collagen [Cync	5360532	BAA82043.1	8.78	21.95	33.74	6.15	0	0.04	0	0.01	1.82	0	0	0
2-3.c52175/1/2513	MGC79035 protein [Xenopus	147901187	NP_00108691	2.45	12.66	3.88	2.35	0.26	10.46	1.66	2.78	2.24	0.01	0	1.33
2-3.c52610/1/2742	PREDICTED: uncharacterize	847164282	XP_01282609	0	2.41	0.92	0	1.3	0.82	0	0.06	0	0	0	0
2-3.c53295/1/2070	PREDICTED: eukaryotic tran	558148079	XP_00612003	2.29	4.53	3	1.14	0	1.08	0.91	0	0	0	0	0
2-3.c53583/1/2006	solute carrier family 40 (iron-	147904330	NP_00109074	0	6.52	5.16	3.27	0.45	0	0	0	0	0	0	0
2-3.c53966/2/1914	Unknown (protein for MGC:8	49257408	AAH73381.1	0	0	0	5.35	0	0	10.08	0	0	4.82	18.12	10.77
2-3.c54192/1/2006	PREDICTED: tetratricopepti	530607046	XP_00529521	5.51	6.43	8.13	0	5	2.51	3.52	3.5	1.51	0	0	1.65
2-3.c54663/1/1999	death effector domain contair	148234641	NP_00108871	0	0	1.3	1.63	0.03	5.4	6.98	1.35	1.52	23.99	32.1	9.03
2-3.c54730/1/2370	PREDICTED: transcription e	512819310	XP_00491160	0.81	4.41	0.19	0	0	4.12	0.18	0	0	0	0	0
2-3.c54819/1/2637	--	--	--	0	0	0	2.68	0	0	0.87	1.03	3.01	1.25	0.76	0
2-3.c55405/1/2100.	PREDICTED: mucin-4-like i	847172450	XP_01280947	31.02	9.82	29.93	12.92	8.66	0.06	5.8	9.84	20.62	0	0	3.1
2-3.c55701/1/2518	PREDICTED: AT-hook DNA	847107809	XP_01281378	0.25	14.11	4.27	0	0	0	7.61	0	0	0.55	16.77	0
2-3.c56059/1/1916	--	--	--	0	0	0	0	55.41	20.46	53.81	0.27	71.08	19.1	14.2	16.65
2-3.c56406/1/2361	PREDICTED: moesin isoforr	301614728	XP_00293682	0	40.85	7.53	0	6.37	0	0	0	2.57	0	0	0
2-3.c56698/1/2650	PREDICTED: collagen alpha	512867119	XP_00293154	1.63	5.22	0	0	2.27	33.29	0	0	0	0	0	0
2-3.c57787/6/2491	serine incorporator 1 precurso	58332654	NP_00101139	9.16	2.18	5.47	0	0	1.9	2.09	0	0	0	0	0
2-3.c58668/8/2320	PREDICTED: dihydropyrimi	301603867	XP_00293158	0.69	16.93	1.88	0	0.43	22.04	0.54	0	0.56	0	0	0
2-3.c58683/2/2016	PREDICTED: serine/threonir	817327488	XP_01229070	0	0	0	4.08	0	1.62	8.81	1.89	2.14	20.99	13.96	11.91
2-3.c58886/1/2150	phosphatidylinositol glycan a	148237151	NP_00108743	0	0	0	0	0.3	0	0.67	0	0	2.35	5.06	2.05
2-3.c59882/1/2489	PREDICTED: dual specificit	847142251	XP_01282152	1.64	0	1.35	0	0	0	0.47	0	1.07	0	0	0
2-3.c60913/1/2359.	F-box/LRR-repeat protein 5 [	147901440	NP_00108506	0	0	0	0	0	3.01	1.23	0	0	0	3.11	2.58
2-3.c61128/1/2336.	gsn protein [Xenopus (Silurai	159155320	AAI54876.1	0	0	0	0.09	23.13	17.12	10.27	0	6.94	37.01	39.97	31.44
2-3.c61138/1/2177.	--	--	--	0.07	0	0.06	9.8	0	0	8.16	10.62	9.92	8.37	9.54	0.05
2-3.c62149/4/2004	zinc finger protein 207 [Xenc	89269088	CAJ81941.1	1.37	12.77	4.68	0	0	0	1.8	2.69	0	0	0	13.3
2-3.c62633/2/2459	--	--	--	0	0	0	0	0	14.75	12.88	0	8.31	11.4	0	0
2-3.c63021/2/1844	myosin-9 [Xenopus laevis]>g	148225656	NP_00108184	0	0	0	2.87	0	0	16.02	0	1.62	1.28	30.88	11.05
2-3.c63212/11/2174	gelsolin [Xenopus laevis]>gi|	148223299	NP_00108152	0	0	0	2.13	0	0	8.92	10.02	0.64	0	0	12.73
2-3.c63455/12/2006	PREDICTED: STAM-bindin	847108531	XP_01281402	0	0	0.36	1.36	0	0	2.56	0	2.39	9.7	6.9	1.79
2-3.c63615/1/1860.	PREDICTED: keratin, type II	795360232	XP_01178596	5.65	6.76	8.82	2.84	5.94	0	3.41	2.96	2.97	0	0	0
3-6.c2263/41/3494	PREDICTED: catenin beta-1-	573895693	XP_00663559	0	0	0	0	10.24	16.15	0	2.74	0	25.78	18.06	0.05
3-6.c2421/16/2779.	cyclin-dependent kinase inhit	168693517	NP_00110827	0.04	13.68	6.26	3.28	18.55	0	0	0.69	0	0	0	0
3-6.c2820/6/3827	vigilin [Xenopus (Silurana) tr	55742112	NP_00100685	0	7.56	2.88	2.22	0	0	4.47	0.35	0	0	0	0
3-6.c2877/4/3189	PREDICTED: nebulin isofori	847166508	XP_01282661	4.89	0	3.33	0	0	0	0	0	0	0	0	0
3-6.c3556/4/3493	profilin-2 [Xenopus (Silurana	62859423	NP_00101609	7.42	9.53	8.05	5.18	3.64	2.35	2.63	5.95	1.72	1.61	0.58	0.77
3-6.c3844/4/2748	PREDICTED: collagen alpha	847130150	XP_01281895	3.55	14.79	7.23	2.83	0	0	7.89	0	7.48	0	0	1.4
3-6.c4196/1/3592	PREDICTED: NACHT, LRR	699666029	XP_00988359	0	0	0	7.56	1.92	0	0	8.08	11.96	0.11	15.03	8.42
3-6.c4295/1/3409	importin-4 [Xenopus (Siluran	315707014	NP_00100801	2.79	6.07	2.62	0.84	1.32	8.96	1.19	0.85	0.55	0.07	0.21	0.03
3-6.c4314/1/3336	PREDICTED: spectrin alpha	847152581	XP_01282324	2.91	3.87	0.64	2.83	0.25	0	2.15	0	2.79	0.24	0	0
3-6.c5625/1/3448	PREDICTED: von Willebran	847103802	XP_01281319	0	0	0	1.8	1.38	0	0	0	0.1	2.14	2.67	0
3-6.c5795/1/2929	PREDICTED: N-alpha-acety	729723264	XP_01057483	0.22	2.94	1.42	0	0	0	0	1.42	0.78	0	0	0
3-6.c5821/1/3249.1	PREDICTED: target of Nesh	542203441	XP_00547555	0.09	3.6	1.09	0.08	0.7	6.15	0	0.28	0.08	0	0	0
3-6.c5936/1/3201	PREDICTED: serine/threonir	512819921	XP_00491168	0	0	0	0.59	0	0	1.59	0	0	3.62	2.6	0
3-6.c5940/1/2788.1	PREDICTED: DNA damage-	348560393	XP_00346599	4.56	1.56	2.25	0	0	0	0	0	0	0	0	0
3-6.c6437/1/3419	RecName: Full=Coiled-coil d	123892992	Q28G12.1	1.33	3.01	0.7	0	1.09	0.36	0.85	0	1.57	0.11	0	0
3-6.c6560/3/3081.1	MHC class II beta-chain, part	726968496	AIY53001.1	2.39	1.75	2.59	1.12	0	0.56	0.38	1.94	0	0	0	0
3-6.c6836/1/3338	PREDICTED: nebulin isofori	847166512	XP_01282661	18.93	18.17	0	0.01	25.62	0.92	0	0	0	0	0	0
3-6.c6860/1/3324.2	PREDICTED: ubiquitin carbo	719774765	XP_01022040	0.01	0	0	0	0	2.98	0.82	2.58	0	17	48.77	6.94
3-6.c6948/3/3149	PREDICTED: bcl-2-associate	847126738	XP_01281815	1.51	4.87	1.45	0	0.86	2.14	0.95	0.64	0.31	0.31	0	0

3-6.c7299/1/3683	PREDICTED: collagen alpha	512867463	XP_00293207	10.3	72.68	28.93	8.75	6.05	37.38	5.92	1.43	11.13	0	0	2.38
3-6.c7795/1/2729.1	--	--	--	10.4	5.84	9.66	9.88	0	0	0	5.11	0.11	0	0	0
3-6.c8144/1/2798.1	Collagen alpha-2(VI) chain, p	565318950	ETE70123.1	17.02	7.97	13.1	6.3	4.09	0.37	0.98	7.29	2.98	0	0	0
3-6.c8254/1/3164	PREDICTED: DDB1- and CU	512862186	XP_00293537	0	0	0	0	0	1.09	0.88	5.84	0.51	5.54	2.48	3.39
3-6.c8319/2/3083	PREDICTED: filamin-A isof	847155161	XP_01282406	2.08	6.46	7.31	0	0	9.06	0	5.83	0	0	0	0
3-6.c8547/1/3362	PREDICTED: transcription e	512819310	XP_00491160	0.08	14.22	2.06	0	0.59	12.05	0	0.72	0	0	0	0
3-6.c8713/1/2915.2	E3 ubiquitin-protein ligase M	62858193	NP_00101691	0	0	0	0	0	0.63	0	0	2.1	8.58	6.12	4.25
3-6.c10459/1/2744	PREDICTED: angiopoietin-r	634857825	XP_00794251	1.16	2.91	1.69	0	0	1.41	0	0	0	3.15	0.66	0
3-6.c10710/1/2755	heavy chain 6 cardiac muscle	388242704	AFK15622.1	0.86	5.39	2.43	0	0	0.03	1.93	7.15	1.86	0.89	0	1.8
3-6.c10790/1/3187	PREDICTED: glycine dehydr	512813230	XP_00293422	0	0	0	0.69	0	0	7.48	0	0.26	0	4.71	7.78
3-6.c10815/1/3255	PREDICTED: synaptopodin 2	301612611	XP_00293581	3.7	4.94	1.83	1.85	1.61	14.65	0.69	1.05	1.07	0	0	0.35
3-6.c11051/1/3707.	DNA polymerase delta cataly	113931510	NP_00103920	0.36	3.6	0.53	0	0.18	0.32	0.26	0.35	0.29	0	0	0
3-6.c11562/1/2885	PREDICTED: myocilin [Xen	512836045	XP_00293419	0	0	0	0	8.55	0.05	9.13	6.08	0	0.93	1.04	0.82
3-6.c13061/2/3077	PREDICTED: catenin beta-1	573895693	XP_00663559	0.01	66	14.41	0.07	0	0	0	3.78	0	0	0	0
3-6.c14063/1/3289	PREDICTED: E3 ubiquitin-p	512838975	XP_00491420	0	0	0	1.59	0	0.64	0	1.26	1.73	1.53	1.29	2.26
3-6.c14261/1/2908	spastin [Xenopus laevis]>gi|8	148231546	NP_00108672	0.91	10.49	6.12	0.78	0.1	0	0	0	0	0	0	2.69
3-6.c14395/1/2662	zinc transporter 7-A [Xenopu	147906284	NP_00108942	4.37	9.82	3.18	0	2.03	2.45	0	0	0	0	0	11.34
3-6.c14544/1/3195	PREDICTED: MIP18 family	301606783	XP_00293299	1.4	3.4	0	0.97	0	0.15	0	0	0	0	0	0
3-6.c14711/2/3327	PREDICTED: pre-mRNA-pr	847162749	XP_01282561	0.17	3.16	0.56	0	0	0	0	0	0	0	0	0
3-6.c15003/1/3224	Nucleoprotein TPR, partial [C	697445750	KGL86452.1	1.28	3.58	1.3	0.31	1.8	0.15	0.18	1.13	0.56	0	0.08	0.18
3-6.c15547/1/3120	cadherin-2 precursor [Xenopu	188528929	NP_00112089	0.73	1.48	0.84	0.27	0	1.24	0.15	0.38	0	0	0	0
3-6.c15615/1/2894	Guanine nucleotide-binding p	226372634	ACO51942.1	3.51	6.85	3.93	1.84	2.52	0	1.37	2.08	1.81	0.4	0.05	0.6
3-6.c15688/1/2756	clathrin, heavy chain (Hc) [X	148231021	NP_00108586	0.73	6.29	0.98	0	3.41	0	0	0	0	0	0	0
3-6.c15964/1/3916	PREDICTED: protein FAM1	530638059	XP_00530584	2.72	7.19	3.72	2.29	5.77	0.93	0.35	0.79	2.42	0.61	0.07	0.25
3-6.c16139/1/3102	PREDICTED: dickkopf-relat	301622013	XP_00294033	3.23	19.6	4.84	4.01	3.43	2.84	0	1.96	1.69	0.73	1.51	0
3-6.c16165/1/2871	replication factor C subunit 1	313760612	NP_00118649	9.28	27.31	5.28	5.4	3.13	4.83	1.85	6.42	2.67	0.77	0.97	1.49
3-6.c16516/1/3093	PREDICTED: laminin subun	512844752	XP_00293635	8.69	4.71	5.77	5.38	0	0	4.38	5.44	0	0	0	0.48
3-6.c16896/1/3338	PREDICTED: cold shock dor	847087986	XP_01281925	0	0	0.01	0.66	0	0.01	3.54	2.93	0	0.12	0	11.18
3-6.c16960/1/3804	PREDICTED: LOW QUALI	558146048	XP_00611959	0	0	0	0	0.36	1.41	2.04	1.9	0	0	0	2.97
3-6.c17082/1/2858.	hypothetical protein EH28_02	808879603	KKF27606.1	7.71	7.94	14.93	7.83	0	0	0	4.34	0	0	0	0
3-6.c17694/7/2933.	--	--	--	11.44	6.02	10.29	10.83	0.28	0.17	0.36	6.15	0	0	0.27	0.18
3-6.c18185/1/2750	CD82 molecule [Xenopus lae	148234160	NP_00108581	0	0	0	1.2	0	0	0	0	0.18	1.38	0.16	2.73
3-6.c18261/1/2819	PREDICTED: probable ATP-	591372795	XP_00706109	0.55	2.73	0.76	0.11	0.31	2.12	0.48	0	0.78	0	0	0
3-6.c18327/1/3138	forkhead box protein K2 [Xer	213982945	NP_00113563	3.15	13.61	4.73	2.46	3	0.56	1.82	0.97	2.38	0	0	1.8
3-6.c19028/3/3006	PREDICTED: cytochrome P4	512848263	XP_00293789	6.24	16.42	3.12	0.26	0	0	0	2.36	1.68	2.34	0	4.79
