# Supplementary material for: Gene Expression Program Underlying Tail Resorption During Thyroid Hormone-Dependent Metamorphosis of the Ornamented Pygmy Frog Microhyla fissipes
Source: Front Endocrinol (Lausanne). 2019 Jan 25;10:11. doi: 10.3389/fendo.2019.00011 (PMC6357680; doi:10.3389/fendo.2019.00011)
Supplement: Supplementary file 3 [file Table_3.pdf]

Table S3. Enriched KEGG pathways among the up-regulated genes during tail resorption.
The 1,030 genes in the upregulated clusters 5, 8, 13, 21, 23, 26, in Fig. 6, were combined and analyzed. Eleven pathways were found to be significantly enriched (p<0.01, ranking by p value).

KEGG Pathway	Pathway ID	Enriched Genes	Background Genes	p-Value	q-Value
Vitamin digestion and absorption	ko04977	6	40	0.000403	0.07461
Glycosphingolipid biosynthesis globo series	ko00603	4	15	0.000599	0.07461
Other glycan degradation	ko00511	6	57	0.002157	0.13887
Vibrio cholerae infection	ko05110	14	259	0.002258	0.13887
Bladder cancer	ko05219	9	129	0.002788	0.13887
Parkinson's disease	ko05012	15	306	0.003864	0.15907
Vitamin B6 metabolism	ko00750	4	28	0.004472	0.15907
Lysosome	ko04142	21	517	0.005975	0.17136
Glycosphingolipid biosynthesis ganglio series	ko00604	4	31	0.006194	0.17136
Retinol metabolism	ko00830	10	181	0.007867	0.1959
Gap junction	ko04540	13	274	0.008767	0.19845
