# Supplementary material for: Gene Expression Program Underlying Tail Resorption During Thyroid Hormone-Dependent Metamorphosis of the Ornamented Pygmy Frog Microhyla fissipes
Source: Front Endocrinol (Lausanne). 2019 Jan 25;10:11. doi: 10.3389/fendo.2019.00011 (PMC6357680; doi:10.3389/fendo.2019.00011)
Supplement: Supplementary file 4 [file Table_4.pdf]

Table S4. Enriched GO terms among the up-regulated genes during tail resorption.
The 1,030 genes in the upregulated clusters 5, 8, 13, 21, 23, 26, in Fig. 6, were combined and analyzed. Eighty-eight significantly enriched (p<0.01, ranking by p value) GO terms were found.

GO_accession	Description	Term_type	p-Value	q-Value	Enriched Genes	Background Genes
GO:0006508	proteolysis	biological_process	1.97E-05	0.13829	60	2187
GO:0006810	transport	biological_process	9.92E-05	0.16396	159	7641
GO:0004175	endopeptidase activity	molecular_function	0.00011712	0.16396	35	1105
GO:0051234	establishment of localization	biological_process	0.00012829	0.16396	159	7682
GO:0015929	hexosaminidase activity	molecular_function	0.00013785	0.16396	4	14
GO:0008233	peptidase activity	molecular_function	0.00014028	0.16396	51	1861
GO:0005737	cytoplasm	cellular_component	0.00016831	0.16862	151	7215
GO:0070011	peptidase activity, acting on L-amino acid peptides	molecular_function	0.00023691	0.20768	47	1707
GO:0004563	beta-N-acetylhexosaminidase activity	molecular_function	0.00043527	0.29156	3	8
GO:0004222	metalloendopeptidase activity	molecular_function	0.00043852	0.29156	14	328
GO:0051179	localization	biological_process	0.00045732	0.29156	163	8123
GO:0007517	muscle organ development	biological_process	0.00052462	0.30606	5	45
GO:0005739	mitochondrion	cellular_component	0.00061814	0.30606	31	995
GO:0006687	glycosphingolipid metabolic process	biological_process	0.00061944	0.30606	6	59
GO:0012505	endomembrane system	cellular_component	0.00065462	0.30606	49	1927
GO:0044429	mitochondrial part	cellular_component	0.00077083	0.33786	25	761
GO:1902578	single-organism localization	biological_process	0.00093391	0.38526	116	5531
GO:0044765	single-organism transport	biological_process	0.0011363	0.4427	113	5396
GO:0019538	protein metabolic process	biological_process	0.0012224	0.45118	147	7350
GO:0006869	lipid transport	biological_process	0.0013097	0.45924	13	314
GO:0044444	cytoplasmic part	cellular_component	0.001848	0.4983	110	5293
GO:0006665	sphingolipid metabolic process	biological_process	0.0019179	0.4983	6	75
GO:0034214	protein hexamerization	biological_process	0.0020467	0.4983	3	21
GO:0031967	organelle envelope	cellular_component	0.0020555	0.4983	24	791
GO:0016715	oxidoreductase activity, acting on paired donors, with incorporation or reduction of molecular oxygen, reduced ascorbate as one donor, and incorporation of one atom of oxygen	molecular_function	0.0021313	0.4983	2	5
GO:0008237	metallopeptidase activity	molecular_function	0.0021555	0.4983	16	455
GO:0031975	envelope	cellular_component	0.0021777	0.4983	25	837
GO:0008483	transaminase activity	molecular_function	0.0022598	0.4983	6	89
GO:0008053	mitochondrial fusion	biological_process	0.002269	0.4983	6	86
GO:0005741	mitochondrial outer membrane	cellular_component	0.0022945	0.4983	11	240
GO:0031090	organelle membrane	cellular_component	0.0023213	0.4983	43	1742
GO:0010876	lipid localization	biological_process	0.0023596	0.4983	13	333

GO:0031968	organelle outer membrane	cellular_component	0.0024201	0.4983	11	242
GO:0005740	mitochondrial envelope	cellular_component	0.0024903	0.4983	21	659
GO:0008641	small protein activating enzyme activity	molecular_function	0.0025526	0.4983	5	70
GO:0008289	lipid binding	molecular_function	0.002558	0.4983	21	669
GO:0001575	globoside metabolic process	biological_process	0.0029215	0.52585	3	16
GO:0006509	membrane protein ectodomain proteolysis	biological_process	0.0029426	0.52585	2	5
GO:0033619	membrane protein proteolysis	biological_process	0.0029426	0.52585	2	5
GO:0098588	bounding membrane of organelle	cellular_component	0.0030902	0.52585	34	1322
GO:0031419	cobalamin binding	molecular_function	0.0031276	0.52585	3	17
GO:0003824	catalytic activity	molecular_function	0.0032031	0.52585	331	18458
GO:0031966	mitochondrial membrane	cellular_component	0.0032242	0.52585	20	628
GO:0016769	transferase activity, transferring nitrogenous groups	molecular_function	0.0033606	0.53563	6	95
GO:0004252	serine-type endopeptidase activity	molecular_function	0.0036532	0.56099	10	218
GO:0005783	endoplasmic reticulum	cellular_component	0.0038488	0.56099	28	1029
GO:0071702	organic substance transport	biological_process	0.0038648	0.56099	75	3512
GO:0003994	aconitate hydratase activity	molecular_function	0.003901	0.56099	3	21
GO:0005244	voltage-gated ion channel activity	molecular_function	0.0041069	0.56099	7	139
GO:0022832	voltage-gated channel activity	molecular_function	0.0041069	0.56099	7	139
GO:0008135	translation factor activity, RNA binding	molecular_function	0.0042731	0.56099	12	324
GO:0016877	ligase activity, forming carbon-sulfur bonds	molecular_function	0.0042796	0.56099	5	79
GO:0019825	oxygen binding	molecular_function	0.0043302	0.56099	3	17
GO:0004012	phospholipid-translocating ATPase activity	molecular_function	0.0044374	0.56099	2	7
GO:0015917	aminophospholipid transport	biological_process	0.0044374	0.56099	2	7
GO:0051259	protein oligomerization	biological_process	0.0044796	0.56099	13	401
GO:0005548	phospholipid transporter activity	molecular_function	0.0048885	0.59109	2	8
GO:0015914	phospholipid transport	biological_process	0.0048885	0.59109	2	8
GO:0061061	muscle structure development	biological_process	0.0052152	0.6199	5	77
GO:0048284	organelle fusion	biological_process	0.0053538	0.62577	6	109
GO:0006448	regulation of translational elongation	biological_process	0.0058647	0.64647	5	85
GO:0042157	lipoprotein metabolic process	biological_process	0.0059155	0.64647	9	222
GO:0000726	non-recombinational repair	biological_process	0.0060105	0.64647	3	22
GO:0006303	double-strand break repair via nonhomologous end joining	biological_process	0.0060105	0.64647	3	22
GO:0006629	lipid metabolic process	biological_process	0.0061326	0.64647	34	1312
GO:0044255	cellular lipid metabolic process	biological_process	0.0061648	0.64647	27	975
GO:0030354	melanin-concentrating hormone activity	molecular_function	0.0061762	0.64647	2	6
GO:0006414	translational elongation	biological_process	0.0063255	0.6506	5	86
GO:0070647	protein modification by small protein conjugation or removal	biological_process	0.0064012	0.6506	29	1182
GO:0006099	tricarboxylic acid cycle	biological_process	0.0071633	0.67668	7	141
GO:0006101	citrate metabolic process	biological_process	0.0071633	0.67668	7	141
GO:0044801	single-organism membrane fusion	biological_process	0.0072858	0.67668	6	115
GO:0072350	tricarboxylic acid metabolic process	biological_process	0.0073579	0.67668	7	142

GO:0004348	glucosylceramidase activity	molecular_function	0.0073945	0.67668	2	7
GO:0030170	pyridoxal phosphate binding	molecular_function	0.0074189	0.67668	9	225
GO:0098805	whole membrane	cellular_component	0.0074805	0.67668	19	662
GO:0006417	regulation of translation	biological_process	0.0075262	0.67668	13	406
GO:0034248	regulation of cellular amide metabolic process	biological_process	0.0075262	0.67668	13	406
GO:0006820	anion transport	biological_process	0.0081605	0.72336	14	454
GO:0008199	ferric iron binding	molecular_function	0.0082517	0.72336	4	52
GO:0035101	FACT complex	cellular_component	0.0083602	0.72383	2	11
GO:0016885	ligase activity, forming carbon-carbon bonds	molecular_function	0.0086802	0.72476	4	50
GO:0003746	translation elongation factor activity	molecular_function	0.0089373	0.72476	4	49
GO:0032446	protein modification by small protein conjugation	biological_process	0.0090226	0.72476	25	999
GO:0010608	posttranscriptional regulation of gene expression	biological_process	0.0090623	0.72476	13	419
GO:0016838	carbon-oxygen lyase activity, acting on phosphates	molecular_function	0.0094076	0.72476	3	29
GO:0019028	viral capsid	cellular_component	0.0095691	0.72476	31	1213
GO:0019012	virion	cellular_component	0.0097053	0.72476	39	1642
