# Supplementary material for: Gene Expression Program Underlying Tail Resorption During Thyroid Hormone-Dependent Metamorphosis of the Ornamented Pygmy Frog Microhyla fissipes
Source: Front Endocrinol (Lausanne). 2019 Jan 25;10:11. doi: 10.3389/fendo.2019.00011 (PMC6357680; doi:10.3389/fendo.2019.00011)
Supplement: Supplementary file 5 [file Table_5.pdf]

Table S5. Upregulated genes in the proteolysis GO terms during tail resorption. 60 genes were dramatically upregulated at S43. These genes mainly encode enzymes, including hydrolase, protease, and matrix metalloproteinase, and are consistent with rapid tissue resorption.

<table>
<tr><th>Gene ID</th><th>NR GI</th><th>NR ID</th><th>NR Description</th></tr>
<tr><td>1-2.c43575/1/</td><td>823470855</td><td>XP_012424179.1</td><td>PREDICTED: TATA-binding protein-associated factor 2N isoform X2 [Taeniopygia guttata]</td></tr>
<tr><td>1-2.c43666/1/</td><td>4249695</td><td>AAD13769.1</td><td>myosin heavy chain, partial [Rana catesbeiana]</td></tr>
<tr><td>1-2.c34981/1/</td><td>49522510</td><td>AAH75571.1</td><td>HLA-B associated transcript 5 [Xenopus (Silurana) tropicalis]</td></tr>
<tr><td>1-2.c50028/1/</td><td>847108531</td><td>XP_012814021.1</td><td>PREDICTED: STAM-binding protein isoform X1 [Xenopus (Silurana) tropicalis]>gi|847108535|ref|XP_012814022.1| PREDICTED: STAM-binding protein isoform X1 [Xenopus (Silurana) tropicalis]</td></tr>
<tr><td>3-6.c14239/1/</td><td>780154032</td><td>XP_011682115.1</td><td>PREDICTED: glutamic acid-rich protein-like [Strongylocentrotus purpuratus]</td></tr>
<tr><td>2-3.c20238/3/</td><td>120538440</td><td>AAI29682.1</td><td>Unknown (protein for MGC:160365) [Xenopus laevis]</td></tr>
<tr><td>2-3.c29517/3/</td><td>301614077</td><td>XP_002936515.1</td><td>PREDICTED: ubiquitin carboxyl-terminal hydrolase 4 [Xenopus (Silurana) tropicalis]</td></tr>
<tr><td>1-2.c43062/1/</td><td>56270034</td><td>AAH87471.1</td><td>Htra1 protein [Xenopus laevis]</td></tr>
<tr><td>2-3.c7627/1/2</td><td>189441985</td><td>AAI67321.1</td><td>LOC100170459 protein [Xenopus (Silurana) tropicalis]</td></tr>
<tr><td>1-2.c19112/1/</td><td>148231526</td><td>NP_001087077.1</td><td>E3 ubiquitin-protein ligase HACE1 [Xenopus laevis]>gi|82200059|sp|Q6DCL5.1|HACE1_XENLA RecName: Full=E3 ubiquitin-protein ligase HACE1; AltName: Full=HECT domain and ankyrin repeat-containing E3 ubiquitin-protein ligase 1 [Xenopus laevis]>gi|50415848|gb|AAH77993.1| Hace1-prov protein [Xenopus laevis]</td></tr>
<tr><td>2-3.c63455/12</td><td>847108531</td><td>XP_012814021.1</td><td>PREDICTED: STAM-binding protein isoform X1 [Xenopus (Silurana) tropicalis]>gi|847108535|ref|XP_012814022.1| PREDICTED: STAM-binding protein isoform X1 [Xenopus (Silurana) tropicalis]</td></tr>
<tr><td>1-2.c38617/1/</td><td>147901642</td><td>NP_001091305.1</td><td>matrix metalloproteinase-9TH precursor [Xenopus laevis]>gi|119709546|dbj|BAF42673.1| matrix metalloproteinase-9TH [Xenopus laevis]</td></tr>
<tr><td>2-3.c43189/1/</td><td>148237354</td><td>NP_001081970.1</td><td>cingulin [Xenopus laevis]>gi|6636514|gb|AAF20208.1|AF207901_1 cingulin [Xenopus laevis]</td></tr>
<tr><td>1-2.c51308/1(</td><td>847156920</td><td>XP_012824466.1</td><td>PREDICTED: myosin-4 [Xenopus (Silurana) tropicalis]</td></tr>
<tr><td>1-2.c38664/1/</td><td>148235901</td><td>NP_001080371.1</td><td>elongation of very long chain fatty acids-like 1 [Xenopus laevis]>gi|27503186|gb|AAH42304.1| Elovl1-prov protein [Xenopus laevis]</td></tr>
<tr><td>1-2.c19767/1/</td><td>148222777</td><td>NP_001081720.1</td><td>protease, serine, 8 precursor [Xenopus laevis]>gi|2599504|gb|AAB96905.1| serine protease [Xenopus laevis]</td></tr>
<tr><td>2-3.c62973/3/</td><td>132424622</td><td>ABO33468.1</td><td>endothelin converting enzyme-1 [Xenopus laevis]</td></tr>
<tr><td>2-3.c47226/1/</td><td>156717508</td><td>NP_001096294.1</td><td>tripeptidyl-peptidase 1 precursor [Xenopus (Silurana) tropicalis]>gi|134023873|gb|AAI35588.1| LOC100124868 protein [Xenopus (Silurana) tropicalis]</td></tr>
<tr><td>1-2.c15452/2/</td><td>147901642</td><td>NP_001091305.1</td><td>matrix metalloproteinase-9TH precursor [Xenopus laevis]>gi|119709546|dbj|BAF42673.1| matrix metalloproteinase-9TH [Xenopus laevis]</td></tr>
<tr><td>2-3.c41773/1/</td><td>148225652</td><td>NP_001088463.1</td><td>ubiquitin thioesterase zranb1-A [Xenopus laevis]>gi|82180146|sp|Q5U595.1|ZRN1A_XENLA RecName: Full=Ubiquitin thioesterase zranb1-A; AltName: Full=Zinc finger Ran-binding domain-containing protein 1A>gi|54311385|gb|AAH84789.1| Zranb1-a protein [Xenopus laevis]</td></tr>
<tr><td>1-2.c51832/2/</td><td>54020950</td><td>NP_001005720.1</td><td>legumain precursor [Xenopus (Silurana) tropicalis]>gi|49523231|gb|AAH75316.1| legumain [Xenopus (Silurana) tropicalis]</td></tr>
<tr><td>2-3.c5808/1/7</td><td>147901642</td><td>NP_001091305.1</td><td>matrix metalloproteinase-9TH precursor [Xenopus laevis]>gi|119709546|dbj|BAF42673.1| matrix metalloproteinase-9TH [Xenopus laevis]</td></tr>
<tr><td>1-2.c45335/1/</td><td>156717508</td><td>NP_001096294.1</td><td>tripeptidyl-peptidase 1 precursor [Xenopus (Silurana) tropicalis]>gi|134023873|gb|AAI35588.1| LOC100124868 protein [Xenopus (Silurana) tropicalis]</td></tr>
<tr><td>2-3.c26883/1/</td><td>847108105</td><td>XP_012813879.1</td><td>PREDICTED: dipeptidyl peptidase 8 isoform X1 [Xenopus (Silurana) tropicalis]</td></tr>
<tr><td>1-2.c24732/1/</td><td>148237346</td><td>NP_001084981.1</td><td>RNA-binding motif protein, X chromosome [Xenopus laevis]>gi|82236857|sp|Q6IRQ4.1|RBMX_XENLA RecName: Full=RNA-binding motif protein, X chromosome; AltName: Full=Heterogeneous nuclear ribonucleoprotein G; Short=hnRNP G [Xenopus laevis]>gi|47682580|gb|AAH70649.1| MGC82187 protein [Xenopus laevis]</td></tr>
<tr><td>1-2.c25479/4/</td><td>148222361</td><td>NP_001086687.1</td><td>ubiquinol-cytochrome c reductase core protein II [Xenopus laevis]>gi|50418237|gb|AAH77311.1| Uqcrc2 protein [Xenopus laevis]>gi|77748481|gb|AAI06253.1| Uqcrc2 protein [Xenopus laevis]</td></tr>
<tr><td>1-2.c32693/1/</td><td>847165172</td><td>XP_012826266.1</td><td>PREDICTED: matrix metalloproteinase-25 [Xenopus (Silurana) tropicalis]</td></tr>
<tr><td>1-2.c12883/1/</td><td>301612173</td><td>XP_002935612.1</td><td>PREDICTED: 26S proteasome non-ATPase regulatory subunit 11 [Xenopus (Silurana) tropicalis]>gi|410591661|sp|F6XBL2.2|PSD11_XENTR RecName: Full=26S proteasome non-ATPase regulatory subunit 11; AltName: Full=26S proteasome regulatory subunit RPN6</td></tr>
<tr><td>1-2.c8712/1/1</td><td>54020950</td><td>NP_001005720.1</td><td>legumain precursor [Xenopus (Silurana) tropicalis]>gi|49523231|gb|AAH75316.1| legumain [Xenopus (Silurana) tropicalis]</td></tr>
<tr><td>1-2.c50942/4/</td><td>213625177</td><td>AAI69984.1</td><td>Complement factor B [Xenopus laevis]</td></tr>
<tr><td>1-2.c44265/1/</td><td>669271014</td><td>XP_008627462.1</td><td>PREDICTED: phosphate-regulating neutral endopeptidase [Corvus brachyrhynchos]>gi|727005437|ref|XP_010392798.1| PREDICTED: phosphate-regulating neutral endopeptidase [Corvus cornix cornix]>gi|676411760|gb|KFO55745.1| Metalloendopeptidase PEX [Corvus brachyrhynchos]</td></tr>
<tr><td>1-2.c45883/1/</td><td>558221328</td><td>XP_006135955.1</td><td>PREDICTED: cathepsin L1-like [Pelodiscus sinensis]</td></tr>
<tr><td>1-2.c35572/1/</td><td>113931624</td><td>NP_001039264.1</td><td>novel aldo-keto reductase family 1 c (akr1c) protein [Xenopus (Silurana) tropicalis]>gi|89272462|emb|CAJ83089.1| novel aldo-keto reductase family 1 c (akr1c) protein [Xenopus (Silurana) tropicalis]>gi|157423623|gb|AAI53677.1| novel aldo-keto reductase family 1 c (akr1c) protein [Xenopus (Silurana) tropicalis]</td></tr>
<tr><td>1-2.c36197/1/</td><td>134085433</td><td>NP_001015975.2</td><td>cullin-3 [Xenopus (Silurana) tropicalis]>gi|254766448|sp|A4IHP4.1|CUL3_XENTR RecName: Full=Cullin-3; Short=CUL-3>gi|134023875|gb|AAI35617.1| cul3 protein [Xenopus (Silurana) tropicalis]</td></tr>
<tr><td>2-3.c20888/1/</td><td>213626825</td><td>AAI70192.1</td><td>Complement factor B [Xenopus laevis]</td></tr>
<tr><td>2-3.c8132/1/2</td><td>257051069</td><td>P23787.3</td><td>RecName: Full=Transitional endoplasmic reticulum ATPase; Short=TER ATPase; AltName: Full=15S Mg(2+)-ATPase p97 subunit; Short=p97; AltName: Full=Valosin-containing protein; Short=VCP [Xenopus laevis]>gi|28422362|gb|AAH46949.1| Vcp-prov protein [Xenopus laevis]</td></tr>
<tr><td>2-3.c46272/1/</td><td>187608155</td><td>NP_001120167.1</td><td>ubiquitin carboxyl-terminal hydrolase 48 [Xenopus (Silurana) tropicalis]>gi|166796872|gb|AAI59175.1| LOC100145207 protein [Xenopus (Silurana) tropicalis]</td></tr>
<tr><td>1-2.c18576/1/</td><td>301614077</td><td>XP_002936515.1</td><td>PREDICTED: ubiquitin carboxyl-terminal hydrolase 4 [Xenopus (Silurana) tropicalis]</td></tr>
<tr><td>1-2.c1924/24/</td><td>847087076</td><td>XP_012816229.1</td><td>PREDICTED: cytosol aminopeptidase isoform X1 [Xenopus (Silurana) tropicalis]</td></tr>
<tr><td>1-2.c8562/2/1</td><td>148228853</td><td>NP_001081066.1</td><td>matriptase a [Xenopus laevis]>gi|49257232|gb|AAH71077.1| St14-A-prov protein [Xenopus laevis]</td></tr>
<tr><td>1-2.c51922/5/</td><td>148234658</td><td>NP_001080810.1</td><td>keratin, type I cytoskeletal 18-B [Xenopus laevis]>gi|82207929|sp|Q7SY65.1|K118B_XENLA RecName: Full=Keratin, type I cytoskeletal 18-B; AltName: Full=Cytokeratin-18-B; Short=CK-18-B; AltName: Full=Keratin-18-B; Short=K18-B [Xenopus laevis]>gi|32766495|gb|AAH54993.1| Krt18-prov protein [Xenopus laevis]</td></tr>
<tr><td>1-2.c19245/2/</td><td>148228748</td><td>NP_001084691.1</td><td>leucine aminopeptidase 3 [Xenopus laevis]>gi|46249508|gb|AAH68707.1| MGC81140 protein [Xenopus laevis]</td></tr>
<tr><td>1-2.c50141/1/</td><td>147902934</td><td>NP_001079714.1</td><td>ubiquinol-cytochrome c reductase core protein I [Xenopus laevis]>gi|29351607|gb|AAH49288.1| Uqcrc1 protein [Xenopus laevis]</td></tr>
<tr><td>1-2.c25522/1/</td><td>847156920</td><td>XP_012824466.1</td><td>PREDICTED: myosin-4 [Xenopus (Silurana) tropicalis]</td></tr>
<tr><td>1-2.c33102/1/</td><td>147902934</td><td>NP_001079714.1</td><td>ubiquinol-cytochrome c reductase core protein I [Xenopus laevis]>gi|29351607|gb|AAH49288.1| Uqcrc1 protein [Xenopus laevis]</td></tr>
<tr><td>2-3.c29390/1/</td><td>124504280</td><td>AAI28677.1</td><td>Mmp-9th protein [Xenopus laevis]</td></tr>
<tr><td>1-2.c52297/1/</td><td>257051069</td><td>P23787.3</td><td>RecName: Full=Transitional endoplasmic reticulum ATPase; Short=TER ATPase; AltName: Full=15S Mg(2+)-ATPase p97 subunit; Short=p97; AltName: Full=Valosin-containing protein; Short=VCP [Xenopus laevis]>gi|28422362|gb|AAH46949.1| Vcp-prov protein [Xenopus laevis]</td></tr>
<tr><td>1-2.c39392/1/</td><td>89268944</td><td>CAJ81959.1</td><td>myosin X [Xenopus (Silurana) tropicalis]</td></tr>
<tr><td>2-3.c39049/1/</td><td>--</td><td>--</td><td>--</td></tr>
<tr><td>1-2.c40238/1/</td><td>148225152</td><td>NP_001079747.1</td><td>F-box/LRR-repeat protein 15 [Xenopus laevis]>gi|82187926|sp|Q7SZ73.1|FXL15_XENLA RecName: Full=F-box/LRR-repeat protein 15 [Xenopus laevis]>gi|32450295|gb|AAH53821.1| MGC64561 protein [Xenopus laevis]</td></tr>
<tr><td>1-2.c33717/1/</td><td>147901642</td><td>NP_001091305.1</td><td>matrix metalloproteinase-9TH precursor [Xenopus laevis]>gi|119709546|dbj|BAF42673.1| matrix metalloproteinase-9TH [Xenopus laevis]</td></tr>
<tr><td>1-2.c19308/2/</td><td>56118680</td><td>NP_001008015.1</td><td>E3 ubiquitin-protein ligase RNF13 precursor [Xenopus (Silurana) tropicalis]>gi|847127421|ref|XP_012818369.1| PREDICTED: E3 ubiquitin-protein ligase RNF13 isoform X1 [Xenopus (Silurana) tropicalis]>gi|51704075|gb|AAH80893.1| ring finger protein 13 [Xenopus (Silurana) tropicalis]</td></tr>
<tr><td>2-3.c49598/1/</td><td>213623754</td><td>AAI70178.1</td><td>Casp9-A protein [Xenopus laevis]>gi|213627647|gb|AAI70180.1| Casp9-A protein [Xenopus laevis]</td></tr>
<tr><td>1-2.c34897/1/</td><td>147906566</td><td>NP_001091172.1</td><td>uncharacterized protein LOC100036932 [Xenopus laevis]>gi|120538016|gb|AAI29683.1| LOC100036932 protein [Xenopus laevis]</td></tr>
<tr><td>1-2.c24322/1/</td><td>301608280</td><td>XP_002933705.1</td><td>PREDICTED: paraplegin [Xenopus (Silurana) tropicalis]</td></tr>
<tr><td>2-3.c60913/1/</td><td>147901440</td><td>NP_001085061.1</td><td>F-box/LRR-repeat protein 5 [Xenopus laevis]>gi|82236774|sp|Q6INS1.1|FBXL5_XENLA RecName: Full=F-box/LRR-repeat protein 5; AltName: Full=F-box and leucine-rich repeat protein 5 [Xenopus laevis]>gi|47940274|gb|AAH72202.1| MGC81139 protein [Xenopus laevis]</td></tr>
<tr><td>2-3.c27424/1/</td><td>59861981</td><td>AAH90369.1</td><td>LOC548400 protein, partial [Xenopus (Silurana) tropicalis]</td></tr>
<tr><td>1-2.c52192/7/</td><td>148222777</td><td>NP_001081720.1</td><td>protease, serine, 8 precursor [Xenopus laevis]>gi|2599504|gb|AAB96905.1| serine protease [Xenopus laevis]</td></tr>
<tr><td>2-3.c25918/1/</td><td>120538440</td><td>AAI29682.1</td><td>Unknown (protein for MGC:160365) [Xenopus laevis]</td></tr>
<tr><td>1-2.c31270/2/</td><td>213982845</td><td>NP_001135590.1</td><td>beta-secretase 2 precursor [Xenopus (Silurana) tropicalis]>gi|195540077|gb|AAI68114.1| Unknown (protein for MGC:186115) [Xenopus (Silurana) tropicalis]</td></tr>
</table>
