# Supplementary material for: Gene Expression Program Underlying Tail Resorption During Thyroid Hormone-Dependent Metamorphosis of the Ornamented Pygmy Frog Microhyla fissipes
Source: Front Endocrinol (Lausanne). 2019 Jan 25;10:11. doi: 10.3389/fendo.2019.00011 (PMC6357680; doi:10.3389/fendo.2019.00011)
Supplement: Supplementary file 6 [file Table_6.pdf]

Table S6. Enriched KEGG pathways among the down-regulated genes during tail resorption. 1,596 genes in the down-regulated cluster-3, 9, 10, 12, 20, 22, 25 in Fig. 6, were combined and analyzed. Twenty-one pathways were found to be significantly enriched ($q<0.05$, ranking by q value).

KEGG Pathway	Pathway ID	Enriched Genes	Background Genes	p-Value	q-Value
Tight junction	ko04530	110	1367	1.33E-14	3.51E-12
Valine, leucine and isoleucine degradation	ko00280	31	247	6.81E-09	8.99E-07
Aminoacyl-tRNA biosynthesis	ko00970	25	186	5.77E-08	5.07E-06
Cardiac muscle contraction	ko04260	38	401	1.45E-07	9.59E-06
ECM-receptor interaction	ko04512	53	687	3.01E-07	1.59E-05
Protein digestion and absorption	ko04974	46	617	4.07E-06	0.000179255
Citrate cycle (TCA cycle)	ko00020	23	218	8.14E-06	0.00030707
Focal adhesion	ko04510	75	1341	9.16E-05	0.002765869
Propanoate metabolism	ko00640	17	158	9.43E-05	0.002765869
Pyruvate metabolism	ko00620	19	196	0.00013392	0.003535594
Parkinson's disease	ko05012	25	306	0.00016526	0.003966171
Adrenergic signaling in cardiomyocytes	ko04261	43	678	0.00025602	0.005632485
Starch and sucrose metabolism	ko00500	18	195	0.00034301	0.006647452
Glycolysis / Gluconeogenesis	ko00010	36	542	0.00035252	0.006647452
Glyoxylate and dicarboxylate metabolism	ko00630	17	181	0.00041185	0.007248551
Purine metabolism	ko00230	28	402	0.00077144	0.012728804
Tyrosine metabolism	ko00350	11	100	0.00130962	0.020262416
PI3K-Akt signaling pathway	ko04151	67	1293	0.00138153	0.020262416
Galactose metabolism	ko00052	13	139	0.00194271	0.026993456
Glycine, serine and threonine metabolism	ko00260	15	186	0.00346062	0.045642838
Alzheimer's disease	ko05010	36	626	0.00363068	0.045642838
