# Supplementary material for: Gene Expression Program Underlying Tail Resorption During Thyroid Hormone-Dependent Metamorphosis of the Ornamented Pygmy Frog Microhyla fissipes
Source: Front Endocrinol (Lausanne). 2019 Jan 25;10:11. doi: 10.3389/fendo.2019.00011 (PMC6357680; doi:10.3389/fendo.2019.00011)
Supplement: Supplementary file 7 [file Table_7.pdf]

Table S7. Enriched GO terms among the down-regulated genes during tail resorption. 1,596 genes in the down-regulated cluster-3, 9, 10, 12, 20, 22, 25 in Fig. 6, were combined and analyzed, and 499 significantly enriched (p<0.05, ranking by q value) GO terms were addressed.

GO_accession	Description	p-Value	q-Value	DETs	Background genes
GO:0005882	intermediate filament	8.82E-26	3.09E-22	104	1019
GO:0045111	intermediate filament cytoskeleton	8.82E-26	3.09E-22	104	1019
GO:0015629	actin cytoskeleton	4.65E-23	1.09E-19	100	983
GO:0016459	myosin complex	1.60E-22	2.80E-19	92	850
GO:0005856	cytoskeleton	1.95E-18	2.73E-15	172	2839
GO:0003774	motor activity	1.52E-17	1.78E-14	94	1075
GO:0044430	cytoskeletal part	1.07E-16	9.75E-14	154	2526
GO:0006606	protein import into nucleus	1.67E-16	9.75E-14	75	849
GO:0034504	protein localization to nucleus	1.67E-16	9.75E-14	75	849
GO:0044744	protein targeting to nucleus	1.67E-16	9.75E-14	75	849
GO:0051170	nuclear import	1.67E-16	9.75E-14	75	849
GO:1902593	single-organism nuclear import	1.67E-16	9.75E-14	75	849
GO:0006913	nucleocytoplasmic transport	5.37E-16	2.90E-13	77	908
GO:0051169	nuclear transport	6.98E-16	3.30E-13	77	912
GO:0017038	protein import	7.06E-16	3.30E-13	78	931
GO:0016462	pyrophosphatase activity	2.76E-15	1.21E-12	170	2922
GO:0006605	protein targeting	3.35E-15	1.38E-12	86	1116
GO:0072594	establishment of protein localization to organelle	4.26E-15	1.66E-12	83	1055
GO:0017111	nucleoside-triphosphatase activity	8.38E-15	3.09E-12	168	2908
GO:0043623	cellular protein complex assembly	9.62E-15	3.37E-12	99	1418
GO:0016818	hydrolase activity, acting on acid anhydrides, in phosphorus-containing anhydrides	1.10E-14	3.68E-12	171	2991
GO:0036094	small molecule binding	1.94E-14	6.18E-12	299	6416
GO:0005198	structural molecule activity	4.23E-14	1.29E-11	162	2843
GO:0001775	cell activation	4.44E-14	1.30E-11	65	762
GO:0051258	protein polymerization	4.69E-14	1.32E-11	70	875
GO:0005577	fibrinogen complex	5.07E-14	1.32E-11	64	747
GO:0030168	platelet activation	5.07E-14	1.32E-11	64	747
GO:1902580	single-organism cellular localization	1.27E-13	3.18E-11	85	1168
GO:0000166	nucleotide binding	1.87E-13	4.37E-11	289	6257
GO:1901265	nucleoside phosphate binding	1.87E-13	4.37E-11	289	6257
GO:0016482	cytoplasmic transport	1.98E-13	4.47E-11	89	1255
GO:0007596	blood coagulation	2.18E-13	4.78E-11	64	775
GO:0033365	protein localization to organelle	2.55E-13	5.42E-11	84	1158
GO:0030674	protein binding, bridging	3.65E-13	7.31E-11	64	779
GO:0060090	binding, bridging	3.65E-13	7.31E-11	64	779
GO:1902582	single-organism intracellular transport	3.93E-13	7.65E-11	113	1746
GO:0016817	hydrolase activity, acting on acid anhydrides	4.54E-13	8.61E-11	172	3159
GO:0007599	hemostasis	4.87E-13	8.76E-11	64	792
GO:0050878	regulation of body fluid levels	4.87E-13	8.76E-11	64	792
GO:0042060	wound healing	6.10E-13	1.07E-10	66	837
GO:0050817	coagulation	6.24E-13	1.07E-10	64	796
GO:0034622	cellular macromolecular complex assembly	6.89E-13	1.15E-10	103	1611

GO:0009611	response to wounding	9.47E-13	1.54E-10	66	846
GO:0043232	intracellular non-membrane-bounded organelle	1.03E-12	1.64E-10	238	5085
GO:0043228	non-membrane-bounded organelle	3.14E-12	4.89E-10	239	5177
GO:0048193	Golgi vesicle transport	3.24E-12	4.93E-10	69	784
GO:0019898	extrinsic component of membrane	5.90E-12	8.80E-10	64	721
GO:0000921	septin ring assembly	1.63E-11	2.29E-09	45	414
GO:0005940	septin ring	1.63E-11	2.29E-09	45	414
GO:0031106	septin ring organization	1.63E-11	2.29E-09	45	414
GO:0032156	septin cytoskeleton	2.42E-11	3.33E-09	45	420
GO:0032185	septin cytoskeleton organization	2.64E-11	3.56E-09	45	421
GO:0043168	anion binding	2.90E-11	3.84E-09	274	6119
GO:0071822	protein complex subunit organization	6.76E-11	8.78E-09	114	2018
GO:0051015	actin filament binding	8.34E-11	1.06E-08	24	114
GO:0006461	protein complex assembly	2.09E-10	2.61E-08	107	1882
GO:0070271	protein complex biogenesis	2.14E-10	2.63E-08	107	1883
GO:0070925	organelle assembly	2.28E-10	2.76E-08	58	719
GO:0005737	cytoplasm	3.15E-10	3.74E-08	301	7215
GO:0043565	sequence-specific DNA binding	3.67E-10	4.29E-08	83	1314
GO:0098813	nuclear chromosome segregation	6.35E-10	7.31E-08	45	517
GO:0008092	cytoskeletal protein binding	7.41E-10	8.26E-08	98	1613
GO:0044448	cell cortex part	7.42E-10	8.26E-08	50	568
GO:0007155	cell adhesion	7.76E-10	8.51E-08	73	1074
GO:0005102	receptor binding	9.86E-10	1.06E-07	87	1473
GO:0022610	biological adhesion	1.14E-09	1.21E-07	74	1106
GO:0065003	macromolecular complex assembly	1.79E-09	1.87E-07	108	1978
GO:0031514	motile cilium	1.94E-09	1.97E-07	52	671
GO:0045502	dynein binding	1.94E-09	1.97E-07	50	678
GO:0005938	cell cortex	2.15E-09	2.16E-07	51	610
GO:0048870	cell motility	3.19E-09	3.11E-07	64	941
GO:0051674	localization of cell	3.19E-09	3.11E-07	64	941
GO:0030286	dynein complex	3.56E-09	3.42E-07	52	736
GO:0044421	extracellular region part	3.89E-09	3.69E-07	98	1678
GO:0097367	carbohydrate derivative binding	4.25E-09	3.97E-07	231	5204
GO:0032553	ribonucleotide binding	4.44E-09	4.10E-07	225	5042
GO:0005929	cilium	6.97E-09	6.35E-07	52	698
GO:0007059	chromosome segregation	8.84E-09	7.95E-07	48	621
GO:0042802	identical protein binding	1.21E-08	1.08E-06	58	889
GO:0035639	purine ribonucleoside triphosphate binding	1.28E-08	1.10E-06	221	4995
GO:0008134	transcription factor binding	1.28E-08	1.10E-06	61	948
GO:0017076	purine nucleotide binding	1.29E-08	1.10E-06	224	5085
GO:0001883	purine nucleoside binding	1.33E-08	1.10E-06	221	4999
GO:0032550	purine ribonucleoside binding	1.33E-08	1.10E-06	221	4999
GO:0032549	ribonucleoside binding	1.34E-08	1.10E-06	221	5000
GO:0032555	purine ribonucleotide binding	1.40E-08	1.14E-06	221	5003
GO:0001882	nucleoside binding	1.48E-08	1.19E-06	221	5007
GO:0044699	single-organism process	1.59E-08	1.27E-06	689	20106
GO:0043933	macromolecular complex subunit organization	1.63E-08	1.28E-06	118	2336
GO:0019752	carboxylic acid metabolic process	1.93E-08	1.51E-06	146	3111
GO:0044765	single-organism transport	2.05E-08	1.58E-06	228	5396
GO:0046907	intracellular transport	2.15E-08	1.64E-06	136	2651

GO:1902578	single-organism localization	2.71E-08	2.04E-06	232	5531
GO:0051649	establishment of localization in cell	2.89E-08	2.15E-06	142	2827
GO:0044711	single-organism biosynthetic process	3.12E-08	2.30E-06	132	2790
GO:0065008	regulation of biological quality	3.54E-08	2.57E-06	94	1822
GO:0048037	cofactor binding	3.56E-08	2.57E-06	65	1079
GO:0070206	protein trimerization	3.62E-08	2.59E-06	29	242
GO:0006082	organic acid metabolic process	3.71E-08	2.60E-06	147	3171
GO:0043436	oxoacid metabolic process	3.71E-08	2.60E-06	147	3171
GO:0005615	extracellular space	3.87E-08	2.69E-06	67	1092
GO:0045995	regulation of embryonic development	4.19E-08	2.88E-06	22	151
GO:0072686	mitotic spindle	4.41E-08	3.01E-06	27	234
GO:0044422	organelle part	4.47E-08	3.02E-06	278	6813
GO:0042803	protein homodimerization activity	4.54E-08	3.03E-06	50	752
GO:0044446	intracellular organelle part	6.11E-08	4.04E-06	275	6746
GO:0040011	locomotion	6.59E-08	4.32E-06	70	1155
GO:0016875	ligase activity, forming carbon-oxygen bonds	7.85E-08	5.05E-06	26	262
GO:0016876	ligase activity, forming aminoacyl-tRNA and related compounds	7.85E-08	5.05E-06	26	262
GO:0030155	regulation of cell adhesion	8.22E-08	5.22E-06	21	144
GO:0010639	negative regulation of organelle organization	8.27E-08	5.22E-06	33	362
GO:0006928	movement of cell or subcellular component	8.34E-08	5.22E-06	71	1185
GO:0032434	regulation of proteasomal ubiquitin-dependent protein catabolic process	9.81E-08	6.03E-06	31	324
GO:0061136	regulation of proteasomal protein catabolic process	9.81E-08	6.03E-06	31	324
GO:0005875	microtubule associated complex	1.01E-07	6.15E-06	52	809
GO:0031329	regulation of cellular catabolic process	1.08E-07	6.54E-06	50	754
GO:0043038	amino acid activation	1.12E-07	6.58E-06	26	267
GO:0043039	tRNA aminoacylation	1.12E-07	6.58E-06	26	267
GO:1903050	regulation of proteolysis involved in cellular protein catabolic process	1.13E-07	6.58E-06	31	326
GO:1903362	regulation of cellular protein catabolic process	1.13E-07	6.58E-06	31	326
GO:0004812	aminoacyl-tRNA ligase activity	1.21E-07	6.82E-06	25	247
GO:0000778	condensed nuclear chromosome kinetochore	1.22E-07	6.82E-06	28	273
GO:0000780	condensed nuclear chromosome, centromeric region	1.22E-07	6.82E-06	28	273
GO:0000942	condensed nuclear chromosome outer kinetochore	1.22E-07	6.82E-06	28	273
GO:0042729	DASH complex	1.22E-07	6.82E-06	28	273
GO:0044085	cellular component biogenesis	1.50E-07	8.33E-06	153	3402
GO:0007010	cytoskeleton organization	1.60E-07	8.81E-06	81	1424
GO:0051129	negative regulation of cellular component organization	1.61E-07	8.83E-06	33	378
GO:0042995	cell projection	1.66E-07	8.89E-06	57	900
GO:0006418	tRNA aminoacylation for protein translation	1.73E-07	8.89E-06	25	252
GO:0008608	attachment of spindle microtubules to kinetochore	1.77E-07	8.89E-06	23	198

GO:1901988	negative regulation of cell cycle phase transition	1.81E-07	8.89E-06	29	293
GO:1901991	negative regulation of mitotic cell cycle phase transition	1.81E-07	8.89E-06	29	293
GO:0032435	negative regulation of proteasomal ubiquitin-dependent protein catabolic process	1.83E-07	8.89E-06	29	293
GO:0042177	negative regulation of protein catabolic process	1.83E-07	8.89E-06	29	293
GO:1901799	negative regulation of proteasomal protein catabolic process	1.83E-07	8.89E-06	29	293
GO:1903051	negative regulation of proteolysis involved in cellular protein catabolic process	1.83E-07	8.89E-06	29	293
GO:1903363	negative regulation of cellular protein catabolic process	1.83E-07	8.89E-06	29	293
GO:1901987	regulation of cell cycle phase transition	1.87E-07	8.89E-06	31	334
GO:1901990	regulation of mitotic cell cycle phase transition	1.87E-07	8.89E-06	31	334
GO:0007091	metaphase/anaphase transition of mitotic cell cycle	1.90E-07	8.89E-06	30	316
GO:0010965	regulation of mitotic sister chromatid separation	1.90E-07	8.89E-06	30	316
GO:0030071	regulation of mitotic metaphase/anaphase transition	1.90E-07	8.89E-06	30	316
GO:0033045	regulation of sister chromatid segregation	1.90E-07	8.89E-06	30	316
GO:0033047	regulation of mitotic sister chromatid segregation	1.90E-07	8.89E-06	30	316
GO:0044784	metaphase/anaphase transition of cell cycle	1.90E-07	8.89E-06	30	316
GO:0051306	mitotic sister chromatid separation	1.90E-07	8.89E-06	30	316
GO:0051983	regulation of chromosome segregation	1.90E-07	8.89E-06	30	316
GO:1902099	regulation of metaphase/anaphase transition of cell cycle	1.90E-07	8.89E-06	30	316
GO:0022607	cellular component assembly	1.90E-07	8.89E-06	123	2567
GO:0051641	cellular localization	1.98E-07	9.21E-06	145	3012
GO:0000940	condensed chromosome outer kinetochore	2.00E-07	9.22E-06	31	336
GO:0051348	negative regulation of transferase activity	2.06E-07	9.43E-06	29	298
GO:0009895	negative regulation of catabolic process	2.08E-07	9.43E-06	29	295
GO:0031330	negative regulation of cellular catabolic process	2.08E-07	9.43E-06	29	295
GO:0051304	chromosome separation	2.39E-07	1.08E-05	30	320
GO:0005819	spindle	2.60E-07	1.16E-05	38	465
GO:0010948	negative regulation of cell cycle process	2.64E-07	1.17E-05	29	300
GO:0034453	microtubule anchoring	2.82E-07	1.24E-05	23	204
GO:0030554	adenyl nucleotide binding	3.01E-07	1.32E-05	186	4203
GO:0033044	regulation of chromosome organization	3.08E-07	1.34E-05	30	323
GO:0007088	regulation of mitotic nuclear division	3.09E-07	1.34E-05	30	323
GO:0000070	mitotic sister chromatid segregation	3.14E-07	1.34E-05	30	324

GO:0000819	sister chromatid segregation	3.14E-07	1.34E-05	30	324
GO:0031145	anaphase-promoting complex-dependent proteasomal ubiquitin-dependent protein catabolic process	3.25E-07	1.38E-05	30	323
GO:0051783	regulation of nuclear division	3.36E-07	1.42E-05	30	325
GO:0009894	regulation of catabolic process	3.38E-07	1.42E-05	50	773
GO:0007094	mitotic spindle assembly checkpoint	3.74E-07	1.43E-05	28	286
GO:0031577	spindle checkpoint	3.74E-07	1.43E-05	28	286
GO:0033046	negative regulation of sister chromatid segregation	3.74E-07	1.43E-05	28	286
GO:0033048	negative regulation of mitotic sister chromatid segregation	3.74E-07	1.43E-05	28	286
GO:0045839	negative regulation of mitotic nuclear division	3.74E-07	1.43E-05	28	286
GO:0045841	negative regulation of mitotic metaphase/anaphase transition	3.74E-07	1.43E-05	28	286
GO:0051352	negative regulation of ligase activity	3.74E-07	1.43E-05	28	286
GO:0051436	negative regulation of ubiquitin-protein ligase activity involved in mitotic cell cycle	3.74E-07	1.43E-05	28	286
GO:0051444	negative regulation of ubiquitin-protein transferase activity	3.74E-07	1.43E-05	28	286
GO:0051985	negative regulation of chromosome segregation	3.74E-07	1.43E-05	28	286
GO:0071173	spindle assembly checkpoint	3.74E-07	1.43E-05	28	286
GO:0071174	mitotic spindle checkpoint	3.74E-07	1.43E-05	28	286
GO:1902100	negative regulation of metaphase/anaphase transition of cell cycle	3.74E-07	1.43E-05	28	286
GO:1904667	negative regulation of ubiquitin protein ligase activity	3.74E-07	1.43E-05	28	286
GO:2000816	negative regulation of mitotic sister chromatid separation	3.74E-07	1.43E-05	28	286
GO:2001251	negative regulation of chromosome organization	3.74E-07	1.43E-05	28	286
GO:0042176	regulation of protein catabolic process	3.96E-07	1.51E-05	31	345
GO:0031397	negative regulation of protein ubiquitination	4.02E-07	1.52E-05	28	287
GO:1903321	negative regulation of protein modification by small protein conjugation or removal	4.02E-07	1.52E-05	28	287
GO:0051784	negative regulation of nuclear division	4.09E-07	1.53E-05	28	288
GO:0051782	negative regulation of cell division	4.25E-07	1.58E-05	28	289
GO:0033043	regulation of organelle organization	4.29E-07	1.59E-05	40	540
GO:0051439	regulation of ubiquitin-protein ligase activity involved in mitotic cell cycle	5.23E-07	1.92E-05	28	291
GO:1904666	regulation of ubiquitin protein ligase activity	5.23E-07	1.92E-05	28	291
GO:0007093	mitotic cell cycle checkpoint	5.33E-07	1.95E-05	29	312
GO:0016874	ligase activity	5.36E-07	1.95E-05	45	668
GO:0051340	regulation of ligase activity	6.00E-07	2.16E-05	28	293
GO:0051438	regulation of ubiquitin-protein transferase activity	6.00E-07	2.16E-05	28	293

GO:0005622	intracellular	6.14E-07	2.20E-05	535	15206
GO:0050662	coenzyme binding	6.41E-07	2.27E-05	49	806
GO:0031396	regulation of protein ubiquitination	6.43E-07	2.27E-05	28	294
GO:1903320	regulation of protein modification by small protein conjugation or removal	6.43E-07	2.27E-05	28	294
GO:0044770	cell cycle phase transition	6.70E-07	2.34E-05	31	358
GO:0044772	mitotic cell cycle phase transition	6.70E-07	2.34E-05	31	358
GO:0000777	condensed chromosome kinetochore	6.84E-07	2.36E-05	31	355
GO:0000779	condensed chromosome, centromeric region	6.84E-07	2.36E-05	31	355
GO:0000794	condensed nuclear chromosome	6.96E-07	2.39E-05	29	330
GO:0005524	ATP binding	7.03E-07	2.39E-05	183	4129
GO:0016192	vesicle-mediated transport	7.03E-07	2.39E-05	89	1506
GO:0044763	single-organism cellular process	7.13E-07	2.42E-05	570	16473
GO:0032559	adenyl ribonucleotide binding	7.53E-07	2.54E-05	183	4135
GO:0045930	negative regulation of mitotic cell cycle	8.30E-07	2.78E-05	29	319
GO:0006886	intracellular protein transport	8.70E-07	2.91E-05	110	2177
GO:0010564	regulation of cell cycle process	8.84E-07	2.94E-05	32	388
GO:0001071	nucleic acid binding transcription factor activity	9.03E-07	2.97E-05	92	1824
GO:0003700	transcription factor activity, sequence-specific DNA binding	9.03E-07	2.97E-05	92	1824
GO:0044707	single-multicellular organism process	9.06E-07	2.97E-05	106	2159
GO:0031400	negative regulation of protein modification process	9.46E-07	3.09E-05	29	317
GO:0009058	biosynthetic process	9.80E-07	3.18E-05	364	9886
GO:0051302	regulation of cell division	1.13E-06	3.66E-05	31	374
GO:0010498	proteasomal protein catabolic process	1.24E-06	3.96E-05	33	404
GO:0043161	proteasome-mediated ubiquitin-dependent protein catabolic process	1.24E-06	3.96E-05	33	404
GO:0030334	regulation of cell migration	1.74E-06	5.54E-05	21	174
GO:0003779	actin binding	1.92E-06	6.10E-05	43	596
GO:0045861	negative regulation of proteolysis	1.95E-06	6.16E-05	30	351
GO:0000776	kinetochore	1.99E-06	6.27E-05	36	487
GO:1901576	organic substance biosynthetic process	2.03E-06	6.31E-05	354	9629
GO:0051270	regulation of cellular component movement	2.03E-06	6.31E-05	21	177
GO:2000145	regulation of cell motility	2.03E-06	6.31E-05	21	177
GO:0016491	oxidoreductase activity	2.11E-06	6.51E-05	131	3136
GO:0005667	transcription factor complex	2.12E-06	6.51E-05	121	2661
GO:0009790	embryo development	2.16E-06	6.61E-05	29	324
GO:0015630	microtubule cytoskeleton	2.39E-06	7.27E-05	74	1396
GO:0006559	L-phenylalanine catabolic process	2.55E-06	7.72E-05	4	5
GO:1902222	erythrose 4-phosphate/phosphoenolpyruvate family amino acid catabolic process	2.55E-06	7.72E-05	4	5
GO:0008565	protein transporter activity	2.69E-06	8.10E-05	36	433
GO:0040012	regulation of locomotion	2.76E-06	8.28E-05	21	181
GO:0007346	regulation of mitotic cell cycle	2.82E-06	8.42E-05	31	392
GO:0003824	catalytic activity	2.88E-06	8.54E-05	627	18458
GO:0015031	protein transport	3.53E-06	0.000105	125	2645
GO:0030162	regulation of proteolysis	3.58E-06	0.000105	32	408
GO:0005623	cell	3.70E-06	0.000108	563	16218

GO:0044464	cell part	3.70E-06	0.000108	563	16218
GO:0043086	negative regulation of catalytic activity	3.88E-06	0.000113	30	367
GO:0045184	establishment of protein localization	4.18E-06	0.000121	126	2682
GO:0000226	microtubule cytoskeleton organization	4.37E-06	0.000126	44	649
GO:0032269	negative regulation of cellular protein metabolic process	4.91E-06	0.000141	32	408
GO:0051248	negative regulation of protein metabolic process	4.91E-06	0.000141	32	408
GO:0055114	oxidation-reduction process	5.01E-06	0.000143	133	3215
GO:0044424	intracellular part	5.14E-06	0.000146	509	14542
GO:0043234	protein complex	5.43E-06	0.000154	345	9192
GO:0044092	negative regulation of molecular function	5.84E-06	0.000164	30	379
GO:0032501	multicellular organismal process	6.17E-06	0.000173	109	2308
GO:0000075	cell cycle checkpoint	6.29E-06	0.000176	31	401
GO:0006520	cellular amino acid metabolic process	6.77E-06	0.000188	108	2337
GO:0016477	cell migration	7.62E-06	0.000211	24	248
GO:0032879	regulation of localization	7.90E-06	0.000218	28	316
GO:0034613	cellular protein localization	1.07E-05	0.000293	113	2369
GO:0044454	nuclear chromosome part	1.08E-05	0.000295	29	377
GO:0044712	single-organism catabolic process	1.11E-05	0.000304	70	1325
GO:0070727	cellular macromolecule localization	1.12E-05	0.000304	113	2371
GO:0045786	negative regulation of cell cycle	1.15E-05	0.00031	30	392
GO:0044249	cellular biosynthetic process	1.18E-05	0.000318	334	9143
GO:0000793	condensed chromosome	1.23E-05	0.000332	32	438
GO:0034660	ncRNA metabolic process	1.26E-05	0.000336	53	944
GO:1901363	heterocyclic compound binding	1.34E-05	0.000356	477	13561
GO:0097159	organic cyclic compound binding	1.39E-05	0.000368	477	13566
GO:0050793	regulation of developmental process	1.44E-05	0.000381	28	335
GO:0051338	regulation of transferase activity	1.46E-05	0.000386	33	466
GO:2000026	regulation of multicellular organismal development	1.51E-05	0.000395	24	259
GO:0005816	spindle pole body	1.60E-05	0.000418	13	86
GO:0005823	central plaque of spindle pole body	1.60E-05	0.000418	13	86
GO:0006091	generation of precursor metabolites and energy	1.97E-05	0.000512	60	1181
GO:0044444	cytoplasmic part	2.04E-05	0.000527	210	5293
GO:0019031	viral envelope	2.29E-05	0.000589	49	859
GO:0036338	viral membrane	2.29E-05	0.000589	49	859
GO:0051259	protein oligomerization	2.40E-05	0.000614	31	401
GO:0044281	small molecule metabolic process	2.46E-05	0.000628	226	5774
GO:0008104	protein localization	2.54E-05	0.000645	128	2836
GO:0000775	chromosome, centromeric region	2.58E-05	0.000654	39	610
GO:0000228	nuclear chromosome	2.61E-05	0.000658	29	398
GO:0051607	defense response to virus	2.71E-05	0.000681	22	289
GO:0006399	tRNA metabolic process	2.89E-05	0.000723	45	782
GO:0006733	oxidoreduction coenzyme metabolic process	3.20E-05	0.0008	41	724
GO:0051239	regulation of multicellular organismal process	3.44E-05	0.000855	26	321
GO:0009615	response to virus	3.51E-05	0.000871	24	333
GO:0002252	immune effector process	4.00E-05	0.000989	22	298

GO:1901566	organonitrogen compound biosynthetic process	4.08E-05	0.001004	118	2722
GO:0006694	steroid biosynthetic process	4.55E-05	0.001117	13	124
GO:0098687	chromosomal region	4.58E-05	0.00112	39	627
GO:0007049	cell cycle	4.81E-05	0.001172	89	1964
GO:0046983	protein dimerization activity	5.13E-05	0.001245	57	1105
GO:0006914	autophagy	5.49E-05	0.001327	62	1208
GO:0006810	transport	5.65E-05	0.001361	286	7641
GO:0004411	homogentisate 1,2-dioxygenase activity	6.05E-05	0.001453	3	4
GO:0051726	regulation of cell cycle	6.59E-05	0.001577	35	565
GO:0008610	lipid biosynthetic process	7.35E-05	0.001753	38	674
GO:0044450	microtubule organizing center part	7.74E-05	0.00184	13	100
GO:0032991	macromolecular complex	8.16E-05	0.001934	368	10211
GO:0006511	ubiquitin-dependent protein catabolic process	8.37E-05	0.001972	40	643
GO:0008924	malate dehydrogenase (quinone) activity	8.40E-05	0.001972	4	10
GO:0051234	establishment of localization	8.41E-05	0.001972	286	7682
GO:0044257	cellular protein catabolic process	8.59E-05	0.002001	41	670
GO:0051603	proteolysis involved in cellular protein catabolic process	8.59E-05	0.002001	41	670
GO:0044710	single-organism metabolic process	8.71E-05	0.002023	370	10538
GO:0043229	intracellular organelle	8.96E-05	0.002074	396	11184
GO:0000287	magnesium ion binding	9.02E-05	0.002082	26	379
GO:0005741	mitochondrial outer membrane	0.00011	0.002415	19	240
GO:0019941	modification-dependent protein catabolic process	0.00011	0.002471	40	651
GO:0043632	modification-dependent macromolecule catabolic process	0.00011	0.002471	40	651
GO:0031968	organelle outer membrane	0.00012	0.002631	19	242
GO:0050660	flavin adenine dinucleotide binding	0.00012	0.002815	20	274
GO:0042262	DNA protection	0.00013	0.002953	8	61
GO:0000922	spindle pole	0.00014	0.003102	14	125
GO:0006412	translation	0.00014	0.003136	56	1128
GO:0016614	oxidoreductase activity, acting on CH-OH group of donors	0.00015	0.003257	24	376
GO:0044283	small molecule biosynthetic process	0.00015	0.003293	57	1155
GO:0043226	organelle	0.00016	0.003579	398	11329
GO:0033036	macromolecule localization	0.00017	0.003861	137	3220
GO:0032268	regulation of cellular protein metabolic process	0.00018	0.003874	54	1033
GO:0051179	localization	0.00018	0.00392	298	8123
GO:0005200	structural constituent of cytoskeleton	0.00018	0.00403	13	111
GO:0031399	regulation of protein modification process	0.00019	0.004055	33	530
GO:0061061	muscle structure development	0.00019	0.004081	10	77
GO:0009074	aromatic amino acid family catabolic process	0.0002	0.004299	5	18
GO:0072524	pyridine-containing compound metabolic process	0.0002	0.004325	33	573
GO:0030163	protein catabolic process	0.0002	0.004377	41	697
GO:0019362	pyridine nucleotide metabolic process	0.00021	0.004456	32	550
GO:0043603	cellular amide metabolic process	0.00022	0.00473	74	1613

GO:0005801	cis-Golgi network	0.00023	0.004869	17	209
GO:0006518	peptide metabolic process	0.00025	0.005243	59	1241
GO:0016901	oxidoreductase activity, acting on the CH-OH group of donors, quinone or similar compound as acceptor	0.00025	0.005306	4	12
GO:0007067	mitotic nuclear division	0.00026	0.005537	34	556
GO:0016053	organic acid biosynthetic process	0.00027	0.00564	44	832
GO:0046394	carboxylic acid biosynthetic process	0.00027	0.00564	44	832
GO:0051246	regulation of protein metabolic process	0.00028	0.005852	54	1052
GO:0022402	cell cycle process	0.00028	0.005899	68	1435
GO:0008202	steroid metabolic process	0.00029	0.006077	13	146
GO:0009605	response to external stimulus	0.00032	0.006595	43	855
GO:0042438	melanin biosynthetic process	0.00032	0.006595	5	19
GO:0046189	phenol-containing compound biosynthetic process	0.00032	0.006595	5	19
GO:1901605	alpha-amino acid metabolic process	0.00033	0.006897	92	2112
GO:0032403	protein complex binding	0.00034	0.006963	44	791
GO:0006558	L-phenylalanine metabolic process	0.00034	0.007021	14	165
GO:1902221	erythrose 4-phosphate/phosphoenolpyruvate family amino acid metabolic process	0.00034	0.007021	14	165
GO:0043043	peptide biosynthetic process	0.00035	0.007172	56	1171
GO:1901564	organonitrogen compound metabolic process	0.00035	0.007172	249	6717
GO:0007017	microtubule-based process	0.00036	0.007302	46	836
GO:0006099	tricarboxylic acid cycle	0.00038	0.007666	13	141
GO:0006101	citrate metabolic process	0.00038	0.007666	13	141
GO:0008855	exodeoxyribonuclease VII activity	0.00039	0.007871	29	466
GO:0009318	exodeoxyribonuclease VII complex	0.00039	0.007871	29	466
GO:0051128	regulation of cellular component organization	0.00039	0.007871	41	773
GO:0072350	tricarboxylic acid metabolic process	0.00041	0.008157	13	142
GO:0004529	exodeoxyribonuclease activity	0.00041	0.008157	29	468
GO:0016895	exodeoxyribonuclease activity, producing 5'-phosphomonoesters	0.00041	0.008157	29	468
GO:0016787	hydrolase activity	0.00043	0.008539	313	8525
GO:0008537	proteasome activator complex	0.00044	0.008585	7	40
GO:0006090	pyruvate metabolic process	0.00044	0.008599	31	556
GO:0010830	regulation of myotube differentiation	0.00047	0.009135	3	5
GO:0051147	regulation of muscle cell differentiation	0.00047	0.009135	3	5
GO:0051153	regulation of striated muscle cell differentiation	0.00047	0.009135	3	5
GO:0000280	nuclear division	0.00048	0.009263	36	632
GO:0005740	mitochondrial envelope	0.00048	0.009282	36	659
GO:0005576	extracellular region	0.00049	0.009476	145	3600
GO:0005815	microtubule organizing center	0.00051	0.009729	23	322
GO:0032787	monocarboxylic acid metabolic process	0.00051	0.009824	50	1028
GO:0005850	eukaryotic translation initiation factor 2 complex	0.00052	0.009989	6	31
GO:0072509	divalent inorganic cation transmembrane transporter activity	0.00053	0.010059	26	409
GO:1902589	single-organism organelle organization	0.00053	0.010063	82	1839
GO:0048856	anatomical structure development	0.00056	0.010696	58	1180

GO:0071840	cellular component organization or biogenesis	0.00058	0.010912	205	5478
GO:0031975	envelope	0.00066	0.012418	43	837
GO:0022892	substrate-specific transporter activity	0.00066	0.012418	113	2760
GO:0043604	amide biosynthetic process	0.00071	0.013399	65	1428
GO:0048285	organelle fission	0.00073	0.01362	36	645
GO:0000746	conjugation	0.00075	0.013969	9	91
GO:0031967	organelle envelope	0.00075	0.013988	41	791
GO:0009060	aerobic respiration	0.00076	0.014047	13	153
GO:0014902	myotube differentiation	0.00082	0.015185	3	6
GO:0044248	cellular catabolic process	0.00083	0.015345	114	2758
GO:0016043	cellular component organization	0.00084	0.015524	179	4708
GO:0051287	NAD binding	0.00089	0.016313	18	270
GO:0044429	mitochondrial part	0.00089	0.016335	39	761
GO:0071702	organic substance transport	0.00092	0.016731	140	3512
GO:0010508	positive regulation of autophagy	0.00092	0.016838	24	405
GO:0004448	isocitrate dehydrogenase activity	0.00093	0.016899	4	22
GO:0031324	negative regulation of cellular metabolic process	0.00093	0.016899	33	582
GO:0019829	cation-transporting ATPase activity	0.00097	0.017502	13	164
GO:0031023	microtubule organizing center organization	0.00098	0.017707	17	213
GO:0008299	isoprenoid biosynthetic process	0.00099	0.017736	13	164
GO:0016114	terpenoid biosynthetic process	0.00099	0.017766	12	141
GO:0009085	lysine biosynthetic process	0.00099	0.017767	5	31
GO:0010605	negative regulation of macromolecule metabolic process	0.00101	0.01802	34	609
GO:0044877	macromolecular complex binding	0.00103	0.018358	52	1033
GO:0005739	mitochondrion	0.00104	0.018478	47	995
GO:0030199	collagen fibril organization	0.00104	0.018478	4	17
GO:1901607	alpha-amino acid biosynthetic process	0.00106	0.018704	28	486
GO:0022624	proteasome accessory complex	0.00106	0.018762	7	47
GO:0006582	melanin metabolic process	0.00109	0.019074	5	25
GO:0051276	chromosome organization	0.00113	0.019802	54	1148
GO:0051301	cell division	0.00117	0.020395	44	876
GO:0070838	divalent metal ion transport	0.00121	0.021064	30	504
GO:0009896	positive regulation of catabolic process	0.00122	0.02121	24	413
GO:0031331	positive regulation of cellular catabolic process	0.00122	0.02121	24	413
GO:0044265	cellular macromolecule catabolic process	0.00122	0.02121	61	1314
GO:0009892	negative regulation of metabolic process	0.00123	0.02123	34	617
GO:0030522	intracellular receptor signaling pathway	0.00126	0.021752	6	37
GO:0044271	cellular nitrogen compound biosynthetic process	0.00131	0.022531	252	7095
GO:0031076	embryonic camera-type eye development	0.00132	0.022531	3	10
GO:0048048	embryonic eye morphogenesis	0.00132	0.022531	3	10
GO:0048596	embryonic camera-type eye morphogenesis	0.00132	0.022531	3	10
GO:0006721	terpenoid metabolic process	0.00133	0.022531	12	147

GO:0016616	oxidoreductase activity, acting on the CH-OH group of donors, NAD or NADP as acceptor	0.00133	0.022531	20	343
GO:0098542	defense response to other organism	0.00135	0.02292	26	474
GO:0009056	catabolic process	0.00138	0.023238	134	3422
GO:0031966	mitochondrial membrane	0.00138	0.023238	33	628
GO:1901362	organic cyclic compound biosynthetic process	0.00139	0.023371	225	6232
GO:0006996	organelle organization	0.0014	0.023556	118	2918
GO:0006081	cellular aldehyde metabolic process	0.00141	0.023675	23	398
GO:0015631	tubulin binding	0.00143	0.023947	33	579
GO:1901617	organic hydroxy compound biosynthetic process	0.00145	0.024218	8	72
GO:0004506	squalene monooxygenase activity	0.00149	0.024595	4	19
GO:0015988	energy coupled proton transmembrane transport, against electrochemical gradient	0.00149	0.024595	24	410
GO:0015991	ATP hydrolysis coupled proton transport	0.00149	0.024595	24	410
GO:0006732	coenzyme metabolic process	0.00152	0.025032	54	1202
GO:0008442	3-hydroxyisobutyrate dehydrogenase activity	0.00155	0.025444	4	17
GO:0005093	Rab GDP-dissociation inhibitor activity	0.00161	0.026515	3	9
GO:0030976	thiamine pyrophosphate binding	0.00162	0.026551	9	81
GO:0046497	nicotinate nucleotide metabolic process	0.00168	0.027465	6	40
GO:0046496	nicotinamide nucleotide metabolic process	0.00168	0.027465	28	527
GO:0015093	ferrous iron transmembrane transporter activity	0.00176	0.028419	15	202
GO:0015684	ferrous iron transport	0.00176	0.028419	15	202
GO:0043207	response to external biotic stimulus	0.00176	0.028419	28	531
GO:0051707	response to other organism	0.00176	0.028419	28	531
GO:0004749	ribose phosphate diphosphokinase activity	0.00178	0.028758	3	12
GO:0016796	exonuclease activity, active with either ribo- or deoxyribonucleic acids and producing 5'-phosphomonoesters	0.00179	0.028782	30	538
GO:0046933	proton-transporting ATP synthase activity, rotational mechanism	0.00179	0.028782	7	67
GO:0090407	organophosphate biosynthetic process	0.00181	0.028835	36	721
GO:0008652	cellular amino acid biosynthetic process	0.00181	0.028835	28	509
GO:0045298	tubulin complex	0.00181	0.028835	33	590
GO:0044550	secondary metabolite biosynthetic process	0.00181	0.028835	6	44
GO:0008184	glycogen phosphorylase activity	0.00182	0.028842	8	65
GO:0098799	outer mitochondrial membrane protein complex	0.00187	0.029291	8	80
GO:0004883	glucocorticoid receptor activity	0.00188	0.029291	4	14
GO:0031958	corticosteroid receptor signaling pathway	0.00188	0.029291	4	14
GO:0031960	response to corticosteroid	0.00188	0.029291	4	14

GO:0042921	glucocorticoid receptor signaling pathway	0.00188	0.029291	4	14
GO:0043402	glucocorticoid mediated signaling pathway	0.00188	0.029291	4	14
GO:0051384	response to glucocorticoid	0.00188	0.029291	4	14
GO:0071384	cellular response to corticosteroid stimulus	0.00188	0.029291	4	14
GO:0071385	cellular response to glucocorticoid stimulus	0.00188	0.029291	4	14
GO:0004645	phosphorylase activity	0.00193	0.029865	8	66
GO:0015075	ion transmembrane transporter activity	0.00193	0.029865	88	2140
GO:0009165	nucleotide biosynthetic process	0.00202	0.031149	25	453
GO:1901293	nucleoside phosphate biosynthetic process	0.00202	0.031149	25	453
GO:0016701	oxidoreductase activity, acting on single donors with incorporation of molecular oxygen	0.00209	0.032159	11	124
GO:0016778	diphosphotransferase activity	0.0021	0.032168	3	13
GO:0016887	ATPase activity	0.00212	0.032406	47	968
GO:0015980	energy derivation by oxidation of organic compounds	0.00213	0.032525	28	547
GO:0006720	isoprenoid metabolic process	0.00214	0.032677	13	180
GO:0033179	proton-transporting V-type ATPase, V0 domain	0.00215	0.032743	18	277
GO:0030474	spindle pole body duplication	0.00219	0.033212	13	146
GO:0051300	spindle pole body organization	0.00219	0.033212	13	146
GO:0003707	steroid hormone receptor activity	0.0022	0.033317	6	42
GO:0019867	outer membrane	0.00221	0.033332	27	502
GO:0071944	cell periphery	0.0023	0.034582	86	1977
GO:1903047	mitotic cell cycle process	0.0023	0.034582	44	905
GO:0000278	mitotic cell cycle	0.0024	0.035906	44	907
GO:0030198	extracellular matrix organization	0.00253	0.03779	4	21
GO:0043062	extracellular structure organization	0.00253	0.03779	4	21
GO:0003842	1-pyrroline-5-carboxylate dehydrogenase activity	0.00255	0.037968	2	3
GO:0042625	ATPase activity, coupled to transmembrane movement of ions	0.00257	0.038146	13	180
GO:0043401	steroid hormone mediated signaling pathway	0.0026	0.038362	7	61
GO:0048545	response to steroid hormone	0.0026	0.038362	7	61
GO:0071383	cellular response to steroid hormone stimulus	0.0026	0.038362	7	61
GO:0008037	cell recognition	0.00276	0.040675	4	25
GO:0072511	divalent inorganic cation transport	0.00277	0.040713	30	535
GO:0009067	aspartate family amino acid biosynthetic process	0.00286	0.041917	6	53
GO:0018966	styrene metabolic process	0.00289	0.042161	4	20
GO:0042207	styrene catabolic process	0.00289	0.042161	4	20
GO:1900673	olefin metabolic process	0.00289	0.042161	4	20
GO:1901575	organic substance catabolic process	0.00292	0.042501	104	2588
GO:0044445	cytosolic part	0.00293	0.042537	17	261
GO:0071396	cellular response to lipid	0.00302	0.043802	7	64
GO:0005829	cytosol	0.00314	0.045338	22	384

GO:0009607	response to biotic stimulus	0.00317	0.045531	31	614
GO:0051186	cofactor metabolic process	0.00318	0.045531	58	1347
GO:0009064	glutamine family amino acid metabolic process	0.00319	0.045531	19	320
GO:0004656	procollagen-proline 4-dioxygenase activity	0.00322	0.045531	5	27
GO:0018126	protein hydroxylation	0.00322	0.045531	5	27
GO:0018401	peptidyl-proline hydroxylation to 4-hydroxy-L-proline	0.00322	0.045531	5	27
GO:0019471	4-hydroxyproline metabolic process	0.00322	0.045531	5	27
GO:0019511	peptidyl-proline hydroxylation	0.00322	0.045531	5	27
GO:0019798	procollagen-proline dioxygenase activity	0.00322	0.045531	5	27
GO:0031543	peptidyl-proline dioxygenase activity	0.00322	0.045531	5	27
GO:0031545	peptidyl-proline 4-dioxygenase activity	0.00322	0.045531	5	27
GO:0034645	cellular macromolecule biosynthetic process	0.00339	0.0478	256	7323
GO:0042537	benzene-containing compound metabolic process	0.00346	0.048794	10	119
GO:0055086	nucleobase-containing small molecule metabolic process	0.00348	0.048912	91	2365
GO:0006753	nucleoside phosphate metabolic process	0.0035	0.049058	65	1608
GO:0051146	striated muscle cell differentiation	0.00357	0.049953	6	42
