# Supplementary figures and images for: Gene Expression Program Underlying Tail Resorption During Thyroid Hormone-Dependent Metamorphosis of the Ornamented Pygmy Frog Microhyla fissipes
Source: Front Endocrinol (Lausanne). 2019 Jan 25;10:11. doi: 10.3389/fendo.2019.00011 (PMC6357680; doi:10.3389/fendo.2019.00011)

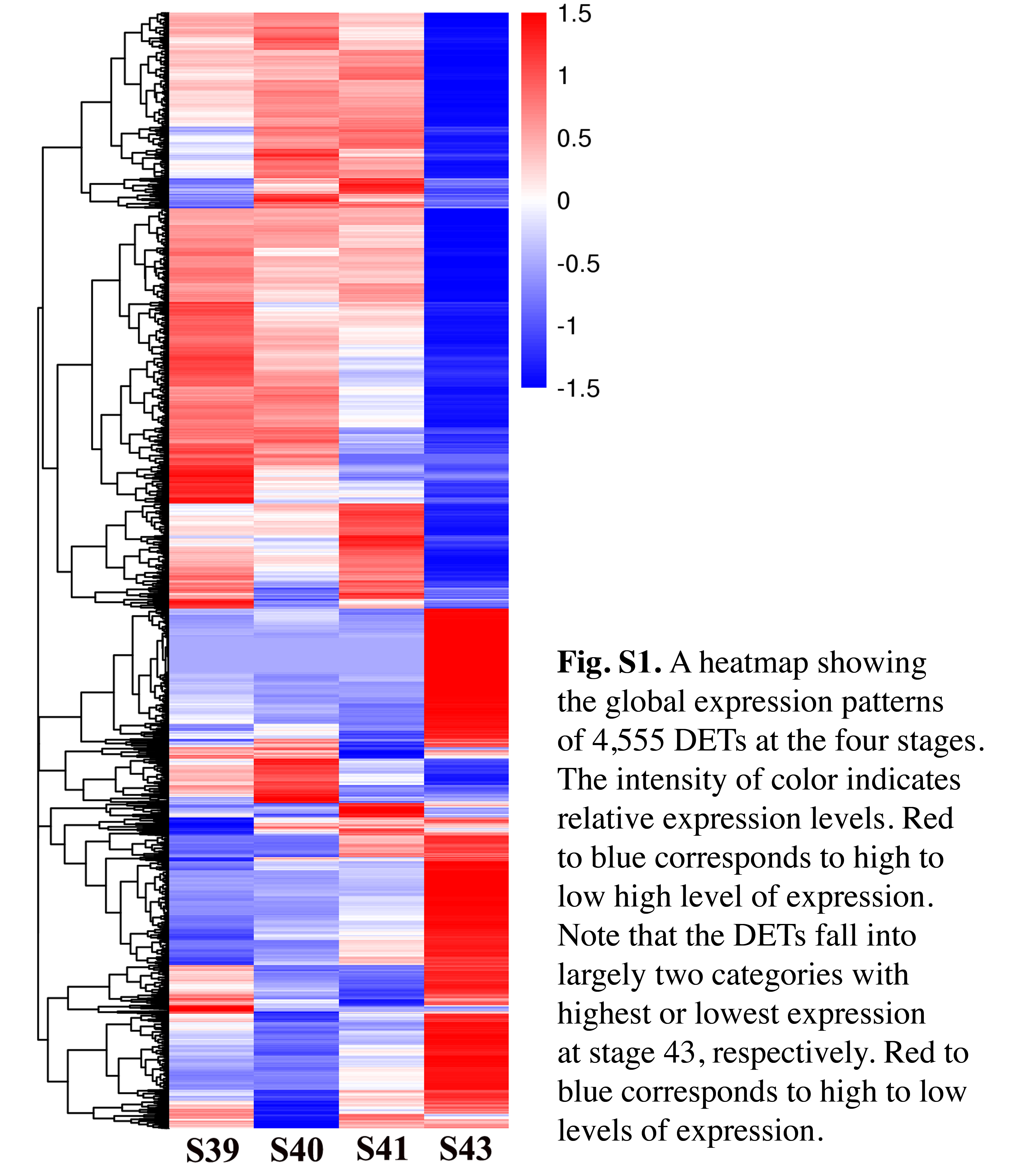

Supplement: Supplementary file 8 [file Image_1.TIF]

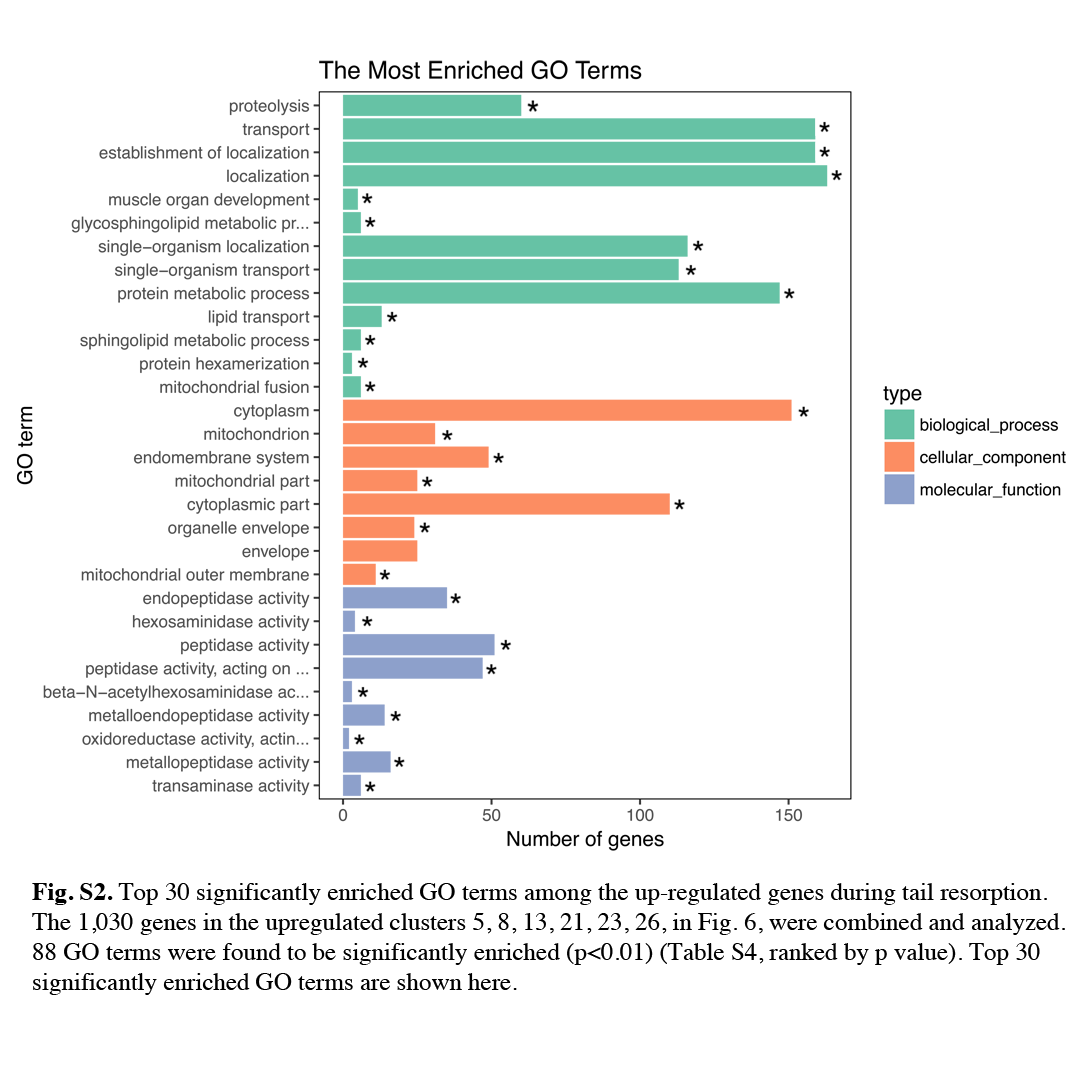

Supplement: Supplementary file 9 [file Image_2.TIF]

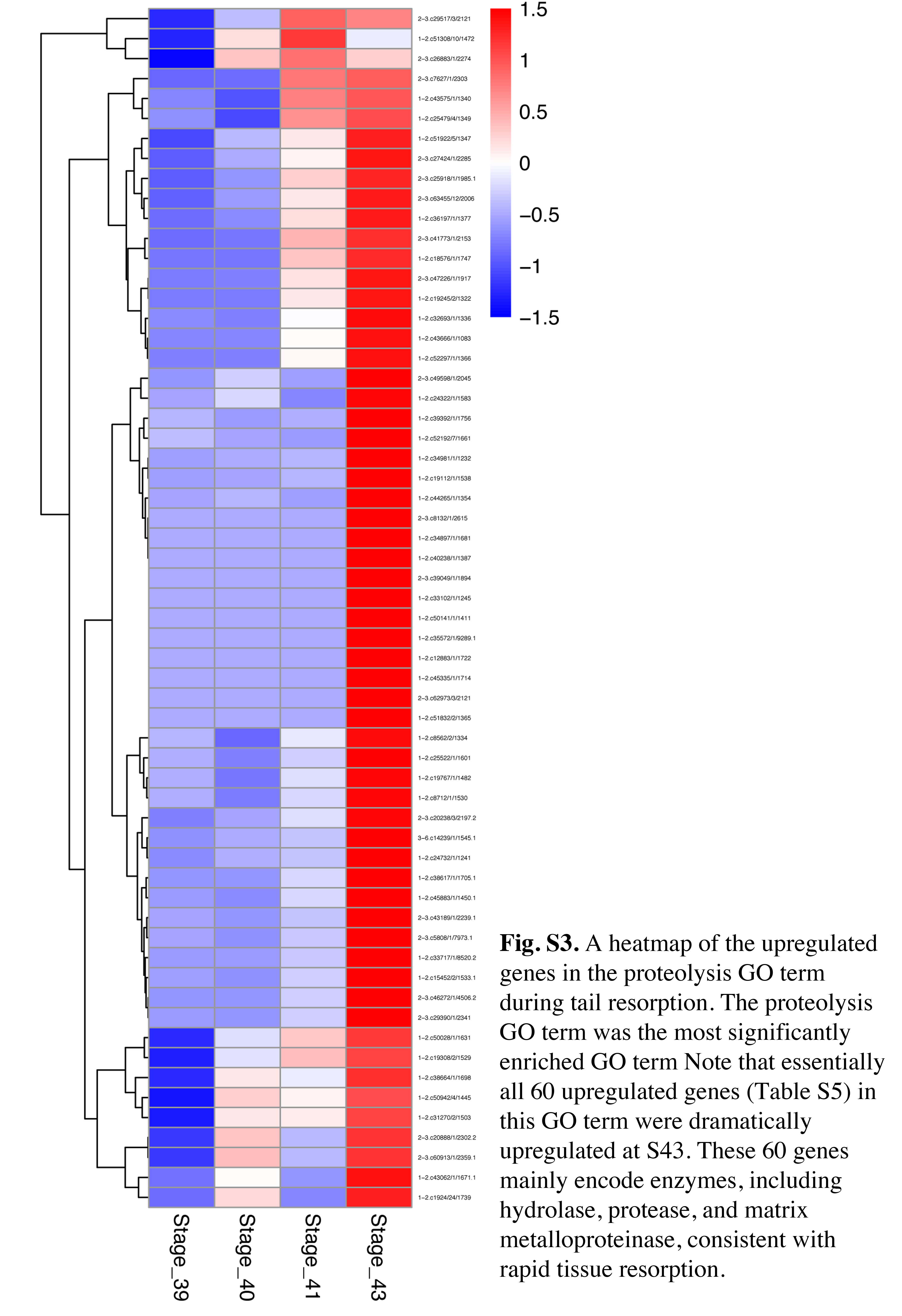

Supplement: Supplementary file 10 [file Image_3.TIF]
